# DNA Methylation at the Neonatal State and at the Time of Diagnosis: Preliminary Support for an Association with the Estrogen Receptor 1, Gamma-Aminobutyric Acid B Receptor 1, and Myelin Oligodendrocyte Glycoprotein in Female Adolescent Patients with OCD

**DOI:** 10.3389/fpsyt.2016.00035

**Published:** 2016-03-18

**Authors:** Judith Becker Nissen, Christine Søholm Hansen, Anna Starnawska, Manuel Mattheisen, Anders Dupont Børglum, Henriette Nørmølle Buttenschøn, Mads Hollegaard

**Affiliations:** ^1^Center of Child and Adolescent Psychiatry (BUC), Aarhus University Hospital, Risskov, Denmark; ^2^Department of Congenital Diseases, Neonatal Genetics, Statens Serum Institut, Copenhagen, Denmark; ^3^Department of Biomedicine, Aarhus University, Aarhus, Denmark; ^4^The Lundbeck Foundation Initiative for Integrative Psychiatric Research (iPSYCH), Aarhus, Denmark; ^5^Centre for Integrative Sequencing (iSEQ), Aarhus, Denmark; ^6^Translational Neuropsychiatry Unit (TNU), Department of Clinical Medicine, Aarhus University, Aarhus, Denmark

**Keywords:** obsessive–compulsive disorder, children, adolescents, epigenetics, estrogen receptor 1, gamma-aminobutyric acid B receptor 1, myelin oligodendrocyte glycoprotein, association

## Abstract

Obsessive–compulsive disorder (OCD) is a neuropsychiatric disorder. Non-genetic factors and their interaction with genes have attracted increasing attention. Epigenetics is regarded an important interface between environmental signals and activation/repression of genomic responses. Epigenetic mechanisms have not previously been examined in OCD in children and adolescents. The aim of the present study was to examine the DNA methylation profile of selected genes in blood spots from neonates later diagnosed with OCD and in the same children/adolescents at the time of diagnosis compared with age- and sex-matched controls. Furthermore, we wanted to characterize the association of the differential methylation profiles with the severity of OCD and treatment outcome. Dried and new blood spot samples were obtained from 21 female children/adolescents with verified OCD and 12 female controls. The differential methylation was analyzed using a linear model and the correlation with the severity of OCD and treatment outcome was analyzed using the Pearson correlation. We evaluated selected Illumina Infinium HumanMethylation450 BeadChip probes within and up to 100,000 bp up- and downstream of 14 genes previously associated with OCD (*SLC1A1, SLC25A12, GABBR1, GAD1, DLGAP1, MOG, BDNF, OLIG2, NTRK2* and *3, ESR1, SL6A4, TPH2*, and *COMT*). The study found no significantly differential methylation. However, preliminary support for a difference was found for the gamma-aminobutyric acid (GABA) B receptor 1 (cg10234998, cg17099072) in blood samples at birth and for the estrogen receptor 1 (*ESR1*) (cg10939667), the myelin oligodendrocyte glycoprotein (*MOG*) (cg16650906), and the brain-derived neurotrophic factor (*BDNF*) (cg14080521) in blood samples at the time of diagnosis. Preliminary support for an association was observed between the methylation profiles of *GABBR1* and *MOG* and baseline severity, treatment effect, and responder status; and between the methylation profile of *ESR1* and baseline severity. To our knowledge, this is the first study to examine the DNA methylation profiles in OCD. The study points towards possible differences in the methylation profiles and suggests a correlation with the severity of OCD. However, the results warrant further studies in larger sample sets.

## Introduction

Obsessive–compulsive disorder (OCD) is a frequently encountered neuropsychiatric disorder with prevalence rates among children and adolescents comparable to those of the adult population (1–3%) (1). It is characterized by obsessions, i.e., intrusive thoughts that are unpleasant and frightening, and which the affected individuals attempt to resist or reject and by compulsions, i.e., repetitive, often stereotypic movements or mental rituals, which are semi-voluntary and performed to reduce the fear/anxiety or bodily reaction. OCD is a heterogeneous disorder in regard to its clinical presentation and treatment response (2–4). Its complex nature has likely complicated efforts to identify causal mechanisms associated with this disorder.

Both genetic and non-shared environmental factors are important in the etiology of OCD. Based on separate twin modeling analyses, a recent population-based study suggested that the increased familial risk of OCD was attributable to additive genetic factors (47%). No significant effect could be shown for shared environmental factors, whereas non-shared environmental factors were estimated to be as important as the genetic factors (5).

According to several review articles, genes affecting different neurotransmitter systems, neurotrophic factors, and sex hormones have been implicated in OCD (6, 7). It has been suggested that these systems form a delicate network of interacting components (8). However, as described in the review articles, results have been divergent. In association studies, the most stable association has been related to *SLC1A1*, the neuronal glutamate transporter gene, which encodes the glutamate transporter EACC1 (9) (Table 1). Other components of the glutamatergic system have also been associated with OCD. These components include genes encoding for the GABA B receptor (*GABBR1*) (10), glutamate decarboxylase 1 (*GAD 1*) (11), and Disks large-associated protein 1 (DAP-1) (*DLGAP1*) (12) (Table 1). Both the GABA B receptor and Gad67 are important in regulating the activity of glutamate, and DAP-1 is a member of the neuronal postsynaptic density complex known to be important in relation to the glutamatergic system. Furthermore, myelin oligodendrocyte glycoprotein (MOG) has been associated with OCD and with the severity of OCD. Moreover, the MOG G511C (Val142Leu) Val/Val genotype has been associated with morphological changes in the total white matter volume in patients with OCD (13). Zai and coauthors (14) showed an ­association of genetic variants and haplotypes within the *MOG* gene with a further association to the severity of OCD (Table 1). The serotonin transporter is a main target for the selective serotonin reuptake inhibitors (SSRIs), and several studies have investigated the association between variants within the gene encoding the serotonin transporter (*SLC6A4*) and OCD. Positive associations with especially early onset OCD have been identified (6, 15) (Table 1). Also, the brain-specific tryptophan hydroxylase-2 (TPH-2) enzyme involved in the synthesis of serotonin has been associated with OCD (16). On the contrary, studies of variations in the *COMT* gene have shown conflicting results (6). However, a recent study by Alonso et al. showed an association between dysfunctional beliefs assessed by the Obsessive Beliefs Questionnaire (OBQ-44) and an interaction between the Val158Met variant in the *COMT* gene and the Val66Met variant in the *BDNF* gene rather than an effect of either variant (17). Other studies have also suggested an association between neurotrophic factors and OCD. Hall et al. showed a strong association between *BDNF* gene variants and OCD (18) pointing towards possible risk alleles (including the *Val66Met BDNF* polymorphism). These findings have later been replicated by Hemmings et al. (19) and Katerberg et al. (20). Furthermore, Márquez et al. have shown a significant association between the occurrence of OCD and genetic variation at certain *BDNF* loci suggesting a role for BDNF in the development of OCD (21). Furthermore, the *BDNF* Val66Met polymorphism has been implicated in the increased risk of OCD resulting from trauma experienced during childhood (22). Additionally, it has been suggested that sex hormones may be implicated in the development or the worsening of OCD. Forray et al. (23) showed a relation between pregnancy and the onset of OCD. Furthermore, compared to other women, women with onset or worsening of OCD perinatally experienced a worsening of their OCD in relation to their menstrual period. Alonso et al. (24) investigated the association between variants in the estrogen receptor genes (*ESR1* and *ESR2*) and the occurrence of OCD. Although no direct association was observed, they found that certain OCD subphenotypes (contamination obsessions and cleaning compulsions) were associated with both a SNP in the *ESR1* gene (rs34535804) and a five-marker haplotype in the *ESR1* (5′ end of intron 1). These findings suggest that the *ESR1* gene could contribute to the genetic vulnerability associated with OCD manifestations. Furthermore, a study examining the severity of Post Traumatic Stress Disorder (PTSD) (often including anxiety and OCD) showed that a low level of estradiol could promote stronger PTSD symptoms and could be associated with poorer fear extinction (25) (Table 1). Apart from the association to Disks large-associated protein 1 (DAP-1) (*DLGAP1*), two recent genome-wide association studies (GWAS) were not able to replicate the findings from the candidate gene studies described above (12, 26). Since several of the associations were described either as a combined effect or as being related to epidemiological factors, a possible explanation is that the systems exert their effect through a delicate network of mutual influences that also involve environmental factors influencing the gene transcription and expression. Epigenetics is regarded an important interface between environmental signals and activation or repression of genomic responses. The mechanisms are dynamic and interchangeable over time. Through different mechanisms including modifications of DNA molecules, the epigenetic mechanisms regulate the accessibility for transcription factors and influence gene expression (27). The role of epigenetics in anxiety disorders has been underlined in studies examining the mechanisms of fear-related learning and memory involving histone acetylation [reviewed in Ref. (28), where different anxiety mechanisms and stress are discussed]. Furthermore, anxiety also involves DNA methylation mechanisms where DNA methyltransferases are activated by fear learning and associated with methylation within the promoter region of the gene encoding the protein phosphatase (28). In a publication from 2013, Domschke et al. showed a significantly lower *GAD1* methylation in adults with panic disorder than in age- and sex-matched healthy controls, and the occurrence of negative life events correlated with the decreased average methylation in a sex-differentiated manner. The authors suggested that the hypomethylation could be a compensatory reaction (29). A recent study examined the methylation of *SLC6A4* in children with anxiety disorder (30). The children were treated with cognitive behavioral therapy (CBT). An evaluation of the total group showed no differences in the methylation profiles, but comparison of responders with non-responders showed an increased methylation in one CpG-site of the *SLC6A4* promotor region in the responders (29). Even though several studies have examined epigenetic mechanisms in psychiatric disorders including in anxiety disorders, which are closely related to OCD, no study has examined epigenetic mechanisms in relation to OCD.

**Table 1:** **Previously published association studies in OCD**.

Systems	Gene	Association	Studies	Study type
Glutamatergic system	*SLC1A1*	Positive	Dickel et al. (9)	Family based
			Arnold et al. (42)	Family based
			Stewart et al. (43)	Family based
	*GABBR1*	A tendency for overtransmission	Zai et al. (10)	Family based
		A tendency for association to YBOCS		
	*DLGAP1*	Positive	Stewart et al. (12)	GWAS
	*MOG*	Positive	Atmaca et al. (13)	Case-control
			Zai et al. (14)	Family-based
Neurotrophic factors	*OLIG2*	Positive	Stewart et al. (43)	Family-based
	*NTRK3*	Positive	Alonso et al. (44)	Case-control
	*NTRK2*			
	*BDNF*	Positive	Hall et al. (18)	Family based
		No association	Mossner et al. (45)	Family based
Sex hormone	Estrogen receptor alpha 1 (*ESR1*)	Positive	Alonso et al. (24)	
Serotonergic system	*SLC6A4, 5HTTLPR*, and *VNTRSTin2*	A tendency for an association Positive	Walitza et al. (15)	Family based
	Serotonin transporter		Bloch et al. (46)	Meta-analysis
		Positive	Walitza et al. (47)	Meta-analysis
	*TPH2*	Overtransmission	Mössner et al. (16)	Family based
Dopaminergic system	Catecholamine-*O*-methyltransferase (*COMT*) (*COMT*val158met)	No association	Walitza et al. (48)	Family based

In the present study, we wanted to examine the methylation profile in selected genomic regions in children and adolescents diagnosed with OCD compared with age- and sex-matched controls. Furthermore, we wanted to compare the methylation profiles at birth and after OCD onset. Finally, we investigated the association of the methylation profiles with the severity of OCD and with treatment effect. To the best of our knowledge, this is the first study to examine the implication of epigenetics mechanisms in OCD in a population of children and adolescents.

## Materials and Methods

Permission to perform the study was granted by the Scientific Ethics Committee (1-10-72-149-12).

Children and adolescents were recruited through referrals from general practitioners, child mental health specialists, or pediatricians. All the children and adolescents were examined and diagnosed in the specialized OCD clinic in Aarhus, Denmark, by a team consisting of experienced and skilled psychologists and a child and adolescent psychiatrist. For diagnostic assessment, we used questions concerning obsessive–compulsive symptoms and comorbidity from The Schedule for Affective Disorders and Schizophrenia for School-Age Children Present and Lifetime Version (K-SADS-PL) (31). The Children’s Yale–Brown Obsessive–Compulsive Scale (CY-BOCS) (32) was used for assessment of severity scores at baseline and after treatment with CBT. The CY-BOCS is a clinician-administered instrument that evaluates obsessions and compulsions separately on time consumed, distress, interference, resistance, and control. It yields separate severity scores for obsessions and compulsions (0–20) and a composite severity score (0–40). The CY-BOCS scores were obtained at the time of diagnosis (defined as pre-treatment CY-BOCS scores) and at 13 weeks or at dismissal (defined as post-treatment CY-BOCS scores).

Age- and sex-matched controls were recruited through advertising at schools and a subsequent contact to parents through email or telephone. If the child or adolescent wanted to participate, a short interview was conducted that included questions on anxiety and OCD (ever experienced symptoms). Only information on females is presented, since the total number of males was very low.

The neonatal blood spot samples from Guthrie cards (referred to as “OLD samples” in the following sections) were obtained from the Danish Neonatal Screening Biobank (DNSB) located in the Statens Serum Institut (SSI). New blood spots (referred to as “NEW samples” in the following sections) were collected from a fingertip on S&S903 filter paper (33) (GA Whatman) from the same individuals at the time of diagnosis. As such, OLD samples (neonatal blood samples) and NEW samples were from the same individuals, but the samples were taken at different time points. Before collecting the blood from the fingertips, the finger was cleaned with a wipe with isopropyl alcohol. Next, the area was air-dried, and the insert was carried out with a lancette (Microlet, Bayer, Germany). The first drop of blood was dried up. A large drop of blood was allowed to form on the finger, and then the center of the pre-printed circles on filter paper was gently placed on the droplet so that it was absorbed. Blood spots were dried at room temperature for about 3 h and then kept frozen (−20°C) until analysis.

DNA was extracted from the filter paper as previously described (34, 35). The epigenetic analysis was conducted using the Illumina Infinium HumanMethylation450 Beadchip (Illumina San Diego, CA, USA). The degree of methylation was examined by beta-values. This method describes the percentage methylation of a CpG probe [beta = methylated/(methylated + unmethylated)].

We investigated genes in the glutamatergic (*SLC1A1, SLC25A12, GABBR1, GAD1, DLGAP1, MOG*), the serotonergic (*SLC6A4, TPH2*), and the dopaminergic systems (*COMT*), as well as neurotrophic genes (*OLIG2, NTRK3, NTRK2, BDNF*), and the sex hormone receptor (*ESR1*) (Table 2). The genes and their CpGs tags (all CpGs 100,000 bp up and downstream from the open reading frame – 1,486 CpG probes) were selected based on hypotheses about gene location, gene function, and the results from association studies in relation to OCD (Table 1). The selection of CpG sites was performed due to the small sample size.

**Table 2:** Overview of the genes tested for a hypothesized association with OCD and the number of probes included 100,000 bp up and down stream of the genetic region – *413 probes were overlapping between *GABBR1* and *MOG* (*GABBR1/MOG*).

Systems	Gene	Probes (count)	Methylated variable positions (limma) Case vs. ctrl	
			OLD (case: *N* = 21, control: *N* = 11)	NEW (case: *N* = 21, control: *N* = 12)
Glutamatergic system	*SLC1A1*	28	NS	NS

	*SLC25A12*	47	NS	NS

	*GABBR1*	519*	NS	NS
			^cg10234998* (*GABBR1*, TSS1500), *p*-val: 0.00023/FDR *p* 0.18, Fold-change (case/ctrl): 1.14	
			^cg17099072* (*GABBR1*, TSS1500) *p*-val: 0.00026/FDR *p* 0.18 Fold-change (case/ctrl): 1.12	

	*GAD1*	87	NS	NS

	*DLGAP1*	103	NS	NS

	*MOG*	571*	NS	NS
			^cg10234998* (*GABBR1*, TSS1500), *p*-val: 0.00023/FDR *p* 0.18, Fold-change (case/ctrl): 1.14	**^cg16650906** (*LOC285830*, body) *p*-val: 0.00017/FDR *p* 0.12, Fold-change (case/ctrl): 0.976
			^cg17099072* (*GABBR1*, TSS1500) *p*-val: 0.00026/FDR *p* 0.18
			Fold-change (case/ctrl): 1.12

Neurotrophic factors	*BDNF*	86	NS	NS
				**^cg14080521** (*BDNF-AS*, IGR) *p*-val: 0.00031/FDR *p* 0.14, Fold-change (case/ctrl): 0.98

	*OLIG2*	82	NS	NS

	*NTRK3*	51	NS	NS

	*NTRK2*	19	NS	NS

Sex hormone	*ESR1*	73	NS	NS
				**^cg10939667** (*ESR1*, Body) *p*-val: 0.00016/FDR *p* 0.12, Fold-change (case/ctrl): 0.98

Serotonergic system	*SLC6A4* (*SERT*)	63	NS	NS

	*TPH2*	42	NS	NS

Dopaminergic system	*COMT*	128	NS	NS

*NS, not-significant describes that a FDR corrected *p*-value of <0.05 was not achieved for any genetic region. However, we have mentioned (bold writing) the top three probes that had *p*-values <0.2 for each methylation variable position analysis (OLD and NEW) (see the full list of *p*-values as in Supplementary Material). ^The top three probes with *p*-values <0.2 are written in bold writing, and location abbreviation of the probe position relative to the gene are: TSS1500, 1500 bp upstream from transcription start site; body, in gene body; IGR, intergenic region*.

### DNA Methylation Variable Positions

The methylation profiles were examined by comparing methylation beta-values of OCD patients from blood obtained at the time of diagnosis (NEW case) with healthy controls (NEW control) of comparable age distribution and birth year. Likewise, methylation profiles were compared in neonatal blood from the same patients (OLD case) and controls (OLD control) obtained from the DNSB. For statistical methylation variable positions (MVP) analysis, we used the ChAMP package in *R* (36). Raw data was filtered to remove probes with detection *p*-value >0.01 and bead count <3. Likewise, probes aligning to multiple locations and containing SNPs identified by Nordlund et al. (37) were also removed. No X and Y chromosome hybridizing probes were included in this study. This resulted in a total of 434,493 passed probes of the 485,512 probes in the Illumina Human Methylation 450k array. The data were normalized by the SWAN method and converted to beta values hereafter batch corrected for technical variation by the combat method build into the ChAMP package pipeline.

After filtering with the list of 1,486 hypothesized OCD probes (CpG probes located ± 100.000 bp from the genetic reading frame of the selected genes, *SLC1A1, GABBR1, GAD1, DLGAP1, MOG, TPH2, OLIG2, NTRK3, NTRK2, ESR1, SLC25A12, SLC6A4, COMT*, and *BDNF*), 1,368 probes were included for further analysis, and the remaining were not used further in this study.

Beta values were chosen due to the small sample size. The probes were filtered for Det *P* < 0.01, and a sample Call Rate >99% was used (all samples met these criteria). MVPs between the case and control group for either the NEW or OLD samples were investigated using a linear model across all selected probes, and correction for multiple testing was applied by the false discovery rate method (FDR).

Top ranking genes from the MVP association were associated with the severity scores (CY-BOCS). Probes ± 100,000 bp up/downstream of *ESR1, MOG*, and *BDNF* were analyzed for the NEW cases only; and probes ± 100,000 bp up/downstream of *GABBR1* and *MOG* were analyzed for the OLD cases only.

The association between the methylation status and the severity of OCD was examined by correlating the methylation status and the baseline CY-BOCS score at the time of diagnosis and the CY-BOCS differences (post-treatment CY-BOCS scores subtracted from baseline CY-BOCS scores). The analysis was performed in *R* by applying the Pearson correlation test between scores and probe methylation status across all evaluated probes. We corrected for multiple testing using the FDR method.

Treatment response was assigned to cases having a CY-BOCS <16 at the end of treatment (week 13). Responder methylation status was compared with non-responder methylation status by a double-sided *t*-test across all probes. We corrected for multiple testing using the FDR method.

### Differential Analysis

The difference between methylation status at birth and at the time of diagnosis between cases and controls was assessed by differential methylation analysis on delta-beta-values [beta-values (OLD) minus beta-values (NEW)] with a linear model analysis in the ChAMP pipeline between cases and controls. We corrected for multiple testing using the FDR method.

## Results

### Demographic Description of the Cohorts

The clinical population consisted of 21 female children and adolescents (13.29 ± 2.63) with verified OCD according to the ICD-10. The clinical cohort was age- and sex-matched with controls; it included 12 females (13.17 ± 2.48) in the NEW cohort and 11 females (13.18 ± 2.60) in the OLD cohort. The severity of the OCD in cases at baseline/pre-treatment was 25.86 ± 5.00 and at post-treatment 14.71 ± 5.66 (Table 3). The occurrence of comorbid psychiatric disorders is shown in Table 4. Out of the 21 patients, 8 patients had one comorbid disorder, and 5 patients had more than one comorbid disorder (2 or 3 comorbid disorders). None of the matched controls had ever experienced OCD or anxiety symptoms.

**Table 3:** **Sample statistics of age and OC symptom severity**.

Sample statistics	Case female *N* = 21 (NEW and OLD)	Control female	
		*N* = 12 (NEW)	*N* = 11 (OLD)
Age	13.29 ± 2.63	13.17 ± 2.48	13.18 ± 2.60
Birth year	1999 ± 2.5	2000 ± 2.6	2000 ± 2.7
CY-BOCS baseline	25.86 ± 5.00	NA	
CY-BOCS at 13 weeks/dismissal	14.71 ± 5.66	NA	
Comorbidity	13/21 (61.9%)	NA	

*In the case group, 21 females with clinically diagnosed OCD have been analyzed for their methylation profile at birth (OLD) and at the clinical time of diagnosis (NEW). The 12 females of comparable age distribution were included as NEW controls, and 11 of these were included as neonatal controls (OLD) – of comparable birth year distribution. Age, year/months, and OC symptom severity (±SD)*.

**Table 4:** **The occurrence of comorbid disorders in the case group (*N* = 21) described in accordance with the ICD 10 diagnostic sub-classification**.

Comorbidity	Case female
	*N* = 21 (NEW and OLD)
Mood disorders	2
Anxiety disorders	4
Trichotillomania	1
Developmental disorders	3
Hyperkinetic disorder	1
Conduct disorder	1
Emotional disorders	6
Tic disorders	4

### MVP Analysis

The microarray analyses included 21 OLD and NEW case pairs, 11 OLD controls, and 12 NEW controls.

Comparison of the methylation profiles of female OCD subjects (NEW) with the methylation profiles of control subjects (NEW) revealed no significant differences in the methylation of CpG sites associated with the selected genes. The top-three most altered methylation sites in terms of *p*-value were seen in the body of the *LOC285830* gene within the chosen range (100,000 bp) of the *MOG* gene on chromosome 6 (probe cg16650906), in the vicinity of the *BDNF* gene on chromosome 11 (probe cg14080521), and in the gene body of the *ESR1* gene on chromosome 6 (probe cg10939667). For all sites, the fold-change (case/control) was 0.98 showing a 2% difference between the cases and the controls. In blood samples at birth (OLD), the most altered methylation sites were in the *GABBR1* gene overlapping with the *MOG* gene selected probes on chromosome 6 (probes cg10234998 and cg17099072). The two probes were located close to each other (only 2 bp apart) near the *GABBR1* transcription start site (TSS1500 shore – within 1500 bp of the transcription start site) (Table 2). The fold-change (case/control) was 1.14 and 1.12, respectively.

No significant MVPs were found in the longitudinal analysis of the delta-beta values (OLD-NEW). Still, like in the OLD sample MVP analysis, the two same *GABBR1/MOG* probes (cg17099072 and cg10234998), as found in the OLD sample MVP analysis, showed a differential methylation (FDR *p*-value 0.135 for both) with a fold change (case/control) in delta-beta values of 1.88 and 1.45, respectively. This effect appears to be inherent from the MVP seen in the OLD cohort MVP analysis, where the more methylated OLD cases no longer show support for a differentially methylation in the NEW cases compared with their respective controls (FDR *p*-values 0.664 and 0.956, respectively).

### Severity Score (CY-BOCS) and Treatment Responder Status Associated With Methylation Status in Selected Probes

In relation to the severity scores (CY-BOCS), we analyzed the probes associated with the MVP top hits both for NEW cases: *ESR1*, *MOG* (inherently some *GABBR1/MOG* overlapping probes) and *BDNF*, and for OLD cases: *GABBR1* and *MOG*. Neither significant association was found between the methylation profiles in the OLD cases and baseline CY-BOCS nor were any differences found between baseline and differences in CY-BOCS scores (post-treatment compared with baseline CY-BOCS scores). However, a *MOG*-associated probe in a CpG island near the gene *IFITM4P* (cg15395148, *r* = −0.7) had a lower *p*-value than the remaining samples (association to baseline CY-BOCS score), and a probe in a CpG island at the 5′ UTR end of the *GABBR1* gene showed the same tendency (cg19456996, *r* = −0.7) (association to the differences in CY-BOCS scores).

We investigated the correlation of the baseline CY-BOCS and CY-BOCS difference in the NEW cases with the methylation status of the selected NEW probes and found none of the probes to be significantly associated with the scores. However, one *ESR1* probe in the 5′ UTR end of the gene (cg25565730, *r* = −0.68) and two probes selected for *MOG*, but located within the *GABBR1* gene (cg08967211 and cg17071948, *r* = −0.64 and −0.65), showed a lower *p*-value than the remaining probes.

Finally, in NEW samples, there was a preliminary support for a positive association between the responders and the methylation state of probes near the *MOG* gene, where the top scoring probe was located in the *GABBR1* gene body [cg16922688, fold change (responder/non-responder) = 1.02], while two probes in the TSS1500 of the *LOC285830* gene, only 9 bp apart (cg09567915 and cg11497864) exhibited the same tendency of association with comparable fold changes of 1.26 and 1.27, respectively (Table 5).

**Table 5:** **Top hit genes from MVP analysis of NEW (*N* = 21) (*ESR1, MOG*, and *BDNF*) and OLD (*MOG* and *GABBR1*) association with CY-BOCS baseline (A), CY-BOCS difference from admission until the end of treatment (B) and patient responsiveness to treatment (C) evaluated as a CY-BOCS <16 at the end of treatment**.

(A) CY-BOCS BASELINE	CpG	Gene	RefGENE/feature	Corr *r*-value	*p*-value	FDR *p*-value
NEW case (*N* = 21)	cg25565730	*ESR1*	*ESR1*, 5′ UTR	−0.68	0.0007	0.39
	cg08967211	*GABBR1*/*MOG*	*GABBR1*, body	−0.64	0.0016	0.39
	cg17071948	*GABBR1*/*MOG*	*GABBR1*, body	−0.64	0.0018	0.39
OLD case (*N* = 21)	cg15395148	*MOG*	*IFITM4P*, IGR	−0.70	0.00043	0.26
	cg06613392	*GABBR1*	*UBD*, IGR	−0.65	0.00157	0.48
	cg19348622	*GABBR1*/*MOG*	*HLA-F*, body	−0.60	0.00438	0.89

**(B) CY-BOCS DIFFERENCE**	**CpG**	**Gene**	**RefGENE/feature**	**Corr *r*-value**	***p*-value**	**FDR *p*-value**

NEW case (*N* = 21)	cg06380702	*MOG*	*IFITM4P*, IGR	−0.64	0.0019	0.68
	cg24764793	*ESR1*	*ESR1*, 5′ UTR	−0.59	0.0050	0.68
	cg10601943	*GABBR1*/*MOG*	*HLA-F*, body	−0.59	0.0051	0.68
OLD case (*N* = 21)	cg1945699	*GABBR1*/*MOG*	*GABBR1*, 5′ UTR	−0.70	0.00039	0.24
	cg01306985	*MOG*	*IFITM4P*, TSS1500	−0.62	0.0027	0.52
	cg12296326	*MOG*	*LOC285830*, TSS1500	−0.60	0.0037	0.52

**(C) TREATMENT RESPONDER**	**CpG**	**Gene**	**RefGENE/feature**	**Fold-change**	***p*-value**	**FDR *p*-value**

NEW case (*N* = 21)	cg16922688	*GABBR1*/*MOG*	*GABBR1*, body	1.02	0.00042	0.15
	cg09567915	*MOG*	*LOC285830*, TSS1500	1.26	0.00048	0.15
	cg11497864	*MOG*	*LOC285830*, TSS1500	1.27	0.00068	0.15
OLD case (*N* = 21)	cg08541345	*MOG*	*LOC285830*, body	1.30	0.0014	0,52
	cg16118803	*GABBR1*/*MOG*	*MOG*, body	1.17	0.0019	0.52
	cg12463578	*GABBR1*/*MOG*	*ZFP57*, first exon	0.84	0.0028	0.52

*A and B reflect the correlation between severity scores and beta-values, while C reflects the most different hits between responders and non-responders tested by a parametric *t*-test, and fold change describing the responder/non-responder ratio in the mean beta-value. All *p*-values are corrected for multiple testing by the FDR method. RefGENE/Chr denotes the exact genetic location of the three top-hit probes in each category. 5′ UTR, 5′ untranslated region; body, gene body; IGR, intergenic region; TSS1500, within 1500 bp of transcription start site; first exon, in the first exon. Gene investigated by probes in its genetic region and 100,000 bp up/downstream. Exact RefGene and feature of the probe is annotated for all top-hit genes in this table*.

## Discussion

The present study examines the methylation profiles of genes hypothesized to be important in OCD in children and adolescents. To the best of our knowledge, no other study has investigated the methylation status in OCD in children and adolescents or examined the difference between methylation status at birth and at the time of the OCD diagnosis.

In the present study, differential methylation analysis between case and control was performed based on a hypothesis-driven selection of genes. We found no MVPs. However, CpG probes in *GABBR1* and *MOG* (OLD samples) and probes in *MOG, BDNF* and *ESR1* (NEW samples) had the lowest *p*-values. In OLD samples, the study supported an increased methylation of probes associated with *GABBR1*/*MOG*, whereas in NEW samples, cases showed a slightly lower degree of methylation of a CpG in *ESR1*. The extent of modification required in order to achieve functional significance is largely unknown. However, methylation changes in blood are influenced by the heterogeneous nature of the tissue, and markers associated with diseases in the brain are known to be of a smaller effect size when analyzed in blood (38).

Previous studies have shown a reduced expression of *BDNF* in relation to anxiety disorders and OCD (39), which could be caused by a possible differential methylation in the *BDNF* gene. *BDNF* has been implicated in fear extinction where it has been suggested that the serotonergic and glutamatergic systems may mediate their effects on fear through the *BDNF* gene expression (40, 41). Thus, an altered methylation of *BNDF* probes could reflect an altered risk of expressing OCD.

To our knowledge, no previous study has examined the methylation status of *ESR1* in relation to OCD or anxiety. It has been suggested that estrogen receptor genes play a role in relation to OCD (24). Even though Alonso et al. could not show any difference between controls and subjects with the categorical OCD phenotype, their study identified genetic variants that were associated with the presence of certain OCD symptoms. This finding suggests that the *ESR1* gene could contribute to the genetic vulnerability to OCD manifestations. In the present study, we observed a tendency towards a reduced methylation in NEW female cases compared with NEW female controls and tendency towards an inverse correlation between the methylation of *ESR1* and the pre-treatment severity of OCD (as evaluated by the CY-BOCS score). Interestingly, that the altered methylation of the *ESR1* gene was observed only in NEW samples from female cases; that is in females in their teens where estrogens become increasingly important and where the sex ratio observed in relation to OCD is altered. We only conducted the analyses for females, but other studies have shown a sex difference in relation to the expression of ESR. For example, Delgrange et al. (49) showed that the expression of ESR1 was inversely correlated with the size and proliferative activity of a prolactin pituitary tumor. This finding could suggest a sex-specific receptor expression. The associated probes are located in the gene body and in the 5′ end of the gene on chromosome 6, respectively. These locations do not rule out a regulatory role, and the results could suggest that the percentage methylation of the CpG probe plays an important role for OCD in females, and for the severity of the disorder. This finding would be in accordance with the association between the expression of *ESR1* and the prognosis of pituitary tumor (47) and with the proposed exacerbating role of a low level of estradiol on PTSD and the described poorer fear extinction (25). To explore the importance of the expression of *ESR1* in relation to OCD further, future studies could evaluate the degree of methylation correlated with the pubertal development in females.

The *MOG* gene encodes the MOG which is thought to be important in the myelination of nerves potentially providing the structure of myelin sheath and also in relation to the immune system (13, 14). The *GABBR1* gene encodes the gamma-­aminobutyric acid (GABA) B receptor 1 (GABAB1) (10). Through the GABAB1 receptor, GABA is thought to fine-tune the inhibitory synaptic transmission including the effect of glutamate, which has been associated with the occurrence of OCD. In the present study, probes for the two genes were overlapping showing a potential regulatory role for one or both genes. Both *GABBR1* and *MOG* showed altered methylation in the blood samples at birth (OLD). This may suggest that they are important to the developing brain where we may hypothesize that early alterations alter the risk of later developing OCD. Both GABA receptor signaling and glutamate have been implicated in proliferation, migration, differentiation, and survival processes with a differential expression over lifetime (50). The altered methylation of *GABBR1/MOC* observed in the newborn children, however, seemed to stabilize to control levels from OLD to NEW cases, as shown by the differential longitudinal analysis of the most significant changes from OLD to NEW. *MOG* (*LOC285830*) showed an altered methylation in the NEW samples. The *MOG* gene has been associated with the presence of OCD, and it has also been related to the severity of the disorder. Furthermore, it has been related to changes in white matter and to inflammatory processes (13, 14). It is not known whether the observed changes arise before the OCD onset or if they could be a consequence of having OCD. However, the observed tendency towards altered methylation in cases compared with controls and in relation to the severity of the disorder and the responder status could suggest a regulatory role.

Since methylation levels are not confined to single probes, but are more likely to affect entire regions causing differentially methylated regions, most of these tendencies may be seen as possible candidates for future investigation. However, the closely related LOC285830 transcription start site probes placed in the vicinity of the *MOG* gene appeared to exhibit uniformly higher methylation level in treatment responders compared with non-responder in the adolescent OCD patients. Furthermore, the consistent tendency of the *GABBR1* transcription start site probes to have at higher methylation level in newborn cases compared with controls reveals that epigenetic regional methylation levels might indeed be involved in the pathogenesis of OCD, even if no overall differentially methylated regions were identified in this study.

## Limitations

The present study includes only very few patients and controls. Nevertheless, it reached nominally significant results and gave preliminary support for an association between the methylation of *ESR1, GABBR1*, and *MOG* and the severity of the OCD and treatment outcome. However, due to the limited number of patients and controls, the study did not account for potentially confounding factors, including parental smoking or illnesses in cases/controls at birth. Furthermore, the investigated genes were not selected based on GWAS results, which have shown different results than candidate gene studies. However, in view of the limited number of patients and controls, we chose to focus on hypothesis-driven selected genes in a cohort of females. We are collecting additional blood specimen from children and adolescents with OCD as to perform a future epigenome-wide association study. Specimen from the central nervous system would have been optimal for examining the methylation patterns. However, by necessity, the DNA was extracted from peripheral blood rather than from central nervous tissue. It has been discussed in previous articles that even though the epigenome does show tissue-specific patterns, epigenetic modifications may be detectable across tissues (51, 52). The specific positions of the probes we have examined in the present study and the indicated methylation differences are not assessed for their gene regulatory functions. However, a larger study providing significant MVPs and especially differentially methylated regions would be able to link the methylation changes to the annotated genetic regions and to speculate on the biological significance.

The findings do not explain the implicated mechanisms, i.e., the nominal differences in methylation may be either the cause or the result of OCD. Nevertheless, the study supports the hypothesis that epigenetic factors are of importance in mental disorders.

## Ethical Considerations

The study was approved by the ethics committee and was conducted in accordance with the latest version of the ethical standards as laid down in the 1964 Declaration of Helsinki and its later amendments. Informed consent was obtained from the participants after the nature of the procedures had been fully explained.

## Author Contributions

All authors listed, have made substantial, direct, and intellectual contribution to the work, and approved it for publication. However, MH only approved the first edition of the manuscript, since he passed away before the final edition was ready.

## Conflict of Interest Statement

The authors declare that the research was conducted in the absence of any commercial or financial relationships that could be construed as a potential conflict of interest.
