# Supplementary material for: DNA Methylation at the Neonatal State and at the Time of Diagnosis: Preliminary Support for an Association with the Estrogen Receptor 1, Gamma-Aminobutyric Acid B Receptor 1, and Myelin Oligodendrocyte Glycoprotein in Female Adolescent Patients with OCD
Source: Front Psychiatry. 2016 Mar 18;7:35. doi: 10.3389/fpsyt.2016.00035 (PMC4796012; doi:10.3389/fpsyt.2016.00035)
Supplement: Supplementary file 1 [file table_1.PDF]

logFC	AveExpr	t	P.Value	adj.P.Val	B	probeID	CHR	MAPINFO	arm	OCD_GENE(+/-100.000bp)	gene	distancetoGene	feature	cgi	feat.cgi	conserved_tfbs	Case_OLD-NEW_AVG	Ctrl_OLD-NEW_AVG	deltaBeta	Foldchange (Case/ctrl)
-0.070938959	0.127299411	-4.345017602	0.000130835	0.135117649	0.203291086	cg17099072	6	29601489	p	GABBR1/MOG	GABBR1	NA	TSS1500	shore	TSS1500 - shore	NA	0.151684678	0.080745719	-0.070938959	1.87854761
-0.045030254	0.129599368	-4.200641977	0.00019754	0.135117649	-0.200023874	cg10234998	6	29601491	p	GABBR1/MOG	GABBR1	NA	TSS1500	shore	TSS1500 - shore	NA	0.145078518	0.100048264	-0.045030254	1.450085306
-0.032323964	-0.011721209	-3.723171904	0.000753994	0.264366237	-1.502562347	cg06684850	11	27742369	p	BDNF	BDNF	NA	Body	shore	Body - shore	NA	-0.000609846	-0.032933811	-0.032323964	0.018517333
-0.031641855	0.001078856	-3.714149031	0.000773001	0.264366237	-1.526625997	cg16650906	6	29697700	p	MOG	LOC285830	NA	Body	open sea	Body - open sea	NA	0.011955744	-0.019686112	-0.031641855	-0.607318708
-0.036001224	0.013908672	-3.43484061	0.001654917	0.438609573	-2.259306068	cg03739409	12	72335078	q	TPH2	TPH2	NA	Body	open sea	Body - open sea	NA	0.026284093	-0.009717131	-0.036001224	-2.704923037
-0.033339096	-0.00443814	-3.348036262	0.002088078	0.438609573	-2.481749833	cg21026120	6	29581121	p	GABBR1/MOG	GABBR1	NA	Body	open sea	Body - open sea	V$PAX5_01	0.007022175	-0.026316922	-0.033339096	-0.266831155
-0.075503053	-0.028182376	-3.320928159	0.002244347	0.438609573	-2.550659836	cg08725892	6	29629986	p	GABBR1/MOG	MOG	NA	Body	open sea	Body - open sea	NA	-0.002228201	-0.077731255	-0.075503053	0.028665448
-0.011717364	0.004304954	-3.193349428	0.003142879	0.507775588	-2.871226192	cg03851143	6	29691250	p	GABBR1/MOG	HLA-F	NA	1stExon	island	1stExon - island	NA	0.008332798	-0.003384566	-0.011717364	-2.461999075
-0.054906276	0.013692599	-3.147812159	0.003539925	0.507775588	-2.98408923	cg08289189	22	19937277	q	COMT	COMT	NA	5'UTR	open sea	5'UTR - open sea	NA	0.032566632	-0.022339644	-0.054906276	-1.45779546
-0.024553537	-0.008880836	-3.041187052	0.004664579	0.507775588	-3.244967547	cg17242574	6	29625991	p	GABBR1/MOG	MOG	NA	Body	open sea	Body - open sea	NA	-0.000440557	-0.024994095	-0.024553537	0.017626463
-0.023834544	-0.005100136	-2.988166436	0.005342745	0.507775588	-3.372853033	cg17532626	6													

29589036 p GABBR1/MOG GABBR1 NA Body open
sea Body - open sea NA 0.003092989 -0.020741555
-0.023834544 -0.149120378
-0.030988702 0.032313827 -2.974306156 0.005534826
0.507775588 -3.406075877 cg14291693 11
27683959 p BDNF BDNF NA Body open
sea Body - open sea NA 0.042966193 0.011977491
-0.030988702 3.587244738
-0.036901777 0.003153835 -2.966911786 0.00563995
0.507775588 -3.423764284 cg27521571 22
19938424 q COMT COMT NA 5'UTR open
sea 5'UTR - open sea NA 0.015838821 -0.021062956
-0.036901777 -0.751975205
-0.013106309 -0.004759313 -2.888601551 0.006875121
0.507775588 -3.609538355 cg02117021 6
29425960 p GABBR1 OR2H1 NA TSS1500 open
sea TSS1500 - open sea NA -0.00025402 -0.013360328
-0.013106309 0.019012974
-0.053053667 0.011030207 -2.875564578 0.007103985
0.507775588 -3.640184811 cg23627083 2
171787727 q GAD1 GORASP2 NA Body shore
Body - shore NA 0.029267405 -0.023786262
-0.053053667 -1.230433121
-0.01464871 0.00535812 -2.840217761 0.007761029
0.507775588 -3.722863154 cg10939667 6
152201611 q ESR1 ESR1 NA Body open
sea Body - open sea NA 0.010393614 -0.004255096
-0.01464871 -2.442627007
-0.017397019 0.00763263 -2.830111283 0.007959125
0.507775588 -3.74639084 cg18676033 6
29691815 p GABBR1/MOG HLA-F NA Body island
Body - island NA 0.013612856 -0.003784164
-0.017397019 -3.59732226
-0.017962036 -0.004061082 -2.802303383 0.008528914
0.507775588 -3.810866806 cg16324072 22
19978018 q COMT ARVCF NA Body shelf
Body - shelf NA 0.002113368 -0.015848668
-0.017962036 -0.133346747
-0.027723092 0.007578543 -2.773485292 0.009159529
0.507775588 -3.877277476 cg26718763 6
29573014 p GABBR1/MOG GABBR1 NA Body open
sea Body - open sea NA 0.017108356 -0.010614736
-0.027723092 -1.611755281
-0.02182192 -0.024106675 -2.75533555 0.009578777
0.507775588 -3.918887536 cg21229268 21
34442350 q OLIG2 OLIG1 NA TSS200 island
TSS200 - island NA -0.01660539 -0.03842731
-0.02182192 0.432124715
-0.081450934 -0.014651163 -2.755179198 0.009582465
0.507775588 -3.919245258 cg03198009 6
29648604 p GABBR1/MOG ZFP57 8435 IGR open
sea IGR - open sea NA 0.013347596 -0.068103338
-0.081450934 -0.195990332
0.045201574 -0.130458932 2.724510593 0.010332092
0.507775588 -3.989169842 cg02475474 6
29635158 p GABBR1/MOG MOG NA 3'UTR open

sea 3'UTR - open sea NA -0.145996973 -0.100795399
0.045201574 1.448448787
-0.019860252 0.00802097 -2.707164606 0.010779914
0.507775588 -4.028502928 cg13254553 6
29457282 p GABBR1 MAS1L 2739 IGR open
sea IGR - open sea NA 0.014847932 -0.00501232
-0.019860252 -2.962287135
-0.032545308 0.001919551 -2.702446136 0.010904819
0.507775588 -4.039175127 cg14787188 6
29636011 p GABBR1/MOG MOG NA 3'UTR open
sea 3'UTR - open sea NA 0.013107001 -0.019438307
-0.032545308 -0.674287148
-0.015826121 -0.000420998 -2.700258306 0.010963191
0.507775588 -4.044119578 cg24437600 12
72361228 q TPH2 TPH2 NA Body open
sea Body - open sea NA 0.005019231 -0.01080689
-0.015826121 -0.464447289
-0.031290699 0.051244516 -2.685302475 0.01137006
0.507775588 -4.077851898 cg16343924 6
29430158 p GABBR1 OR2H1 NA Body open
sea Body - open sea NA 0.062000694 0.030709995
-0.031290699 2.018909297
-0.014408435 0.02027714 -2.680826127 0.011494539
0.507775588 -4.087925136 cg01311802 6
29430435 p GABBR1 OR2H1 NA Body open
sea Body - open sea NA 0.02523004 0.010821605 -
0.014408435 2.331450837
0.009145914 -0.002348334 2.662018695 0.012031501
0.507775588 -4.130131462 cg00298481 11
27722063 p BDNF BDNF NA Body island
Body - island NA -0.005492242 0.003653673
0.009145914 -1.503211334
-0.032552778 -0.054482285 -2.655896265 0.012211261
0.507775588 -4.143830188 cg27491398 6
29708311 p MOG LOC285830 NA Body open
sea Body - open sea NA -0.043292268 -0.075845045
-0.032552778 0.57079889
-0.023831811 0.029554585 -2.641142293 0.012654735
0.507775588 -4.17675882 cg21468949 6
29430334 p GABBR1 OR2H1 NA Body open
sea Body - open sea NA 0.03774677 0.013914959 -
0.023831811 2.712675579
-0.017712312 0.002138448 -2.635463591 0.012829368
0.507775588 -4.189401469 cg14377523 6
29572373 p GABBR1/MOG GABBR1 NA Body open
sea Body - open sea NA 0.008227055 -0.009485256
-0.017712312 -0.867351941
-0.008833353 -0.001902527 -2.629155308 0.013025978
0.507775588 -4.20342525 cg18488157 6
29521598 p GABBR1 UBD -1791 IGR island
IGR - island NA 0.001133938 -0.007699415
-0.008833353 -0.147275817
-0.016606024 -0.005846171 -2.627757905 0.013069906
0.507775588 -4.206528852 cg00806253 6
29571432 p GABBR1/MOG GABBR1 NA Body open
sea Body - open sea V$TAXCREB_01 -0.00013785 -
0.016743874 -0.016606024 0.008232879

0.008201544 -0.002943919 2.627648459 0.013073352
0.507775588 -4.206771885 cg09547815 6
29691943 p GABBR1/MOG HLA-F NA Body island
Body - island NA -0.0057632 0.002438344
0.008201544 -2.363571144
-0.027630213 0.002240044 -2.623849367 0.013193503
0.507775588 -4.21520398 cg07238832 11
27681475 p BDNF BDNF NA Body open
sea Body - open sea NA 0.011737929 -0.015892283
-0.027630213 -0.738593006
-0.02629864 -0.000164994 -2.618559161 0.013362515
0.507775588 -4.226932484 cg20884110 6
152464870 q ESR1 SYNE1 NA Body open
sea Body - open sea NA 0.008875163 -0.017423476
-0.02629864 -0.5093796
-0.019153362 0.002635204 -2.586537697 0.014429022
0.533483822 -4.29759652 cg14847514 12
72340376 q TPH2 TPH2 NA Body open
sea Body - open sea NA 0.009219172 -0.00993419
-0.019153362 -0.928024581
0.009572777 -0.001889574 2.564661696 0.01520223
0.534632545 -4.345544853 cg10446968 2
172750661 q SLC25A12 SLC25A12 NA Body shore
Body - shore NA -0.005180216 0.004392561
0.009572777 -1.179315619
0.01403797 -0.005668293 2.543665204 0.015980102
0.534632545 -4.391312854 cg10523903 18 4455646
p DLGAP1 DLGAP1-AS5 191044 IGR island IGR -
island V$PAX5_01;V$AHRARNT_01 -0.010493845 0.003544125
0.01403797 -2.960913321
0.00665486 -0.001209809 2.539959542 0.016121134
0.534632545 -4.399364556 cg24738387 2
171627280 q GAD1 GAD1 -45920 IGR island
IGR - island NA -0.003497417 0.003157443
0.00665486 -1.107674111
-0.014652148 -0.008330269 -2.532511542 0.016408064
0.534632545 -4.41552405 cg11286436 15
88798582 q NTRK3 NTRK3 NA Body shore
Body - shore NA -0.003293593 -0.017945741
-0.014652148 0.183530613
-0.020757984 0.009369577 -2.532354684 0.016414157
0.534632545 -4.415864038 cg21797131 22
19970601 q COMT ARVCF NA Body shelf
Body - shelf NA 0.016505134 -0.00425285
-0.020757984 -3.880958516
-0.041826568 0.018100112 -2.47705482 0.018696573
0.560110385 -4.5348441 cg10665848 6 29631447 p
GABBR1/MOG MOG NA Body open sea Body - open sea
NA 0.032477994 -0.009348573 -0.041826568
-3.474112336
-0.021470466 -0.001078877 -2.475279348 0.018774454
0.560110385 -4.538634734 cg04324598 6
29602034 p GABBR1/MOG GABBR1 NA TSS1500 shore
TSS1500 - shore NA 0.006301596 -0.01516887
-0.021470466 -0.415429489
0.027542925 -0.00345161 2.475102848 0.018782213
0.560110385 -4.53901146 cg16403860 21

34394412 q OLIG2 OLIG2 -3804 IGR shore
IGR - shore NA -0.012919491 0.014623434
0.027542925 -0.883478565
-0.008064678 0.000809001 -2.473923671 0.018834121
0.560110385 -4.541527864 cg11539173 22
19843009 q COMT GNB1L NA TSS1500 island
TSS1500 - island NA 0.003581234 -0.004483444
-0.008064678 -0.798768672
-0.019239483 0.012963669 -2.442355589 0.02027353
0.58037298 -4.608591296 cg14809932 6 29525723 p
GABBR1/MOG UBD NA Body shelf Body - shelf
NA 0.019577241 0.000337758 -0.019239483
57.96226063
-0.02446893 -0.021137346 -2.402283217 0.02224524
0.58037298 -4.692868164 cg13369999 6 29711465 p
MOG LOC285830 NA Body open sea Body - open sea
NA -0.012726151 -0.037195081 -0.02446893
0.342146081
-0.026496599 0.011271254 -2.399841351 0.022370857
0.58037298 -4.697972503 cg08190562 6 29528774 p
GABBR1/MOG UBD NA TSS1500 open sea TSS1500 - open
sea NA 0.02037946 -0.006117139 -0.026496599 -
3.331534565
0.01495634 -0.006759235 2.394624971 0.022641367
0.58037298 -4.708864442 cg07375883 18 3448693 p
DLGAP1 TGIF1 NA 5'UTR island 5'UTR - island
NA -0.011900477 0.003055864 0.01495634 -
3.89430866
-0.031893929 0.009454401 -2.392992615 0.022726627
0.58037298 -4.712269457 cg04018625 2 171608293 q
GAD1 SP5 36436 IGR open sea IGR - open sea
NA 0.020417939 -0.01147599 -0.031893929
-1.779187664
-0.021726968 -0.055490145 -2.388771015 0.022948481
0.58037298 -4.72106801 cg19227924 17 28565709 q
SLC6A4 BLMH -9504 IGR shelf IGR - shelf
NA -0.048021499 -0.069748467 -0.021726968
0.688495406
0.010249156 -0.007584437 2.383719239 0.023216551
0.58037298 -4.731582558 cg06570025 21 34444245 q
OLIG2 OLIG1 NA 1stExon island 1stExon - island
NA -0.011107584 -0.000858428 0.010249156
12.93944273
-0.020319395 -0.031408345 -2.37524325 0.023672726
0.58037298 -4.749189173 cg10217445 21 34397784 q
OLIG2 OLIG2 NA TSS1500 island TSS1500 - island
NA -0.024423553 -0.044742948 -0.020319395
0.545863742
-0.019149719 -0.016764027 -2.372985773 0.023795588
0.58037298 -4.753871082 cg02681389 6 29570781 p
GABBR1/MOG GABBR1 NA 3'UTR open sea 3'UTR - open sea
NA -0.010181311 -0.02933103 -0.019149719
0.347117409
-0.029583864 -0.002653547 -2.368082672 0.024064433
0.58037298 -4.764029151 cg25252977 6 29696650 p
MOG LOC285830 NA Body open sea Body - open sea

NA	0.007515906	-0.022067958	-0.029583864
-0.340580048
-0.012314095	0.000918012	-2.35789368	0.024631949
0.58037298	-4.785091152	cg04718263	6	29554942	p
GABBR1/MOG	OR2H2	NA	TSS1500	open sea	TSS1500 - open
sea	NA	0.005150982	-0.007163113	-0.012314095
-0.719098175
-0.012944929	-0.004376837	-2.353011207	0.024908177
0.58037298	-4.795161226	cg06087028	6	29526389	p
GABBR1/MOG	UBD	NA	Body	open sea	Body - open sea
NA	7.30E-05	-0.012871947	-0.012944929	-
0.005669845
-0.062188553	0.009481837	-2.343563983	0.025450644
0.58037298	-4.814604267	cg11383134	6	29648590	p
GABBR1/MOG	ZFP57	8421	IGR	open sea	IGR - open sea
NA	0.030859152	-0.031329401	-0.062188553
-0.984990171
0.018896865	-0.002660923	2.343489642	0.025454955
0.58037298	-4.814757048	cg01558660	9	4679986	p
SLC1A1	CDC37L1	NA	Body	island	Body - island
NA	-0.00915672	0.009740145	0.018896865
-0.940101027
-0.036572433	0.028382736	-2.319555542	0.026877699
0.590925016	-4.863765873	cg18004110	6
29589729	p	GABBR1/MOG	GABBR1	NA	Body	open
sea	Body - open sea	NA	0.04095451	0.004382076	-
0.036572433	9.345914339
-0.012686575	-0.004393295	-2.313651004	0.027239569
0.590925016	-4.875801324	cg15543523	6
152127812	q	ESR1	ESR1	NA	TSS1500	shore
TSS1500 - shore	NA	-3.23E-05	-0.01271886	-
0.012686575	0.002538317
0.011925727	-0.033412194	2.300942411	0.028033409
0.590925016	-4.901631435	cg11215918	21
34395699	q	OLIG2	OLIG2	-2517	IGR	island
IGR - island	NA	-0.037511662	-0.025585936
0.011925727	1.46610476
-0.006289639	0.000995583	-2.298577434	0.02818342
0.590925016	-4.906426998	cg03202557	6
29617599	p	GABBR1/MOG	MOG	-7159	IGR	shore
IGR - shore	NA	0.003157647	-0.003131992
-0.006289639	-1.008191184
-0.009646963	-0.004764667	-2.297495203	0.028252308
0.590925016	-4.908620305	cg25669230	12
72332708	q	TPH2	TPH2	NA	1stExon	open
sea	1stExon - open sea	NA	-0.001448524	-0.011095487
-0.009646963	0.130550713
-0.008428231	-0.000605	-2.292435353	0.028576403
0.590925016	-4.918865045	cg17869426	21
34442160	q	OLIG2	OLIG1	NA	TSS1500	shore
TSS1500 - shore	NA	0.002292204	-0.006136027
-0.008428231	-0.373564852
0.02180732	-0.004000282	2.279629257	0.029411693
0.590925016	-4.944721168	cg12760563	18	3594396
p	DLGAP1	DLGAP1	NA	Body	open sea	Body -
open sea	NA	-0.011496549	0.010310772	0.02180732	-
1.115003748

-0.018203883 -0.042449521 -2.274962241 0.02972153
0.590925016 -4.954118168 cg09926027 9
87285693 q NTRK2 NTRK2 NA Body island
Body - island NA -0.036191936 -0.054395819
-0.018203883 0.66534408
-0.021146574 0.018194757 -2.27370603 0.029805428
0.590925016 -4.956645164 cg03639249 6
29574225 p GABBR1/MOG GABBR1 NA Body open
sea Body - open sea NA 0.025463891 0.004317317
-0.021146574 5.898082006
0.009761515 -0.005571235 2.264541815 0.030423955
0.594571007 -4.975049419 cg02736969 21
34442674 q OLIG2 OLIG1 NA 1stExon island
1stExon - island NA -0.008926756 0.000834759
0.009761515 -10.69381365
-0.020654356 -0.002552611 -2.247044427 0.031637064
0.609570465 -5.010039347 cg11441173 6
29707887 p MOG LOC285830 NA Body open
sea Body - open sea NA 0.004547324 -0.016107031
-0.020654356 -0.282319192
-0.026107347 -0.010874258 -2.239313085 0.032186787
0.609702133 -5.025437033 cg23148731 6
29697938 p MOG LOC285830 NA Body open
sea Body - open sea NA -0.001899858 -0.028007205
-0.026107347 0.067834617
-0.014637498 -0.006516189 -2.232229579 0.032697953
0.609702133 -5.039510529 cg24444839 21
34350730 q OLIG2 OLIG2 -47486 IGR island
IGR - island NA -0.001484549 -0.016122047
-0.014637498 0.092081909
-0.021564907 0.003049763 -2.228350345 0.032980963
0.609702133 -5.047203984 cg21022792 6
29589798 p GABBR1/MOG GABBR1 NA Body open
sea Body - open sea NA 0.0104627 -0.011102207 -
0.021564907 -0.942398163
-0.006957049 -0.003605458 -2.212102368 0.034190334
0.613822613 -5.079321122 cg13477819 6
29617973 p GABBR1/MOG MOG -6785 IGR island
IGR - island NA -0.001213972 -0.008171022
-0.006957049 0.148570464
-0.018382269 0.004199772 -2.210482233 0.034313074
0.613822613 -5.082514162 cg16150863 6
29456577 p GABBR1 MAS1L NA TSS1500 open
sea TSS1500 - open sea NA 0.010518677 -0.007863591
-0.018382269 -1.33764292
-0.033141115 -0.033684844 -2.194071141 0.035578754
0.613822613 -5.114760837 cg20265360 2
172756307 q SLC25A12 HAT1 -22628 IGR open
sea IGR - open sea NA -0.022292586 -0.055433701
-0.033141115 0.40214861
-0.023899156 0.031173633 -2.19007542 0.035893162
0.613822613 -5.122585318 cg01465527 6
29548535 p GABBR1/MOG SNORD32B NA TSS1500 open
sea TSS1500 - open sea NA 0.039388968 0.015489812
-0.023899156 2.542895188
-0.010548941 0.001345444 -2.189796872 0.035915173
0.613822613 -5.123130381 cg01455471 6

29600468 p GABBR1/MOG GABBR1 NA 5'UTR island
5'UTR - island NA 0.004971642 -0.005577299
-0.010548941 -0.891406763
-0.022634134 0.003369159 -2.189780104 0.035916498
0.613822613 -5.123163191 cg23306453 6
29599012 p GABBR1/MOG GABBR1 NA Body shore
Body - shore NA 0.011149642 -0.011484492
-0.022634134 -0.970843355
-0.01179616 0.002267158 -2.184390536 0.03634476
0.613822613 -5.133699366 cg02887726 6
29641082 p GABBR1/MOG ZFP57 NA Body open
sea Body - open sea NA 0.006322088 -0.005474072
-0.01179616 -1.15491501
-0.030324655 0.009220426 -2.174218224 0.037165497
0.62002926 -5.153533 cg02613510 11 27723789 p BDNF
BDNF NA Body shore Body - shore NA
0.019644526 -0.010680128 -0.030324655 -
1.83935307
-0.03537982 0.08656069 -2.164150886 0.037993988
0.622625888 -5.173094252 cg17825311 6
29717188 p MOG LOC285830 NA TSS1500 shore
TSS1500 - shore NA 0.098722503 0.063342683
-0.03537982 1.558546285
0.021753683 0.018244277 2.160343694 0.038311561
0.622625888 -5.180474175 cg25077271 6
29614368 p GABBR1/MOG MOG -10390 IGR shelf
IGR - shelf NA 0.010766449 0.032520131
0.021753683 0.33107027
-0.0257953 -0.00719572 -2.151746878 0.03903736 0.622625888
-5.197102729 cg20322862 18 3412088 p DLGAP1
TGIF1 NA 5'UTR open sea 5'UTR - open sea NA
0.001671414 -0.024123886 -0.0257953 -0.069284608
-0.015461031 -0.0002352 -2.149164855 0.039257726
0.622625888 -5.202087387 ch.2.3493243F 2
172737063 q SLC25A12 SLC25A12 NA Body open
sea Body - open sea NA 0.005079529 -0.010381502
-0.015461031 -0.489286533
-0.01645946 0.003205842 -2.125885272 0.041295005
0.622625888 -5.24682659 cg13599596 9 4541807
p SLC1A1 SLC1A1 NA Body open sea Body -
open sea NA 0.008863781 -0.007595678 -0.01645946
-1.166950584
-0.014914859 -0.002005095 -2.117588266 0.042043458
0.622625888 -5.262683399 cg01095157 2
171784674 q GAD1 GORASP2 NA TSS1500 shore
TSS1500 - shore NA 0.003121888 -0.011792971
-0.014914859 -0.264724487
-0.008682041 -0.000705557 -2.117373625 0.042062979
0.622625888 -5.26309299 cg05237001 6
29691245 p GABBR1/MOG HLA-F NA 1stExon island
1stExon - island NA 0.002278895 -0.006403146
-0.008682041 -0.355902365
0.010102016 -0.008843211 2.114077885 0.042363723
0.622625888 -5.269378184 cg06106763 21
34444104 q OLIG2 OLIG1 NA 1stExon island
1stExon - island NA -0.012315779 -0.002213763
0.010102016 5.563278439

-0.024582454 -0.045865947 -2.103717607 0.04332156
0.622625888 -5.289087747 cg08093277 6
29595299 p GABBR1/MOG GABBR1 NA Body island
Body - island NA -0.037415728 -0.061998182
-0.024582454 0.603497177
-0.028933137 -0.062151441 -2.102928451 0.043395299
0.622625888 -5.290586048 cg17264941 6
29698405 p MOG LOC285830 NA Body open
sea Body - open sea NA -0.052205675 -0.081138813
-0.028933137 0.643411871
0.014472662 -0.00218298 2.100365929 0.043635508
0.622625888 -5.295448348 ch.2.3495108F 2
172804051 q SLC25A12 HAT1 NA Body open
sea Body - open sea NA -0.007157957 0.007314705
0.014472662 -0.978570863
-0.030622048 -0.0020618 -2.099333152 0.043732652
0.622625888 -5.297406738 cg25633045 17
28458728 q SLC6A4 CCDC55 NA Body open
sea Body - open sea NA 0.008464529 -0.022157519
-0.030622048 -0.382016123
-0.02761892 0.008657501 -2.098566049 0.04380493
0.622625888 -5.298860875 cg24034959 6
29524905 p GABBR1/MOG UBD NA Body shelf
Body - shelf NA 0.018151504 -0.009467415
-0.02761892 -1.917260793
-0.019115548 -0.002746291 -2.094107751 0.044227093
0.622625888 -5.307304146 cg27474149 6
29572333 p GABBR1/MOG GABBR1 NA Body open
sea Body - open sea NA 0.003824678 -0.015290869
-0.019115548 -0.250128253
0.022460684 0.010442152 2.0905447 0.044567065
0.622625888 -5.314042171 cg24058145 6
29523592 p GABBR1 UBD NA 3'UTR shore
3'UTR - shore NA 0.002721292 0.025181976
0.022460684 0.108065056
-0.019904157 0.001467529 -2.090054595 0.044614009
0.622625888 -5.314968318 cg13499155 12
72376553 q TPH2 TPH2 NA Body open
sea Body - open sea NA 0.008309583 -0.011594574
-0.019904157 -0.716678568
-0.016968415 -0.040506975 -2.085436942 0.045058452
0.622625888 -5.323686159 cg05171584 6
152128535 q ESR1 ESR1 NA TSS1500 shore
TSS1500 - shore NA -0.034674083 -0.051642497
-0.016968415 0.671425363
-0.05376783 0.016617421 -2.075369471 0.046041006
0.623739287 -5.342642023 cg15570656 6
29648628 p GABBR1/MOG ZFP57 8459 IGR open
sea IGR - open sea NA 0.035100112 -0.018667718
-0.05376783 -1.880257238
0.086068677 -0.06437597 2.075268787 0.046050927
0.623739287 -5.342831246 cg24900542 6
29723315 p MOG IFITM4P 4731 IGR shelf
IGR - shelf NA -0.093962077 -0.0078934
0.086068677 11.90387821
0.073640249 0.128327802 2.058839 0.047695334
0.625269787 -5.373615019 cg21114334 6

29720137 p MOG IFITM4P NA TSS1500 shore
TSS1500 - shore NA 0.103013967 0.176654216
0.073640249 0.583139022
-0.01372253 -0.000281751 -2.055261836 0.048060139
0.625269787 -5.380292569 cg22659852 6
152464820 q ESR1 SYNE1 NA Body open
sea Body - open sea NA 0.004435369 -0.009287161
-0.01372253 -0.477580739
0.011414307 -0.004199293 2.054694319 0.04811824
0.625269787 -5.381351144 cg03837627 17
28443756 q SLC6A4 CCDC55 NA TSS200 island
TSS200 - island NA -0.00812296 0.003291346
0.011414307 -2.467975181
-0.051609869 0.013952448 -2.051939725 0.048401129
0.625269787 -5.386486032 cg03449857 6
29648623 p GABBR1/MOG ZFP57 8454 IGR open
sea IGR - open sea NA 0.03169334 -0.019916529 -
0.051609869 -1.591308451
0.017735237 -0.021704946 2.051472399 0.048449267
0.625269787 -5.387356659 cg21632158 6
29521356 p GABBR1 UBD -2033 IGR island
IGR - island NA -0.027801434 -0.010066197
0.017735237 2.761860626
-0.011885422 -0.002088276 -2.043294739 0.049298462
0.630283147 -5.402566951 cg09539438 9
87283789 q NTRK2 NTRK2 NA TSS1500 island
TSS1500 - island V$MEIS1AHOXA9_01 0.001997338 -
0.009888084 -0.011885422 -0.201994411
-0.018254555 -0.00422373 -2.031787298 0.050515568
0.633824035 -5.423891376 cg21167956 9 4603153
p SLC1A1 C9orf68 NA 3'UTR open sea 3'UTR -
open sea NA 0.002051273 -0.016203282 -0.018254555
-0.126596135
-0.03022879 0.129793843 -2.031688849 0.050526094
0.633824035 -5.424073412 cg24531536 6
29520698 p GABBR1 UBD -2691 IGR shore
IGR - shore NA 0.140184989 0.109956199
-0.03022879 1.274916655
-0.034855186 0.014596335 -2.020277436 0.051759219
0.633824035 -5.445127304 cg18354203 11
27696004 p BDNF BDNF NA Body open
sea Body - open sea NA 0.026577805 -0.008277381
-0.034855186 -3.210895586
-0.015148529 0.000712553 -2.015952821 0.052233384
0.633824035 -5.45308219 cg15054873 6
152464791 q ESR1 SYNE1 NA Body open
sea Body - open sea NA 0.00591986 -0.009228669 -
0.015148529 -0.64146409
-0.018581734 0.012597788 -2.015108791 0.052326369
0.633824035 -5.454633196 cg13821571 21
34406537 q OLIG2 OLIG2 8321 IGR island
IGR - island NA 0.018985259 0.000403526
-0.018581734 47.04845084
-0.01891692 -9.47E-06 -2.014097083 0.052438017
0.633824035 -5.456491668 cg00028318 6
29570739 p GABBR1/MOG GABBR1 NA 3'UTR open

sea 3'UTR - open sea NA 0.006493224 -0.012423696
-0.01891692 -0.522648319
-0.016561895 0.038457144 -2.010661884 0.05281867
0.633824035 -5.462796607 cg16580937 21
34347126 q OLIG2 OLIG2 -51090 IGR shelf
IGR - shelf NA 0.044150295 0.0275884 -
0.016561895 1.600320983
-0.010097048 -0.004620237 -1.996016321 0.054468788
0.643761919 -5.489583172 cg18861140 18 3449789
p DLGAP1 TGIF1 NA 5'UTR island 5'UTR -
island NA -0.001149377 -0.011246424 -0.010097048
0.102199299
-0.009710604 -0.011394932 -1.994973353 0.054587999
0.643761919 -5.491484938 cg22839866 6
152128584 q ESR1 ESR1 NA TSS1500 shore
TSS1500 - shore NA -0.008056912 -0.017767516
-0.009710604 0.45346305
-0.035888249 0.017611916 -1.980792354 0.056231629
0.64591051 -5.517265901 cg25804443 18 3875823 p
DLGAP1 DLGAP1 NA Body shelf Body - shelf
NA 0.029948502 -0.005939747 -0.035888249
-5.042049691
-0.031307825 -0.084080129 -1.972858214 0.057169919
0.64591051 -5.531627415 cg22584138 17 28562220 q
SLC6A4 SLC6A4 NA 5'UTR shore 5'UTR - shore
NA -0.073318065 -0.104625889 -0.031307825
0.700764075
-0.039606311 -0.079733037 -1.971896055 0.057284625
0.64591051 -5.533365943 cg21100518 6 29595002 p
GABBR1/MOG GABBR1 NA Body shore Body - shore
NA -0.066118368 -0.105724679 -0.039606311
0.625382534
-0.022877825 0.009791393 -1.969625007 0.05755617
0.64591051 -5.537466873 cg17014345 6 29716186 p
MOG LOC285830 NA Body shore Body - shore
NA 0.017655646 -0.005222179 -0.022877825
-3.380896249
-0.015108345 0.028607501 -1.957247832 0.059055859
0.64591051 -5.559751693 cg16912910 6 29497165 p
GABBR1 LINC01015 -18 IGR open sea IGR - open sea
NA 0.033800995 0.01869265 -0.015108345
1.808250594
0.014523605 -0.008404801 1.955664196 0.059250172
0.64591051 -5.562595029 cg13015908 21 34481860 q
OLIG2 OLIG1 39410 IGR open sea IGR - open sea
NA -0.013397291 0.001126314 0.014523605
-11.89480435
0.007917059 -0.007772457 1.955264495 0.059299303
0.64591051 -5.563312385 cg27280904 21 34443601 q
OLIG2 OLIG1 NA 1stExon island 1stExon - island
NA -0.010493946 -0.002576888 0.007917059
4.07233388
-0.027667378 0.00737943 -1.951877715 0.059717027
0.64591051 -5.569386122 cg05292788 6 29586060 p
GABBR1/MOG GABBR1 NA Body open sea Body - open sea
NA 0.016890092 -0.010777286 -0.027667378
-1.567193356

-0.064713561 -0.000180818 -1.950228942 0.05992131
0.64591051 -5.572339976 cg13835168 6 29648756 p
GABBR1/MOG ZFP57 8587 IGR open sea IGR - open sea
NA 0.022064468 -0.042649092 -0.064713561
-0.517349072
-0.017714777 0.017819825 -1.950176542 0.059927812
0.64591051 -5.57243382 cg05189570 11 27680480 p
BDNF BDNF NA Body open sea Body - open sea
NA 0.023909279 0.006194502 -0.017714777
3.859757917
-0.011400194 0.001804859 -1.949885692 0.059963914
0.64591051 -5.572954677 cg07511633 6 29455331 p
GABBR1 MAS1L NA 1stExon open sea 1stExon - open
sea NA 0.005723675 -0.005676519 -0.011400194
-1.008307304
0.073136383 -0.057635717 1.943979911 0.060701079
0.648742787 -5.583517553 cg18505691 6
29723320 p MOG IFITM4P 4736 IGR shelf
IGR - shelf NA -0.082776349 -0.009639966
0.073136383 8.586788693
-0.010561845 0.000544418 -1.926668693 0.062907348
0.661058639 -5.614333965 cg00923678 6
29581105 p GABBR1/MOG GABBR1 NA Body open
sea Body - open sea V$PAX5_01 0.004175052 -0.006386793
-0.010561845 -0.653700841
-0.017037724 -0.002841534 -1.921185402 0.063620505
0.661058639 -5.624049491 cg07463294 12
72243682 q TPH2 TBC1D15 NA Body open
sea Body - open sea NA 0.003015183 -0.014022541
-0.017037724 -0.215024026
-0.009884074 -0.004986749 -1.918917243 0.063917541
0.661058639 -5.628061891 cg05720454 21
34442511 q OLIG2 OLIG1 NA 1stExon island
1stExon - island NA -0.001589099 -0.011473173
-0.009884074 0.138505604
-0.026791478 0.112387906 -1.917928106 0.064047452
0.661058639 -5.629810508 cg21481950 6
29601498 p GABBR1/MOG GABBR1 NA TSS1500 shore
TSS1500 - shore NA 0.121597477 0.094805999
-0.026791478 1.282592645
-0.009229494 -0.004608949 -1.908936408 0.065238908
0.661058639 -5.645673368 cg23268677 22
19929097 q COMT TXNRD2 NA Body island
Body - island NA -0.001436311 -0.010665805
-0.009229494 0.134665043
0.01860796 -0.009512802 1.892416683 0.067477799
0.661058639 -5.674662183 cg21224669 6
29639803 p GABBR1/MOG MOG NA 3'UTR open
sea 3'UTR - open sea NA -0.015909288 0.002698672
0.01860796 -5.895228648
-0.006500361 -0.001155021 -1.892258235 0.06749959
0.661058639 -5.674939254 cg03760316 18 3594197
p DLGAP1 DLGAP1 NA Body open sea Body -
open sea NA 0.001079478 -0.005420883 -0.006500361
-0.199133231
0.02184002 -0.005604046 1.891280005 0.067634257
0.661058639 -5.676649434 cg04836472 21

34405997 q OLIG2 OLIG2 7781 IGR island
IGR - island NA -0.013111553 0.008728467
0.02184002 -1.502159808
-0.029545612 0.014238283 -1.890211526 0.067781613
0.661058639 -5.678516582 cg01715172 6
152128024 q ESR1 ESR1 NA TSS1500 shore
TSS1500 - shore NA 0.024394587 -0.005151025
-0.029545612 -4.735870201
-0.015023265 -0.008844035 -1.881121908 0.069046394
0.661058639 -5.694366407 cg15385139 6
29595506 p GABBR1/MOG GABBR1 NA Body island
Body - island NA -0.003679787 -0.018703052
-0.015023265 0.196747967
-0.02386393 0.008657655 -1.868431996 0.070846059
0.661058639 -5.716391747 cg04615964 6
29591153 p GABBR1/MOG GABBR1 NA Body open
sea Body - open sea NA 0.016860881 -0.007003049
-0.02386393 -2.407648685
-0.050911708 -0.001310529 -1.862319422 0.071727213
0.661058639 -5.726958355 cg20140394 18 4456154
p DLGAP1 DLGAP1-AS5 191552 IGR shore IGR -
shore NA 0.016190371 -0.034721338 -0.050911708
-0.466294553
-0.014507293 -0.003265946 -1.853201223 0.073059105
0.661058639 -5.742668896 cg16472542 22
20020575 q COMT C22orf25 NA 5'UTR open
sea 5'UTR - open sea NA 0.001720936 -0.012786357
-0.014507293 -0.134591605
-0.045438801 0.012579449 -1.851356846 0.073331074
0.661058639 -5.74583918 cg27602828 18 3845348
p DLGAP1 DLGAP1 NA TSS200 open sea TSS200
- open sea NA 0.028199037 -0.017239763 -0.045438801
-1.635697454
-0.025378401 -0.009917882 -1.849559409 0.073596955
0.661058639 -5.748926328 cg18014983 6
29618382 p GABBR1/MOG MOG -6376 IGR shore
IGR - shore NA -0.001194057 -0.026572458
-0.025378401 0.044935884
-0.017783884 -0.005695934 -1.849120388 0.073662021
0.661058639 -5.749679992 cg13210820 6
29632605 p GABBR1/MOG MOG NA 3'UTR open
sea 3'UTR - open sea NA 0.000417276 -0.017366608
-0.017783884 -0.024027503
0.015431881 -0.012856118 1.845950189 0.074133332
0.661058639 -5.755117957 cg13927803 6
29524042 p GABBR1 UBD NA Body shelf
Body - shelf NA -0.018160827 -0.002728947
0.015431881 6.654885553
-0.042234195 0.019484169 -1.844326953 0.074375655
0.661058639 -5.757899441 cg13504410 2
172546870 q SLC25A12 DYNC1I2 NA Body shelf
Body - shelf NA 0.034002173 -0.008232022
-0.042234195 -4.130476622
-0.01815955 0.006631601 -1.83884242 0.075199425
0.661058639 -5.767282791 cg09963892 15
88496177 q NTRK3 NTRK3 NA Body open

sea Body - open sea NA 0.012873946 -0.005285604
-0.01815955 -2.43566239
-0.018074211 -0.003598266 -1.835526819 0.075701197
0.661058639 -5.772944407 cg13311832 6
29549147 p GABBR1/MOG SNORD32B NA TSS1500 open
sea TSS1500 - open sea NA 0.002614744 -0.015459467
-0.018074211 -0.169135431
0.017201226 -0.013121478 1.833043649 0.076078864
0.661058639 -5.777179175 cg21153898 6
29521788 p GABBR1 UBD -1601 IGR island
IGR - island NA -0.0190344 -0.001833174
0.017201226 10.38330218
0.012465141 -0.026954138 1.832634444 0.076141254
0.661058639 -5.777876582 cg22377998 22
20004881 q COMT ARVCF NA TSS1500 shore
TSS1500 - shore NA -0.03123903 -0.018773889
0.012465141 1.663961577
-0.037192243 0.018157689 -1.828703426 0.076742834
0.661058639 -5.784569782 cg02754084 12
72338080 q TPH2 TPH2 NA Body open
sea Body - open sea NA 0.030942523 -0.00624972
-0.037192243 -4.951025408
-0.009291435 -0.003925739 -1.828643323 0.076752064
0.661058639 -5.784672028 cg21372810 2
172542111 q SLC25A12 DYNC1I2 -1871 IGR shore
IGR - shore NA -0.000731808 -0.010023243
-0.009291435 0.073011125
-0.018958183 0.00195886 -1.824293065 0.077422595
0.661058639 -5.792065269 cg19930203 22
19956281 q COMT COMT NA 3'UTR open
sea 3'UTR - open sea NA 0.008475736 -0.010482447
-0.018958183 -0.808564567
-0.020739314 -0.004385053 -1.821241505 0.077895928
0.661058639 -5.797242853 cg22561592 15
88320395 q NTRK3 NTRK3-AS1 -99593 IGR open
sea IGR - open sea NA 0.002744086 -0.017995228
-0.020739314 -0.152489679
0.028478755 -0.041625078 1.818548601 0.078315675
0.661058639 -5.801806059 cg06486622 6
29718119 p MOG LOC285830 NA TSS1500 shore
TSS1500 - shore NA -0.051414651 -0.022935895
0.028478755 2.241667476
-0.010176726 -0.003491973 -1.817897592 0.078417438
0.661058639 -5.802908389 cg26741280 17
28563089 q SLC6A4 SLC6A4 NA TSS200 island
TSS200 - island NA 6.28E-06 -0.010170449 -
0.010176726 -0.000617121
-0.019762524 0.009809759 -1.817230059 0.0785219
0.661058639 -5.804038364 cg25329933 6
29636669 p GABBR1/MOG MOG NA 3'UTR open
sea 3'UTR - open sea NA 0.016603127 -0.003159397
-0.019762524 -5.255156582
-0.01128419 -0.017740834 -1.816715865 0.078602448
0.661058639 -5.804908544 cg05265512 18 3447583
p DLGAP1 TGIF1 NA 5'UTR shore 5'UTR -
shore NA -0.013861893 -0.025146084 -0.01128419
0.551254558

-0.020583321 -0.002991498 -1.810412276 0.079595626
0.661058639 -5.815559941 cg13209878 6
29586025 p GABBR1/MOG GABBR1 NA Body open
sea Body - open sea NA 0.004084018 -0.016499302
-0.020583321 -0.247526725
-0.01180872 -0.012622166 -1.802148633 0.080913788
0.661058639 -5.82947763 cg02450267 6
29627395 p GABBR1/MOG MOG NA Body open
sea Body - open sea NA -0.008562918 -0.020371638
-0.01180872 0.420335286
-0.020755821 0.004389375 -1.797363016 0.081685611
0.661058639 -5.837513855 cg23266797 6
29636003 p GABBR1/MOG MOG NA 3'UTR open
sea 3'UTR - open sea NA 0.011524188 -0.009231633
-0.020755821 -1.248336948
-0.012914956 0.024351469 -1.797306579 0.08169475
0.661058639 -5.837608522 cg24710480 6
29717136 p MOG LOC285830 NA TSS1500 island
TSS1500 - island NA 0.028790985 0.015876029
-0.012914956 1.813487835
-0.022457091 0.062235442 -1.797108656 0.081726809
0.661058639 -5.837940499 cg04517749 6
29570507 p GABBR1/MOG GABBR1 NA 3'UTR open
sea 3'UTR - open sea V$OCT_C 0.069955067 0.047497976
-0.022457091 1.472801007
-0.019734813 -0.025683444 -1.794952624 0.082076721
0.661058639 -5.841554886 cg24764793 6
152126745 q ESR1 ESR1 NA 5'UTR shelf
5'UTR - shelf NA -0.018899602 -0.038634415
-0.019734813 0.489190843
-0.014489104 -0.01196808 -1.792927093 0.082406612
0.661058639 -5.844947272 cg14841796 6
29598285 p GABBR1/MOG GABBR1 NA Body shore
Body - shore NA -0.006987451 -0.021476555
-0.014489104 0.325352494
0.006908011 -0.000907964 1.790597962 0.082787339
0.661058639 -5.84884426 cg05901579 15
88800567 q NTRK3 NTRK3 NA TSS1500 island
TSS1500 - island NA -0.003282593 0.003625418
0.006908011 -0.905438514
-0.016034025 0.011362288 -1.787053745 0.083369552
0.661058639 -5.85476632 cg15437231 12
72259306 q TPH2 TBC1D15 NA Body open
sea Body - open sea NA 0.016873984 0.000839959
-0.016034025 20.08904512
-0.027257056 0.007864084 -1.782060593 0.084195673
0.661058639 -5.863093117 cg04321753 6
29586923 p GABBR1/MOG GABBR1 NA Body open
sea Body - open sea NA 0.017233697 -0.010023359
-0.027257056 -1.719353561
0.009231083 -0.008539497 1.779382226 0.084641662
0.661058639 -5.867551812 cg05016953 17
28562813 q SLC6A4 SLC6A4 NA 1stExon island
1stExon - island NA -0.011712682 -0.002481599
0.009231083 4.719813135
0.035313147 -0.097219337 1.770789872 0.08608595
0.661058639 -5.881818418 cg06301399 6

29634495 p GABBR1/MOG MOG NA 3'UTR open
sea 3'UTR - open sea NA -0.109358231 -0.074045085
0.035313147 1.476914125
-0.011148399 0.01087636 -1.768354141 0.086499144
0.661058639 -5.88585235 cg07854670 2
171667934 q GAD1 GAD1 -5266 IGR shelf
IGR - shelf NA 0.014708622 0.003560223
-0.011148399 4.131376466
-0.006024576 -0.003769776 -1.767406059 0.086660429
0.661058639 -5.88742128 cg12766058 6
29720894 p MOG IFITM4P 2310 IGR island
IGR - island NA -0.001698828 -0.007723404
-0.006024576 0.219958416
-0.01802241 0.009795557 -1.764419032 0.087170235
0.661058639 -5.892359829 cg05358170 6
29692399 p GABBR1/MOG HLA-F NA Body shore
Body - shore NA 0.015990761 -0.00203165
-0.01802241 -7.870826083
-0.025135218 0.008819596 -1.764054839 0.087232566
0.661058639 -5.89296149 cg03063857 6
29585617 p GABBR1/MOG GABBR1 NA Body open
sea Body - open sea NA 0.017459827 -0.007675391
-0.025135218 -2.274780069
0.007481754 -0.006125655 1.761269808 0.087710465
0.661058639 -5.897559099 cg06087185 6
29521499 p GABBR1 UBD -1890 IGR island
IGR - island NA -0.008697508 -0.001215754
0.007481754 7.15400517
-0.02980806 -0.098044022 -1.760779838 0.087794771
0.661058639 -5.898367337 cg06519422 6
29599226 p GABBR1/MOG GABBR1 NA Body shore
Body - shore NA -0.087797501 -0.117605561
-0.02980806 0.746542091
-0.016336547 0.083802562 -1.75936578 0.08803846
0.661058639 -5.900698882 cg11742207 15
88784902 q NTRK3 NTRK3 NA Body open
sea Body - open sea NA 0.08941825 0.073081703 -
0.016336547 1.223538132
-0.014530114 0.000245438 -1.758976136 0.088105709
0.661058639 -5.901341067 cg13013381 6
29600103 p GABBR1/MOG GABBR1 NA Body shore
Body - shore V$TAXCREB_02 0.005240165 -
0.009289949 -0.014530114 -0.56406816
-0.027262072 0.004955891 -1.75734537 0.088387635
0.661058639 -5.904027521 cg06898502 18 3410587
p DLGAP1 TGIF1 NA TSS1500 open sea TSS1500
- open sea NA 0.014327229 -0.012934843 -0.027262072
-1.107646078
-0.009368172 -0.005529482 -1.75584236 0.088648148
0.661058639 -5.906501698 cg23497217 11
27723214 p BDNF BDNF NA TSS1500 shore
TSS1500 - shore NA -0.002309173 -0.011677345
-0.009368172 0.197748138
-0.040460576 0.017156524 -1.755646075 0.088682217
0.661058639 -5.906824682 cg16885113 6
29648507 p GABBR1/MOG ZFP57 8338 IGR open

sea IGR - open sea NA 0.031064847 -0.009395729
-0.040460576 -3.306273036
-0.026537075 0.037057656 -1.755041205 0.088787275
0.661058639 -5.907819804 cg01405582 6
29692365 p GABBR1/MOG HLA-F NA Body shore
Body - shore NA 0.046179776 0.019642701
-0.026537075 2.35098916
-0.017477301 0.008577143 -1.752498322 0.089230087
0.661058639 -5.912000213 cg19548470 18 3880510
p DLGAP1 DLGAP1 NA TSS1500 shore TSS1500
- shore NA 0.014584965 -0.002892336 -0.017477301
-5.042625427
-0.01051368 0.000751683 -1.751399458 0.089422017
0.661058639 -5.913805161 cg16922688 6
29588173 p GABBR1/MOG GABBR1 NA Body open
sea Body - open sea NA 0.00436576 -0.00614792 -
0.01051368 -0.710119942
-0.006194129 -0.0037242 -1.749620518 0.089733468
0.661058639 -5.916725193 cg12217400 6
29521604 p GABBR1 UBD -1785 IGR island
IGR - island NA -0.001594969 -0.007789097
-0.006194129 0.204769379
-0.008605112 -0.003683214 -1.748780916 0.08988078
0.661058639 -5.918102503 cg08447405 17
28619272 q SLC6A4 BLMH NA TSS200 island
TSS200 - island NA -0.000725207 -0.009330318
-0.008605112 0.077725804
-0.01341083 0.014018612 -1.744369692 0.090658102
0.661102922 -5.925329841 cg21080452 6
29589960 p GABBR1/MOG GABBR1 NA Body open
sea Body - open sea NA 0.018628585 0.005217754
-0.01341083 3.570230237
0.013956662 -0.001674281 1.742690121 0.090955551
0.661102922 -5.928077683 cg14121971 6
29621375 p GABBR1/MOG MOG -3383 IGR shelf
IGR - shelf NA -0.006471884 0.007484778
0.013956662 -0.86467277
-0.016698585 -0.00715804 -1.736107919 0.092129182
0.661102922 -5.938825312 cg00376979 21
34350727 q OLIG2 OLIG2 -47489 IGR island
IGR - island NA -0.001417901 -0.018116486
-0.016698585 0.078265802
0.011982937 0.004372525 1.73469081 0.092383517
0.661102922 -5.941134809 cg25490334 6
152387590 q ESR1 ESR1 NA Body open
sea Body - open sea NA 0.00025339 0.012236327
0.011982937 0.020707999
-0.021859141 0.038881122 -1.732398975 0.092796092
0.661102922 -5.944866561 cg13468667 18 3411996
p DLGAP1 TGIF1 NA TSS200 open sea TSS200
- open sea NA 0.046395202 0.02453606 -0.021859141
1.890898588
-0.020514766 0.027412257 -1.727826006 0.093623946
0.661102922 -5.952300427 cg15803671 18 3457981
p DLGAP1 TGIF1 NA 3'UTR open sea 3'UTR -
open sea NA 0.034464208 0.013949442 -0.020514766
2.470651406

0.029964815 0.10771284 1.727080913 0.093759418
0.661102922 -5.953510115 cg00390484 22
20019695 q COMT C22orf25 NA 5'UTR open
sea 5'UTR - open sea NA 0.097412435 0.12737725
0.029964815 0.764755366
0.005772891 0.000626174 1.724813301 0.094172725
0.661102922 -5.957189008 cg24675879 17
28444056 q SLC6A4 MIR423 NA TSS200 shore
TSS200 - shore NA -0.001358257 0.004414634
0.005772891 -0.307671449
0.005733089 -0.001151207 1.724465994 0.094236162
0.661102922 -5.957752111 cg20891813 6
29691735 p GABBR1/MOG HLA-F NA Body island
Body - island NA -0.003121956 0.002611133
0.005733089 -1.195632725
-0.005361219 5.09E-05 -1.717139243 0.095582802
0.667128944 -5.969609351 cg19655006 2
172750994 q SLC25A12 SLC25A12 NA TSS200 island
TSS200 - island NA 0.001893823 -0.003467396
-0.005361219 -0.546180233
0.024117485 -0.010728818 1.711514966 0.096627444
0.670996669 -5.978682918 cg17369088 17
28444068 q SLC6A4 MIR423 NA TSS200 shore
TSS200 - shore NA -0.019019204 0.005098281
0.024117485 -3.730512816
0.009948897 -0.007553839 1.696814452 0.099403125
0.686785228 -6.002281908 cg13933279 15
88418502 q NTRK3 NTRK3-AS1 -1486 IGR open
sea IGR - open sea NA -0.010973772 -0.001024875
0.009948897 10.70742718
0.030622406 -0.071689387 1.690182059 0.10067706
0.692091548 -6.012873375 cg14172108 21
34405553 q OLIG2 OLIG2 7337 IGR shore
IGR - shore NA -0.082215839 -0.051593434
0.030622406 1.593533002
-0.043142321 -0.001319805 -1.680802954 0.102501806
0.694461412 -6.027791919 cg24100841 6
29649024 p GABBR1/MOG ZFP57 8855 IGR open
sea IGR - open sea NA 0.013510368 -0.029631953
-0.043142321 -0.45593916
-0.016467373 0.006853761 -1.674897081 0.103664896
0.694461412 -6.037150204 cg20168024 6
29456611 p GABBR1 MAS1L NA TSS1500 open
sea TSS1500 - open sea NA 0.01251442 -0.003952953 -
0.016467373 -3.165841314
-0.037188538 -0.02240088 -1.672778614 0.10408477
0.694461412 -6.040500337 cg08041448 6
29648901 p GABBR1/MOG ZFP57 8732 IGR open
sea IGR - open sea NA -0.00961732 -0.046805858
-0.037188538 0.205472576
0.006726739 -0.006795322 1.672346004 0.104170685
0.694461412 -6.041184026 cg23253569 21
34398222 q OLIG2 OLIG2 NA TSS200 island
TSS200 - island NA -0.009107639 -0.0023809
0.006726739 3.825292965
0.004650007 0.000564543 1.670280658 0.104581673
0.694461412 -6.044446019 cg09805507 6

29691426 p GABBR1/MOG HLA-F NA Body island
Body - island NA -0.001033897 0.003616111
0.004650007 -0.285913979
-0.015569642 -0.003907957 -1.668648573 0.104907399
0.694461412 -6.047021329 cg14067066 6
29717475 p MOG LOC285830 NA TSS1500 shore
TSS1500 - shore NA 0.001444107 -0.014125535
-0.015569642 -0.102233784
-0.011496445 -0.003289625 -1.665103673 0.105617782
0.694461412 -6.052607635 cg07839627 6
29599836 p GABBR1/MOG GABBR1 NA Body shore
Body - shore NA 0.000662278 -0.010834167
-0.011496445 -0.061128615
-0.009257765 0.005528732 -1.664003861 0.10583899
0.694461412 -6.054338765 cg05661333 6
29600200 p GABBR1/MOG GABBR1 NA 5'UTR island
5'UTR - island NA 0.008711089 -0.000546676
-0.009257765 -15.93463971
-0.010533655 0.004998473 -1.663619112 0.105916466
0.694461412 -6.054944143 cg04852097 6
29431228 p GABBR1 OR2H1 NA 3'UTR open
sea 3'UTR - open sea NA 0.008619417 -0.001914239
-0.010533655 -4.50279085
-0.011850048 -0.000380133 -1.662717181 0.106098271
0.694461412 -6.056362812 cg09595044 6
29426132 p GABBR1 OR2H1 NA TSS200 open
sea TSS200 - open sea NA 0.003693321 -0.008156727
-0.011850048 -0.452794519
-0.013710802 0.021832058 -1.64654436 0.109402489
0.709864444 -6.081691454 cg06308109 6
29548691 p GABBR1/MOG SNORD32B NA TSS1500 open
sea TSS1500 - open sea NA 0.026545146 0.012834344
-0.013710802 2.068290033
-0.029421497 0.042601521 -1.641855526 0.11037623
0.709864444 -6.088995704 cg05866854 2
171784945 q GAD1 GORASP2 NA TSS1500 shore
TSS1500 - shore NA 0.052715161 0.023293663
-0.029421497 2.263068697
-0.013235156 -0.001707156 -1.641175073 0.110518135
0.709864444 -6.09005425 cg21652192 6
29571483 p GABBR1/MOG GABBR1 NA Body open
sea Body - open sea NA 0.002842429 -0.010392727
-0.013235156 -0.273501718
0.009752776 0.026473599 1.638303173 0.111118719
0.709864444 -6.094517835 cg13570637 22
20052305 q COMT C22orf25 NA 3'UTR open
sea 3'UTR - open sea NA 0.023121082 0.032873859
0.009752776 0.703327305
0.00828266 0.002372133 1.637383637 0.111311585
0.709864444 -6.095945607 cg25635805 6
29581054 p GABBR1/MOG GABBR1 NA Body open
sea Body - open sea NA -0.000475031 0.007807628
0.00828266 -0.060841971
-0.034856909 0.034705028 -1.636177647 0.111564953
0.709864444 -6.09781713 cg11587584 6
29692372 p GABBR1/MOG HLA-F NA Body shore

Body - shore NA 0.046687091 0.011830182
-0.034856909 3.946438907
-0.026970795 0.034779368 -1.627298233 0.113445139
0.718485879 -6.111560781 cg13150094 6
29714824 p MOG LOC285830 NA Body shore
Body - shore NA 0.044050579 0.017079783
-0.026970795 2.579106402
-0.022330921 0.00604081 -1.622940789 0.11437733 0.718607899
-6.118282107 cg15014679 11 27695210 p BDNF
BDNF NA Body open sea Body - open sea NA
0.013717064 -0.008613857 -0.022330921 -
1.592441535
-0.011760673 0.000256159 -1.622299738 0.114515001
0.718607899 -6.119269634 cg00886856 22
19960296 q COMT ARVCF NA Body shore
Body - shore NA 0.004298891 -0.007461782
-0.011760673 -0.576121147
0.006633978 -0.005928158 1.615939336 0.115888369
0.720316883 -6.129049783 cg04672351 11
27722889 p BDNF BDNF NA TSS1500 shore
TSS1500 - shore NA -0.008208587 -0.00157461
0.006633978 5.213093489
-0.017196117 -0.015568064 -1.614812969 0.116132987
0.720316883 -6.130778355 cg07579946 22
19949893 q COMT COMT NA 5'UTR open
sea 5'UTR - open sea NA -0.009656898 -0.026853016
-0.017196117 0.359620634
-0.00642611 0.002401205 -1.612019141 0.116741568
0.720316883 -6.135061467 cg09124190 18 3448419
p DLGAP1 TGIF1 NA 5'UTR island 5'UTR -
island V$HEN1_02 0.00461018 -0.00181593 -0.00642611 -
2.53874395
0.013845802 0.008277042 1.611323397 0.116893529
0.720316883 -6.136127106 cg18281744 6
29455512 p GABBR1 MAS1L NA 1stExon open
sea 1stExon - open sea NA 0.003517547 0.017363349
0.013845802 0.202584617
-0.021376349 0.006615142 -1.60701712 0.117837711
0.722878874 -6.142714129 cg13801656 9 4685060
p SLC1A1 CDC37L1 NA Body open sea Body -
open sea NA 0.013963262 -0.007413087 -0.021376349
-1.883596124
-0.017913418 -0.008611768 -1.603824553 0.118541741
0.723951348 -6.147587905 cg18356448 18 3881547
p DLGAP1 DLGAP1 NA TSS1500 shore TSS1500
- shore NA -0.00245403 -0.020367448 -0.017913418
0.120487863
0.009426321 -0.001546167 1.599552637 0.11948919
0.726494274 -6.154096509 cg24377657 11
27723245 p BDNF BDNF NA TSS1500 shore
TSS1500 - shore NA -0.004786465 0.004639856
0.009426321 -1.031597866
-0.012214926 -0.004759763 -1.593354645 0.12087485
0.728715896 -6.16351334 cg23908638 6
29579475 p GABBR1/MOG GABBR1 NA Body open
sea Body - open sea NA -0.000560883 -0.012775808
-0.012214926 0.043901922

0.005459532 -0.000877952 1.589886305 0.121655981
0.728715896 -6.168769305 cg17290446 12
72233346 q TPH2 TBC1D15 NA TSS200 island
TSS200 - island NA -0.002754666 0.002704865
0.005459532 -1.018411621
-0.014748371 0.005866561 -1.582078448 0.123429576
0.728715896 -6.180565634 cg03881768 6
29582192 p GABBR1/MOG GABBR1 NA Body open
sea Body - open sea NA 0.010936313 -0.003812058
-0.014748371 -2.868873734
-0.013798429 0.02706056 -1.579844539 0.123940892
0.728715896 -6.183931552 cg07016276 6
29692009 p GABBR1/MOG HLA-F NA Body island
Body - island NA 0.03180377 0.018005341 -
0.013798429 1.766351959
-0.007599943 -0.004165928 -1.575475823 0.124945842
0.728715896 -6.190502312 cg01543173 21
34442534 q OLIG2 OLIG1 NA 1stExon island
1stExon - island NA -0.001553448 -0.009153391
-0.007599943 0.169712792
-0.011518373 -0.001616184 -1.573371095 0.125432369
0.728715896 -6.193662364 cg10002133 15
88798448 q NTRK3 NTRK3 NA Body shore
Body - shore NA 0.002343257 -0.009175116
-0.011518373 -0.255392638
0.007522267 -0.002606244 1.570615962 0.126071578
0.728715896 -6.197793471 cg04648747 12
72233970 q TPH2 TBC1D15 NA Body shore
Body - shore NA -0.005192024 0.002330243
0.007522267 -2.228103824
0.004574931 -0.005342584 1.568421363 0.126582638
0.728715896 -6.201079662 cg15299832 21
34398131 q OLIG2 OLIG2 NA TSS200 island
TSS200 - island NA -0.006915216 -0.002340285
0.004574931 2.954860284
-0.015204841 -0.008254274 -1.566960502 0.126923766
0.728715896 -6.203264971 cg01426208 6
29720641 p MOG IFITM4P 2057 IGR island
IGR - island NA -0.00302761 -0.018232451
-0.015204841 0.166056135
-0.005470314 -0.003942441 -1.566482684 0.127035505
0.728715896 -6.203979362 cg18877200 6
29720927 p MOG IFITM4P 2343 IGR island
IGR - island NA -0.002062021 -0.007532335
-0.005470314 0.273755873
-0.011522551 0.01117523 -1.564409716 0.127521202
0.728715896 -6.207076517 cg20642417 6
29577082 p GABBR1/MOG GABBR1 NA Body open
sea Body - open sea NA 0.015136107 0.003613556
-0.011522551 4.188701699
-0.013566738 0.006708891 -1.564312009 0.127544132
0.728715896 -6.20722241 cg02837432 12
72232889 q TPH2 TBC1D15 NA TSS1500 shore
TSS1500 - shore NA 0.011372457 -0.00219428
-0.013566738 -5.182772939
-0.007480588 -0.009246216 -1.564136269 0.127585383
0.728715896 -6.207484803 cg13641185 6

29521143 p GABBR1 UBD -2246 IGR island
IGR - island NA -0.006674764 -0.014155352
-0.007480588 0.47153644
0.020467376 -0.010690774 1.563222007 0.127800163
0.728715896 -6.208849449 cg25017994 6
29627296 p GABBR1/MOG MOG NA Body open
sea Body - open sea NA -0.017726435 0.002740941
0.020467376 -6.467279683
0.021515647 0.0201194 1.563031754 0.127844894
0.728715896 -6.209133338 cg25978138 6
29648161 p GABBR1/MOG ZFP57 7992 IGR open
sea IGR - open sea NA 0.012723397 0.034239044
0.021515647 0.371604903
-0.009744131 0.005069903 -1.553431918 0.130118567
0.732771946 -6.223419382 cg12166917 6
29571429 p GABBR1/MOG GABBR1 NA Body open
sea Body - open sea V$TAXCREB_01 0.008419448 -
0.001324683 -0.009744131 -6.355820428
0.007287476 -0.008551163 1.550929656 0.130716585
0.732771946 -6.227130707 cg23619332 11
27722060 p BDNF BDNF NA Body island
Body - island NA -0.011056233 -0.003768757
0.007287476 2.933655158
-0.00595074 -0.001265919 -1.550349542 0.130855545
0.732771946 -6.22799039 cg11851910 21
34482086 q OLIG2 OLIG1 39636 IGR open
sea IGR - open sea NA 0.000779648 -0.005171092
-0.00595074 -0.150770406
-0.009706018 -0.000592189 -1.550214765 0.130887846
0.732771946 -6.22819008 cg25396488 6
29641118 p GABBR1/MOG ZFP57 NA Body open
sea Body - open sea NA 0.002744254 -0.006961763
-0.009706018 -0.39418958
-0.005808262 0.002124048 -1.548769059 0.131234742
0.732771946 -6.230331138 cg12209876 6
152381560 q ESR1 ESR1 NA Body open
sea Body - open sea NA 0.004120638 -0.001687623
-0.005808262 -2.441680951
0.022054753 0.079310004 1.546330908 0.131821463
0.733055942 -6.23393809 cg00504285 22
20019887 q COMT C22orf25 NA 5'UTR open
sea 5'UTR - open sea NA 0.071728683 0.093783436
0.022054753 0.764833176
0.010935859 -0.00215647 1.534526944 0.134692152
0.737649211 -6.251331396 cg26015683 6
29720519 p MOG IFITM4P 1935 IGR island
IGR - island NA -0.005915672 0.005020187
0.010935859 -1.178376729
-0.020316774 0.005160428 -1.533242099 0.135007654
0.737649211 -6.253217697 cg04369302 6
29581439 p GABBR1/MOG GABBR1 NA Body open
sea Body - open sea NA 0.012144319 -0.008172455
-0.020316774 -1.486006319
0.011269178 0.008435842 1.53234686 0.135227839
0.737649211 -6.254531205 cg09149541 22
19898335 q COMT TXNRD2 NA Body shelf

Body - shelf NA 0.004562062 0.015831239
0.011269178 0.288168325
-0.013502797 0.016163621 -1.531833102 0.13535433
0.737649211 -6.255284698 cg07326586 6
29528119 p GABBR1/MOG UBD NA TSS1500 open
sea TSS1500 - open sea NA 0.020805207 0.00730241 -
0.013502797 2.849087801
0.011642797 0.016335665 1.531035963 0.13555078
0.737649211 -6.256453377 cg02403412 22
19894638 q COMT TXNRD2 NA Body island
Body - island NA 0.012333453 0.023976251
0.011642797 0.514402927
-0.012707993 0.014039765 -1.529691045 0.135882749
0.737649211 -6.258423957 cg11348701 2
171704223 q GAD1 GAD1 NA Body open
sea Body - open sea V$FREAC2_01 0.018408137
0.005700145 -0.012707993 3.229415772
-0.00525286 0.00109694 -1.524770626 0.137102874
0.739126776 -6.265620629 cg15395148 6
29720485 p MOG IFITM4P 1901 IGR island
IGR - island NA 0.00290261 -0.002350249 -
0.00525286 -1.235022209
-0.008778072 -0.023410586 -1.524237809 0.137235527
0.739126776 -6.266398731 cg05238769 21
34399260 q OLIG2 OLIG2 NA Body island
Body - island NA -0.020393124 -0.029171196
-0.008778072 0.699084272
-0.009872572 0.0046452 -1.515586397 0.139403966
0.745291963 -6.278999948 cg14080521 11
27761859 p BDNF BDNF-AS 85417 IGR open
sea IGR - open sea NA 0.008038896 -0.001833676
-0.009872572 -4.384033557
0.010356335 0.007720881 1.51302781 0.140050531
0.745291963 -6.282714759 cg12457376 22
19928061 q COMT COMT NA TSS1500 shore
TSS1500 - shore NA 0.004160891 0.014517225
0.010356335 0.286617489
-0.003249123 -0.000846563 -1.511876062 0.14034237
0.745291963 -6.284385206 cg18943949 22
19842501 q COMT GNB1L NA TSS200 island
TSS200 - island NA 0.000270323 -0.0029788 -
0.003249123 -0.090748852
-0.010342013 -0.017558315 -1.510541038 0.140681261
0.745291963 -6.28632009 cg20997792 6
29595491 p GABBR1/MOG GABBR1 NA Body island
Body - island NA -0.014003248 -0.024345261
-0.010342013 0.575194015
-0.011709267 0.010749881 -1.507890969 0.141355924
0.745291963 -6.29015651 cg10139151 6
29549352 p GABBR1/MOG SNORD32B NA TSS1500 open
sea TSS1500 - open sea NA 0.014774942 0.003065675
-0.011709267 4.819474461
0.00843248 -0.013853649 1.50559486 0.141942578 0.745291963
-6.29347579 cg21053831 18 4455512 p DLGAP1
DLGAP1-AS5 190910 IGR island IGR - island NA
-0.016752314 -0.008319834 0.00843248 2.013539354

0.011163088 0.00370153 1.504613692 0.142193861
0.745291963 -6.294892838 cg08730070 22
19938378 q COMT COMT NA 5'UTR open
sea 5'UTR - open sea NA -0.000135782 0.011027306
0.011163088 -0.012313249
-0.013463277 0.003651408 -1.499851599 0.143418552
0.748841903 -6.301759082 cg10781870 17
28648320 q SLC6A4 TMIGD1 NA Body open
sea Body - open sea NA 0.00827941 -0.005183867 -
0.013463277 -1.597149338
-0.0356051 -0.142053979 -1.495282652 0.14460152 0.748878979
-6.308329064 cg17806418 6 29599319 p
GABBR1/MOG GABBR1 NA Body shore Body - shore
NA -0.129814725 -0.165419826 -0.0356051
0.784759172
0.00978924 -0.007199922 1.4940041 0.144933954 0.748878979
-6.310164455 cg12635048 6 29720957 p MOG
IFITM4P 2373 IGR island IGR - island NA
-0.010564973 -0.000775733 0.00978924 13.61934146
-0.009637522 -0.000321667 -1.493489487 0.145067931
0.748878979 -6.310902809 cg01292475 9
87284571 q NTRK2 NTRK2 NA TSS200 island
TSS200 - island NA 0.002991231 -0.006646291
-0.009637522 -0.450060142
-0.012480924 -0.006448752 -1.485146568 0.147253853
0.752364704 -6.322842133 cg19539318 2
172544773 q SLC25A12 DYNC1I2 NA 5'UTR shore
5'UTR - shore NA -0.002158435 -0.014639359
-0.012480924 0.147440514
-0.005613027 0.002520562 -1.484940329 0.147308222
0.752364704 -6.323136537 cg01779447 6
29720315 p MOG IFITM4P NA TSS1500 shore
TSS1500 - shore NA 0.00445004 -0.001162987 -
0.005613027 -3.826388723
-0.008692353 0.000233701 -1.484009795 0.147553729
0.752364704 -6.324464428 cg13497069 6
29426119 p GABBR1 OR2H1 NA TSS200 open
sea TSS200 - open sea NA 0.003221697 -0.005470656
-0.008692353 -0.588905067
-0.014231954 0.030716239 -1.477659184 0.149237996
0.752364704 -6.333507507 cg02408532 6
29556369 p GABBR1/MOG OR2H2 NA 1stExon open
sea 1stExon - open sea NA 0.035608473 0.021376519
-0.014231954 1.665775112
-0.034656506 0.003955423 -1.477127267 0.149379762
0.752364704 -6.334263407 cg26038589 17
28444874 q SLC6A4 CCDC55 NA Body shore
Body - shore NA 0.015868597 -0.018787909
-0.034656506 -0.844617487
-0.029261407 0.144858456 -1.474526564 0.150074449
0.752364704 -6.337955806 cg07529654 18 3447016
p DLGAP1 TGIF1 NA 5'UTR shore 5'UTR -
shore NA 0.154917065 0.125655658 -0.029261407
1.232869794
0.019457376 -0.000374225 1.468073473 0.151809312
0.752364704 -6.347093173 cg03296810 15
88826454 q NTRK3 NTRK3-AS1 30493 IGR open

sea	IGR - open sea	NA	-0.007062698	0.012394678
	0.019457376	-0.569817015
-0.027654285	0.018938101	-1.466934402	0.152117197
	0.752364704	-6.348702426	cg16073467 6
	29586772	p	GABBR1/MOG GABBR1	NA	Body	open
sea	Body - open sea	NA	0.028444262	0.000789977
	-0.027654285	36.00645247
0.011201596	-0.013438132	1.46641424 0.152257959
	0.752364704	-6.349436934	cg26840770 11
	27723290	p	BDNF	BDNF	NA	TSS1500	shore
	TSS1500 - shore	V$STAT5A_02;V$BRN2_01	-0.017288681
	-0.006087085	0.011201596	2.840223433
-0.01605682	0.007019847	-1.46625911	0.152299959
	0.752364704	-6.349655947	cg04337734 2
	172734703 q	SLC25A12	SLC25A12	NA	Body	open
sea	Body - open sea	NA	0.012539379	-0.003517441
	-0.01605682	-3.564915145
0.011210427	-0.002397572	1.465824746	0.152417609
	0.752364704	-6.350269071	cg21756465 2
	172543834 q	SLC25A12	DYNC1I2	NA	TSS200	shore
	TSS200 - shore	NA	-0.006251157	0.00495927
	0.011210427	-1.260499262
-0.006983219	-0.00364514	-1.462302975	0.153374175
	0.752364704	-6.355234347	cg05929831 6
	29720460	p	MOG	IFITM4P	1876	IGR	island
	IGR - island	NA	-0.001244658	-0.008227877
	-0.006983219	0.151273301
0.021842732	0.042352877	1.459319144	0.154188364
	0.752364704	-6.359433015	cg12315353 6
	29573089	p	GABBR1/MOG GABBR1	NA	Body	open
sea	Body - open sea	NA	0.034844438	0.05668717
	0.021842732	0.614679446
-0.019172727	-0.01843539	-1.459134546	0.154238848
	0.752364704	-6.359692524	cg01176329 6
	29592381	p	GABBR1/MOG GABBR1	NA	Body	shelf
	Body - shelf	NA	-0.011844765	-0.031017491
	-0.019172727	0.38187372
-0.016219516	-0.011426523	-1.458384888	0.154443998
	0.752364704	-6.360746102	cg22402007 9
	87282823	q	NTRK2	NTRK2	NA	TSS1500	shore
	TSS1500 - shore	NA	-0.005851065	-0.022070581
	-0.016219516	0.265106961
-0.011914299	-0.009220246	-1.458024311	0.15454275
	0.752364704	-6.361252692	cg19372359 6
	29575128	p	GABBR1/MOG GABBR1	NA	Body	open
sea	Body - open sea	NA	-0.005124706	-0.017039004
	-0.011914299	0.300763209
-0.004401713	-0.003287188	-1.454483758	0.15551508
	0.75441358 -6.366221141	cg02954262 18	3451750	p
	DLGAP1	TGIF1	NA	5'UTR	island	5'UTR - island
	NA	-0.001774099	-0.006175812	-0.004401713
	0.287265731
-0.028200604	-0.040655551	-1.451077212	0.156455192
	0.754882407	-6.370991547	cg07420274 2
	171676306 q	GAD1	GAD1	NA	Body	shore
	Body - shore	NA	-0.030961593	-0.059162197
	-0.028200604	0.523334067

0.01476318 -0.012170704 1.450137376 0.156715354
0.754882407 -6.372305935 cg25565730 6
152085565 q ESR1 ESR1 NA 5'UTR open
sea 5'UTR - open sea NA -0.017245547 -0.002482367
0.01476318 6.947219167
-0.020399756 0.151913353 -1.439258517 0.159751851
0.760099502 -6.387465949 cg09083279 6
29454873 p GABBR1 MAS1L NA 1stExon open
sea 1stExon - open sea NA 0.158925769 0.138526013
-0.020399756 1.147262994
0.004119752 -0.00228048 1.439195647 0.159769533
0.760099502 -6.387553269 cg09523380 6
29720748 p MOG IFITM4P 2164 IGR island
IGR - island NA -0.003696645 0.000423107
0.004119752 -8.736902105
-0.009761243 0.000852291 -1.43856369 0.159947362
0.760099502 -6.388430805 cg27657867 11
27818018 p BDNF BDNF-AS 141576 IGR open
sea IGR - open sea NA 0.004207718 -0.005553525
-0.009761243 -0.75766619
-0.00870711 0.006616604 -1.429692643 0.162460197
0.760099502 -6.400713399 cg15209921 6
29430506 p GABBR1 OR2H1 NA 3'UTR open
sea 3'UTR - open sea NA 0.009609673 0.000902563
-0.00870711 10.64709608
-0.046023591 0.01923984 -1.429165058 0.16261062 0.760099502
-6.401441774 cg07134666 6 29648400 p
GABBR1/MOG ZFP57 8231 IGR open sea IGR - open sea
NA 0.03506045 -0.010963141 -0.046023591 -
3.198029572
-0.009406319 0.021490412 -1.427828864 0.162992081
0.760099502 -6.403285439 cg10441070 6
152126250 q ESR1 ESR1 NA 5'UTR shelf
5'UTR - shelf NA 0.024723834 0.015317515
-0.009406319 1.614089077
-0.009710011 -0.002074278 -1.425753546 0.16358595
0.760099502 -6.406145928 cg26089753 6
152127821 q ESR1 ESR1 NA TSS1500 shore
TSS1500 - shore NA 0.001263539 -0.008446473
-0.009710011 -0.149593642
0.009232942 -0.011457463 1.425365447 0.163697197
0.760099502 -6.406680452 cg27316393 6
152128675 q ESR1 ESR1 NA 5'UTR shore
5'UTR - shore NA -0.014631287 -0.005398345
0.009232942 2.710328174
0.013927914 0.01223071 1.422047818 0.16465062 0.760099502
-6.411244559 cg18584905 17 28563300 q SLC6A4
SLC6A4 NA TSS1500 shore TSS1500 - shore NA
0.007442989 0.021370903 0.013927914
0.348276781
-0.0126934 0.00265343 -1.42134671 0.164852664 0.760099502
-6.412207884 cg08921491 15 88577641 q NTRK3
NTRK3 NA Body open sea Body - open sea
V$POU6F1_01 0.007016786 -0.005676614 -
0.0126934 -1.23608651
-0.012547464 0.003392731 -1.420502575 0.165096185
0.760099502 -6.413367172 cg03160788 6

29572219 p GABBR1/MOG GABBR1 NA Body open
sea Body - open sea NA 0.007705922 -0.004841542
-0.012547464 -1.591625438
-0.019562203 0.050669127 -1.420325938 0.165147178
0.760099502 -6.413609679 cg14140375 6
29692582 p GABBR1/MOG HLA-F NA Body shore
Body - shore NA 0.057393635 0.037831431
-0.019562203 1.51708864
-0.015693695 -0.014100287 -1.418394734 0.165705505
0.760099502 -6.416259314 cg04892672 6
29695164 p GABBR1/MOG LOC285830 NA Body shelf
Body - shelf NA -0.008705579 -0.024399274
-0.015693695 0.356796645
-0.02050956 0.044035449 -1.418016488 0.165815033
0.760099502 -6.416777899 cg16891968 6
29698284 p MOG LOC285830 NA Body open
sea Body - open sea NA 0.05108561 0.03057605 -0.02050956
1.670772039
-0.010666618 0.00403242 -1.415300289 0.166603234
0.760099502 -6.420498301 cg01306985 6
29720340 p MOG IFITM4P NA TSS1500 shore
TSS1500 - shore NA 0.00769907 -0.002967548 -
0.010666618 -2.594421516
0.025018799 -0.1162753 1.410791182 0.16791822 0.760099502
-6.426660567 cg03147503 6 29633969 p
GABBR1/MOG MOG NA 3'UTR open sea 3'UTR - open sea
NA -0.124875512 -0.099856714 0.025018799
1.250546989
0.011038254 -0.005073873 1.407009969 0.16902721
0.760099502 -6.431814689 cg05119316 6
29716135 p MOG LOC285830 NA Body shore
Body - shore NA -0.008868273 0.002169982
0.011038254 -4.086796406
-0.013571646 -0.087729328 -1.405978253 0.169330798
0.760099502 -6.433218886 cg08415141 6
29598310 p GABBR1/MOG GABBR1 NA Body shore
Body - shore NA -0.083064075 -0.096635721
-0.013571646 0.859558704
-0.020696288 -0.003639053 -1.405969016 0.169333518
0.760099502 -6.433231454 cg18977283 6
29579492 p GABBR1/MOG GABBR1 NA Body open
sea Body - open sea NA 0.003475296 -0.017220992
-0.020696288 -0.201805778
-0.004952657 -0.006087017 -1.404419105 0.169790399
0.760099502 -6.435339207 cg10491628 6
29521220 p GABBR1 UBD -2169 IGR island
IGR - island NA -0.004384541 -0.009337198
-0.004952657 0.469577831
-0.00659802 0.003139238 -1.403641819 0.170019891
0.760099502 -6.436395478 cg03467156 6
29425910 p GABBR1 OR2H1 NA TSS1500 open
sea TSS1500 - open sea NA 0.005407307 -0.001190713
-0.00659802 -4.541235475
-0.011834389 0.006693376 -1.402430294 0.170378075
0.760099502 -6.438040817 cg19535685 22
19845442 q COMT GNB1L 11781 IGR shelf

IGR - shelf NA 0.010761447 -0.001072942
-0.011834389 -10.02985029
-0.011478419 -0.042285718 -1.401755324 0.170577885
0.760099502 -6.43895693 cg08862148 6
29595315 p GABBR1/MOG GABBR1 NA Body island
Body - island NA -0.038340012 -0.049818431
-0.011478419 0.769594927
-0.014773864 0.003133287 -1.399286086 0.171310416
0.760885222 -6.442305017 cg21552290 6
29578423 p GABBR1/MOG GABBR1 NA Body open
sea Body - open sea NA 0.008211803 -0.006562061
-0.014773864 -1.251406124
-0.017518383 -0.008255235 -1.396069331 0.172268403
0.762663996 -6.446658842 cg00521863 2
171783942 q GAD1 GORASP2 -1006 IGR shore
IGR - shore NA -0.00223329 -0.019751673
-0.017518383 0.113068413
-0.025426902 0.010307796 -1.386949638 0.175007171
0.772289711 -6.458953948 cg22494932 6
29648379 p GABBR1/MOG ZFP57 8210 IGR open
sea IGR - open sea NA 0.019048294 -0.006378608
-0.025426902 -2.986277424
0.011390555 -0.007791424 1.384754504 0.175671455
0.772728459 -6.461902747 cg08362738 11
27722636 p BDNF BDNF NA TSS200 island
TSS200 - island NA -0.011706927 -0.000316373
0.011390555 37.00359285
-0.012953258 -0.006352358 -1.38215308 0.176461237
0.773714656 -6.465391966 cg18971999 6
29712014 p MOG LOC285830 NA Body open
sea Body - open sea NA -0.001899675 -0.014852933
-0.012953258 0.127898984
-0.013163755 0.021537765 -1.376744572 0.178112106
0.777025898 -6.472627608 cg21185936 6
29716247 p MOG LOC285830 NA Body shore
Body - shore NA 0.026062806 0.012899051
-0.013163755 2.020521171
-0.003281278 0.001723735 -1.375960478 0.178352436
0.777025898 -6.473674499 cg17137980 22
19842443 q COMT GNB1L NA 1stExon island
1stExon - island NA 0.002851674 -0.000429603
-0.003281278 -6.637922592
-0.010431923 0.006476509 -1.374057569 0.178936739
0.777096695 -6.476212983 cg02139827 21
34404457 q OLIG2 OLIG2 6241 IGR shore
IGR - shore NA 0.010062482 -0.000369441
-0.010431923 -27.23706243
-0.010500462 0.000221256 -1.368611778 0.180617151
0.781318223 -6.483460421 cg02404255 6
152419175 q ESR1 ESR1 NA Body open
sea Body - open sea NA 0.003830789 -0.006669672
-0.010500462 -0.574359429
0.012560434 -0.063154809 1.367211932 0.181051079
0.781318223 -6.485319247 cg24302412 6
29595196 p GABBR1/MOG GABBR1 NA Body shore
Body - shore NA -0.067472458 -0.054912025
0.012560434 1.228737397

0.009556995 0.020164074 1.362914549 0.182388259
0.784577021 -6.491015076 cg11423684 6
29691993 p GABBR1/MOG HLA-F NA Body island
Body - island NA 0.016878856 0.026435852
0.009556995 0.638483548
-0.006345133 -0.00472038 -1.361106094 0.182953267
0.784577021 -6.493407264 cg20340508 21
34442377 q OLIG2 OLIG1 NA TSS200 island
TSS200 - island NA -0.002539241 -0.008884373
-0.006345133 0.285809754
-0.002828258 7.57E-05 -1.353940043 0.185205493
0.791753481 -6.502858543 cg16956031 18 3451545
p DLGAP1 TGIF1 NA 5'UTR island 5'UTR -
island NA 0.001047933 -0.001780326 -0.002828258
-0.588618645
-0.008193343 0.000198935 -1.350652288 0.186245965
0.793721121 -6.507179865 cg01373089 6
29589631 p GABBR1/MOG GABBR1 NA Body open
sea Body - open sea NA 0.003015396 -0.005177947
-0.008193343 -0.582353693
0.005617816 -0.001700949 1.344265956 0.188279961
0.798771081 -6.515547065 cg26375461 6
29716541 p MOG LOC285830 NA Body island
Body - island NA -0.003632073 0.001985743
0.005617816 -1.829075403
-0.015632464 -0.034831075 -1.343123139 0.188645743
0.798771081 -6.517040618 cg27639046 2
171608303 q GAD1 SP5 36446 IGR open
sea IGR - open sea NA -0.029457415 -0.045089879
-0.015632464 0.653304379
-0.034466606 0.317059693 -1.341448849 0.189182624
0.798771081 -6.519226706 cg20103692 6
29454672 p GABBR1 MAS1L NA 1stExon open
sea 1stExon - open sea NA 0.328907589 0.294440983
-0.034466606 1.117057774
-0.019930156 -0.029773482 -1.338209067 0.190224848
0.79879968 -6.523449907 cg03536022 6 29706879 p
MOG LOC285830 NA Body open sea Body - open sea
NA -0.022922491 -0.042852647 -0.019930156
0.534914234
-0.005349443 -0.009558722 -1.335053652 0.191244182
0.79879968 -6.527554355 cg23947039 11 27722037 p
BDNF BDNF NA Body island Body - island
NA -0.007719851 -0.013069294 -0.005349443
0.59068613
-0.017725617 -0.039286615 -1.334197934 0.19152134
0.79879968 -6.528665948 cg02014853 6 29595335 p
GABBR1/MOG GABBR1 NA Body island Body - island
NA -0.033193434 -0.050919051 -0.017725617
0.651886347
-0.014966142 0.039757665 -1.332589216 0.192043223
0.79879968 -6.530753974 cg00758854 6 29576422 p
GABBR1/MOG GABBR1 NA Body open sea Body - open sea
NA 0.044902276 0.029936134 -0.014966142
1.499935711
0.007728423 -0.014784942 1.332386736 0.192108987
0.79879968 -6.531016623 cg01642653 11 27743476 p

BDNF BDNF NA TSS1500 island TSS1500 - island
V$BRACH_01 -0.017441587 -0.009713164 0.007728423
1.795664806
0.021831739 0.140753061 1.328240554 0.193459452
0.800922199 -6.53638701 cg22298860 6
29690822 p GABBR1/MOG HLA-F NA TSS1500 shore
TSS1500 - shore NA 0.1332484 0.15508014 0.021831739
0.859222854
0.009899472 -0.0048298 1.327227922 0.193790386
0.800922199 -6.537696355 cg06350404 18 3452443
p DLGAP1 TGIF1 NA 5'UTR shore 5'UTR -
shore NA -0.008232744 0.001666728 0.009899472
-4.939463819
-0.00771937 -0.000703789 -1.324573803 0.194659833
0.801259774 -6.541123918 cg16527407 6
29582672 p GABBR1/MOG GABBR1 NA Body open
sea Body - open sea NA 0.001949744 -0.005769626
-0.00771937 -0.337932523
-0.024563081 -5.73E-06 -1.322593504 0.195310497
0.801259774 -6.543677297 cg12644888 6
29648360 p GABBR1/MOG ZFP57 8191 IGR open
sea IGR - open sea NA 0.008437826 -0.016125254
-0.024563081 -0.523267798
0.003740246 -0.000354454 1.320137957 0.19611963
0.801259774 -6.546838701 cg08319422 6
29617817 p GABBR1/MOG MOG -6941 IGR island
IGR - island NA -0.001640164 0.002100082
0.003740246 -0.78099996
0.011032273 0.006020613 1.309554931 0.199636317
0.801259774 -6.560403532 cg08884395 6
152127887 q ESR1 ESR1 NA TSS1500 shore
TSS1500 - shore NA 0.002228269 0.013260542
0.011032273 0.168037547
0.043413534 0.222020258 1.307308869 0.200388841
0.801259774 -6.563269817 cg04186657 6
29690893 p GABBR1/MOG HLA-F NA TSS1500 shore
TSS1500 - shore NA 0.207096856 0.25051039
0.043413534 0.826699666
-0.019086757 0.011126018 -1.307264449 0.200403745
0.801259774 -6.563326459 ch.6.2958553R 6
152519966 q ESR1 SYNE1 NA Body open
sea Body - open sea NA 0.01768709 -0.001399667 -
0.019086757 -12.63664349
0.003694589 -0.001407217 1.306429017 0.200684218
0.801259774 -6.564391425 cg08967211 6
29596174 p GABBR1/MOG GABBR1 NA Body shore
Body - shore NA -0.002677231 0.001017357
0.003694589 -2.631554436
-0.009565604 -0.035699225 -1.306041378 0.200814459
0.801259774 -6.56488536 cg13612847 2
171672205 q GAD1 GAD1 NA TSS1500 shore
TSS1500 - shore NA -0.032411048 -0.041976652
-0.009565604 0.772120852
-0.00952442 0.000283893 -1.302719623 0.201933167
0.801259774 -6.569112579 cg10591943 17
28447544 q SLC6A4 CCDC55 NA Body shelf

Body - shelf NA 0.003557913 -0.005966507
-0.00952442 -0.596314169
-0.007132617 -0.015637767 -1.302458231 0.202021401
0.801259774 -6.569444813 cg03565659 12
72332624 q TPH2 TPH2 NA TSS200 open
sea TSS200 - open sea NA -0.01318593 -0.020318547
-0.007132617 0.648960298
0.006227312 -0.011397911 1.302244227 0.20209366
0.801259774 -6.569716771 cg02285263 6
152129749 q ESR1 ESR1 NA Body island
Body - island NA -0.01353855 -0.007311237
0.006227312 1.851745358
-0.00709831 0.008064111 -1.300029256 0.202842717
0.801259774 -6.572529208 cg00041368 18 3879131
p DLGAP1 DLGAP1 NA 1stExon shore 1stExon
- shore NA 0.010504155 0.003405845 -0.00709831
3.084155467
-0.010881674 -0.014770168 -1.296621013 0.203999446
0.801259774 -6.576848376 cg21645752 6
29598695 p GABBR1/MOG GABBR1 NA Body shore
Body - shore NA -0.011029593 -0.021911267
-0.010881674 0.50337539
-0.004592488 0.000789752 -1.294789269 0.204623199
0.801259774 -6.579165477 cg07306299 6
29617902 p GABBR1/MOG MOG -6856 IGR island
IGR - island NA 0.00236842 -0.002224068 -
0.004592488 -1.064904581
0.009992703 -0.032253514 1.292453869 0.205420563
0.801259774 -6.582115407 cg13537510 6
29623721 p GABBR1/MOG MOG NA TSS1500 open
sea TSS1500 - open sea NA -0.035688506 -0.025695803
0.009992703 1.388884622
-0.01437189 0.013123441 -1.291377069 0.205789005
0.801259774 -6.583473938 cg27318000 18 3845667
p DLGAP1 DLGAP1 NA TSS1500 open sea TSS1500
- open sea NA 0.018063778 0.003691888 -0.01437189
4.892829622
-0.011576382 0.014091785 -1.289112525 0.20656549
0.801259774 -6.586327641 cg22115805 15
88804912 q NTRK3 NTRK3-AS1 8951 IGR shelf
IGR - shelf NA 0.018071166 0.006494784
-0.011576382 2.78241207
-0.007249022 -0.015686047 -1.286709448 0.20739191
0.801259774 -6.589350982 cg11813455 6
152128515 q ESR1 ESR1 NA TSS1500 shore
TSS1500 - shore NA -0.013194196 -0.020443218
-0.007249022 0.64540702
0.007630985 0.001611423 1.286114256 0.207596985
0.801259774 -6.590099015 cg04960880 6
29577006 p GABBR1/MOG GABBR1 NA Body open
sea Body - open sea NA -0.001011728 0.006619256
0.007630985 -0.152846236
0.004585288 -0.003488074 1.285465867 0.207820564
0.801259774 -6.590913549 cg14888846 17
28443962 q SLC6A4 MIR423 NA TSS200 island
TSS200 - island NA -0.005064267 -0.000478979
0.004585288 10.5730372

-0.014021048 0.016683808 -1.282203365 0.208948324
0.801259774 -6.595006426 cg09298147 6
29550134 p GABBR1/MOG SNORD32B 105 IGR open
sea IGR - open sea NA 0.021503544 0.007482496
-0.014021048 2.873846474
-0.003867327 0.002473936 -1.277290587 0.210655293
0.801259774 -6.601151901 cg09029526 6
29720653 p MOG IFITM4P 2069 IGR island
IGR - island NA 0.00380333 -6.40E-05 -0.003867327
-59.4299543
-0.00623297 0.003407565 -1.275165293 0.211397001
0.801259774 -6.603803864 cg25758242 6
29427011 p GABBR1 OR2H1 NA 5'UTR open
sea 5'UTR - open sea NA 0.005550149 -0.000682822
-0.00623297 -8.128255215
-0.013598898 0.002338446 -1.275115956 0.211414243
0.801259774 -6.603865379 cg07189962 6
152126092 q ESR1 ESR1 NA 5'UTR shelf
5'UTR - shelf NA 0.007013068 -0.00658583
-0.013598898 -1.064872208
-0.010182276 -0.026142814 -1.273366894 0.21202617
0.801259774 -6.606044809 cg09793121 21
34398263 q OLIG2 OLIG2 NA 1stExon island
1stExon - island NA -0.022642657 -0.032824933
-0.010182276 0.689800564
0.040056034 0.131001677 1.272802962 0.212223752
0.801259774 -6.606746923 cg11201654 6
29690766 p GABBR1/MOG HLA-F NA TSS1500 shore
TSS1500 - shore NA 0.117232416 0.15728845
0.040056034 0.745333913
-0.008019572 -0.002159461 -1.272701964 0.212259153
0.801259774 -6.60687264 cg02945359 6
29694676 p GABBR1/MOG LOC285830 NA Body shelf
Body - shelf NA 0.000597267 -0.007422305
-0.008019572 -0.080469167
-0.027432455 -0.057144777 -1.266355738 0.214492549
0.801259774 -6.614753953 cg15584790 6
29692475 p GABBR1/MOG HLA-F NA Body shore
Body - shore NA -0.047714871 -0.075147325
-0.027432455 0.634951017
-0.006940207 -0.001962837 -1.26128565 0.216289554
0.801259774 -6.621024833 cg03205258 22
19929274 q COMT TXNRD2 NA 1stExon island
1stExon - island V$ELK1_02 0.000422859 -0.006517347
-0.006940207 -0.064882104
-0.010037289 -0.005032764 -1.257877179 0.217503992
0.801259774 -6.625227758 cg26126367 17
28559497 q SLC6A4 SLC6A4 NA 5'UTR shelf
5'UTR - shelf NA -0.001582446 -0.011619734
-0.010037289 0.136186044
-0.013949256 -0.008880829 -1.25493356 0.218556929
0.801259774 -6.628849191 cg27642588 6
29709602 p MOG LOC285830 NA Body open
sea Body - open sea NA -0.004085772 -0.018035028
-0.013949256 0.226546497
-0.016181332 0.046702572 -1.25395927 0.218906278
0.801259774 -6.630046134 cg20927242 6

29692011 p GABBR1/MOG HLA-F NA Body island
Body - island NA 0.052264905 0.036083573
-0.016181332 1.448440394
-0.007423689 -0.00689643 -1.253619838 0.219028086
0.801259774 -6.630462938 cg24650785 11
27741916 p BDNF BDNF NA Body shore
Body - shore V$CEBP_C;V$ZIC2_01 -0.004344537 -
0.011768226 -0.007423689 0.369175175
0.029481635 -0.177329911 1.2535502 0.219053082
0.801259774 -6.630548436 cg15628633 6
29706377 p MOG LOC285830 NA Body open
sea Body - open sea NA -0.187464223 -0.157982588
0.029481635 1.186613191
-0.012365169 0.033846405 -1.252011172 0.219606061
0.801259774 -6.632436899 cg14093720 18 3712400
p DLGAP1 DLGAP1 NA Body open sea Body -
open sea NA 0.038096932 0.025731762 -0.012365169
1.480541105
-0.027780326 0.030001928 -1.24985164 0.220383758
0.801259774 -6.635083205 cg20215212 17
28618041 q SLC6A4 BLMH NA Body shore
Body - shore NA 0.039551415 0.011771089
-0.027780326 3.36004715
-0.023833392 -0.042130925 -1.249335642 0.220569886
0.801259774 -6.635714899 cg00495303 18 3771110
p DLGAP1 DLGAP1 NA Body shore Body -
shore NA -0.033938197 -0.057771588 -0.023833392
0.587454796
-0.007326019 0.000773509 -1.24859463 0.220837387
0.801259774 -6.636621645 cg21034903 2
172544336 q SLC25A12 DYNC1I2 NA 5'UTR island
5'UTR - island V$STAT3_01;V$STAT1_01 0.003291828
-0.004034191 -0.007326019 -0.815982313
0.006610352 0.006999999 1.248050105 0.221034113
0.801259774 -6.637287648 cg05087623 6
29527870 p GABBR1/MOG UBD NA TSS200 open
sea TSS200 - open sea NA 0.00472769 0.011338042
0.006610352 0.416975888
-0.009639655 0.002397187 -1.24655389 0.221575341
0.801259774 -6.639116294 cg13228862 18 3729405
p DLGAP1 DLGAP1 NA Body island Body -
island NA 0.005710819 -0.003928836 -0.009639655
-1.453564968
-0.01629989 0.008570337 -1.244000377 0.222501324
0.801259774 -6.642232553 cg09926649 22
19938096 q COMT COMT NA 5'UTR open
sea 5'UTR - open sea NA 0.014173424 -0.002126466
-0.01629989 -6.665247629
-0.006139263 0.005981443 -1.23816213 0.224629346
0.801259774 -6.649335639 cg20067272 6
29571693 p GABBR1/MOG GABBR1 NA Body open
sea Body - open sea NA 0.008091815 0.001952552
-0.006139263 4.144224384
-0.007399496 -0.000386298 -1.237872729 0.224735227
0.801259774 -6.649686949 cg17071948 6
29590143 p GABBR1/MOG GABBR1 NA Body open

sea	Body - open sea	NA	0.002157279	-0.005242218
-0.007399496	-0.411520224
0.028056659	-0.19311758	1.23742622 0.224898661
0.801259774	-6.650228828	cg20704602 6
29635371	p	GABBR1/MOG MOG	NA	3'UTR	open
sea	3'UTR - open sea	NA	-0.202762057	-0.174705398
0.028056659	1.160594118
0.025946872	0.048100916	1.236511428	0.225233775
0.801259774	-6.651338457	cg23606396 6
29717917	p	MOG	LOC285830 NA	TSS1500	shore
TSS1500 - shore	NA	0.039181679	0.065128551
0.025946872	0.601605261
-0.005837787	0.003572289	-1.235903261	0.225456771
0.801259774	-6.65207574	cg12248981 6
29570707	p	GABBR1/MOG GABBR1	NA	3'UTR	open
sea	3'UTR - open sea	NA	0.005579028	-0.000258759
-0.005837787	-21.56074768
-0.034942698	-0.064295335	-1.234851512	0.225842804
0.801259774	-6.653350005	cg17977304 6
29623992	p	GABBR1/MOG MOG	NA	TSS1500	open
sea	TSS1500 - open sea	NA	-0.052283782	-0.087226481
-0.034942698	0.599402634
-0.008555025	0.009118564	-1.23402454	0.226146682
0.801259774	-6.654351243	cg13224710 18	3411743
p	DLGAP1	TGIF1	NA	TSS1500	open sea	TSS1500
- open sea NA	0.012059354	0.003504329	-0.008555025
3.441273378
-0.014680288	0.001297418	-1.233971899	0.226166035
0.801259774	-6.654414957	cg00667298 6
29576329	p	GABBR1/MOG GABBR1	NA	Body	open
sea	Body - open sea	NA	0.006343767	-0.008336521
-0.014680288	-0.760960913
-0.030870567	-0.007703003	-1.233816587	0.226223144
0.801259774	-6.654602922	cg00588198 6
29648452	p	GABBR1/MOG ZFP57	8283	IGR	open
sea	IGR - open sea	NA	0.002908754	-0.027961813
-0.030870567	-0.104025962
0.007710644	-0.013365782	1.232965591	0.226536247
0.801259774	-6.655632453	cg13612689 6
152128634 q	ESR1	ESR1	NA	5'UTR	shore
5'UTR - shore	NA	-0.016016315	-0.008305672
0.007710644	1.928358857
0.010200452	-0.008502666	1.231045551	0.227243869
0.801259774	-6.657952935	cg12074493 17
28564117	q	SLC6A4	SLC6A4	NA	TSS1500	shore
TSS1500 - shore	NA	-0.012009072	-0.001808619
0.010200452	6.63991092
0.013921046	-0.002628546	1.230982658	0.227267075
0.801259774	-6.658028889	cg20386586 18	3449692
p	DLGAP1	TGIF1	NA	5'UTR	island	5'UTR -
island	NA	-0.007413906	0.00650714 0.013921046	-
1.139349354
0.011452868	-0.037972494	1.22962783 0.227767421
0.801259774	-6.659664223	cg19382714 6
29624846	p	GABBR1/MOG MOG	NA	1stExon	open
sea	1stExon - open sea	NA	-0.041909418	-0.030456549
0.011452868	1.376039586

-0.01714681 -0.002742318 -1.227910624 0.228402775
0.801259774 -6.661734609 cg25927551 6
29707293 p MOG LOC285830 NA Body open
sea Body - open sea NA 0.003151898 -0.013994913
-0.01714681 -0.225217412
-0.021950478 0.077426997 -1.22737422 0.228601511
0.801259774 -6.662380796 cg04831505 12
72233240 q TPH2 TBC1D15 NA TSS1500 shore
TSS1500 - shore NA 0.084972474 0.063021997
-0.021950478 1.348298669
-0.028722616 0.01546786 -1.227200845 0.228665774
0.801259774 -6.6625896 cg02979010 12 72319077 q
TPH2 TBC1D15 NA 3'UTR open sea 3'UTR - open sea
NA 0.025341259 -0.003381356 -0.028722616
-7.494407249
-0.039272874 -0.024239387 -1.225283437 0.229377373
0.801259774 -6.664897036 cg11617938 6
29692281 p GABBR1/MOG HLA-F NA Body shore
Body - shore NA -0.010739336 -0.050012211
-0.039272874 0.214734286
-0.020477305 -0.094120112 -1.222309563 0.230484314
0.801259774 -6.668469339 cg25642476 6
29595011 p GABBR1/MOG GABBR1 NA Body shore
Body - shore NA -0.087081038 -0.107558343
-0.020477305 0.809616765
-0.015600163 0.121742107 -1.221417907 0.230816982
0.801259774 -6.669538881 cg07059469 6
152421432 q ESR1 ESR1 NA 3'UTR open
sea 3'UTR - open sea NA 0.127104663 0.1115045 -
0.015600163 1.139906132
-0.004881853 -0.007694982 -1.219742177 0.231443147
0.801259774 -6.671546998 cg11281641 2
171674855 q GAD1 GAD1 NA 5'UTR island
5'UTR - island NA -0.006016845 -0.010898698
-0.004881853 0.552070042
-0.012083685 0.005255141 -1.217936726 0.232119196
0.801259774 -6.673707754 cg13730341 6
29589573 p GABBR1/MOG GABBR1 NA Body open
sea Body - open sea V$HSF2_01 0.009408907 -0.002674778
-0.012083685 -3.517640796
-0.009251612 0.003983408 -1.217440035 0.23230544
0.801259774 -6.674301681 cg19530293 12
72426134 q TPH2 TPH2 NA 3'UTR open
sea 3'UTR - open sea NA 0.007163649 -0.002087962
-0.009251612 -3.430928623
-0.02630089 -0.121144292 -1.216580309 0.232628071
0.801259774 -6.67532919 cg22679406 6
29599331 p GABBR1/MOG GABBR1 NA Body shore
Body - shore NA -0.112103361 -0.138404252
-0.02630089 0.809970502
-0.012948225 0.112711011 -1.216226423 0.232760971
0.801259774 -6.675751947 cg26129669 6
29593913 p GABBR1/MOG GABBR1 NA Body shore
Body - shore NA 0.117161964 0.104213739
-0.012948225 1.124246815
-0.009131194 0.001690623 -1.215518492 0.233027001
0.801259774 -6.676597316 cg03732055 6

152201038 q ESR1 ESR1 NA Body open
sea Body - open sea NA 0.004829471 -0.004301723
-0.009131194 -1.12268284
0.01260013 -0.022080952 1.215273266 0.233119206
0.801259774 -6.676890046 cg26131286 6
29521783 p GABBR1 UBD -1606 IGR island
IGR - island NA -0.026412247 -0.013812117
0.01260013 1.912251886
-0.010172655 0.004180297 -1.210040759 0.235093086
0.801259774 -6.683123322 cg05863862 6
29649807 p GABBR1/MOG ZFP57 9638 IGR open
sea IGR - open sea NA 0.007677147 -0.002495508
-0.010172655 -3.076386582
0.010955732 -0.004972422 1.207579497 0.236025833
0.801259774 -6.686046838 cg27527874 6
29571074 p GABBR1/MOG GABBR1 NA 3'UTR open
sea 3'UTR - open sea NA -0.008738455 0.002217277
0.010955732 -3.941075713
-0.011265674 0.000194357 -1.206854459 0.236301123
0.801259774 -6.68690701 cg20720118 6
29523976 p GABBR1 UBD NA Body shelf
Body - shelf NA 0.004066932 -0.007198742
-0.011265674 -0.56495045
-0.008583878 -0.000512302 -1.204787874 0.23708709
0.801259774 -6.689356183 cg20795635 6
29578496 p GABBR1/MOG GABBR1 NA Body open
sea Body - open sea NA 0.002438406 -0.006145472
-0.008583878 -0.39678084
0.008486277 -0.012663673 1.204384921 0.237240567
0.801259774 -6.689833288 cg15753746 2
171679591 q GAD1 GAD1 NA Body island
Body - island NA -0.015580831 -0.007094554
0.008486277 2.196167792
0.012698186 -0.014028329 1.204248542 0.237292528
0.801259774 -6.689994731 cg16994534 6
29620138 p GABBR1/MOG MOG -4620 IGR shelf
IGR - shelf NA -0.018393331 -0.005695145
0.012698186 3.229651166
-0.009529616 -0.011103872 -1.201746534 0.238247295
0.801259774 -6.692953594 cg01225698 11
27742355 p BDNF BDNF NA Body shore
Body - shore NA -0.007828067 -0.017357683
-0.009529616 0.450985711
-0.022281288 -0.004804788 -1.201561435 0.238318041
0.801259774 -6.693172268 cg20704819 6
29585653 p GABBR1/MOG GABBR1 NA Body open
sea Body - open sea NA 0.002854405 -0.019426883
-0.022281288 -0.146930674
-0.016433541 -0.007777307 -1.199621626 0.239060388
0.801259774 -6.695462086 cg07379508 2
171630114 q GAD1 GAD1 -43086 IGR shelf
IGR - shelf NA -0.002128277 -0.018561818
-0.016433541 0.114658856
0.005149793 0.009860397 1.198913014 0.239331993
0.801259774 -6.696297713 cg19070841 6
29691643 p GABBR1/MOG HLA-F NA Body island

Body - island NA 0.008090156 0.013239949
0.005149793 0.611041329
-0.024451688 -0.004217494 -1.198324001 0.239557929
0.801259774 -6.69699196 cg14005211 2
171676925 q GAD1 GAD1 NA Body island
Body - island NA 0.004187774 -0.020263914
-0.024451688 -0.206661645
-0.014855362 0.013243365 -1.196262031 0.240350111
0.801948665 -6.699419867 cg13680362 6
29426320 p GABBR1 OR2H1 NA 1stExon open
sea 1stExon - open sea NA 0.018349896 0.003494534
-0.014855362 5.251028334
-0.010395631 0.003302298 -1.19458431 0.240996095
0.802147587 -6.701392511 cg21905167 22
19949585 q COMT COMT NA 5'UTR open
sea 5'UTR - open sea NA 0.006875796 -0.003519835
-0.010395631 -1.953442468
0.008927644 0.004753148 1.192198315 0.241916994
0.803258369 -6.704193575 cg05542661 6
29717068 p MOG LOC285830 NA TSS1500 island
TSS1500 - island NA 0.00168427 0.010611914
0.008927644 0.158715013
-0.002720102 0.00048291 -1.188039048 0.2435285 0.803750816
-6.709064135 cg14583606 9 4490315 p SLC1A1
SLC1A1 NA TSS200 island TSS200 - island NA
0.001417945 -0.001302156 -0.002720102 -
1.088920908
0.007588047 0.000440264 1.186527478 0.24411611
0.803750816 -6.710830344 cg13555101 9 4490751
p SLC1A1 SLC1A1 NA 1stExon island 1stExon
- island NA -0.002168127 0.00541992 0.007588047 -
0.400029433
0.013952134 -0.008522411 1.186475715 0.244136251
0.803750816 -6.710890791 cg18374181 21
34401798 q OLIG2 OLIG2 3582 IGR shore
IGR - shore NA -0.013318457 0.000633677
0.013952134 -21.01774236
-0.003082198 -0.000667256 -1.185758476 0.244415453
0.803750816 -6.711728103 cg24710870 6
29720670 p MOG IFITM4P 2086 IGR island
IGR - island NA 0.00039225 -0.002689948 -
0.003082198 -0.145820477
-0.013059142 -0.053889957 -1.181189113 0.246199696
0.804932682 -6.717051528 cg27293992 2
171671648 q GAD1 GAD1 -1552 IGR island
IGR - island NA -0.049400877 -0.062460019
-0.013059142 0.790919974
-0.014511358 -0.006189318 -1.17914084 0.247002601
0.804932682 -6.719431705 cg17723549 17
28443042 q SLC6A4 CCDC55 NA TSS1500 shore
TSS1500 - shore NA -0.001201039 -0.015712396
-0.014511358 0.076438922
-0.006799772 -0.005853464 -1.177404519 0.247684726
0.804932682 -6.721446415 cg21956337 15
88799707 q NTRK3 NTRK3 NA TSS200 island
TSS200 - island NA -0.003516043 -0.010315815
-0.006799772 0.34084007

-0.005149859 0.001468772 -1.176648514 0.247982159
0.804932682 -6.722322782 cg04711050 9 4490757
p SLC1A1 SLC1A1 NA 1stExon island 1stExon
- island NA 0.003239036 -0.001910823 -0.005149859
-1.695099723
-0.012736214 0.006946719 -1.175468121 0.248447081
0.804932682 -6.72369007 cg09414638 6
152239860 q ESR1 ESR1 NA Body open
sea Body - open sea NA 0.011324792 -0.001411422
-0.012736214 -8.023675241
-0.007139321 0.013846251 -1.174010504 0.249022073
0.804932682 -6.725376739 cg20838323 6
29549759 p GABBR1/MOG SNORD32B NA TSS1500 open
sea TSS1500 - open sea NA 0.016300392 0.009161072
-0.007139321 1.779310625
0.003593232 -0.002405237 1.172586997 0.24958455
0.804932682 -6.727022084 cg13770529 17
28443770 q SLC6A4 CCDC55 NA TSS200 island
TSS200 - island NA -0.00364041 -4.72E-05
0.003593232 77.16302182
-0.031996198 -0.057828009 -1.17144423 0.250036769
0.804932682 -6.728341613 cg21156276 9 4491917
p SLC1A1 SLC1A1 NA Body shore Body -
shore NA -0.046829316 -0.078825514 -0.031996198
0.594088308
-0.003901782 -0.000959846 -1.171030941 0.250200464
0.804932682 -6.72881854 cg19128271 22
19842843 q COMT GNB1L NA TSS1500 island
TSS1500 - island NA 0.000381392 -0.003520391
-0.003901782 -0.108337852
-0.013141106 -0.050356649 -1.169273351 0.250897486
0.804932682 -6.730845031 cg07746998 6
152126785 q ESR1 ESR1 NA 5'UTR shelf
5'UTR - shelf NA -0.045839394 -0.058980499
-0.013141106 0.777195757
0.011601117 0.004955989 1.168392708 0.251247263
0.804932682 -6.731859357 cg12257233 6
29597083 p GABBR1/MOG GABBR1 NA TSS1500 shore
TSS1500 - shore NA 0.000968105 0.012569222
0.011601117 0.077021861
0.011167878 -0.016424412 1.162604027 0.2535553
0.807435342 -6.738509294 cg09205920 6
29521506 p GABBR1 UBD -1883 IGR island
IGR - island NA -0.02026337 -0.009095493
0.011167878 2.227847466
0.014522262 0.006962549 1.154693288 0.256734379
0.807435342 -6.747547918 cg25836061 22
19939028 q COMT COMT NA 5'UTR open
sea 5'UTR - open sea NA 0.001970521 0.016492783
0.014522262 0.119477791
-0.011155595 0.033003168 -1.15449413 0.256814787
0.807435342 -6.747774738 cg00114160 6
29430096 p GABBR1 OR2H1 NA Body open
sea Body - open sea NA 0.036837903 0.025682309
-0.011155595 1.434368836
-0.009635132 0.01145507 -1.153874898 0.257064912
0.807435342 -6.74847975 cg12867320 6

29574715 p GABBR1/MOG GABBR1 NA Body open
sea Body - open sea V$TAL1ALPHAE47_01 0.014767146
0.005132014 -0.009635132 2.87745644
0.009025868 0.013946907 1.152179346 0.257750697
0.807435342 -6.750408401 cg05219421 6
29691003 p GABBR1/MOG HLA-F NA TSS200 shore
TSS200 - shore NA 0.010844265 0.019870133
0.009025868 0.545757048
-0.009933715 0.017292934 -1.151745872 0.257926234
0.807435342 -6.750901049 cg03215160 6
29424926 p GABBR1 OR2H1 NA TSS1500 open
sea TSS1500 - open sea NA 0.020707648 0.010773933
-0.009933715 1.922013739
-0.02445283 -0.20441634 -1.149979626 0.258642379
0.807435342 -6.752906641 cg15626350 6
152130207 q ESR1 ESR1 NA Body shore
Body - shore NA -0.19601068 -0.22046351
-0.02445283 0.889084456
-0.012324389 -0.011617262 -1.146492397 0.260060551
0.807435342 -6.756858107 cg11629449 6
29556084 p GABBR1/MOG OR2H2 NA 1stExon open
sea 1stExon - open sea V$PPARG_01 -0.007380753 -0.019705142
-0.012324389 0.374559748
0.012839019 -0.043453434 1.145871066 0.260313822
0.807435342 -6.757560994 cg11358777 6
29638498 p GABBR1/MOG MOG NA 3'UTR open
sea 3'UTR - open sea NA -0.047866846 -0.035027827
0.012839019 1.366537696
-0.012078158 -0.001601734 -1.144963386 0.260684137
0.807435342 -6.758587183 cg24962941 6
29577916 p GABBR1/MOG GABBR1 NA Body open
sea Body - open sea NA 0.002550132 -0.009528025
-0.012078158 -0.267645429
0.016903766 -0.117920172 1.144808534 0.260747352
0.807435342 -6.758762178 cg01644592 6
29633971 p GABBR1/MOG MOG NA 3'UTR open
sea 3'UTR - open sea NA -0.123730841 -0.106827075
0.016903766 1.158234848
-0.012182939 -0.003228576 -1.144563067 0.26084758
0.807435342 -6.75903953 cg22179059 6
29714945 p MOG LOC285830 NA Body shore
Body - shore NA 0.000959309 -0.01122363
-0.012182939 -0.085472274
-0.008675585 -0.007643058 -1.142674248 0.261619754
0.807435342 -6.761171869 cg15710245 11
27722620 p BDNF BDNF NA TSS200 island
TSS200 - island NA -0.004660826 -0.013336411
-0.008675585 0.34948129
-0.011871468 0.014151472 -1.142064823 0.261869248
0.807435342 -6.761859172 cg10625096 6
29518072 p GABBR1 UBD -5317 IGR shelf
IGR - shelf NA 0.018232289 0.006360822
-0.011871468 2.866341877
-0.011620777 -0.008867584 -1.139529543 0.262909018
0.807435342 -6.76471481 cg12904135 2
171669275 q GAD1 GAD1 -3925 IGR shore

IGR - shore NA -0.004872942 -0.016493718
-0.011620777 0.29544228
-0.00653961 0.008038371 -1.138579012 0.263299619
0.807435342 -6.765783942 cg03810301 6
29590017 p GABBR1/MOG GABBR1 NA Body open
sea Body - open sea NA 0.010286362 0.003746752
-0.00653961 2.745407921
0.013323066 -0.0084672 1.137453545 0.263762648
0.807435342 -6.767048773 cg05218375 11
27723218 p BDNF BDNF NA TSS1500 shore
TSS1500 - shore NA -0.013047004 0.000276062
0.013323066 -47.26114589
-0.012095216 0.052949981 -1.136705616 0.264070679
0.807435342 -6.767888678 cg25798600 6
29454954 p GABBR1 MAS1L NA 1stExon open
sea 1stExon - open sea NA 0.057107712 0.045012495
-0.012095216 1.268707972
-0.004253554 0.003598635 -1.135430618 0.26459638
0.807435342 -6.769319294 cg00346247 6
29716851 p MOG LOC285830 NA TSS200 island
TSS200 - island NA 0.005060794 0.00080724 -
0.004253554 6.269253753
-0.019612909 -0.11165753 -1.134523237 0.264970966
0.807435342 -6.770336522 cg12991385 6
29599259 p GABBR1/MOG GABBR1 NA Body shore
Body - shore NA -0.104915592 -0.124528501
-0.019612909 0.842502651
0.008413495 -0.004921753 1.133608073 0.265349153
0.807435342 -6.771361714 cg02892153 18 3593461
p DLGAP1 FLJ35776 NA TSS1500 open sea TSS1500
- open sea NA -0.007813892 0.000599603 0.008413495
-13.03178113
-0.007423002 0.010628732 -1.133361704 0.26545103
0.807435342 -6.771637574 cg03175417 22
20050298 q COMT C22orf25 NA Body open
sea Body - open sea NA 0.013180389 0.005757387
-0.007423002 2.289300617
0.007654595 -0.008076293 1.129654931 0.266987242
0.807435342 -6.775781364 cg15673034 18 3499093
p DLGAP1 DLGAP1 NA 3'UTR island 3'UTR -
island NA -0.01070756 -0.003052965 0.007654595
3.507265745
-0.008786225 -0.053707274 -1.12923941 0.267159846
0.807435342 -6.776245092 cg20757073 6
29598333 p GABBR1/MOG GABBR1 NA Body shore
Body - shore NA -0.050687009 -0.059473234
-0.008786225 0.852265891
-0.004254406 -0.003151283 -1.127378188 0.26793397
0.807435342 -6.778320314 cg13773705 15
88799820 q NTRK3 NTRK3 NA TSS200 island
TSS200 - island NA -0.001688831 -0.005943237
-0.004254406 0.284160149
-0.008801214 -0.023401576 -1.124675025 0.269061148
0.807435342 -6.781328651 cg11147094 15
88420438 q NTRK3 NTRK3 NA Body open
sea Body - open sea NA -0.020376159 -0.029177373
-0.008801214 0.698354827

-0.023034021 0.028856482 -1.12380613 0.269424187
0.807435342 -6.782294223 cg19409546 12
72477363 q TPH2 TPH2 144737 IGR open
sea IGR - open sea NA 0.036774427 0.013740406
-0.023034021 2.676371211
0.014217968 -0.022215963 1.122955925 0.269779757
0.807435342 -6.783238358 cg09577455 6
29593382 p GABBR1/MOG GABBR1 NA Body shore
Body - shore NA -0.02710339 -0.012885422
0.014217968 2.103415036
0.008913919 -0.008319863 1.121274291 0.270484038
0.807435342 -6.785103832 cg11251858 6
152129036 q ESR1 ESR1 NA 5'UTR island
5'UTR - island NA -0.011384023 -0.002470104
0.008913919 4.608722885
-0.005329853 -0.004971786 -1.120176739 0.270944413
0.807435342 -6.786319977 cg20954537 11
27721668 p BDNF BDNF NA Body island
Body - island NA -0.00313965 -0.008469502
-0.005329853 0.370700602
-0.008017426 1.98E-05 -1.119299526 0.271312769
0.807435342 -6.787291184 cg25743221 6
152501416 q ESR1 SYNE1 NA Body open
sea Body - open sea NA 0.00277577 -0.005241656 -
0.008017426 -0.529559678
-0.003603384 -0.005058404 -1.117951675 0.271879454
0.807435342 -6.788782085 cg24000444 18 3771452
p DLGAP1 DLGAP1 NA Body island Body -
island NA -0.003819741 -0.007423125 -0.003603384
0.514573159
0.006637874 0.006841845 1.117634865 0.272012775
0.807435342 -6.789132278 cg12728623 22
19938992 q COMT COMT NA 5'UTR open
sea 5'UTR - open sea NA 0.004560076 0.01119795
0.006637874 0.407224162
-0.003724947 0.001012765 -1.116723425 0.272396592
0.807435342 -6.790139246 cg15688670 11
27723190 p BDNF BDNF NA TSS1500 shore
TSS1500 - shore NA 0.002293216 -0.001431732
-0.003724947 -1.601708089
-0.024854213 0.001612995 -1.115692074 0.272831374
0.807435342 -6.791277777 cg10648573 6
29648348 p GABBR1/MOG ZFP57 8179 IGR open
sea IGR - open sea NA 0.01015663 -0.014697583 -
0.024854213 -0.691040878
-0.017131297 0.053342613 -1.114636863 0.273276728
0.807435342 -6.79244164 cg08027810 6
29593479 p GABBR1/MOG GABBR1 NA Body shore
Body - shore NA 0.059231496 0.0421002 -
0.017131297 1.406917236
-0.01201629 -0.00634016 -1.112574544 0.274148636
0.808265805 -6.794713366 cg15999796 6
29623821 p GABBR1/MOG MOG NA TSS1500 open
sea TSS1500 - open sea NA -0.00220956 -0.01422585
-0.01201629 0.15532009
0.011533924 -0.025876023 1.103963615 0.277810666
0.808963442 -6.804156559 cg04765420 6

29698866 p MOG LOC285830 NA Body open
sea Body - open sea NA -0.029840809 -0.018306885
0.011533924 1.630032052
-0.003643646 -0.001585566 -1.103703031 0.277922028
0.808963442 -6.804441269 cg09503780 2
171786316 q GAD1 GORASP2 NA Body island
Body - island NA -0.000333063 -0.003976708
-0.003643646 0.083753324
0.003541674 -0.001870281 1.103411091 0.278046828
0.808963442 -6.804760165 cg05128992 18 3450160
p DLGAP1 TGIF1 NA 5'UTR island 5'UTR -
island NA -0.003087731 0.000453942 0.003541674
-6.802031808
-0.003791852 0.001061055 -1.102618409 0.278385888
0.808963442 -6.805625643 cg13604811 6
29720802 p MOG IFITM4P 2218 IGR island
IGR - island NA 0.002364504 -0.001427348
-0.003791852 -1.656571345
-0.004742364 4.12E-05 -1.102617738 0.278386175
0.808963442 -6.805626375 cg05737526 18 3454175
p DLGAP1 TGIF1 NA 5'UTR shore 5'UTR -
shore NA 0.001671432 -0.003070932 -0.004742364
-0.544275283
-0.006107128 0.024675991 -1.100887995 0.279127075
0.808963442 -6.807512967 cg13908968 9
87188847 q NTRK2 NTRK2 -94619 IGR open
sea IGR - open sea NA 0.026775317 0.020668189
-0.006107128 1.295484444
0.004756627 -0.002571615 1.097703383 0.280494809
0.808963442 -6.810979169 cg06971248 17
28618449 q SLC6A4 BLMH NA Body island
Body - island NA -0.004206705 0.000549922
0.004756627 -7.64964315
-0.010644933 -0.020333283 -1.096254437 0.281118681
0.808963442 -6.812553151 cg08451469 6
29711588 p MOG LOC285830 NA Body open
sea Body - open sea NA -0.016674087 -0.02731902
-0.010644933 0.610347196
-0.006827958 0.001062248 -1.09525715 0.281548654
0.808963442 -6.813635377 cg25414639 15
88360194 q NTRK3 NTRK3-AS1 -59794 IGR open
sea IGR - open sea NA 0.003409358 -0.003418599
-0.006827958 -0.997296842
-0.006096767 -0.003740373 -1.092694238 0.28265578
0.808963442 -6.816412383 cg25756033 17
28512754 q SLC6A4 CCDC55 NA 3'UTR open
sea 3'UTR - open sea NA -0.001644609 -0.007741377
-0.006096767 0.21244403
0.009363653 -0.012408305 1.091035573 0.283373935
0.808963442 -6.818206388 cg00674706 6
29521145 p GABBR1 UBD -2244 IGR island
IGR - island NA -0.015627061 -0.006263408
0.009363653 2.494977404
-0.009163067 -0.023126715 -1.088191123 0.284608512
0.808963442 -6.821277041 cg05489989 6
29456162 p GABBR1 MAS1L NA TSS1500 open

sea TSS1500 - open sea NA -0.019976911 -0.029139978
-0.009163067 0.685549966
-0.025188182 0.029181189 -1.08795346 0.284711837
0.808963442 -6.821533267 cg19103838 6
29562688 p GABBR1/MOG OR2H2 7005 IGR open
sea IGR - open sea NA 0.037839626 0.012651445
-0.025188182 2.99093323
0.029526208 0.057363199 1.086615587 0.285293979
0.808963442 -6.822974667 cg23098068 2
172650722 q SLC25A12 SLC25A12 NA Body open
sea Body - open sea NA 0.047213565 0.076739773
0.029526208 0.615242435
0.004239768 -0.002356378 1.085329568 0.285854353
0.808963442 -6.824358648 cg24759658 12
72233341 q TPH2 TBC1D15 NA TSS200 island
TSS200 - island NA -0.003813798 0.00042597
0.004239768 -8.953213969
0.007360671 -0.014808215 1.082541727 0.287071808
0.808963442 -6.827353622 cg21950534 6
152128483 q ESR1 ESR1 NA TSS1500 shore
TSS1500 - shore NA -0.017338445 -0.009977775
0.007360671 1.73770665
0.005657455 -0.009424115 1.076712326 0.289629351
0.808963442 -6.833592999 cg02720618 6
152129791 q ESR1 ESR1 NA Body shore
Body - shore NA -0.011368866 -0.005711411
0.005657455 1.990552934
-0.016413045 -0.071699776 -1.072522693 0.291477373
0.808963442 -6.838057931 cg01765653 6
29599160 p GABBR1/MOG GABBR1 NA Body shore
Body - shore NA -0.066057791 -0.082470837
-0.016413045 0.800983644
-0.023805718 -0.000654682 -1.071171616 0.292075091
0.808963442 -6.839494332 cg00504902 6
29692183 p GABBR1/MOG HLA-F NA Body shore
Body - shore NA 0.007528533 -0.016277185
-0.023805718 -0.462520618
0.009920687 0.016890911 1.070989492 0.292155729
0.808963442 -6.839687829 cg24703717 6
29691168 p GABBR1/MOG HLA-F NA 1stExon island
1stExon - island NA 0.013480675 0.023401362
0.009920687 0.576063678
-0.009622549 -0.000695567 -1.069601534 0.29277078
0.808963442 -6.841161452 cg18950940 6
29580896 p GABBR1/MOG GABBR1 NA Body open
sea Body - open sea NA 0.002612184 -0.007010364
-0.009622549 -0.372617451
-0.01378189 -0.030562842 -1.069512577 0.292810231
0.808963442 -6.841255839 cg04335343 6
29597113 p GABBR1/MOG GABBR1 NA TSS1500 shore
TSS1500 - shore NA -0.025825318 -0.039607207
-0.01378189 0.652035814
-0.007944753 0.002162549 -1.069178461 0.292958439
0.808963442 -6.841610283 cg20486877 6
29594481 p GABBR1/MOG GABBR1 NA Body shore
Body - shore NA 0.004893558 -0.003051195
-0.007944753 -1.603816732

0.003453343 0.002393878 1.066888051 0.293975844
0.808963442 -6.844037272 cg25777153 22
20008297 q COMT C22orf25 NA TSS1500 shore
TSS1500 - shore NA 0.001206791 0.004660134
0.003453343 0.258960553
0.013287155 0.017110865 1.064694302 0.294952637
0.808963442 -6.846357292 cg11219691 22
19967280 q COMT ARVCF NA Body island
Body - island NA 0.012543405 0.02583056
0.013287155 0.485603294
-0.009131773 -0.019724076 -1.061696318 0.296291204
0.808963442 -6.849520642 cg01583131 11
27744675 p BDNF BDNF NA TSS1500 shore
TSS1500 - shore NA -0.016585029 -0.025716802
-0.009131773 0.644910255
-0.025115348 0.032715771 -1.059461507 0.297291787
0.808963442 -6.851873315 cg09740560 6
29602390 p GABBR1/MOG GABBR1 NA TSS1500 shore
TSS1500 - shore NA 0.041349172 0.016233824
-0.025115348 2.547099946
-0.007408556 0.00490294 -1.05775084 0.298059295
0.808963442 -6.853671079 cg02342892 6
29431208 p GABBR1 OR2H1 NA 3'UTR open
sea 3'UTR - open sea NA 0.007449631 4.11E-05 -
0.007408556 181.3682249
0.009154984 -0.002247742 1.05512897 0.299238311
0.808963442 -6.85642118 cg02274336 6
29427807 p GABBR1 OR2H1 NA 5'UTR open
sea 5'UTR - open sea NA -0.005394768 0.003760216
0.009154984 -1.434696393
-0.012238113 0.093960291 -1.053423802 0.300006846
0.808963442 -6.858206328 cg03316098 6
29601398 p GABBR1/MOG GABBR1 NA TSS1500 shore
TSS1500 - shore NA 0.098167142 0.085929029
-0.012238113 1.142421173
-0.003319446 -0.002794469 -1.053091346 0.300156847
0.808963442 -6.858554063 cg22841338 6
29720580 p MOG IFITM4P 1996 IGR island
IGR - island NA -0.001653409 -0.004972855
-0.003319446 0.332486957
0.003125956 -0.003932292 1.052662557 0.300350391
0.808963442 -6.859002409 cg00033220 18 3451606
p DLGAP1 TGIF1 NA 5'UTR island 5'UTR -
island NA -0.005006839 -0.001880883 0.003125956
2.661961946
-0.003697144 -0.002320076 -1.050602783 0.30128133
0.808963442 -6.86115375 cg24399395 21
34392203 q OLIG2 OLIG2 -6013 IGR island
IGR - island NA -0.001049183 -0.004746327
-0.003697144 0.221051567
0.005532193 0.002624361 1.049575627 0.301746317
0.808963442 -6.862225098 cg24628013 6
29580367 p GABBR1/MOG GABBR1 NA Body open
sea Body - open sea NA 0.00072267 0.006254863
0.005532193 0.115537328
0.007064516 -0.000153166 1.049522966 0.301770169
0.808963442 -6.862279999 cg19700470 6

29589973	p	GABBR1/MOG GABBR1	NA	Body	open
sea	Body - open sea	NA	-0.002581594	0.004482923
0.007064516	-0.575872884
0.004096249	-0.008347159	1.049408208	0.301822153
0.808963442	-6.862399627	cg14545305 2
171670978 q	GAD1	GAD1	-2222	IGR	shore
IGR - shore	NA	-0.009755244	-0.005658995
0.004096249	1.72384747
0.00361085 -0.004565316	1.048637632	0.302171377
0.808963442	-6.863202596	cg17298239 18	3499253
p	DLGAP1	DLGAP1	NA	Body	island	Body -
island	NA	-0.005806546	-0.002195696	0.00361085
2.644512726
-0.005750156	-0.002749594	-1.047926754	0.302493796
0.808963442	-6.863942868	cg02039276 6
29719081	p	MOG	IFITM4P	NA	TSS200	shore
TSS200 - shore	NA	-0.000772978	-0.006523134
-0.005750156	0.118497934
-0.027692645	0.415722019	-1.046636097	0.303079785
0.808963442	-6.865285693	cg12423733 6
29454623	p	GABBR1	MAS1L	NA	1stExon	open
sea	1stExon - open sea	NA	0.425241365	0.397548721
-0.027692645	1.069658493
-0.011496681	0.012971837	-1.046116558	0.303315891
0.808963442	-6.865825796	cg07821417 22
19972146	q	COMT	ARVCF	NA	Body	shelf
Body - shelf	NA	0.016923821	0.00542714 -
0.011496681	3.118368278
-0.006216666	0.004003364	-1.045686495	0.303511431
0.808963442	-6.866272691	cg15320474 6
29528005	p	GABBR1/MOG UBD	NA	TSS1500	open
sea	TSS1500 - open sea	NA	0.006140343	-7.63E-05	-
0.006216666	-80.45248873
-0.005412686	0.013558302	-1.044341198	0.304123675
0.808963442	-6.867669535	cg21108220 6
29716658	p	MOG	LOC285830 NA	Body	island
Body - island	NA	0.015418913	0.010006227
-0.005412686	1.540931824
-0.01009418	-0.017440603	-1.041731231	0.305313917
0.808963442	-6.870374716	cg24727561 6
29524112	p	GABBR1	UBD	NA	Body	shelf
Body - shelf	NA	-0.013970728	-0.024064909
-0.01009418	0.580543583
0.004465045	-0.001734442	1.040983688	0.30565542
0.808963442	-6.871148365	cg23918507 18	3771380
p	DLGAP1	DLGAP1	NA	Body	island	Body -
island	NA	-0.003269301	0.001195744	0.004465045
-2.734115643
0.008510678	0.013448479	1.039669736	0.306256321
0.808963442	-6.872506945	cg14273545 9
87449508	q	NTRK2	NTRK2	NA	Body	open
sea	Body - open sea	NA	0.010522934	0.019033611
0.008510678	0.552860601
0.002769902	-0.000414461	1.038723785	0.306689433
0.808963442	-6.873484032	cg14481263 22
20008608	q	COMT	C22orf25	NA	TSS200	island

TSS200 - island NA -0.001366615 0.001403288
0.002769902 -0.973866374
-0.006113254 -0.005673667 -1.036019318 0.307930042
0.808963442 -6.87627293 cg00786657 15
88515810 q NTRK3 NTRK3 NA Body open
sea Body - open sea NA -0.003572236 -0.00968549
-0.006113254 0.368823505
-0.009989172 -0.03739503 -1.035388097 0.308220099
0.808963442 -6.876922879 cg05733135 11
27740876 p BDNF BDNF NA Body island
Body - island NA -0.033961253 -0.043950425
-0.009989172 0.772717276
-0.004042899 -0.008893993 -1.034039486 0.308840444
0.808963442 -6.87831026 cg19538089 2
171673547 q GAD1 GAD1 NA 5'UTR island
5'UTR - island NA -0.007504247 -0.011547146
-0.004042899 0.649878966
0.005342671 0.003248644 1.033155716 0.309247436
0.808963442 -6.87921852 cg17675298 22
19961051 q COMT ARVCF NA Body island
Body - island NA 0.0014121 0.006754772
0.005342671 0.209052277
-0.018330772 0.020361502 -1.032419287 0.309586858
0.808963442 -6.879974801 cg16518990 12
72244947 q TPH2 TBC1D15 NA Body open
sea Body - open sea NA 0.026662705 0.008331933
-0.018330772 3.200062433
0.005929479 -0.000840637 1.031379789 0.310066404
0.808963442 -6.881041462 cg06841846 17
28564094 q SLC6A4 SLC6A4 NA TSS1500 shore
TSS1500 - shore NA -0.002878896 0.003050584
0.005929479 -0.943719534
-0.004840876 -4.15E-05 -1.031011374 0.310236487
0.808963442 -6.881419263 cg06358612 17
28619293 q SLC6A4 BLMH NA TSS1500 shore
TSS1500 - shore NA 0.00162251 -0.003218366 -
0.004840876 -0.504140854
-0.004742563 0.000146401 -1.030659681 0.310398909
0.808963442 -6.881779797 cg04630810 9 4662735
p SLC1A1 C9orf68 NA Body island Body -
island NA 0.001776657 -0.002965906 -0.004742563
-0.599026586
0.008583597 0.022145305 1.030640219 0.310407898
0.808963442 -6.881799745 cg18595174 11
27701991 p BDNF BDNF NA Body open
sea Body - open sea NA 0.019194694 0.027778291
0.008583597 0.690996227
-0.007279114 -0.014029031 -1.030360131 0.310537296
0.808963442 -6.882086787 cg22305167 6
29521420 p GABBR1 UBD -1969 IGR island
IGR - island NA -0.011526835 -0.01880595
-0.007279114 0.612935566
-0.004600815 -0.005045071 -1.029930472 0.310735867
0.808963442 -6.882526972 cg15961225 2
171626884 q GAD1 GAD1 -46316 IGR shore
IGR - shore NA -0.003463541 -0.008064356
-0.004600815 0.429487598

-0.008826821	-0.00605981	-1.029910955	0.310744889
0.808963442	-6.882546963	cg22319611 22
19840851 q	COMT	GNB1L	NA	5'UTR	shore
5'UTR - shore	NA	-0.00302559	-0.011852412
-0.008826821	0.25527213
-0.002691895	0.00076668 -1.028747569	0.311283008
0.808963442	-6.883737966	cg11525479 15
88799523 q	NTRK3	NTRK3	NA	5'UTR	island
5'UTR - island	NA	0.001692019	-0.000999876
-0.002691895	-1.692229832
-0.01096718	-0.034745253	-1.027806655	0.311718695
0.808963442	-6.884700295	cg00287322 2
171670379 q	GAD1	GAD1	-2821	IGR	island
IGR - island	NA	-0.030975285	-0.041942465
-0.01096718	0.738518472
0.003583866	-0.000553363	1.025070193	0.312988195
0.808963442	-6.887494352	cg18095109 6
29720427 p	MOG	IFITM4P	1843	IGR	island
IGR - island	NA	-0.001785317	0.001798549
0.003583866	-0.992642829
0.00312761 -0.004415251	1.024354922	0.31332061 0.808963442
-6.888223528	cg16017358 2	172779282 q
SLC25A12 HAT1	NA	Body	island	Body - island
NA	-0.005490367	-0.002362757	0.00312761
2.323712285
0.003855725	-0.002202816	1.023203574	0.3138562
0.808963442	-6.889396257	cg17221604 6
29720989 p	MOG	IFITM4P	2405	IGR	island
IGR - island	NA	-0.003528222	0.000327503
0.003855725	-10.77309037
-0.008136875	0.007391664	-1.022730672	0.314076369
0.808963442	-6.889877583	cg13615337 6
29427915 p	GABBR1	OR2H1	NA	5'UTR	open
sea	5'UTR - open sea	NA	0.010188715	0.00205184 -
0.008136875	4.965648407
-0.003846362	-0.007422104	-1.022176286	0.31433461
0.808963442	-6.890441579	cg18588589 6
29521705 p	GABBR1	UBD	-1684	IGR	island
IGR - island	NA	-0.006099917	-0.009946279
-0.003846362	0.613286331
-0.006928101	-0.002506013	-1.020458289	0.315135808
0.808963442	-6.892187537	cg01321962 6
152126441 q	ESR1	ESR1	NA	5'UTR	shelf
5'UTR - shelf	NA	-0.000124478	-0.007052579
-0.006928101	0.017650012
-0.006204117	-0.012573872	-1.018227567	0.316178212
0.808963442	-6.894450459	cg27237300 21
34442292 q	OLIG2	OLIG1	NA	TSS200	island
TSS200 - island	NA	-0.010441207	-0.016645324
-0.006204117	0.62727568
0.008781755	0.006303853	1.01818418 0.31619851 0.808963442
-6.894494426	cg06787004 22	19938981 q	COMT
COMT	NA	5'UTR	open sea	5'UTR - open sea	NA
0.003285124	0.012066879	0.008781755
0.272243081
-0.009168208	-0.027150231	-1.016443715	0.317013501
0.808963442	-6.89625672	cg03443455 2

171671795 q GAD1 GAD1 NA TSS1500 island
TSS1500 - island NA -0.023998659 -0.033166867
-0.009168208 0.723573288
0.004893115 -0.004616296 1.014990258 0.317695202
0.808963442 -6.897726241 cg12801329 2
171670795 q GAD1 GAD1 -2405 IGR shore
IGR - shore NA -0.006298305 -0.00140519
0.004893115 4.482173989
-0.019897102 0.152809745 -1.013974419 0.318172247
0.808963442 -6.898752136 cg12463578 6
29644756 p GABBR1/MOG ZFP57 NA 1stExon open
sea 1stExon - open sea NA 0.159649374 0.139752272
-0.019897102 1.142374085
0.006830457 -0.011306874 1.012971999 0.318643473
0.808963442 -6.899763534 cg25980242 18 4454472
p DLGAP1 DLGAP1-AS5 189870 IGR island IGR -
island NA -0.013654844 -0.006824387 0.006830457
2.000889417
0.00475444 -0.006065401 1.011760454 0.319213645
0.808963442 -6.90098468 cg03732762 21
34443010 q OLIG2 OLIG1 NA 1stExon island
1stExon - island NA -0.00769974 -0.002945299
0.00475444 2.614246803
-0.007366753 -0.034346572 -1.010193777 0.319951983
0.808963442 -6.902561739 cg07671949 6
152128338 q ESR1 ESR1 NA TSS1500 shore
TSS1500 - shore V$OCT1_02 -0.03181425 -0.039181003
-0.007366753 0.811981511
-0.015222792 -0.001561123 -1.008642958 0.320683998
0.808963442 -6.904120576 cg25699073 6
29648381 p GABBR1/MOG ZFP57 8212 IGR open
sea IGR - open sea NA 0.003671712 -0.01155108
-0.015222792 -0.317867432
0.009630814 -0.024352487 1.008622138 0.320693834
0.808963442 -6.904141488 cg02115911 21
34395548 q OLIG2 OLIG2 -2668 IGR island
IGR - island NA -0.02766308 -0.018032266
0.009630814 1.534087857
-0.005825948 0.002213871 -1.008057509 0.320960639
0.808963442 -6.904708468 cg06872047 6
29585579 p GABBR1/MOG GABBR1 NA Body open
sea Body - open sea NA 0.004216541 -0.001609407
-0.005825948 -2.619934274
-0.005749987 0.004124738 -1.006665697 0.321618964
0.808963442 -6.906104801 cg12322235 6
29549724 p GABBR1/MOG SNORD32B NA TSS1500 open
sea TSS1500 - open sea NA 0.006101296 0.000351309
-0.005749987 17.3673086
-0.011368052 -0.047274359 -1.006244584 0.321818331
0.808963442 -6.906526925 cg14352032 17
28564834 q SLC6A4 BLMH -10379 IGR shore
IGR - shore NA -0.043366591 -0.054734643
-0.011368052 0.792306095
-0.007528172 0.042310573 -1.004767929 0.322518091
0.808963442 -6.908005815 cg25166896 22
20009063 q COMT C22orf25 NA 5'UTR island

5'UTR - island NA 0.044898382 0.03737021 -
0.007528172 1.201448469
-0.005034274 0.016202312 -1.004751707 0.322525784
0.808963442 -6.90802205 cg26069562 6
29691981 p GABBR1/MOG HLA-F NA Body island
Body - island NA 0.017932843 0.01289857 -
0.005034274 1.390297031
0.009360766 0.029390984 1.004014012 0.32287576
0.808963442 -6.908760087 cg24177217 6
29702053 p MOG LOC285830 NA Body open
sea Body - open sea NA 0.026173221 0.035533987
0.009360766 0.736568651
-0.015620451 0.012147116 -0.999528847 0.325009178
0.812820029 -6.913236373 cg06122864 6
29629187 p GABBR1/MOG MOG NA Body open
sea Body - open sea NA 0.017516646 0.001896195
-0.015620451 9.237787553
0.005674962 0.005218015 0.994993853 0.327176046
0.816439532 -6.917743244 cg06212631 2
171785547 q GAD1 GORASP2 NA TSS200 island
TSS200 - island NA 0.003267246 0.008942208
0.005674962 0.365373557
0.012053067 -0.015116164 0.994004491 0.327650075
0.816439532 -6.91872391 cg10364942 21
34401201 q OLIG2 OLIG2 NA 3'UTR shore
3'UTR - shore NA -0.019259406 -0.007206338
0.012053067 2.672564774
-0.007199604 -0.012583608 -0.991347371 0.328925482
0.816884535 -6.921353138 cg15814717 6
29521228 p GABBR1 UBD -2161 IGR island
IGR - island NA -0.010108744 -0.017308348
-0.007199604 0.584038644
-0.01401413 0.020479138 -0.989892767 0.329625112
0.816884535 -6.92278967 cg18389339 18 3730593
p DLGAP1 DLGAP1 NA Body shore Body -
shore NA 0.025296495 0.011282366 -0.01401413
2.242126891
-0.002774932 -0.001029602 -0.987823271 0.330622228
0.816884535 -6.924830034 cg19766164 6
29716939 p MOG LOC285830 NA TSS200 island
TSS200 - island NA -7.57E-05 -0.002850651 -
0.002774932 0.026561894
0.013560057 0.100941776 0.987193609 0.330926014
0.816884535 -6.925450035 cg25385819 18 3445098
p DLGAP1 TGIF1 NA 5'UTR shelf 5'UTR -
shelf NA 0.096280507 0.109840563 0.013560057
0.876547823
-0.013382866 0.099980069 -0.984298374 0.332325281
0.816884535 -6.928296062 cg11092416 6
29617549 p GABBR1/MOG MOG -7209 IGR shore
IGR - shore NA 0.104580429 0.091197563
-0.013382866 1.14674587
0.012163684 -0.067505719 0.983762662 0.332584629
0.816884535 -6.928821806 cg08755130 6
29705878 p MOG LOC285830 NA Body open
sea Body - open sea NA -0.071686985 -0.059523301
0.012163684 1.204351643

-0.001985431 -0.00099642 -0.983296821 0.332810262
0.816884535 -6.92927876 cg18587988 22
20008614 q COMT C22orf25 NA TSS200 island
TSS200 - island NA -0.000313928 -0.002299358
-0.001985431 0.136528435
-0.008806551 0.015626739 -0.982607515 0.333144321
0.816884535 -6.929954543 cg08196667 6
29549180 p GABBR1/MOG SNORD32B NA TSS1500 open
sea TSS1500 - open sea NA 0.018653991 0.009847439
-0.008806551 1.894298618
-0.009213551 0.00709676 -0.982396815 0.333246479
0.816884535 -6.93016102 cg15617548 2
171785815 q GAD1 GORASP2 NA 5'UTR island
5'UTR - island NA 0.010263918 0.001050368
-0.009213551 9.771739728
-0.007368847 -0.008134767 -0.980928361 0.333959041
0.816884535 -6.931598886 cg15479387 6
29712425 p MOG LOC285830 NA Body open
sea Body - open sea NA -0.005601726 -0.012970572
-0.007368847 0.431879601
-0.008617573 -0.001815932 -0.973500993 0.337578904
0.816884535 -6.938840502 cg23600322 18 3457201
p DLGAP1 TGIF1 NA Body open sea Body -
open sea NA 0.001146359 -0.007471214 -0.008617573
-0.153436748
0.006564112 0.007924149 0.972433349 0.338101402
0.816884535 -6.939877182 cg15983385 6
29589056 p GABBR1/MOG GABBR1 NA Body open
sea Body - open sea NA 0.005667736 0.012231848
0.006564112 0.46335891
-0.013230933 -0.007053427 -0.970741488 0.338930502
0.816884535 -6.941517779 cg09313122 11
27827916 p BDNF BDNF-AS 151474 IGR open
sea IGR - open sea NA -0.002505294 -0.015736227
-0.013230933 0.159205504
-0.006292654 -0.034431401 -0.970291149 0.339151422
0.816884535 -6.941954018 cg27254482 21
34398085 q OLIG2 OLIG2 NA TSS200 island
TSS200 - island NA -0.032268301 -0.038560955
-0.006292654 0.836812818
-0.004015635 0.001100074 -0.969382174 0.339597625
0.816884535 -6.942833953 cg01453816 6
29600108 p GABBR1/MOG GABBR1 NA Body shore
Body - shore V$TAXCREB_02 0.002480448 -
0.001535187 -0.004015635 -1.615730739
-0.012275638 0.000880479 -0.968575987 0.339993702
0.816884535 -6.943613733 cg11588423 6
29578191 p GABBR1/MOG GABBR1 NA Body open
sea Body - open sea NA 0.00510023 -0.007175409 -
0.012275638 -0.710792903
0.008688994 -0.026225358 0.968151971 0.340202144
0.816884535 -6.944023615 cg25627226 18 4455337
p DLGAP1 DLGAP1-AS5 190735 IGR island IGR -
island NA -0.0292122 -0.020523206 0.008688994
1.423374132
-0.00746511 0.001602232 -0.967860166 0.340345642
0.816884535 -6.944305595 cg06132455 22

19839565 q COMT C22orf29 NA Body shelf
Body - shelf NA 0.004168364 -0.003296746
-0.00746511 -1.264387266
-0.008050117 -0.003998938 -0.967815337 0.34036769
0.816884535 -6.944348907 cg06979684 11
27677125 p BDNF BDNF NA 3'UTR open
sea 3'UTR - open sea NA -0.001231711 -0.009281828
-0.008050117 0.132701297
-0.008560869 -0.011653993 -0.966334134 0.341096745
0.816884535 -6.945778937 cg23134100 6
29701494 p MOG LOC285830 NA Body open
sea Body - open sea NA -0.008711195 -0.017272064
-0.008560869 0.504351693
0.007254491 -0.027400797 0.966270104 0.341128284
0.816884535 -6.945840709 cg16971273 6
29634336 p GABBR1/MOG MOG NA 3'UTR open
sea 3'UTR - open sea NA -0.029894528 -0.022640037
0.007254491 1.320427516
0.004450787 0.002604619 0.965520784 0.341497524
0.816884535 -6.946563309 cg04057037 6
29600203 p GABBR1/MOG GABBR1 NA 5'UTR island
5'UTR - island NA 0.00107466 0.005525448
0.004450787 0.194492909
0.01217674 -0.034097634 0.965388308 0.341562832
0.816884535 -6.946691007 cg09781307 11
27648324 p BDNF BDNFOS NA Body open
sea Body - open sea V$GRE_C -0.038283388 -0.026106649
0.01217674 1.466422925
0.007887811 -0.010329301 0.963821211 0.34233601
0.817304819 -6.948200318 cg14843922 21
34398849 q OLIG2 OLIG2 NA 5'UTR island
5'UTR - island NA -0.013040736 -0.005152925
0.007887811 2.530744287
0.008609146 0.033796633 0.95959171 0.344428616
0.820111072 -6.952262307 cg23330212 11
27672697 p BDNF BDNFOS NA Body open
sea Body - open sea NA 0.030837239 0.039446385
0.008609146 0.781750693
-0.024161985 -0.194791952 -0.955999558 0.346212584
0.820111072 -6.955698944 cg04063345 6
152130058 q ESR1 ESR1 NA Body shore
Body - shore NA -0.18648627 -0.210648255
-0.024161985 0.885297007
-0.00528487 -0.005413453 -0.955883273 0.346270437
0.820111072 -6.955809991 cg23221504 2
171673110 q GAD1 GAD1 NA TSS200 island
TSS200 - island NA -0.003596779 -0.008881649
-0.00528487 0.404967461
0.00906566 0.006779642 0.955452375 0.346484871
0.820111072 -6.95622137 cg27204993 9 4662937
p SLC1A1 C9orf68 NA Body island Body -
island NA 0.003663321 0.012728981 0.00906566
0.287793712
0.004975426 -0.004404744 0.955404066 0.346508918
0.820111072 -6.95626748 cg07813142 2
171573223 q GAD1 SP5 NA Body island

Body - island NA -0.006115047 -0.00113962
0.004975426 5.365863383
-0.023491611 0.009545942 -0.953680273 0.347367678
0.820723634 -6.957911354 cg21013431 18 3495888
p DLGAP1 DLGAP1-AS1 -142 IGR shelf IGR -
shelf NA 0.017621184 -0.005870427 -0.023491611
-3.001686649
0.01990126 0.057633506 0.947086908 0.350665439
0.827086759 -6.964173147 cg11834473 6
29591753 p GABBR1/MOG GABBR1 NA Body shelf
Body - shelf NA 0.050792449 0.070693708
0.01990126 0.718486127
-0.015837962 -0.008395211 -0.945459234 0.351482731
0.827587566 -6.965712647 cg03570920 12
72332964 q TPH2 TPH2 NA Body open
sea Body - open sea NA -0.002950912 -0.018788874
-0.015837962 0.157056343
-0.005079691 -0.000694766 -0.943091665 0.352673796
0.828965212 -6.96794749 cg16448399 18 3880076
p DLGAP1 DLGAP1 NA 1stExon island 1stExon
- island NA 0.001051377 -0.004028314 -0.005079691
-0.260996892
-0.008278813 0.002889285 -0.936870017 0.355816485
0.830257781 -6.973795077 cg17706972 6
152126337 q ESR1 ESR1 NA 5'UTR shelf
5'UTR - shelf NA 0.005735127 -0.002543686
-0.008278813 -2.25465202
0.021658976 -0.066993702 0.935710537 0.356404202
0.830257781 -6.974880796 cg00274965 21
34405681 q OLIG2 OLIG2 7465 IGR island
IGR - island NA -0.074438975 -0.052779999
0.021658976 1.410363324
0.006820331 -0.009302184 0.93458358 0.356976047
0.830257781 -6.975934842 cg25412831 11
27742138 p BDNF BDNF NA Body island
Body - island NA -0.011646673 -0.004826342
0.006820331 2.413147234
0.007579289 -0.017858182 0.93436622 0.357086411
0.830257781 -6.976138002 cg18056738 6
29715160 p MOG LOC285830 NA Body shore
Body - shore NA -0.020463563 -0.012884274
0.007579289 1.588258899
0.007937856 0.010235829 0.93398776 0.357278625
0.830257781 -6.976491628 cg10810078 2
172543808 q SLC25A12 DYNC1I2 NA TSS200 shore
TSS200 - shore NA 0.007507192 0.015445047
0.007937856 0.486058184
0.012114386 -0.087988936 0.933800153 0.357373934
0.830257781 -6.976666874 cg12790373 6
29635347 p GABBR1/MOG MOG NA 3'UTR open
sea 3'UTR - open sea NA -0.092153256 -0.08003887
0.012114386 1.151356286
-0.01567085 0.002844478 -0.933209746 0.357673982
0.830257781 -6.977218166 cg23717106 18 3768748
p DLGAP1 DLGAP1 NA Body shelf Body -
shelf NA 0.008231333 -0.007439517 -0.01567085
-1.106433837

-0.018988549 -0.011241167 -0.932413282 0.358079014
0.830257781 -6.977961339 cg08022281 6
29648345 p GABBR1/MOG ZFP57 8176 IGR open
sea IGR - open sea NA -0.004713853 -0.023702402
-0.018988549 0.198876591
0.005259035 -0.010747709 0.931214716 0.358689098
0.83026512 -6.979078576 cg24772753 2 171573419 q
GAD1 SP5 NA Body island Body - island
NA -0.012555502 -0.007296467 0.005259035
1.720764518
-0.004798032 -0.014249678 -0.924404987 0.362168313
0.836902453 -6.985400386 cg21608605 6
152128258 q ESR1 ESR1 NA TSS1500 shore
TSS1500 - shore NA -0.012600354 -0.017398386
-0.004798032 0.724225443
0.006670084 -0.011459226 0.922130357 0.36333538
0.837201038 -6.987502239 cg15462887 11
27744049 p BDNF BDNF NA TSS1500 island
TSS1500 - island NA -0.013752068 -0.007081983
0.006670084 1.941838432
0.006859798 -0.006436973 0.920454974 0.36419656
0.837201038 -6.989047222 cg19549714 18 3447713
p DLGAP1 TGIF1 NA 5'UTR shore 5'UTR -
shore NA -0.008795028 -0.001935231 0.006859798
4.544692252
-0.002827202 -0.000307859 -0.920204539 0.364325403
0.837201038 -6.989277936 cg09697651 6
29716568 p MOG LOC285830 NA Body island
Body - island NA 0.000663992 -0.00216321
-0.002827202 -0.306947309
-0.009815417 -0.005416878 -0.919388428 0.364745482
0.837201038 -6.990029368 cg20830965 17
28619340 q SLC6A4 BLMH NA TSS1500 shore
TSS1500 - shore NA -0.002042829 -0.011858245
-0.009815417 0.172270754
0.009167049 0.000169711 0.911876425 0.36862702
0.844693072 -6.996916307 cg03747251 11
27722722 p BDNF BDNF NA TSS200 shore
TSS200 - shore NA -0.002981462 0.006185587
0.009167049 -0.482001447
0.004766075 0.005203579 0.909152749 0.370041001
0.8465152 -6.9994001 cg02039214 15 88521695 q NTRK3
NTRK3 NA Body open sea Body - open sea NA
0.003565241 0.008331316 0.004766075
0.427932486
0.005344826 0.003473099 0.905165272 0.372117433
0.849364526 -7.00302366 cg08907436 6
152125965 q ESR1 ESR1 NA 5'UTR shelf
5'UTR - shelf NA 0.001635815 0.006980641
0.005344826 0.234335917
0.003641557 -0.001925516 0.902198958 0.373667003
0.849364526 -7.005709436 cg03541635 22
20004063 q COMT ARVCF NA 5'UTR island
5'UTR - island NA -0.003177301 0.000464256
0.003641557 -6.843849011
-0.005089615 -0.010714092 -0.899526458 0.375066663
0.849364526 -7.008122011 cg22098375 6

29590966 p GABBR1/MOG GABBR1 NA Body open
sea Body - open sea NA -0.008964537 -0.014054151
-0.005089615 0.637856849
0.006086245 -0.002439599 0.899330831 0.375169252
0.849364526 -7.008298344 cg15242223 6
29573377 p GABBR1/MOG GABBR1 NA Body open
sea Body - open sea NA -0.004531745 0.0015545
0.006086245 -2.915243127
-0.005878783 0.00120128 -0.899250535 0.375211365
0.849364526 -7.008370711 cg09646983 6
152125861 q ESR1 ESR1 NA 5'UTR shelf
5'UTR - shelf NA 0.003222112 -0.002656672
-0.005878783 -1.212837811
0.002964751 0.00148863 0.896717202 0.3765416 0.849364526
-7.010650705 cg23105568 6 29617607 p
GABBR1/MOG MOG -7151 IGR shore IGR - shore
NA 0.000469497 0.003434248 0.002964751
0.136710269
0.005571595 -0.018187828 0.896156968 0.376836186
0.849364526 -7.011154089 cg15818307 6
29521430 p GABBR1 UBD -1959 IGR island
IGR - island NA -0.020103064 -0.014531469
0.005571595 1.383415787
-0.008087579 0.003084505 -0.894427932 0.377746297
0.849364526 -7.012705782 cg02405128 6
29432580 p GABBR1 OR2H1 6350 IGR open
sea IGR - open sea NA 0.00586461 -0.002222969 -
0.008087579 -2.6381882
0.006794029 -0.01307326 0.894371502 0.377776024
0.849364526 -7.012756376 cg03402459 6
29521407 p GABBR1 UBD -1982 IGR island
IGR - island NA -0.015408708 -0.008614679
0.006794029 1.788657297
-0.013808269 -0.073904208 -0.893547791 0.378210121
0.849364526 -7.013494554 cg16118803 6
29629716 p GABBR1/MOG MOG NA Body open
sea Body - open sea NA -0.069157615 -0.082965885
-0.013808269 0.833566899
-0.008177443 0.015293584 -0.888886334 0.380672771
0.849364526 -7.017659778 cg09144707 2
171678251 q GAD1 GAD1 NA Body shore
Body - shore NA 0.01810458 0.009927138 -
0.008177443 1.823746294
-0.002909887 0.00052812 -0.887050547 0.381645443
0.849364526 -7.019294447 cg19348622 6
29691760 p GABBR1/MOG HLA-F NA Body island
Body - island NA 0.001528394 -0.001381493
-0.002909887 -1.106334382
-0.004231465 -0.004732689 -0.886583508 0.381893154
0.849364526 -7.019709806 cg02527472 11
27743348 p BDNF BDNF NA TSS1500 shore
TSS1500 - shore NA -0.003278123 -0.007509587
-0.004231465 0.436525013
0.004171236 -0.001363693 0.886207561 0.382092625
0.849364526 -7.020044002 cg09575114 9 4666694
p SLC1A1 C9orf68 NA TSS200 shelf TSS200

- shelf	NA	-0.002797555	0.001373681	0.004171236
-2.036538757
-0.005326142	-0.01110831	-0.884473999	0.383013291
0.849364526	-7.021583296	cg27351358 11
27743258	p	BDNF	BDNF	NA	TSS1500	shore
TSS1500 - shore	NA	-0.009277449	-0.014603591
-0.005326142	0.635285436
-0.004959049	-0.000452679	-0.883901656	0.383317565
0.849364526	-7.022090869	cg04066686 6
29580347	p	GABBR1/MOG GABBR1	NA	Body	open
sea	Body - open sea	NA	0.001251994	-0.003707055
-0.004959049	-0.337732869
-0.005713678	0.022318705	-0.883193185	0.383694423
0.849364526	-7.022718734	cg08816824 6
29716244	p	MOG	LOC285830 NA	Body	shore
Body - shore	NA	0.024282782	0.018569104
-0.005713678	1.307698074
-0.007474202	-0.005510965	-0.881342591	0.384679933
0.849364526	-7.024356512	cg10110652 6
29589927	p	GABBR1/MOG GABBR1	NA	Body	open
sea	Body - open sea	NA	-0.002941708	-0.01041591
-0.007474202	0.282424488
-0.010004213	-0.023099635	-0.881317292	0.384693417
0.849364526	-7.024378879	cg24444631 6
29636366	p	GABBR1/MOG MOG	NA	3'UTR	open
sea	3'UTR - open sea	NA	-0.019660686	-0.0296649 -
0.010004213	0.662759242
0.008347943	0.004157515	0.879788602	0.385508741
0.849364526	-7.025729267	cg25699759 15
88576311	q	NTRK3	NTRK3	NA	Body	open
sea	Body - open sea	NA	0.001287909	0.009635852
0.008347943	0.133658052
-0.006897121	0.006376638	-0.879387687	0.385722752
0.849364526	-7.026083051	cg22166516 2
172641053 q	SLC25A12	SLC25A12	NA	3'UTR	open
sea	3'UTR - open sea	NA	0.008747523	0.001850402
-0.006897121	4.727362993
0.003405601	-0.002370372	0.877259832	0.386859882
0.849364526	-7.027958184	cg11594927 6
29720600	p	MOG	IFITM4P	2016	IGR	island
IGR - island	NA	-0.003541048	-0.000135447
0.003405601	26.14346275
0.005599709	0.000711995	0.876870887	0.387067966
0.849364526	-7.028300467	cg07159484 11
27722523	p	BDNF	BDNF	NA	Body	island
Body - island	NA	-0.001212905	0.004386804
0.005599709	-0.276489551
0.00205674 -0.000250724	0.876318856	0.387363423
0.849364526	-7.028786022	cg13433942 9	4679784
p	SLC1A1	CDC37L1	NA	1stExon	island	1stExon
- island	V$PAX5_01 -0.000957728	0.001099012	0.00205674 -
0.87144464
0.004528807	0.015677692	0.876314642	0.387365679
0.849364526	-7.028789727	cg05603292 6
29691631	p	GABBR1/MOG HLA-F	NA	Body	island
Body - island	NA	0.014120914	0.018649721
0.004528807	0.757164894

-0.014003988 -0.087859617 -0.875251316 0.387935199
0.849364526 -7.02972418 cg20139800 6
29599178 p GABBR1/MOG GABBR1 NA Body shore
Body - shore NA -0.083045746 -0.097049735
-0.014003988 0.855702973
0.007954172 0.038990037 0.873944857 0.388635673
0.849364526 -7.030870818 cg00089464 6
29717223 p MOG LOC285830 NA TSS1500 shore
TSS1500 - shore NA 0.036255791 0.044209963
0.007954172 0.820081902
-0.00906399 -0.004403009 -0.873878648 0.388671194
0.849364526 -7.030928884 cg10216820 12
72332539 q TPH2 TPH2 NA TSS200 open
sea TSS200 - open sea NA -0.001287262 -0.010351252
-0.00906399 0.124358104
-0.004392116 0.001596113 -0.871877607 0.389745701
0.84993975 -7.032681842 cg18344922 6 29595661 p
GABBR1/MOG GABBR1 NA Body island Body - island
NA 0.003105903 -0.001286213 -0.004392116
-2.414766273
-0.013187654 0.027270974 -0.870702894 0.390377373
0.84993975 -7.033709132 cg04351905 21 34441330 q
OLIG2 OLIG1 NA TSS1500 shore TSS1500 - shore
NA 0.03180423 0.018616576 -0.013187654
1.708382333
-0.004672225 -0.010252093 -0.869920733 0.390798321
0.84993975 -7.034392402 cg23009221 6 152128588 q
ESR1 ESR1 NA TSS1500 shore TSS1500 - shore
NA -0.008646016 -0.013318241 -0.004672225
0.649186025
0.007815649 0.022329324 0.86791661 0.391878228
0.850446197 -7.036140465 cg17053201 6
29593246 p GABBR1/MOG GABBR1 NA Body shelf
Body - shelf NA 0.019642695 0.027458343
0.007815649 0.715363429
0.005595548 -0.002999855 0.866760431 0.392502089
0.850446197 -7.037147172 cg12296326 6
29717114 p MOG LOC285830 NA TSS1500 island
TSS1500 - island NA -0.004923325 0.000672223
0.005595548 -7.32394452
-0.00783917 0.013225453 -0.866030647 0.392896196
0.850446197 -7.03778195 cg21661983 12
72353490 q TPH2 TPH2 NA Body open
sea Body - open sea NA 0.015920168 0.008080997
-0.00783917 1.970074624
-0.006281236 0.002509387 -0.864016148 0.393985394
0.851323462 -7.039531546 cg24936467 6
29598417 p GABBR1/MOG GABBR1 NA Body shore
Body - shore NA 0.004668562 -0.001612675
-0.006281236 -2.894918696
-0.007785955 0.00113356 -0.862965896 0.394554002
0.851323462 -7.04044215 cg03906120 6
29583573 p GABBR1/MOG GABBR1 NA Body open
sea Body - open sea NA 0.003809982 -0.003975973
-0.007785955 -0.958251606
-0.014663446 0.021407776 -0.861832109 0.39516842
0.851323462 -7.041423994 cg18204321 18 3453798

p DLGAP1 TGIF1 NA 5'UTR shore 5'UTR -
shore NA 0.026448336 0.01178489 -0.014663446
2.244258196
-0.004306809 -0.004417381 -0.857609758 0.397461903
0.85430363 -7.045069652 cg01963885 22 20004367 q
COMT ARVCF NA TSS200 island TSS200 - island
NA -0.002936916 -0.007243724 -0.004306809
0.405442755
-0.006075935 -0.002252165 -0.856987258 0.39780074
0.85430363 -7.045605684 cg00224929 2 171705463 q
GAD1 GAD1 NA Body open sea Body - open sea
NA -0.000163563 -0.006239497 -0.006075935
0.026214076
0.005463678 -0.009264208 0.854741373 0.399024728
0.855491494 -7.047536516 cg01350824 6
29550425 p GABBR1/MOG SNORD32B 396 IGR open
sea IGR - open sea NA -0.011142347 -0.005678669
0.005463678 1.962140495
0.012450379 0.066804955 0.852737569 0.400118785
0.855491494 -7.049255139 cg22906524 22
19960525 q COMT ARVCF NA Body island
Body - island V$MYCMAX_03;V$MYCMAX_02 0.062525137
0.074975517 0.012450379 0.833940733
-0.005699534 -0.022762597 -0.850041022 0.401594051
0.855491494 -7.05156183 cg05012697 15
88798331 q NTRK3 NTRK3 NA Body shore
Body - shore NA -0.020803382 -0.026502916
-0.005699534 0.7849469
-0.002563459 -0.001453006 -0.849321488 0.401988281
0.855491494 -7.052176157 cg00267325 6
29691936 p GABBR1/MOG HLA-F NA Body island
Body - island NA -0.000571817 -0.003135276
-0.002563459 0.182381836
0.002287671 -0.003039478 0.848721143 0.402317394
0.855491494 -7.052688341 cg17911882 9 4679326
p SLC1A1 CDC37L1 NA TSS1500 island TSS1500
- island NA -0.003825865 -0.001538194 0.002287671
2.487244442
0.00432322 -0.003648266 0.847917746 0.402758086
0.855491494 -7.053373218 cg24025650 6
29720582 p MOG IFITM4P 1998 IGR island
IGR - island NA -0.005134372 -0.000811153
0.00432322 6.329722568
-0.006404262 0.003869264 -0.847826672 0.402808062
0.855491494 -7.053450817 cg23742601 6
29589895 p GABBR1/MOG GABBR1 NA Body open
sea Body - open sea NA 0.006070729 -0.000333533
-0.006404262 -18.20129826
0.005409244 -0.005801207 0.843953481 0.40493706
0.855491494 -7.056743577 cg00729049 2
171679402 q GAD1 GAD1 NA Body island
Body - island NA -0.007660635 -0.002251391
0.005409244 3.402623707
-0.010000614 -0.018414634 -0.841971106 0.406029444
0.855491494 -7.058423296 cg26142965 6
29521803 p GABBR1 UBD -1586 IGR island

IGR - island NA -0.014976923 -0.024977537
-0.010000614 0.599615695
0.002310179 -0.003709096 0.839912125 0.40716599
0.855491494 -7.060163924 cg05141870 17
28444127 q SLC6A4 MIR423 NA Body shore
Body - shore NA -0.00450322 -0.002193041
0.002310179 2.053413302
-0.011606965 -0.013216033 -0.837374791 0.408569315
0.855491494 -7.062303335 cg05913325 6
29639793 p GABBR1/MOG MOG NA 3'UTR open
sea 3'UTR - open sea NA -0.009226139 -0.020833104
-0.011606965 0.442859553
-0.006775234 -0.004258851 -0.836421156 0.409097521
0.855491494 -7.063105813 cg01418645 11
27679469 p BDNF BDNF NA Body open
sea Body - open sea NA -0.001929864 -0.008705098
-0.006775234 0.221693533
0.012652236 -0.104514583 0.833763556 0.410571776
0.855491494 -7.065337547 cg23122901 22
19880135 q COMT TXNRD2 NA Body open
sea Body - open sea NA -0.108863788 -0.096211553
0.012652236 1.131504327
0.007780854 0.003760511 0.833428729 0.410757749
0.855491494 -7.065618237 cg18860567 6
29578925 p GABBR1/MOG GABBR1 NA Body open
sea Body - open sea NA 0.001085842 0.008866696
0.007780854 0.122462988
-0.010619659 -0.054783767 -0.83234357 0.41136084
0.855491494 -7.066527199 cg13524919 21
34396506 q OLIG2 OLIG2 -1710 IGR island
IGR - island NA -0.051133259 -0.061752918
-0.010619659 0.828029836
-0.015704708 -0.002508614 -0.83165183 0.411745571
0.855491494 -7.067106031 cg16438069 9 4488463
p SLC1A1 SLC1A1 -1964 IGR shore IGR -
shore NA 0.002889879 -0.012814828 -0.015704708
-0.225510581
-0.009589387 0.035682279 -0.83114732 0.412026309
0.855491494 -7.067527901 cg26701815 18 3446566
p DLGAP1 TGIF1 NA 5'UTR shore 5'UTR -
shore NA 0.038978631 0.029389244 -0.009589387
1.326289006
-0.010657872 0.005207746 -0.830802834 0.412218069
0.855491494 -7.067815819 cg12055610 6
29585658 p GABBR1/MOG GABBR1 NA Body open
sea Body - open sea NA 0.008871389 -0.001786483
-0.010657872 -4.965841067
-0.003293419 -0.000580815 -0.830467144 0.412404986
0.855491494 -7.068096276 cg02996397 6
29720477 p MOG IFITM4P 1893 IGR island
IGR - island NA 0.000551298 -0.002742121
-0.003293419 -0.201048045
0.004391861 -0.004864308 0.830041863 0.412641864
0.855491494 -7.068451426 cg14111380 6
29576818 p GABBR1/MOG GABBR1 NA Body open
sea Body - open sea NA -0.00637401 -0.001982149
0.004391861 3.215707367

0.005576877	-0.039543884	0.82980428	0.412774233
	0.855491494	-7.068649755	cg00860808	2
	171670500	q	GAD1	GAD1	-2700	IGR	island
	IGR - island	NA	-0.041460935	-0.035884058
	0.005576877	1.155413777
0.002485545	-0.001078558	0.828957326	0.413246324
	0.855491494	-7.06935633	cg18518183	2
	172544446	q	SLC25A12	DYNC1I2	NA	5'UTR	island
	5'UTR - island	NA	-0.001932964	0.000552581
	0.002485545	-3.498068263
-0.006513301	0.003613467	-0.82853795	0.413480208
	0.855491494	-7.06970594	cg16850687	18	3594398
	p	DLGAP1	DLGAP1	NA	Body	open sea	Body -
open sea	NA	0.005852414	-0.000660887	-0.006513301
	-8.855395053
-0.00683216	-0.008464383	-0.826995671	0.414341035
	0.855491494	-7.070990194	cg04385220	6
	29726705	p	MOG	IFITM4P	8121	IGR	open
sea	IGR - open sea	NA	-0.006115827	-0.012947988
	-0.00683216	0.472338067
0.010975945	-0.024720568	0.826052878	0.414867804
	0.855491494	-7.071774127	cg27241909	6
	29714043	p	MOG	LOC285830	NA	Body	shelf
	Body - shelf	NA	-0.028493549	-0.017517604
	0.010975945	1.626566556
-0.007184631	0.028131695	-0.82555335	0.415147074
	0.855491494	-7.072189138	cg18423469	18	3726858
	p	DLGAP1	DLGAP1	NA	Body	shelf	Body -
shelf	NA	0.030601412	0.023416781	-0.007184631
	1.306815502
0.002328861	-0.000278784	0.825389753	0.415238561
	0.855491494	-7.072325003	cg08729407	17
	28431896	q	SLC6A4	EFCAB5	NA	Body	open
sea	Body - open sea	NA	-0.00107933	0.001249531
	0.002328861	-0.863788373
-0.003165859	-0.004804748	-0.822162692	0.417045757
	0.857922701	-7.074999758	cg21709140	2
	171785487	q	GAD1	GORASP2	NA	TSS1500	island
	TSS1500 - island	NA	-0.003716484	-0.006882343
	-0.003165859	0.540002721
0.012153935	0.096051052	0.820472926	0.417993982
	0.858582233	-7.076396316	cg17514431	6
	29717656	p	MOG	LOC285830	NA	TSS1500	shore
	TSS1500 - shore	NA	0.091873136	0.104027072
	0.012153935	0.883165649
-0.015643317	-0.172299463	-0.818647308	0.419019935
	0.858583682	-7.077902058	cg23019585	6
	29635110	p	GABBR1/MOG	MOG	NA	3'UTR	open
sea	3'UTR - open sea	NA	-0.166922073	-0.18256539
	-0.015643317	0.914313896
0.002407914	-0.008219001	0.817212438	0.419827384
	0.858583682	-7.079083259	cg21614759	6
	152128426	q	ESR1	ESR1	NA	TSS1500	shore
	TSS1500 - shore	NA	-0.009046722	-0.006638807
	0.002407914	1.362702822
0.006731233	-0.005343548	0.814592235	0.42130433
	0.858583682	-7.08123511	cg21535772	2

171679906 q GAD1 GAD1 NA Body island
Body - island NA -0.00765741 -0.000926176
0.006731233 8.267766956
-0.001884891 -0.002734633 -0.814482451 0.421366282
0.858583682 -7.081325126 cg16723445 18 3451456
p DLGAP1 TGIF1 NA 5'UTR island 5'UTR -
island NA -0.002086702 -0.003971593 -0.001884891
0.52540682
0.010016229 0.019003319 0.814036036 0.421618256
0.858583682 -7.081691037 cg12546695 6
29425610 p GABBR1 OR2H1 NA TSS1500 open
sea TSS1500 - open sea NA 0.01556024 0.025576469
0.010016229 0.608381087
-0.006574449 0.006392947 -0.813784268 0.421760405
0.858583682 -7.081897319 cg05844420 2
171621820 q GAD1 SP5 49963 IGR open
sea IGR - open sea NA 0.008652914 0.002078465
-0.006574449 4.163127275
0.012764343 -0.028593902 0.812055131 0.422737473
0.859294001 -7.083312398 cg13813710 6
29726626 p MOG IFITM4P 8042 IGR open
sea IGR - open sea NA -0.032981645 -0.020217302
0.012764343 1.631357412
-0.006010097 0.011700604 -0.810339969 0.423708015
0.859542387 -7.084713187 cg24251942 15
88576320 q NTRK3 NTRK3 NA Body open
sea Body - open sea NA 0.013766575 0.007756477
-0.006010097 1.774848843
0.010761962 0.007668835 0.809619141 0.424116309
0.859542387 -7.085301046 cg00140112 18 3879595
p DLGAP1 DLGAP1 NA 1stExon island 1stExon
- island NA 0.003969411 0.014731373 0.010761962
0.269452863
-0.004320499 -0.002329625 -0.804973332 0.426753586
0.863198101 -7.089077803 cg08388004 11
27679632 p BDNF BDNF NA Body open
sea Body - open sea V$PAX5_02;V$P53_02 -0.000844454 -
0.005164953 -0.004320499 0.163496925
-0.002926142 -0.001387503 -0.804220122 0.427182101
0.863198101 -7.08968815 cg00370229 6
29521602 p GABBR1 UBD -1787 IGR island
IGR - island NA -0.000381642 -0.003307784
-0.002926142 0.115376982
-0.007064881 -0.00632673 -0.801030597 0.428999586
0.863316078 -7.09226663 cg00465975 22
19929557 q COMT TXNRD2 NA TSS200 shore
TSS200 - shore NA -0.003898177 -0.010963058
-0.007064881 0.35557386
0.00564171 -0.0045985 0.80020163 0.429472726 0.863316078 -
7.092935173 cg03200120 6 29641443 p GABBR1/MOG ZFP57
NA Body open sea Body - open sea NA -
0.006537838 -0.000896128 0.00564171 7.29565216
-0.003689084 -0.004661736 -0.799603993 0.429814029
0.863316078 -7.093416741 cg02100602 21
34396665 q OLIG2 OLIG2 -1551 IGR island
IGR - island NA -0.003393614 -0.007082698
-0.003689084 0.479141401

0.007992115	0.009611377	0.799082213	0.430112146
	0.863316078	-7.093836902	cg13698224	9
	87309394	q	NTRK2	NTRK2	NA	Body	open
sea	Body - open sea	NA	0.006864088	0.014856203
	0.007992115	0.462035145
0.008432462	0.009354389	0.79858581	0.430395881
	0.863316078	-7.094236384	cg17606558	6
	29456563	p	GABBR1	MAS1L	NA	TSS1500	open
sea	TSS1500 - open sea	NA	0.00645573	0.014888192
	0.008432462	0.433614104
-0.007713883	0.0007539	-0.796792113	0.431422072
	0.864107459	-7.095677877	cg08292919	6
	29565696	p	GABBR1/MOG	GABBR1	-4309	IGR	open
sea	IGR - open sea	NA	0.003405548	-0.004308335
	-0.007713883	-0.790455628
-0.003803117	-0.014939696	-0.795113015	0.432384045
	0.864768091	-7.097024451	cg04481212	11
	27740495	p	BDNF	BDNF	NA	Body	shore
	Body - shore	NA	-0.013632374	-0.017435491
	-0.003803117	0.78187499
0.010276687	0.021842301	0.793231547	0.433463502
	0.865551924	-7.098530073	cg12772565	6
	29618315	p	GABBR1/MOG	MOG	-6443	IGR	shore
	IGR - shore	NA	0.01830969	0.028586377
	0.010276687	0.640504044
0.002704375	-0.001226028	0.79132008	0.43456184	0.865551924
	-7.100056189	cg20973396	6	29691899	p
	GABBR1/MOG	HLA-F	NA	Body	island	Body - island
	NA	-0.002155657	0.000548718	0.002704375
	-3.928534908
-0.003149086	-0.008401191	-0.790892049	0.434808019
	0.865551924	-7.100397445	cg01009697	9
	87283470	q	NTRK2	NTRK2	NA	TSS1500	island
	TSS1500 - island	NA	-0.007318693	-0.010467779
	-0.003149086	0.699163876
-0.007235771	0.005575366	-0.789904423	0.435376367
	0.865551924	-7.101184169	cg21297992	22
	19961060	q	COMT	ARVCF	NA	Body	island
	Body - island	NA	0.008062662	0.000826891
	-0.007235771	9.750568829
-0.008582785	0.004219798	-0.787745733	0.436620188
	0.865551924	-7.102900449	cg02373484	6
	29631227	p	GABBR1/MOG	MOG	NA	Body	open
sea	Body - open sea	NA	0.007170131	-0.001412654
	-0.008582785	-5.075644584
0.005432125	0.010764533	0.786430704	0.437378947
	0.865551924	-7.103943757	cg16128363	18	3880558
	p	DLGAP1	DLGAP1	NA	TSS1500	shore	TSS1500
- shore	NA	0.00889724	0.014329365	0.005432125
	0.620909579
-0.006472134	-0.002492077	-0.786068468	0.437588093
	0.865551924	-7.10423085	cg04105250	2
	171679114	q	GAD1	GAD1	NA	Body	island
	Body - island	NA	-0.000267281	-0.006739415
	-0.006472134	0.039659316
0.007617975	0.032057803	0.785312748	0.438024621
	0.865551924	-7.104829393	cg13040666	6

29693534 p GABBR1/MOG HLA-F NA Body shore
Body - shore NA 0.029439124 0.037057099
0.007617975 0.794426024
-0.008760174 0.003318641 -0.784541512 0.438470383
0.865551924 -7.105439654 cg26074662 22
19969472 q COMT ARVCF NA Body shore
Body - shore NA 0.006329951 -0.002430224
-0.008760174 -2.604678328
-0.020474763 -0.177791189 -0.782997849 0.439363414
0.866065057 -7.106659384 cg21644740 6
29599248 p GABBR1/MOG GABBR1 NA Body shore
Body - shore NA -0.170752989 -0.191227752
-0.020474763 0.892929962
-0.002756583 -0.006301842 -0.781614086 0.440164869
0.866396461 -7.107750804 cg14372466 18 3448468
p DLGAP1 TGIF1 NA 5'UTR island 5'UTR -
island NA -0.005354267 -0.00811085 -0.002756583
0.660136362
0.001975856 0.00125663 0.777090118 0.442791207
0.870313751 -7.111306038 cg12173216 6
29720762 p MOG IFITM4P 2178 IGR island
IGR - island NA 0.00057743 0.002553286
0.001975856 0.22615158
0.006723104 0.010971355 0.774994953 0.444010704
0.871216466 -7.112945824 cg17085250 6
29591016 p GABBR1/MOG GABBR1 NA Body open
sea Body - open sea NA 0.008660288 0.015383393
0.006723104 0.562963481
0.012642554 0.04747668 0.773037447 0.445151889
0.871216466 -7.114474018 cg02333875 6
29618205 p GABBR1/MOG MOG -6553 IGR shore
IGR - shore NA 0.043130802 0.055773356
0.012642554 0.773322695
0.00294457 0.002781809 0.773021753 0.445161045
0.871216466 -7.114486255 cg14402472 6
29691165 p GABBR1/MOG HLA-F NA 1stExon island
1stExon - island NA 0.001769613 0.004714183
0.00294457 0.375380686
-0.006379232 -0.016460874 -0.768718375 0.447676003
0.871898731 -7.117832677 cg21288364 6
29524117 p GABBR1 UBD NA Body shelf
Body - shelf NA -0.014268013 -0.020647245
-0.006379232 0.691037137
-0.005664068 -0.018822972 -0.764485851 0.450157783
0.871898731 -7.121106438 cg05724110 21
34398532 q OLIG2 OLIG2 NA 5'UTR island
5'UTR - island NA -0.016875948 -0.022540016
-0.005664068 0.748710568
0.003756635 -0.002855705 0.764451127 0.450178178
0.871898731 -7.121133223 cg14589148 11
27743648 p BDNF BDNF NA TSS200 island
TSS200 - island NA -0.004147048 -0.000390413
0.003756635 10.62221615
-0.004640008 -0.001215307 -0.763831199 0.450542373
0.871898731 -7.12161124 cg06564500 22
19892663 q COMT TXNRD2 NA Body shore

Body - shore NA 0.000379696 -0.004260312
-0.004640008 -0.089123989
0.012294074 -0.029631322 0.763778874 0.45057312
0.871898731 -7.12165157 cg03150111 6
29718049 p MOG LOC285830 NA TSS1500 shore
TSS1500 - shore NA -0.03385741 -0.021563336
0.012294074 1.570137846
-0.008801308 0.011114733 -0.763167897 0.450932242
0.871898731 -7.122122287 cg10819807 6
29461549 p GABBR1 MAS1L 7006 IGR open
sea IGR - open sea NA 0.014140182 0.005338875
-0.008801308 2.648532271
0.00499588 -0.008392522 0.761685659 0.451804181
0.871898731 -7.123262743 cg18264657 18 4455862
p DLGAP1 DLGAP1-AS5 191260 IGR shore IGR -
shore NA -0.010109856 -0.005113975 0.00499588
1.976907357
0.005522961 -0.003337742 0.761102494 0.452147506
0.871898731 -7.123710853 cg04480313 6
29455322 p GABBR1 MAS1L NA 1stExon open
sea 1stExon - open sea NA -0.00523626 0.000286701
0.005522961 -18.26383019
-0.002473554 -0.001596725 -0.760696612 0.452386551
0.871898731 -7.124022542 cg10901968 17
28563108 q SLC6A4 SLC6A4 NA TSS200 island
TSS200 - island NA -0.000746441 -0.003219995
-0.002473554 0.231814423
0.002341742 -0.002922981 0.759733803 0.452953898
0.871898731 -7.124761269 cg17597787 2
171785529 q GAD1 GORASP2 NA TSS200 island
TSS200 - island NA -0.003727955 -0.001386213
0.002341742 2.68930848
0.015253126 -0.006623009 0.759387088 0.453158307
0.871898731 -7.12502707 cg14193550 6
29621467 p GABBR1/MOG MOG -3291 IGR shelf
IGR - shelf NA -0.011866271 0.003386855
0.015253126 -3.503625626
-0.002711652 0.001021946 -0.756330152 0.45496291
0.871898731 -7.127365528 cg10253022 22
19929467 q COMT TXNRD2 NA TSS200 island
TSS200 - island NA 0.001954077 -0.000757575
-0.002711652 -2.57938329
-0.004397863 -0.010797382 -0.75507094 0.455707493
0.871898731 -7.128326139 cg14384532 15
88800624 q NTRK3 NTRK3 NA TSS1500 island
TSS1500 - island NA -0.009285616 -0.013683479
-0.004397863 0.678600514
0.003597159 -0.007037709 0.754719436 0.455915468
0.871898731 -7.128594013 cg13504245 9
87282610 q NTRK2 NTRK2 NA TSS1500 shore
TSS1500 - shore NA -0.008274232 -0.004677073
0.003597159 1.769104574
0.015812567 -0.075389514 0.753482061 0.456648034
0.871898731 -7.129536037 cg23732781 6
29595016 p GABBR1/MOG GABBR1 NA Body shore
Body - shore NA -0.080825084 -0.065012517
0.015812567 1.243223429

0.016389448 -0.027710486 0.752800243 0.457051989
0.871898731 -7.130054473 cg17741339 6
152085619 q ESR1 ESR1 NA 5'UTR open
sea 5'UTR - open sea NA -0.033344359 -0.01695491
0.016389448 1.966649073
-0.00815011 -0.012504548 -0.752186866 0.457415573
0.871898731 -7.130520481 cg21632181 6
29700035 p MOG LOC285830 NA Body open
sea Body - open sea NA -0.009702948 -0.017853058
-0.00815011 0.543489405
-0.003866544 -0.002738591 -0.751680222 0.457716019
0.871898731 -7.130905122 cg21032292 21
34395093 q OLIG2 OLIG2 -3123 IGR shore
IGR - shore NA -0.001409466 -0.00527601
-0.003866544 0.2671462
-0.00436661 0.001612026 -0.749516833 0.45900024
0.871898731 -7.132544744 cg12074883 6
29591155 p GABBR1/MOG GABBR1 NA Body open
sea Body - open sea NA 0.003113048 -0.001253562
-0.00436661 -2.48336309
-0.006202239 0.000825804 -0.74930201 0.459127877
0.871898731 -7.132707308 cg26158180 15
88798666 q NTRK3 NTRK3 NA Body shore
Body - shore NA 0.002957823 -0.003244416
-0.006202239 -0.911666025
-0.00686689 0.000776226 -0.748603169 0.459543239
0.871898731 -7.133235834 cg01078434 6
29455532 p GABBR1 MAS1L NA 1stExon open
sea 1stExon - open sea NA 0.003136719 -0.00373017
-0.00686689 -0.840905111
-0.00727478 0.040408798 -0.747250205 0.46034801
0.871898731 -7.134257714 cg01976913 6
29430900 p GABBR1 OR2H1 NA 3'UTR open
sea 3'UTR - open sea NA 0.042909504 0.035634724
-0.00727478 1.204148626
-0.002665164 -0.005504192 -0.746821327 0.460603288
0.871898731 -7.134581269 cg19622911 18 3771570
p DLGAP1 DLGAP1 NA Body island Body -
island NA -0.004588042 -0.007253206 -0.002665164
0.632553681
-0.013898054 0.124149997 -0.745688259 0.461278114
0.871898731 -7.135435216 cg10433043 6
152432725 q ESR1 SYNE1 -10094 IGR open
sea IGR - open sea NA 0.128927453 0.115029399
-0.013898054 1.120821758
-0.006297955 -0.017669029 -0.745410748 0.46144348
0.871898731 -7.135644174 cg00619335 2
171670134 q GAD1 GAD1 -3066 IGR island
IGR - island V$CMYB_01 -0.015504107 -0.021802061
-0.006297955 0.711130303
-0.008428823 0.039300318 -0.742426547 0.463223931
0.874055638 -7.137886443 cg21879791 6
29594830 p GABBR1/MOG GABBR1 NA Body shore
Body - shore NA 0.042197726 0.033768903
-0.008428823 1.249603114
-0.004827067 -0.005593818 -0.733868357 0.468352149
0.877121418 -7.144268685 cg02348151 21

34442257 q OLIG2 OLIG1 NA TSS200 island
TSS200 - island NA -0.003934513 -0.00876158
-0.004827067 0.449064334
-0.005722635 -0.004732944 -0.73267363 0.46907066
0.877121418 -7.145153959 cg03366312 21
34388564 q OLIG2 OLIG2 -9652 IGR shelf
IGR - shelf NA -0.002765788 -0.008488423
-0.005722635 0.325830579
-0.007053731 0.004974381 -0.732594192 0.469118457
0.877121418 -7.145212772 cg14447193 9
87433864 q NTRK2 NTRK2 NA Body open
sea Body - open sea NA 0.0073991 0.00034537 -0.007053731
21.42371127
0.005445047 -0.006346778 0.732550128 0.469144971
0.877121418 -7.145245392 cg03724721 22
19939061 q COMT COMT NA 5'UTR open
sea 5'UTR - open sea NA -0.008218513 -0.002773466
0.005445047 2.963264426
-0.002906438 -0.002339874 -0.732419088 0.469223825
0.877121418 -7.14534239 cg17016394 21
34442360 q OLIG2 OLIG1 NA TSS200 island
TSS200 - island NA -0.001340786 -0.004247224
-0.002906438 0.315685165
0.012427413 -0.04687141 0.728777287 0.471418361
0.877121418 -7.148031392 cg20108357 11
27718978 p BDNF BDNF NA Body shelf
Body - shelf NA -0.051143333 -0.03871592
0.012427413 1.320989736
-0.005816055 -0.008377522 -0.72748605 0.472197877
0.877121418 -7.148981691 cg06477632 6
29526338 p GABBR1/MOG UBD NA Body open
sea Body - open sea NA -0.006378253 -0.012194308
-0.005816055 0.523051674
-0.006604022 -0.051221993 -0.725723661 0.473263026
0.877121418 -7.150276108 cg00858840 2
171573839 q GAD1 SP5 NA Body island
Body - island NA -0.048951861 -0.055555883
-0.006604022 0.881128299
-0.007525888 0.002012698 -0.724367452 0.47408363
0.877121418 -7.151270131 cg17036562 12
72335305 q TPH2 TPH2 NA Body open
sea Body - open sea NA 0.004599722 -0.002926165
-0.007525888 -1.571928238
0.004007312 -0.005725434 0.723623268 0.474534263
0.877121418 -7.15181481 cg01777019 6
152128805 q ESR1 ESR1 NA 5'UTR shore
5'UTR - shore NA -0.007102948 -0.003095636
0.004007312 2.29450368
0.003640573 0.001874394 0.722965816 0.474932581
0.877121418 -7.152295558 cg13654445 9
87636383 q NTRK2 NTRK2 NA 3'UTR open
sea 3'UTR - open sea NA 0.000622947 0.00426352
0.003640573 0.146110911
0.008754948 0.007437497 0.722423149 0.475261501
0.877121418 -7.152692053 cg05009707 6
29555799 p GABBR1/MOG OR2H2 NA 1stExon open

sea 1stExon - open sea NA 0.004427983 0.013182932
0.008754948 0.33588761
-0.00530711 -0.000855864 -0.721667873 0.475719505
0.877121418 -7.15324341 cg00040027 2
172747482 q SLC25A12 SLC25A12 NA Body shelf
Body - shelf NA 0.000968455 -0.004338655
-0.00530711 -0.223215557
-0.003503334 -0.002563949 -0.721382291 0.47589275
0.877121418 -7.153451741 cg14519950 9 4662766
p SLC1A1 C9orf68 NA Body island Body -
island NA -0.001359678 -0.004863012 -0.003503334
0.279595877
-0.006374532 0.000126378 -0.720133965 0.476650456
0.877121418 -7.154361455 cg15400220 6
29712541 p MOG LOC285830 NA Body shelf
Body - shelf NA 0.002317624 -0.004056908
-0.006374532 -0.571278363
-0.016983682 0.001914238 -0.719966819 0.476751963
0.877121418 -7.154483146 cg12588917 6
29692082 p GABBR1/MOG HLA-F NA Body island
Body - island NA 0.007752379 -0.009231303
-0.016983682 -0.839792512
-0.00708421 0.006591382 -0.719362253 0.477119214
0.877121418 -7.154923074 cg23991188 6
29607758 p GABBR1/MOG MOG -17000 IGR open
sea IGR - open sea NA 0.00902658 0.00194237 -0.00708421
4.647199402
0.003342073 0.003194558 0.718661598 0.477545039
0.877121418 -7.155432476 cg18293833 6
29600462 p GABBR1/MOG GABBR1 NA 5'UTR island
5'UTR - island NA 0.00204572 0.005387793
0.003342073 0.379695327
-0.002624129 -0.000564581 -0.717599806 0.478190761
0.877121418 -7.156203523 cg16525287 6
29720702 p MOG IFITM4P 2118 IGR island
IGR - island NA 0.000337463 -0.002286666
-0.002624129 -0.1475788
-0.002513479 -0.003215662 -0.717188899 0.478440785
0.877121418 -7.156501617 cg01075763 18 3449181
p DLGAP1 TGIF1 NA 5'UTR island 5'UTR -
island V$FOXJ2_01;V$HFH3_01 -0.002351654 -0.004865133 -
0.002513479 0.483368827
0.008244188 -0.009346682 0.716692355 0.478743017
0.877121418 -7.156861616 cg23174322 6
29627215 p GABBR1/MOG MOG NA Body open
sea Body - open sea NA -0.012180622 -0.003936433
0.008244188 3.094329435
-0.001742866 0.000369037 -0.715666962 0.479367489
0.877121418 -7.157604271 cg26332258 6
29720425 p MOG IFITM4P 1841 IGR island
IGR - island NA 0.000968147 -0.000774719
-0.001742866 -1.249676241
0.003491834 0.001447961 0.715292543 0.479595629
0.877121418 -7.157875193 cg05309760 6
152501942 q ESR1 SYNE1 NA Body open
sea Body - open sea NA 0.000247643 0.003739476
0.003491834 0.066223909

0.011476057 -0.084282581 0.713447362 0.480720835
0.877490386 -7.15920832 cg12292060 6
29597991 p GABBR1/MOG GABBR1 NA Body shelf
Body - shelf NA -0.088227475 -0.076751418
0.011476057 1.149522413
0.002866527 -0.004062584 0.711597021 0.481850699
0.877490386 -7.160541826 cg01388957 9 4679439
p SLC1A1 CDC37L1 NA TSS200 island TSS200
- island NA -0.005047952 -0.002181426 0.002866527
2.314061063
0.003654053 -0.000678179 0.711539667 0.481885745
0.877490386 -7.160583106 cg18116160 6
29575145 p GABBR1/MOG GABBR1 NA Body open
sea Body - open sea NA -0.00193426 0.001719793
0.003654053 -1.124704768
-0.011222262 -0.059763149 -0.710758634 0.482363136
0.877490386 -7.161144931 cg09641376 6
29520965 p GABBR1 UBD -2424 IGR shore
IGR - shore NA -0.055905496 -0.067127758
-0.011222262 0.832822337
-0.003395122 -0.000775815 -0.709554612 0.483099599
0.877663017 -7.162009856 cg11108676 15
88801004 q NTRK3 NTRK3 NA TSS1500 island
TSS1500 - island NA 0.000391259 -0.003003863
-0.003395122 -0.130251783
0.002688445 -0.001951703 0.707987256 0.484059259
0.87817849 -7.163133657 cg09859456 12 72234313 q
TPH2 TBC1D15 NA Body shore Body - shore
NA -0.002875856 -0.000187411 0.002688445
15.34520059
0.005098623 -0.020822987 0.706995219 0.484667222
0.87817849 -7.163843707 cg20627916 6 152128328 q
ESR1 ESR1 NA TSS1500 shore TSS1500 - shore
V$OCT1_02 -0.022575639 -0.017477016 0.005098623
1.291733047
0.003986835 -0.047888323 0.705403097 0.485643848
0.878784105 -7.164981251 cg17781710 2
171671480 q GAD1 GAD1 -1720 IGR shore
IGR - shore NA -0.049258798 -0.045271963
0.003986835 1.088064114
-0.008167699 -0.009828465 -0.70278283 0.487253575
0.880532221 -7.166847973 cg18371410 6
29699406 p MOG LOC285830 NA Body open
sea Body - open sea NA -0.007020819 -0.015188518
-0.008167699 0.462245167
0.006472305 0.009951368 0.695787394 0.491565878
0.887153194 -7.171798636 cg18078658 12
72374129 q TPH2 TPH2 NA Body open
sea Body - open sea NA 0.007726514 0.014198818
0.006472305 0.54416596
-0.021359403 -0.194232804 -0.692977051 0.493304325
0.889117676 -7.173773984 cg06512249 6
29599390 p GABBR1/MOG GABBR1 NA Body shore
Body - shore NA -0.186890509 -0.208249912
-0.021359403 0.897433794
0.004340795 -0.000391004 0.689018807 0.495758687
0.892365637 -7.176543014 cg05421799 2

172778343 q SLC25A12 HAT1 NA TSS1500 shore
TSS1500 - shore NA -0.001883152 0.002457643
0.004340795 -0.766243007
0.004033975 -0.007290357 0.687182324 0.496899738
0.893244207 -7.177822516 cg21010859 11
27740161 p BDNF BDNF NA Body shore
Body - shore NA -0.008677036 -0.004643061
0.004033975 1.868817991
-0.009981889 0.030981679 -0.685749294 0.497791131
0.893254086 -7.178818623 cg12646029 6
29427451 p GABBR1 OR2H1 NA 5'UTR open
sea 5'UTR - open sea NA 0.034412954 0.024431065
-0.009981889 1.408573657
0.004525542 0.003843795 0.684415913 0.498621339
0.893254086 -7.17974365 cg14656245 6
29627290 p GABBR1/MOG MOG NA Body open
sea Body - open sea NA 0.00228814 0.006813682
0.004525542 0.335815471
-0.00273189 0.002002394 -0.683570367 0.499148203
0.893254086 -7.180329338 cg25663764 22
19965534 q COMT ARVCF NA Body shore
Body - shore NA 0.002941481 0.000209591
-0.00273189 14.03435515
0.001855429 -0.004084858 0.679426016 0.501735045
0.893254086 -7.183189852 cg11582100 2
171673207 q GAD1 GAD1 NA 5'UTR island
5'UTR - island NA -0.004722662 -0.002867233
0.001855429 1.647115059
-0.002253902 -0.003239451 -0.678184329 0.502511532
0.893254086 -7.184043598 cg08541345 6
29716319 p MOG LOC285830 NA Body shore
Body - shore NA -0.002464672 -0.004718574
-0.002253902 0.522334024
0.005933831 -0.056484877 0.677746691 0.502785367
0.893254086 -7.184344143 cg18397357 17
28565258 q SLC6A4 BLMH -9955 IGR shelf
IGR - shelf NA -0.058524631 -0.052590801
0.005933831 1.112830199
0.002683479 -0.002051379 0.676562931 0.503526472
0.893254086 -7.185156138 cg23952754 17
28431834 q SLC6A4 EFCAB5 NA Body open
sea Body - open sea NA -0.002973825 -0.000290347
0.002683479 10.24232749
0.003672898 -0.008315512 0.674270044 0.504963675
0.893254086 -7.18672501 cg03984780 11
27722617 p BDNF BDNF NA TSS200 island
TSS200 - island NA -0.009578071 -0.005905173
0.003672898 1.621979858
-0.001747586 -0.002824838 -0.673716854 0.505310758
0.893254086 -7.187102747 cg23399426 2
171627790 q GAD1 GAD1 -45410 IGR island
IGR - island NA -0.002224105 -0.003971691
-0.001747586 0.559989522
-0.003275492 -0.009357867 -0.671458149 0.506729282
0.893254086 -7.188641938 cg06378770 6
29521714 p GABBR1 UBD -1675 IGR island

IGR - island NA -0.008231916 -0.011507408
-0.003275492 0.715357992
-0.004565577 -0.039729154 -0.669940967 0.50768334
0.893254086 -7.189672997 cg26949694 11
27742060 p BDNF BDNF NA Body island
Body - island NA -0.038159737 -0.042725314
-0.004565577 0.893141172
-0.00732303 -0.035443104 -0.669715283 0.507825342
0.893254086 -7.189826176 cg01335087 22
19950166 q COMT COMT NA Body open
sea Body - open sea NA -0.032925813 -0.040248843
-0.00732303 0.818056136
0.003944718 0.006524876 0.669640608 0.507872333
0.893254086 -7.189876849 cg05157433 6
29527885 p GABBR1/MOG UBD NA TSS200 open
sea TSS200 - open sea NA 0.005168879 0.009113597
0.003944718 0.567161287
-0.009703554 -0.037271732 -0.669503895 0.507958369
0.893254086 -7.189969606 cg11046380 2
172543743 q SLC25A12 DYNC1I2 NA TSS1500 shore
TSS1500 - shore NA -0.033936136 -0.04363969
-0.009703554 0.777643835
-0.003004795 -0.003380613 -0.668366035 0.508674757
0.893254086 -7.190740904 cg03409187 6
29521624 p GABBR1 UBD -1765 IGR island
IGR - island NA -0.002347714 -0.005352509
-0.003004795 0.438619405
0.003914286 0.006387832 0.667067381 0.509493056
0.893254086 -7.191619638 cg04419754 22
20044371 q COMT C22orf25 NA Body open
sea Body - open sea NA 0.005042296 0.008956582
0.003914286 0.562971009
-0.003992423 -0.004494545 -0.66656806 0.509807877
0.893254086 -7.191957061 cg12710376 15
88495888 q NTRK3 NTRK3 NA Body open
sea Body - open sea NA -0.003122149 -0.007114572
-0.003992423 0.438838635
0.002680359 -0.000761394 0.666406268 0.50990991
0.893254086 -7.192066342 cg14075496 9 4679516
p SLC1A1 CDC37L1 NA TSS200 island TSS200
- island NA -0.001682768 0.000997591 0.002680359
-1.686830898
0.002096097 -0.002672411 0.665774376 0.510308513
0.893254086 -7.192492898 cg09742688 2
171672899 q GAD1 GAD1 NA TSS1500 island
TSS1500 - island NA -0.003392945 -0.001296848
0.002096097 2.61630065
-0.002943126 -0.000623343 -0.665671031 0.51037372
0.893254086 -7.192562624 cg24809935 6
29720530 p MOG IFITM4P 1946 IGR island
IGR - island NA 0.000388356 -0.002554769
-0.002943126 -0.152012343
-0.005790628 0.002422878 -0.665284787 0.510617467
0.893254086 -7.192823123 cg09603409 6
29587016 p GABBR1/MOG GABBR1 NA Body open
sea Body - open sea NA 0.004413407 -0.001377221
-0.005790628 -3.204573236

-0.014324059 0.047411555 -0.662959456 0.512086261
0.894225711 -7.194388318 cg23892836 6
29692085 p GABBR1/MOG HLA-F NA Body island
Body - island NA 0.052335451 0.038011392
-0.014324059 1.376835959
-0.012901767 -0.009979494 -0.662336358 0.512480232
0.894225711 -7.194806823 cg25439807 18 3771151
p DLGAP1 DLGAP1 NA Body shore Body -
shore NA -0.005544512 -0.018446279 -0.012901767
0.300576175
-0.010852125 0.0002857 -0.660628544 0.513560894
0.894969812 -7.195951918 cg10600786 6
29719569 p MOG IFITM4P NA TSS1500 shore
TSS1500 - shore NA 0.004016118 -0.006836007
-0.010852125 -0.58749478
-0.004730253 0.004656531 -0.656050333 0.516463987
0.896928208 -7.199007427 cg05114858 6
29526343 p GABBR1/MOG UBD NA Body open
sea Body - open sea NA 0.006282556 0.001552303
-0.004730253 4.047249182
0.004680968 -0.00634631 0.655600543 0.516749684
0.896928208 -7.199306502 cg21265702 6
152201605 q ESR1 ESR1 NA Body open
sea Body - open sea NA -0.007955393 -0.003274425
0.004680968 2.42955387
0.01092613 0.016076532 0.655310157 0.516934176
0.896928208 -7.19949948 cg18704527 22
19962042 q COMT ARVCF NA Body shore
Body - shore NA 0.012320674 0.023246805
0.01092613 0.529994315
0.009789852 -0.034518587 0.654723076 0.517307278
0.896928208 -7.199889374 cg25661973 21
34400733 q OLIG2 OLIG2 NA 3'UTR shore
3'UTR - shore NA -0.037883849 -0.028093997
0.009789852 1.348467758
-0.003946965 -0.005205866 -0.652066023 0.518997714
0.898720092 -7.201649731 cg05818894 11
27740078 p BDNF BDNF NA Body shore
Body - shore V$SP1_Q6 -0.003849097 -0.007796062
-0.003946965 0.493723198
0.002232548 0.004849013 0.650610673 0.519924883
0.899187409 -7.202610978 cg09749751 6
29600125 p GABBR1/MOG GABBR1 NA Body shore
Body - shore NA 0.004081574 0.006314122
0.002232548 0.646419915
-0.002748431 -0.000607921 -0.648102832 0.521524666
0.89919901 -7.204262478 cg15156844 18 3880020 p
DLGAP1 DLGAP1 NA 1stExon island 1stExon - island
NA 0.000336852 -0.002411578 -0.002748431
-0.139681341
-0.004951455 -0.012410383 -0.647688407 0.521789288
0.89919901 -7.204534793 cg23105820 6 29624956 p
GABBR1/MOG MOG NA 1stExon open sea 1stExon - open
sea NA -0.01070832 -0.015659775 -0.004951455
0.683810593
-0.00509257 -0.003501121 -0.647509545 0.521903519
0.89919901 -7.204652269 cg13473117 22 19867665 q

COMT TXNRD2 NA Body open sea Body - open sea
NA -0.00175055 -0.00684312 -0.00509257
0.255811691
-0.006002448 0.014384386 -0.64390571 0.524207985
0.900537491 -7.207012527 cg20704972 6
29574700 p GABBR1/MOG GABBR1 NA Body open
sea Body - open sea NA 0.016447727 0.010445279
-0.006002448 1.574656595
-0.005048335 -0.015641779 -0.643566747 0.524425015
0.900537491 -7.207233863 cg06515159 21
34400659 q OLIG2 OLIG2 NA 3'UTR shore
3'UTR - shore NA -0.013906414 -0.018954749
-0.005048335 0.733663867
0.010866128 -0.02635758 0.642990573 0.524794036
0.900537491 -7.207609833 cg15094605 6
29429346 p GABBR1 OR2H1 NA 5'UTR open
sea 5'UTR - open sea NA -0.030092812 -0.019226684
0.010866128 1.565158722
-0.0050301 0.003392302 -0.642179815 0.525313536
0.900537491 -7.20813832 cg18773129 22
19938916 q COMT COMT NA 5'UTR open
sea 5'UTR - open sea NA 0.005121399 9.13E-05 -
0.0050301 56.094814
-0.003372349 -0.014031354 -0.639775678 0.526855628
0.90062591 -7.20970162 cg02149189 6 29521138 p
GABBR1 UBD -2251 IGR island IGR - island
NA -0.012872109 -0.016244458 -0.003372349
0.792400037
-0.007928953 0.063955664 -0.639176985 0.527240025
0.90062591 -7.210090034 cg16834011 22 19931790 q
COMT COMT NA 5'UTR shelf 5'UTR - shelf
NA 0.066681242 0.058752289 -0.007928953
1.134955643
-0.003450267 0.022671557 -0.638885384 0.527427304
0.90062591 -7.210279087 cg14738290 6 29690998 p
GABBR1/MOG HLA-F NA TSS200 shore TSS200 - shore
NA 0.023857586 0.020407319 -0.003450267
1.16907008
0.002593862 -0.011270042 0.637414606 0.528372448
0.90062591 -7.211231354 cg27357571 21 34398226 q
OLIG2 OLIG2 NA TSS200 island TSS200 - island
NA -0.012161682 -0.00956782 0.002593862
1.271102745
-0.007507486 -0.023804627 -0.636972271 0.528656875
0.90062591 -7.211517329 cg09755181 6 29712115 p
MOG LOC285830 NA Body open sea Body - open sea
NA -0.021223929 -0.028731415 -0.007507486
0.738701143
-0.002283244 -0.00547145 -0.630855395 0.532598465
0.905697345 -7.21545211 cg20104535 2
172750996 q SLC25A12 SLC25A12 NA TSS200 island
TSS200 - island NA -0.004686585 -0.006969829
-0.002283244 0.672410333
0.004625899 -0.001022731 0.629024431 0.533781324
0.905697345 -7.216622706 cg13206902 6
29523786 p GABBR1 UBD NA Body shore

Body - shore NA -0.002612884 0.002013016
0.004625899 -1.297994899
-0.003284157 0.007206774 -0.628019063 0.534431413
0.905697345 -7.21726406 cg24640415 6
29574953 p GABBR1/MOG GABBR1 NA Body open
sea Body - open sea V$ZID_01 0.008335703 0.005051546
-0.003284157 1.650129088
0.00649212 0.001912291 0.626943796 0.535127162
0.905697345 -7.217948896 cg25189241 2
171785027 q GAD1 GORASP2 NA TSS1500 shore
TSS1500 - shore NA -0.000319375 0.006172745
0.00649212 -0.051739557
-0.007373549 -0.056054065 -0.6264703 0.535433688
0.905697345 -7.218250102 cg09842161 6
29598321 p GABBR1/MOG GABBR1 NA Body shore
Body - shore V$BRN2_01 -0.053519407 -0.060892956
-0.007373549 0.878909655
0.004928425 -0.007110692 0.625587295 0.536005566
0.905697345 -7.218811216 cg15154411 6
29576987 p GABBR1/MOG GABBR1 NA Body open
sea Body - open sea NA -0.008804839 -0.003876414
0.004928425 2.271387806
-0.00541661 -0.004612823 -0.624361033 0.536800286
0.905697345 -7.219589176 cg06542928 2
171608308 q GAD1 SP5 36451 IGR open
sea IGR - open sea NA -0.002750863 -0.008167473
-0.00541661 0.336807163
0.002097653 -0.007177224 0.623989471 0.537041212
0.905697345 -7.219824607 cg26057780 11
27721277 p BDNF BDNF NA Body shore
Body - shore NA -0.007898293 -0.00580064
0.002097653 1.36162442
-0.007011524 0.043663333 -0.622343122 0.538109412
0.905697345 -7.220866127 cg26751972 6
29692000 p GABBR1/MOG HLA-F NA Body island
Body - island NA 0.046073544 0.03906202 -
0.007011524 1.17949721
0.008431679 -0.022140197 0.622119881 0.538254343
0.905697345 -7.221007148 cg14730102 21
34401634 q OLIG2 OLIG2 3418 IGR shore
IGR - shore NA -0.025038587 -0.016606908
0.008431679 1.507721193
-0.016896282 -0.013601384 -0.620951148 0.539013436
0.905860418 -7.221744628 cg15331332 6
29692111 p GABBR1/MOG HLA-F NA Body shore
Body - shore NA -0.007793287 -0.024689569
-0.016896282 0.315651006
-0.001997623 -0.001325512 -0.616625923 0.541827552
0.908417275 -7.224462104 cg03539474 2
172751088 q SLC25A12 SLC25A12 NA TSS1500 shore
TSS1500 - shore NA -0.000638829 -0.002636453
-0.001997623 0.2423063
0.002685535 -0.004814198 0.616281482 0.542051985
0.908417275 -7.224677714 cg26391350 2
171673572 q GAD1 GAD1 NA 5'UTR island
5'UTR - island NA -0.00573735 -0.003051815
0.002685535 1.879979342

-0.005279362	0.035536321	-0.615552738	0.542526984
0.908417275	-7.225133498	cg02965092 12
72347052 q	TPH2	TPH2	NA	Body	open
sea	Body - open sea	NA	0.037351101	0.03207174 -
0.005279362	1.16461102
0.007109489	0.00564108 0.613231955	0.544041129
0.908936404	-7.226581496	cg21346639 18	3447463
p	DLGAP1	TGIF1	NA	5'UTR	shore	5'UTR -
shore	NA	0.003197194	0.010306682	0.007109489
0.310205891
-0.008923804	0.019772301	-0.612065462	0.544803013
0.908936404	-7.227307284	cg18217459 6
152429948 q	ESR1	SYNE1	-12871	IGR	open
sea	IGR - open sea	NA	0.022839859	0.013916055
-0.008923804	1.641259631
-0.010618966	-0.008409222	-0.612023699	0.544830301
0.908936404	-7.227333244	cg15018934 6
29689744 p	GABBR1/MOG HLA-F	NA	TSS1500	shore
TSS1500 - shore	NA	-0.004758953	-0.015377919
-0.010618966	0.30946663
-0.002964584	0.007135862	-0.609522247	0.546466011
0.910292244	-7.228884984	cg24544803 6
29596840 p	GABBR1/MOG GABBR1	NA	TSS1500	shore
TSS1500 - shore	NA	0.008154938	0.005190354
-0.002964584	1.571171854
0.002556301	-0.000931434	0.6072041 0.54798413 0.910292244
-7.230317469	cg10672884 2	171785479 q	GAD1
GORASP2	NA	TSS1500	island	TSS1500 - island	NA
-0.001810163	0.000746138	0.002556301	-
2.426042739
-0.006327275	-0.005727719	-0.606907581	0.548178473
0.910292244	-7.230500316	cg03898786 6
29586020 p	GABBR1/MOG GABBR1	NA	Body	open
sea	Body - open sea	NA	-0.003552718	-0.009879992
-0.006327275	0.35958711
0.005667673	0.000177184	0.606715043	0.548304685
0.910292244	-7.230618998	cg24217789 6
29717384 p	MOG	LOC285830 NA	TSS1500	shore
TSS1500 - shore	NA	-0.001771079	0.003896594
0.005667673	-0.454519765
0.006323603	-0.005257409	0.603344618	0.550516479
0.912856416	-7.232690579	ch.6.2949012F	6
152044517 q	ESR1	ESR1	NA	5'UTR	open
sea	5'UTR - open sea	NA	-0.007431148	-0.001107544
0.006323603	6.709570524
-0.008662019	0.015923713	-0.601755687	0.551560786
0.913480818	-7.23366328	cg06346307 22
19949965 q	COMT	COMT	NA	5'UTR	open
sea	5'UTR - open sea	NA	0.018901281	0.010239263
-0.008662019	1.84596116
-0.002306286	0.002437938	-0.595909255	0.555412044
0.918746888	-7.237220714	cg23666278 2
171785822 q	GAD1	GORASP2	NA	5'UTR	island
5'UTR - island	NA	0.003230723	0.000924437
-0.002306286	3.494800359
-0.017025666	0.016012638	-0.59256588	0.557620614
0.921286231	-7.239239822	cg20228636 6

29648525 p GABBR1/MOG ZFP57 8356 IGR open
sea IGR - open sea NA 0.021865211 0.004839545
-0.017025666 4.518030194
-0.002572631 -0.005122365 -0.590665754 0.558877792
0.921615155 -7.24038238 cg06860277 22
19930072 q COMT COMT NA 5'UTR shore
5'UTR - shore NA -0.004238023 -0.006810654
-0.002572631 0.622263674
-0.002253049 -0.001702394 -0.590195087 0.559189422
0.921615155 -7.24066484 cg15202447 6
29720635 p MOG IFITM4P 2051 IGR island
IGR - island NA -0.000927908 -0.003180957
-0.002253049 0.291707112
0.007551253 -0.01398653 0.588334532 0.560422164
0.921615155 -7.241779253 cg24938286 6
29631295 p GABBR1/MOG MOG NA Body open
sea Body - open sea NA -0.016582273 -0.00903102
0.007551253 1.836146228
-0.005570312 -0.02631362 -0.588195284 0.56051448
0.921615155 -7.24186252 cg16101636 6
29711438 p MOG LOC285830 NA Body open
sea Body - open sea NA -0.024398826 -0.029969137
-0.005570312 0.814131735
0.001980526 -0.000104842 0.584835303 0.562744365
0.921837631 -7.243865852 cg25456960 6
29717019 p MOG LOC285830 NA TSS200 island
TSS200 - island NA -0.000785648 0.001194878
0.001980526 -0.657512961
0.003374045 0.002955726 0.583844316 0.563402899
0.921837631 -7.244454566 cg20592995 17
28524160 q SLC6A4 SLC6A4 NA 3'UTR open
sea 3'UTR - open sea NA 0.001795898 0.005169943
0.003374045 0.347372826
0.009525301 -0.045908445 0.582936849 0.564006273
0.921837631 -7.244992806 cg10915739 21
34405733 q OLIG2 OLIG2 7517 IGR island
IGR - island NA -0.049182767 -0.039657466
0.009525301 1.240189341
0.00281884 -0.003167307 0.581803522 0.564760279
0.921837631 -7.245663858 cg26852170 6
29720448 p MOG IFITM4P 1864 IGR island
IGR - island NA -0.004136283 -0.001317443
0.00281884 3.139629191
-0.003382087 -0.001114063 -0.581404871 0.565025623
0.921837631 -7.245899598 cg10833114 12
72246108 q TPH2 MRS2P2 NA TSS1500 open
sea TSS1500 - open sea NA 4.85E-05 -0.003333557 -
0.003382087 -0.014557856
-0.013374065 0.073014468 -0.580211823 0.565820096
0.921837631 -7.246604156 cg06297863 6
29626990 p GABBR1/MOG MOG NA Body open
sea Body - open sea NA 0.077611803 0.064237737
-0.013374065 1.208196392
0.00771877 0.000520381 0.577812842 0.567419323
0.921837631 -7.248016592 cg11450715 2
171627404 q GAD1 GAD1 -45796 IGR island

IGR - island NA -0.002132946 0.005585823
0.00771877 -0.381849919
-0.002994789 -0.004373837 -0.577687147 0.567503178
0.921837631 -7.248090439 cg15370577 6
29580340 p GABBR1/MOG GABBR1 NA Body open
sea Body - open sea NA -0.003344379 -0.006339168
-0.002994789 0.527573792
-0.005613694 0.002814118 -0.577334012 0.567738796
0.921837631 -7.248297825 cg23426002 11
27679729 p BDNF BDNF NA Body open
sea Body - open sea NA 0.004743825 -0.000869869
-0.005613694 -5.453492334
-0.007476496 0.022738547 -0.576132218 0.568541023
0.921837631 -7.249002671 cg21147063 21
34354927 q OLIG2 OLIG2 -43289 IGR shelf
IGR - shelf NA 0.025308593 0.017832097
-0.007476496 1.41927185
-0.008710813 -0.069662438 -0.575938623 0.568670305
0.921837631 -7.249116078 cg06343355 6
29521023 p GABBR1 UBD -2366 IGR shore
IGR - shore NA -0.066668096 -0.07537891
-0.008710813 0.884439648
-0.006735622 -0.080671736 -0.575840133 0.568736082
0.921837631 -7.24917376 cg05812266 6
29595194 p GABBR1/MOG GABBR1 NA Body shore
Body - shore NA -0.078356366 -0.085091988
-0.006735622 0.920843052
-0.00572842 -0.000373688 -0.573635936 0.570209163
0.922085 -7.25046213 cg24782003 18 4459167 p
DLGAP1 DLGAP1-AS5 194565 IGR shelf IGR - shelf
NA 0.001595456 -0.004132964 -0.00572842
-0.386031874
-0.003381569 0.009289904 -0.571913441 0.571361643
0.922085 -7.251465572 cg22124493 6 29717032 p
MOG LOC285830 NA TSS1500 island TSS1500 - island
NA 0.010452318 0.007070749 -0.003381569
1.478247638
0.00186353 -0.001187219 0.571844534 0.571407771
0.922085 -7.251505653 cg21472700 2 172543946 q
SLC25A12 DYNC1I2 NA TSS200 island TSS200 - island
NA -0.001827808 3.57E-05 0.00186353 -51.16636746
-0.007205555 0.044733463 -0.571361488 0.571731187
0.922085 -7.251786488 cg11565911 12 72233249 q
TPH2 TBC1D15 NA TSS1500 shore TSS1500 - shore
NA 0.047210373 0.040004818 -0.007205555
1.180117172
0.005270956 0.000315761 0.570411101 0.57236777
0.922085 -7.252338349 cg12550837 6 29697949 p
MOG LOC285830 NA Body open sea Body - open sea
NA -0.001496131 0.003774826 0.005270956
-0.396344301
-0.002090874 -0.001587426 -0.568295035 0.573786406
0.922085 -7.253563851 cg13709765 18 3451484 p
DLGAP1 TGIF1 NA 5'UTR island 5'UTR - island
NA -0.000868688 -0.002959562 -0.002090874
0.293519207

0.00272599 -0.01048274 0.567189325 0.574528381
0.922085 -7.254202438 cg16044251 6 29521695 p
GABBR1 UBD -1694 IGR island IGR - island
NA -0.0114198 -0.008693809 0.00272599 1.31355533
-0.004247288 -0.002237319 -0.567074206 0.574605657
0.922085 -7.254268853 cg20806676 6 29579306 p
GABBR1/MOG GABBR1 NA Body open sea Body - open sea
NA -0.000777313 -0.005024601 -0.004247288
0.154701514
0.00285823 -0.002303248 0.566406519 0.575053961
0.922085 -7.254653799 cg17301635 18 3451475 p
DLGAP1 TGIF1 NA 5'UTR island 5'UTR - island
NA -0.003285765 -0.000427535 0.00285823
7.685373495
-0.003589664 -0.012847136 -0.565550327 0.575629086
0.922085 -7.255146775 cg10829693 21 34396221 q
OLIG2 OLIG2 -1995 IGR island IGR - island
NA -0.011613189 -0.015202854 -0.003589664
0.763882197
-0.004725266 0.005772489 -0.562401929 0.577746386
0.923659816 -7.256953264 cg24567537 6
29520527 p GABBR1 UBD -2862 IGR shore
IGR - shore NA 0.007396799 0.002671534
-0.004725266 2.76874639
0.010933158 -0.002107235 0.562080779 0.577962575
0.923659816 -7.257136978 cg04507121 2
172778341 q SLC25A12 HAT1 NA TSS1500 shore
TSS1500 - shore NA -0.005865508 0.00506765
0.010933158 -1.157441485
-0.003338364 0.000387284 -0.558254955 0.580541073
0.92523193 -7.259317626 cg18679753 6 29443446 p
GABBR1 MAS1L -11097 IGR open sea IGR - open sea
NA 0.001534847 -0.001803517 -0.003338364
-0.851029834
-0.003215591 -0.016196435 -0.55788143 0.58079312
0.92523193 -7.259529746 cg17587327 2 171670878 q
GAD1 GAD1 -2322 IGR shore IGR - shore
NA -0.015091076 -0.018306667 -0.003215591
0.82434865
0.015332929 -0.005993688 0.557611477 0.580975313
0.92523193 -7.259682962 cg14791008 12 72332386 q
TPH2 TPH2 NA TSS1500 open sea TSS1500 - open
sea NA -0.011264382 0.004068546 0.015332929
-2.768650317
0.003809311 -0.004454087 0.556290595 0.581867185
0.925574778 -7.2604316 cg07196069 9 4491175 p
SLC1A1 SLC1A1 NA Body island Body - island
NA -0.005763538 -0.001954226 0.003809311
2.949268365
-0.004334559 0.010740946 -0.555242022 0.582575669
0.925625453 -7.261024661 cg17987649 6
29526534 p GABBR1/MOG UBD NA Body open
sea Body - open sea NA 0.012230951 0.007896391
-0.004334559 1.548929117
0.003254504 0.00023206 0.553430579 0.583800589
0.926495598 -7.262046606 cg11912202 21
34350875 q OLIG2 OLIG2 -47341 IGR island

IGR - island NA -0.000886675 0.002367829
0.003254504 -0.374467696
-0.001576793 -0.000445059 -0.547663402 0.587708771
0.931617148 -7.265278401 cg00845219 22
19842449 q COMT GNB1L NA 1stExon island
1stExon - island NA 9.70E-05 -0.001479829 -
0.001576793 -0.065523106
-0.009023563 0.004890691 -0.546356754 0.588595991
0.931699433 -7.266006 cg01138652 22 19973978 q
COMT ARVCF NA Body shore Body - shore
NA 0.007992541 -0.001031022 -0.009023563
-7.752055556
0.001652975 -0.003701415 0.545581331 0.589122814
0.931699433 -7.266436983 cg19690984 6
29521631 p GABBR1 UBD -1758 IGR island
IGR - island NA -0.004269625 -0.00261665
0.001652975 1.631714268
0.002944179 -0.008368118 0.539791737 0.593063446
0.934895562 -7.269635868 cg03152033 18 4453856
p DLGAP1 DLGAP1-AS5 189254 IGR shore IGR -
shore NA -0.00938018 -0.006436001 0.002944179
1.457454665
-0.00541434 0.003289225 -0.539291702 0.593404383
0.934895562 -7.269910578 cg25562031 6
29624808 p GABBR1/MOG MOG NA 1stExon open
sea 1stExon - open sea V$HTF_01;V$CREB_02;V$XBP1_01;V$ATF_01;V$ATF6_01
0.005150404 -0.000263936 -0.00541434 -
19.51386549
-0.001763099 -0.003313821 -0.539019729 0.593589861
0.934895562 -7.270059889 cg07063032 18 3450262
p DLGAP1 TGIF1 NA 5'UTR island 5'UTR -
island NA -0.002707756 -0.004470855 -0.001763099
0.605646105
0.002765416 -0.000871749 0.5382636 0.594105665
0.934895562 -7.27047461 cg16535180 22
20008367 q COMT C22orf25 NA TSS1500 island
TSS1500 - island NA -0.001822361 0.000943055
0.002765416 -1.932400721
-0.002625782 -8.76E-05 -0.534247288 0.59684904 0.934895562
-7.27266789 cg07119172 17 28512018 q SLC6A4
CCDC55 NA Body open sea Body - open sea NA
0.000815012 -0.00181077 -0.002625782 -
0.450091146
0.007306123 0.085254182 0.534226497 0.596863257
0.934895562 -7.272679202 cg11712482 22
19928667 q COMT COMT NA TSS1500 shore
TSS1500 - shore NA 0.082742703 0.090048825
0.007306123 0.918864874
-0.001210095 -0.000674282 -0.53416339 0.596906411
0.934895562 -7.272713534 cg04920689 15
88800510 q NTRK3 NTRK3 NA TSS1500 island
TSS1500 - island NA -0.000258312 -0.001468407
-0.001210095 0.175913286
-0.003041109 0.003097616 -0.534066566 0.596972626
0.934895562 -7.272766202 cg12083232 6
29571438 p GABBR1/MOG GABBR1 NA Body open

sea Body - open sea NA 0.004142997 0.001101889
-0.003041109 3.759905677
-0.004015999 0.020477366 -0.533204581 0.597562256
0.934895562 -7.273234667 cg08829877 22
19960832 q COMT ARVCF NA Body island
Body - island NA 0.021857866 0.017841866
-0.004015999 1.225088515
0.001796964 -0.002290028 0.532597351 0.59797779
0.934895562 -7.273564233 cg00063945 18 3450342
p DLGAP1 TGIF1 NA 5'UTR island 5'UTR -
island NA -0.002907735 -0.001110771 0.001796964
2.61776238
0.001382362 -0.001856324 0.529034376 0.600418739
0.935371805 -7.275490558 cg06025631 11
27722549 p BDNF BDNF NA Body island
Body - island NA -0.002331511 -0.000949149
0.001382362 2.456422087
0.004961736 0.014204238 0.528507907 0.600779817
0.935371805 -7.275774117 cg13620631 9
87489528 q NTRK2 NTRK2 NA 3'UTR open
sea 3'UTR - open sea NA 0.012498641 0.017460377
0.004961736 0.715828808
0.001772409 0.001398421 0.527943336 0.60116714
0.935371805 -7.276077889 cg23248007 6
29716601 p MOG LOC285830 NA Body island
Body - island NA 0.000789155 0.002561564
0.001772409 0.308075602
0.001316746 -0.000388024 0.526831429 0.601930308
0.935371805 -7.276675227 cg26196213 6
29617956 p GABBR1/MOG MOG -6802 IGR island
IGR - island NA -0.000840655 0.00047609
0.001316746 -1.765748737
-0.015702951 -0.154161752 -0.525623501 0.602759899
0.935371805 -7.277322746 cg08065408 6
29520774 p GABBR1 UBD -2615 IGR shore
IGR - shore NA -0.148763863 -0.164466814
-0.015702951 0.904522067
0.003128217 -0.003593283 0.525404437 0.602910407
0.935371805 -7.277440021 cg21569150 6
29580360 p GABBR1/MOG GABBR1 NA Body open
sea Body - open sea NA -0.004668607 -0.00154039
0.003128217 3.030794585
-0.00189139 -0.00262679 -0.525174123 0.603068663
0.935371805 -7.277563266 cg08856772 2
172751016 q SLC25A12 SLC25A12 NA TSS1500 island
TSS1500 - island NA -0.001976625 -0.003868015
-0.00189139 0.511017879
-0.005546086 0.014037492 -0.522290618 0.605051674
0.93724912 -7.279101788 cg12021641 6 29555593 p
GABBR1/MOG OR2H2 NA TSS200 open sea TSS200 - open sea
NA 0.015943959 0.010397873 -0.005546086
1.53338661
-0.004612138 0.013895219 -0.520846136 0.606046206
0.93724912 -7.279869374 cg02928278 6 29574918 p
GABBR1/MOG GABBR1 NA Body open sea Body - open sea
NA 0.015480642 0.010868504 -0.004612138
1.424358073

0.005638124 -0.00370816 0.519921871 0.606682968
0.93724912 -7.280359425 cg03258475 6 29577223 p
GABBR1/MOG GABBR1 NA Body open sea Body - open sea
NA -0.005646265 -8.14E-06 0.005638124
693.5296598
-0.004714022 0.026919633 -0.519433528 0.607019533
0.93724912 -7.280618002 cg00721193 22 19966373 q
COMT ARVCF NA Body shore Body - shore
NA 0.028540079 0.023826057 -0.004714022
1.19785154
-0.003820333 -0.001401237 -0.516647641 0.608941227
0.938715626 -7.282088554 cg00542638 22
20007500 q COMT C22orf25 NA TSS1500 shore
TSS1500 - shore NA -8.80E-05 -0.00390833 -
0.003820333 0.022515291
-0.009394392 -0.079662654 -0.516067559 0.609341722
0.938715626 -7.282393777 cg12017635 12
72343656 q TPH2 TPH2 NA Body open
sea Body - open sea NA -0.076433332 -0.085827724
-0.009394392 0.890543625
-0.004074326 0.022622157 -0.510369937 0.613281923
0.940710156 -7.285373777 cg03049917 15
88752391 q NTRK3 NTRK3 NA Body open
sea Body - open sea NA 0.024022706 0.019948381
-0.004074326 1.204243426
0.004491772 -0.033934615 0.508617176 0.614496414
0.940710156 -7.286283968 cg14692377 17
28562685 q SLC6A4 SLC6A4 NA 1stExon island
1stExon - island NA -0.035478661 -0.030986889
0.004491772 1.144957167
0.007152408 0.050653151 0.508551128 0.6145422
0.940710156 -7.286318206 cg13763482 9 4663107
p SLC1A1 C9orf68 NA Body shore Body -
shore NA 0.04819451 0.055346919 0.007152408
0.870771335
-0.004078104 -0.029968577 -0.508294652 0.614720011
0.940710156 -7.286451116 cg00359010 6
29635692 p GABBR1/MOG MOG NA 3'UTR open
sea 3'UTR - open sea NA -0.028566729 -0.032644832
-0.004078104 0.875076597
0.00562303 -0.016417572 0.507996464 0.614926771
0.940710156 -7.286605559 cg16713743 21
34397135 q OLIG2 OLIG2 NA TSS1500 island
TSS1500 - island NA -0.018350488 -0.012727458
0.00562303 1.441803082
-0.005865646 -0.023107327 -0.507111927 0.615540287
0.940710156 -7.28706317 cg22113926 17
28566331 q SLC6A4 BLMH -8882 IGR shelf
IGR - shelf NA -0.021091011 -0.026956657
-0.005865646 0.782404545
-0.003490002 -0.029599053 -0.504245663 0.617530262
0.940710156 -7.288540626 cg22043168 11
27741077 p BDNF BDNF NA Body island
Body - island NA -0.028399365 -0.031889366
-0.003490002 0.890559075
0.001286428 -0.00117863 0.503352518 0.618150952
0.940710156 -7.288999325 cg26151531 22

19842652 q COMT GNB1L NA TSS200 island
TSS200 - island NA -0.001620839 -0.000334412
0.001286428 4.846839899
0.002329341 -0.006660139 0.501710145 0.619293063
0.940710156 -7.289840723 cg06879567 18 3594243
p DLGAP1 DLGAP1 NA Body open sea Body -
open sea V$HTF_01;V$RFX1_01 -0.00746085 -0.005131509
0.002329341 1.453929174
0.00298799 0.000766567 0.501636269 0.61934446 0.940710156
-7.289878507 cg04408897 6 29596901 p
GABBR1/MOG GABBR1 NA TSS1500 shore TSS1500 - shore
NA -0.000260555 0.002727435 0.00298799 -
0.09553108
0.001641102 -0.00128512 0.501525128 0.619421785
0.940710156 -7.289935339 cg09492354 11
27720709 p BDNF BDNF NA Body shore
Body - shore NA -0.001849249 -0.000208147
0.001641102 8.884361084
0.011703889 0.021328975 0.499477866 0.620846941
0.940710156 -7.290979993 cg06591579 6
29461269 p GABBR1 MAS1L 6726 IGR open
sea IGR - open sea NA 0.017305763 0.029009652
0.011703889 0.5965519
-0.005579714 -0.0201757 -0.499187648 0.62104909 0.940710156
-7.291127741 cg13819127 6 29717368 p MOG
LOC285830 NA TSS1500 shore TSS1500 - shore NA
-0.018257673 -0.023837387 -0.005579714
0.765925942
0.002972095 -0.009653024 0.498865869 0.621273258
0.940710156 -7.291291459 cg20664238 15
88798877 q NTRK3 NTRK3 NA Body shore
Body - shore NA -0.010674681 -0.007702587
0.002972095 1.385856728
0.00273705 -0.008784936 0.49786464 0.621971003 0.940710156
-7.291800208 cg25328597 11 27722638 p BDNF
BDNF NA TSS200 island TSS200 - island NA
-0.009725797 -0.006988747 0.00273705 1.391636756
0.004863441 -0.008557138 0.497626497 0.622137014
0.940710156 -7.291921067 cg08698936 6
29689809 p GABBR1/MOG HLA-F NA TSS1500 shore
TSS1500 - shore NA -0.010228946 -0.005365505
0.004863441 1.906427466
-0.006278635 0.034047448 -0.496999606 0.622574121
0.940710156 -7.292238943 cg13666340 9 4666266
p SLC1A1 C9orf68 NA 5'UTR shelf 5'UTR -
shelf NA 0.036205728 0.029927094 -0.006278635
1.209797668
-0.006538869 0.022048665 -0.494774896 0.62412645
0.940710156 -7.293363838 cg06061002 6
29638918 p GABBR1/MOG MOG NA 3'UTR open
sea 3'UTR - open sea NA 0.024296401 0.017757532
-0.006538869 1.368230717
-0.00460577 0.00260692 -0.494415091 0.624377674
0.940710156 -7.293545301 cg09721630 6
29523835 p GABBR1 UBD NA Body shelf
Body - shelf NA 0.004190153 -0.000415617
-0.00460577 -10.08177262

-0.001324232 -0.001530905 -0.493648436 0.624913124
0.940710156 -7.293931522 cg12312205 18 3594173
p DLGAP1 DLGAP1 NA Body open sea Body -
open sea NA -0.0010757 -0.002399932 -0.001324232
0.448221025
0.002366351 -0.001590487 0.491464109 0.626439845
0.940710156 -7.295028691 cg07412232 6
29495773 p GABBR1 LINC01015 -1410 IGR open
sea IGR - open sea NA -0.00240392 -3.76E-05
0.002366351 63.98602296
-0.005103718 -0.022586774 -0.491452349 0.626448069
0.940710156 -7.295034585 cg13682912 6
29627142 p GABBR1/MOG MOG NA Body open
sea Body - open sea NA -0.020832371 -0.025936089
-0.005103718 0.80321945
0.002663745 -0.008163223 0.491446083 0.62645245
0.940710156 -7.295037725 cg06362065 18 3594098
p DLGAP1 DLGAP1 NA Body open sea Body -
open sea NA -0.009078885 -0.00641514 0.002663745
1.415227958
-0.001699825 -0.001355855 -0.488199817 0.628724526
0.943086789 -7.296659383 cg00414306 2
172779015 q SLC25A12 HAT1 NA 1stExon island
1stExon - island NA -0.000771541 -0.002471366
-0.001699825 0.312191966
0.006908843 0.089896252 0.486953091 0.629598096
0.943276728 -7.297279366 cg10601943 6
29692824 p GABBR1/MOG HLA-F NA Body shore
Body - shore NA 0.087521337 0.09443018
0.006908843 0.926836494
0.004483285 -0.000433485 0.486051443 0.630230212
0.943276728 -7.297726772 cg18868933 6
29708164 p MOG LOC285830 NA Body open
sea Body - open sea NA -0.001974614 0.002508671
0.004483285 -0.787115496
-0.006764536 -0.041053116 -0.483260072 0.632188946
0.945174293 -7.299106695 cg20893956 6
152126736 q ESR1 ESR1 NA 5'UTR shelf
5'UTR - shelf NA -0.038727807 -0.045492343
-0.006764536 0.851303865
0.002126836 0.002093991 0.480232446 0.634316521
0.94731987 -7.300594556 cg11854392 22 19879320 q
COMT TXNRD2 NA Body open sea Body - open sea
NA 0.001362891 0.003489728 0.002126836
0.390543778
-0.015734898 -0.097494456 -0.476821221 0.636717457
0.94835782 -7.302259885 cg11935738 6 29520752 p
GABBR1 UBD -2637 IGR shore IGR - shore
NA -0.092085585 -0.107820483 -0.015734898
0.854063924
0.00625778 -0.010150765 0.476223481 0.637138579
0.94835782 -7.302550491 cg21446343 22 19974866 q
COMT ARVCF NA Body shore Body - shore
NA -0.012301877 -0.006044097 0.00625778
2.035354067
-0.004253051 0.002810936 -0.476166988 0.637178386
0.94835782 -7.302577938 cg09444802 6 29691312 p

GABBR1/MOG HLA-F NA Body island Body - island
NA 0.004272922 1.99E-05 -0.004253051
215.0262021
-0.002150126 0.002132611 -0.474931584 0.638049171
0.94835782 -7.303177352 cg00118317 21 34392029 q
OLIG2 OLIG2 -6187 IGR shore IGR - shore
NA 0.002871716 0.00072159 -0.002150126
3.979704414
-0.008472312 0.014326692 -0.473844388 0.638815923
0.94835782 -7.303703585 cg05255330 18 3498963 p
DLGAP1 DLGAP1 NA 3'UTR island 3'UTR - island
NA 0.017239049 0.008766737 -0.008472312
1.966415634
0.001229826 -0.001279463 0.471382861 0.640553425
0.94835782 -7.304890637 cg07252731 15 88799999 q
NTRK3 NTRK3 NA TSS1500 island TSS1500 - island
NA -0.001702216 -0.00047239 0.001229826
3.603412479
-0.005271118 -0.06669921 -0.470642495 0.641076427
0.94835782 -7.305246481 cg24900983 6 152128528 q
ESR1 ESR1 NA TSS1500 shore TSS1500 - shore
NA -0.064887263 -0.070158381 -0.005271118
0.924868301
0.001460542 -0.001423871 0.468100294 0.642873682
0.94835782 -7.306464143 cg10969051 6 29617771 p
GABBR1/MOG MOG -6987 IGR island IGR - island
NA -0.001925932 -0.00046539 0.001460542
4.138314561
-0.004132803 0.008508924 -0.466862665 0.64374944
0.94835782 -7.307054587 cg13175282 22 19938541 q
COMT COMT NA 5'UTR open sea 5'UTR - open sea
NA 0.009929575 0.005796772 -0.004132803
1.712949072
-0.002900971 -0.003447222 -0.466373525 0.644095703
0.94835782 -7.30728752 cg13965062 9 87284706 q
NTRK2 NTRK2 NA 5'UTR island 5'UTR - island
NA -0.002450013 -0.005350984 -0.002900971
0.457862199
-0.002626692 -0.00241804 -0.46553134 0.644692077
0.94835782 -7.307688011 cg24952408 6 29696264 p
MOG LOC285830 NA Body open sea Body - open sea
NA -0.001515115 -0.004141807 -0.002626692
0.365810146
-0.003568808 -0.000577505 -0.462415435 0.646900613
0.94835782 -7.309163532 cg26569800 6 29578313 p
GABBR1/MOG GABBR1 NA Body open sea Body - open sea
NA 0.000649273 -0.002919535 -0.003568808
-0.222388999
0.001445097 0.001568645 0.462389792 0.646918802
0.94835782 -7.309175635 cg21241839 6 29716536 p
MOG LOC285830 NA Body island Body - island
NA 0.001071893 0.002516989 0.001445097
0.425863008
-0.010579018 -0.152095316 -0.461880371 0.647280192
0.94835782 -7.309415926 cg05951817 17 28562142 q
SLC6A4 SLC6A4 NA 5'UTR shore 5'UTR - shore

NA -0.148458778 -0.159037796 -0.010579018
0.933481109
0.003486089 -0.023827295 0.461549445 0.647515002
0.94835782 -7.309571881 cg27546977 6 29521152 p
GABBR1 UBD -2237 IGR island IGR - island
NA -0.025025638 -0.021539549 0.003486089
1.161845968
0.002300368 0.001347677 0.461130397 0.647812392
0.94835782 -7.309769208 cg14128584 6 29600583 p
GABBR1/MOG GABBR1 NA 5'UTR island 5'UTR - island
NA 0.000556925 0.002857293 0.002300368
0.194913574
-0.002442625 -0.005856836 -0.45919041 0.649189928
0.94835782 -7.310680429 cg23912231 6 29691408 p
GABBR1/MOG HLA-F NA Body island Body - island
NA -0.005017183 -0.007459808 -0.002442625
0.672561972
-0.004339635 -0.001872838 -0.45909916 0.649254753
0.94835782 -7.310723197 cg01024792 6 29524067 p
GABBR1 UBD NA Body shelf Body - shelf
NA -0.000381089 -0.004720724 -0.004339635
0.08072676
-0.005637946 -0.021765166 -0.457294819 0.650537155
0.94835782 -7.311567135 cg23325570 6 29711975 p
MOG LOC285830 NA Body open sea Body - open sea
NA -0.019827122 -0.025465068 -0.005637946
0.778600773
-0.001913725 -0.002727969 -0.456060917 0.651414751
0.94835782 -7.312142376 cg19868007 6 29691890 p
GABBR1/MOG HLA-F NA Body island Body - island
NA -0.002070126 -0.003983852 -0.001913725
0.519629365
-0.002280957 0.006787438 -0.454133847 0.652786368
0.94835782 -7.3130377 cg20294320 6 29617586 p
GABBR1/MOG MOG -7172 IGR shore IGR - shore
NA 0.007571516 0.00529056 -0.002280957
1.431137197
-0.002261413 0.00128637 -0.452709087 0.653801249
0.94835782 -7.313697242 cg18699287 6 29721001 p
MOG IFITM4P 2417 IGR island IGR - island
NA 0.002063731 -0.000197682 -0.002261413
-10.43962981
-0.006517745 -0.068010754 -0.452087771 0.654244034
0.94835782 -7.313984218 cg18458352 6 29711249 p
MOG LOC285830 NA Body open sea Body - open sea
NA -0.065770279 -0.072288024 -0.006517745
0.909836442
0.002134047 -0.00349814 0.452041074 0.654277318
0.94835782 -7.314005771 cg25197880 6 29695413 p
GABBR1/MOG LOC285830 NA Body shelf Body - shelf
NA -0.004231719 -0.002097672 0.002134047
2.017340324
-0.003081418 -0.016318153 -0.451726377 0.654501642
0.94835782 -7.31415096 cg10022526 11 27744557 p
BDNF BDNF NA TSS1500 island TSS1500 - island
NA -0.015258916 -0.018340334 -0.003081418
0.831986819

0.003300708 -0.013652097 0.451386302 0.654744093
0.94835782 -7.314307747 cg11718030 11 27744363 p
BDNF BDNF NA TSS1500 island TSS1500 - island
NA -0.014786716 -0.011486007 0.003300708
1.287367753
-0.001064305 -0.003223392 -0.450034993 0.655707864
0.94835782 -7.314929595 cg25990314 22 19841927 q
COMT GNB1L NA 5'UTR island 5'UTR - island
NA -0.002857537 -0.003921842 -0.001064305
0.7286211
-0.005155172 -0.045023867 -0.450023052 0.655716383
0.94835782 -7.314935082 cg01089249 2 171676553 q
GAD1 GAD1 NA Body island Body - island
NA -0.043251777 -0.048406949 -0.005155172
0.893503481
0.001136024 0.000142828 0.449532473 0.656066421
0.94835782 -7.315160376 cg27220153 6 29720768 p
MOG IFITM4P 2184 IGR island IGR - island
NA -0.00024768 0.000888344 0.001136024
-0.278810779
0.00601938 0.025813126 0.448876896 0.656534312
0.94835782 -7.315461065 cg19786983 15 88336651 q
NTRK3 NTRK3-AS1 -83337 IGR open sea IGR - open sea
NA 0.023743964 0.029763344 0.00601938
0.797758602
0.003151804 0.056694108 0.446924588 0.657928525
0.94835782 -7.316353952 cg18055585 15 88668892 q
NTRK3 NTRK3 NA Body open sea Body - open sea
NA 0.055610675 0.05876248 0.003151804
0.946363658
0.001460605 -0.001094184 0.446680574 0.658102872
0.94835782 -7.316465281 cg04207032 22 20004746 q
COMT ARVCF NA TSS1500 shore TSS1500 - shore
NA -0.001596267 -0.000135661 0.001460605
11.76654838
0.005781588 -0.019241229 0.446335587 0.658349397
0.94835782 -7.316622576 cg26987604 6 29696647 p
MOG LOC285830 NA Body open sea Body - open sea
NA -0.02122865 -0.015447062 0.005781588
1.374283981
0.001576592 0.000206651 0.446010384 0.65858182
0.94835782 -7.31677074 cg25381667 11 27743651 p
BDNF BDNF NA TSS200 island TSS200 - island
NA -0.000335303 0.00124129 0.001576592 -
0.270124604
0.001453775 -0.00366307 0.443458174 0.660407086
0.94853867 -7.31792984 cg07855056 17 28443892 q
SLC6A4 MIR423 NA TSS1500 island TSS1500 - island
NA -0.004162805 -0.002709031 0.001453775
1.536640096
0.00121608 0.002599975 0.442896253 0.660809241
0.94853867 -7.318184157 cg17111795 17 28618907 q
SLC6A4 BLMH NA 1stExon island 1stExon - island
NA 0.002181948 0.003398028 0.00121608
0.642121878
0.001571617 0.004250348 0.442115881 0.661367906
0.94853867 -7.318536814 cg11576659 6 29691135 p

GABBR1/MOG HLA-F NA 1stExon island 1stExon - island
NA 0.003710105 0.005281722 0.001571617
0.702442269
0.005429236 -0.003708475 0.441004964 0.662163548
0.94853867 -7.319037786 cg15893431 21 34442183 q
OLIG2 OLIG1 NA TSS1500 shore TSS1500 - shore
NA -0.005574775 -0.000145538 0.005429236
38.30447095
0.001169177 -0.003131429 0.440095077 0.662815509
0.94853867 -7.319447176 cg03670115 2 172544069 q
SLC25A12 DYNC1I2 NA 5'UTR island 5'UTR - island
NA -0.003533333 -0.002364156 0.001169177
1.494543278
0.002780637 0.00934361 0.4400223 0.662867667 0.94853867 -
7.319479884 cg25736198 12 72345225 q TPH2 TPH2
NA Body open sea Body - open sea NA
0.008387766 0.011168403 0.002780637
0.751026446
-0.005545024 0.041236988 -0.434367452 0.666925651
0.952518221 -7.322005048 cg01066157 6
152451023 q ESR1 SYNE1 NA Body open
sea Body - open sea NA 0.04314309 0.037598066 -
0.005545024 1.147481641
0.001539692 -0.003662338 0.431995972 0.668630503
0.952518221 -7.323054422 cg27034819 15
88799526 q NTRK3 NTRK3 NA 5'UTR island
5'UTR - island V$CDPCR1_01 -0.004191607 -
0.002651915 0.001539692 1.580596292
0.003030756 -0.015635054 0.431818367 0.668758255
0.952518221 -7.323132783 cg06816235 11
27742219 p BDNF BDNF NA Body island
Body - island NA -0.016676876 -0.01364612
0.003030756 1.222096525
-0.002961019 0.004076495 -0.429674764 0.670300949
0.952518221 -7.324076052 cg11116776 6
29691329 p GABBR1/MOG HLA-F NA Body island
Body - island NA 0.005094346 0.002133326
-0.002961019 2.387982274
0.001397181 0.000973972 0.428296117 0.671293895
0.952518221 -7.324680258 cg18117895 11
27722066 p BDNF BDNF NA Body island
Body - island NA 0.000493691 0.001890873
0.001397181 0.261091766
-0.005216134 0.058030096 -0.426611142 0.672508285
0.952518221 -7.325416109 cg03037684 6
152421333 q ESR1 ESR1 NA 3'UTR open
sea 3'UTR - open sea NA 0.059823142 0.054607008
-0.005216134 1.095521331
-0.001208928 -0.000299426 -0.426523315 0.672571608
0.952518221 -7.325454386 cg09545452 2
172779111 q SLC25A12 HAT1 NA Body island
Body - island NA 0.000116143 -0.001092785
-0.001208928 -0.106281622
-0.001438783 -0.002134766 -0.426216557 0.6727928
0.952518221 -7.325588015 cg12941622 2
172778750 q SLC25A12 HAT1 NA TSS200 island

TSS200 - island NA -0.001640184 -0.003078967
-0.001438783 0.532706031
-0.006683838 -0.011128226 -0.424988054 0.67367892
0.952518221 -7.326122221 cg15922678 6
29694484 p GABBR1/MOG LOC285830 NA Body shelf
Body - shelf NA -0.008830656 -0.015514494
-0.006683838 0.569187497
-0.002188475 0.001794066 -0.422530806 0.675452757
0.952518221 -7.327186164 cg00528793 22
19842837 q COMT GNB1L NA TSS1500 island
TSS1500 - island NA 0.002546354 0.000357879
-0.002188475 7.115131009
0.002663188 -0.00837202 0.42145033 0.676233329
0.952518221 -7.327652058 cg04352676 6
29596516 p GABBR1/MOG GABBR1 NA TSS1500 shore
TSS1500 - shore NA -0.009287491 -0.006624303
0.002663188 1.402032997
0.004385265 -0.014014908 0.421401538 0.676268587
0.952518221 -7.327673069 cg17264271 6
152126938 q ESR1 ESR1 NA 5'UTR shore
5'UTR - shore NA -0.015522343 -0.011137078
0.004385265 1.39375364
0.001111752 -0.00268257 0.420335444 0.677039143
0.952518221 -7.328131554 cg19760323 6
29716554 p MOG LOC285830 NA Body island
Body - island NA -0.003064735 -0.001952982
0.001111752 1.569258676
-0.004285851 0.099766091 -0.420290196 0.677071855
0.952518221 -7.328150988 cg08805037 22
20009275 q COMT C22orf25 NA 5'UTR shore
5'UTR - shore NA 0.101239352 0.096953501
-0.004285851 1.044205223
0.003197252 -0.009058638 0.418997277 0.67800685
0.952518221 -7.32870542 cg10122187 22
19938620 q COMT COMT NA 5'UTR open
sea 5'UTR - open sea NA -0.010157694 -0.006960442
0.003197252 1.459346145
0.003198886 -0.024825894 0.418405726 0.678434812
0.952518221 -7.328958527 cg14293300 21
34399361 q OLIG2 OLIG2 NA Body island
Body - island NA -0.025925511 -0.022726625
0.003198886 1.140754978
0.001282524 0.000874415 0.416453167 0.679848179
0.952518221 -7.329791457 cg25638186 6
29720443 p MOG IFITM4P 1859 IGR island
IGR - island NA 0.000433547 0.001716072
0.001282524 0.252639325
-0.002763858 0.006449414 -0.416050728 0.680139633
0.952518221 -7.329962653 cg06309882 6
29443400 p GABBR1 MAS1L -11143 IGR open
sea IGR - open sea NA 0.00739949 0.004635632 -
0.002763858 1.596220213
-0.002590795 -0.009213773 -0.415329508 0.680662078
0.952518221 -7.330269046 cg27193031 11
27721088 p BDNF BDNF NA Body shore
Body - shore NA -0.008323187 -0.010913982
-0.002590795 0.762616891

-0.001621249 -0.001949607 -0.414737883 0.681090766
0.952518221 -7.330519991 cg17810098 22
19929066 q COMT TXNRD2 NA Body shore
Body - shore NA -0.001392303 -0.003013551
-0.001621249 0.462013927
0.001779685 -0.006045393 0.413644795 0.681883095
0.952518221 -7.330982706 cg24438313 6
29717010 p MOG LOC285830 NA TSS200 island
TSS200 - island NA -0.00665716 -0.004877475
0.001779685 1.364878372
-0.001187789 0.001286781 -0.411846927 0.683187085
0.952518221 -7.331741134 cg13344806 18 3450921
p DLGAP1 TGIF1 NA 5'UTR island 5'UTR -
island NA 0.001695083 0.000507294 -0.001187789
3.341419597
0.002526071 -0.025432004 0.411180687 0.68367056
0.952518221 -7.332021357 cg13723118 9
87284722 q NTRK2 NTRK2 NA 5'UTR island
5'UTR - island NA -0.026300341 -0.02377427
0.002526071 1.106252306
0.005221892 -0.076079746 0.409997139 0.684529767
0.952518221 -7.332518053 cg03363743 17
28562474 q SLC6A4 SLC6A4 NA 5'UTR island
5'UTR - island NA -0.077874772 -0.07265288
0.005221892 1.071874536
-0.00154232 -0.002836174 -0.40984827 0.68463787
0.952518221 -7.332580429 cg17514757 6
29720651 p MOG IFITM4P 2067 IGR island
IGR - island NA -0.002306001 -0.003848321
-0.00154232 0.599222625
0.00166798 -0.006424842 0.408654315 0.685505121
0.952518221 -7.333079877 cg27079446 18 3449844
p DLGAP1 TGIF1 NA 5'UTR island 5'UTR -
island NA -0.006998211 -0.00533023 0.00166798
1.3129284
-0.005009464 -0.003321184 -0.406230385 0.687267119
0.952518221 -7.334089407 cg03861097 21
34393589 q OLIG2 OLIG2 -4627 IGR shore
IGR - shore NA -0.001599181 -0.006608645
-0.005009464 0.241983174
-0.001439156 -0.005953386 -0.405987696 0.687443632
0.952518221 -7.334190155 cg04378940 6
29617766 p GABBR1/MOG MOG -6992 IGR island
IGR - island NA -0.005458676 -0.006897832
-0.001439156 0.791361098
0.001263804 -0.000479161 0.404522196 0.688509902
0.952518221 -7.33479727 cg00060933 6
29617602 p GABBR1/MOG MOG -7156 IGR shore
IGR - shore NA -0.000913594 0.00035021
0.001263804 -2.608704063
-0.009167154 0.026724956 -0.404221358 0.688728866
0.952518221 -7.33492163 cg11747594 6
29648225 p GABBR1/MOG ZFP57 8056 IGR open
sea IGR - open sea NA 0.029876165 0.020709012
-0.009167154 1.44266496
-0.001314863 -0.001037874 -0.403454451 0.689287182
0.952518221 -7.335238238 cg00920970 6

152129388 q ESR1 ESR1 NA Body island
Body - island V$AP2_Q6;V$HEN1_02;V$E47_01 -0.00058589
-0.001900753 -0.001314863 0.308240819
0.002285319 -0.021819067 0.401824772 0.690474191
0.952518221 -7.335909055 cg25962210 11
27721222 p BDNF BDNF NA Body shore
Body - shore NA -0.022604646 -0.020319327
0.002285319 1.112470207
0.001155905 -0.002850127 0.401795448 0.690495557
0.952518221 -7.335921101 cg18094551 22
20004364 q COMT ARVCF NA TSS200 island
TSS200 - island NA -0.003247469 -0.002091564
0.001155905 1.552651301
0.01547114 -0.065222559 0.401384368 0.690795106
0.952518221 -7.336089875 cg18280909 6
29723301 p MOG IFITM4P 4717 IGR shelf
IGR - shelf NA -0.070540764 -0.055069624
0.01547114 1.280937824
-0.002048278 -0.003218652 -0.400440633 0.691482984
0.952518221 -7.33647669 cg14665414 6
29596574 p GABBR1/MOG GABBR1 NA TSS1500 shore
TSS1500 - shore NA -0.002514557 -0.004562835
-0.002048278 0.551095282
-0.00552582 -0.004923566 -0.400439572 0.691483757
0.952518221 -7.336477125 cg02226939 17
28619481 q SLC6A4 BLMH NA TSS1500 shore
TSS1500 - shore NA -0.003024065 -0.008549885
-0.00552582 0.353696611
0.003624916 -0.0004765 0.399583532 0.692107947
0.952518221 -7.336827215 cg17728820 6
29709366 p MOG LOC285830 NA Body open
sea Body - open sea NA -0.001722565 0.001902351
0.003624916 -0.905492705
-0.001640171 -0.001705731 -0.398103721 0.69318748
0.952518221 -7.337430657 cg15136600 6
29571468 p GABBR1/MOG GABBR1 NA Body open
sea Body - open sea NA -0.001141922 -0.002782093
-0.001640171 0.41045441
0.007178595 0.006812535 0.397563667 0.693581617
0.952518221 -7.33765033 cg25437807 6
29589545 p GABBR1/MOG GABBR1 NA Body open
sea Body - open sea NA 0.004344893 0.011523488
0.007178595 0.377046669
-0.00301904 -0.005296945 -0.397536889 0.693601161
0.952518221 -7.337661214 cg13663738 21
34392756 q OLIG2 OLIG2 -5460 IGR shore
IGR - shore NA -0.00425915 -0.00727819
-0.00301904 0.585193609
-0.001823841 -0.007287856 -0.396721523 0.694196394
0.952518221 -7.337992291 cg04991728 6
29720954 p MOG IFITM4P 2370 IGR island
IGR - island NA -0.006660911 -0.008484752
-0.001823841 0.785044872
-0.002745024 -0.001349777 -0.394808117 0.695593992
0.95309066 -7.338766581 cg02035039 6 29691888 p
GABBR1/MOG HLA-F NA Body island Body - island

NA -0.000406175 -0.003151199 -0.002745024
0.128895292
-0.003803624 -0.061726145 -0.394242971 0.696006995
0.95309066 -7.338994567 cg21829923 2 171573891 q
GAD1 SP5 NA Body island Body - island
V$PAX4_01 -0.060418649 -0.064222273 -0.003803624
0.940774074
-0.001158497 -0.002268703 -0.391059625 0.698335118
0.953381907 -7.340272722 cg14337085 12
72233324 q TPH2 TBC1D15 NA TSS200 island
TSS200 - island NA -0.00187047 -0.003028967
-0.001158497 0.617527257
-0.002848537 0.007400231 -0.390899975 0.698451956
0.953381907 -7.340336553 cg00162046 22
20006956 q COMT TANGO2 -1675 IGR shore
IGR - shore NA 0.008379416 0.005530879
-0.002848537 1.515024348
0.003633208 0.027896972 0.390390182 0.698825091
0.953381907 -7.340540206 cg26646411 22
19841374 q COMT GNB1L NA 5'UTR shore
5'UTR - shore NA 0.026648057 0.030281264
0.003633208 0.880017964
-0.004129319 -0.00132022 -0.390141212 0.699007348
0.953381907 -7.340639569 cg10477592 21
34406589 q OLIG2 OLIG2 8373 IGR shore
IGR - shore NA 9.92E-05 -0.004030085 -
0.004129319 -0.024623222
-0.002430393 -0.007849895 -0.38714899 0.701199211
0.953907388 -7.341828843 cg10558494 11
27721280 p BDNF BDNF NA Body shore
Body - shore NA -0.007014447 -0.009444841
-0.002430393 0.742675024
-0.005670014 0.005329365 -0.38697417 0.701327351
0.953907388 -7.341898045 cg09517033 6
29461584 p GABBR1 MAS1L 7041 IGR open
sea IGR - open sea NA 0.007278432 0.001608418
-0.005670014 4.525210951
-0.004708597 0.042065561 -0.386173946 0.701914014
0.953907388 -7.342214419 cg01393604 6
29695590 p GABBR1/MOG LOC285830 NA Body shelf
Body - shelf NA 0.043684141 0.038975544
-0.004708597 1.120809009
0.002839256 0.007258255 0.384708023 0.7029892
0.953907388 -7.342792299 cg18126978 6
29588988 p GABBR1/MOG GABBR1 NA Body open
sea Body - open sea NA 0.006282261 0.009121517
0.002839256 0.688729797
0.001390966 -0.004416063 0.382446548 0.704649107
0.953907388 -7.343679521 cg22617773 6
29521751 p GABBR1 UBD -1638 IGR island
IGR - island NA -0.004894208 -0.003503242
0.001390966 1.397051023
-0.005699842 -0.018925253 -0.38244154 0.704652784
0.953907388 -7.34368148 cg22546130 22
19950026 q COMT COMT NA 5'UTR open
sea 5'UTR - open sea NA -0.016965933 -0.022665775
-0.005699842 0.748526503

-0.001057946 0.000109884 -0.379586693 0.70675033
0.953907388 -7.344794078 cg10687604 6
29720687 p MOG IFITM4P 2103 IGR island
IGR - island NA 0.000473553 -0.000584393
-0.001057946 -0.810333439
-0.001755528 -0.003924088 -0.37937299 0.706907438
0.953907388 -7.34487703 cg21834061 6
29521162 p GABBR1 UBD -2227 IGR island
IGR - island NA -0.003320625 -0.005076153
-0.001755528 0.654161778
0.00382962 0.008914893 0.379352834 0.706922256
0.953907388 -7.344884851 cg05115679 6
29593490 p GABBR1/MOG GABBR1 NA Body shore
Body - shore NA 0.007598462 0.011428082
0.00382962 0.664893877
0.006841101 -0.058767777 0.378105749 0.707839346
0.953907388 -7.34536798 cg05279622 6
29629758 p GABBR1/MOG MOG NA Body open
sea Body - open sea NA -0.061119405 -0.054278304
0.006841101 1.126037485
-0.003033154 0.013408147 -0.377123535 0.708561965
0.953907388 -7.345747386 cg25693099 18 3879303
p DLGAP1 DLGAP1 NA 1stExon island 1stExon
- island NA 0.014450794 0.01141764 -0.003033154
1.26565509
0.001138608 -0.003079966 0.376791357 0.708806413
0.953907388 -7.345875477 cg20563534 22
20004369 q COMT ARVCF NA TSS200 island
TSS200 - island NA -0.003471362 -0.002332754
0.001138608 1.488096297
-0.002554918 -0.013930195 -0.376766299 0.708824854
0.953907388 -7.345885135 cg17546147 6
29710109 p MOG LOC285830 NA Body open
sea Body - open sea NA -0.013051942 -0.01560686
-0.002554918 0.836295185
-0.000983138 0.000424569 -0.374510553 0.710485685
0.953907388 -7.346751955 cg26770917 21
34444339 q OLIG2 OLIG1 NA 1stExon island
1stExon - island V$CART1_01 0.000762522 -0.000220616
-0.000983138 -3.45633782
0.003029151 -0.010103991 0.374320416 0.710625743
0.953907388 -7.346824784 cg00782607 2
171672156 q GAD1 GAD1 NA TSS1500 shore
TSS1500 - shore NA -0.011145262 -0.00811611
0.003029151 1.373226984
0.00657741 -0.076650189 0.37381718 0.710996483 0.953907388
-7.347017363 cg20709110 22 19946873 q COMT
COMT NA 5'UTR open sea 5'UTR - open sea NA
-0.078911174 -0.072333764 0.00657741 1.090931396
-0.003511947 0.028215802 -0.373477545 0.711246737
0.953907388 -7.347147189 cg02547724 6
29455365 p GABBR1 MAS1L NA 1stExon open
sea 1stExon - open sea NA 0.029423034 0.025911087
-0.003511947 1.135538369
0.001008281 -0.001199563 0.370587795 0.71337731
0.95490594 -7.348247073 cg15490715 6 29521568 p
GABBR1 UBD -1821 IGR island IGR - island

NA -0.001546159 -0.000537879 0.001008281
2.874550371
0.001625145 -0.001882354 0.370574209 0.713387332
0.95490594 -7.348252224 cg11032634 22 19929254 q
COMT TXNRD2 NA 1stExon island 1stExon - island
NA -0.002440998 -0.000815852 0.001625145
2.99195979
-0.004191526 -0.084109265 -0.367474279 0.715675486
0.957032322 -7.349422646 cg04188862 15
88801474 q NTRK3 NTRK3-AS1 5513 IGR shore
IGR - shore NA -0.082668427 -0.086859954
-0.004191526 0.951743859
0.005400884 0.033138803 0.365834822 0.716886704
0.957715831 -7.350037704 cg27368718 18 3883519
p DLGAP1 DLGAP1-AS3 5339 IGR shelf IGR -
shelf NA 0.031282249 0.036683133 0.005400884
0.852769276
0.002094239 -0.016138478 0.364045064 0.718209815
0.958547343 -7.350706036 cg23164938 6
152128366 q ESR1 ESR1 NA TSS1500 shore
TSS1500 - shore NA -0.016858373 -0.014764133
0.002094239 1.141846419
-0.0028218 -0.037066678 -0.362197623 0.719576497
0.959118176 -7.351392497 cg18183163 2
171574141 q GAD1 SP5 NA 3'UTR shore
3'UTR - shore NA -0.036096684 -0.038918484
-0.0028218 0.927494595
0.011251082 -0.059290821 0.36157171 0.720039742
0.959118176 -7.351624285 cg05813221 6
29629988 p GABBR1/MOG MOG NA Body open
sea Body - open sea NA -0.06315838 -0.051907299
0.011251082 1.216753363
-0.002640921 0.02249658 -0.359999332 0.721203951
0.959426728 -7.352204813 cg01558785 6
29638162 p GABBR1/MOG MOG NA 3'UTR open
sea 3'UTR - open sea NA 0.023404397 0.020763476
-0.002640921 1.127190706
-0.002845452 -0.016591675 -0.359364675 0.721674052
0.959426728 -7.352438419 cg10131972 22
19879787 q COMT TXNRD2 NA Body open
sea Body - open sea NA -0.015613551 -0.018459003
-0.002845452 0.845850167
-0.005015028 -0.01113377 -0.356973879 0.723445939
0.959838127 -7.35331476 cg12508693 11
27657625 p BDNF BDNFOS NA Body open
sea Body - open sea NA -0.009409854 -0.014424882
-0.005015028 0.652334903
-0.001746306 -0.003445884 -0.356098631 0.724094999
0.959838127 -7.35363413 cg20253551 6
152129400 q ESR1 ESR1 NA Body island
Body - island V$HEN1_02;V$E47_01 -0.002845592 -
0.004591898 -0.001746306 0.619698438
0.009741994 0.207282178 0.355353968 0.724647386
0.959838127 -7.353905237 cg13638427 6
29617320 p GABBR1/MOG MOG -7438 IGR shore
IGR - shore NA 0.203933368 0.213675362
0.009741994 0.954407501

-0.003103084 -0.001860908 -0.35516168 0.724790048
0.959838127 -7.353975152 cg21864378 6
29719180 p MOG IFITM4P NA TSS1500 shore
TSS1500 - shore NA -0.000794223 -0.003897307
-0.003103084 0.203787621
0.002472483 -0.020444139 0.351319514 0.727642718
0.962683983 -7.355364261 cg08470639 9
87285186 q NTRK2 NTRK2 NA 5'UTR island
5'UTR - island NA -0.021294055 -0.018821572
0.002472483 1.131364328
-0.004580626 -0.009864887 -0.35020285 0.728472545
0.962850668 -7.355765171 cg06877423 6
152200760 q ESR1 ESR1 NA Body open
sea Body - open sea NA -0.008290297 -0.012870923
-0.004580626 0.644110536
-0.002660336 0.008655615 -0.347261831 0.730659698
0.964006123 -7.35681501 cg00145961 15
88576296 q NTRK3 NTRK3 NA Body open
sea Body - open sea NA 0.009570106 0.00690977 -
0.002660336 1.38501087
-0.0020264 -0.006085614 -0.347132268 0.730756103
0.964006123 -7.356861057 cg26813908 17
28443598 q SLC6A4 CCDC55 NA TSS1500 shore
TSS1500 - shore NA -0.005389039 -0.007415439
-0.0020264 0.726732312
0.002163467 0.005806222 0.345531114 0.731947858
0.964434129 -7.357428706 cg24611631 9 4490288
p SLC1A1 SLC1A1 NA TSS200 island TSS200
- island NA 0.00506253 0.007225997 0.002163467
0.700599564
0.00159813 -0.003961202 0.3441493 0.732976901 0.964434129
-7.357916501 cg14629287 6 29716767 p MOG
LOC285830 NA Body island Body - island NA
-0.004510559 -0.002912428 0.00159813 1.548727813
-0.000852717 0.000669345 -0.338620369 0.737099331
0.964434129 -7.359848853 cg04852989 22
19974685 q COMT ARVCF NA Body island
Body - island NA 0.000962466 0.000109749
-0.000852717 8.769666351
-0.001066006 -0.000121322 -0.337861677 0.737665642
0.964434129 -7.36011159 cg14857596 12
72233493 q TPH2 TBC1D15 NA 1stExon island
1stExon - island NA 0.000245117 -0.000820889
-0.001066006 -0.298599944
0.002334403 0.008182523 0.337664784 0.737812634
0.964434129 -7.360179679 cg04049102 6
29716651 p MOG LOC285830 NA Body island
Body - island NA 0.007380072 0.009714475
0.002334403 0.759698502
0.003382047 -0.011725003 0.336509527 0.738675298
0.964434129 -7.360578393 cg09990446 6
29716108 p MOG LOC285830 NA Body shore
Body - shore NA -0.012887581 -0.009505534
0.003382047 1.35579769
0.005532893 -0.062528172 0.336074416 0.739000298
0.964434129 -7.360728211 cg20198768 6
29635579 p GABBR1/MOG MOG NA 3'UTR open

sea	3'UTR - open sea	NA	-0.064430104	-0.058897211
0.005532893	1.093941514
0.003997161	0.09457685 0.335055339	0.739761673
0.964434129	-7.361078348	cg24547396 22
19928740	q	COMT	COMT	NA	TSS1500	shore
TSS1500 - shore	NA	0.093202826	0.097199986
0.003997161	0.958876943
0.001681809	-0.005796863	0.334791247	0.739959025
0.964434129	-7.361168913	cg27230724 2
172751074	q	SLC25A12	SLC25A12	NA	TSS1500	shore
TSS1500 - shore	NA	-0.006374985	-0.004693176
0.001681809	1.35835192
-0.002405583	0.002173128	-0.333416229	0.740986849
0.964434129	-7.361639302	cg05972518 6
29577348	p	GABBR1/MOG GABBR1	NA	Body	open
sea	Body - open sea	NA	0.003000047	0.000594464
-0.002405583	5.046640703
-0.010956042	0.004796384	-0.332713103	0.741512621
0.964434129	-7.361879096	cg02157626 6
29648736	p	GABBR1/MOG ZFP57	8567	IGR	open
sea	IGR - open sea	NA	0.008562523	-0.002393519
-0.010956042	-3.577378554
0.003768055	0.022537263	0.332656282	0.741555115
0.964434129	-7.361898452	cg23708209 15
88843899	q	NTRK3	NTRK3-AS1 47938	IGR	open
sea	IGR - open sea	NA	0.021241994	0.025010049
0.003768055	0.849338351
-0.002236718	-0.008609059	-0.332330903	0.74179847
0.964434129	-7.36200923	cg21919834 22
19948833	q	COMT	COMT	NA	5'UTR	open
sea	5'UTR - open sea	NA	-0.007840187	-0.010076904
-0.002236718	0.778035255
-0.001808102	0.012540499	-0.332145492	0.741937153
0.964434129	-7.362072307	cg18761756 18	3732002
p	DLGAP1	DLGAP1	NA	Body	shelf	Body -
shelf	NA	0.013162034	0.011353933	-0.001808102
1.159248934
0.002311277	-0.011772235	0.331221471	0.742628429
0.964434129	-7.362386137	cg03167496 11
27743619	p	BDNF	BDNF	NA	TSS200	island
TSS200 - island	NA	-0.012566737	-0.010255459
0.002311277	1.225370421
0.001562376	0.002912615	0.329189148	0.744149609
0.964434129	-7.363073332	cg21434114 18	3450282
p	DLGAP1	TGIF1	NA	5'UTR	island	5'UTR -
island	NA	0.002375548	0.003937924	0.001562376
0.603248885
-0.001384464	-0.000319386	-0.328646408	0.744556023
0.964434129	-7.363256139	cg21619773 6
29720720	p	MOG	IFITM4P	2136	IGR	island
IGR - island	NA	0.000156523	-0.00122794
-0.001384464	-0.127468009
0.001169052	-0.005245199	0.328624427	0.744572485
0.964434129	-7.363263536	ch.15.1497565F	15
88581122	q	NTRK3	NTRK3	NA	Body	open
sea	Body - open sea	NA	-0.005647061	-0.004478008
0.001169052	1.261065268

0.002468145 0.001995802 0.328300038 0.744815431
0.964434129 -7.363372647 cg07169712 6
29571419 p GABBR1/MOG GABBR1 NA Body open
sea Body - open sea V$RREB1_01;V$TAXCREB_01 0.001147377
0.003615522 0.002468145 0.317347509
-0.003109008 -0.013341005 -0.327812702 0.745180464
0.964434129 -7.363536366 cg13696752 9 4662858
p SLC1A1 C9orf68 NA Body island Body -
island V$CP2_01;V$E2F_03 -0.012272283 -0.015381291 -
0.003109008 0.797870801
-0.003692982 -0.02827264 -0.326123558 0.74644616
0.965159117 -7.364101958 cg14298020 6
29712462 p MOG LOC285830 NA Body open
sea Body - open sea NA -0.027003178 -0.03069616
-0.003692982 0.87969238
-0.001381447 -0.01114864 -0.324097087 0.747965564
0.965588936 -7.364776671 cg08870743 21
34398199 q OLIG2 OLIG2 NA TSS200 island
TSS200 - island NA -0.010673767 -0.012055215
-0.001381447 0.885406643
0.009622207 0.189007729 0.322574458 0.749107874
0.965588936 -7.365280881 cg11768167 6
29690889 p GABBR1/MOG HLA-F NA TSS1500 shore
TSS1500 - shore NA 0.185700095 0.195322302
0.009622207 0.95073677
-0.000929382 0.00102981 -0.321936402 0.749586729
0.965588936 -7.365491467 cg17851017 6
29600994 p GABBR1/MOG GABBR1 NA TSS200 shore
TSS200 - shore NA 0.001349285 0.000419904
-0.000929382 3.213321021
0.001487898 -0.007406535 0.321623129 0.749821875
0.965588936 -7.36559471 cg00465250 2
171627741 q GAD1 GAD1 -45459 IGR island
IGR - island NA -0.007918 -0.006430102
0.001487898 1.231395716
-0.000992899 -0.001933925 -0.320975888 0.750307777
0.965588936 -7.365807699 cg24279419 6
29617791 p GABBR1/MOG MOG -6967 IGR island
IGR - island NA -0.001592616 -0.002585514
-0.000992899 0.615976378
0.001793732 -0.011508027 0.318302394 0.752315942
0.966082963 -7.366682954 cg21569006 21
34443672 q OLIG2 OLIG1 NA 1stExon island
1stExon - island NA -0.012124623 -0.010330891
0.001793732 1.173627994
-0.002719697 0.005263106 -0.316928276 0.75334878
0.966082963 -7.367129986 cg06538238 6
29588575 p GABBR1/MOG GABBR1 NA Body open
sea Body - open sea NA 0.006198002 0.003478305
-0.002719697 1.781902815
-0.009324015 0.104078641 -0.31653061 0.753647767
0.966082963 -7.367258997 cg08739755 2
172815385 q SLC25A12 HAT1 NA Body open
sea Body - open sea NA 0.107283771 0.097959756
-0.009324015 1.095182101
0.00132323 -0.00108739 0.316105486 0.753967442
0.966082963 -7.367396738 cg15568960 15

88790380 q NTRK3 NTRK3 NA Body open sea Body - open sea NA -0.00154225 -0.00021902 0.00132323 7.041591727
-0.001876114 -0.00503237 -0.315766116 0.754222664 0.966082963 -7.367506562 cg03448612 2 171672694 q GAD1 GAD1 NA TSS1500 island TSS1500 - island NA -0.004387456 -0.00626357 -0.001876114 0.700472066
0.0018177 -0.00430726 0.313666032 0.755802653 0.96720115 -7.36818357 cg05157371 6 29588894 p GABBR1/MOG GABBR1 NA Body open sea Body - open sea NA -0.004932094 -0.003114394 0.0018177 1.583644857
-0.003497267 0.007483841 -0.31010537 0.758483943 0.969725266 -7.369321165 cg21734356 18 3498854 p DLGAP1 DLGAP1 NA 3'UTR shore 3'UTR - shore NA 0.008686027 0.00518876 -0.003497267 1.674008348
-0.002477766 0.004846954 -0.307154704 0.760708196 0.970883989 -7.370254091 cg16733589 6 29628224 p GABBR1/MOG MOG NA Body open sea Body - open sea NA 0.005698686 0.00322092 - 0.002477766 1.769272678
-0.003579348 0.145167014 -0.307020149 0.760809675 0.970883989 -7.370296422 cg12061917 6 29601556 p GABBR1/MOG GABBR1 NA TSS1500 shore TSS1500 - shore NA 0.146397415 0.142818067 -0.003579348 1.025062293
-0.002948977 -0.002941249 -0.304628102 0.762614433 0.972280097 -7.371045891 cg23270757 6 29427699 p GABBR1 OR2H1 NA 5'UTR open sea 5'UTR - open sea NA -0.001927538 -0.004876515 -0.002948977 0.395269542
0.003164332 -0.009272269 0.30155949 0.764931626 0.973592734 -7.371998805 cg26310969 17 28662096 q SLC6A4 TMIGD1 NA TSS1500 open sea TSS1500 - open sea NA -0.010360008 -0.007195676 0.003164332 1.43975464
0.001367237 0.001489731 0.297976449 0.767640069 0.973592734 -7.373099328 cg24312412 17 28512027 q SLC6A4 CCDC55 NA Body open sea Body - open sea NA 0.001019744 0.002386981 0.001367237 0.427210663
0.00157381 -0.010705061 0.297266832 0.768176826 0.973592734 -7.373315733 cg15980539 6 152128865 q ESR1 ESR1 NA 5'UTR island 5'UTR - island NA -0.011246058 -0.009672248 0.00157381 1.162713964
0.001526762 -0.000674385 0.296137297 0.769031452 0.973592734 -7.373659138 cg09606766 11 27722971 p BDNF BDNF NA TSS1500 shore TSS1500 - shore NA -0.00119921 0.000327552 0.001526762 -3.661127289
-0.001227703 -0.000782624 -0.296051725 0.76909621 0.973592734 -7.373685101 cg06380702 6 29720825 p MOG IFITM4P 2241 IGR island

IGR - island NA -0.000360601 -0.001588304
-0.001227703 0.227035079
-0.003885587 0.004342039 -0.295691981 0.769368467
0.973592734 -7.373794167 cg04856117 22
19949901 q COMT COMT NA 5'UTR open
sea 5'UTR - open sea NA 0.00567771 0.001792123 -
0.003885587 3.168147999
-0.000826593 -0.000422041 -0.295485083 0.769525062
0.973592734 -7.373856834 cg13550731 2
172543902 q SLC25A12 DYNC1I2 NA TSS200 island
TSS200 - island NA -0.0001379 -0.000964493 -
0.000826593 0.142976669
0.002114171 -0.007911281 0.294713043 0.770109487
0.973592734 -7.374090292 cg26219152 6
152443475 q ESR1 SYNE1 NA 3'UTR open
sea 3'UTR - open sea NA -0.008638027 -0.006523856
0.002114171 1.324067657
0.005399279 -0.024627401 0.292327631 0.771916074
0.973592734 -7.374807778 cg07664000 17
28659293 q SLC6A4 TMIGD1 NA 5'UTR open
sea 5'UTR - open sea NA -0.026483403 -0.021084124
0.005399279 1.256082673
0.003507053 -0.056808611 0.290392626 0.773382499
0.973592734 -7.37538553 cg13974632 11
27740813 p BDNF BDNF NA Body island
Body - island NA -0.058014161 -0.054507108
0.003507053 1.064341207
-0.002827822 0.00488667 -0.289565069 0.774009915
0.973592734 -7.375631455 cg06266853 6
29589707 p GABBR1/MOG GABBR1 NA Body open
sea Body - open sea NA 0.005858733 0.003030912
-0.002827822 1.932993709
0.000814957 -0.001996088 0.289270735 0.774233103
0.973592734 -7.375718755 cg15306595 2
171673272 q GAD1 GAD1 NA 5'UTR island
5'UTR - island NA -0.00227623 -0.001461273
0.000814957 1.55770349
-0.002466623 -0.018736952 -0.288923 0.774496808
0.973592734 -7.375821779 cg21433429 18 4455713
p DLGAP1 DLGAP1-AS5 191111 IGR shore IGR -
shore NA -0.01788905 -0.020355673 -0.002466623
0.878823815
-0.00186865 -0.015336982 -0.288728922 0.774644
0.973592734 -7.375879225 cg15555970 18 3452317
p DLGAP1 TGIF1 NA 5'UTR island 5'UTR -
island NA -0.014694634 -0.016563283 -0.00186865
0.887181202
0.002573789 0.051246054 0.288198672 0.775046192
0.973592734 -7.376035981 cg26371172 6
29454888 p GABBR1 MAS1L NA 1stExon open
sea 1stExon - open sea NA 0.050361314 0.052935103
0.002573789 0.951378415
-0.004325107 -0.017021675 -0.288076101 0.775139171
0.973592734 -7.376072175 cg20801464 18 3454953
p DLGAP1 TGIF1 NA 5'UTR shelf 5'UTR -
shelf NA -0.015534919 -0.019860026 -0.004325107
0.782220468

-0.000688724 -0.000644071 -0.286284003 0.776498985
0.973592734 -7.376599621 cg00740645 6
29617868 p GABBR1/MOG MOG -6890 IGR island
IGR - island NA -0.000407322 -0.001096046
-0.000688724 0.371628665
-0.001788335 0.004696303 -0.285932074 0.776766107
0.973592734 -7.376702815 cg05183668 21
34350584 q OLIG2 OLIG2 -47632 IGR shore
IGR - shore NA 0.005311044 0.003522709
-0.001788335 1.507659091
-0.001311718 -0.000141123 -0.28540512 0.777166129
0.973592734 -7.376857095 cg20927575 6
29716340 p MOG LOC285830 NA Body shore
Body - shore NA 0.00030978 -0.001001937 -
0.001311718 -0.30918116
0.001069387 -0.002904029 0.281640803 0.780025498
0.976254239 -7.377950962 cg27569822 17
28563119 q SLC6A4 SLC6A4 NA TSS200 island
TSS200 - island NA -0.003271631 -0.002202244
0.001069387 1.485589672
-0.001384961 0.004954066 -0.28006803 0.781221099
0.976254239 -7.378403711 cg21864713 6
29555314 p GABBR1/MOG OR2H2 NA TSS1500 open
sea TSS1500 - open sea NA 0.005430146 0.004045185
-0.001384961 1.342372712
0.000715579 0.000221267 0.278389358 0.782497802
0.976254239 -7.378884162 cg10201663 6
29720685 p MOG IFITM4P 2101 IGR island
IGR - island NA -2.47E-05 0.000690866
0.000715579 -0.035772044
0.001451365 -0.007030283 0.276422112 0.783994758
0.976254239 -7.379443547 cg15126544 2
171678954 q GAD1 GAD1 NA Body island
Body - island NA -0.00752919 -0.006077824
0.001451365 1.238796842
-0.004290408 -0.081803042 -0.276263803 0.784115258
0.976254239 -7.37948839 cg05442477 21
34444584 q OLIG2 OLIG1 NA 1stExon shore
1stExon - shore NA -0.080328214 -0.084618622
-0.004290408 0.94929712
-0.001886109 -0.004481665 -0.275472108 0.784717954
0.976254239 -7.379712266 cg02589899 6
29627167 p GABBR1/MOG MOG NA Body open
sea Body - open sea V$TAXCREB_02 -0.003833315 -
0.005719424 -0.001886109 0.670227481
-0.010213088 -0.110506914 -0.271475815 0.787762282
0.976254239 -7.380832578 cg15841167 6
29633622 p GABBR1/MOG MOG NA 3'UTR open
sea 3'UTR - open sea NA -0.106996165 -0.117209253
-0.010213088 0.912864491
0.001721731 -0.002907641 0.270599463 0.788430333
0.976254239 -7.381076075 cg11257193 17
28444188 q SLC6A4 MIR423 NA Body shore
Body - shore NA -0.003499486 -0.001777755
0.001721731 1.968485769
-0.001472193 -0.022191284 -0.269080743 0.789588451
0.976254239 -7.381496198 cg22869726 21

34398265 q OLIG2 OLIG2 NA 1stExon island
1stExon - island NA -0.021685218 -0.023157411
-0.001472193 0.936426684
-0.001644997 0.008308072 -0.268688954 0.789887294
0.976254239 -7.381604196 cg15313740 2
171625337 q GAD1 GAD1 -47863 IGR shore
IGR - shore NA 0.008873539 0.007228543
-0.001644997 1.227569591
-0.002097176 -0.006409652 -0.267084876 0.791111165
0.976254239 -7.382044735 cg20496034 6
29574810 p GABBR1/MOG GABBR1 NA Body open
sea Body - open sea NA -0.005688748 -0.007785924
-0.002097176 0.730645252
-0.000828464 -0.001151035 -0.267062027 0.791128601
0.976254239 -7.382050991 cg17302062 18 3451564
p DLGAP1 TGIF1 NA 5'UTR island 5'UTR -
island V$P53_01 -0.00086625 -0.001694714 -0.000828464
0.51114841
0.000700952 -0.002213054 0.266228452 0.791764815
0.976254239 -7.382278865 cg26491697 18 3451447
p DLGAP1 TGIF1 NA 5'UTR island 5'UTR -
island NA -0.002454006 -0.001753054 0.000700952
1.399846351
0.000926709 0.001477302 0.266008514 0.791932704
0.976254239 -7.382338871 cg05200610 12
72233572 q TPH2 TBC1D15 NA 1stExon island
1stExon - island V$AP2_Q6;V$FAC1_01 0.001158746
0.002085455 0.000926709 0.555632073
0.00288314 0.016130244 0.264828878 0.792833346
0.976254239 -7.38265987 cg03534481 21
34439545 q OLIG2 OLIG1 -2905 IGR shelf
IGR - shelf NA 0.015139165 0.018022304
0.00288314 0.840023794
-0.007583271 0.033495001 -0.264349391 0.793199513
0.976254239 -7.382789941 cg02155405 2
172776401 q SLC25A12 HAT1 -2534 IGR shelf
IGR - shelf NA 0.03610175 0.028518479 -
0.007583271 1.265907282
-0.001656242 -0.00513782 -0.264146959 0.793354117
0.976254239 -7.382844784 cg21627017 6
29719792 p MOG IFITM4P NA TSS1500 shore
TSS1500 - shore NA -0.004568487 -0.006224729
-0.001656242 0.733925404
-0.001436591 0.002542013 -0.263129625 0.794131219
0.976254239 -7.38311977 cg17521020 6
29617613 p GABBR1/MOG MOG -7145 IGR shore
IGR - shore NA 0.003035841 0.00159925 -
0.001436591 1.898290406
-0.002910293 0.007305656 -0.262160213 0.794871915
0.976254239 -7.383380819 cg00539542 6
29644544 p GABBR1/MOG ZFP57 NA Body open
sea Body - open sea NA 0.00830607 0.005395777 -
0.002910293 1.539365001
0.001148277 -0.001670044 0.262129304 0.794895535
0.976254239 -7.383389126 cg03538731 18 3451548
p DLGAP1 TGIF1 NA 5'UTR island 5'UTR -

island NA -0.002064765 -0.000916488 0.001148277
2.252910534
-0.0007039 -0.000606426 -0.262101519 0.794916767
0.976254239 -7.383396593 cg11308211 2
172750783 q SLC25A12 SLC25A12 NA 5'UTR island
5'UTR - island NA -0.000364461 -0.00106836
-0.0007039 0.341140106
0.001734952 0.005904664 0.260038984 0.79649335
0.976254239 -7.383948689 cg10262357 6
29691832 p GABBR1/MOG HLA-F NA Body island
Body - island NA 0.005308274 0.007043226
0.001734952 0.753670771
0.001606369 -0.011994579 0.255592812 0.799894916
0.976254239 -7.385124058 cg12899157 18 3452302
p DLGAP1 TGIF1 NA 5'UTR island 5'UTR -
island NA -0.012546768 -0.010940399 0.001606369
1.146829135
0.002106583 -0.026425955 0.255143767 0.800238683
0.976254239 -7.385241643 cg24249411 11
27744759 p BDNF BDNF NA TSS1500 shore
TSS1500 - shore NA -0.027150093 -0.02504351
0.002106583 1.084116924
-0.002426633 0.001321107 -0.252053904 0.802605223
0.976254239 -7.386045159 cg09043524 22
19891284 q COMT TXNRD2 NA Body shelf
Body - shelf NA 0.002155262 -0.000271371
-0.002426633 -7.942112805
-0.002111376 -0.013858843 -0.250587183 0.803729257
0.976254239 -7.386423165 cg27646908 12
72332608 q TPH2 TPH2 NA TSS200 open
sea TSS200 - open sea NA -0.013133057 -0.015244433
-0.002111376 0.861498539
-0.003089638 0.019556351 -0.249443524 0.804606005
0.976254239 -7.386716386 cg05309280 2
171785085 q GAD1 GORASP2 NA TSS1500 shore
TSS1500 - shore NA 0.020618414 0.017528776
-0.003089638 1.176260925
-0.003258788 0.001656375 -0.249311625 0.804707137
0.976254239 -7.386750117 cg24351901 6
29692092 p GABBR1/MOG HLA-F NA Body island
Body - island NA 0.002776583 -0.000482205
-0.003258788 -5.75809748
0.000929626 -0.004740327 0.249125151 0.80485012
0.976254239 -7.386797775 cg13355041 18 3593715
p DLGAP1 FLJ35776 NA TSS1500 open sea TSS1500
- open sea V$P53_01 -0.005059886 -0.00413026 0.000929626
1.225076848
0.003439989 -0.061929095 0.248677603 0.805193317
0.976254239 -7.386912012 cg14531834 9
87655988 q NTRK2 NTRK2 371362 IGR open
sea IGR - open sea NA -0.063111592 -0.059671603
0.003439989 1.057648673
-0.001082178 -0.00227181 -0.24818499 0.805571115
0.976254239 -7.387037516 cg15313332 11
27721270 p BDNF BDNF NA Body shore
Body - shore NA -0.001899811 -0.002981989
-0.001082178 0.637095382

-0.000716841 -0.001811996 -0.248135472 0.805609094
0.976254239 -7.387050117 cg19783435 22
19974682 q COMT ARVCF NA Body island
Body - island NA -0.001565582 -0.002282423
-0.000716841 0.685929986
-0.002096927 -0.023811972 -0.247479213 0.806112478
0.976254239 -7.387216895 cg21164232 18 3452359
p DLGAP1 TGIF1 NA 5'UTR island 5'UTR -
island NA -0.023091153 -0.02518808 -0.002096927
0.916749242
0.004336143 0.155279073 0.244669353 0.808268731
0.976254239 -7.387925998 cg02824029 6
29570008 p GABBR1/MOG GABBR1 NA 3'UTR open
sea 3'UTR - open sea V$TCF11MAFG_01;V$BACH1_01;V$NFE2_01;V$AP1_01
0.153788524 0.158124667 0.004336143
0.972577691
-0.003944273 -0.05476279 -0.244377483 0.808492796
0.976254239 -7.387999193 cg00094412 6
29592854 p GABBR1/MOG GABBR1 NA Body shelf
Body - shelf NA -0.053406947 -0.05735122
-0.003944273 0.931225998
0.002061615 0.016545319 0.242213805 0.810154339
0.976254239 -7.388539081 cg22965752 6
29425885 p GABBR1 OR2H1 NA TSS1500 open
sea TSS1500 - open sea NA 0.015836639 0.017898254
0.002061615 0.884814755
-0.001595421 0.007162583 -0.241568654 0.810649941
0.976254239 -7.388699136 cg12760319 2
172541622 q SLC25A12 DYNC1I2 -2360 IGR shelf
IGR - shelf NA 0.007711009 0.006115588
-0.001595421 1.260877691
-0.000863308 -0.002687641 -0.241073508 0.811030363
0.976254239 -7.388821687 cg04661674 6
29596874 p GABBR1/MOG GABBR1 NA TSS1500 shore
TSS1500 - shore V$BRACH_01 -0.002390879 -0.003254187
-0.000863308 0.734708533
-0.002318667 0.023314711 -0.239654273 0.812121025
0.976254239 -7.389171567 cg12344104 6
29693309 p GABBR1/MOG HLA-F NA Body shore
Body - shore NA 0.024111753 0.021793085
-0.002318667 1.106394641
-0.001396727 -0.01792018 -0.239230296 0.812446919
0.976254239 -7.38927569 cg22593533 21
34397654 q OLIG2 OLIG2 NA TSS1500 island
TSS1500 - island NA -0.017440055 -0.018836782
-0.001396727 0.925851065
0.005214878 -0.017112255 0.237829382 0.813523989
0.976254239 -7.389618428 cg16289618 6
29705939 p MOG LOC285830 NA Body open
sea Body - open sea NA -0.018904869 -0.013689992
0.005214878 1.380926281
-0.001719839 0.003973087 -0.237175849 0.814026573
0.976254239 -7.389777631 cg21491555 22
19967786 q COMT ARVCF NA Body island
Body - island NA 0.004564282 0.002844443
-0.001719839 1.604631153

-0.000820379 0.002683369 -0.235623868 0.815220409
0.976254239 -7.390153949 cg18388802 22
20004382 q COMT ARVCF NA TSS200 island
TSS200 - island V$SP1_01 0.002965375 0.002144996
-0.000820379 1.382461935
0.001351222 -0.018450746 0.235253265 0.815505556
0.976254239 -7.390243448 cg05634149 21
34395317 q OLIG2 OLIG2 -2899 IGR island
IGR - island V$ARP1_01 -0.018915229 -0.017564006
0.001351222 1.076931329
0.003703548 -0.008017287 0.235036938 0.815672013
0.976254239 -7.390295624 cg07704699 11
27742832 p BDNF BDNF NA Body shore
Body - shore NA -0.009290381 -0.005586833
0.003703548 1.662906546
-0.002096368 0.005830343 -0.235033562 0.815674611
0.976254239 -7.390296438 cg18701449 12
72335228 q TPH2 TPH2 NA Body open
sea Body - open sea NA 0.006550969 0.004454601
-0.002096368 1.47060735
0.000900637 -0.001296066 0.234394555 0.816166357
0.976254239 -7.390450281 cg04627496 15
88799973 q NTRK3 NTRK3 NA TSS1500 island
TSS1500 - island NA -0.00160566 -0.000705022
0.000900637 2.277459165
0.001255905 -0.007829457 0.233930885 0.816523221
0.976254239 -7.39056165 cg05645755 18 4454548
p DLGAP1 DLGAP1-AS5 189946 IGR island IGR -
island NA -0.008261175 -0.00700527 0.001255905
1.179280051
-0.001915598 -0.003352523 -0.231395883 0.818474989
0.976254239 -7.391166645 cg01057705 22
19892666 q COMT TXNRD2 NA Body shore
Body - shore NA -0.002694037 -0.004609635
-0.001915598 0.584436015
0.001671298 -0.019019913 0.230551249 0.819125559
0.976254239 -7.391366763 cg03793625 2
171669862 q GAD1 GAD1 -3338 IGR shore
IGR - shore NA -0.019594422 -0.017923124
0.001671298 1.093248141
0.001110433 -0.005507702 0.230093096 0.8194785
0.976254239 -7.391475008 cg26232187 15
88799300 q NTRK3 NTRK3 NA Body shore
Body - shore NA -0.005889413 -0.00477898
0.001110433 1.232357618
0.000601768 -0.005452024 0.230025602 0.819530498
0.976254239 -7.391490936 cg10694442 22
20004377 q COMT ARVCF NA TSS200 island
TSS200 - island V$SP1_01 -0.005658882 -0.005057113
0.000601768 1.118994443
0.002961633 -0.02682048 0.229465059 0.819962376
0.976254239 -7.391623041 cg20196537 2
171574548 q GAD1 SP5 2691 IGR shore
IGR - shore NA -0.027838542 -0.024876909
0.002961633 1.119051489
-0.002569311 -0.024842971 -0.228221663 0.82092057
0.976254239 -7.39191493 cg04106006 11

27742454 p BDNF BDNF NA Body shore
Body - shore NA -0.02395977 -0.026529081
-0.002569311 0.903151158
-0.000706659 -0.008601506 -0.227690284 0.821330151
0.976254239 -7.39203919 cg23467008 6
152128537 q ESR1 ESR1 NA TSS1500 shore
TSS1500 - shore NA -0.008358592 -0.009065251
-0.000706659 0.922047534
0.001467875 -0.008626385 0.227445131 0.821519128
0.976254239 -7.39209642 cg02858594 21
34400211 q OLIG2 OLIG2 NA 3'UTR island
3'UTR - island NA -0.009130967 -0.007663092
0.001467875 1.191551238
0.000864025 -0.003705421 0.226112514 0.822546576
0.976254239 -7.39240644 cg22215258 2
172544429 q SLC25A12 DYNC1I2 NA 5'UTR island
5'UTR - island NA -0.00400243 -0.003138404
0.000864025 1.275307214
-0.00170343 0.006647882 -0.224385341 0.823878701
0.976254239 -7.392805548 cg03085859 6
29457094 p GABBR1 MAS1L NA TSS1500 open
sea TSS1500 - open sea NA 0.007233436 0.005530006
-0.00170343 1.308033944
0.00156341 -0.014260997 0.223434073 0.824612617
0.976254239 -7.39302406 cg20723129 6
29521501 p GABBR1 UBD -1888 IGR island
IGR - island NA -0.014798419 -0.013235009
0.00156341 1.118126844
0.000568419 7.73E-05 0.222993487 0.82495259 0.976254239
-7.393124952 cg24899205 22 19929184 q COMT
TXNRD2 NA Body island Body - island NA
-0.000118081 0.000450338 0.000568419 -
0.262204444
0.001462486 0.001055534 0.221377026 0.826200205
0.976254239 -7.393493412 cg14914809 2
171705073 q GAD1 GAD1 NA Body open
sea Body - open sea NA 0.000552804 0.00201529
0.001462486 0.274304936
-0.00262476 -0.015589833 -0.220848849 0.826607961
0.976254239 -7.393613227 cg08729810 21
34396944 q OLIG2 OLIG2 NA TSS1500 island
TSS1500 - island NA -0.014687572 -0.017312331
-0.00262476 0.848387862
-0.004817654 0.039400816 -0.220788481 0.826654569
0.976254239 -7.393626903 cg15708526 6
29648271 p GABBR1/MOG ZFP57 8102 IGR open
sea IGR - open sea NA 0.041056884 0.036239231
-0.004817654 1.13294028
-0.002497986 -0.014916952 -0.220257297 0.827064703
0.976254239 -7.393747079 cg23601416 22
19950040 q COMT COMT NA 5'UTR open
sea 5'UTR - open sea NA -0.01405827 -0.016556255
-0.002497986 0.849121341
0.000560008 -0.001405031 0.219729322 0.827472407
0.976254239 -7.393866243 cg22860601 2
171627721 q GAD1 GAD1 -45479 IGR island

IGR - island NA -0.001597534 -0.001037525
0.000560008 1.539753979
-0.002808834 -0.012339792 -0.219634618 0.827545543
0.976254239 -7.393887588 cg08805586 6
29689688 p GABBR1/MOG HLA-F NA TSS1500 shore
TSS1500 - shore NA -0.011374256 -0.014183089
-0.002808834 0.80195897
-0.00088524 -0.00075843 -0.217678047 0.829056871
0.976254239 -7.394326511 cg27306787 22
19879176 q COMT TXNRD2 NA Body open
sea Body - open sea NA -0.000454129 -0.001339368
-0.00088524 0.339061714
-0.002310945 -0.012875601 -0.217390443 0.829279083
0.976254239 -7.3943907 cg16257091 11 27743580 p
BDNF BDNF NA TSS1500 island TSS1500 - island
NA -0.012081214 -0.014392158 -0.002310945
0.839430291
0.001453626 0.00554077 0.217051277 0.829541153
0.976254239 -7.394466288 cg24430207 6
29589714 p GABBR1/MOG GABBR1 NA Body open
sea Body - open sea NA 0.005041086 0.006494712
0.001453626 0.776183163
0.002948872 -0.053359315 0.216800889 0.829734637
0.976254239 -7.394522016 cg16716320 15
88406509 q NTRK3 NTRK3-AS1 -13479 IGR open
sea IGR - open sea NA -0.05437299 -0.051424118
0.002948872 1.057344143
-0.001932049 0.002219284 -0.216151592 0.830236422
0.976254239 -7.394666226 cg19931596 2
172645256 q SLC25A12 SLC25A12 NA Body open
sea Body - open sea NA 0.002883425 0.000951377
-0.001932049 3.030792401
0.003821445 0.07710247 0.215245325 0.83093692 0.976254239
-7.39486679 cg26272069 6 29591706 p
GABBR1/MOG GABBR1 NA Body shelf Body - shelf
NA 0.075788849 0.079610293 0.003821445
0.95199811
-0.001380974 -0.001149603 -0.214664292 0.831386103
0.976254239 -7.394994935 cg09519060 6
29572346 p GABBR1/MOG GABBR1 NA Body open
sea Body - open sea NA -0.000674894 -0.002055867
-0.001380974 0.328276841
-0.003532883 0.008673476 -0.213315504 0.832429041
0.97664059 -7.395291074 cg19352507 6 29717262 p
MOG LOC285830 NA TSS1500 shore TSS1500 - shore
NA 0.009887905 0.006355022 -0.003532883
1.555919937
0.001075851 -0.006988421 0.21097584 0.834238896
0.977135293 -7.395800354 cg14276488 9 4491114
p SLC1A1 SLC1A1 NA Body shore Body -
shore NA -0.007358245 -0.006282394 0.001075851
1.171248639
0.001652374 -0.031363283 0.210923678 0.834279256
0.977135293 -7.395811644 cg03628748 9
87285133 q NTRK2 NTRK2 NA 5'UTR island
5'UTR - island NA -0.031931287 -0.030278913
0.001652374 1.054571784

-0.000626459 0.001772566 -0.209920741 0.835055373
0.977207656 -7.396028187 cg13924755 6
29691755 p GABBR1/MOG HLA-F NA Body island
Body - island NA 0.001987911 0.001361452
-0.000626459 1.46013993
-0.003381567 -0.074786426 -0.20893331 0.835819655
0.977266058 -7.396240377 cg12749246 6
29521013 p GABBR1 UBD -2376 IGR shore
IGR - shore NA -0.073624012 -0.077005579
-0.003381567 0.956086732
-0.001658709 -0.003599918 -0.207737056 0.836745784
0.977513436 -7.396496104 cg18656132 6
29571363 p GABBR1/MOG GABBR1 NA Body open
sea Body - open sea NA -0.003029737 -0.004688445
-0.001658709 0.646213504
-0.001067335 0.003164217 -0.206490374 0.837711208
0.977806257 -7.396761053 cg12111808 6
29588168 p GABBR1/MOG GABBR1 NA Body open
sea Body - open sea NA 0.003531113 0.002463778
-0.001067335 1.433210516
-0.001951377 0.003448926 -0.205437093 0.838527063
0.977924145 -7.39698366 cg13690679 17
28622405 q SLC6A4 TMIGD1 -20961 IGR shelf
IGR - shelf NA 0.004119712 0.002168335
-0.001951377 1.899942734
0.001496134 0.003580245 0.202126338 0.841092699
0.979423212 -7.397675983 cg01863613 17
28443583 q SLC6A4 CCDC55 NA TSS1500 shore
TSS1500 - shore V$MEF2_01 0.003065949 0.004562083
0.001496134 0.672050249
0.000642821 -0.00110521 0.200510186 0.842345764
0.979423212 -7.398009867 cg10190161 2
172778811 q SLC25A12 HAT1 NA TSS200 island
TSS200 - island V$MIF1_01;V$RFX1_01 -0.00132618 -
0.000683359 0.000642821 1.940678409
0.001634276 0.009110233 0.199676974 0.842991949
0.979423212 -7.398180957 cg20041152 15
88795689 q NTRK3 NTRK3 NA Body shelf
Body - shelf NA 0.008548451 0.010182727
0.001634276 0.839505078
0.000868906 0.001315552 0.199548186 0.843091839
0.979423212 -7.398207339 cg14514600 9 4666477
p SLC1A1 C9orf68 NA 1stExon shelf 1stExon
- shelf V$RFX1_02 0.001016866 0.001885772 0.000868906
0.539230524
-0.001450086 0.000338579 -0.19890715 0.843589073
0.979423212 -7.3983384 cg01176826 6 29720527 p
MOG IFITM4P 1943 IGR island IGR - island
NA 0.000837046 -0.000613039 -0.001450086
-1.36540393
0.002557332 -0.002890103 0.198238027 0.844108163
0.979423212 -7.398474754 cg21157690 6
152126895 q ESR1 ESR1 NA 5'UTR shore
5'UTR - shore NA -0.003769186 -0.001211854
0.002557332 3.1102633
0.001108682 0.001939198 0.195381485 0.846324998
0.981163218 -7.399051709 cg13427473 15

88406836	q	NTRK3	NTRK3-AS1	-13152	IGR	open
sea	IGR - open sea	NA	0.001558088	0.00266677
0.001108682	0.584260472
-0.003349426	0.00841509	-0.19259887	0.848485687
0.981392188	-7.399605699	cg09567915	6
29717260	p	MOG	LOC285830	NA	TSS1500	shore
TSS1500 - shore	NA	0.009566455	0.00621703	-
0.003349426	1.538750139
0.003497564	0.368889182	0.191544658	0.849304591
0.981392188	-7.399813511	cg25730428	6
29454755	p	GABBR1	MAS1L	NA	1stExon	open
sea	1stExon - open sea	NA	0.367686895	0.371184459
0.003497564	0.990577289
-0.003762751	-0.023954264	-0.190608447	0.850031975
0.981392188	-7.399997108	cg20208009	22
19974048	q	COMT	ARVCF	NA	Body	shore
Body - shore	NA	-0.022660818	-0.02642357
-0.003762751	0.85759868
-0.001407024	-0.042131584	-0.190144202	0.850392718
0.981392188	-7.400087816	cg21091227	18	4454304
p	DLGAP1	DLGAP1-AS5	189702	IGR	island	IGR -
island	NA	-0.041647919	-0.043054943	-0.001407024
0.96732027
0.000470347	-0.001178211	0.189300399	0.85104848
0.981392188	-7.400252121	cg03471611	17
28431762	q	SLC6A4	EFCAB5	NA	Body	open
sea	Body - open sea	NA	-0.001339893	-0.000869546
0.000470347	1.540910511
0.001717493	-0.011375578	0.187527858	0.852426363
0.981392188	-7.400594895	cg06874218	18	3447454
p	DLGAP1	TGIF1	NA	5'UTR	shore	5'UTR -
shore	NA	-0.011965966	-0.010248474	0.001717493
1.167585224
-0.001647787	-0.056859878	-0.186985698	0.852847905
0.981392188	-7.400699095	cg06014588	15
88801339	q	NTRK3	NTRK3-AS1	5378	IGR	shore
IGR - shore	NA	-0.056293451	-0.057941239
-0.001647787	0.971561059
-0.000496047	-0.00278982	-0.18623721	0.853429945
0.981392188	-7.400842456	cg12912949	22
20004611	q	COMT	ARVCF	NA	TSS1500	island
TSS1500 - island	NA	-0.002619304	-0.003115352
-0.000496047	0.840773216
-0.002050182	0.026655234	-0.185742866	0.853814402
0.981392188	-7.400936825	cg25191596	6
29455126	p	GABBR1	MAS1L	NA	1stExon	open
sea	1stExon - open sea	NA	0.027359984	0.025309802
-0.002050182	1.08100348
-0.001949606	0.020345322	-0.185227359	0.854215357
0.981392188	-7.401034968	cg06643882	18	3773774
p	DLGAP1	DLGAP1	NA	Body	shelf	Body -
shelf	NA	0.021015499	0.019065893	-0.001949606
1.102256207
-0.001717741	-0.013400021	-0.18463648	0.854674984
0.981392188	-7.401147125	cg09265315	6
29572317	p	GABBR1/MOG	GABBR1	NA	Body	open

sea Body - open sea NA -0.012809548 -0.014527289
-0.001717741 0.881757632
0.001199762 -0.009725418 0.183270684 0.855737593
0.981392188 -7.401405003 cg06412358 21
34392373 q OLIG2 OLIG2 -5843 IGR island
IGR - island NA -0.010137836 -0.008938074
0.001199762 1.134230519
-0.003080048 0.003036978 -0.183128029 0.855848596
0.981392188 -7.401431827 cg12931591 18 3411821
p DLGAP1 TGIF1 NA TSS1500 open sea TSS1500
- open sea NA 0.004095744 0.001015696 -0.003080048
4.032449204
-0.001683102 -0.019308236 -0.181194693 0.857353269
0.982294197 -7.401793315 cg18513624 22
19974307 q COMT ARVCF NA Body island
Body - island NA -0.01872967 -0.020412772
-0.001683102 0.917546632
-0.001823816 -0.026885937 -0.180249183 0.858089335
0.98231482 -7.401968708 cg18772882 15 88616079 q
NTRK3 NTRK3 NA Body open sea Body - open sea
NA -0.026259 -0.028082816 -0.001823816
0.935055816
0.000856566 -0.003149588 0.178650153 0.859334453
0.982917669 -7.402263247 cg06078334 6
29595653 p GABBR1/MOG GABBR1 NA Body island
Body - island NA -0.003444032 -0.002587466
0.000856566 1.331044294
-0.001153412 0.013059479 -0.176317298 0.861151635
0.984173297 -7.402688256 cg25000210 6
29455302 p GABBR1 MAS1L NA 1stExon open
sea 1stExon - open sea NA 0.013455964 0.012302553
-0.001153412 1.093753854
0.001258963 -0.007981339 0.175356017 0.861900651
0.984207088 -7.402861764 cg12448003 11
27742365 p BDNF BDNF NA Body shore
Body - shore NA -0.008414108 -0.007155145
0.001258963 1.175952071
0.000745984 -0.001206 0.168855176 0.866969392
0.988418481 -7.404010294 cg00476814 22
19974536 q COMT ARVCF NA Body island
Body - island NA -0.001462432 -0.000716448
0.000745984 2.041224891
0.001610671 0.043296589 0.168380016 0.867340105
0.988418481 -7.404092544 cg06760467 6
29496514 p GABBR1 LINC01015 -669 IGR open
sea IGR - open sea NA 0.042742921 0.044353592
0.001610671 0.963685679
-0.000604996 -0.003046851 -0.167393981 0.868109491
0.988418481 -7.404262488 cg00415702 6
29720841 p MOG IFITM4P 2257 IGR island
IGR - island NA -0.002838884 -0.00344388
-0.000604996 0.824327103
0.001889806 0.07417424 0.166685147 0.868662663
0.988418481 -7.404384041 cg15198068 22
19843949 q COMT GNB1L NA TSS1500 shore
TSS1500 - shore NA 0.073524619 0.075414425
0.001889806 0.974941049

-0.000485336 -0.000747086 -0.165220833 0.869805622
0.988418481 -7.404633515 cg19339932 22
20004356 q COMT ARVCF NA TSS200 island
TSS200 - island NA -0.000580252 -0.001065588
-0.000485336 0.544536535
0.00244167 -0.123924272 0.165069362 0.869923868
0.988418481 -7.404659195 cg08179037 6
29705815 p MOG LOC285830 NA Body open
sea Body - open sea NA -0.124763596 -0.122321926
0.00244167 1.019961018
-0.00219455 -0.001393811 -0.163904972 0.870832952
0.988630272 -7.404855822 cg10503635 6
29634213 p GABBR1/MOG MOG NA 3'UTR open
sea 3'UTR - open sea NA -0.000639434 -0.002833984
-0.00219455 0.225630738
-0.000724756 -0.026673934 -0.161435598 0.872761474
0.989172808 -7.405268219 cg27278787 18 3499813
p DLGAP1 DLGAP1 NA Body shore Body -
shore NA -0.0264248 -0.027149555 -0.000724756
0.973305061
-0.001493101 0.020731829 -0.160986145 0.873112571
0.989172808 -7.405342608 cg15641340 6
29429909 p GABBR1 OR2H1 NA Body open
sea Body - open sea V$PPARG_01;V$PPARA_01 0.021245083
0.019751982 -0.001493101 1.075592485
0.000858734 -0.010294479 0.160515717 0.873480082
0.989172808 -7.405420246 cg26598649 18 3880086
p DLGAP1 DLGAP1 NA 1stExon island 1stExon
- island NA -0.010589669 -0.009730935 0.000858734
1.088247845
0.002689051 0.002250297 0.159160266 0.874539155
0.98955299 -7.405642678 cg11497864 6 29717269 p
MOG LOC285830 NA TSS1500 shore TSS1500 - shore
NA 0.001325935 0.004014987 0.002689051
0.330246559
-0.001365694 0.006893123 -0.156646426 0.876503951
0.989562488 -7.406050218 cg27012424 18 3773224
p DLGAP1 DLGAP1 NA Body shore Body -
shore NA 0.00736258 0.005996887 -0.001365694
1.22773377
-0.004364526 0.00080356 -0.156343339 0.876740895
0.989562488 -7.406098916 cg19636627 6
29649084 p GABBR1/MOG ZFP57 8915 IGR open
sea IGR - open sea NA 0.002303866 -0.002060661
-0.004364526 -1.118022842
0.001072799 0.003195014 0.156179685 0.87686884
0.989562488 -7.406125172 cg25804470 6
29600193 p GABBR1/MOG GABBR1 NA 5'UTR island
5'UTR - island NA 0.002826239 0.003899038
0.001072799 0.724855517
-0.000709089 -0.006567712 -0.155218596 0.877620287
0.989562488 -7.406278811 cg06991510 11
27723237 p BDNF BDNF NA TSS1500 shore
TSS1500 - shore NA -0.006323963 -0.007033052
-0.000709089 0.89917759
0.002530368 0.015143718 0.152377238 0.879842532
0.989562488 -7.406727489 cg20720918 2

171785124 q GAD1 GORASP2 NA TSS1500 shore
TSS1500 - shore NA 0.014273904 0.016804272
0.002530368 0.849421139
0.00071332 -0.002605524 0.152054759 0.880094808
0.989562488 -7.406777889 cg18867480 11
27744816 p BDNF BDNF NA TSS1500 shore
TSS1500 - shore NA -0.002850728 -0.002137408
0.00071332 1.333731187
-0.000722247 0.002265675 -0.150599113 0.881233721
0.989562488 -7.407004062 cg03789152 12
72233372 q TPH2 TBC1D15 NA TSS200 island
TSS200 - island NA 0.002513947 0.0017917 -
0.000722247 1.403106809
0.001267125 -0.021095532 0.150407187 0.881383906
0.989562488 -7.40703372 cg09545764 6
29715162 p MOG LOC285830 NA Body shore
Body - shore NA -0.021531106 -0.020263981
0.001267125 1.062530918
0.001048871 0.000850376 0.150013271 0.881692163
0.989562488 -7.407094475 cg04847841 21
34351148 q OLIG2 OLIG2 -47068 IGR shore
IGR - shore NA 0.000489827 0.001538698
0.001048871 0.318338642
-0.000389473 -0.002036917 -0.149037124 0.882456125
0.989562488 -7.407244342 cg14480858 9 4666499
p SLC1A1 C9orf68 NA 1stExon shelf 1stExon
- shelf NA -0.001903036 -0.002292508 -0.000389473
0.830110658
-0.000986437 0.005045258 -0.148975243 0.882504558
0.989562488 -7.407253809 cg26920808 12
72237548 q TPH2 TBC1D15 NA Body shelf
Body - shelf NA 0.005384345 0.004397909
-0.000986437 1.224296798
0.001199345 0.009284896 0.143257127 0.886982042
0.9903581 -7.408111718 cg05054006 17 28443555 q
SLC6A4 CCDC55 NA TSS1500 shore TSS1500 - shore
NA 0.008872621 0.010071966 0.001199345
0.880922496
0.00259477 -0.090810156 0.143192015 0.887033049
0.9903581 -7.408121294 cg15180617 6 29705436 p
MOG LOC285830 NA Body open sea Body - open sea
NA -0.091702108 -0.089107338 0.00259477
1.029119604
0.001443665 -0.006374476 0.142201026 0.887809424
0.9903581 -7.4082665 cg15899474 2 172779515 q
SLC25A12 HAT1 NA Body shore Body - shore
NA -0.006870735 -0.005427071 0.001443665
1.266011775
0.000501006 -0.005542666 0.141112695 0.88866219
0.9903581 -7.408424809 cg24102938 17 28444044 q
SLC6A4 MIR423 NA TSS200 shore TSS200 - shore
NA -0.005714887 -0.005213881 0.000501006
1.096090759
-0.001033537 -0.007825189 -0.140882208 0.888842806
0.9903581 -7.40845818 cg26744084 6 29581182 p
GABBR1/MOG GABBR1 NA Body open sea Body - open sea

NA -0.00746991 -0.008503447 -0.001033537
0.878456667
-0.000986221 -0.015083422 -0.14083 0.888883719
0.9903581 -7.408465731 cg03696345 21 34398114 q
OLIG2 OLIG2 NA TSS200 island TSS200 - island
NA -0.014744408 -0.01573063 -0.000986221
0.937305657
-0.002265881 0.133305945 -0.140529052 0.889119561
0.9903581 -7.408509206 cg19022254 6 29601705 p
GABBR1/MOG GABBR1 NA TSS1500 shore TSS1500 - shore
NA 0.134084841 0.13181896 -0.002265881
1.017189342
-0.000473164 -0.000150569 -0.139526856 0.889905021
0.9903581 -7.408653311 cg16203801 6 29716637 p
MOG LOC285830 NA Body island Body - island
NA 1.21E-05 -0.000461083 -0.000473164 -
0.026201455
-0.000771583 -0.006543886 -0.13909837 0.890240877
0.9903581 -7.408714609 cg24671939 18 3593798 p
DLGAP1 FLJ35776 NA TSS1500 open sea TSS1500 - open
sea NA -0.006278654 -0.007050237 -0.000771583
0.890559275
0.000927192 -0.001207842 0.138827048 0.890453555
0.9903581 -7.408753326 cg08983330 6 29718869 p
MOG IFITM4P NA Body shore Body - shore
NA -0.001526564 -0.000599372 0.000927192
2.546938463
-0.000428887 -0.000227674 -0.134079143 0.894176565
0.992047836 -7.409418617 cg24847163 6
29600980 p GABBR1/MOG GABBR1 NA TSS200 shore
TSS200 - shore NA -8.02E-05 -0.000509132 -
0.000428887 0.157609748
-0.00087167 -0.016148337 -0.13270373 0.895255539
0.992047836 -7.409607025 cg05522774 21
34443443 q OLIG2 OLIG1 NA 1stExon island
1stExon - island NA -0.0158487 -0.016720371 -
0.00087167 0.947867753
0.001408686 -0.032776927 0.129627697 0.897669332
0.992047836 -7.410021366 cg07601542 21
34396986 q OLIG2 OLIG2 NA TSS1500 island
TSS1500 - island NA -0.033261163 -0.031852477
0.001408686 1.044225307
0.000396217 -0.001038691 0.125952037 0.900554958
0.992047836 -7.410503746 cg03434929 6
29617848 p GABBR1/MOG MOG -6910 IGR island
IGR - island NA -0.001174891 -0.000778673
0.000396217 1.508836544
-0.000328337 0.001798963 -0.125448162 0.90095064
0.992047836 -7.410568793 cg14555167 22
19842472 q COMT GNB1L NA TSS200 island
TSS200 - island NA 0.001911829 0.001583492
-0.000328337 1.207350028
-0.000943066 0.001125986 -0.124767277 0.901485365
0.992047836 -7.410656276 cg17375177 6
29627845 p GABBR1/MOG MOG NA Body open
sea Body - open sea NA 0.001450165 0.000507099
-0.000943066 2.859726924

-0.001026643	0.006037745	-0.123838641	0.902214734
0.992047836	-7.410774825	cg07476327 2
171730024 q	GAD1	GORASP2	-54924	IGR	open
sea	IGR - open sea	NA	0.006390653	0.005364011
-0.001026643	1.191394608
0.000481364	-0.002955704	0.123190598	0.902723771
0.992047836	-7.410857029	cg25346576 17
28443852 q	SLC6A4	CCDC55	NA	5'UTR	island
5'UTR - island	NA	-0.003121173	-0.002639809
0.000481364	1.182348135
0.000706427	0.000274269	0.122646177	0.903151446
0.992047836	-7.410925756	cg06581978 21
34408891 q	OLIG2	OLIG2	10675	IGR	shelf
IGR - shelf	NA	3.14E-05	0.000737862
0.000706427	0.042602546
0.000744863	0.007816328	0.121752818	0.903853294
0.992047836	-7.411037873	cg07584093 6
152126180 q	ESR1	ESR1	NA	5'UTR	shelf
5'UTR - shelf	NA	0.007560282	0.008305144
0.000744863	0.910313094
0.000718903	-0.006743416	0.121237958	0.904257819
0.992047836	-7.411102116	cg14278853 6
29521756 p	GABBR1	UBD	-1633	IGR	island
IGR - island	NA	-0.006990539	-0.006271636
0.000718903	1.114627618
-0.000440541	0.002698902	-0.120258178	0.905027703
0.992047836	-7.41122362	cg17625506 6
29691726 p	GABBR1/MOG HLA-F	NA	Body	island
Body - island	NA	0.002850338	0.002409797
-0.000440541	1.182812569
0.001117715	0.010256763	0.119669836	0.905490051
0.992047836	-7.411296108	cg06045576 22
19948957 q	COMT	COMT	NA	5'UTR	open
sea	5'UTR - open sea	NA	0.009872549	0.010990263
0.001117715	0.898299546
0.000827802	-0.002593841	0.118592458	0.906336794
0.992047836	-7.411427928	cg25504668 6
29572379 p	GABBR1/MOG GABBR1	NA	Body	open
sea	Body - open sea	NA	-0.002878397	-0.002050595
0.000827802	1.403688605
0.002457155	-0.002253881	0.11767072 0.907061303
0.992047836	-7.411539759	cg04522432 6
29618347 p	GABBR1/MOG MOG	-6411	IGR	shore
IGR - shore	NA	-0.003098529	-0.000641373
0.002457155	4.831084357
0.000714646	-0.011867608	0.116672614	0.907845931
0.992047836	-7.411659873	cg20034792 6
29521781 p	GABBR1	UBD	-1608	IGR	island
IGR - island	NA	-0.012113268	-0.011398621
0.000714646	1.062695841
0.000734818	0.005115262	0.116249909	0.908178255
0.992047836	-7.411710434	cg02324737 2
172543905 q	SLC25A12	DYNC1I2	NA	TSS200	island
TSS200 - island	NA	0.004862668	0.005597486
0.000734818	0.868723596
0.000941824	-0.00101724	0.115968369	0.908399607
0.992047836	-7.411744008	cg16924391 6

29719411 p MOG IFITM4P NA TSS1500 shore
TSS1500 - shore NA -0.001340992 -0.000399167
0.000941824 3.359471913
-0.001233166 0.013435087 -0.115665298 0.908637894
0.992047836 -7.411780059 cg11806762 11
27732958 p BDNF BDNF NA Body open
sea Body - open sea NA 0.013858988 0.012625822
-0.001233166 1.097670169
-0.001236839 0.003904744 -0.115377585 0.908864115
0.992047836 -7.411814195 cg06212263 21
34392851 q OLIG2 OLIG2 -5365 IGR shore
IGR - shore NA 0.004329907 0.003093068
-0.001236839 1.399874377
-0.000680313 -0.000515003 -0.114954133 0.909197078
0.992047836 -7.411864282 cg13811469 22
20020657 q COMT C22orf25 NA 5'UTR open
sea 5'UTR - open sea NA -0.000281145 -0.000961458
-0.000680313 0.292415407
0.001698598 -0.043259202 0.114376777 0.909651083
0.992047836 -7.411932278 cg21670199 15
88801401 q NTRK3 NTRK3-AS1 5440 IGR shore
IGR - shore V$AHRARNT_02 -0.043843095 -
0.042144497 0.001698598 1.040304144
0.000408309 -0.008317803 0.113206889 0.910571122
0.992047836 -7.412069006 cg01763173 2
171674437 q GAD1 GAD1 NA 5'UTR island
5'UTR - island NA -0.008458159 -0.00804985
0.000408309 1.050722571
-0.000416344 0.00252344 -0.11315867 0.910609045
0.992047836 -7.412074611 cg14446129 2
172778865 q SLC25A12 HAT1 NA TSS200 island
TSS200 - island V$ELK1_01 0.002666558 0.002250215
-0.000416344 1.185023936
-0.000668751 -0.021081893 -0.112924772 0.910793007
0.992047836 -7.412101768 cg23165623 6
152128411 q ESR1 ESR1 NA TSS1500 shore
TSS1500 - shore NA -0.020852009 -0.021520761
-0.000668751 0.968925289
0.000260884 0.002284776 0.112195841 0.911366344
0.992047836 -7.412186039 cg05101432 6
29720737 p MOG IFITM4P 2153 IGR island
IGR - island NA 0.002195097 0.002455981
0.000260884 0.89377601
0.000570925 0.01373299 0.111935992 0.911570739
0.992047836 -7.412215948 cg04661128 9 4661938
p SLC1A1 PPAPDC2 NA TSS1500 shore TSS1500
- shore NA 0.013536734 0.01410766 0.000570925
0.959530816
-0.001219815 -0.051200082 -0.109565904 0.913435311
0.992047836 -7.412485551 cg11234013 11
27646797 p BDNF BDNFOS NA Body open
sea Body - open sea NA -0.050780771 -0.052000585
-0.001219815 0.976542291
0.001161605 0.010825079 0.109535299 0.913459391
0.992047836 -7.412488995 cg00521620 6
29434416 p GABBR1 OR2H1 8186 IGR open

sea IGR - open sea NA 0.010425778 0.011587382
0.001161605 0.899752625
-0.000525326 -0.008093398 -0.108732938 0.914090732
0.992047836 -7.412578934 cg21133153 6
29521488 p GABBR1 UBD -1901 IGR island
IGR - island NA -0.007912817 -0.008438143
-0.000525326 0.937743921
0.000997373 0.00948179 0.107967494 0.914693078
0.992047836 -7.412664119 cg27297993 6
29581228 p GABBR1/MOG GABBR1 NA Body open
sea Body - open sea V$PAX4_04 0.009138943 0.010136316
0.000997373 0.901604026
0.000750987 -0.01701767 0.107350512 0.915178633
0.992047836 -7.412732344 cg22762215 6
29521272 p GABBR1 UBD -2117 IGR island
IGR - island NA -0.017275822 -0.016524835
0.000750987 1.045445943
-0.000293745 -0.000729508 -0.103902557 0.917892722
0.992049348 -7.413106423 cg14420670 6
29617961 p GABBR1/MOG MOG -6797 IGR island
IGR - island NA -0.000628533 -0.000922278
-0.000293745 0.681500448
0.000907631 0.043572188 0.102746938 0.918802604
0.992049348 -7.413229069 cg00237606 6
29455256 p GABBR1 MAS1L NA 1stExon open
sea 1stExon - open sea NA 0.04326019 0.044167821
0.000907631 0.979450403
0.000245481 0.002054378 0.102475906 0.919016019
0.992049348 -7.413257635 cg16548348 22
19842481 q COMT GNB1L NA TSS200 island
TSS200 - island NA 0.001969994 0.002215475
0.000245481 0.889196961
0.000895503 -0.00607336 0.101985623 0.91940209
0.992049348 -7.413309118 cg13873869 6
29638112 p GABBR1/MOG MOG NA 3'UTR open
sea 3'UTR - open sea NA -0.006381189 -0.005485686
0.000895503 1.163243609
0.001235053 0.011253397 0.10169347 0.919632154
0.992049348 -7.413339679 cg02723395 2
171678751 q GAD1 GAD1 NA Body island
Body - island NA 0.010828848 0.012063901
0.001235053 0.897624064
-0.000294228 -0.001425846 -0.10129002 0.919949874
0.992049348 -7.413381738 cg14520770 22
19842507 q COMT GNB1L NA TSS200 island
TSS200 - island NA -0.001324705 -0.001618933
-0.000294228 0.818257975
0.001055202 0.018231872 0.098942964 0.921798461
0.992049348 -7.413623102 cg09072576 22
19838287 q COMT GNB1L NA 5'UTR shelf
5'UTR - shelf NA 0.017869146 0.018924348
0.001055202 0.944241026
0.000966853 0.000497396 0.098583539 0.922081591
0.992049348 -7.413659565 cg13460297 2
172581104 q SLC25A12 DYNC1I2 NA Body open
sea Body - open sea NA 0.000165041 0.001131893
0.000966853 0.145809396

-0.000394771 0.002929963 -0.098046729 0.92250447
0.992049348 -7.413713776 cg17748329 6
29717058 p MOG LOC285830 NA TSS1500 island
TSS1500 - island NA 0.003065665 0.002670894
-0.000394771 1.147804938
-0.000736624 0.001207368 -0.096722999 0.923547356
0.992049348 -7.413846193 cg00107488 22
19930437 q COMT COMT NA 5'UTR shore
5'UTR - shore NA 0.001460583 0.000723958
-0.000736624 2.017495104
-0.001746496 -0.003619651 -0.095623235 0.924413897
0.992049348 -7.413954837 cg12349676 21
34350934 q OLIG2 OLIG2 -47282 IGR island
IGR - island NA -0.003019293 -0.004765789
-0.001746496 0.633534714
-0.001104358 0.014888037 -0.094960669 0.924936001
0.992049348 -7.414019692 cg11361387 22
19949873 q COMT COMT NA 5'UTR open
sea 5'UTR - open sea NA 0.015267661 0.014163303
-0.001104358 1.077973188
-0.001183801 0.006603127 -0.093801531 0.925849486
0.992049348 -7.414132069 cg09902251 15
88590236 q NTRK3 NTRK3 NA Body open
sea Body - open sea NA 0.007010059 0.005826257
-0.001183801 1.203183853
-0.001061913 0.005215008 -0.093235448 0.926295638
0.992049348 -7.414186449 cg19601636 22
20001066 q COMT ARVCF NA 5'UTR shelf
5'UTR - shelf NA 0.005580041 0.004518128
-0.001061913 1.23503394
-0.000575305 -0.010541954 -0.092078901 0.927207232
0.992049348 -7.414296528 cg06260077 11
27721350 p BDNF BDNF NA Body shore
Body - shore NA -0.010344193 -0.010919498
-0.000575305 0.947313947
0.000639141 0.003498898 0.091917657 0.927334333
0.992049348 -7.414311766 cg24937995 21
34448038 q OLIG2 OLIG1 5588 IGR shelf
IGR - shelf NA 0.003279194 0.003918335
0.000639141 0.836884461
-0.00035268 -0.003936011 -0.091459636 0.927695381
0.992049348 -7.414354905 cg11241206 11
27723128 p BDNF BDNF NA TSS1500 shore
TSS1500 - shore NA -0.003814777 -0.004167457
-0.00035268 0.915372875
-0.000175519 -0.001155753 -0.088643863 0.929915326
0.992049348 -7.414615376 cg00716604 12
72233552 q TPH2 TBC1D15 NA 1stExon island
1stExon - island NA -0.001095418 -0.001270937
-0.000175519 0.861897601
0.000546587 -0.000553742 0.084696979 0.933027995
0.992049348 -7.414966776 cg14592798 9
87257941 q NTRK2 NTRK2 -25525 IGR open
sea IGR - open sea NA -0.000741631 -0.000195044
0.000546587 3.802378805
0.000517632 2.32E-05 0.08466886 0.933050175 0.992049348
-7.414969223 cg20408707 6 29431410 p GABBR1

OR2H1 NA 3'UTR open sea 3'UTR - open sea NA
-0.000154727 0.000362905 0.000517632 -
0.426355998
-0.000444753 -0.003514631 -0.084332894 0.933315181
0.992049348 -7.414998386 cg27217194 6
29600139 p GABBR1/MOG GABBR1 NA Body shore
Body - shore NA -0.003361747 -0.003806501
-0.000444753 0.88315949
-0.000887777 -0.037572135 -0.08320914 0.934201644
0.992049348 -7.415095091 cg00433866 6
29623646 p GABBR1/MOG MOG NA TSS1500 open
sea TSS1500 - open sea NA -0.037266962 -0.038154739
-0.000887777 0.976732191
0.001428386 -0.106057566 0.082640635 0.934650137
0.992049348 -7.41514352 cg00601836 6
152130332 q ESR1 ESR1 NA Body shore
Body - shore NA -0.106548573 -0.105120188
0.001428386 1.013588122
0.000905219 0.002240571 0.082019741 0.935139984
0.992049348 -7.415196033 cg16164802 6
29691808 p GABBR1/MOG HLA-F NA Body island
Body - island NA 0.001929401 0.002834621
0.000905219 0.680655942
-0.000532546 0.000854637 -0.078170758 0.938177159
0.992049348 -7.415512731 cg14053318 22
19967559 q COMT ARVCF NA Body island
Body - island V$NRSF_01 0.0010377 0.000505154 -
0.000532546 2.054226592
0.001275041 0.012898501 0.076896663 0.939182739
0.992049348 -7.415614213 cg04071440 6
29648275 p GABBR1/MOG ZFP57 8106 IGR open
sea IGR - open sea NA 0.012460205 0.013735246
0.001275041 0.907170123
-0.000413171 -0.00134805 -0.076339777 0.939622293
0.992049348 -7.415658046 cg11209538 6
29600112 p GABBR1/MOG GABBR1 NA Body shore
Body - shore NA -0.001206023 -0.001619193
-0.000413171 0.744829358
-0.00020247 -0.002870782 -0.074791828 0.940844202
0.992049348 -7.415778212 cg16639998 2
171626995 q GAD1 GAD1 -46205 IGR shore
IGR - shore NA -0.002801183 -0.003003653
-0.00020247 0.932592085
0.000520136 -0.000905248 0.074006679 0.941464032
0.992049348 -7.415838222 cg07436579 6
29581117 p GABBR1/MOG GABBR1 NA Body open
sea Body - open sea V$PAX5_01 -0.001084045 -0.000563909
0.000520136 1.92237503
-0.000314134 0.001497335 -0.07322313 0.942082637
0.992049348 -7.415897479 cg27066254 17
28443640 q SLC6A4 CCDC55 NA TSS200 shore
TSS200 - shore NA 0.001605319 0.001291185
-0.000314134 1.243291086
0.000726734 -0.003587827 0.07301132 0.942249866
0.992049348 -7.415913389 cg05437995 6
29571875 p GABBR1/MOG GABBR1 NA Body open

sea Body - open sea NA -0.003837642 -0.003110908
0.000726734 1.233608251
0.000968597 -0.017926207 0.07297304 0.942280089
0.992049348 -7.41591626 cg10635145 11
27742435 p BDNF BDNF NA Body shore
Body - shore NA -0.018259162 -0.017290566
0.000968597 1.056018792
0.000149018 -9.78E-05 0.072949154 0.942298948
0.992049348 -7.41591805 cg07038887 17
28618554 q SLC6A4 BLMH NA Body island
Body - island NA -0.000149051 -3.28E-08
0.000149018 4545.250654
0.000518121 0.00280041 0.070718697 0.944060108
0.992049348 -7.416082651 cg05598246 6
29624414 p GABBR1/MOG MOG NA TSS1500 open
sea TSS1500 - open sea NA 0.002622306 0.003140427
0.000518121 0.83501584
-0.000774454 -0.013001089 -0.069918402 0.944692088
0.992049348 -7.416140465 cg14317321 6
29700690 p MOG LOC285830 NA Body open
sea Body - open sea NA -0.012734871 -0.013509325
-0.000774454 0.942672657
-0.001535686 0.175661576 -0.068995094 0.945421255
0.992049348 -7.416206348 cg25583651 6
29570040 p GABBR1/MOG GABBR1 NA 3'UTR open
sea 3'UTR - open sea NA 0.176189468 0.174653782
-0.001535686 1.008792741
0.001186844 -0.012273427 0.068521835 0.945795022
0.992049348 -7.416239779 cg18132851 6
152085641 q ESR1 ESR1 NA 5'UTR open
sea 5'UTR - open sea NA -0.012681404 -0.01149456
0.001186844 1.103252661
-0.000929638 -0.007079737 -0.068436465 0.945862446
0.992049348 -7.416245785 cg01089319 2
171676809 q GAD1 GAD1 NA Body island
Body - island NA -0.006760174 -0.007689812
-0.000929638 0.87910787
0.000396656 -0.016432909 0.068121676 0.946111066
0.992049348 -7.416267866 cg11950383 21
34400072 q OLIG2 OLIG2 NA Body island
Body - island NA -0.01656926 -0.016172604
0.000396656 1.024526444
0.000694635 0.006431062 0.067651832 0.946482159
0.992049348 -7.416300634 cg17930583 6
29591082 p GABBR1/MOG GABBR1 NA Body open
sea Body - open sea NA 0.006192281 0.006886916
0.000694635 0.899136953
0.000194845 0.000516801 0.067334427 0.946732859
0.992049348 -7.416322643 cg13293756 2
172778961 q SLC25A12 HAT1 NA 1stExon island
1stExon - island NA 0.000449823 0.000644668
0.000194845 0.697758549
-0.000853255 0.062236891 -0.06642549 0.947450807
0.992049348 -7.416385095 cg27322282 18 3411906
p DLGAP1 TGIF1 NA TSS200 open sea TSS200
- open sea NA 0.062530197 0.061676942 -0.000853255
1.013834264

-0.000749058 -0.022606991 -0.065244459 0.948383742
0.992049348 -7.416464975 cg17745803 6
29631321 p GABBR1/MOG MOG NA Body open
sea Body - open sea NA -0.022349502 -0.02309856
-0.000749058 0.967571213
-0.000202368 -0.000414418 -0.064143976 0.949253116
0.992049348 -7.416538118 cg19862860 22
20008420 q COMT C22orf25 NA TSS1500 island
TSS1500 - island NA -0.000344854 -0.000547222
-0.000202368 0.630189605
0.000386109 -0.00293907 0.063582639 0.949696593
0.992049348 -7.416574948 cg10580691 18 3451079
p DLGAP1 TGIF1 NA 5'UTR island 5'UTR -
island NA -0.003071794 -0.002685686 0.000386109
1.143765426
0.001466038 0.006420688 0.062431046 0.950606445
0.992049348 -7.416649492 cg27390206 17
28585657 q SLC6A4 BLMH NA Body open
sea Body - open sea NA 0.005916738 0.007382776
0.001466038 0.801424539
-0.000420678 0.003081355 -0.059136292 0.953209936
0.992049348 -7.416855241 cg02219071 6
29596540 p GABBR1/MOG GABBR1 NA TSS1500 shore
TSS1500 - shore NA 0.003225963 0.002805284
-0.000420678 1.149959288
0.000245508 -0.006257572 0.057887527 0.95419684
0.992049348 -7.41693031 cg06046431 11
27744490 p BDNF BDNF NA TSS1500 island
TSS1500 - island V$P53_01 -0.006341965 -0.006096457
0.000245508 1.040270563
-0.000235656 -0.009356854 -0.056993607 0.954903353
0.992049348 -7.416983064 cg13227691 12
72233472 q TPH2 TBC1D15 NA TSS200 island
TSS200 - island NA -0.009275847 -0.009511503
-0.000235656 0.97522409
-0.000338016 -0.012848599 -0.056461323 0.955324063
0.992049348 -7.417014086 cg25725890 17
28563054 q SLC6A4 SLC6A4 NA TSS200 island
TSS200 - island NA -0.012732406 -0.013070423
-0.000338016 0.97413883
0.000332947 -0.007604111 0.056353583 0.955409221
0.992049348 -7.41702033 cg06613392 6
29521595 p GABBR1 UBD -1794 IGR island
IGR - island NA -0.007718561 -0.007385614
0.000332947 1.045080504
0.000228326 -0.005840846 0.05586849 0.955792646
0.992049348 -7.417048294 cg03770593 2
172750719 q SLC25A12 SLC25A12 NA 1stExon island
1stExon - island V$ER_Q6 -0.005919333 -0.005691007
0.000228326 1.040120425
-0.000539733 -0.00296945 -0.055129725 0.956376598
0.992049348 -7.417090418 cg16902425 6
29526459 p GABBR1/MOG UBD NA Body open
sea Body - open sea NA -0.002783917 -0.00332365
-0.000539733 0.837608374
-0.000565257 -0.029083207 -0.055119829 0.95638442
0.992049348 -7.417090979 cg21348357 2

171574592 q GAD1 SP5 2735 IGR shore
IGR - shore NA -0.0288889 -0.029454157 -
0.000565257 0.980808935
-0.000283032 0.004717923 -0.054963571 0.956507937
0.992049348 -7.417099815 cg01636003 11
27723385 p BDNF BDNF NA TSS1500 shore
TSS1500 - shore NA 0.004815215 0.004532183
-0.000283032 1.062449303
0.000791646 0.003171353 0.054948727 0.956519671
0.992049348 -7.417100653 cg08863440 2
171680337 q GAD1 GAD1 NA Body island
Body - island NA 0.002899224 0.003690871
0.000791646 0.785512317
0.001132149 0.079379964 0.05311578 0.957968632
0.992049348 -7.417202405 cg18731680 22
19953712 q COMT COMT NA Body open
sea Body - open sea NA 0.078990787 0.080122936
0.001132149 0.985869853
-0.000445751 -0.030664195 -0.051345891 0.959367883
0.992049348 -7.417297382 cg00594408 6
29595349 p GABBR1/MOG GABBR1 NA Body island
Body - island NA -0.030510969 -0.030956719
-0.000445751 0.985600837
-0.00022411 0.004436185 -0.049539009 0.960796515
0.992049348 -7.417391024 cg16728223 6
29691603 p GABBR1/MOG HLA-F NA Body island
Body - island NA 0.004513223 0.004289113
-0.00022411 1.052250935
0.000538109 -0.039497053 0.048102348 0.961932523
0.992049348 -7.417463085 cg01382110 18 4454189
p DLGAP1 DLGAP1-AS5 189587 IGR island IGR -
island NA -0.039682028 -0.039143918 0.000538109
1.013746945
0.001085637 0.004829681 0.046702845 0.963039226
0.992049348 -7.417531245 cg07989678 2
172543677 q SLC25A12 DYNC1I2 NA TSS1500 shore
TSS1500 - shore NA 0.004456493 0.00554213
0.001085637 0.804111932
-0.000191079 0.002498229 -0.046679148 0.963057965
0.992049348 -7.417532381 cg01583365 2
172778734 q SLC25A12 HAT1 NA TSS200 island
TSS200 - island NA 0.002563912 0.002372833
-0.000191079 1.080527567
-0.000488678 0.028134388 -0.046462009 0.963229682
0.992049348 -7.417542771 cg12833048 6
29639582 p GABBR1/MOG MOG NA 3'UTR open
sea 3'UTR - open sea NA 0.028302371 0.027813693
-0.000488678 1.0175697
-0.00044394 -0.007242719 -0.043899702 0.965256126
0.992049348 -7.417661714 cg19846314 2
171680113 q GAD1 GAD1 NA Body island
Body - island NA -0.007090115 -0.007534055
-0.00044394 0.941075616
0.000153044 0.000639265 0.043418541 0.965636686
0.992049348 -7.417683297 cg16489427 6
29721015 p MOG IFITM4P 2431 IGR island

IGR - island NA 0.000586657 0.000739701
0.000153044 0.793100179
0.000241032 0.004257848 0.043286658 0.965740997
0.992049348 -7.417689171 cg21836827 6
29720549 p MOG IFITM4P 1965 IGR island
IGR - island NA 0.004174993 0.004416025
0.000241032 0.945418686
-0.000148127 0.002064381 -0.042431568 0.966417331
0.992049348 -7.417726825 cg05434863 6
29600206 p GABBR1/MOG GABBR1 NA 5'UTR island
5'UTR - island NA 0.0021153 0.001967173 -
0.000148127 1.075299363
0.000281528 0.011228607 0.041265408 0.967339746
0.992049348 -7.417776966 cg03741824 21
34350407 q OLIG2 OLIG2 -47809 IGR shore
IGR - shore NA 0.011131831 0.011413359
0.000281528 0.975333453
0.000320619 0.014303068 0.04067347 0.967807978
0.992049348 -7.417801883 cg22310628 6
29692995 p GABBR1/MOG HLA-F NA Body shore
Body - shore NA 0.014192856 0.014513474
0.000320619 0.977908901
-0.000208144 -0.015972625 -0.039023375 0.969113288
0.992049348 -7.417869441 cg19411146 6
152128471 q ESR1 ESR1 NA TSS1500 shore
TSS1500 - shore NA -0.015901076 -0.016109219
-0.000208144 0.987079225
-0.000282591 -0.012084567 -0.038518737 0.969512501
0.992049348 -7.417889544 cg25654517 6
29627131 p GABBR1/MOG MOG NA Body open
sea Body - open sea NA -0.011987426 -0.012270017
-0.000282591 0.976969012
-0.000250243 -0.003334557 -0.038206509 0.969759504
0.992049348 -7.41790185 cg24065044 11
27723409 p BDNF BDNF NA TSS1500 shore
TSS1500 - shore NA -0.003248536 -0.003498778
-0.000250243 0.928477173
0.000435758 0.042507723 0.037563574 0.970268139
0.992049348 -7.417926876 cg06085713 18 3453726
p DLGAP1 TGIF1 NA 5'UTR shore 5'UTR -
shore NA 0.042357931 0.042793689 0.000435758
0.989817232
-0.000915732 0.118488253 -0.036602532 0.971028456
0.992049348 -7.417963493 cg14713146 2
171782647 q GAD1 GORASP2 -2301 IGR shelf
IGR - shelf NA 0.118803036 0.117887303
-0.000915732 1.007767862
-0.000128845 0.001548337 -0.036550105 0.971069934
0.992049348 -7.417965463 cg15914769 11
27722774 p BDNF BDNF NA TSS200 shore
TSS200 - shore NA 0.001592628 0.001463783
-0.000128845 1.088022156
-0.000491243 -0.09892745 -0.035744911 0.971706977
0.992049348 -7.417995368 cg11182965 22
19864308 q COMT TXNRD2 NA 3'UTR open
sea 3'UTR - open sea NA -0.098758586 -0.099249829
-0.000491243 0.995050439

-0.000270304 0.018712346 -0.035099897 0.972217304
0.992049348 -7.418018843 cg14026788 12
72233266 q TPH2 TBC1D15 NA TSS1500 shore
TSS1500 - shore NA 0.018805263 0.018534959
-0.000270304 1.014583487
0.000194611 -0.004443165 0.034254176 0.972886446
0.992049348 -7.418048976 cg02365078 12
72234032 q TPH2 TBC1D15 NA Body shore
Body - shore NA -0.004510063 -0.004315451
0.000194611 1.045096415
9.33E-05 -0.001818051 0.032572276 0.97421724 0.992049348
-7.418106717 cg03867475 21 34444382 q OLIG2
OLIG1 NA 1stExon island 1stExon - island NA
-0.001850127 -0.001756814 9.33E-05 1.053114783
0.000445713 -0.033080341 0.032262169 0.974462618
0.992049348 -7.418117045 cg17413943 11
27739827 p BDNF BDNF NA Body shore
Body - shore NA -0.033233555 -0.032787842
0.000445713 1.013593855
0.000121783 -0.001169623 0.031784848 0.974840314
0.992049348 -7.418132751 cg23727007 6
29716796 p MOG LOC285830 NA Body island
Body - island NA -0.001211486 -0.001089703
0.000121783 1.1117583
9.75E-05 -0.002602738 0.031714947 0.974895625
0.992049348 -7.418135031 cg21930443 17
28443747 q SLC6A4 CCDC55 NA TSS200 island
TSS200 - island NA -0.002636255 -0.002538752
9.75E-05 1.038405888
0.000298077 0.002348205 0.029727015 0.976468704
0.992049348 -7.418197779 cg18484299 2
171787393 q GAD1 GORASP2 NA Body shore
Body - shore NA 0.002245741 0.002543818
0.000298077 0.882822896
-0.000295501 -0.036485747 -0.0296397 0.9765378 0.992049348
-7.418200442 cg03564415 6 29638575 p
GABBR1/MOG MOG NA 3'UTR open sea 3'UTR - open sea
NA -0.036384169 -0.03667967 -0.000295501
0.991943741
-0.000126651 -0.005238307 -0.028906768 0.977117806
0.992049348 -7.418222487 cg25457956 11
27743664 p BDNF BDNF NA TSS200 island
TSS200 - island NA -0.00519477 -0.005321422
-0.000126651 0.976199701
-0.000113338 0.003610275 -0.027431752 0.978285098
0.992049348 -7.418265178 cg25122820 6
29716643 p MOG LOC285830 NA Body island
Body - island NA 0.003649235 0.003535897
-0.000113338 1.03205366
-0.000546519 -0.127521753 -0.026940764 0.978673665
0.992049348 -7.418278893 cg03127104 6
29599250 p GABBR1/MOG GABBR1 NA Body shore
Body - shore NA -0.127333887 -0.127880406
-0.000546519 0.995726328
0.000243956 0.004655489 0.026533386 0.978996067
0.992049348 -7.418290085 cg13802605 9 4495359
p SLC1A1 SLC1A1 NA Body shelf Body -

shelf NA 0.004571629 0.004815585 0.000243956
0.949340216
0.000264101 -0.009948435 0.025369921 0.979916862
0.992247422 -7.418321108 cg14528040 6
29628293 p GABBR1/MOG MOG NA Body open
sea Body - open sea NA -0.01003922 -0.009775119
0.000264101 1.027017653
0.000184499 -0.021191091 0.022277533 0.982364387
0.993990001 -7.418396804 cg00655307 6
152128743 q ESR1 ESR1 NA 5'UTR shore
5'UTR - shore NA -0.021254513 -0.021070014
0.000184499 1.008756475
-0.00018419 0.009869369 -0.019428469 0.984619485
0.995535443 -7.418457849 cg24984698 17
28548496 q SLC6A4 SLC6A4 NA Body open
sea Body - open sea NA 0.009932684 0.009748494
-0.00018419 1.018894235
-0.000202066 -0.034902396 -0.017553681 0.986103492
0.996299539 -7.418493468 cg25729445 6
29595347 p GABBR1/MOG GABBR1 NA Body island
Body - island NA -0.034832936 -0.035035002
-0.000202066 0.994232444
0.00011849 0.000657792 0.012603216 0.990022309
0.996942978 -7.418570165 cg01296653 9 4678949
p SLC1A1 CDC37L1 NA TSS1500 shore TSS1500
- shore NA 0.000617061 0.000735551 0.00011849
0.838909939
-0.000192579 -0.000783629 -0.012206596 0.990336289
0.996942978 -7.41857522 cg14486905 2
171677602 q GAD1 GAD1 NA Body shore
Body - shore NA -0.00071743 -0.000910009
-0.000192579 0.788376668
0.000133747 -0.043216368 0.011752118 0.990696072
0.996942978 -7.418580815 cg10647703 6
29708958 p MOG LOC285830 NA Body open
sea Body - open sea NA -0.043262343 -0.043128597
0.000133747 1.003101114
3.65E-05 -0.003718255 0.011518282 0.990881188
0.996942978 -7.41858361 cg04000205 18 3450626
p DLGAP1 TGIF1 NA 5'UTR island 5'UTR -
island NA -0.003730803 -0.003694298 3.65E-05
1.009881587
8.66E-05 0.009591112 0.011368032 0.991000132
0.996942978 -7.418585377 cg04957663 6
29587487 p GABBR1/MOG GABBR1 NA Body open
sea Body - open sea NA 0.009561352 0.009647925
8.66E-05 0.991026823
-5.35E-05 -0.004469195 -0.010429745 0.991742928
0.996942978 -7.418595884 cg10699871 22
19879696 q COMT TXNRD2 NA Body open
sea Body - open sea NA -0.004450802 -0.004504309
-5.35E-05 0.988121011
-8.32E-05 0.03676189 -0.009195995 0.992719639 0.996942978
-7.418608323 cg23270523 18 4450489 p DLGAP1
DLGAP1-AS5 185887 IGR shelf IGR - shelf NA
0.036790474 0.036707321 -8.32E-05 1.002265285

0.00010167 -0.017411868 0.009131753 0.992770497
0.996942978 -7.418608928 cg02965237 21
34393668 q OLIG2 OLIG2 -4548 IGR shore
IGR - shore NA -0.017446818 -0.017345147
0.00010167 1.005861607
-4.87E-05 -7.87E-05 -0.008463944 0.993299181 0.996942978
-7.418614965 cg19456996 6 29600642 p
GABBR1/MOG GABBR1 NA 5'UTR island 5'UTR - island
NA -6.20E-05 -0.000110689 -4.87E-05 0.559814238
8.95E-05 -0.071158524 0.005442982 0.995690815
0.998344451 -7.418636549 cg18342026 6
29521046 p GABBR1 UBD -2343 IGR shore
IGR - shore NA -0.071189304 -0.071099761
8.95E-05 1.001259395
1.34E-05 -0.00715339 0.004265375 0.996623115
0.998344451 -7.418642422 cg20340655 11
27723075 p BDNF BDNF NA TSS1500 shore
TSS1500 - shore NA -0.007158011 -0.007144569
1.34E-05 1.001881431
2.47E-05 0.001868359 0.003758736 0.997024217
0.998344451 -7.418644511 cg25774457 6
29600114 p GABBR1/MOG GABBR1 NA Body shore
Body - shore NA 0.001859882 0.001884541
2.47E-05 0.986915249
-1.55E-05 0.016508566 -0.003012931 0.997614667
0.998344451 -7.418647105 ch.2.171328329F 2
171620083 q GAD1 SP5 48226 IGR open
sea IGR - open sea NA 0.016513905 0.016498373
-1.55E-05 1.000941391
-2.58E-06 0.001773988 -0.000350145 0.99972279 0.99972279 -
7.418651706 cg07269000 6 29526234 p GABBR1/MOG UBD
NA Body open sea Body - open sea NA
0.001774873 0.001772297 -2.58E-06 1.001453611
