# Supplementary material for: DNA Methylation at the Neonatal State and at the Time of Diagnosis: Preliminary Support for an Association with the Estrogen Receptor 1, Gamma-Aminobutyric Acid B Receptor 1, and Myelin Oligodendrocyte Glycoprotein in Female Adolescent Patients with OCD
Source: Front Psychiatry. 2016 Mar 18;7:35. doi: 10.3389/fpsyt.2016.00035 (PMC4796012; doi:10.3389/fpsyt.2016.00035)
Supplement: Supplementary file 2 [file table_2.PDF]

logFC	AveExpr	t	P.Value	adj.P.Val	B	probeID	CHR	MAPINFO	arm	OCD_GENE(+/-100.000bp)	gene	distancetoGene	feature	cgi	feat.cgi	conserved_tfbs	Case_NEW_AVG	Ctrl_NEW_AVG	deltaBeta	Fold_Change(Case/Ctrl)
0.018608978	0.896427034	4.255065026	0.000163667	0.119191724	-0.323240448	cg10939667	6	152201611	q	ESR1	ESR1	NA	Body	open sea	Body - open sea	NA	0.889660133	0.908269111	0.018608978	0.979511603
0.021538359	0.896442668	4.233234714	0.000174257	0.119191724	-0.38469912	cg16650906	6	29697700	p	MOG	LOC285830	NA	Body	open sea	Body - open sea	NA	0.888610538	0.910148897	0.021538359	0.976335346
0.018493625	0.88556681	4.031094739	0.000310343	0.141516185	-0.94915223	cg14080521	11	27761859	p	BDNF	BDNF-AS	85417	IGR	open sea	IGR - open sea	NA	0.878841855	0.897335481	0.018493625	0.979390511
0.016156	0.068711945	3.801212236	0.000593116	0.181695403	-1.579516278	cg11286436	15	88798582	q	NTRK3	NTRK3	NA	Body	shore	Body - shore	NA	0.062837036	0.078993036	0.016156	0.795475642
0.014809963	0.186461288	3.760707673	0.000664091	0.181695403	-1.689142184	cg26741280	17	28563089	q	SLC6A4	SLC6A4	NA	TSS200	island	TSS200 - island	NA	0.181075847	0.19588581	0.014809963	0.924394917
0.015506136	0.911822444	3.537168267	0.001230869	0.239195913	-2.285381281	cg17242574	6	29625991	p	GABBR1/MOG	MOG	NA	Body	open sea	Body - open sea	NA	0.906183849	0.921689985	0.015506136	0.983176408
0.038368883	0.874350984	3.505118665	0.00134337	0.239195913	-2.36956225	cg05292788	6	29586060	p	GABBR1/MOG	GABBR1	NA	Body	open sea	Body - open sea	NA	0.860398663	0.898767546	0.038368883	0.957309448
0.048282608	0.188519138	3.463115592	0.001505905	0.239195913	-2.479357978	cg02613510	11	27723789	p	BDNF	BDNF	NA	Body	shore	Body - shore	NA	0.170961826	0.219244434	0.048282608	0.779777269
-0.024755735	0.115038858	-3.446883925	0.001573657	0.239195913	-2.521623532	cg26131286	6	29521783	p	GABBR1	UBD	-1606	IGR	island	IGR - island	NA	0.124040944	0.099285208	-0.024755735	1.24933962
0.01744555	0.869824715	3.394940243	0.001810759	0.243721944	-2.656250707	cg16472542	22	20020575	q	COMT	C22orf25	NA	5'UTR	open sea	5'UTR - open sea	NA	0.863480878	0.880926428	0.01744555	0.98019636
0.016240839	0.913520124	3.36058472	0.001986054	0.243721944	-2.744755129	cg00886856	22													

19960296 q COMT ARVCF NA Body shore
Body - shore NA 0.907614364 0.923855203
0.016240839 0.98242058
0.011374972 0.919813661 3.284751807 0.002432416
0.243721944 -2.938542853 cg00923678 6
29581105 p GABBR1/MOG GABBR1 NA Body open
sea Body - open sea V$PAX5_01 0.915677307 0.927052279
0.011374972 0.987729956
0.007346025 0.049329578 3.225095321 0.002849497
0.243721944 -3.089422309 cg11539173 22
19843009 q COMT GNB1L NA TSS1500 island
TSS1500 - island NA 0.046658297 0.054004322
0.007346025 0.863973387
0.012054734 0.919357006 3.220857969 0.002881586
0.243721944 -3.100085077 cg02887726 6
29641082 p GABBR1/MOG ZFP57 NA Body open
sea Body - open sea NA 0.914973467 0.927028201
0.012054734 0.986996368
0.007942028 0.041300086 3.211808447 0.002951276
0.243721944 -3.122832711 cg17869426 21
34442160 q OLIG2 OLIG1 NA TSS1500 shore
TSS1500 - shore NA 0.038412076 0.046354104
0.007942028 0.828666131
0.012875944 0.908297987 3.170339312 0.00329171
0.243721944 -3.22664393 cg24444839 21
34350730 q OLIG2 OLIG2 -47486 IGR island
IGR - island NA 0.903615826 0.916491769
0.012875944 0.985950836
0.063577237 0.721390188 3.146341298 0.003505476
0.243721944 -3.286392608 cg08725892 6
29629986 p GABBR1/MOG MOG NA Body open
sea Body - open sea NA 0.698271192 0.761848429
0.063577237 0.916548706
0.014652793 0.872776071 3.13925181 0.003571113
0.243721944 -3.303997112 cg19372359 6
29575128 p GABBR1/MOG GABBR1 NA Body open
sea Body - open sea NA 0.867447782 0.882100575
0.014652793 0.98338875
0.00699888 0.038901116 3.108799855 0.003866573
0.243721944 -3.379371228 cg18488157 6
29521598 p GABBR1 UBD -1791 IGR island
IGR - island NA 0.036356068 0.043354949
0.00699888 0.838567888
0.01156815 0.903850899 3.106956479 0.003885185
0.243721944 -3.383921133 cg16971273 6
29634336 p GABBR1/MOG MOG NA 3'UTR open
sea 3'UTR - open sea NA 0.899644299 0.911212449
0.01156815 0.987304662
0.012098628 0.928613067 3.092364967 0.004035537
0.243721944 -3.419884606 cg17071948 6
29590143 p GABBR1/MOG GABBR1 NA Body open
sea Body - open sea NA 0.924213566 0.936312193
0.012098628 0.987078426
0.019349496 0.843971073 3.089313599 0.00406767
0.243721944 -3.427393561 cg13821571 21
34406537 q OLIG2 OLIG2 8321 IGR island

IGR - island NA 0.836934892 0.856284388
0.019349496 0.977402956
0.022790874 0.063680091 3.083021253 0.004134699
0.243721944 -3.442865223 cg21229268 21
34442350 q OLIG2 OLIG1 NA TSS200 island
TSS200 - island NA 0.055392501 0.078183375
0.022790874 0.708494626
0.010615072 0.930285537 3.054039959 0.004457178
0.243721944 -3.51389973 cg22659852 6
152464820 q ESR1 SYNE1 NA Body open
sea Body - open sea NA 0.92642551 0.937040583
0.010615072 0.988671704
0.015535613 0.905560158 3.051353962 0.004488243
0.243721944 -3.520464388 cg02405128 6
29432580 p GABBR1 OR2H1 6350 IGR open
sea IGR - open sea NA 0.899910844 0.915446457
0.015535613 0.983029468
0.03077871 0.881030361 3.039137386 0.004632142
0.243721944 -3.550281415 cg06898502 18 3410587
p DLGAP1 TGIF1 NA TSS1500 open sea TSS1500
- open sea NA 0.869838103 0.900616814 0.03077871
0.965824854
0.021447525 0.889195892 2.967384273 0.005569438
0.267559025 -3.724042208 cg13209878 6
29586025 p GABBR1/MOG GABBR1 NA Body open
sea Body - open sea NA 0.881396792 0.902844317
0.021447525 0.976244492
0.017507093 0.901657751 2.955226311 0.005745011
0.267559025 -3.753248961 cg21167956 9 4603153
p SLC1A1 C9orf68 NA 3'UTR open sea 3'UTR -
open sea NA 0.895291535 0.912798628 0.017507093
0.980820421
0.008418841 0.056168177 2.941074296 0.005955953
0.267559025 -3.78715849 cg24809935 6
29720530 p MOG IFITM4P 1946 IGR island
IGR - island NA 0.05310678 0.061525621
0.008418841 0.863165282
0.011684934 0.920843726 2.93945798 0.005980503
0.267559025 -3.791025315 cg03732055 6
152201038 q ESR1 ESR1 NA Body open
sea Body - open sea NA 0.916594659 0.928279593
0.011684934 0.987412269
0.023286966 0.854165053 2.93406531 0.006063107
0.267559025 -3.80391761 cg22179059 6
29714945 p MOG LOC285830 NA Body shore
Body - shore NA 0.845697066 0.868984032
0.023286966 0.973202078
0.015925072 0.918825974 2.890149907 0.006776918
0.289713246 -3.90838865 cg00376979 21
34350727 q OLIG2 OLIG2 -47489 IGR island
IGR - island NA 0.913035039 0.928960111
0.015925072 0.982857098
0.018960671 0.850803655 2.828235418 0.007918301
0.328249584 -4.054080411 cg03739409 12
72335078 q TPH2 TPH2 NA Body open
sea Body - open sea NA 0.843908865 0.862869536
0.018960671 0.978026028

0.026693713 0.863276055 2.803436658 0.008424105
0.338946337 -4.111898528 cg09298147 6
29550134 p GABBR1/MOG SNORD32B 105 IGR open
sea IGR - open sea NA 0.85356925 0.880262963
0.026693713 0.969675297
0.017183372 0.89839124 2.747658884 0.009674192
0.378122703 -4.240796196 cg18971999 6
29712014 p MOG LOC285830 NA Body open
sea Body - open sea NA 0.892142741 0.909326114
0.017183372 0.981103179
0.02794972 0.392509323 2.735147312 0.009977405
0.379141397 -4.269487778 cg10216820 12
72332539 q TPH2 TPH2 NA TSS200 open
sea TSS200 - open sea NA 0.382345788 0.410295509
0.02794972 0.931879047
0.010089549 0.036498265 2.713683274 0.010518282
0.379274343 -4.31851725 cg02324737 2
172543905 q SLC25A12 DYNC1I2 NA TSS200 island
TSS200 - island NA 0.032829338 0.042918887
0.010089549 0.764915875
-0.017671802 0.098446228 -2.713020924 0.010535398
0.379274343 -4.320026352 cg21153898 6
29521788 p GABBR1 UBD -1601 IGR island
IGR - island NA 0.104872338 0.087200536
-0.017671802 1.202657034
0.017771834 0.740239194 2.672699313 0.011627549
0.391230641 -4.41145348 cg18056738 6
29715160 p MOG LOC285830 NA Body shore
Body - shore NA 0.733776709 0.751548543
0.017771834 0.976353046
0.024825953 0.526807162 2.67148563 0.011661996
0.391230641 -4.414191873 cg05489989 6
29456162 p GABBR1 MAS1L NA TSS1500 open
sea TSS1500 - open sea NA 0.517779543 0.542605496
0.024825953 0.954246772
0.012003705 0.846711319 2.669257636 0.01172548
0.391230641 -4.419216744 cg10833114 12
72246108 q TPH2 MRS2P2 NA TSS1500 open
sea TSS1500 - open sea NA 0.842346336 0.85435004
0.012003705 0.985949899
0.062699216 0.598119123 2.651859586 0.012232346
0.39842497 -4.458362294 cg21013431 18 3495888 p
DLGAP1 DLGAP1-AS1 -142 IGR shelf IGR - shelf
NA 0.575319409 0.638018624 0.062699216
0.901728237
0.007428909 0.071606374 2.605012274 0.013699807
0.435845019 -4.562941403 cg23947039 11
27722037 p BDNF BDNF NA Body island
Body - island NA 0.068904952 0.076333861
0.007428909 0.902678721
0.016512199 0.273690607 2.593817604 0.014073766
0.437566187 -4.58775101 cg10262357 6
29691832 p GABBR1/MOG HLA-F NA Body island
Body - island NA 0.267686171 0.28419837
0.016512199 0.941899037
0.006734145 0.056536163 2.560457709 0.015244731
0.441546963 -4.661263913 cg12217400 6

29521604 p GABBR1 UBD -1785 IGR island
IGR - island NA 0.054087383 0.060821528
0.006734145 0.88928024
-0.009556386 0.121900497 -2.551919086 0.015558541
0.441546963 -4.679978155 cg11215918 21
34395699 q OLIG2 OLIG2 -2517 IGR island
IGR - island NA 0.125375547 0.115819161
-0.009556386 1.082511269
0.007204392 0.928108765 2.550999039 0.015592706
0.441546963 -4.681992147 cg12209876 6
152381560 q ESR1 ESR1 NA Body open
sea Body - open sea NA 0.925488986 0.932693378
0.007204392 0.992275712
0.018159069 0.339368507 2.539945907 0.016008594
0.441546963 -4.70614956 cg11742207 15
88784902 q NTRK3 NTRK3 NA Body open
sea Body - open sea NA 0.332765209 0.350924278
0.018159069 0.948253597
0.013267043 0.665539114 2.536873773 0.016125985
0.441546963 -4.712851441 cg15437231 12
72259306 q TPH2 TBC1D15 NA Body open
sea Body - open sea NA 0.660714735 0.673981778
0.013267043 0.980315428
0.051833764 0.499283682 2.536549747 0.016138412
0.441546963 -4.713557989 cg02754084 12
72338080 q TPH2 TPH2 NA Body open
sea Body - open sea NA 0.480435041 0.532268805
0.051833764 0.902617318
0.016962072 0.824996839 2.513929399 0.017028055
0.446911351 -4.762732006 cg13369999 6
29711465 p MOG LOC285830 NA Body open
sea Body - open sea NA 0.818828813 0.835790885
0.016962072 0.979705364
0.007921574 0.075941058 2.510027227 0.017186002
0.446911351 -4.77118479 cg15961225 2
171626884 q GAD1 GAD1 -46316 IGR shore
IGR - shore NA 0.073060486 0.08098206
0.007921574 0.902181125
0.013883309 0.091617642 2.506268383 0.017339417
0.446911351 -4.779318691 ch.2.3493243F 2
172737063 q SLC25A12 SLC25A12 NA Body open
sea Body - open sea NA 0.086569167 0.100452475
0.013883309 0.861792275
0.062728701 0.734847291 2.498961633 0.017641238
0.446911351 -4.795106374 cg15841167 6
29633622 p GABBR1/MOG MOG NA 3'UTR open
sea 3'UTR - open sea NA 0.712036854 0.774765555
0.062728701 0.919035248
0.00601518 0.030181736 2.490722429 0.017987347
0.447394379 -4.812871215 cg00414306 2
172779015 q SLC25A12 HAT1 NA 1stExon island
1stExon - island NA 0.027994398 0.034009578
0.00601518 0.823132766
0.013392291 0.917150107 2.4675827 0.01899292 0.462732509
-4.862549259 cg07854670 2 171667934 q GAD1
GAD1 -5266 IGR shelf IGR - shelf NA

0.912280183 0.925672475 0.013392291
0.985532364
0.014276514 0.902026151 2.461170712 0.019280521
0.462732509 -4.876258731 cg13599596 9 4541807
p SLC1A1 SLC1A1 NA Body open sea Body -
open sea NA 0.896834692 0.911111206 0.014276514
0.984330657
0.021702804 0.851916166 2.446292606 0.019963254
0.47021722 -4.907974967 cg08698936 6 29689809 p
GABBR1/MOG HLA-F NA TSS1500 shore TSS1500 - shore
NA 0.844024237 0.865727041 0.021702804
0.974931124
0.011173961 0.237589137 2.434803015 0.020505519
0.47021722 -4.932376823 cg19456996 6 29600642 p
GABBR1/MOG GABBR1 NA 5'UTR island 5'UTR - island
NA 0.233525878 0.24469984 0.011173961
0.954336047
0.016501713 0.133359776 2.430061712 0.02073318
0.47021722 -4.942423302 cg10217445 21 34397784 q
OLIG2 OLIG2 NA TSS1500 island TSS1500 - island
NA 0.127359153 0.143860867 0.016501713
0.885293935
0.020394177 0.697083735 2.425235403 0.020967288
0.47021722 -4.952635935 cg23134100 6 29701494 p
MOG LOC285830 NA Body open sea Body - open sea
NA 0.689667671 0.710061848 0.020394177
0.971278309
0.006902356 0.071103917 2.408903024 0.021777497
0.476520356 -4.987090879 cg06860277 22
19930072 q COMT COMT NA 5'UTR shore
5'UTR - shore NA 0.06859397 0.075496326
0.006902356 0.908573617
0.020831024 0.811113975 2.405595277 0.021945016
0.476520356 -4.994049138 cg21026120 6
29581121 p GABBR1/MOG GABBR1 NA Body open
sea Body - open sea V$PAX5_01 0.803539058 0.824370082
0.020831024 0.97473098
0.025379899 0.87784651 2.388144685 0.02284836 0.482225377
-5.030647686 cg10600786 6 29719569 p MOG
IFITM4P NA TSS1500 shore TSS1500 - shore NA
0.868617456 0.893997355 0.025379899
0.971610767
0.008645074 0.055239882 2.382020369 0.023173315
0.482225377 -5.043447614 cg21032292 21
34395093 q OLIG2 OLIG2 -3123 IGR shore
IGR - shore NA 0.052096219 0.060741293
0.008645074 0.857673856
0.009717746 0.084003633 2.37776263 0.023401697
0.482225377 -5.05233272 cg09539438 9
87283789 q NTRK2 NTRK2 NA TSS1500 island
TSS1500 - island V$MEIS1AHOXA9_01 0.080469908
0.090187653 0.009717746 0.892249718
0.017659481 0.142466795 2.35807464 0.024484539
0.482225377 -5.093271713 cg08805037 22
20009275 q COMT C22orf25 NA 5'UTR shore
5'UTR - shore NA 0.136045165 0.153704647
0.017659481 0.88510769

0.026256166	0.783537886	2.357767678	0.024501775
0.482225377	-5.093908097	cg06122864 6
29629187 p	GABBR1/MOG MOG	NA	Body	open
sea	Body - open sea	NA	0.773990189	0.800246355
0.026256166	0.967189896
0.01014576 0.060904078	2.357484102	0.024517709
0.482225377	-5.094495945	cg26813908 17
28443598 q	SLC6A4	CCDC55	NA	TSS1500	shore
TSS1500 - shore	NA	0.05721471 0.06736047 0.01014576
0.849381098
0.01165175 0.846057159	2.351698188	0.024844861
0.482225377	-5.106479092	cg14377523 6
29572373 p	GABBR1/MOG GABBR1	NA	Body	open
sea	Body - open sea	NA	0.841820159	0.853471909
0.01165175 0.986347822
0.017560122	0.878944725	2.344864362	0.025236355
0.482225377	-5.120605584	cg24034959 6
29524905 p	GABBR1/MOG UBD	NA	Body	shelf
Body - shelf	NA	0.872559226	0.890119348
0.017560122	0.980272171
0.03318574 0.147053028	2.337451813	0.025667309
0.482225377	-5.135895252	cg18204321 18	3453798
p	DLGAP1	TGIF1	NA	5'UTR	shore	5'UTR -
shore	NA	0.134985487	0.168171226	0.03318574
0.802666962
0.006645436	0.066987238	2.325473114	0.026377819
0.482225377	-5.160530269	cg21834061 6
29521162 p	GABBR1	UBD	-2227	IGR	island
IGR - island	NA	0.064570716	0.071216151
0.006645436	0.906686406
0.008761142	0.929290343	2.32205737 0.026583654
0.482225377	-5.167538374	cg04718263 6
29554942 p	GABBR1/MOG OR2H2	NA	TSS1500	open
sea	TSS1500 - open sea	NA	0.926104474	0.934865616
0.008761142	0.990628448
0.027440515	0.158475656	2.319170527	0.026758748
0.482225377	-5.173455568	cg08863440 2
171680337 q	GAD1	GAD1	NA	Body	island
Body - island	NA	0.148497286	0.175937802
0.027440515	0.844032859
0.008568566	0.070378368	2.318287643	0.026812504
0.482225377	-5.175264171	cg08447405 17
28619272 q	SLC6A4	BLMH	NA	TSS200	island
TSS200 - island	NA	0.067262525	0.075831091
0.008568566	0.88700458
0.009697319	0.082177301	2.312897718	0.027142803
0.482225377	-5.186294792	cg23497217 11
27723214 p	BDNF	BDNF	NA	TSS1500	shore
TSS1500 - shore	NA	0.078651003	0.088348323
0.009697319	0.890237645
0.030435767	0.213726597	2.306774194	0.02752251
0.482702484	-5.198804331	cg14005211 2
171676925 q	GAD1	GAD1	NA	Body	island
Body - island	NA	0.202659045	0.233094813
0.030435767	0.869427519
0.025797652	0.855644618	2.30101672 0.027883884
0.482850034	-5.210544282	cg24764793 6

152126745 q ESR1 ESR1 NA 5'UTR shelf
5'UTR - shelf NA 0.846263653 0.872061306
0.025797652 0.970417615
0.004747638 0.025197283 2.291860049 0.02846743
0.486793057 -5.229171833 cg15395148 6
29720485 p MOG IFITM4P 1901 IGR island
IGR - island NA 0.023470869 0.028218507
0.004747638 0.831754458
0.016189157 0.928269101 2.273816639 0.029649621
0.49689295 -5.26572027 cg23148731 6 29697938 p
MOG LOC285830 NA Body open sea Body - open sea
NA 0.922382135 0.938571292 0.016189157
0.982751276
0.014207443 0.143462659 2.271799077 0.029784519
0.49689295 -5.269793968 cg05171584 6 152128535 q
ESR1 ESR1 NA TSS1500 shore TSS1500 - shore
NA 0.138296316 0.152503759 0.014207443
0.906838736
0.005752719 0.108843657 2.265826946 0.030187067
0.497474507 -5.281836978 cg19766164 6
29716939 p MOG LOC285830 NA TSS200 island
TSS200 - island NA 0.10675176 0.112504478
0.005752719 0.948866764
0.021945473 0.88630688 2.260552009 0.03054668 0.497474507
-5.292454853 cg20704819 6 29585653 p
GABBR1/MOG GABBR1 NA Body open sea Body - open sea
NA 0.878326708 0.900272181 0.021945473
0.975623513
0.007706313 0.060698922 2.253035305 0.031065774
0.499976216 -5.307553933 cg01455471 6
29600468 p GABBR1/MOG GABBR1 NA 5'UTR island
5'UTR - island NA 0.057896627 0.06560294
0.007706313 0.882530981
0.019564627 0.872864453 2.242728807 0.031790397
0.501149462 -5.328197124 cg04615964 6
29591153 p GABBR1/MOG GABBR1 NA Body open
sea Body - open sea NA 0.865750043 0.88531467
0.019564627 0.977900934
0.019006634 0.826228304 2.241590546 0.031871347
0.501149462 -5.330472727 cg07326586 6
29528119 p GABBR1/MOG UBD NA TSS1500 open
sea TSS1500 - open sea NA 0.8193168 0.838323435
0.019006634 0.977327802
0.004810459 0.046511294 2.229890665 0.032714192
0.508556984 -5.353813818 cg22841338 6
29720580 p MOG IFITM4P 1996 IGR island
IGR - island NA 0.044762036 0.049572495
0.004810459 0.902961128
0.007397652 0.948472585 2.223226137 0.033203171
0.51035886 -5.367069251 cg02117021 6 29425960 p
GABBR1 OR2H1 NA TSS1500 open sea TSS1500 - open
sea NA 0.94578253 0.953180182 0.007397652
0.992238978
0.016984589 0.117185604 2.21096513 0.034119882
0.518622209 -5.391379285 cg06684850 11
27742369 p BDNF BDNF NA Body shore

Body - shore NA 0.11100939 0.127993979
0.016984589 0.867301656
0.011209728 0.893129781 2.19850578 0.035074536
0.518632677 -5.415980571 cg01311802 6
29430435 p GABBR1 OR2H1 NA Body open
sea Body - open sea NA 0.889053517 0.900263245
0.011209728 0.987548389
0.009521172 0.02307231 2.194264633 0.035404899
0.518632677 -5.424331271 cg05720454 21
34442511 q OLIG2 OLIG1 NA 1stExon island
1stExon - island NA 0.019610066 0.029131238
0.009521172 0.673162809
0.007727577 0.936241354 2.190929392 0.035666645
0.518632677 -5.430889845 cg16527407 6
29582672 p GABBR1/MOG GABBR1 NA Body open
sea Body - open sea NA 0.933431326 0.941158904
0.007727577 0.991789295
0.014615447 0.80778701 2.188162415 0.035885102
0.518632677 -5.436325323 cg15584790 6
29692475 p GABBR1/MOG HLA-F NA Body shore
Body - shore NA 0.802472302 0.817087749
0.014615447 0.982112757
0.013295795 0.102465107 2.184812194 0.036151205
0.518632677 -5.442899682 cg01225698 11
27742355 p BDNF BDNF NA Body shore
Body - shore NA 0.097630273 0.110926068
0.013295795 0.880138229
0.026031077 0.212916934 2.178179342 0.036683237
0.518632677 -5.455893599 cg11497864 6
29717269 p MOG LOC285830 NA TSS1500 shore
TSS1500 - shore NA 0.203451087 0.229482165
0.026031077 0.886566008
0.004051474 0.042503017 2.177051507 0.036774393
0.518632677 -5.458100122 cg11308211 2
172750783 q SLC25A12 SLC25A12 NA 5'UTR island
5'UTR - island NA 0.041029754 0.045081228
0.004051474 0.910129467
0.018779443 0.827533724 2.171167648 0.037253231
0.520024693 -5.469597583 cg00433866 6
29623646 p GABBR1/MOG MOG NA TSS1500 open
sea TSS1500 - open sea NA 0.820704836 0.839484279
0.018779443 0.977629786
0.008497312 0.069253697 2.157564958 0.03838155
0.526624564 -5.496088919 cg25669230 12
72332708 q TPH2 TPH2 NA 1stExon open
sea 1stExon - open sea NA 0.066163765 0.074661077
0.008497312 0.886188194
0.009306706 0.888166421 2.156205748 0.038495948
0.526624564 -5.498729125 cg20795635 6
29578496 p GABBR1/MOG GABBR1 NA Body open
sea Body - open sea NA 0.884782164 0.89408887
0.009306706 0.989590849
0.005979367 0.059021378 2.142282343 0.039685373
0.533127888 -5.525702622 cg20294320 6
29617586 p GABBR1/MOG MOG -7172 IGR shore
IGR - shore NA 0.056847063 0.06282643
0.005979367 0.904827204

0.005535179 0.059233574 2.137915117 0.040065113
0.533127888 -5.534136015 cg17810098 22
19929066 q COMT TXNRD2 NA Body shore
Body - shore NA 0.057220781 0.06275596
0.005535179 0.911798353
0.009858958 0.899325171 2.132580219 0.040533366
0.533127888 -5.544420408 cg12083232 6
29571438 p GABBR1/MOG GABBR1 NA Body open
sea Body - open sea NA 0.895740095 0.905599053
0.009858958 0.98911333
0.012173158 0.895630713 2.127177186 0.041012539
0.533127888 -5.554816343 cg19535685 22
19845442 q COMT GNB1L 11781 IGR shelf
IGR - shelf NA 0.89120411 0.903377269
0.012173158 0.986524834
0.012423433 0.891047641 2.12534436 0.041176221
0.533127888 -5.558338336 cg11348701 2
171704223 q GAD1 GAD1 NA Body open
sea Body - open sea V$FREAC2_01 0.886530029
0.898953463 0.012423433 0.986180114
0.043068085 0.231386971 2.114492756 0.042157245
0.533127888 -5.579143803 cg11768167 6
29690889 p GABBR1/MOG HLA-F NA TSS1500 shore
TSS1500 - shore NA 0.215725849 0.258793934
0.043068085 0.833581551
0.00940945 0.946292453 2.109150341 0.042647775
0.533127888 -5.589356943 cg20806676 6
29579306 p GABBR1/MOG GABBR1 NA Body open
sea Body - open sea NA 0.942870834 0.952280284
0.00940945 0.990119033
-0.006949726 0.023861375 -2.107873689 0.042765739
0.533127888 -5.591794623 cg23253569 21
34398222 q OLIG2 OLIG2 NA TSS200 island
TSS200 - island NA 0.026388548 0.019438822
-0.006949726 1.357517858
0.011868035 0.912180914 2.106817751 0.042863527
0.533127888 -5.593810014 cg04324598 6
29602034 p GABBR1/MOG GABBR1 NA TSS1500 shore
TSS1500 - shore NA 0.907865264 0.9197333
0.011868035 0.98709622
0.013219723 0.740123448 2.105486255 0.042987115
0.533127888 -5.596350245 cg21468949 6
29430334 p GABBR1 OR2H1 NA Body open
sea Body - open sea NA 0.735316276 0.748535999
0.013219723 0.982339229
0.00673099 0.046568023 2.101701483 0.043340137
0.533127888 -5.603564167 cg21034903 2
172544336 q SLC25A12 DYNC1I2 NA 5'UTR island
5'UTR - island V$STAT3_01;V$STAT1_01 0.04412039
0.05085138 0.00673099 0.867634074
0.015614632 0.819176004 2.098424132 0.043647897
0.533127888 -5.609802929 cg14291693 11
27683959 p BDNF BDNF NA Body open
sea Body - open sea NA 0.813497956 0.829112588
0.015614632 0.981167055
0.008906654 0.917445514 2.082013618 0.045218137
0.541691238 -5.640930062 cg09519060 6

29572346 p GABBR1/MOG GABBR1 NA Body open
sea Body - open sea NA 0.914206731 0.923113385
0.008906654 0.990351506
0.024550991 0.70366998 2.067305151 0.04666763 0.541691238
-5.668669569 cg20893956 6 152126736 q ESR1
ESR1 NA 5'UTR shelf 5'UTR - shelf NA
0.694742347 0.719293338 0.024550991
0.965867902
0.015638654 0.115582743 2.066115999 0.046786587
0.541691238 -5.670905654 cg04105250 2
171679114 q GAD1 GAD1 NA Body island
Body - island NA 0.10989596 0.125534613
0.015638654 0.875423577
0.019455753 0.763414592 2.06053247 0.047348711
0.541691238 -5.681391694 cg13813710 6
29726626 p MOG IFITM4P 8042 IGR open
sea IGR - open sea NA 0.756339773 0.775795526
0.019455753 0.974921545
0.02171969 0.797060166 2.059185106 0.047485244
0.541691238 -5.683918813 cg27521571 22
19938424 q COMT COMT NA 5'UTR open
sea 5'UTR - open sea NA 0.789162097 0.810881787
0.02171969 0.973214727
0.003870719 0.037102004 2.057998564 0.047605767
0.541691238 -5.686143237 cg03202557 6
29617599 p GABBR1/MOG MOG -7159 IGR shore
IGR - shore NA 0.03569447 0.039565189
0.003870719 0.90216857
0.006709186 0.032288015 2.056826178 0.047725116
0.541691238 -5.688340154 cg11281641 2
171674855 q GAD1 GAD1 NA 5'UTR island
5'UTR - island NA 0.029848311 0.036557496
0.006709186 0.816475806
0.012780789 0.824999626 2.056651984 0.047742872
0.541691238 -5.688666492 cg10139151 6
29549352 p GABBR1/MOG SNORD32B NA TSS1500 open
sea TSS1500 - open sea NA 0.820352066 0.833132855
0.012780789 0.984659363
0.006938109 0.106091571 2.054868825 0.047924961
0.541691238 -5.692005857 cg25346576 17
28443852 q SLC6A4 CCDC55 NA 5'UTR island
5'UTR - island NA 0.103568622 0.110506731
0.006938109 0.937215508
0.005556142 0.076741496 2.051130177 0.048308721
0.541691238 -5.699000056 cg09124190 18 3448419
p DLGAP1 TGIF1 NA 5'UTR island 5'UTR -
island V$HEN1_02 0.07472108 0.080277223 0.005556142
0.930788052
0.008783744 0.904889823 2.040669261 0.049396881
0.548148159 -5.718517845 cg02404255 6
152419175 q ESR1 ESR1 NA Body open
sea Body - open sea NA 0.901695734 0.910479478
0.008783744 0.990352617
0.018937847 0.821636967 2.037052088 0.049778119
0.548148159 -5.725248727 cg00114160 6
29430096 p GABBR1 OR2H1 NA Body open

sea	Body - open sea	NA	0.814750477	0.833688324
0.018937847	0.97728426
0.020929692	0.825915906	2.028081586	0.050734734
0.548148159	-5.741901184	cg16891968	6
29698284	p	MOG	LOC285830	NA	Body	open
sea	Body - open sea	NA	0.818305109	0.839234801
0.020929692	0.975060982
0.013182967	0.810578823	2.024487261	0.051122531
0.548148159	-5.748557519	cg06308109	6
29548691	p	GABBR1/MOG	SNORD32B	NA	TSS1500	open
sea	TSS1500 - open sea	NA	0.805785016	0.818967983
0.013182967	0.983902952
0.016832006	0.876231922	2.02038111	0.051568724
0.548148159	-5.756150469	cg18356448	18	3881547
p	DLGAP1	DLGAP1	NA	TSS1500	shore	TSS1500
- shore	NA	0.870111192	0.886943198	0.016832006
0.981022453
0.011936249	0.845108598	2.016430256	0.052001256
0.548148159	-5.763444923	cg21080452	6
29589960	p	GABBR1/MOG	GABBR1	NA	Body	open
sea	Body - open sea	NA	0.840768143	0.852704393
0.011936249	0.98600189
0.009991095	0.084256523	2.01632099	0.052013263
0.548148159	-5.763646503	cg02348151	21
34442257	q	OLIG2	OLIG1	NA	TSS200	island
TSS200 - island	NA	0.080623397	0.090614492
0.009991095	0.88974065
0.045602721	0.543285895	2.015622265	0.052090103
0.548148159	-5.764935344	cg16118803	6
29629716	p	GABBR1/MOG	MOG	NA	Body	open
sea	Body - open sea	NA	0.526703087	0.572305808
0.045602721	0.920317564
-0.019595112	0.173875392	-2.009115676	0.052810425
0.55148597	-5.776920436	cg12772565	6	29618315	p
GABBR1/MOG	MOG	-6443	IGR	shore	IGR - shore
NA	0.181000887	0.161405775	-0.019595112
1.121402794
0.005638768	0.129107359	2.003184339	0.053474635
0.554191675	-5.787819559	cg14372466	18	3448468
p	DLGAP1	TGIF1	NA	5'UTR	island	5'UTR -
island	NA	0.127056897	0.132695665	0.005638768
0.957506012
0.031513101	0.654557866	1.991291337	0.054828465
0.554496005	-5.809597574	cg09313122	11
27827916	p	BDNF	BDNF-AS	151474	IGR	open
sea	IGR - open sea	NA	0.643098557	0.674611658
0.031513101	0.953287049
0.011393422	0.900327226	1.986733803	0.055355139
0.554496005	-5.817916221	cg01095157	2
171784674	q	GAD1	GORASP2	NA	TSS1500	shore
TSS1500 - shore	NA	0.896184164	0.907577586
0.011393422	0.987446338
0.012128883	0.851104058	1.985945213	0.055446715
0.554496005	-5.819354078	cg20486877	6
29594481	p	GABBR1/MOG	GABBR1	NA	Body	shore
Body - shore	NA	0.846693556	0.858822438
0.012128883	0.985877311

0.008864171 0.100933702 1.984559765 0.055607923
0.554496005 -5.821879119 cg23221504 2
171673110 q GAD1 GAD1 NA TSS200 island
TSS200 - island NA 0.097710367 0.106574538
0.008864171 0.916826559
0.014182964 0.129555667 1.982816741 0.055811317
0.554496005 -5.825053889 cg19846314 2
171680113 q GAD1 GAD1 NA Body island
Body - island NA 0.124398225 0.138581189
0.014182964 0.897655922
-0.011003683 0.091187665 -1.981750978 0.055936001
0.554496005 -5.826994008 cg27646908 12
72332608 q TPH2 TPH2 NA TSS200 open
sea TSS200 - open sea NA 0.095189005 0.084185321
-0.011003683 1.13070787
0.019492506 0.760321255 1.977471121 0.056439147
0.554818586 -5.834776814 cg24444631 6
29636366 p GABBR1/MOG MOG NA 3'UTR open
sea 3'UTR - open sea NA 0.753233071 0.772725577
0.019492506 0.974774349
0.015606856 0.813517344 1.974356142 0.056807823
0.554818586 -5.840432997 cg26718763 6
29573014 p GABBR1/MOG GABBR1 NA Body open
sea Body - open sea NA 0.807842124 0.82344898
0.015606856 0.981046967
0.028399015 0.274381293 1.971185404 0.057185249
0.554818586 -5.84618322 cg07704699 11
27742832 p BDNF BDNF NA Body shore
Body - shore NA 0.264054378 0.292453393
0.028399015 0.902893878
0.014863566 0.874083169 1.961999143 0.058291068
0.556172885 -5.862801618 cg10625096 6
29518072 p GABBR1 UBD -5317 IGR shelf
IGR - shelf NA 0.868678236 0.883541802
0.014863566 0.983177292
0.023081484 0.773233181 1.961066966 0.058404313
0.556172885 -5.864484549 cg08027810 6
29593479 p GABBR1/MOG GABBR1 NA Body shore
Body - shore NA 0.764839914 0.787921399
0.023081484 0.970705853
0.07443437 0.617408477 1.954446882 0.059214062
0.556172885 -5.876418108 cg18505691 6
29723320 p MOG IFITM4P 4736 IGR shelf
IGR - shelf NA 0.590341433 0.664775803
0.07443437 0.888030867
0.031827865 0.324001857 1.952472224 0.059457476
0.556172885 -5.879971508 cg09567915 6
29717260 p MOG LOC285830 NA TSS1500 shore
TSS1500 - shore NA 0.312428088 0.344255953
0.031827865 0.907545927
0.036260426 0.647011863 1.944617433 0.060434345
0.556172885 -5.894078041 cg20140394 18 4456154
p DLGAP1 DLGAP1-AS5 191552 IGR shore IGR -
shore NA 0.633826254 0.67008668 0.036260426
0.945886962
-0.007998353 0.049435206 -1.941408613 0.060837399
0.556172885 -5.899827838 cg05016953 17

28562813 q SLC6A4 SLC6A4 NA 1stExon island
1stExon - island NA 0.052343698 0.044345345
-0.007998353 1.180365109
0.009531902 0.07514359 1.938806486 0.061165956
0.556172885 -5.904484985 cg13663738 21
34392756 q OLIG2 OLIG2 -5460 IGR shore
IGR - shore NA 0.071677443 0.081209346
0.009531902 0.882625542
0.008761595 0.922852251 1.933927688 0.06178612
0.556172885 -5.913203424 cg09595044 6
29426132 p GABBR1 OR2H1 NA TSS200 open
sea TSS200 - open sea NA 0.919666216 0.928427811
0.008761595 0.990562977
0.014159704 0.87952826 1.928063614 0.062538725
0.556172885 -5.923659439 cg02373484 6
29631227 p GABBR1/MOG MOG NA Body open
sea Body - open sea NA 0.874379277 0.888538981
0.014159704 0.98406406
0.010302482 0.85079823 1.922319253 0.063283642
0.556172885 -5.933877483 cg04385220 6
29726705 p MOG IFITM4P 8121 IGR open
sea IGR - open sea NA 0.847051873 0.857354355
0.010302482 0.987983403
0.006427079 0.970365693 1.913946119 0.064383185
0.556172885 -5.948728021 cg25663764 22
19965534 q COMT ARVCF NA Body shore
Body - shore NA 0.968028573 0.974455652
0.006427079 0.993404442
0.017376819 0.683966025 1.913240855 0.064476547
0.556172885 -5.949976509 cg26701815 18 3446566
p DLGAP1 TGIF1 NA 5'UTR shore 5'UTR -
shore NA 0.677647181 0.695024 0.017376819
0.974998246
0.007514664 0.052170064 1.907093046 0.065295354
0.556172885 -5.960844045 cg21956337 15
88799707 q NTRK3 NTRK3 NA TSS200 island
TSS200 - island NA 0.049437459 0.056952124
0.007514664 0.868052946
0.028496358 0.430985056 1.906412781 0.065386505
0.556172885 -5.962044836 cg20720918 2
171785124 q GAD1 GORASP2 NA TSS1500 shore
TSS1500 - shore NA 0.420622744 0.449119102
0.028496358 0.936550554
0.013688471 0.873639795 1.905735043 0.065477427
0.556172885 -5.963240827 cg03150111 6
29718049 p MOG LOC285830 NA TSS1500 shore
TSS1500 - shore NA 0.868662169 0.882350641
0.013688471 0.984486358
0.041104327 0.453731225 1.900717147 0.066153999
0.556172885 -5.972085209 cg05279622 6
29629758 p GABBR1/MOG MOG NA Body open
sea Body - open sea NA 0.438784197 0.479888524
0.041104327 0.914346093
0.006285142 0.031179597 1.900311387 0.06620897
0.556172885 -5.972799572 cg05237001 6
29691245 p GABBR1/MOG HLA-F NA 1stExon island

1stExon - island NA 0.028894091 0.035179233
0.006285142 0.821339425
0.010895906 0.868402722 1.897425499 0.066601083
0.556172885 -5.977876814 cg16150863 6
29456577 p GABBR1 MAS1L NA TSS1500 open
sea TSS1500 - open sea NA 0.864440574 0.87533648
0.010895906 0.987552323
0.005020294 0.048359953 1.896910033 0.066671331
0.556172885 -5.978783038 cg05434863 6
29600206 p GABBR1/MOG GABBR1 NA 5'UTR island
5'UTR - island NA 0.046534392 0.051554686
0.005020294 0.902621965
0.007131098 0.030126132 1.894306756 0.067027081
0.556172885 -5.983356766 cg20340508 21
34442377 q OLIG2 OLIG1 NA TSS200 island
TSS200 - island NA 0.027533005 0.034664103
0.007131098 0.794280037
0.004340445 0.029146316 1.894023223 0.067065926
0.556172885 -5.983854605 cg00370229 6
29521602 p GABBR1 UBD -1787 IGR island
IGR - island NA 0.027567973 0.031908418
0.004340445 0.86397179
0.038598083 0.618521853 1.893847164 0.067090057
0.556172885 -5.984163706 cg03147503 6
29633969 p GABBR1/MOG MOG NA 3'UTR open
sea 3'UTR - open sea NA 0.604486187 0.643084269
0.038598083 0.939979745
0.007447501 0.934366315 1.893544324 0.067131581
0.556172885 -5.984695339 cg02450267 6
29627395 p GABBR1/MOG MOG NA Body open
sea Body - open sea NA 0.931658132 0.939105633
0.007447501 0.992069581
0.015612924 0.838637191 1.891117206 0.067465177
0.556172885 -5.988953654 cg26074662 22
19969472 q COMT ARVCF NA Body shore
Body - shore NA 0.832959764 0.848572687
0.015612924 0.98160096
-0.007111624 0.044809212 -1.890945613 0.067488815
0.556172885 -5.989254541 cg06087185 6
29521499 p GABBR1 UBD -1890 IGR island
IGR - island NA 0.047395257 0.040283633
-0.007111624 1.176538794
0.042518801 0.498558073 1.883339231 0.068543851
0.561484961 -6.002570334 cg17977304 6
29623992 p GABBR1/MOG MOG NA TSS1500 open
sea TSS1500 - open sea NA 0.48309669 0.525615492
0.042518801 0.919106642
0.010132063 0.911891762 1.871409262 0.070227139
0.571849564 -6.023367986 cg13254553 6
29457282 p GABBR1 MAS1L 2739 IGR open
sea IGR - open sea NA 0.908207376 0.918339439
0.010132063 0.988966974
0.014701538 0.714060443 1.866341963 0.070952789
0.574339735 -6.032169612 cg13311832 6
29549147 p GABBR1/MOG SNORD32B NA TSS1500 open
sea TSS1500 - open sea NA 0.708714429 0.723415967
0.014701538 0.979677615

0.02209968 0.297468325 1.856265395 0.0724149 0.582726961
-6.049614746 cg21114334 6 29720137 p MOG
IFITM4P NA TSS1500 shore TSS1500 - shore NA
0.289432078 0.311531757 0.02209968 0.929061232
-0.003289981 0.036766389 -1.843266464 0.074339096
0.588403357 -6.07200622 cg08319422 6
29617817 p GABBR1/MOG MOG -6941 IGR island
IGR - island NA 0.037962746 0.034672764
-0.003289981 1.094886638
0.011366106 0.134076069 1.840293055 0.074785333
0.588403357 -6.077110155 cg07671949 6
152128338 q ESR1 ESR1 NA TSS1500 shore
TSS1500 - shore V$OCT1_02 0.129942939 0.141309045
0.011366106 0.919565616
-0.005318791 0.035323538 -1.838500959 0.075055388
0.588403357 -6.080183103 cg22617773 6
29521751 p GABBR1 UBD -1638 IGR island
IGR - island NA 0.037257644 0.031938853
-0.005318791 1.166530432
0.011505924 0.849044495 1.834494062 0.07566221
0.588403357 -6.087045012 cg15400220 6
29712541 p MOG LOC285830 NA Body shelf
Body - shelf NA 0.844860523 0.856366446
0.011505924 0.986564253
0.01365427 0.169893714 1.832445789 0.075974024
0.588403357 -6.090548025 cg27657867 11
27818018 p BDNF BDNF-AS 141576 IGR open
sea IGR - open sea NA 0.164928525 0.178582795
0.01365427 0.923540955
0.014776249 0.741888632 1.830117202 0.076329842
0.588403357 -6.094526571 cg02408532 6
29556369 p GABBR1/MOG OR2H2 NA 1stExon open
sea 1stExon - open sea NA 0.73651545 0.751291699
0.014776249 0.980332208
-0.015477283 0.172686585 -1.825996943 0.076962919
0.588403357 -6.101556208 cg16713743 21
34397135 q OLIG2 OLIG2 NA TSS1500 island
TSS1500 - island NA 0.178314688 0.162837405
-0.015477283 1.095047468
0.017181942 0.883341374 1.82402763 0.077267081
0.588403357 -6.104911522 cg05913325 6
29639793 p GABBR1/MOG MOG NA 3'UTR open
sea 3'UTR - open sea NA 0.877093395 0.894275337
0.017181942 0.980786743
0.024343034 0.475556954 1.816913265 0.078374451
0.588403357 -6.117008353 cg20265360 2
172756307 q SLC25A12 HAT1 -22628 IGR open
sea IGR - open sea NA 0.466704942 0.491047976
0.024343034 0.950426363
0.013948981 0.79676933 1.815785636 0.078551205
0.588403357 -6.11892216 cg18458352 6
29711249 p MOG LOC285830 NA Body open
sea Body - open sea NA 0.791696973 0.805645955
0.013948981 0.982685965
0.008687495 0.127212376 1.815627482 0.078576022
0.588403357 -6.119190501 cg18676033 6
29691815 p GABBR1/MOG HLA-F NA Body island

Body - island NA 0.124053287 0.132740782
0.008687495 0.934552932
0.009775662 0.852359203 1.812598196 0.079052665
0.588403357 -6.124326619 cg01558785 6
29638162 p GABBR1/MOG MOG NA 3'UTR open
sea 3'UTR - open sea NA 0.848804417 0.858580079
0.009775662 0.988614152
0.003365896 0.010351532 1.811414902 0.079239517
0.588403357 -6.126330977 cg11525479 15
88799523 q NTRK3 NTRK3 NA 5'UTR island
5'UTR - island NA 0.00912757 0.012493466
0.003365896 0.730587493
-0.01464838 0.056314452 -1.808666211 0.07967501
0.588403357 -6.130982792 cg21632158 6
29521356 p GABBR1 UBD -2033 IGR island
IGR - island NA 0.061641136 0.046992756
-0.01464838 1.311715704
0.006953203 0.925629538 1.804614733 0.080320621
0.588403357 -6.137828871 cg21569150 6
29580360 p GABBR1/MOG GABBR1 NA Body open
sea Body - open sea NA 0.923101101 0.930054303
0.006953203 0.992523875
-0.013432306 0.845674881 -1.804248821 0.080379148
0.588403357 -6.138446562 cg13698224 9
87309394 q NTRK2 NTRK2 NA Body open
sea Body - open sea NA 0.850559356 0.83712705 -
0.013432306 1.01604572
-0.011794754 0.109401606 -1.803916521 0.08043233
0.588403357 -6.139007422 cg05119316 6
29716135 p MOG LOC285830 NA Body shore
Body - shore NA 0.113690608 0.101895854
-0.011794754 1.115753032
0.006380562 0.940749171 1.792253744 0.082317902
0.593013724 -6.158638346 cg06872047 6
29585579 p GABBR1/MOG GABBR1 NA Body open
sea Body - open sea NA 0.938428967 0.944809529
0.006380562 0.993246721
0.014161166 0.799062037 1.789164362 0.082823612
0.593013724 -6.163820917 cg03639249 6
29574225 p GABBR1/MOG GABBR1 NA Body open
sea Body - open sea NA 0.793912522 0.808073688
0.014161166 0.982475403
0.007396609 0.028915354 1.783805698 0.083707035
0.593013724 -6.17279287 cg01543173 21
34442534 q OLIG2 OLIG1 NA 1stExon island
1stExon - island NA 0.026225678 0.033622287
0.007396609 0.780008748
0.011095485 0.919813539 1.781633992 0.084067325
0.593013724 -6.176422629 cg11441173 6
29707887 p MOG LOC285830 NA Body open
sea Body - open sea NA 0.915778817 0.926874302
0.011095485 0.988029137
0.017397967 0.770310171 1.780968929 0.084177923
0.593013724 -6.177533479 cg01405582 6
29692365 p GABBR1/MOG HLA-F NA Body shore
Body - shore NA 0.763983637 0.781381604
0.017397967 0.977734353

0.010616506 0.863989193 1.780146259 0.084314901
0.593013724 -6.178907105 cg18656132 6
29571363 p GABBR1/MOG GABBR1 NA Body open
sea Body - open sea NA 0.860128645 0.870745151
0.010616506 0.987807562
0.07813924 0.409306207 1.779254987 0.084463513
0.593013724 -6.180394687 cg11201654 6
29690766 p GABBR1/MOG HLA-F NA TSS1500 shore
TSS1500 - shore NA 0.380891938 0.459031178
0.07813924 0.829773567
0.015688687 0.156093895 1.778721813 0.084552522
0.593013724 -6.181284291 cg03861097 21
34393589 q OLIG2 OLIG2 -4627 IGR shore
IGR - shore NA 0.150388918 0.166077605
0.015688687 0.905534
-0.03377023 0.884455679 -1.771961327 0.085688027
0.593013724 -6.192545135 cg25978138 6
29648161 p GABBR1/MOG ZFP57 7992 IGR open
sea IGR - open sea NA 0.896735763 0.862965533
-0.03377023 1.039132768
0.00397867 0.048895541 1.771031851 0.085845147
0.593013724 -6.194090585 cg13477819 6
29617973 p GABBR1/MOG MOG -6785 IGR island
IGR - island NA 0.047448752 0.051427422
0.00397867 0.922635243
0.007677577 0.930232028 1.770064496 0.086008929
0.593013724 -6.195698305 cg13497069 6
29426119 p GABBR1 OR2H1 NA TSS200 open
sea TSS200 - open sea NA 0.927440182 0.935117758
0.007677577 0.991789723
0.038065159 0.460770838 1.76855856 0.086264423
0.593013724 -6.198199691 cg20103692 6
29454672 p GABBR1 MAS1L NA 1stExon open
sea 1stExon - open sea NA 0.446928962 0.484994121
0.038065159 0.921514185
0.004553861 0.095282445 1.764786965 0.08690712
0.594444703 -6.204456661 cg07306299 6
29617902 p GABBR1/MOG MOG -6856 IGR island
IGR - island NA 0.093626496 0.098180357
0.004553861 0.953617392
0.031661197 0.324602679 1.747706519 0.089868534
0.597483226 -6.232654249 cg09740560 6
29602390 p GABBR1/MOG GABBR1 NA TSS1500 shore
TSS1500 - shore NA 0.313089516 0.344750714
0.031661197 0.908162052
0.009920094 0.87227164 1.747663726 0.089876059
0.597483226 -6.232724611 cg10441070 6
152126250 q ESR1 ESR1 NA 5'UTR shelf
5'UTR - shelf NA 0.868664333 0.878584427
0.009920094 0.988709003
0.026240999 0.84453181 1.739433667 0.091333161
0.597483226 -6.246229902 cg10665848 6
29631447 p GABBR1/MOG MOG NA Body open
sea Body - open sea NA 0.834989628 0.861230628
0.026240999 0.96953081
0.004606543 0.970017414 1.738187925 0.091555438
0.597483226 -6.248269515 cg15136600 6

29571468 p GABBR1/MOG GABBR1 NA Body open
sea Body - open sea NA 0.968342307 0.97294885
0.004606543 0.99526538
0.002986823 0.012978329 1.734180004 0.092273653
0.597483226 -6.254823315 cg17137980 22
19842443 q COMT GNB1L NA 1stExon island
1stExon - island NA 0.011892212 0.014879035
0.002986823 0.799259629
0.009256447 0.194567271 1.730685105 0.092903793
0.597483226 -6.260527943 cg00619335 2
171670134 q GAD1 GAD1 -3066 IGR island
IGR - island V$CMYB_01 0.191201291 0.200457737
0.009256447 0.953823454
0.004060542 0.018611069 1.72900835 0.093207397
0.597483226 -6.263261463 cg13550731 2
172543902 q SLC25A12 DYNC1I2 NA TSS200 island
TSS200 - island NA 0.017134508 0.02119505
0.004060542 0.808420268
-0.007481021 0.916710672 -1.727962544 0.093397179
0.597483226 -6.264965266 cg03724721 22
19939061 q COMT COMT NA 5'UTR open
sea 5'UTR - open sea NA 0.919431043 0.911950022
-0.007481021 1.008203323
0.020525645 0.619984809 1.724976829 0.093940781
0.597483226 -6.269824798 cg05255330 18 3498963
p DLGAP1 DLGAP1 NA 3'UTR island 3'UTR -
island NA 0.612520938 0.633046583 0.020525645
0.967576407
-0.01060083 0.887800154 -1.724273562 0.094069208
0.597483226 -6.27096841 cg27527874 6
29571074 p GABBR1/MOG GABBR1 NA 3'UTR open
sea 3'UTR - open sea NA 0.891655001 0.881054172
-0.01060083 1.012031983
0.004883429 0.040682882 1.719739957 0.094900654
0.597483226 -6.278331375 cg15688670 11
27723190 p BDNF BDNF NA TSS1500 shore
TSS1500 - shore NA 0.03890709 0.043790519
0.004883429 0.888482048
0.014909079 0.842249638 1.717083457 0.095390701
0.597483226 -6.282638244 cg08292919 6
29565696 p GABBR1/MOG GABBR1 -4309 IGR open
sea IGR - open sea NA 0.836828154 0.851737233
0.014909079 0.982495682
0.018406782 0.23942911 1.715716771 0.09564364 0.597483226
-6.28485183 cg22402007 9 87282823 q NTRK2
NTRK2 NA TSS1500 shore TSS1500 - shore NA
0.232735734 0.251142516 0.018406782
0.926707822
0.025866543 0.800071501 1.714501287 0.095869067
0.597483226 -6.286819279 cg19103838 6
29562688 p GABBR1/MOG OR2H2 7005 IGR open
sea IGR - open sea NA 0.790665485 0.816532029
0.025866543 0.968321458
0.009782324 0.072664603 1.713529538 0.09604961
0.597483226 -6.288391369 cg09793121 21
34398263 q OLIG2 OLIG2 NA 1stExon island

1stExon - island NA 0.069107394 0.078889718
0.009782324 0.876000013
0.008980293 0.121543199 1.70730835 0.097212212
0.597483226 -6.298438314 cg27193031 11
27721088 p BDNF BDNF NA Body shore
Body - shore NA 0.118277638 0.127257932
0.008980293 0.929432344
-0.002934264 0.043197302 -1.707165569 0.097239033
0.597483226 -6.298668541 cg26196213 6
29617956 p GABBR1/MOG MOG -6802 IGR island
IGR - island NA 0.044264307 0.041330043
-0.002934264 1.07099591
0.009965295 0.109361513 1.70627057 0.097407291
0.597483226 -6.300111309 cg05265512 18 3447583
p DLGAP1 TGIF1 NA 5'UTR shore 5'UTR -
shore NA 0.10573777 0.115703064 0.009965295
0.913871823
-0.010566667 0.087997805 -1.70485352 0.097674193
0.597483226 -6.302394346 cg12074493 17
28564117 q SLC6A4 SLC6A4 NA TSS1500 shore
TSS1500 - shore NA 0.091840229 0.081273562
-0.010566667 1.130013583
0.007386494 0.903922408 1.703336918 0.097960522
0.597483226 -6.304836015 cg16922688 6
29588173 p GABBR1/MOG GABBR1 NA Body open
sea Body - open sea NA 0.90123641 0.908622904
0.007386494 0.991870672
0.011312782 0.598257052 1.703054345 0.098013948
0.597483226 -6.305290746 cg06542928 2
171608308 q GAD1 SP5 36451 IGR open
sea IGR - open sea NA 0.594143313 0.605456095
0.011312782 0.981315273
0.019158534 0.840537966 1.702324687 0.098152018
0.597483226 -6.306464654 cg06877423 6
152200760 q ESR1 ESR1 NA Body open
sea Body - open sea NA 0.833571226 0.85272976
0.019158534 0.977532702
-0.004635825 0.050569342 -1.699907073 0.098610653
0.597483226 -6.310351219 cg17221604 6
29720989 p MOG IFITM4P 2405 IGR island
IGR - island NA 0.052255097 0.047619271
-0.004635825 1.097351889
0.015618556 0.887442639 1.699812568 0.098628617
0.597483226 -6.310503051 cg15014679 11
27695210 p BDNF BDNF NA Body open
sea Body - open sea NA 0.881763163 0.89738172
0.015618556 0.982595414
0.008803804 0.9193649 1.69919726 0.098745648 0.597483226
-6.311491438 cg14847514 12 72340376 q TPH2
TPH2 NA Body open sea Body - open sea NA
0.916163517 0.924967321 0.008803804
0.990482038
0.006006208 0.093579983 1.69749105 0.099070772
0.597483226 -6.314230601 cg05929831 6
29720460 p MOG IFITM4P 1876 IGR island
IGR - island NA 0.091395907 0.097402115
0.006006208 0.938335959

0.018591801 0.714276789 1.697108554 0.099143781
0.597483226 -6.314844346 cg16518990 12
72244947 q TPH2 TBC1D15 NA Body open
sea Body - open sea NA 0.707516134 0.726107935
0.018591801 0.974395265
-0.009552157 0.086854899 -1.693593525 0.099816814
0.598900885 -6.320479064 cg13015908 21
34481860 q OLIG2 OLIG1 39410 IGR open
sea IGR - open sea NA 0.09032841 0.080776253 -
0.009552157 1.11825452
0.011398848 0.884344402 1.689357794 0.100632902
0.598930886 -6.32725607 cg20704972 6
29574700 p GABBR1/MOG GABBR1 NA Body open
sea Body - open sea NA 0.880199366 0.891598214
0.011398848 0.987215264
0.007984739 0.895229613 1.689024027 0.100697444
0.598930886 -6.327789479 cg00786657 15
88515810 q NTRK3 NTRK3 NA Body open
sea Body - open sea NA 0.892326071 0.90031081
0.007984739 0.991131131
0.007658203 0.080523878 1.681360801 0.102188829
0.601048239 -6.34001208 cg21608605 6
152128258 q ESR1 ESR1 NA TSS1500 shore
TSS1500 - shore NA 0.077739077 0.08539728
0.007658203 0.910322636
0.010577857 0.78584493 1.678573709 0.102735782
0.601048239 -6.344445811 cg10781870 17
28648320 q SLC6A4 TMIGD1 NA Body open
sea Body - open sea NA 0.781998436 0.792576293
0.010577857 0.986653831
0.01517882 0.326576418 1.676903837 0.103064651
0.601048239 -6.347099293 cg03443455 2
171671795 q GAD1 GAD1 NA TSS1500 island
TSS1500 - island NA 0.321056847 0.336235667
0.01517882 0.954856604
-0.011757163 0.874410427 -1.673892871 0.103659852
0.601048239 -6.3518782 cg17085250 6 29591016 p
GABBR1/MOG GABBR1 NA Body open sea Body - open sea
NA 0.878685759 0.866928596 -0.011757163
1.013561859
0.008715379 0.675206196 1.672268212 0.103982196
0.601048239 -6.354453799 cg07269000 6
29526234 p GABBR1/MOG UBD NA Body open
sea Body - open sea NA 0.672036967 0.680752347
0.008715379 0.987197429
0.027158496 0.160542807 1.669108933 0.104611405
0.601048239 -6.359456225 cg04186657 6
29690893 p GABBR1/MOG HLA-F NA TSS1500 shore
TSS1500 - shore NA 0.15066699 0.177825486
0.027158496 0.847274445
0.014472607 0.686967952 1.666870061 0.105059217
0.601048239 -6.362996443 cg03085859 6
29457094 p GABBR1 MAS1L NA TSS1500 open
sea TSS1500 - open sea NA 0.681705186 0.696177793
0.014472607 0.979211335
0.008459855 0.053265943 1.664471932 0.105540646
0.601048239 -6.366784038 cg22869726 21

34398265 q OLIG2 OLIG2 NA 1stExon island
1stExon - island NA 0.050189632 0.058649487
0.008459855 0.855755686
-0.010755857 0.477773324 -1.664454802 0.105544091
0.601048239 -6.366811076 cg24302412 6
29595196 p GABBR1/MOG GABBR1 NA Body shore
Body - shore NA 0.481684545 0.470928688
-0.010755857 1.022839672
0.008901153 0.915932962 1.664017969 0.105631986
0.601048239 -6.367500507 cg00041368 18 3879131
p DLGAP1 DLGAP1 NA 1stExon shore 1stExon
- shore NA 0.91269618 0.921597332 0.008901153
0.990341604
0.007917345 0.934819415 1.661590612 0.106121497
0.601048239 -6.371328695 cg04852097 6
29431228 p GABBR1 OR2H1 NA 3'UTR open
sea 3'UTR - open sea NA 0.93194038 0.939857725
0.007917345 0.991576018
0.009844893 0.790716834 1.660580348 0.106325785
0.601048239 -6.372920594 cg13210820 6
29632605 p GABBR1/MOG MOG NA 3'UTR open
sea 3'UTR - open sea NA 0.787136873 0.796981766
0.009844893 0.987647279
0.011215744 0.063317903 1.657580373 0.10693434
0.601135924 -6.377642909 cg20927242 6
29692011 p GABBR1/MOG HLA-F NA Body island
Body - island NA 0.059239451 0.070455195
0.011215744 0.840810262
-0.012217996 0.835924272 -1.656176307 0.10722015
0.601135924 -6.379850594 cg24984698 17
28548496 q SLC6A4 SLC6A4 NA Body open
sea Body - open sea NA 0.84036718 0.828149183 -
0.012217996 1.014753377
0.006382447 0.921558845 1.647591131 0.108981536
0.608088644 -6.393315089 cg09646983 6
152125861 q ESR1 ESR1 NA 5'UTR shelf
5'UTR - shelf NA 0.919237955 0.925620402
0.006382447 0.993104682
0.018328195 0.760266554 1.639400761 0.110684169
0.608088644 -6.406105134 cg12646029 6
29427451 p GABBR1 OR2H1 NA 5'UTR open
sea 5'UTR - open sea NA 0.753601756 0.771929951
0.018328195 0.97625666
0.081082459 0.644978104 1.637928232 0.1109926
0.608088644 -6.408398895 cg18280909 6
29723301 p MOG IFITM4P 4717 IGR shelf
IGR - shelf NA 0.615493573 0.696576032
0.081082459 0.883598552
0.062651465 0.648678957 1.637028704 0.111181361
0.608088644 -6.409799232 cg24900542 6
29723315 p MOG IFITM4P 4731 IGR shelf
IGR - shelf NA 0.625896606 0.688548071
0.062651465 0.909009309
0.005310621 0.068491511 1.636358412 0.111322191
0.608088644 -6.410842282 cg22124493 6
29717032 p MOG LOC285830 NA TSS1500 island

TSS1500 - island NA 0.066560376 0.071870997
0.005310621 0.926108984
0.023728108 0.387640094 1.634377631 0.111739218
0.608088644 -6.413922481 cg20322862 18 3412088
p DLGAP1 TGIF1 NA 5'UTR open sea 5'UTR -
open sea NA 0.379011691 0.402739799 0.023728108
0.94108328
0.005868564 0.929286648 1.631503872 0.11234654
0.608088644 -6.418385665 cg18116160 6
29575145 p GABBR1/MOG GABBR1 NA Body open
sea Body - open sea NA 0.927152624 0.933021189
0.005868564 0.993710148
0.016498046 0.806947017 1.630982145 0.11245709
0.608088644 -6.419195235 cg16834011 22
19931790 q COMT COMT NA 5'UTR shelf
5'UTR - shelf NA 0.800947728 0.817445774
0.016498046 0.979817565
0.004670822 0.045496951 1.630964464 0.112460838
0.608088644 -6.419222667 cg11241206 11
27723128 p BDNF BDNF NA TSS1500 shore
TSS1500 - shore NA 0.04379847 0.048469292
0.004670822 0.903633377
-0.002987127 0.014881341 -1.626877906 0.113329881
0.608844436 -6.425556151 cg24738387 2
171627280 q GAD1 GAD1 -45920 IGR island
IGR - island NA 0.015967569 0.012980442
-0.002987127 1.230125215
0.019021007 0.787119331 1.626124325 0.113490739
0.608844436 -6.4267226 cg01644592 6 29633971 p
GABBR1/MOG MOG NA 3'UTR open sea 3'UTR - open sea
NA 0.780202602 0.799223609 0.019021007
0.976200644
0.003981142 0.026980375 1.619754553 0.114857951
0.612284077 -6.436563839 cg12941622 2
172778750 q SLC25A12 HAT1 NA TSS200 island
TSS200 - island NA 0.025532687 0.029513829
0.003981142 0.865109268
0.004695378 0.055491713 1.618971063 0.115027053
0.612284077 -6.437772053 cg11851910 21
34482086 q OLIG2 OLIG1 39636 IGR open
sea IGR - open sea NA 0.053784303 0.058479681
0.004695378 0.919709241
0.003803437 0.039951167 1.603766381 0.118349442
0.623202152 -6.461120339 cg09503780 2
171786316 q GAD1 GORASP2 NA Body island
Body - island NA 0.038568099 0.042371536
0.003803437 0.910236037
0.015581503 0.831276025 1.602026518 0.118734593
0.623202152 -6.463780078 cg03536022 6
29706879 p MOG LOC285830 NA Body open
sea Body - open sea NA 0.825610024 0.841191526
0.015581503 0.981476868
0.012656594 0.876383024 1.601400708 0.118873378
0.623202152 -6.464736152 cg10647703 6
29708958 p MOG LOC285830 NA Body open
sea Body - open sea NA 0.871780626 0.88443722
0.012656594 0.985689664

-0.003577123 0.048571661 -1.600232838 0.119132732
0.623202152 -6.466519502 cg05128992 18 3450160
p DLGAP1 TGIF1 NA 5'UTR island 5'UTR -
island NA 0.049872433 0.04629531 -0.003577123
1.077267503
0.01786233 0.837190059 1.59473897 0.12035901 0.623202152 -
6.474893761 cg23627083 2 171787727 q GAD1 GORASP2
NA Body shore Body - shore NA
0.830694667 0.848556996 0.01786233 0.978949759
0.008524247 0.749080218 1.593691242 0.120594042
0.623202152 -6.476488006 cg06087028 6
29526389 p GABBR1/MOG UBD NA Body open
sea Body - open sea NA 0.745980492 0.754504739
0.008524247 0.988702196
0.010515938 0.894020598 1.593126557 0.12072087
0.623202152 -6.477346871 cg07238832 11
27681475 p BDNF BDNF NA Body open
sea Body - open sea NA 0.890196621 0.900712559
0.010515938 0.988324868
0.003018921 0.051717777 1.593118686 0.120722639
0.623202152 -6.477358841 cg09697651 6
29716568 p MOG LOC285830 NA Body island
Body - island NA 0.050619988 0.053638909
0.003018921 0.943717703
0.011188678 0.132807055 1.588374166 0.121792593
0.626361907 -6.484564752 cg00089464 6
29717223 p MOG LOC285830 NA TSS1500 shore
TSS1500 - shore NA 0.128738445 0.139927123
0.011188678 0.920039248
0.018381883 0.892430365 1.584509064 0.122669948
0.62818012 -6.490421401 cg25252977 6 29696650 p
MOG LOC285830 NA Body open sea Body - open sea
NA 0.885746044 0.904127928 0.018381883
0.979668935
0.006115671 0.116952146 1.582363449 0.123159216
0.62818012 -6.493667291 cg27217194 6 29600139 p
GABBR1/MOG GABBR1 NA Body shore Body - shore
NA 0.114728266 0.120843937 0.006115671
0.949391991
-0.013331576 0.850541172 -1.579603149 0.123790994
0.62818012 -6.497837528 cg24058145 6 29523592 p
GABBR1 UBD NA 3'UTR shore 3'UTR - shore
NA 0.855389018 0.842057442 -0.013331576
1.015832146
0.002881989 0.036412101 1.577518892 0.12426979
0.62818012 -6.500982265 cg23399426 2 171627790 q
GAD1 GAD1 -45410 IGR island IGR - island
NA 0.035364104 0.038246094 0.002881989
0.924646161
0.009017191 0.905393428 1.573630429 0.125167089
0.62818012 -6.506839659 cg23600322 18 3457201 p
DLGAP1 TGIF1 NA Body open sea Body - open sea
NA 0.902114449 0.91113164 0.009017191
0.990103306
0.011015113 0.863365759 1.57309065 0.125292065
0.62818012 -6.507651774 cg12867320 6 29574715 p
GABBR1/MOG GABBR1 NA Body open sea Body - open sea

V$TAL1ALPHAE47_01 0.859360263 0.870375377
0.011015113 0.98734441
0.009978164 0.919959595 1.572291142 0.125477363
0.62818012 -6.508854222 cg17036562 12 72335305 q
TPH2 TPH2 NA Body open sea Body - open sea
NA 0.916331171 0.926309335 0.009978164
0.989228043
0.013135837 0.926120116 1.570816572 0.125819702
0.62818012 -6.511070571 cg25927551 6 29707293 p
MOG LOC285830 NA Body open sea Body - open sea
NA 0.921343448 0.934479285 0.013135837
0.985943148
0.013688637 0.733154138 1.566191014 0.126898524
0.630894063 -6.518011402 cg08983330 6
29718869 p MOG IFITM4P NA Body shore
Body - shore NA 0.728176452 0.741865089
0.013688637 0.981548347
0.011719359 0.695806768 1.564208507 0.127363205
0.630894063 -6.520980839 cg00028318 6
29570739 p GABBR1/MOG GABBR1 NA 3'UTR open
sea 3'UTR - open sea NA 0.691545183 0.703264542
0.011719359 0.983335774
0.017918532 0.763659482 1.561142368 0.128084607
0.630894063 -6.525566982 cg12017635 12
72343656 q TPH2 TPH2 NA Body open
sea Body - open sea NA 0.757143652 0.775062184
0.017918532 0.976881169
0.013379394 0.832048969 1.557517668 0.128941709
0.630894063 -6.530978587 cg01976913 6
29430900 p GABBR1 OR2H1 NA 3'UTR open
sea 3'UTR - open sea NA 0.827183734 0.840563129
0.013379394 0.98408282
-0.012478286 0.138763464 -1.55717458 0.129023077
0.630894063 -6.531490248 cg05218375 11
27723218 p BDNF BDNF NA TSS1500 shore
TSS1500 - shore NA 0.143301023 0.130822737
-0.012478286 1.09538316
0.007114977 0.915774579 1.555214711 0.129488687
0.630894063 -6.534411221 cg07511633 6
29455331 p GABBR1 MAS1L NA 1stExon open
sea 1stExon - open sea NA 0.913187314 0.920302291
0.007114977 0.99226887
0.00673438 0.916884343 1.553789502 0.129828134
0.630894063 -6.536533349 cg02039276 6
29719081 p MOG IFITM4P NA TSS200 shore
TSS200 - shore NA 0.914435477 0.921169858
0.00673438 0.992689317
0.003432428 0.016333099 1.552848197 0.130052723
0.630894063 -6.537934026 cg16525287 6
29720702 p MOG IFITM4P 2118 IGR island
IGR - island NA 0.015084944 0.018517371
0.003432428 0.814637456
0.011323086 0.741739892 1.545884808 0.131723959
0.633431566 -6.5482729 cg09545764 6 29715162 p
MOG LOC285830 NA Body shore Body - shore
NA 0.737622406 0.748945492 0.011323086
0.984881295

0.007319567 0.768304644 1.545832743 0.13173652
0.633431566 -6.548350052 cg18055585 15
88668892 q NTRK3 NTRK3 NA Body open
sea Body - open sea NA 0.765642984 0.77296255
0.007319567 0.990530504
-0.018995127 0.733643255 -1.54222535 0.132609196
0.633431566 -6.55369018 cg06519422 6
29599226 p GABBR1/MOG GABBR1 NA Body shore
Body - shore NA 0.740550574 0.721555447
-0.018995127 1.026325249
0.008845103 0.816092266 1.54017253 0.133107888
0.633431566 -6.556724213 cg27474149 6
29572333 p GABBR1/MOG GABBR1 NA Body open
sea Body - open sea NA 0.812875865 0.821720967
0.008845103 0.989235881
0.002959893 0.022103426 1.537193678 0.133834239
0.633431566 -6.561120687 cg19655006 2
172750994 q SLC25A12 SLC25A12 NA TSS200 island
TSS200 - island NA 0.021027102 0.023986995
0.002959893 0.87660426
0.011145141 0.923215857 1.535006121 0.134369685
0.633431566 -6.564344602 cg00521863 2
171783942 q GAD1 GORASP2 -1006 IGR shore
IGR - shore NA 0.919163079 0.93030822
0.011145141 0.988019948
0.014459272 0.689912544 1.534911748 0.134392823
0.633431566 -6.564483595 cg23270523 18 4450489
p DLGAP1 DLGAP1-AS5 185887 IGR shelf IGR -
shelf NA 0.684654627 0.6991139 0.014459272
0.979317715
0.006714846 0.079437625 1.532487712 0.134988257
0.633431566 -6.568051187 cg10002133 15
88798448 q NTRK3 NTRK3 NA Body shore
Body - shore NA 0.076995862 0.083710708
0.006714846 0.919785101
0.010871925 0.895278576 1.532386684 0.135013119
0.633431566 -6.568199771 cg09414638 6
152239860 q ESR1 ESR1 NA Body open
sea Body - open sea NA 0.891325149 0.902197074
0.010871925 0.987949501
0.018735517 0.871399943 1.531602808 0.135206153
0.633431566 -6.569352335 cg21022792 6
29589798 p GABBR1/MOG GABBR1 NA Body open
sea Body - open sea NA 0.864587027 0.883322545
0.018735517 0.978789721
0.020811428 0.362397843 1.525982955 0.136596612
0.637761655 -6.577600464 cg08805586 6
29689688 p GABBR1/MOG HLA-F NA TSS1500 shore
TSS1500 - shore NA 0.354830051 0.37564148
0.020811428 0.944597628
0.00396097 0.048992463 1.521002626 0.137838467
0.641370826 -6.584887998 cg03851143 6
29691250 p GABBR1/MOG HLA-F NA 1stExon island
1stExon - island NA 0.04755211 0.05151308 0.00396097
0.92310749
0.002428603 0.014611287 1.51186388 0.140140901
0.649873737 -6.598206575 cg18943949 22

19842501 q COMT GNB1L NA TSS200 island
TSS200 - island NA 0.013728158 0.016156762
0.002428603 0.84968498
0.01353074 0.217867967 1.504948124 0.141903764
0.655825504 -6.608239025 cg04018625 2
171608293 q GAD1 SP5 36436 IGR open
sea IGR - open sea NA 0.212947698 0.226478438
0.01353074 0.940255946
0.004333177 0.952147084 1.499592766 0.143281079
0.658165697 -6.615980342 cg15156844 18 3880020
p DLGAP1 DLGAP1 NA 1stExon island 1stExon
- island NA 0.950571383 0.95490456 0.004333177
0.995462188
0.006383825 0.926388079 1.499006584 0.143432486
0.658165697 -6.616826225 cg20408707 6
29431410 p GABBR1 OR2H1 NA 3'UTR open
sea 3'UTR - open sea NA 0.924066688 0.930450513
0.006383825 0.993138996
0.011822712 0.590039283 1.497379323 0.143853467
0.658165697 -6.619172912 cg15999796 6
29623821 p GABBR1/MOG MOG NA TSS1500 open
sea TSS1500 - open sea NA 0.585740115 0.597562827
0.011822712 0.980215115
-0.010416916 0.07189305 -1.494806413 0.144521116
0.659016289 -6.622878792 cg14843922 21
34398849 q OLIG2 OLIG2 NA 5'UTR island
5'UTR - island NA 0.07568102 0.065264103 -
0.010416916 1.159611739
-0.011350743 0.218844579 -1.490767904 0.145574083
0.661612442 -6.628684428 cg00594408 6
29595349 p GABBR1/MOG GABBR1 NA Body island
Body - island NA 0.222972122 0.211621379
-0.011350743 1.053637034
0.007610805 0.912494799 1.488268978 0.146228703
0.662386974 -6.632269941 cg26089753 6
152127821 q ESR1 ESR1 NA TSS1500 shore
TSS1500 - shore NA 0.909727234 0.917338039
0.007610805 0.99170338
0.022876846 0.29110087 1.482639651 0.147711999
0.664388983 -6.640327769 cg17099072 6
29601489 p GABBR1/MOG GABBR1 NA TSS1500 shore
TSS1500 - shore NA 0.282782017 0.305658863
0.022876846 0.925155627
0.00970066 0.864985931 1.482080806 0.147859906
0.664388983 -6.641126243 cg08196667 6
29549180 p GABBR1/MOG SNORD32B NA TSS1500 open
sea TSS1500 - open sea NA 0.861458418 0.871159078
0.00970066 0.988864651
-0.016737946 0.382712203 -1.481070267 0.148127661
0.664388983 -6.642569429 cg07601542 21
34396986 q OLIG2 OLIG2 NA TSS1500 island
TSS1500 - island NA 0.388798729 0.372060783
-0.016737946 1.044987128
0.004437675 0.940268111 1.474883766 0.149775307
0.668938309 -6.65138581 cg21372810 2
172542111 q SLC25A12 DYNC1I2 -1871 IGR shore

IGR - shore NA 0.938654411 0.943092085
0.004437675 0.995294549
0.008316145 0.922832663 1.468207399 0.151569793
0.668938309 -6.660864044 cg00040027 2
172747482 q SLC25A12 SLC25A12 NA Body shelf
Body - shelf NA 0.919808611 0.928124755
0.008316145 0.991039842
0.003889345 0.056927622 1.466789951 0.151952973
0.668938309 -6.662871502 cg14857596 12
72233493 q TPH2 TBC1D15 NA 1stExon island
1stExon - island NA 0.055513315 0.05940266
0.003889345 0.934525743
0.009317647 0.893210026 1.464485821 0.152577497
0.668938309 -6.666131094 cg05863862 6
29649807 p GABBR1/MOG ZFP57 9638 IGR open
sea IGR - open sea NA 0.88982179 0.899139438
0.009317647 0.989637149
0.018265819 0.674261905 1.462920998 0.153002799
0.668938309 -6.668342245 cg06061002 6
29638918 p GABBR1/MOG MOG NA 3'UTR open
sea 3'UTR - open sea NA 0.667619789 0.685885608
0.018265819 0.973369001
-0.008781851 0.834293062 -1.461798809 0.153308379
0.668938309 -6.669926661 cg02403412 22
19894638 q COMT TXNRD2 NA Body island
Body - island NA 0.837486462 0.828704612
-0.008781851 1.010597081
-0.019088539 0.801019403 -1.461770995 0.153315959
0.668938309 -6.669965918 cg25437807 6
29589545 p GABBR1/MOG GABBR1 NA Body open
sea Body - open sea NA 0.80796069 0.788872151 -
0.019088539 1.024197253
0.00657713 0.098329526 1.457242011 0.154554217
0.668938309 -6.67634943 cg27278787 18 3499813
p DLGAP1 DLGAP1 NA Body shore Body -
shore NA 0.095937842 0.102514972 0.00657713
0.935842249
0.014921576 0.630083672 1.456433145 0.154776201
0.668938309 -6.677487679 cg07746998 6
152126785 q ESR1 ESR1 NA 5'UTR shelf
5'UTR - shelf NA 0.624657644 0.63957922
0.014921576 0.976669699
0.013544523 0.698682411 1.45332939 0.155630343
0.668938309 -6.681850182 cg00758854 6
29576422 p GABBR1/MOG GABBR1 NA Body open
sea Body - open sea NA 0.69375713 0.707301652
0.013544523 0.980850431
0.006272159 0.143892447 1.447512337 0.157241252
0.668938309 -6.690004341 cg01292475 9
87284571 q NTRK2 NTRK2 NA TSS200 island
TSS200 - island NA 0.141611662 0.147883821
0.006272159 0.957587254
-0.006797489 0.918384268 -1.447224384 0.157321337
0.668938309 -6.690407238 cg12457376 22
19928061 q COMT COMT NA TSS1500 shore
TSS1500 - shore NA 0.920856082 0.914058593
-0.006797489 1.007436601

0.016036568 0.594127027 1.446818584 0.157434252
0.668938309 -6.690974902 cg11046380 2
172543743 q SLC25A12 DYNC1I2 NA TSS1500 shore
TSS1500 - shore NA 0.588295548 0.604332116
0.016036568 0.973463982
0.003340007 0.06136073 1.442614244 0.158607903
0.668938309 -6.696848018 cg03760316 18 3594197
p DLGAP1 DLGAP1 NA Body open sea Body -
open sea NA 0.060146182 0.063486189 0.003340007
0.947390022
0.013490701 0.831393083 1.442432307 0.158658847
0.668938309 -6.697101828 cg13801656 9 4685060
p SLC1A1 CDC37L1 NA Body open sea Body -
open sea NA 0.826487374 0.839978075 0.013490701
0.983939222
0.002810627 0.043759058 1.442337214 0.158685479
0.668938309 -6.697234477 cg17111795 17
28618907 q SLC6A4 BLMH NA 1stExon island
1stExon - island NA 0.042737012 0.045547639
0.002810627 0.938292586
0.018885335 0.771385884 1.442327697 0.158688145
0.668938309 -6.697247753 cg05866854 2
171784945 q GAD1 GORASP2 NA TSS1500 shore
TSS1500 - shore NA 0.764518489 0.783403824
0.018885335 0.975893231
0.019171589 0.866415916 1.441191678 0.159006581
0.668938309 -6.698831817 cg08289189 22
19937277 q COMT COMT NA 5'UTR open
sea 5'UTR - open sea NA 0.859444429 0.878616018
0.019171589 0.978179787
0.005485705 0.06361432 1.440547284 0.159187435
0.668938309 -6.699729872 cg20253551 6
152129400 q ESR1 ESR1 NA Body island
Body - island V$HEN1_02;V$E47_01 0.061619518
0.067105223 0.005485705 0.91825219
0.022647919 0.441263393 1.439731967 0.159416493
0.668938309 -6.700865625 cg20108357 11
27718978 p BDNF BDNF NA Body shelf
Body - shelf NA 0.433027786 0.455675705
0.022647919 0.950298164
0.008854747 0.803010573 1.439407617 0.15950769
0.668938309 -6.701317293 cg21797131 22
19970601 q COMT ARVCF NA Body shelf
Body - shelf NA 0.799790665 0.808645411
0.008854747 0.989049903
-0.004269813 0.062887319 -1.438014964 0.159899727
0.668938309 -6.703255592 cg05901579 15
88800567 q NTRK3 NTRK3 NA TSS1500 island
TSS1500 - island NA 0.064439978 0.060170165
-0.004269813 1.070962295
0.003998288 0.040757882 1.430102024 0.162141739
0.673822874 -6.71423744 cg24000444 18 3771452
p DLGAP1 DLGAP1 NA Body island Body -
island NA 0.039303959 0.043302247 0.003998288
0.907665577
0.009548024 0.875392087 1.427692422 0.162829371
0.673822874 -6.717570949 cg10477592 21

34406589 q OLIG2 OLIG2 8373 IGR shore
IGR - shore NA 0.871920078 0.881468103
0.009548024 0.989168043
0.011367743 0.835356973 1.427279017 0.162947576
0.673822874 -6.718142366 cg03049917 15
88752391 q NTRK3 NTRK3 NA Body open
sea Body - open sea NA 0.831223248 0.842590991
0.011367743 0.986508587
0.009751651 0.865385763 1.426148942 0.163271044
0.673822874 -6.719703634 cg17532626 6
29589036 p GABBR1/MOG GABBR1 NA Body open
sea Body - open sea NA 0.861839708 0.871591358
0.009751651 0.988811672
0.009170628 0.895100643 1.425245128 0.163530113
0.673822874 -6.720951521 cg20168024 6
29456611 p GABBR1 MAS1L NA TSS1500 open
sea TSS1500 - open sea NA 0.89176587 0.900936498
0.009170628 0.989821005
-0.005134531 0.091474079 -1.422196731 0.164406294
0.675332508 -6.725155264 cg23619332 11
27722060 p BDNF BDNF NA Body island
Body - island NA 0.093341182 0.08820665 -
0.005134531 1.05821026
0.005757382 0.914599403 1.41882708 0.16537911 0.675332508
-6.729792759 cg10591943 17 28447544 q SLC6A4
CCDC55 NA Body shelf Body - shelf NA
0.91250581 0.918263192 0.005757382 0.99373014
0.024718785 0.858233542 1.417954337 0.165631808
0.675332508 -6.73099229 cg27602828 18 3845348
p DLGAP1 DLGAP1 NA TSS200 open sea TSS200
- open sea NA 0.849244893 0.873963678 0.024718785
0.971716462
0.004276713 0.948312953 1.416309161 0.166108986
0.675332508 -6.733251705 cg20067272 6
29571693 p GABBR1/MOG GABBR1 NA Body open
sea Body - open sea NA 0.946757784 0.951034497
0.004276713 0.995503094
0.00523936 0.073398224 1.411813548 0.167418441
0.675332508 -6.739413956 cg20954537 11
27721668 p BDNF BDNF NA Body island
Body - island NA 0.071493002 0.076732362
0.00523936 0.931719031
0.006412919 0.059113225 1.410842585 0.16770232
0.675332508 -6.740742602 cg05238769 21
34399260 q OLIG2 OLIG2 NA Body island
Body - island NA 0.056781254 0.063194173
0.006412919 0.898520406
0.009799282 0.89750141 1.409855953 0.167991167
0.675332508 -6.742091861 cg27297993 6
29581228 p GABBR1/MOG GABBR1 NA Body open
sea Body - open sea V$PAX4_04 0.893938035 0.903737317
0.009799282 0.989156936
-0.011486203 0.223513004 -1.406918988 0.168853308
0.675332508 -6.746103329 cg25565730 6
152085565 q ESR1 ESR1 NA 5'UTR open
sea 5'UTR - open sea NA 0.227689805 0.216203602
-0.011486203 1.053126788

0.003148394 0.053706845 1.406868151 0.168868262
0.675332508 -6.7461727 cg00267325 6 29691936 p
GABBR1/MOG HLA-F NA Body island Body - island
NA 0.052561975 0.055710369 0.003148394
0.943486391
0.00222072 0.027702292 1.405443195 0.169287834
0.675332508 -6.748116251 cg04920689 15
88800510 q NTRK3 NTRK3 NA TSS1500 island
TSS1500 - island NA 0.026894757 0.029115478
0.00222072 0.923727132
0.003945657 0.050897978 1.405253392 0.169343783
0.675332508 -6.748374998 cg17016394 21
34442360 q OLIG2 OLIG1 NA TSS200 island
TSS200 - island NA 0.049463194 0.053408851
0.003945657 0.926123537
0.008254146 0.928305308 1.403638287 0.169820455
0.675332508 -6.750575526 cg05844420 2
171621820 q GAD1 SP5 49963 IGR open
sea IGR - open sea NA 0.925303801 0.933557946
0.008254146 0.991158401
0.005398292 0.080276907 1.392912604 0.173012712
0.685291982 -6.765131958 cg23268677 22
19929097 q COMT TXNRD2 NA Body island
Body - island NA 0.078313892 0.083712183
0.005398292 0.935513675
0.017749606 0.764902535 1.390761087 0.173658679
0.685291982 -6.768039965 cg11617938 6
29692281 p GABBR1/MOG HLA-F NA Body shore
Body - shore NA 0.758448132 0.776197739
0.017749606 0.977132622
0.00715383 0.090033599 1.389394082 0.174070084
0.685291982 -6.769885545 cg24650785 11
27741916 p BDNF BDNF NA Body shore
Body - shore V$CEBP_C;V$ZIC2_01 0.087432206
0.094586037 0.00715383 0.924366944
-0.011425225 0.169696808 -1.388028039 0.17448196
0.685291982 -6.771728214 cg20386586 18 3449692
p DLGAP1 TGIF1 NA 5'UTR island 5'UTR -
island NA 0.173851436 0.16242621 -0.011425225
1.070341024
-0.0093929 0.088050809 -1.38474703 0.175474327
0.685291982 -6.77614742 cg24217789 6
29717384 p MOG LOC285830 NA TSS1500 shore
TSS1500 - shore NA 0.091466409 0.082073508
-0.0093929 1.11444498
0.007367999 0.887189427 1.383927635 0.175722846
0.685291982 -6.777249614 cg21905167 22
19949585 q COMT COMT NA 5'UTR open
sea 5'UTR - open sea NA 0.884510155 0.891878153
0.007367999 0.991738784
-0.006372737 0.877528259 -1.382834027 0.17605496
0.685291982 -6.778719759 cg25635805 6
29581054 p GABBR1/MOG GABBR1 NA Body open
sea Body - open sea NA 0.879845617 0.873472881
-0.006372737 1.00729586
0.011080741 0.331073447 1.380391057 0.176798626
0.685291982 -6.782000124 cg03316098 6

29601398 p GABBR1/MOG GABBR1 NA TSS1500 shore
TSS1500 - shore NA 0.327044086 0.338124828
0.011080741 0.967228842
0.014308842 0.105402883 1.377550869 0.177666284
0.685291982 -6.785807373 cg17825311 6
29717188 p MOG LOC285830 NA TSS1500 shore
TSS1500 - shore NA 0.100199668 0.11450851
0.014308842 0.875041235
-0.009357293 0.842715826 -1.377248375 0.177758889
0.685291982 -6.786212452 cg03258475 6
29577223 p GABBR1/MOG GABBR1 NA Body open
sea Body - open sea NA 0.846118478 0.836761185
-0.009357293 1.011182752
0.01385831 0.854650469 1.376998959 0.177835273
0.685291982 -6.786546393 cg16073467 6
29586772 p GABBR1/MOG GABBR1 NA Body open
sea Body - open sea NA 0.849611083 0.863469393
0.01385831 0.983950433
0.02715322 0.383136456 1.37534169 0.178343464 0.685319828
-6.788763931 cg21156276 9 4491917 p SLC1A1
SLC1A1 NA Body shore Body - shore NA
0.373262558 0.400415778 0.02715322 0.932187437
0.0031085 0.064229196 1.364088205 0.181824222
0.696738196 -6.803758881 cg18588589 6
29521705 p GABBR1 UBD -1684 IGR island
IGR - island NA 0.063098833 0.066207333
0.0031085 0.953049007
-0.019247703 0.568372537 -1.3557149 0.184448167
0.697775019 -6.814844718 cg25642476 6
29595011 p GABBR1/MOG GABBR1 NA Body shore
Body - shore NA 0.575371702 0.556123999
-0.019247703 1.034610452
-0.003240282 0.035733056 -1.350442922 0.186115232
0.697775019 -6.821793254 cg15299832 21
34398131 q OLIG2 OLIG2 NA TSS200 island
TSS200 - island NA 0.03691134 0.033671058 -
0.003240282 1.096233448
0.014443074 0.807061776 1.349016869 0.186568164
0.697775019 -6.82366865 cg18004110 6
29589729 p GABBR1/MOG GABBR1 NA Body open
sea Body - open sea NA 0.80180975 0.816252823
0.014443074 0.982305638
0.021879632 0.415394959 1.347754486 0.186969823
0.697775019 -6.825327326 cg22298860 6
29690822 p GABBR1/MOG HLA-F NA TSS1500 shore
TSS1500 - shore NA 0.407438729 0.429318361
0.021879632 0.949036347
-0.009325075 0.453385196 -1.347286171 0.187118999
0.697775019 -6.825942303 cg11450715 2
171627404 q GAD1 GAD1 -45796 IGR island
IGR - island NA 0.456776132 0.447451057
-0.009325075 1.020840436
0.012976548 0.790751964 1.347199599 0.187146586
0.697775019 -6.826055965 cg18484299 2
171787393 q GAD1 GORASP2 NA Body shore
Body - shore NA 0.786033219 0.799009767
0.012976548 0.983759212

0.003354512 0.024268371 1.346667731 0.187316136
0.697775019 -6.826754126 cg24399395 21
34392203 q OLIG2 OLIG2 -6013 IGR island
IGR - island NA 0.023048549 0.02640306
0.003354512 0.872949916
-0.004409761 0.9185293 -1.344375321 0.188048277
0.697775019 -6.82976045 cg14111380 6
29576818 p GABBR1/MOG GABBR1 NA Body open
sea Body - open sea NA 0.920132849 0.915723088
-0.004409761 1.004815605
0.00538105 0.935564498 1.344081326 0.188142332
0.697775019 -6.830145672 cg08388004 11
27679632 p BDNF BDNF NA Body open
sea Body - open sea V$PAX5_02;V$P53_02 0.933607753
0.938988802 0.00538105 0.994269315
0.003051085 0.038330403 1.343777447 0.188239586
0.697775019 -6.830543764 cg14337085 12
72233324 q TPH2 TBC1D15 NA TSS200 island
TSS200 - island NA 0.037220917 0.040272002
0.003051085 0.92423806
-0.01811193 0.253518871 -1.342420983 0.188674189
0.697775019 -6.832319798 cg02333875 6
29618205 p GABBR1/MOG MOG -6553 IGR shore
IGR - shore NA 0.260105028 0.241993098
-0.01811193 1.074844821
0.00941915 0.844514633 1.338127288 0.190054964
0.697775019 -6.837930988 cg16912910 6
29497165 p GABBR1 LINC01015 -18 IGR open
sea IGR - open sea NA 0.841089488 0.850508638
0.00941915 0.988925274
0.007288086 0.073729738 1.33784368 0.19014644 0.697775019
-6.838301052 cg27254482 21 34398085 q OLIG2
OLIG2 NA TSS200 island TSS200 - island NA
0.071079525 0.078367612 0.007288086
0.907001288
0.007778027 0.875046547 1.336272265 0.190653909
0.697775019 -6.840350229 cg17264941 6
29698405 p MOG LOC285830 NA Body open
sea Body - open sea NA 0.872218173 0.8799962
0.007778027 0.991161295
0.004371423 0.067746935 1.335186044 0.1910053
0.697775019 -6.841765436 cg24438313 6
29717010 p MOG LOC285830 NA TSS200 island
TSS200 - island NA 0.066157326 0.07052875
0.004371423 0.938019262
0.011162543 0.095641801 1.334437328 0.191247799
0.697775019 -6.842740315 cg15893431 21
34442183 q OLIG2 OLIG1 NA TSS1500 shore
TSS1500 - shore NA 0.091582694 0.102745237
0.011162543 0.891357076
0.004307793 0.061809933 1.333671153 0.191496198
0.697775019 -6.84373742 cg04630810 9 4662735
p SLC1A1 C9orf68 NA Body island Body -
island NA 0.060243463 0.064551256 0.004307793
0.933265543
0.011887632 0.852842645 1.33343138 0.191573984
0.697775019 -6.844049356 cg16580937 21

34347126	q	OLIG2	OLIG2	-51090	IGR	shelf
IGR - shelf	NA	0.848519869	0.860407501
0.011887632	0.986183719
-0.003966045	0.066868659	-1.332125748	0.191997983
0.697775019	-6.845747057	cg24544803 6
29596840	p	GABBR1/MOG GABBR1	NA	TSS1500	shore
TSS1500 - shore	NA	0.068310857	0.064344812
-0.003966045	1.06163737
0.006901739	0.910649166	1.329424703	0.192877427
0.697775019	-6.849254461	cg02837432 12
72232889	q	TPH2	TBC1D15	NA	TSS1500	shore
TSS1500 - shore	NA	0.908139442	0.915041182
0.006901739	0.992457454
0.007162842	0.909237627	1.327469799	0.193515861
0.697775019	-6.851788989	cg03366312 21
34388564	q	OLIG2	OLIG2	-9652	IGR	shelf
IGR - shelf	NA	0.906632957	0.913795799
0.007162842	0.992161441
0.005418674	0.05737406 1.324920429	0.194350874
0.697775019	-6.855089207	cg04481212 11
27740495	p	BDNF	BDNF	NA	Body	shore
Body - shore	NA	0.055403633	0.060822307
0.005418674	0.910909759
0.011811658	0.868699384	1.324209395	0.194584257
0.697775019	-6.856008641	cg04337734 2
172734703 q	SLC25A12	SLC25A12	NA	Body	open
sea	Body - open sea	NA	0.864404236	0.876215893
0.011811658	0.986519696
0.017297416	0.746294624	1.323972991	0.194661899
0.697775019	-6.856314236	cg23717106 18	3768748
p	DLGAP1	DLGAP1	NA	Body	shelf	Body -
shelf	NA	0.740004654	0.75730207 0.017297416
0.977159159
0.007064075	0.105663725	1.3221117	0.195274036
0.697775019	-6.858718574	cg14384532 15
88800624	q	NTRK3	NTRK3	NA	TSS1500	island
TSS1500 - island	NA	0.10309497 0.110159046
0.007064075	0.935873846
0.011331324	0.733599803	1.317374861	0.196838537
0.697775019	-6.864823711	cg12833048 6
29639582	p	GABBR1/MOG MOG	NA	3'UTR	open
sea	3'UTR - open sea	NA	0.729479321	0.740810646
0.011331324	0.984704155
0.004640875	0.935204627	1.315131567	0.197582802
0.697775019	-6.867708133	cg21627017 6
29719792	p	MOG	IFITM4P	NA	TSS1500	shore
TSS1500 - shore	NA	0.933517036	0.938157911
0.004640875	0.995053205
0.008135394	0.880340037	1.314251709	0.197875304
0.697775019	-6.868838246	cg21661983 12
72353490	q	TPH2	TPH2	NA	Body	open
sea	Body - open sea	NA	0.877381712	0.885517106
0.008135394	0.990812832
0.004520626	0.090787539	1.313947111	0.197976642
0.697775019	-6.869229321	cg00528793 22
19842837	q	COMT	GNB1L	NA	TSS1500	island

TSS1500 - island NA 0.089143675 0.093664301
0.004520626 0.95173587
-0.004392822 0.060574677 -1.313928243 0.197982921
0.697775019 -6.869253543 cg27280904 21
34443601 q OLIG2 OLIG1 NA 1stExon island
1stExon - island NA 0.062172067 0.057779246
-0.004392822 1.076027662
-0.075669217 0.689531644 -1.31226102 0.198538314
0.697775019 -6.871392626 cg20228636 6
29648525 p GABBR1/MOG ZFP57 8356 IGR open
sea IGR - open sea NA 0.717047723 0.641378506
-0.075669217 1.117979035
-0.005196043 0.066710891 -1.311672577 0.198734623
0.697775019 -6.872147026 cg10523903 18 4455646
p DLGAP1 DLGAP1-AS5 191044 IGR island IGR -
island V$PAX5_01;V$AHRARNT_01 0.068600361 0.063404318
-0.005196043 1.081950933
0.011513193 0.90465156 1.310229449 0.199216691
0.697775019 -6.87399587 cg14787188 6
29636011 p GABBR1/MOG MOG NA 3'UTR open
sea 3'UTR - open sea NA 0.900464944 0.911978137
0.011513193 0.987375582
0.003393878 0.044366332 1.303873543 0.201350484
0.697775019 -6.882116833 cg02996397 6
29720477 p MOG IFITM4P 1893 IGR island
IGR - island NA 0.043132195 0.046526073
0.003393878 0.927054278
0.006310887 0.950321744 1.301842719 0.202035932
0.697775019 -6.88470412 cg14053318 22
19967559 q COMT ARVCF NA Body island
Body - island V$NRSF_01 0.948026876 0.954337763
0.006310887 0.993387156
0.008287645 0.867383375 1.301477584 0.202159362
0.697775019 -6.88516892 cg20720118 6
29523976 p GABBR1 UBD NA Body shelf
Body - shelf NA 0.864369686 0.87265733
0.008287645 0.99050298
0.003549743 0.068155857 1.301193837 0.202255319
0.697775019 -6.885530035 cg00033220 18 3451606
p DLGAP1 TGIF1 NA 5'UTR island 5'UTR -
island NA 0.066865041 0.070414784 0.003549743
0.949588101
0.008781568 0.136299428 1.299627454 0.202785661
0.697775019 -6.887522242 cg18513624 22
19974307 q COMT ARVCF NA Body island
Body - island NA 0.133106131 0.141887699
0.008781568 0.938109025
0.010036833 0.895779694 1.297786877 0.203410192
0.697775019 -6.889860416 cg23991188 6
29607758 p GABBR1/MOG MOG -17000 IGR open
sea IGR - open sea NA 0.892129937 0.90216677
0.010036833 0.988874748
0.017377729 0.824935509 1.297753963 0.203421374
0.697775019 -6.889902201 cg25804443 18 3875823
p DLGAP1 DLGAP1 NA Body shelf Body -
shelf NA 0.818616334 0.835994064 0.017377729
0.979213094

0.005382368 0.090042998 1.297540496 0.203493903
0.697775019 -6.890173178 cg18867480 11
27744816 p BDNF BDNF NA TSS1500 shore
TSS1500 - shore NA 0.088085773 0.093468141
0.005382368 0.942414945
0.007447737 0.920468168 1.297470431 0.203517714
0.697775019 -6.890262112 cg15479387 6
29712425 p MOG LOC285830 NA Body open
sea Body - open sea NA 0.9177599 0.925207637
0.007447737 0.991950199
0.004132848 0.060697131 1.294610636 0.204491375
0.697815822 -6.893888316 cg19538089 2
171673547 q GAD1 GAD1 NA 5'UTR island
5'UTR - island NA 0.059194277 0.063327125
0.004132848 0.934738108
0.013560493 0.837990359 1.29375758 0.204782497
0.697815822 -6.894968588 cg25077271 6
29614368 p GABBR1/MOG MOG -10390 IGR shelf
IGR - shelf NA 0.83305927 0.846619763
0.013560493 0.983982782
0.007479333 0.138278222 1.291477116 0.205562296
0.697815822 -6.89785331 cg18861140 18 3449789
p DLGAP1 TGIF1 NA 5'UTR island 5'UTR -
island NA 0.135558465 0.143037798 0.007479333
0.947710793
0.014800744 0.788827624 1.29082728 0.205784919
0.697815822 -6.898674493 cg15018934 6
29689744 p GABBR1/MOG HLA-F NA TSS1500 shore
TSS1500 - shore NA 0.783445535 0.798246279
0.014800744 0.981458424
0.005175152 0.92461645 1.289966437 0.206080111
0.697815822 -6.899761745 cg12710376 15
88495888 q NTRK3 NTRK3 NA Body open
sea Body - open sea NA 0.922734576 0.927909728
0.005175152 0.994422785
-0.007222739 0.899160186 -1.28681026 0.20716515
0.698362737 -6.903742418 cg25490334 6
152387590 q ESR1 ESR1 NA Body open
sea Body - open sea NA 0.901786637 0.894563898
-0.007222739 1.008074034
0.002956681 0.016997049 1.282747882 0.208568102
0.698362737 -6.908853049 cg09029526 6
29720653 p MOG IFITM4P 2069 IGR island
IGR - island NA 0.015921892 0.018878573
0.002956681 0.843384296
-0.006324652 0.04962063 -1.28204289 0.208812305
0.698362737 -6.909738469 cg20034792 6
29521781 p GABBR1 UBD -1608 IGR island
IGR - island NA 0.051920504 0.045595851
-0.006324652 1.138711152
0.006657292 0.147538273 1.281980416 0.208833956
0.698362737 -6.909816911 cg20997792 6
29595491 p GABBR1/MOG GABBR1 NA Body island
Body - island NA 0.14511744 0.151774732
0.006657292 0.95613702
0.004968096 0.09935789 1.281965468 0.208839137
0.698362737 -6.909835679 cg13227691 12

72233472 q TPH2 TBC1D15 NA TSS200 island
TSS200 - island NA 0.09755131 0.102519406
0.004968096 0.951539946
-0.058942597 0.745762007 -1.280623513 0.209304621
0.698362737 -6.911519769 cg11747594 6
29648225 p GABBR1/MOG ZFP57 8056 IGR open
sea IGR - open sea NA 0.767195679 0.708253082
-0.058942597 1.083222507
0.00400676 0.047285616 1.278994794 0.209870632
0.698547505 -6.913561592 cg25457956 11
27743664 p BDNF BDNF NA TSS200 island
TSS200 - island NA 0.045828613 0.049835372
0.00400676 0.919600099
0.005563867 0.867096775 1.275449694 0.211106627
0.699490253 -6.917997731 cg21265702 6
152201605 q ESR1 ESR1 NA Body open
sea Body - open sea NA 0.86507355 0.870637418
0.005563867 0.993609432
0.005134659 0.928033293 1.274889077 0.211302589
0.699490253 -6.918698236 cg07119172 17
28512018 q SLC6A4 CCDC55 NA Body open
sea Body - open sea NA 0.926166144 0.931300803
0.005134659 0.994486573
0.025291962 0.477836525 1.27378808 0.21168784 0.699490253
-6.920073145 cg23019585 6 29635110 p
GABBR1/MOG MOG NA 3'UTR open sea 3'UTR - open sea
NA 0.468639448 0.49393141 0.025291962
0.948794587
0.003816915 0.085636235 1.263527774 0.215303596
0.700279298 -6.932834311 cg14446129 2
172778865 q SLC25A12 HAT1 NA TSS200 island
TSS200 - island V$ELK1_01 0.084248266 0.088065181
0.003816915 0.956658069
0.008349638 0.874702888 1.262238701 0.215761141
0.700279298 -6.934430968 cg12166917 6
29571429 p GABBR1/MOG GABBR1 NA Body open
sea Body - open sea V$TAXCREB_01 0.871666656
0.880016294 0.008349638 0.990511951
0.004171129 0.101167909 1.261842566 0.215901892
0.700279298 -6.934921326 cg22839866 6
152128584 q ESR1 ESR1 NA TSS1500 shore
TSS1500 - shore NA 0.099651135 0.103822263
0.004171129 0.959824339
0.007763181 0.843123178 1.26115113 0.216147734
0.700279298 -6.935776891 cg20757073 6
29598333 p GABBR1/MOG GABBR1 NA Body shore
Body - shore NA 0.840300203 0.848063384
0.007763181 0.99084599
0.00804195 0.083222138 1.258029221 0.217260361
0.700279298 -6.939634562 cg10131972 22
19879787 q COMT TXNRD2 NA Body open
sea Body - open sea NA 0.080297792 0.088339743
0.00804195 0.908965651
-0.001963203 0.016039272 -1.257353122 0.217501885
0.700279298 -6.940468859 cg13433942 9 4679784
p SLC1A1 CDC37L1 NA 1stExon island 1stExon

- island	V$PAX5_01	0.016753164	0.014789961	-0.001963203
1.13273889
0.006663225	0.910037456	1.257035296	0.217615492
0.700279298	-6.940860911	cg20884110	6
152464870	q	ESR1	SYNE1	NA	Body	open
sea	Body - open sea	NA	0.907614465	0.91427769
0.006663225	0.992712034
0.009780805	0.781182182	1.256238977	0.217900333
0.700279298	-6.94184281	cg09963892	15
88496177	q	NTRK3	NTRK3	NA	Body	open
sea	Body - open sea	NA	0.777625525	0.787406331
0.009780805	0.987578451
0.008246327	0.055517565	1.253155278	0.219006009
0.700279298	-6.945639825	cg15814717	6
29521228	p	GABBR1	UBD	-2161	IGR	island
IGR - island	NA	0.052518901	0.060765228
0.008246327	0.864292009
0.010078231	0.74733176	1.251838384	0.21947947	0.700279298
-6.947258757	cg09755181	6	29712115	p	MOG
LOC285830	NA	Body	open sea	Body - open sea	NA
0.743666949	0.75374518	0.010078231	0.986629127
0.014979777	0.758366081	1.247880701	0.220906997
0.700279298	-6.952114848	cg11587584	6
29692372	p	GABBR1/MOG	HLA-F	NA	Body	shore
Body - shore	NA	0.75291889	0.767898667
0.014979777	0.980492508
0.0172218	0.505629785	1.246758652	0.221312982
0.700279298	-6.953489063	cg18014983	6
29618382	p	GABBR1/MOG	MOG	-6376	IGR	shore
IGR - shore	NA	0.499367312	0.516589112
0.0172218	0.96666248
0.007755481	0.902826257	1.244844025	0.222007032
0.700279298	-6.955831381	cg03881768	6
29582192	p	GABBR1/MOG	GABBR1	NA	Body	open
sea	Body - open sea	NA	0.900006082	0.907761562
0.007755481	0.991456479
-0.003961271	0.037588457	-1.244716763	0.222053222
0.700279298	-6.955986956	cg09575114	9	4666694
p	SLC1A1	C9orf68	NA	TSS200	shelf	TSS200
- shelf	NA	0.039028919	0.035067648	-0.003961271
1.112960841
-0.001965665	0.02616944	-1.243812173	0.222381753
0.700279298	-6.957092371	cg05141870	17
28444127	q	SLC6A4	MIR423	NA	Body	shore
Body - shore	NA	0.026884227	0.024918562
-0.001965665	1.078883565
0.016080028	0.879459366	1.243399546	0.222531733
0.700279298	-6.957596363	cg16438069	9	4488463
p	SLC1A1	SLC1A1	-1964	IGR	shore	IGR -
shore	NA	0.873612083	0.889692111	0.016080028
0.9819263
0.005841311	0.904310064	1.242284065	0.222937561
0.700279298	-6.958958072	cg12248981	6
29570707	p	GABBR1/MOG	GABBR1	NA	3'UTR	open
sea	3'UTR - open sea	NA	0.902185951	0.908027262
0.005841311	0.993567031

0.006637548 0.903645715 1.241566558 0.223198894
0.700279298 -6.959833371 cg11147094 15
88420438 q NTRK3 NTRK3 NA Body open
sea Body - open sea NA 0.901232061 0.90786961
0.006637548 0.992688874
0.011798526 0.717863386 1.239962896 0.223783813
0.700279298 -6.961788043 cg04351905 21
34441330 q OLIG2 OLIG1 NA TSS1500 shore
TSS1500 - shore NA 0.713573013 0.72537154
0.011798526 0.983734505
0.007014554 0.851527131 1.237936522 0.224524551
0.700279298 -6.964254661 cg00542638 22
20007500 q COMT C22orf25 NA TSS1500 shore
TSS1500 - shore NA 0.848976384 0.855990938
0.007014554 0.991805341
-0.002473171 0.020792608 -1.237048273 0.224849826
0.700279298 -6.96533473 cg27220153 6
29720768 p MOG IFITM4P 2184 IGR island
IGR - island NA 0.021691943 0.019218772
-0.002473171 1.128685173
-0.004238894 0.036656107 -1.23670511 0.224975586
0.700279298 -6.965751811 cg06570025 21
34444245 q OLIG2 OLIG1 NA 1stExon island
1stExon - island NA 0.038197523 0.033958629
-0.004238894 1.12482524
-0.007740839 0.880795579 -1.236166212 0.225173184
0.700279298 -6.966406576 cg18281744 6
29455512 p GABBR1 MAS1L NA 1stExon open
sea 1stExon - open sea NA 0.88361043 0.875869591 -
0.007740839 1.00883789
0.007187528 0.898176801 1.236112839 0.225192761
0.700279298 -6.96647141 cg23105820 6
29624956 p GABBR1/MOG MOG NA 1stExon open
sea 1stExon - open sea NA 0.895563154 0.902750682
0.007187528 0.992038192
-0.019546032 0.086643458 -1.232794471 0.226412445
0.700279298 -6.970497365 cg01426208 6
29720641 p MOG IFITM4P 2057 IGR island
IGR - island NA 0.093751106 0.074205074
-0.019546032 1.263405599
0.005505265 0.91813285 1.232725703 0.226437774
0.700279298 -6.970580692 cg07584093 6
152126180 q ESR1 ESR1 NA 5'UTR shelf
5'UTR - shelf NA 0.916130935 0.921636201
0.005505265 0.994026639
-0.012318266 0.584614095 -1.229279782 0.227709658
0.700279298 -6.974750746 cg12292060 6
29597991 p GABBR1/MOG GABBR1 NA Body shelf
Body - shelf NA 0.589093464 0.576775199
-0.012318266 1.021357134
0.007672337 0.201058558 1.228012788 0.22817864
0.700279298 -6.976281312 cg21535772 2
171679906 q GAD1 GAD1 NA Body island
Body - island NA 0.198268617 0.205940954
0.007672337 0.962744967
-0.0105928 0.599423046 -1.227634984 0.228318625
0.700279298 -6.976737432 cg21879791 6

29594830 p GABBR1/MOG GABBR1 NA Body shore
Body - shore NA 0.603274973 0.592682173
-0.0105928 1.017872648
0.011565964 0.707770244 1.227316026 0.228436856
0.700279298 -6.977122409 cg07579946 22
19949893 q COMT COMT NA 5'UTR open
sea 5'UTR - open sea NA 0.703564439 0.715130403
0.011565964 0.983826776
0.008137112 0.863574665 1.226861933 0.228605257
0.700279298 -6.977670332 cg05189570 11
27680480 p BDNF BDNF NA Body open
sea Body - open sea NA 0.860615716 0.868752827
0.008137112 0.990633572
0.005675391 0.064362248 1.226631833 0.228690625
0.700279298 -6.977947908 cg02527472 11
27743348 p BDNF BDNF NA TSS1500 shore
TSS1500 - shore NA 0.06229847 0.06797386 0.005675391
0.916506286
0.007076238 0.103079339 1.226285039 0.228819332
0.700279298 -6.978366165 cg22043168 11
27741077 p BDNF BDNF NA Body island
Body - island NA 0.100506161 0.107582399
0.007076238 0.934224947
0.006416469 0.887374571 1.22393534 0.229692808
0.701383395 -6.98119722 cg14317321 6
29700690 p MOG LOC285830 NA Body open
sea Body - open sea NA 0.885041309 0.891457778
0.006416469 0.992802274
0.014819313 0.850935602 1.222368745 0.230276549
0.701599821 -6.983081991 cg04321753 6
29586923 p GABBR1/MOG GABBR1 NA Body open
sea Body - open sea NA 0.845546761 0.860366073
0.014819313 0.982775574
-0.011675986 0.85135156 -1.218764157 0.231623875
0.704136579 -6.987410306 cg25017994 6
29627296 p GABBR1/MOG MOG NA Body open
sea Body - open sea NA 0.855597373 0.843921387
-0.011675986 1.013835395
0.002800179 0.045054332 1.2157746 0.232745748
0.705832926 -6.990991247 cg10901968 17
28563108 q SLC6A4 SLC6A4 NA TSS200 island
TSS200 - island NA 0.044036085 0.046836265
0.002800179 0.940213422
0.005763448 0.918283714 1.214530496 0.233213803
0.705832926 -6.992479089 cg02342892 6
29431208 p GABBR1 OR2H1 NA 3'UTR open
sea 3'UTR - open sea NA 0.916187914 0.921951363
0.005763448 0.993748641
0.005774722 0.913721326 1.207824436 0.235748775
0.711115409 -7.000474976 cg05183668 21
34350584 q OLIG2 OLIG2 -47632 IGR shore
IGR - shore NA 0.911621427 0.917396149
0.005774722 0.993705313
0.020960807 0.677658536 1.205513133 0.236627185
0.711115409 -7.003221446 cg05442477 21
34444584 q OLIG2 OLIG1 NA 1stExon shore

1stExon - shore NA 0.670036425 0.690997232
0.020960807 0.96966586
-0.005563681 0.088819141 -1.204283849 0.237095359
0.711115409 -7.004680215 cg05542661 6
29717068 p MOG LOC285830 NA TSS1500 island
TSS1500 - island NA 0.090842298 0.085278616
-0.005563681 1.065241232
0.00560042 0.235211479 1.202987323 0.237589884
0.711115409 -7.006217304 cg02035039 6
29691888 p GABBR1/MOG HLA-F NA Body island
Body - island NA 0.233174963 0.238775383
0.00560042 0.976545237
-0.014825483 0.856864909 -1.201403586 0.238194992
0.711115409 -7.008092838 cg03534481 21
34439545 q OLIG2 OLIG1 -2905 IGR shelf
IGR - shelf NA 0.862255993 0.847430511
-0.014825483 1.017494629
0.006369616 0.876491518 1.199213964 0.239033468
0.711115409 -7.010682163 cg19548470 18 3880510
p DLGAP1 DLGAP1 NA TSS1500 shore TSS1500
- shore NA 0.874175294 0.88054491 0.006369616
0.992766279
0.006404287 0.913278624 1.19803471 0.239485943
0.711115409 -7.012074891 cg13228862 18 3729405
p DLGAP1 DLGAP1 NA Body island Body -
island NA 0.910949793 0.917354079 0.006404287
0.993018741
-0.025185954 0.829327113 -1.195658176 0.240399729
0.711115409 -7.01487782 cg27390206 17
28585657 q SLC6A4 BLMH NA Body open
sea Body - open sea NA 0.838485642 0.813299688
-0.025185954 1.030967618
0.006929249 0.788985183 1.195145055 0.240597363
0.711115409 -7.015482336 cg12074883 6
29591155 p GABBR1/MOG GABBR1 NA Body open
sea Body - open sea NA 0.786465456 0.793394705
0.006929249 0.991266328
-0.006297684 0.0838753 -1.195138857 0.240599751
0.711115409 -7.015489637 cg12635048 6
29720957 p MOG IFITM4P 2373 IGR island
IGR - island NA 0.086165366 0.079867683
-0.006297684 1.078851455
-0.032352043 0.447550452 -1.19439547 0.240886289
0.711115409 -7.016365006 cg12463578 6
29644756 p GABBR1/MOG ZFP57 NA 1stExon open
sea 1stExon - open sea NA 0.459314831 0.426962789
-0.032352043 1.07577251
0.00300716 0.054825555 1.193427873 0.241259624
0.711115409 -7.017503643 cg21709140 2
171785487 q GAD1 GORASP2 NA TSS1500 island
TSS1500 - island NA 0.053732043 0.056739203
0.00300716 0.947000313
0.011010328 0.788997417 1.192244328 0.24171686
0.711115409 -7.018895253 cg12931591 18 3411821
p DLGAP1 TGIF1 NA TSS1500 open sea TSS1500
- open sea NA 0.784993662 0.79600399 0.011010328
0.986167999

0.005295645 0.914064633 1.187612508 0.243512394
0.712002769 -7.024329159 cg03810301 6
29590017 p GABBR1/MOG GABBR1 NA Body open
sea Body - open sea NA 0.912138944 0.917434589
0.005295645 0.994227768
0.009435698 0.835870431 1.187519667 0.243548483
0.712002769 -7.024437878 cg22561592 15
88320395 q NTRK3 NTRK3-AS1 -99593 IGR open
sea IGR - open sea NA 0.832439268 0.841874966
0.009435698 0.988792043
0.006324078 0.891425691 1.184129748 0.244868935
0.712002769 -7.028402242 cg06132455 22
19839565 q COMT C22orf29 NA Body shelf
Body - shelf NA 0.889126026 0.895450104
0.006324078 0.992937543
0.01263197 0.650677273 1.183410943 0.245149601
0.712002769 -7.029241516 cg17264271 6
152126938 q ESR1 ESR1 NA 5'UTR shore
5'UTR - shore NA 0.64608383 0.6587158 0.01263197
0.980823338
-0.004253248 0.098640922 -1.18323156 0.245219679
0.712002769 -7.02945089 cg02736969 21
34442674 q OLIG2 OLIG1 NA 1stExon island
1stExon - island NA 0.100187557 0.095934309
-0.004253248 1.044335004
0.00308566 0.076868643 1.182733631 0.245414281
0.712002769 -7.030031916 cg22215258 2
172544429 q SLC25A12 DYNC1I2 NA 5'UTR island
5'UTR - island NA 0.075746585 0.078832245
0.00308566 0.960857895
-0.004402621 0.045398401 -1.180755252 0.246188592
0.712002769 -7.032338235 cg02720618 6
152129791 q ESR1 ESR1 NA Body shore
Body - shore NA 0.046999354 0.042596733
-0.004402621 1.103355837
0.027904808 0.462542899 1.180190062 0.246410129
0.712002769 -7.032996462 cg14172108 21
34405553 q OLIG2 OLIG2 7337 IGR shore
IGR - shore NA 0.452395696 0.480300505
0.027904808 0.941901354
-0.010229756 0.334076171 -1.17908662 0.246843065
0.712002769 -7.034280708 cg18397357 17
28565258 q SLC6A4 BLMH -9955 IGR shelf
IGR - shelf NA 0.337796082 0.327566326
-0.010229756 1.031229572
0.004339277 0.124933734 1.178118963 0.247223184
0.712002769 -7.035406011 cg00118317 21
34392029 q OLIG2 OLIG2 -6187 IGR shore
IGR - shore NA 0.123355815 0.127695092
0.004339277 0.966018451
0.006620598 0.912733111 1.172340355 0.249502088
0.717056422 -7.042108371 cg24936467 6
29598417 p GABBR1/MOG GABBR1 NA Body shore
Body - shore NA 0.910325621 0.916946219
0.006620598 0.992779731
0.011995597 0.877443766 1.166743377 0.25172397
0.721598498 -7.048571175 cg18354203 11

27696004 p BDNF BDNF NA Body open
sea Body - open sea NA 0.873081731 0.885077328
0.011995597 0.986446837
-0.004231349 0.03011651 -1.1657057 0.252137487 0.721598498
-7.049766247 cg14589148 11 27743648 p BDNF
BDNF NA TSS200 island TSS200 - island NA
0.031655182 0.027423833 -0.004231349
1.154294587
-0.002584544 0.061388293 -1.160347128 0.254280781
0.726213169 -7.055922037 cg09547815 6
29691943 p GABBR1/MOG HLA-F NA Body island
Body - island NA 0.062328127 0.059743584
-0.002584544 1.043260595
-0.005977827 0.900336741 -1.156434242 0.255854194
0.729184452 -7.060400556 cg18868933 6
29708164 p MOG LOC285830 NA Body open
sea Body - open sea NA 0.902510496 0.896532669
-0.005977827 1.006667718
0.004198151 0.086656031 1.15278185 0.257329234
0.731863601 -7.064568344 cg00465250 2
171627741 q GAD1 GAD1 -45459 IGR island
IGR - island NA 0.085129431 0.089327582
0.004198151 0.953002747
0.007308 0.874407066 1.150423845 0.258284803
0.731926866 -7.067252635 cg09043524 22
19891284 q COMT TXNRD2 NA Body shelf
Body - shelf NA 0.871749612 0.879057611
0.007308 0.991686553
0.008838256 0.857515304 1.150086925 0.258421547
0.731926866 -7.067635761 cg13682912 6
29627142 p GABBR1/MOG MOG NA Body open
sea Body - open sea NA 0.854301392 0.863139649
0.008838256 0.989760339
-0.003833273 0.094355585 -1.147946027 0.259291695
0.732874046 -7.070067854 cg13555101 9 4490751
p SLC1A1 SLC1A1 NA 1stExon island 1stExon
- island NA 0.095749503 0.09191623 -0.003833273
1.041703984
-0.002348031 0.043740068 -1.144519111 0.260688947
0.73331212 -7.073952179 cg09805507 6 29691426 p
GABBR1/MOG HLA-F NA Body island Body - island
NA 0.044593898 0.042245866 -0.002348031
1.05558016
0.005888484 0.90183644 1.142109642 0.261674615
0.73331212 -7.076676834 cg20838323 6 29549759 p
GABBR1/MOG SNORD32B NA TSS1500 open sea TSS1500 - open
sea NA 0.899695173 0.905583658 0.005888484
0.99349758
0.009583175 0.849783341 1.141946234 0.26174156
0.73331212 -7.076861426 cg14093720 18 3712400 p
DLGAP1 DLGAP1 NA Body open sea Body - open sea
NA 0.84629855 0.855881725 0.009583175
0.988803155
0.003894367 0.066560276 1.141897098 0.261761692
0.73331212 -7.076916927 cg15313332 11 27721270 p
BDNF BDNF NA Body shore Body - shore

NA 0.065144143 0.06903851 0.003894367
0.943591381
0.007606474 0.820367118 1.140465205 0.262348868
0.73331212 -7.078533338 cg03160788 6 29572219 p
GABBR1/MOG GABBR1 NA Body open sea Body - open sea
NA 0.817601127 0.825207601 0.007606474
0.990782351
0.010768961 0.853555938 1.139700191 0.262662967
0.73331212 -7.079396167 cg15803671 18 3457981 p
DLGAP1 TGIF1 NA 3'UTR open sea 3'UTR - open sea
NA 0.849639952 0.860408913 0.010768961
0.987483903
0.011018728 0.853184894 1.126078047 0.268301472
0.739255024 -7.094670408 cg09926649 22
19938096 q COMT COMT NA 5'UTR open
sea 5'UTR - open sea NA 0.849178083 0.860196812
0.011018728 0.987190456
0.016180116 0.416625488 1.123330213 0.26944933
0.739255024 -7.097730904 cg12588917 6
29692082 p GABBR1/MOG HLA-F NA Body island
Body - island NA 0.410741809 0.426921925
0.016180116 0.962100527
0.009063587 0.839023191 1.122791058 0.269674965
0.739255024 -7.098330596 cg27491398 6
29708311 p MOG LOC285830 NA Body open
sea Body - open sea NA 0.835727342 0.844790928
0.009063587 0.989271208
0.004819497 0.05330898 1.118841944 0.271331798
0.739255024 -7.102714983 cg26232187 15
88799300 q NTRK3 NTRK3 NA Body shore
Body - shore NA 0.051556435 0.056375932
0.004819497 0.914511444
-0.057948831 0.761831914 -1.118684747 0.271397899
0.739255024 -7.102889209 cg19636627 6
29649084 p GABBR1/MOG ZFP57 8915 IGR open
sea IGR - open sea NA 0.782904216 0.724955385
-0.057948831 1.079934341
0.003619148 0.069517823 1.118634157 0.271419175
0.739255024 -7.102945276 cg13641185 6
29521143 p GABBR1 UBD -2246 IGR island
IGR - island NA 0.068201769 0.071820917
0.003619148 0.949608719
0.011928373 0.220952457 1.117640926 0.271837123
0.739255024 -7.104045539 cg08179037 6
29705815 p MOG LOC285830 NA Body open
sea Body - open sea NA 0.216614867 0.22854324
0.011928373 0.947806931
0.0135268 0.781794831 1.116523818 0.272307747
0.739255024 -7.105281947 cg14140375 6
29692582 p GABBR1/MOG HLA-F NA Body shore
Body - shore NA 0.776875995 0.790402795
0.0135268 0.982886194
0.006522282 0.056597693 1.113578927 0.273551192
0.739255024 -7.108535841 cg15710245 11
27722620 p BDNF BDNF NA TSS200 island
TSS200 - island NA 0.054225954 0.060748236
0.006522282 0.892634216

0.014025708 0.323173242 1.11271145 0.273918248
0.739255024 -7.109492823 cg01138652 22
19973978 q COMT ARVCF NA Body shore
Body - shore NA 0.318072985 0.332098693
0.014025708 0.957766446
0.01013429 0.785666989 1.112195197 0.274136856
0.739255024 -7.110062013 cg23270757 6
29427699 p GABBR1 OR2H1 NA 5'UTR open
sea 5'UTR - open sea NA 0.781981793 0.792116082
0.01013429 0.987206056
0.00362134 0.068284925 1.111990054 0.274223759
0.739255024 -7.110288124 cg17514757 6
29720651 p MOG IFITM4P 2067 IGR island
IGR - island NA 0.066968075 0.070589414
0.00362134 0.948698554
0.002299655 0.04955644 1.111175441 0.274569041
0.739255024 -7.111185618 cg12312205 18 3594173
p DLGAP1 DLGAP1 NA Body open sea Body -
open sea NA 0.048720201 0.051019857 0.002299655
0.954926255
0.020112057 0.66943956 1.110638673 0.274796725
0.739255024 -7.111776664 cg06301399 6
29634495 p GABBR1/MOG MOG NA 3'UTR open
sea 3'UTR - open sea NA 0.662126085 0.682238142
0.020112057 0.970520474
-0.014429373 0.162934925 -1.109837924 0.275136635
0.739255024 -7.112657894 cg14531834 9
87655988 q NTRK2 NTRK2 371362 IGR open
sea IGR - open sea NA 0.16818197 0.153752597 -
0.014429373 1.093847995
0.010638817 0.842662704 1.109723832 0.27518509
0.739255024 -7.112783404 cg01715172 6
152128024 q ESR1 ESR1 NA TSS1500 shore
TSS1500 - shore NA 0.838794043 0.849432861
0.010638817 0.987475387
0.006058533 0.082670416 1.108289855 0.275794625
0.739255024 -7.114359881 cg26751972 6
29692000 p GABBR1/MOG HLA-F NA Body island
Body - island NA 0.080467313 0.086525846
0.006058533 0.929980078
-0.005361583 0.918426178 -1.108089992 0.275879656
0.739255024 -7.114579455 cg07169712 6
29571419 p GABBR1/MOG GABBR1 NA Body open
sea Body - open sea V$RREB1_01;V$TAXCREB_01 0.920375845
0.915014262 -0.005361583 1.005859562
0.00701172 0.163275561 1.107139665 0.276284227
0.739255024 -7.115623002 cg15617548 2
171785815 q GAD1 GORASP2 NA 5'UTR island
5'UTR - island NA 0.160725844 0.167737564
0.00701172 0.958198272
0.009860791 0.807008085 1.107016821 0.276336555
0.739255024 -7.115757837 cg25633045 17
28458728 q SLC6A4 CCDC55 NA Body open
sea Body - open sea NA 0.803422343 0.813283134
0.009860791 0.987875328
0.007548208 0.067311293 1.106309775 0.276637872
0.739255024 -7.11653362 cg20830965 17

28619340 q SLC6A4 BLMH NA TSS1500 shore
TSS1500 - shore NA 0.06456649 0.072114698
0.007548208 0.895330519
0.002547273 0.03054593 1.105544663 0.276964198
0.739255024 -7.117372597 cg19128271 22
19842843 q COMT GNB1L NA TSS1500 island
TSS1500 - island NA 0.029619649 0.032166922
0.002547273 0.920810794
0.002772843 0.047113012 1.10494387 0.277220634
0.739255024 -7.118031014 cg03539474 2
172751088 q SLC25A12 SLC25A12 NA TSS1500 shore
TSS1500 - shore NA 0.046104705 0.048877548
0.002772843 0.943269597
0.015756151 0.637077094 1.101467969 0.278707569
0.741716812 -7.121833777 cg07529654 18 3447016
p DLGAP1 TGIF1 NA 5'UTR shore 5'UTR -
shore NA 0.631347584 0.647103736 0.015756151
0.975651273
0.001598157 0.018606249 1.096008266 0.281054588
0.741716812 -7.127784423 cg14583606 9 4490315
p SLC1A1 SLC1A1 NA TSS200 island TSS200
- island NA 0.018025101 0.019623258 0.001598157
0.918558019
0.007229705 0.127699061 1.095822226 0.281134809
0.741716812 -7.127986708 cg16850687 18 3594398
p DLGAP1 DLGAP1 NA Body open sea Body -
open sea NA 0.125070078 0.132299783 0.007229705
0.945353614
0.012182482 0.753826951 1.095653377 0.281207632
0.741716812 -7.128170274 cg21157690 6
152126895 q ESR1 ESR1 NA 5'UTR shore
5'UTR - shore NA 0.749396958 0.761579439
0.012182482 0.984003663
0.006829288 0.204769036 1.094015212 0.28191485
0.741716812 -7.12994985 cg08816824 6
29716244 p MOG LOC285830 NA Body shore
Body - shore NA 0.202285658 0.209114947
0.006829288 0.967341938
0.0051197 0.129999648 1.093720112 0.282042383
0.741716812 -7.130270161 cg06260077 11
27721350 p BDNF BDNF NA Body shore
Body - shore NA 0.128137939 0.133257639
0.0051197 0.961580439
0.006845442 0.903965131 1.092073882 0.282754582
0.741716812 -7.132055556 cg05972518 6
29577348 p GABBR1/MOG GABBR1 NA Body open
sea Body - open sea NA 0.901475879 0.908321321
0.006845442 0.992463634
0.006144899 0.872435795 1.09101653 0.28321269 0.741716812
-7.133200974 cg26126367 17 28559497 q SLC6A4
SLC6A4 NA 5'UTR shelf 5'UTR - shelf NA
0.870201286 0.876346185 0.006144899
0.992988046
0.009009545 0.800634716 1.09050386 0.283434999
0.741716812 -7.133755973 cg23330212 11
27672697 p BDNF BDNFOS NA Body open

sea	Body - open sea	NA	0.797358518	0.806368063
0.009009545	0.988827007
0.005485609	0.904302174	1.08936197 0.2839306	0.741716812
-7.134991271	cg22166516 2	172641053	q
SLC25A12	SLC25A12	NA	3'UTR	open sea	3'UTR - open sea
NA	0.902307407	0.907793016	0.005485609
0.993957203
-0.006502068	0.879936845	-1.088953801	0.284107902
0.741716812	-7.135432537	cg14273545 9
87449508	q	NTRK2	NTRK2	NA	Body	open
sea	Body - open sea	NA	0.882301234	0.875799166
-0.006502068	1.007424154
0.005369522	0.801137839	1.086462054	0.285191974
0.7431288	-7.138122992	cg13473117 22	19867665	q
COMT	TXNRD2	NA	Body	open sea	Body - open sea
NA	0.799185286	0.804554808	0.005369522
0.993326095
-0.015152057	0.51600676 -1.085132955	0.285771414
0.743222994	-7.139555738	cg12315353 6
29573089	p	GABBR1/MOG GABBR1	NA	Body	open
sea	Body - open sea	NA	0.521516599	0.506364542
-0.015152057	1.029923219
0.008815733	0.826959989	1.08039505 0.287843743
0.746483483	-7.144649834	cg21632181 6
29700035	p	MOG	LOC285830	NA	Body	open
sea	Body - open sea	NA	0.823754267	0.832570001
0.008815733	0.98941142
0.006499039	0.126674984	1.079773404	0.288116432
0.746483483	-7.145316674	cg13612847 2
171672205	q	GAD1	GAD1	NA	TSS1500	shore
TSS1500 - shore	NA	0.124311697	0.130810736
0.006499039	0.950317235
0.008784647	0.883272655	1.073289737	0.290971399
0.750637072	-7.1522504 cg08190562 6	29528774	p
GABBR1/MOG UBD	NA	TSS1500	open sea	TSS1500 - open
sea	NA	0.880078237	0.888862885	0.008784647
0.990116982
-0.007136511	0.882804427	-1.072649712	0.291254299
0.750637072	-7.152932742	cg13206902 6
29523786	p	GABBR1	UBD	NA	Body	shore
Body - shore	NA	0.885399521	0.878263011
-0.007136511	1.008125709
-0.005470825	0.895675445	-1.072397788	0.291365706
0.750637072	-7.153201219	cg03741824 21
34350407	q	OLIG2	OLIG2	-47809	IGR	shore
IGR - shore	NA	0.897664835	0.892194011
-0.005470825	1.006131877
-0.002173198	0.046802915	-1.070041389	0.292409213
0.751909404	-7.155709596	cg16017358 2
172779282	q	SLC25A12	HAT1	NA	Body	island
Body - island	NA	0.047593169	0.045419971
-0.002173198	1.04784675
-0.002932375	0.057027587	-1.068226733	0.293214602
0.752565809	-7.157637783	cg03541635 22
20004063	q	COMT	ARVCF	NA	5'UTR	island
5'UTR - island	NA	0.058093905	0.055161531
-0.002932375	1.053159765

0.005936418 0.881900975 1.066228351 0.294103335
0.753433262 -7.15975766 cg21652192 6
29571483 p GABBR1/MOG GABBR1 NA Body open
sea Body - open sea NA 0.879742277 0.885678695
0.005936418 0.993297323
0.008933455 0.834046284 1.0645749 0.294840094
0.753908877 -7.161508834 cg25798600 6
29454954 p GABBR1 MAS1L NA 1stExon open
sea 1stExon - open sea NA 0.830797755 0.839731209
0.008933455 0.989361532
0.002476871 0.042171765 1.059801542 0.296974306
0.756759762 -7.16655008 cg13604811 6
29720802 p MOG IFITM4P 2218 IGR island
IGR - island NA 0.041271084 0.043747956
0.002476871 0.943383138
0.010015817 0.156531828 1.058779192 0.29743281
0.756759762 -7.167627056 cg01089319 2
171676809 q GAD1 GAD1 NA Body island
Body - island NA 0.152889713 0.162905529
0.010015817 0.938517642
-0.006101844 0.13305959 -1.057758625 0.297891008
0.756759762 -7.168701186 cg22377998 22
20004881 q COMT ARVCF NA TSS1500 shore
TSS1500 - shore NA 0.135278442 0.129176598
-0.006101844 1.047236451
-0.042390379 0.707662282 -1.057142707 0.298167772
0.756759762 -7.169348961 cg15708526 6
29648271 p GABBR1/MOG ZFP57 8102 IGR open
sea IGR - open sea NA 0.723076965 0.680686586
-0.042390379 1.062275914
-0.005122574 0.904717545 -1.052956818 0.300053467
0.75816405 -7.173742019 cg26219152 6 152443475 q
ESR1 SYNE1 NA 3'UTR open sea 3'UTR - open sea
NA 0.906580299 0.901457725 -0.005122574
1.005682545
0.005872463 0.903044109 1.050801832 0.3010275
0.75816405 -7.175997308 cg00145961 15 88576296 q
NTRK3 NTRK3 NA Body open sea Body - open sea
NA 0.900908668 0.90678113 0.005872463
0.993523837
0.00552187 0.032650737 1.050769792 0.301041999
0.75816405 -7.176030807 cg27237300 21 34442292 q
OLIG2 OLIG1 NA TSS200 island TSS200 - island
NA 0.030642784 0.036164654 0.00552187
0.84731307
0.0071678 0.86844524 1.050589523 0.301123581 0.75816405 -
7.176219266 cg03215160 6 29424926 p GABBR1 OR2H1
NA TSS1500 open sea TSS1500 - open sea NA
0.865838767 0.873006568 0.0071678 0.991789522
-0.002143511 0.043953093 -1.049775565 0.301492137
0.75816405 -7.177069824 cg10694442 22 20004377 q
COMT ARVCF NA TSS200 island TSS200 - island
V$SP1_01 0.044732551 0.042589041 -0.002143511
1.050330084
0.002683775 0.020968095 1.047123444 0.302695184
0.759482695 -7.179836927 cg23248007 6
29716601 p MOG LOC285830 NA Body island

Body - island NA 0.019992177 0.022675952
0.002683775 0.881646645
0.014825855 0.807766824 1.046044464 0.303185581
0.759482695 -7.180960812 cg13150094 6
29714824 p MOG LOC285830 NA Body shore
Body - shore NA 0.802375604 0.817201459
0.014825855 0.981857772
0.006532356 0.887743717 1.044953379 0.303682043
0.759482695 -7.182096204 cg07463294 12
72243682 q TPH2 TBC1D15 NA Body open
sea Body - open sea NA 0.885368315 0.891900671
0.006532356 0.992675915
0.004697601 0.027486922 1.04284444 0.304643245
0.760496276 -7.184287642 cg07016276 6
29692009 p GABBR1/MOG HLA-F NA Body island
Body - island NA 0.025778703 0.030476304
0.004697601 0.845860541
-0.004595752 0.083322072 -1.040776613 0.305587759
0.761464581 -7.186432337 cg17587327 2
171670878 q GAD1 GAD1 -2322 IGR shore
IGR - shore NA 0.084993254 0.080397502
-0.004595752 1.057162871
-0.004172103 0.922129942 -1.039524184 0.306160814
0.761505442 -7.187729384 cg11912202 21
34350875 q OLIG2 OLIG2 -47341 IGR island
IGR - island NA 0.923647071 0.919474967
-0.004172103 1.004537485
0.00645577 0.888933901 1.037703046 0.306995413
0.761621289 -7.189612794 cg16924391 6
29719411 p MOG IFITM4P NA TSS1500 shore
TSS1500 - shore NA 0.886586348 0.893042118
0.00645577 0.992771035
0.00693929 0.841236655 1.03699381 0.307320871 0.761621289
-7.190345445 cg25562031 6 29624808 p
GABBR1/MOG MOG NA 1stExon open sea 1stExon - open
sea V$HTF_01;V$CREB_02;V$XBP1_01;V$ATF_01;V$ATF6_01 0.838713277
0.845652567 0.00693929 0.99179416
0.002808186 0.059680484 1.032809983 0.309245629
0.762716223 -7.194657851 cg15306595 2
171673272 q GAD1 GAD1 NA 5'UTR island
5'UTR - island NA 0.058659325 0.061467511
0.002808186 0.954314304
0.00200926 0.014730095 1.032542273 0.309369072
0.762716223 -7.194933231 cg26770917 21
34444339 q OLIG2 OLIG1 NA 1stExon island
1stExon - island V$CART1_01 0.013999455 0.016008715
0.00200926 0.874489614
0.006891083 0.860717516 1.032398652 0.30943531
0.762716223 -7.19508094 cg18423469 18 3726858
p DLGAP1 DLGAP1 NA Body shelf Body -
shelf NA 0.858211667 0.865102751 0.006891083
0.992034375
0.005851194 0.092954622 1.030441174 0.310339082
0.7635681 -7.197092216 cg13013381 6 29600103 p
GABBR1/MOG GABBR1 NA Body shore Body - shore
V$TAXCREB_02 0.090826915 0.09667811 0.005851194
0.939477561

0.006952307 0.8675601 1.026908985 0.311974509
0.764541618 -7.200712427 cg03906120 6
29583573 p GABBR1/MOG GABBR1 NA Body open
sea Body - open sea NA 0.865031988 0.871984295
0.006952307 0.992027027
0.001882702 0.030434288 1.026136531 0.312332951
0.764541618 -7.201502578 cg25990314 22
19841927 q COMT GNB1L NA 5'UTR island
5'UTR - island NA 0.029749669 0.031632371
0.001882702 0.940481793
-0.011926483 0.768560781 -1.025967601 0.312411378
0.764541618 -7.201675303 cg16403860 21
34394412 q OLIG2 OLIG2 -3804 IGR shore
IGR - shore NA 0.772897684 0.760971201
-0.011926483 1.015672713
0.003151664 0.065707374 1.021723014 0.314386401
0.765828487 -7.20600651 cg08856772 2
172751016 q SLC25A12 SLC25A12 NA TSS1500 island
TSS1500 - island NA 0.064561314 0.067712978
0.003151664 0.95345554
-0.004661493 0.927740722 -1.021330895 0.314569288
0.765828487 -7.206405781 cg01350824 6
29550425 p GABBR1/MOG SNORD32B 396 IGR open
sea IGR - open sea NA 0.92943581 0.924774317 -
0.004661493 1.005040682
0.008046577 0.083063125 1.020338012 0.315032702
0.765828487 -7.20741613 cg05733135 11
27740876 p BDNF BDNF NA Body island
Body - island NA 0.080137097 0.088183675
0.008046577 0.908752068
-0.00424597 0.048292003 -1.019445644 0.315449603
0.765828487 -7.20832341 cg06106763 21
34444104 q OLIG2 OLIG1 NA 1stExon island
1stExon - island NA 0.049835992 0.045590022
-0.00424597 1.093133756
-0.011158821 0.115460641 -1.018008519 0.316121803
0.765828487 -7.209782984 cg25729445 6
29595347 p GABBR1/MOG GABBR1 NA Body island
Body - island NA 0.119518394 0.108359572
-0.011158821 1.102979569
0.008380889 0.632279091 1.017097936 0.316548227
0.765828487 -7.210706789 cg09072576 22
19838287 q COMT GNB1L NA 5'UTR shelf
5'UTR - shelf NA 0.629231495 0.637612384
0.008380889 0.986855825
0.006141152 0.787915485 1.015572615 0.317263417
0.765828487 -7.212252522 cg13224710 18 3411743
p DLGAP1 TGIF1 NA TSS1500 open sea TSS1500
- open sea NA 0.785682338 0.791823491 0.006141152
0.99224429
0.003866652 0.071269257 1.015248021 0.317415755
0.765828487 -7.212581178 cg03448612 2
171672694 q GAD1 GAD1 NA TSS1500 island
TSS1500 - island NA 0.069863202 0.073729854
0.003866652 0.947556495
0.010184579 0.406389563 1.013901402 0.318048283
0.766003612 -7.213943598 cg12061917 6

29601556 p GABBR1/MOG GABBR1 NA TSS1500 shore
TSS1500 - shore NA 0.40268608 0.412870659
0.010184579 0.975332277
-0.002146552 0.033298752 -1.011669429 0.319098579
0.766045714 -7.21619802 cg19783435 22
19974682 q COMT ARVCF NA Body island
Body - island NA 0.034079317 0.031932765
-0.002146552 1.067220988
0.018583343 0.666051022 1.011484484 0.319185714
0.766045714 -7.216384615 cg20198768 6
29635579 p GABBR1/MOG MOG NA 3'UTR open
sea 3'UTR - open sea NA 0.659293442 0.677876785
0.018583343 0.972585957
0.002264477 0.032182036 1.007678998 0.320982262
0.768342963 -7.220216951 cg13344806 18 3450921
p DLGAP1 TGIF1 NA 5'UTR island 5'UTR -
island NA 0.03135859 0.033623067 0.002264477
0.932651087
-0.003886941 0.055886227 -1.007078867 0.32126621
0.768342963 -7.220820077 cg11116776 6
29691329 p GABBR1/MOG HLA-F NA Body island
Body - island NA 0.057299661 0.053412719
-0.003886941 1.072771843
0.002540209 0.06261371 0.999949933 0.324652346
0.773087911 -7.227958746 cg21930443 17
28443747 q SLC6A4 CCDC55 NA TSS200 island
TSS200 - island NA 0.061689998 0.064230207
0.002540209 0.96045149
0.00605666 0.135201951 0.99877966 0.325210522 0.773087911
-7.229126059 cg01583131 11 27744675 p BDNF
BDNF NA TSS1500 shore TSS1500 - shore NA
0.132999529 0.13905619 0.00605666 0.956444506
-0.002727055 0.025981688 -0.998353991 0.325413712
0.773087911 -7.229550332 cg25381667 11
27743651 p BDNF BDNF NA TSS200 island
TSS200 - island NA 0.026973345 0.02424629 -
0.002727055 1.112473084
0.009249969 0.455419079 0.99776783 0.325693653
0.773087911 -7.230134292 cg04517749 6
29570507 p GABBR1/MOG GABBR1 NA 3'UTR open
sea 3'UTR - open sea V$OCT_C 0.452055454 0.461305423
0.009249969 0.979948276
-0.018857763 0.587742637 -0.994447429 0.327282516
0.773087911 -7.233436136 cg23732781 6
29595016 p GABBR1/MOG GABBR1 NA Body shore
Body - shore NA 0.594600006 0.575742242
-0.018857763 1.032753831
-0.032243342 0.809983356 -0.993929176 0.327530983
0.773087911 -7.233950559 cg25699073 6
29648381 p GABBR1/MOG ZFP57 8212 IGR open
sea IGR - open sea NA 0.821708208 0.789464866
-0.032243342 1.040842023
-0.005188467 0.892396863 -0.992529342 0.328202746
0.773087911 -7.235338782 cg09842161 6
29598321 p GABBR1/MOG GABBR1 NA Body shore
Body - shore V$BRN2_01 0.894283579 0.889095112
-0.005188467 1.005835671

0.00458149 0.490681793 0.991714988 0.328593974
0.773087911 -7.236145534 cg07839627 6
29599836 p GABBR1/MOG GABBR1 NA Body shore
Body - shore NA 0.489015797 0.493597287
0.00458149 0.990718162
0.003541448 0.058017231 0.991336887 0.328775727
0.773087911 -7.236519894 cg11854392 22
19879320 q COMT TXNRD2 NA Body open
sea Body - open sea NA 0.056729431 0.06027088
0.003541448 0.941241127
0.002301678 0.054949887 0.991075458 0.328901436
0.773087911 -7.236778657 cg04378940 6
29617766 p GABBR1/MOG MOG -6992 IGR island
IGR - island NA 0.054112913 0.056414591
0.002301678 0.959200661
-0.002526707 0.035636403 -0.986980942 0.330874547
0.776391733 -7.240823037 cg05200610 12
72233572 q TPH2 TBC1D15 NA 1stExon island
1stExon - island V$AP2_Q6;V$FAC1_01 0.036555206
0.034028499 -0.002526707 1.074252673
0.003417716 0.078934426 0.984844008 0.331907492
0.777481934 -7.24292754 cg24611631 9 4490288
p SLC1A1 SLC1A1 NA TSS200 island TSS200
- island NA 0.07769162 0.081109336 0.003417716
0.957862853
0.045559255 0.313957697 0.982288975 0.333145395
0.778113812 -7.245438152 cg00274965 21
34405681 q OLIG2 OLIG2 7465 IGR island
IGR - island NA 0.297390695 0.34294995
0.045559255 0.867154799
0.008167099 0.862880854 0.981192297 0.333677687
0.778113812 -7.246513878 cg13680362 6
29426320 p GABBR1 OR2H1 NA 1stExon open
sea 1stExon - open sea NA 0.859911 0.868078098
0.008167099 0.990591747
0.001981434 0.041441523 0.97965932 0.334422705
0.778113812 -7.248015668 cg03867475 21
34444382 q OLIG2 OLIG1 NA 1stExon island
1stExon - island NA 0.040721002 0.042702436
0.001981434 0.953599041
0.003702649 0.226711776 0.979455268 0.334521958
0.778113812 -7.248215402 cg13965062 9
87284706 q NTRK2 NTRK2 NA 5'UTR island
5'UTR - island NA 0.225365358 0.229068007
0.003702649 0.983836027
0.007914109 0.116050885 0.978429451 0.335021225
0.778113812 -7.249218916 cg00858840 2
171573839 q GAD1 SP5 NA Body island
Body - island NA 0.113173028 0.121087137
0.007914109 0.934641208
0.006855422 0.911243283 0.97704525 0.335695714
0.778358875 -7.25057145 cg13499155 12
72376553 q TPH2 TPH2 NA Body open
sea Body - open sea NA 0.908750402 0.915605825
0.006855422 0.992512692
0.005232744 0.097603689 0.97145978 0.338426669
0.779303779 -7.256010782 cg11423684 6

29691993 p GABBR1/MOG HLA-F NA Body island
Body - island NA 0.095700873 0.100933617
0.005232744 0.948156579
0.011037351 0.635954875 0.971187274 0.338560289
0.779303779 -7.256275405 cg00094412 6
29592854 p GABBR1/MOG GABBR1 NA Body shelf
Body - shelf NA 0.631941293 0.642978643
0.011037351 0.982834033
0.002368272 0.047281046 0.970934955 0.338684042
0.779303779 -7.256520363 cg13770529 17
28443770 q SLC6A4 CCDC55 NA TSS200 island
TSS200 - island NA 0.046419856 0.048788128
0.002368272 0.951458027
0.018275271 0.636978386 0.969574976 0.339351582
0.779303779 -7.25783963 cg06486622 6
29718119 p MOG LOC285830 NA TSS1500 shore
TSS1500 - shore NA 0.630332833 0.648608104
0.018275271 0.971823863
0.005103635 0.069372833 0.968858989 0.339703377
0.779303779 -7.258533482 cg01176826 6
29720527 p MOG IFITM4P 1943 IGR island
IGR - island NA 0.067516966 0.072620601
0.005103635 0.929721939
0.002556003 0.029615974 0.967502814 0.340370392
0.779303779 -7.259846403 cg15568960 15
88790380 q NTRK3 NTRK3 NA Body open
sea Body - open sea NA 0.028686518 0.031242521
0.002556003 0.918188324
0.008450155 0.786241014 0.966616515 0.340806779
0.779303779 -7.260703495 cg11234013 11
27646797 p BDNF BDNFOS NA Body open
sea Body - open sea NA 0.78316823 0.791618385
0.008450155 0.989325469
0.005790091 0.107098288 0.966077018 0.341072596
0.779303779 -7.261224852 cg02892153 18 3593461
p DLGAP1 FLJ35776 NA TSS1500 open sea TSS1500
- open sea NA 0.1049928 0.110782892 0.005790091
0.947734782
0.005246009 0.765602144 0.965137204 0.341535984
0.779303779 -7.262132407 cg20642417 6
29577082 p GABBR1/MOG GABBR1 NA Body open
sea Body - open sea NA 0.763694505 0.768940513
0.005246009 0.993177615
0.0045011 0.875212149 0.96264552 0.342766582 0.779303779
-7.264534524 cg06309882 6 29443400 p GABBR1
MAS1L -11143 IGR open sea IGR - open sea NA
0.873575386 0.878076486 0.0045011 0.994873909
0.017571035 0.695473237 0.962559953 0.342808895
0.779303779 -7.264616912 cg20704602 6
29635371 p GABBR1/MOG MOG NA 3'UTR open
sea 3'UTR - open sea NA 0.68908377 0.706654805
0.017571035 0.97513491
0.012498532 0.436749806 0.962296414 0.342939236
0.779303779 -7.264870613 cg21481950 6
29601498 p GABBR1/MOG GABBR1 NA TSS1500 shore
TSS1500 - shore NA 0.432204885 0.444703417
0.012498532 0.97189468

0.005148151 0.900578916 0.96079491 0.343682482
0.779697571 -7.266314815 cg01057705 22
19892666 q COMT TXNRD2 NA Body shore
Body - shore NA 0.898706861 0.903855012
0.005148151 0.994304229
0.004405639 0.89554796 0.95536635 0.346378598 0.783268598
-7.271518417 cg14592798 9 87257941 q NTRK2
NTRK2 -25525 IGR open sea IGR - open sea NA
0.89394591 0.898351549 0.004405639 0.995095863
0.006649302 0.898523523 0.955319992 0.346401683
0.783268598 -7.271562733 cg03063857 6
29585617 p GABBR1/MOG GABBR1 NA Body open
sea Body - open sea NA 0.896105595 0.902754897
0.006649302 0.992634433
-0.002853773 0.037603202 -0.953238516 0.34743923
0.784318262 -7.273550466 cg14545305 2
171670978 q GAD1 GAD1 -2222 IGR shore
IGR - shore NA 0.038640938 0.035787165
-0.002853773 1.079742919
-0.037481198 0.642021376 -0.949454293 0.349330834
0.785624323 -7.277153751 cg10648573 6
29648348 p GABBR1/MOG ZFP57 8179 IGR open
sea IGR - open sea NA 0.655650902 0.618169705
-0.037481198 1.060632536
-0.002503421 0.086849028 -0.949133322 0.349491591
0.785624323 -7.277458751 cg04057037 6
29600203 p GABBR1/MOG GABBR1 NA 5'UTR island
5'UTR - island NA 0.087759363 0.085255942
-0.002503421 1.029363596
-0.006318437 0.058919224 -0.948636232 0.349740652
0.785624323 -7.277930914 cg01558660 9 4679986
p SLC1A1 CDC37L1 NA Body island Body -
island NA 0.061216837 0.0548984 -0.006318437
1.115093281
-0.006996416 0.826636986 -0.945294028 0.351418289
0.785792267 -7.281099443 cg24177217 6
29702053 p MOG LOC285830 NA Body open
sea Body - open sea NA 0.829181137 0.822184721
-0.006996416 1.008509543
0.006023185 0.137834063 0.944888487 0.351622214
0.785792267 -7.281483189 cg20627916 6
152128328 q ESR1 ESR1 NA TSS1500 shore
TSS1500 - shore V$OCT1_02 0.135643813 0.141666998
0.006023185 0.957483499
0.004872478 0.065301051 0.944153275 0.351992115
0.785792267 -7.282178493 cg27351358 11
27743258 p BDNF BDNF NA TSS1500 shore
TSS1500 - shore NA 0.06352924 0.068401718
0.004872478 0.928766731
0.002863589 0.048825683 0.941991837 0.35308107
0.785792267 -7.284219636 cg10491628 6
29521220 p GABBR1 UBD -2169 IGR island
IGR - island NA 0.047784378 0.050647967
0.002863589 0.943460929
0.002994275 0.058203602 0.941224313 0.353468293
0.785792267 -7.284943378 cg24102938 17
28444044 q SLC6A4 MIR423 NA TSS200 shore

TSS200 - shore NA 0.057114775 0.06010905
0.002994275 0.950185954
-0.002112255 0.018783229 -0.938218964 0.35498722
0.785792267 -7.28777191 cg17298239 18 3499253
p DLGAP1 DLGAP1 NA Body island Body -
island NA 0.019551321 0.017439067 -0.002112255
1.121121961
0.002033766 0.043975605 0.938128056 0.355033233
0.785792267 -7.287857336 cg00346247 6
29716851 p MOG LOC285830 NA TSS200 island
TSS200 - island NA 0.043236054 0.04526982
0.002033766 0.955074573
0.00433939 0.931715026 0.93685902 0.355675961 0.785792267
-7.289049026 cg23908638 6 29579475 p
GABBR1/MOG GABBR1 NA Body open sea Body - open sea
NA 0.930137066 0.934476456 0.00433939
0.995356341
-0.018473509 0.559155103 -0.933591715 0.357334284
0.785792267 -7.292110157 cg06512249 6
29599390 p GABBR1/MOG GABBR1 NA Body shore
Body - shore NA 0.565872743 0.547399234
-0.018473509 1.03374778
0.008671741 0.75009204 0.933450517 0.357406064
0.785792267 -7.292242217 cg00667298 6
29576329 p GABBR1/MOG GABBR1 NA Body open
sea Body - open sea NA 0.74693868 0.755610421
0.008671741 0.988523529
0.002397358 0.040671029 0.932720987 0.35777708
0.785792267 -7.292924229 cg02954262 18 3451750
p DLGAP1 TGIF1 NA 5'UTR island 5'UTR -
island NA 0.039799262 0.04219662 0.002397358
0.943186018
-0.004608492 0.934511095 -0.932706397 0.357784503
0.785792267 -7.292937863 cg12550837 6
29697949 p MOG LOC285830 NA Body open
sea Body - open sea NA 0.93618691 0.931578419 -
0.004608492 1.004946971
0.005424252 0.122000558 0.932072963 0.358106858
0.785792267 -7.29352962 cg03696345 21
34398114 q OLIG2 OLIG2 NA TSS200 island
TSS200 - island NA 0.120028103 0.125452355
0.005424252 0.956762454
0.006191807 0.063780997 0.931474616 0.358411534
0.785792267 -7.294088249 cg22762215 6
29521272 p GABBR1 UBD -2117 IGR island
IGR - island NA 0.061529431 0.067721238
0.006191807 0.9085692
-0.002478951 0.025750707 -0.930302582 0.359008822
0.785792267 -7.295181496 cg26375461 6
29716541 p MOG LOC285830 NA Body island
Body - island NA 0.026652144 0.024173193
-0.002478951 1.102549589
0.006224831 0.165027032 0.928648107 0.359853083
0.785792267 -7.296722531 cg09926027 9
87285693 q NTRK2 NTRK2 NA Body island
Body - island NA 0.162763457 0.168988288
0.006224831 0.963164128

0.005289042 0.896817496 0.927427852 0.360476601
0.785792267 -7.297857451 cg14809932 6
29525723 p GABBR1/MOG UBD NA Body shelf
Body - shelf NA 0.894894208 0.900183251
0.005289042 0.994124482
-0.004467687 0.089639582 -0.926960802 0.360715439
0.785792267 -7.298291465 cg25804470 6
29600193 p GABBR1/MOG GABBR1 NA 5'UTR island
5'UTR - island NA 0.091264196 0.086796509
-0.004467687 1.051473119
-0.004684169 0.092771172 -0.926933902 0.360729198
0.785792267 -7.298316456 cg12801329 2
171670795 q GAD1 GAD1 -2405 IGR shore
IGR - shore NA 0.094474507 0.089790337
-0.004684169 1.052167863
-0.011912298 0.603865348 -0.924316814 0.362069468
0.78624357 -7.300744521 cg01765653 6 29599160 p
GABBR1/MOG GABBR1 NA Body shore Body - shore
NA 0.608197092 0.596284794 -0.011912298
1.019977531
-0.004328684 0.89099644 -0.923235939 0.36262396 0.78624357 -
7.301745427 cg09149541 22 19898335 q COMT TXNRD2
NA Body shelf Body - shelf NA
0.892570507 0.888241823 -0.004328684
1.004873317
0.004828587 0.876553797 0.923164566 0.362660594
0.78624357 -7.30181148 cg19530293 12 72426134 q
TPH2 TPH2 NA 3'UTR open sea 3'UTR - open sea
NA 0.874797947 0.879626535 0.004828587
0.994510639
0.0059813 0.858620313 0.918286168 0.365170308
0.790431933 -7.306314781 cg19930203 22
19956281 q COMT COMT NA 3'UTR open
sea 3'UTR - open sea NA 0.856445294 0.862426594
0.0059813 0.993064569
0.001866217 0.032540472 0.914473828 0.367139465
0.793438843 -7.309818216 cg16639998 2
171626995 q GAD1 GAD1 -46205 IGR shore
IGR - shore NA 0.031861848 0.033728064
0.001866217 0.944668748
-0.004737036 0.176437315 -0.9124955 0.368164037
0.794138609 -7.311630787 cg15462887 11
27744049 p BDNF BDNF NA TSS1500 island
TSS1500 - island NA 0.178159873 0.173422837
-0.004737036 1.027314949
0.006697249 0.894890031 0.911607991 0.368624281
0.794138609 -7.312442722 cg10819807 6
29461549 p GABBR1 MAS1L 7006 IGR open
sea IGR - open sea NA 0.892454667 0.899151917
0.006697249 0.992551592
0.002887896 0.031460703 0.90855676 0.370209444
0.794263203 -7.315228404 cg11108676 15
88801004 q NTRK3 NTRK3 NA TSS1500 island
TSS1500 - island NA 0.030410559 0.033298454
0.002887896 0.9132724
0.004381853 0.020162996 0.907350535 0.370837317
0.794263203 -7.316327202 cg03205258 22

19929274 q COMT TXNRD2 NA 1stExon island
1stExon - island V$ELK1_02 0.018569594 0.022951448
0.004381853 0.809081588
0.003276584 0.049789386 0.906183677 0.371445355
0.794263203 -7.317388817 cg01009697 9
87283470 q NTRK2 NTRK2 NA TSS1500 island
TSS1500 - island NA 0.0485979 0.051874485
0.003276584 0.936836289
-0.029959965 0.73808476 -0.901424792 0.373931863
0.794263203 -7.321705027 cg04071440 6
29648275 p GABBR1/MOG ZFP57 8106 IGR open
sea IGR - open sea NA 0.748979292 0.719019327
-0.029959965 1.041667816
0.002427098 0.028147146 0.901396697 0.373946574
0.794263203 -7.321730444 cg17748329 6
29717058 p MOG LOC285830 NA TSS1500 island
TSS1500 - island NA 0.027264565 0.029691663
0.002427098 0.918256583
-0.001731483 0.018375297 -0.901113037 0.374095129
0.794263203 -7.321987027 cg08729407 17
28431896 q SLC6A4 EFCAB5 NA Body open
sea Body - open sea NA 0.019004927 0.017273445
-0.001731483 1.10023953
0.002814818 0.089882038 0.900993571 0.374157706
0.794263203 -7.322095067 cg04672351 11
27722889 p BDNF BDNF NA TSS1500 shore
TSS1500 - shore NA 0.088858467 0.091673286
0.002814818 0.969295101
0.001852905 0.092957868 0.899026985 0.375188784
0.794263203 -7.323871594 cg25456960 6
29717019 p MOG LOC285830 NA TSS200 island
TSS200 - island NA 0.092284085 0.094136989
0.001852905 0.98031694
-0.049503336 0.761364617 -0.896472864 0.376530646
0.794263203 -7.326173359 cg16885113 6
29648507 p GABBR1/MOG ZFP57 8338 IGR open
sea IGR - open sea NA 0.779365831 0.729862494
-0.049503336 1.067825566
0.00815394 0.82855298 0.89626532 0.376639819 0.794263203 -
7.326360123 cg01465527 6 29548535 p GABBR1/MOG
SNORD32B NA TSS1500 open sea TSS1500 - open sea NA
0.825587911 0.833741851 0.00815394 0.990220066
-0.002809487 0.068636038 -0.895498744 0.377043236
0.794263203 -7.32704959 cg01388957 9 4679439
p SLC1A1 CDC37L1 NA TSS200 island TSS200
- island NA 0.069657669 0.066848183 -0.002809487
1.042027859
0.003353164 0.065798557 0.895112008 0.377246864
0.794263203 -7.327397212 cg26069562 6
29691981 p GABBR1/MOG HLA-F NA Body island
Body - island NA 0.064579224 0.067932388
0.003353164 0.950639686
0.002568774 0.1156092 0.893621749 0.378032194
0.794263203 -7.328735409 cg14629287 6
29716767 p MOG LOC285830 NA Body island
Body - island NA 0.1146751 0.117243874
0.002568774 0.978090335

-0.00329768 0.068110569 -0.892634894 0.378552822
0.794263203 -7.329620399 cg03837627 17
28443756 q SLC6A4 CCDC55 NA TSS200 island
TSS200 - island NA 0.069309726 0.066012046
-0.00329768 1.049955731
0.013986148 0.865157936 0.890578545 0.379639158
0.794263203 -7.331461496 cg02979010 12
72319077 q TPH2 TBC1D15 NA 3'UTR open
sea 3'UTR - open sea NA 0.860072064 0.874058212
0.013986148 0.983998608
0.003995626 0.132306342 0.889885136 0.380005927
0.794263203 -7.332081409 cg14738290 6
29690998 p GABBR1/MOG HLA-F NA TSS200 shore
TSS200 - shore NA 0.130853387 0.134849014
0.003995626 0.970369624
0.008289449 0.825534773 0.889833643 0.380033172
0.794263203 -7.332127426 cg01335087 22
19950166 q COMT COMT NA Body open
sea Body - open sea NA 0.822520428 0.830809877
0.008289449 0.990022448
-0.003355544 0.095139942 -0.889468198 0.380226569
0.794263203 -7.332453933 cg04408897 6
29596901 p GABBR1/MOG GABBR1 NA TSS1500 shore
TSS1500 - shore NA 0.09636014 0.093004596 -
0.003355544 1.036079335
0.004860209 0.045800574 0.889298465 0.380316415
0.794263203 -7.332605538 cg15555970 18 3452317
p DLGAP1 TGIF1 NA 5'UTR island 5'UTR -
island NA 0.044033225 0.048893434 0.004860209
0.900595876
-0.043557951 0.794095748 -0.888602099 0.380685171
0.794263203 -7.333227241 cg22494932 6
29648379 p GABBR1/MOG ZFP57 8210 IGR open
sea IGR - open sea NA 0.809935003 0.766377052
-0.043557951 1.056836189
0.002211733 0.074392853 0.888244259 0.380874752
0.794263203 -7.333546534 cg09749751 6
29600125 p GABBR1/MOG GABBR1 NA Body shore
Body - shore NA 0.073588587 0.07580032
0.002211733 0.970821588
0.005590008 0.897835763 0.883717463 0.383278248
0.798058817 -7.337575105 cg21288364 6
29524117 p GABBR1 UBD NA Body shelf
Body - shelf NA 0.895803033 0.901393041
0.005590008 0.993798479
-0.00198739 0.043537833 -0.881823853 0.384286534
0.798713036 -7.339254481 cg17597787 2
171785529 q GAD1 GORASP2 NA TSS200 island
TSS200 - island NA 0.04426052 0.04227313 -0.00198739
1.047013079
0.003004872 0.077476717 0.880935471 0.384760154
0.798713036 -7.340041172 cg04711050 9 4490757
p SLC1A1 SLC1A1 NA 1stExon island 1stExon
- island NA 0.076384036 0.079388908 0.003004872
0.962149977
-0.008084343 0.81750877 -0.878821871 0.38588847 0.799438179
-7.341909793 cg11219691 22 19967280 q COMT

ARVCF NA Body island Body - island NA
0.820448531 0.812364188 -0.008084343
1.009951624
0.008510173 0.713246225 0.878092653 0.386278243
0.799438179 -7.342553496 cg23325570 6
29711975 p MOG LOC285830 NA Body open
sea Body - open sea NA 0.710151617 0.71866179
0.008510173 0.988158306
-0.047728268 0.688362833 -0.876140468 0.387322938
0.800389395 -7.344274242 cg24100841 6
29649024 p GABBR1/MOG ZFP57 8855 IGR open
sea IGR - open sea NA 0.705718567 0.657990299
-0.047728268 1.072536431
0.00580108 0.067707562 0.874488438 0.388208415
0.801009218 -7.345727562 cg05724110 21
34398532 q OLIG2 OLIG2 NA 5'UTR island
5'UTR - island NA 0.065598079 0.071399158
0.00580108 0.918751437
0.003322097 0.082642691 0.872205039 0.389434423
0.801484919 -7.347731999 cg05661333 6
29600200 p GABBR1/MOG GABBR1 NA 5'UTR island
5'UTR - island NA 0.081434656 0.084756753
0.003322097 0.960804339
0.002036001 0.049570353 0.87187706 0.389610725
0.801484919 -7.348019498 cg24279419 6
29617791 p GABBR1/MOG MOG -6967 IGR island
IGR - island NA 0.048829989 0.05086599
0.002036001 0.959973236
0.005886315 0.715715327 0.869470104 0.390906112
0.802942284 -7.350126224 cg21224669 6
29639803 p GABBR1/MOG MOG NA 3'UTR open
sea 3'UTR - open sea NA 0.713574849 0.719461164
0.005886315 0.99181844
0.012163328 0.285751665 0.867050771 0.392210914
0.804414588 -7.352238177 cg20215212 17
28618041 q SLC6A4 BLMH NA Body shore
Body - shore NA 0.281328636 0.293491965
0.012163328 0.958556518
0.003945701 0.083524953 0.864521967 0.393577707
0.805511319 -7.354439685 cg11813455 6
152128515 q ESR1 ESR1 NA TSS1500 shore
TSS1500 - shore NA 0.082090152 0.086035854
0.003945701 0.954138864
0.001998722 0.048257089 0.863406222 0.394181714
0.805511319 -7.355409068 cg25777153 22
20008297 q COMT C22orf25 NA TSS1500 shore
TSS1500 - shore NA 0.047530281 0.049529004
0.001998722 0.959645403
0.001707199 0.051739747 0.862796337 0.394512123
0.805511319 -7.355938445 cg19862860 22
20008420 q COMT C22orf25 NA TSS1500 island
TSS1500 - island NA 0.051118947 0.052826146
0.001707199 0.967682689
0.003140532 0.123997936 0.859355236 0.39637964
0.807390887 -7.358918598 cg03984780 11
27722617 p BDNF BDNF NA TSS200 island

TSS200 - island NA 0.122855925 0.125996457
0.003140532 0.975074442
-0.006964624 0.827735341 -0.858925844 0.396613067
0.807390887 -7.359289673 cg02475474 6
29635158 p GABBR1/MOG MOG NA 3'UTR open
sea 3'UTR - open sea NA 0.830267932 0.823303307
-0.006964624 1.008459367
0.003305979 0.029785888 0.856234707 0.398078
0.809168951 -7.361611277 cg11032634 22
19929254 q COMT TXNRD2 NA 1stExon island
1stExon - island NA 0.028583714 0.031889692
0.003305979 0.896330827
0.007022047 0.785712767 0.852393713 0.400174763
0.810791322 -7.364912779 cg00359010 6
29635692 p GABBR1/MOG MOG NA 3'UTR open
sea 3'UTR - open sea NA 0.783159295 0.790181343
0.007022047 0.991113372
0.004484112 0.886591881 0.851283027 0.400782369
0.810791322 -7.365864816 cg00806253 6
29571432 p GABBR1/MOG GABBR1 NA Body open
sea Body - open sea V$TAXCREB_01 0.884961294
0.889445406 0.004484112 0.99495853
0.00474398 0.890195803 0.850641378 0.401133651
0.810791322 -7.366414271 cg25654517 6
29627131 p GABBR1/MOG MOG NA Body open
sea Body - open sea NA 0.888470719 0.893214699
0.00474398 0.994688869
-0.00497682 0.049211411 -0.849671823 0.401664816
0.810791322 -7.367243765 cg26015683 6
29720519 p MOG IFITM4P 1935 IGR island
IGR - island NA 0.051021164 0.046044344
-0.00497682 1.108087543
0.033794868 0.505151691 0.849353034 0.401839559
0.810791322 -7.367516305 cg11935738 6
29520752 p GABBR1 UBD -2637 IGR shore
IGR - shore NA 0.492862648 0.526657516
0.033794868 0.935831414
0.005442075 0.901591252 0.847330409 0.402949366
0.810986164 -7.36924321 cg20496034 6
29574810 p GABBR1/MOG GABBR1 NA Body open
sea Body - open sea NA 0.899612315 0.90505439
0.005442075 0.993987019
0.004407945 0.165765191 0.845950452 0.403707645
0.810986164 -7.370419147 cg14293300 21
34399361 q OLIG2 OLIG2 NA Body island
Body - island NA 0.164162302 0.168570247
0.004407945 0.97385099
-0.05792842 0.715411902 -0.845254661 0.404090317
0.810986164 -7.371011373 cg11383134 6
29648590 p GABBR1/MOG ZFP57 8421 IGR open
sea IGR - open sea NA 0.736476782 0.678548361
-0.05792842 1.085371102
0.01008766 0.504923981 0.841780295 0.406004552
0.810986164 -7.373961616 cg12349676 21
34350934 q OLIG2 OLIG2 -47282 IGR island
IGR - island NA 0.501255741 0.511343401
0.01008766 0.98027224

-0.011071202 0.467494476 -0.841635076 0.406084685
0.810986164 -7.374084675 cg23122901 22
19880135 q COMT TXNRD2 NA Body open
sea Body - open sea NA 0.471520367 0.460449165
-0.011071202 1.024044352
0.003112856 0.067704534 0.839410914 0.407313228
0.810986164 -7.375966892 cg02100602 21
34396665 q OLIG2 OLIG2 -1551 IGR island
IGR - island NA 0.066572587 0.069685443
0.003112856 0.955329896
0.018739297 0.363428827 0.838585286 0.407769863
0.810986164 -7.376664372 cg12423733 6
29454623 p GABBR1 MAS1L NA 1stExon open
sea 1stExon - open sea NA 0.356614537 0.375353834
0.018739297 0.950075648
-0.00976674 0.416094421 -0.838321598 0.40791577
0.810986164 -7.376886994 cg23606396 6
29717917 p MOG LOC285830 NA TSS1500 shore
TSS1500 - shore NA 0.419645963 0.409879223
-0.00976674 1.023828336
0.006531147 0.869380459 0.838240123 0.40796086
0.810986164 -7.376955767 cg23306453 6
29599012 p GABBR1/MOG GABBR1 NA Body shore
Body - shore NA 0.867005496 0.873536644
0.006531147 0.992523327
0.003479181 0.890908351 0.837014218 0.408639665
0.810986164 -7.377989775 cg21864713 6
29555314 p GABBR1/MOG OR2H2 NA TSS1500 open
sea TSS1500 - open sea NA 0.889643194 0.893122375
0.003479181 0.996104474
0.008563942 0.794479603 0.835966455 0.409220388
0.810986164 -7.378872379 cg00140112 18 3879595
p DLGAP1 DLGAP1 NA 1stExon island 1stExon
- island NA 0.791365442 0.799929384 0.008563942
0.989294127
0.006487595 0.867951021 0.835515571 0.409470448
0.810986164 -7.379251863 cg17745803 6
29631321 p GABBR1/MOG MOG NA Body open
sea Body - open sea NA 0.865591895 0.87207949
0.006487595 0.992560776
-0.002590391 0.039762859 -0.834305148 0.410142219
0.810986164 -7.38026964 cg27357571 21
34398226 q OLIG2 OLIG2 NA TSS200 island
TSS200 - island NA 0.04070482 0.038114428 -
0.002590391 1.067963554
-0.014843845 0.553868949 -0.834136825 0.410235691
0.810986164 -7.380411062 cg21644740 6
29599248 p GABBR1/MOG GABBR1 NA Body shore
Body - shore NA 0.559266711 0.544422866
-0.014843845 1.027265286
0.005766219 0.843342025 0.832921574 0.410910926
0.811148842 -7.381431279 cg22310628 6
29692995 p GABBR1/MOG HLA-F NA Body shore
Body - shore NA 0.841245218 0.847011437
0.005766219 0.993192277
0.005216135 0.879057516 0.827308863 0.414038491
0.816145038 -7.386124708 cg24251942 15

88576320 q NTRK3 NTRK3 NA Body open
sea Body - open sea NA 0.877160739 0.882376875
0.005216135 0.99408854
-0.00706795 0.804888104 -0.82622061 0.414646599
0.816167695 -7.387031199 cg25836061 22
19939028 q COMT COMT NA 5'UTR open
sea 5'UTR - open sea NA 0.807458268 0.800390317
-0.00706795 1.00883063
0.01006946 0.641721446 0.823989404 0.415895107
0.816933496 -7.388886165 cg27639046 2
171608303 q GAD1 SP5 36446 IGR open
sea IGR - open sea NA 0.638059824 0.648129284
0.01006946 0.98446381
-0.004051495 0.069298849 -0.822307481 0.416837788
0.816933496 -7.390281291 cg07375883 18 3448693
p DLGAP1 TGIF1 NA 5'UTR island 5'UTR -
island NA 0.07077212 0.066720625 -0.004051495
1.060723277
-0.003272392 0.100220897 -0.822248763 0.416870722
0.816933496 -7.390329947 cg22593533 21
34397654 q OLIG2 OLIG2 NA TSS1500 island
TSS1500 - island NA 0.101410858 0.098138466
-0.003272392 1.033344642
0.003495244 0.100652333 0.820203613 0.418018813
0.816933496 -7.392022561 cg10580691 18 3451079
p DLGAP1 TGIF1 NA 5'UTR island 5'UTR -
island NA 0.099381335 0.102876579 0.003495244
0.966024881
-0.002869761 0.057045854 -0.820198785 0.418021526
0.816933496 -7.392026552 cg07813142 2
171573223 q GAD1 SP5 NA Body island
Body - island NA 0.058089403 0.055219642
-0.002869761 1.051969931
-0.003844564 0.039039645 -0.818324695 0.419075303
0.817824557 -7.393574032 cg21053831 18 4455512
p DLGAP1 DLGAP1-AS5 190910 IGR island IGR -
island NA 0.040437669 0.036593105 -0.003844564
1.105062525
0.009990326 0.772515006 0.813527847 0.421779947
0.818500795 -7.39751942 cg11361387 22
19949873 q COMT COMT NA 5'UTR open
sea 5'UTR - open sea NA 0.768882161 0.778872487
0.009990326 0.987173348
0.012789002 0.701288757 0.812762347 0.422212553
0.818500795 -7.398146979 cg19022254 6
29601705 p GABBR1/MOG GABBR1 NA TSS1500 shore
TSS1500 - shore NA 0.696638211 0.709427213
0.012789002 0.981972778
-0.00600664 0.83858627 -0.812670138 0.422264682
0.818500795 -7.398222534 cg25699759 15
88576311 q NTRK3 NTRK3 NA Body open
sea Body - open sea NA 0.840770502 0.834763862
-0.00600664 1.007195616
0.011278977 0.218173385 0.811571357 0.422886158
0.818500795 -7.399122229 cg18132851 6
152085641 q ESR1 ESR1 NA 5'UTR open

sea 5'UTR - open sea NA 0.214071939 0.225350916
0.011278977 0.949949274
0.002150596 0.028167759 0.8100918 0.423723888
0.818500795 -7.40033186 cg18388802 22
20004382 q COMT ARVCF NA TSS200 island
TSS200 - island V$SP1_01 0.027385724 0.02953632
0.002150596 0.927188086
-0.007164672 0.881054467 -0.808144312 0.424828108
0.818500795 -7.401920821 cg26987604 6
29696647 p MOG LOC285830 NA Body open
sea Body - open sea NA 0.883659802 0.87649513 -
0.007164672 1.008174229
-0.003217963 0.064694038 -0.80774376 0.425055438
0.818500795 -7.402247177 cg06212631 2
171785547 q GAD1 GORASP2 NA TSS200 island
TSS200 - island NA 0.065864207 0.062646243
-0.003217963 1.05136723
0.007492055 0.786110492 0.806561769 0.4257267
0.818500795 -7.403209316 cg02965092 12
72347052 q TPH2 TPH2 NA Body open
sea Body - open sea NA 0.783386108 0.790878164
0.007492055 0.990526915
-0.001968608 0.046090797 -0.805979463 0.426057634
0.818500795 -7.403682815 cg09523380 6
29720748 p MOG IFITM4P 2164 IGR island
IGR - island NA 0.046806654 0.044838046
-0.001968608 1.043904857
0.00483553 0.690146492 0.805576931 0.426286492
0.818500795 -7.404009939 cg14528040 6
29628293 p GABBR1/MOG MOG NA Body open
sea Body - open sea NA 0.688388117 0.693223647
0.00483553 0.993024574
0.001402929 0.016735738 0.805134985 0.426537844
0.818500795 -7.404368913 cg26332258 6
29720425 p MOG IFITM4P 1841 IGR island
IGR - island NA 0.016225582 0.017628511
0.001402929 0.920417045
0.006966647 0.853112477 0.805022809 0.426601657
0.818500795 -7.404459998 cg18977283 6
29579492 p GABBR1/MOG GABBR1 NA Body open
sea Body - open sea NA 0.850579151 0.857545798
0.006966647 0.991876064
-0.001538708 0.032206143 -0.802661205 0.42794645
0.818830984 -7.406374761 cg26491697 18 3451447
p DLGAP1 TGIF1 NA 5'UTR island 5'UTR -
island NA 0.032765673 0.031226965 -0.001538708
1.049274978
-0.011591036 0.635740585 -0.802618358 0.427970873
0.818830984 -7.406409451 cg03127104 6
29599250 p GABBR1/MOG GABBR1 NA Body shore
Body - shore NA 0.639955507 0.628364472
-0.011591036 1.018446356
-0.003729825 0.066451594 -0.80027555 0.429307557
0.819607407 -7.408303533 cg04049102 6
29716651 p MOG LOC285830 NA Body island
Body - island NA 0.067807895 0.064078069
-0.003729825 1.058207528

0.005770233 0.817731605 0.799292204 0.429869359
0.819607407 -7.40909695 cg12904135 2
171669275 q GAD1 GAD1 -3925 IGR shore
IGR - shore NA 0.815633338 0.821403572
0.005770233 0.992975154
0.00494766 0.896658011 0.798759193 0.430174063
0.819607407 -7.409526619 cg16101636 6
29711438 p MOG LOC285830 NA Body open
sea Body - open sea NA 0.894858862 0.899806522
0.00494766 0.994501418
0.010739016 0.541849971 0.794955728 0.432352181
0.822611661 -7.412584656 cg23892836 6
29692085 p GABBR1/MOG HLA-F NA Body island
Body - island NA 0.537944874 0.54868389
0.010739016 0.980427681
0.010227848 0.172080251 0.789021257 0.435763972
0.827951547 -7.417327995 cg14730102 21
34401634 q OLIG2 OLIG2 3418 IGR shore
IGR - shore NA 0.168361033 0.178588881
0.010227848 0.942729648
0.002908036 0.942045663 0.785918471 0.437554246
0.828724118 -7.419794387 cg03467156 6
29425910 p GABBR1 OR2H1 NA TSS1500 open
sea TSS1500 - open sea NA 0.940988196 0.943896232
0.002908036 0.996919115
-0.005961758 0.672966962 -0.782336256 0.439626644
0.828724118 -7.422630235 cg21734356 18 3498854
p DLGAP1 DLGAP1 NA 3'UTR shore 3'UTR -
shore NA 0.675134874 0.669173116 -0.005961758
1.008909142
-0.008202439 0.702493074 -0.780709353 0.44056979
0.828724118 -7.423914045 cg09781307 11
27648324 p BDNF BDNFOS NA Body open
sea Body - open sea V$GRE_C 0.705475779 0.69727334 -
0.008202439 1.011763592
-0.001699265 0.063613667 -0.779921399 0.441027017
0.828724118 -7.424534904 cg00920970 6
152129388 q ESR1 ESR1 NA Body island
Body - island V$AP2_Q6;V$HEN1_02;V$E47_01 0.064231582
0.062532317 -0.001699265 1.027174189
-0.004253908 0.898175504 -0.778556526 0.441819688
0.828724118 -7.425608912 cg12760319 2
172541622 q SLC25A12 DYNC1I2 -2360 IGR shelf
IGR - shelf NA 0.899722379 0.895468472
-0.004253908 1.004750482
0.003624383 0.915047821 0.778494477 0.441855744
0.828724118 -7.425657695 cg25743221 6
152501416 q ESR1 SYNE1 NA Body open
sea Body - open sea NA 0.913729863 0.917354246
0.003624383 0.996049091
0.009952476 0.328661955 0.777769791 0.442276983
0.828724118 -7.426227163 cg20208009 22
19974048 q COMT ARVCF NA Body shore
Body - shore NA 0.325042873 0.334995349
0.009952476 0.970290704
0.002320588 0.052050086 0.777637728 0.442353773
0.828724118 -7.426330885 cg06380702 6

29720825 p MOG IFITM4P 2241 IGR island
IGR - island NA 0.051206236 0.053526824
0.002320588 0.95664626
-0.001768427 0.079173413 -0.776264398 0.443152793
0.828724118 -7.427408488 cg19690984 6
29521631 p GABBR1 UBD -1758 IGR island
IGR - island NA 0.079816478 0.07804805 -
0.001768427 1.022658196
0.001686029 0.02551692 0.775337707 0.443692441
0.828724118 -7.428134593 cg24847163 6
29600980 p GABBR1/MOG GABBR1 NA TSS200 shore
TSS200 - shore NA 0.024903818 0.026589848
0.001686029 0.936591213
-0.03801888 0.643652896 -0.773071918 0.445013548
0.828724118 -7.42990642 cg00588198 6
29648452 p GABBR1/MOG ZFP57 8283 IGR open
sea IGR - open sea NA 0.657477943 0.619459063
-0.03801888 1.061374322
0.013464063 0.829206631 0.772509752 0.445341691
0.828724118 -7.430345254 cg13504410 2
172546870 q SLC25A12 DYNC1I2 NA Body shelf
Body - shelf NA 0.824310608 0.837774671
0.013464063 0.983928778
-0.005032139 0.40870913 -0.772456177 0.445372972
0.828724118 -7.43038706 cg06014588 15
88801339 q NTRK3 NTRK3-AS1 5378 IGR shore
IGR - shore NA 0.410538999 0.40550686 -
0.005032139 1.012409504
-0.004967296 0.834515083 -0.771066168 0.446184991
0.828724118 -7.431470727 cg02039214 15
88521695 q NTRK3 NTRK3 NA Body open
sea Body - open sea NA 0.836321372 0.831354076
-0.004967296 1.005974946
0.006518012 0.072856372 0.768398734 0.447745728
0.828724118 -7.433545016 cg11257193 17
28444188 q SLC6A4 MIR423 NA Body shore
Body - shore NA 0.070486186 0.077004197
0.006518012 0.91535512
0.002908502 0.916623036 0.768151366 0.44789063
0.828724118 -7.433737027 cg15320474 6
29528005 p GABBR1/MOG UBD NA TSS1500 open
sea TSS1500 - open sea NA 0.915565399 0.918473901
0.002908502 0.996833332
0.003565556 0.878013995 0.767274145 0.448404706
0.828724118 -7.434417458 cg16324072 22
19978018 q COMT ARVCF NA Body shelf
Body - shelf NA 0.87671743 0.880282986
0.003565556 0.995949534
0.005676705 0.827868761 0.765080065 0.449692033
0.828724118 -7.436116048 cg17606558 6
29456563 p GABBR1 MAS1L NA TSS1500 open
sea TSS1500 - open sea NA 0.825804505 0.83148121
0.005676705 0.99317278
0.004062617 0.100630637 0.764598771 0.449974715
0.828724118 -7.436488024 cg10810078 2
172543808 q SLC25A12 DYNC1I2 NA TSS200 shore

TSS200 - shore NA 0.099153322 0.103215939
0.004062617 0.960639635
0.009716815 0.411693695 0.763985562 0.450335027
0.828724118 -7.436961625 cg13468667 18 3411996
p DLGAP1 TGIF1 NA TSS200 open sea TSS200
- open sea NA 0.408160308 0.417877123 0.009716815
0.976747196
0.004430659 0.841181941 0.762112176 0.45143686
0.828724118 -7.43840623 cg26310969 17
28662096 q SLC6A4 TMIGD1 NA TSS1500 open
sea TSS1500 - open sea NA 0.839570792 0.844001451
0.004430659 0.994750413
0.003204597 0.085646729 0.761935824 0.451540663
0.828724118 -7.438542041 cg04352676 6
29596516 p GABBR1/MOG GABBR1 NA TSS1500 shore
TSS1500 - shore NA 0.084481421 0.087686018
0.003204597 0.963453729
0.00419478 0.100358478 0.761566542 0.451758074
0.828724118 -7.438826335 cg00655307 6
152128743 q ESR1 ESR1 NA 5'UTR shore
5'UTR - shore NA 0.098833103 0.103027883
0.00419478 0.959285002
0.004545481 0.918286599 0.76066982 0.452286268
0.828724118 -7.439516126 cg04847841 21
34351148 q OLIG2 OLIG2 -47068 IGR shore
IGR - shore NA 0.916633696 0.921179178
0.004545481 0.995065583
-0.002690148 0.917062806 -0.76014404 0.452596136
0.828724118 -7.43992021 cg18761756 18 3732002
p DLGAP1 DLGAP1 NA Body shelf Body -
shelf NA 0.918041041 0.915350893 -0.002690148
1.002938925
0.011448186 0.291429257 0.758352992 0.453652631
0.828724118 -7.441294682 cg19352507 6
29717262 p MOG LOC285830 NA TSS1500 shore
TSS1500 - shore NA 0.28726628 0.298714466
0.011448186 0.961675154
0.002781056 0.901234556 0.758272506 0.453700142
0.828724118 -7.441356375 cg25396488 6
29641118 p GABBR1/MOG ZFP57 NA Body open
sea Body - open sea NA 0.900223263 0.903004319
0.002781056 0.996920218
-0.003350928 0.029971415 -0.758215429 0.453733837
0.828724118 -7.44140012 cg15126544 2
171678954 q GAD1 GAD1 NA Body island
Body - island NA 0.031189935 0.027839007
-0.003350928 1.120368086
0.003732171 0.914237547 0.757103948 0.454390272
0.828724118 -7.442251361 cg05598246 6
29624414 p GABBR1/MOG MOG NA TSS1500 open
sea TSS1500 - open sea NA 0.912880394 0.916612565
0.003732171 0.9959283
-0.003926262 0.123528601 -0.756750949 0.454598869
0.828724118 -7.442521457 cg00674706 6
29521145 p GABBR1 UBD -2244 IGR island
IGR - island NA 0.124956333 0.121030071
-0.003926262 1.032440384

-0.001570389 0.017576064 -0.755432502 0.455378475
0.828724118 -7.443529187 cg12173216 6
29720762 p MOG IFITM4P 2178 IGR island
IGR - island NA 0.018147114 0.016576725
-0.001570389 1.094734575
-0.005009977 0.811912856 -0.755132551 0.455555948
0.828724118 -7.443758213 cg13570637 22
20052305 q COMT C22orf25 NA 3'UTR open
sea 3'UTR - open sea NA 0.813734666 0.808724689
-0.005009977 1.00619491
-0.002080444 0.068744641 -0.753384906 0.456590791
0.829469866 -7.445090864 cg00298481 11
27722063 p BDNF BDNF NA Body island
Body - island NA 0.069501166 0.067420722
-0.002080444 1.030857635
-0.006096326 0.847626679 -0.750342302 0.458395719
0.829469866 -7.447403859 cg02547724 6
29455365 p GABBR1 MAS1L NA 1stExon open
sea 1stExon - open sea NA 0.849843525 0.843747199
-0.006096326 1.007225299
-0.003186661 0.095478819 -0.749893169 0.458662508
0.829469866 -7.447744525 cg06841846 17
28564094 q SLC6A4 SLC6A4 NA TSS1500 shore
TSS1500 - shore NA 0.096637604 0.093450943
-0.003186661 1.034099827
0.009836483 0.274757852 0.749294726 0.459018128
0.829469866 -7.448198136 cg07664000 17
28659293 q SLC6A4 TMIGD1 NA 5'UTR open
sea 5'UTR - open sea NA 0.271180949 0.281017431
0.009836483 0.964996897
0.022274918 0.332244525 0.746713212 0.460554028
0.829469866 -7.450150875 cg10915739 21
34405733 q OLIG2 OLIG2 7517 IGR island
IGR - island NA 0.324144555 0.346419473
0.022274918 0.935699579
-0.004762787 0.172383561 -0.742963675 0.462790201
0.829469866 -7.45297554 cg18584905 17
28563300 q SLC6A4 SLC6A4 NA TSS1500 shore
TSS1500 - shore NA 0.174115483 0.169352696
-0.004762787 1.028123479
-0.005032831 0.849656372 -0.742950063 0.462798331
0.829469866 -7.452985769 cg06045576 22
19948957 q COMT COMT NA 5'UTR open
sea 5'UTR - open sea NA 0.851486492 0.846453661
-0.005032831 1.005945784
0.006851908 0.875319846 0.742300035 0.463186646
0.829469866 -7.453474052 cg23266797 6
29636003 p GABBR1/MOG MOG NA 3'UTR open
sea 3'UTR - open sea NA 0.872828243 0.879680151
0.006851908 0.99221091
0.032958855 0.578616205 0.741262003 0.463807142
0.829469866 -7.454252937 cg08065408 6
29520774 p GABBR1 UBD -2615 IGR shore
IGR - shore NA 0.566631167 0.599590022
0.032958855 0.945031015
0.002535951 0.055172192 0.739189065 0.465047714
0.829469866 -7.455805206 cg19622911 18 3771570

p DLGAP1 DLGAP1 NA Body island Body -
island NA 0.054250028 0.056785979 0.002535951
0.955341952
0.007982515 0.737566757 0.738974269 0.465176371
0.829469866 -7.45596581 cg12546695 6
29425610 p GABBR1 OR2H1 NA TSS1500 open
sea TSS1500 - open sea NA 0.734664024 0.742646539
0.007982515 0.989251259
0.008473891 0.708622907 0.737832995 0.465860312
0.829469866 -7.456818391 cg12508693 11
27657625 p BDNF BDNFOS NA Body open
sea Body - open sea NA 0.705541492 0.714015383
0.008473891 0.988132061
0.002199196 0.081005825 0.73781534 0.465870897
0.829469866 -7.45683157 cg03409187 6
29521624 p GABBR1 UBD -1765 IGR island
IGR - island NA 0.080206117 0.082405313
0.002199196 0.973312449
-0.001806278 0.031217493 -0.737022288 0.466346506
0.829469866 -7.45742325 cg14888846 17
28443962 q SLC6A4 MIR423 NA TSS200 island
TSS200 - island NA 0.031874322 0.030068044
-0.001806278 1.060073013
-0.006408913 0.179011974 -0.736640908 0.466575327
0.829469866 -7.45770757 cg21446343 22
19974866 q COMT ARVCF NA Body shore
Body - shore NA 0.181342488 0.174933575
-0.006408913 1.036636266
0.00318352 0.060042161 0.736613388 0.466591842
0.829469866 -7.457728081 ch.2.171328329F 2
171620083 q GAD1 SP5 48226 IGR open
sea IGR - open sea NA 0.058884517 0.062068037
0.00318352 0.948709188
0.008116422 0.280345342 0.735917631 0.467009461
0.829469866 -7.458246386 cg11565911 12
72233249 q TPH2 TBC1D15 NA TSS1500 shore
TSS1500 - shore NA 0.277393916 0.285510338
0.008116422 0.971572231
0.00175107 0.022373241 0.735259057 0.467404961
0.829469866 -7.458736554 cg14402472 6
29691165 p GABBR1/MOG HLA-F NA 1stExon island
1stExon - island NA 0.021736488 0.023487558
0.00175107 0.925446911
0.003106901 0.93416198 0.734694953 0.467743882
0.829469866 -7.459156072 cg24952408 6
29696264 p MOG LOC285830 NA Body open
sea Body - open sea NA 0.933032198 0.936139099
0.003106901 0.996681155
-0.010926128 0.634884694 -0.733654229 0.468369537
0.829469866 -7.45992923 cg26272069 6
29591706 p GABBR1/MOG GABBR1 NA Body shelf
Body - shelf NA 0.638857831 0.627931704
-0.010926128 1.017400184
0.001486913 0.029486662 0.73310655 0.468698981
0.829469866 -7.460335677 cg13293756 2
172778961 q SLC25A12 HAT1 NA 1stExon island

1stExon - island	NA	0.028945967	0.03043288
0.001486913	0.951141233
0.001787768	0.041627643	0.730581756	0.47021945
0.831085539	-7.462205592	cg16489427 6
29721015	p	MOG	IFITM4P	2431	IGR	island
IGR - island	NA	0.040977546	0.042765313
0.001787768	0.958195863
0.002671351	0.0810138	0.727065484	0.472341743
0.83375936 -7.464799414	cg05645755 18	4454548	p
DLGAP1	DLGAP1-AS5 189946	IGR	island	IGR - island
NA	0.0800424	0.082713751	0.002671351
0.967703665
-0.001566685	0.031777051	-0.719842241	0.4767187
0.839414884	-7.470089709	cg25638186 6
29720443	p	MOG	IFITM4P	1859	IGR	island
IGR - island	NA	0.032346754	0.030780069
-0.001566685	1.050899334
-0.003831709	0.916542285	-0.719752988	0.476772928
0.839414884	-7.470154757	cg05087623 6
29527870	p	GABBR1/MOG UBD	NA	TSS200	open
sea	TSS200 - open sea	NA	0.917935634	0.914103925
-0.003831709	1.004191765
0.005135452	0.834986022	0.718086647	0.477786011
0.840117304	-7.471367774	cg22965752 6
29425885	p	GABBR1	OR2H1	NA	TSS1500	open
sea	TSS1500 - open sea	NA	0.833118585	0.838254037
0.005135452	0.993873633
0.003518488	0.886687739	0.71632516 0.478858277
0.840748858	-7.472647089	cg08921491 15
88577641	q	NTRK3	NTRK3	NA	Body	open
sea	Body - open sea	V$POU6F1_01	0.885408288
0.888926777	0.003518488	0.996041869
-0.051521427	0.68532103 -0.715478176	0.479374349
0.840748858	-7.473261145	cg07134666 6
29648400	p	GABBR1/MOG ZFP57	8231	IGR	open
sea	IGR - open sea	NA	0.704056094	0.652534668
-0.051521427	1.078955845
0.003528252	0.044636632	0.712745679	0.481041437
0.841878404	-7.475237378	cg24377657 11
27723245	p	BDNF	BDNF	NA	TSS1500	shore
TSS1500 - shore	NA	0.043353631	0.046881883
0.003528252	0.924741675
0.003348299	0.9166472	0.711202097	0.481984629
0.841878404	-7.476350508	cg24437600 12
72361228	q	TPH2	TPH2	NA	Body	open
sea	Body - open sea	NA	0.915429637	0.918777936
0.003348299	0.996355704
-0.002834206	0.92730768 -0.710677319	0.48230553 0.841878404
-7.47672841	cg17675298 22	19961051	q	COMT
ARVCF	NA	Body	island	Body - island	NA
0.9283383	0.925504094	-0.002834206	1.003062338
0.004056612	0.882386627	0.710317145	0.482525846
0.841878404	-7.476987622	cg24430207 6
29589714	p	GABBR1/MOG GABBR1	NA	Body	open
sea	Body - open sea	NA	0.880911495	0.884968107
0.004056612	0.995416092

0.002796694 0.934465074 0.70938642 0.483095429
0.841878404 -7.47765686 cg25504668 6
29572379 p GABBR1/MOG GABBR1 NA Body open
sea Body - open sea NA 0.933448095 0.936244788
0.002796694 0.997012861
-0.005163638 0.924506301 -0.707176356 0.484449469
0.843163961 -7.479242598 cg18860567 6
29578925 p GABBR1/MOG GABBR1 NA Body open
sea Body - open sea NA 0.926383987 0.921220349
-0.005163638 1.005605215
0.00272207 0.893861426 0.704637913 0.486007348
0.844441958 -7.481058027 cg24937995 21
34448038 q OLIG2 OLIG1 5588 IGR shelf
IGR - shelf NA 0.892871583 0.895593653
0.00272207 0.996960597
0.003473524 0.071506468 0.703969034 0.486418321
0.844441958 -7.481535338 cg06412358 21
34392373 q OLIG2 OLIG2 -5843 IGR island
IGR - island NA 0.070243369 0.073716892
0.003473524 0.95288023
-0.001939625 0.065695538 -0.702545476 0.487293637
0.844889348 -7.482549723 cg24759658 12
72233341 q TPH2 TBC1D15 NA TSS200 island
TSS200 - island NA 0.066400857 0.064461232
-0.001939625 1.030089791
-0.01413069 0.592549089 -0.700059432 0.488824385
0.84584174 -7.484316425 cg14791008 12 72332386 q
TPH2 TPH2 NA TSS1500 open sea TSS1500 - open
sea NA 0.597687522 0.583556832 -0.01413069
1.024214762
-0.004296366 0.1681454 -0.698871616 0.489556722
0.84584174 -7.485158398 cg03628748 9 87285133 q
NTRK2 NTRK2 NA 5'UTR island 5'UTR - island
NA 0.169707715 0.165411349 -0.004296366
1.025973828
-0.004816605 0.199826819 -0.69864283 0.48969785
0.84584174 -7.485320411 cg09990446 6 29716108 p
MOG LOC285830 NA Body shore Body - shore
NA 0.201578312 0.196761707 -0.004816605
1.024479382
0.002515815 0.070282794 0.692603098 0.49343174
0.850241812 -7.48957879 cg14665414 6
29596574 p GABBR1/MOG GABBR1 NA TSS1500 shore
TSS1500 - shore NA 0.069367952 0.071883767
0.002515815 0.965001625
0.004719058 0.883997618 0.692511802 0.493488303
0.850241812 -7.489642883 cg24782003 18 4459167
p DLGAP1 DLGAP1-AS5 194565 IGR shelf IGR -
shelf NA 0.882281597 0.887000655 0.004719058
0.994679758
0.002231447 0.03150477 0.689952137 0.495075645
0.850262675 -7.49143654 cg09859456 12
72234313 q TPH2 TBC1D15 NA Body shore
Body - shore NA 0.030693335 0.032924781
0.002231447 0.932225943
0.003154765 0.845675217 0.686233374 0.497386861
0.850262675 -7.494030925 cg04960880 6

29577006 p GABBR1/MOG GABBR1 NA Body open
sea Body - open sea NA 0.84452803 0.847682795
0.003154765 0.996278366
0.010685946 0.443792923 0.683122779 0.499324709
0.850262675 -7.496190558 cg18731680 22
19953712 q COMT COMT NA Body open
sea Body - open sea NA 0.439907125 0.450593071
0.010685946 0.976284709
-0.037611026 0.689135552 -0.682646033 0.499622084
0.850262675 -7.496520712 cg02157626 6
29648736 p GABBR1/MOG ZFP57 8567 IGR open
sea IGR - open sea NA 0.702812289 0.665201263
-0.037611026 1.056540822
0.005109604 0.794713688 0.68216162 0.499924343
0.850262675 -7.496855947 cg14298020 6
29712462 p MOG LOC285830 NA Body open
sea Body - open sea NA 0.79285565 0.797965254
0.005109604 0.993596709
0.003926376 0.176252438 0.681751805 0.500180133
0.850262675 -7.497139375 cg17781710 2
171671480 q GAD1 GAD1 -1720 IGR shore
IGR - shore NA 0.174824665 0.178751041
0.003926376 0.978034388
0.006111453 0.869963928 0.681566392 0.500295885
0.850262675 -7.497267553 cg07379508 2
171630114 q GAD1 GAD1 -43086 IGR shelf
IGR - shelf NA 0.867741582 0.873853035
0.006111453 0.993006315
0.006055306 0.134472059 0.680047279 0.501244812
0.850262675 -7.498316451 cg24900983 6
152128528 q ESR1 ESR1 NA TSS1500 shore
TSS1500 - shore NA 0.132270129 0.138325435
0.006055306 0.956224204
-0.009851574 0.655064807 -0.680024142 0.501259272
0.850262675 -7.498332408 cg17514431 6
29717656 p MOG LOC285830 NA TSS1500 shore
TSS1500 - shore NA 0.658647197 0.648795623
-0.009851574 1.015184403
0.004451019 0.079196288 0.679564411 0.501546648
0.850262675 -7.498649379 cg03747251 11
27722722 p BDNF BDNF NA TSS200 shore
TSS200 - shore NA 0.077577735 0.082028754
0.004451019 0.945738308
0.001894234 0.060371195 0.679451565 0.501617202
0.850262675 -7.498727151 cg06358612 17
28619293 q SLC6A4 BLMH NA TSS1500 shore
TSS1500 - shore NA 0.059682383 0.061576616
0.001894234 0.969237787
-0.011771607 0.647161064 -0.677354509 0.502929322
0.850262675 -7.50017013 cg21100518 6
29595002 p GABBR1/MOG GABBR1 NA Body shore
Body - shore NA 0.651441648 0.639670041
-0.011771607 1.018402624
0.00433985 0.920217231 0.677216932 0.50301547 0.850262675
-7.500264645 cg04892672 6 29695164 p
GABBR1/MOG LOC285830 NA Body shelf Body - shelf

NA 0.918639104 0.922978954 0.00433985
0.995297997
0.006876091 0.874340594 0.677016721 0.503140851
0.850262675 -7.500402155 cg00539542 6
29644544 p GABBR1/MOG ZFP57 NA Body open
sea Body - open sea NA 0.871840197 0.878716289
0.006876091 0.992174844
-0.004573135 0.888275449 -0.67686298 0.503237144
0.850262675 -7.500507722 cg13460297 2
172581104 q SLC25A12 DYNC1I2 NA Body open
sea Body - open sea NA 0.889938408 0.885365273
-0.004573135 1.005165252
0.003019818 0.910349602 0.676467023 0.503485189
0.850262675 -7.5007795 cg06564500 22 19892663 q
COMT TXNRD2 NA Body shore Body - shore
NA 0.909251487 0.912271304 0.003019818
0.996689782
-0.001854078 0.036274638 -0.675502656 0.504089595
0.850262675 -7.501440778 cg27066254 17
28443640 q SLC6A4 CCDC55 NA TSS200 shore
TSS200 - shore NA 0.036948848 0.03509477 -
0.001854078 1.052830607
0.008548855 0.512506377 0.674548367 0.504688079
0.850262675 -7.502094243 cg22584138 17
28562220 q SLC6A4 SLC6A4 NA 5'UTR shore
5'UTR - shore NA 0.509397703 0.517946558
0.008548855 0.983494716
-0.003937832 0.865089533 -0.670630653 0.50714918
0.852276119 -7.504767542 cg18595174 11
27701991 p BDNF BDNF NA Body open
sea Body - open sea NA 0.866521472 0.86258364 -
0.003937832 1.00456516
0.001225932 0.015848043 0.669176147 0.508064575
0.852276119 -7.505756186 cg24710870 6
29720670 p MOG IFITM4P 2086 IGR island
IGR - island NA 0.015402249 0.016628181
0.001225932 0.926273836
0.003597929 0.080620597 0.66846559 0.508512095
0.852276119 -7.5062384 cg10558494 11 27721280 p
BDNF BDNF NA Body shore Body - shore
NA 0.079312259 0.082910188 0.003597929
0.956604501
-0.003270835 0.845147793 -0.666762218 0.509585783
0.852276119 -7.507392349 cg19382714 6
29624846 p GABBR1/MOG MOG NA 1stExon open
sea 1stExon - open sea NA 0.846337188 0.843066353
-0.003270835 1.003879689
-0.003707709 0.916403564 -0.666329621 0.50985866
0.852276119 -7.507684956 cg15242223 6
29573377 p GABBR1/MOG GABBR1 NA Body open
sea Body - open sea NA 0.917751821 0.914044112
-0.003707709 1.004056379
0.003031127 0.138906918 0.666052955 0.51003322
0.852276119 -7.507871995 cg23009221 6
152128588 q ESR1 ESR1 NA TSS1500 shore
TSS1500 - shore NA 0.13780469 0.140835816
0.003031127 0.978477591

0.001673654 0.039371675 0.665718546 0.510244256
0.852276119 -7.50809797 cg19348622 6
29691760 p GABBR1/MOG HLA-F NA Body island
Body - island NA 0.038763073 0.040436727
0.001673654 0.958610547
0.005861139 0.730915915 0.660292791 0.513674955
0.854644359 -7.511748951 cg15198068 22
19843949 q COMT GNB1L NA TSS1500 shore
TSS1500 - shore NA 0.728784592 0.734645731
0.005861139 0.992021816
0.005313076 0.189181293 0.660059345 0.513822844
0.854644359 -7.511905384 ch.6.2958553R 6
152519966 q ESR1 SYNE1 NA Body open
sea Body - open sea NA 0.187249265 0.192562341
0.005313076 0.972408541
-0.001984132 0.085224969 -0.660051043 0.513828103
0.854644359 -7.511910945 cg01779447 6
29720315 p MOG IFITM4P NA TSS1500 shore
TSS1500 - shore NA 0.085946471 0.08396234 -
0.001984132 1.023631202
0.004541163 0.871849608 0.659396385 0.514242958
0.854644359 -7.512349341 cg22115805 15
88804912 q NTRK3 NTRK3-AS1 8951 IGR shelf
IGR - shelf NA 0.870198276 0.87473944
0.004541163 0.994808552
0.006131248 0.187118142 0.658093077 0.515069402
0.854644359 -7.513220846 cg17014345 6
29716186 p MOG LOC285830 NA Body shore
Body - shore NA 0.184888598 0.191019846
0.006131248 0.96790256
0.005718011 0.137054486 0.65668044 0.515965987
0.854644359 -7.514163562 cg01382110 18 4454189
p DLGAP1 DLGAP1-AS5 189587 IGR island IGR -
island NA 0.13497521 0.140693221 0.005718011
0.959358305
-0.024765628 0.494037102 -0.655392216 0.516784347
0.854644359 -7.515021532 cg08041448 6
29648901 p GABBR1/MOG ZFP57 8732 IGR open
sea IGR - open sea NA 0.503042785 0.478277157
-0.024765628 1.051780913
-0.039022324 0.697946245 -0.654897073 0.51709908
0.854644359 -7.515350865 cg03449857 6
29648623 p GABBR1/MOG ZFP57 8454 IGR open
sea IGR - open sea NA 0.712136182 0.673113857
-0.039022324 1.057972845
0.004181701 0.885579683 0.65460506 0.517284744
0.854644359 -7.515544978 cg06979684 11
27677125 p BDNF BDNF NA 3'UTR open
sea 3'UTR - open sea NA 0.884059065 0.888240766
0.004181701 0.995292154
0.007431313 0.502220443 0.652058002 0.51890571
0.855298738 -7.517234529 cg16716320 15
88406509 q NTRK3 NTRK3-AS1 -13479 IGR open
sea IGR - open sea NA 0.499518147 0.50694946
0.007431313 0.985341117
0.006519326 0.821389602 0.652017901 0.518931252
0.855298738 -7.517261078 cg25189241 2

171785027 q GAD1 GORASP2 NA TSS1500 shore
TSS1500 - shore NA 0.819018938 0.825538264
0.006519326 0.992102939
-0.003542617 0.078833966 -0.650297182 0.520027911
0.855987179 -7.518398789 cg15753746 2
171679591 q GAD1 GAD1 NA Body island
Body - island NA 0.08012219 0.076579573 -
0.003542617 1.0462606
-0.002886573 0.05263833 -0.649399709 0.52060039 0.855987179
-7.518991021 cg20664238 15 88798877 q NTRK3
NTRK3 NA Body shore Body - shore NA
0.053687993 0.05080142 -0.002886573 1.056820715
0.003752513 0.854458342 0.645415866 0.523145692
0.85655119 -7.521610289 cg02681389 6 29570781 p
GABBR1/MOG GABBR1 NA 3'UTR open sea 3'UTR - open sea
NA 0.853093792 0.856846305 0.003752513
0.995620553
0.002025828 0.08691448 0.643041084 0.524666129
0.85655119 -7.523164168 cg07063032 18 3450262 p
DLGAP1 TGIF1 NA 5'UTR island 5'UTR - island
NA 0.086177815 0.088203643 0.002025828
0.977032377
0.004862915 0.809185212 0.642989849 0.524698958
0.85655119 -7.523197631 cg16902425 6 29526459 p
GABBR1/MOG UBD NA Body open sea Body - open sea
NA 0.807416879 0.812279794 0.004862915
0.994013251
0.004065572 0.916824345 0.64243761 0.525052876
0.85655119 -7.523558146 cg07189962 6 152126092 q
ESR1 ESR1 NA 5'UTR shelf 5'UTR - shelf
NA 0.915345956 0.919411528 0.004065572
0.995578072
0.002706931 0.039287565 0.640730669 0.526147625
0.85655119 -7.52467057 cg07159484 11 27722523 p
BDNF BDNF NA Body island Body - island
NA 0.038303227 0.041010158 0.002706931
0.933993646
0.006229666 0.144251375 0.63976334 0.526768564
0.85655119 -7.525299703 cg00287322 2 171670379 q
GAD1 GAD1 -2821 IGR island IGR - island
NA 0.141986042 0.148215708 0.006229666
0.957968922
0.004056547 0.916220809 0.639054917 0.527223557
0.85655119 -7.52575986 cg24962941 6 29577916 p
GABBR1/MOG GABBR1 NA Body open sea Body - open sea
NA 0.914745701 0.918802248 0.004056547
0.995584962
-0.001117216 0.025148037 -0.637972805 0.527918961
0.85655119 -7.526461788 cg19760323 6 29716554 p
MOG LOC285830 NA Body island Body - island
NA 0.025554298 0.024437082 -0.001117216
1.045718061
0.003184582 0.933639408 0.637922396 0.527951367
0.85655119 -7.526494458 cg25758242 6 29427011 p
GABBR1 OR2H1 NA 5'UTR open sea 5'UTR - open sea
NA 0.932481378 0.93566596 0.003184582
0.996596454

0.003683566 0.891147885 0.63694924 0.528577189
0.85655119 -7.527124669 cg03175417 22 20050298 q
COMT C22orf25 NA Body open sea Body - open sea
NA 0.889808406 0.893491972 0.003683566
0.995877337
-0.002049551 0.062105148 -0.635059654 0.529793483
0.85655119 -7.528345677 cg24025650 6 29720582 p
MOG IFITM4P 1998 IGR island IGR - island
NA 0.06285044 0.060800889 -0.002049551
1.033709228
-0.003446307 0.042748675 -0.632320995 0.531558947
0.85655119 -7.530109053 cg15818307 6 29521430 p
GABBR1 UBD -1959 IGR island IGR - island
NA 0.044001878 0.040555571 -0.003446307
1.084977401
0.008679875 0.630681403 0.632207261 0.531632333
0.85655119 -7.530182124 cg15922678 6 29694484 p
GABBR1/MOG LOC285830 NA Body shelf Body - shelf
NA 0.627525085 0.636204959 0.008679875
0.986356796
0.005330366 0.806447426 0.632137714 0.53167721
0.85655119 -7.530226799 cg06760467 6 29496514 p
GABBR1 LINC01015 -669 IGR open sea IGR - open sea
NA 0.804509111 0.809839477 0.005330366
0.993417997
-0.003600811 0.112823372 -0.631534891 0.532066281
0.85655119 -7.530613839 cg12760563 18 3594396 p
DLGAP1 DLGAP1 NA Body open sea Body - open sea
NA 0.114132757 0.110531947 -0.003600811
1.032577097
0.003315076 0.872401953 0.631020684 0.532398279
0.85655119 -7.530943698 cg13908968 9 87188847 q
NTRK2 NTRK2 -94619 IGR open sea IGR - open sea
NA 0.871196471 0.874511547 0.003315076
0.996209226
0.004528954 0.897721048 0.630324435 0.532847985
0.85655119 -7.531389917 cg13690679 17 28622405 q
SLC6A4 TMIGD1 -20961 IGR shelf IGR - shelf
NA 0.896074155 0.90060311 0.004528954
0.994971198
0.002828646 0.094682679 0.629607946 0.533310974
0.85655119 -7.531848607 cg25725890 17 28563054 q
SLC6A4 SLC6A4 NA TSS200 island TSS200 - island
NA 0.093654081 0.096482727 0.002828646
0.970682359
0.001607686 0.044603385 0.62818252 0.534232706
0.85655119 -7.532759636 cg10190161 2 172778811 q
SLC25A12 HAT1 NA TSS200 island TSS200 - island
V$MIF1_01;V$RFX1_01 0.044018772 0.045626459
0.001607686 0.964764151
-0.003072563 0.097877601 -0.627332088 0.534783025
0.85655119 -7.53330221 cg13933279 15 88418502 q
NTRK3 NTRK3-AS1 -1486 IGR open sea IGR - open sea
NA 0.098994896 0.095922333 -0.003072563
1.032031779
-0.00264727 0.046838339 -0.625911417 0.535703018
0.85655119 -7.534206996 cg10699871 22 19879696 q

COMT TXNRD2 NA Body open sea Body - open sea
NA 0.047800982 0.045153712 -0.00264727
1.05862796
-0.00119544 0.032544725 -0.625892023 0.535715582
0.85655119 -7.534219333 cg20563534 22 20004369 q
COMT ARVCF NA TSS200 island TSS200 - island
NA 0.032979431 0.03178399 -0.00119544
1.03761142
-0.002957225 0.091980532 -0.625483352 0.535980386
0.85655119 -7.534479226 cg25980242 18 4454472 p
DLGAP1 DLGAP1-AS5 189870 IGR island IGR - island
NA 0.093055887 0.090098661 -0.002957225
1.032822086
0.00304568 0.038158728 0.624903692 0.536356102
0.85655119 -7.534847574 cg02149189 6 29521138 p
GABBR1 UBD -2251 IGR island IGR - island
NA 0.037051208 0.040096889 0.00304568
0.924041962
0.003366944 0.209337604 0.622570037 0.537870098
0.85655119 -7.536327131 cg26949694 11 27742060 p
BDNF BDNF NA Body island Body - island
NA 0.20811326 0.211480205 0.003366944
0.984079148
0.007063061 0.348962323 0.621202508 0.538758347
0.85655119 -7.537191645 cg24547396 22 19928740 q
COMT COMT NA TSS1500 shore TSS1500 - shore
NA 0.346393937 0.353456998 0.007063061
0.980017199
0.002059706 0.930873432 0.619728929 0.539716339
0.85655119 -7.538121122 cg15543523 6 152127812 q
ESR1 ESR1 NA TSS1500 shore TSS1500 - shore
NA 0.930124448 0.932184154 0.002059706
0.997790452
-0.008348256 0.57360056 -0.618925771 0.540238858
0.85655119 -7.538626815 cg12991385 6 29599259 p
GABBR1/MOG GABBR1 NA Body shore Body - shore
NA 0.576636289 0.568288033 -0.008348256
1.014690184
0.01130036 0.287792098 0.615693082 0.542344654
0.85655119 -7.540655737 cg23098068 2 172650722 q
SLC25A12 SLC25A12 NA Body open sea Body - open sea
NA 0.283682876 0.294983236 0.01130036
0.961691518
0.00988323 0.22387197 0.614918801 0.542849661 0.85655119 -
7.541140155 cg05009707 6 29555799 p GABBR1/MOG OR2H2
NA 1stExon open sea 1stExon - open sea NA
0.220278068 0.230161298 0.00988323 0.957059549
0.003963806 0.136280618 0.613758503 0.543606897
0.85655119 -7.541864965 cg11718030 11 27744363 p
BDNF BDNF NA TSS1500 island TSS1500 - island
NA 0.134839234 0.13880304 0.003963806
0.971442945
-0.003517666 0.915008843 -0.613654831 0.543674583
0.85655119 -7.541929661 cg03200120 6 29641443 p
GABBR1/MOG ZFP57 NA Body open sea Body - open sea
NA 0.916287994 0.912770328 -0.003517666
1.003853835

-0.039231764 0.690485367 -0.613628006 0.543692097
0.85655119 -7.541946399 cg15570656 6 29648628 p
GABBR1/MOG ZFP57 8459 IGR open sea IGR - open sea
NA 0.704751463 0.6655197 -0.039231764
1.058949063
0.008751702 0.754461981 0.612813499 0.544224034
0.85655119 -7.542454297 cg19409546 12 72477363 q
TPH2 TPH2 144737 IGR open sea IGR - open sea
NA 0.751279544 0.760031246 0.008751702
0.988485076
0.003071219 0.9202353 0.612161369 0.544650121
0.85655119 -7.542860467 cg15054873 6 152464791 q
ESR1 SYNE1 NA Body open sea Body - open sea
NA 0.919118493 0.922189712 0.003071219
0.996669645
0.004633065 0.811241184 0.611772913 0.544904012
0.85655119 -7.54310221 cg17723549 17 28443042 q
SLC6A4 CCDC55 NA TSS1500 shore TSS1500 - shore
NA 0.809556433 0.814189498 0.004633065
0.994309599
-0.007699471 0.153763727 -0.611487338 0.5450907
0.85655119 -7.543279833 cg17369088 17 28444068 q
SLC6A4 MIR423 NA TSS200 shore TSS200 - shore
NA 0.156563535 0.148864063 -0.007699471
1.051721496
0.001166723 0.025392128 0.610489188 0.54574348
0.85655119 -7.543900029 cg14480858 9 4666499 p
SLC1A1 C9orf68 NA 1stExon shelf 1stExon - shelf
NA 0.024967865 0.026134588 0.001166723
0.955357131
0.004092689 0.09103654 0.608543333 0.547017212
0.85655119 -7.54510623 cg19539318 2 172544773 q
SLC25A12 DYNC1I2 NA 5'UTR shore 5'UTR - shore
NA 0.089548289 0.093640978 0.004092689
0.956293825
-0.004894478 0.083628866 -0.60778646 0.547513067
0.85655119 -7.545574385 cg25627226 18 4455337 p
DLGAP1 DLGAP1-AS5 190735 IGR island IGR - island
NA 0.085408677 0.080514198 -0.004894478
1.06079026
0.005634315 0.500780571 0.607190293 0.547903801
0.85655119 -7.545942736 cg08093277 6 29595299 p
GABBR1/MOG GABBR1 NA Body island Body - island
NA 0.498731729 0.504366044 0.005634315
0.988828917
-0.001330076 0.051998671 -0.60655125 0.548322797
0.85655119 -7.546337187 cg20973396 6 29691899 p
GABBR1/MOG HLA-F NA Body island Body - island
NA 0.052482335 0.051152258 -0.001330076
1.026002313
0.003691468 0.106201827 0.606048043 0.548652847
0.85655119 -7.546647507 cg24567537 6 29520527 p
GABBR1 UBD -2862 IGR shore IGR - shore
NA 0.104859475 0.108550943 0.003691468
0.96599322
-0.004043909 0.84471255 -0.605428152 0.549059571
0.85655119 -7.547029437 cg24727561 6 29524112 p

GABBR1 UBD NA Body shelf Body - shelf
NA 0.846183062 0.842139153 -0.004043909
1.004801949
-0.001283843 0.017236179 -0.605336925 0.54911944
0.85655119 -7.547085612 cg00845219 22 19842449 q
COMT GNB1L NA 1stExon island 1stExon - island
NA 0.017703031 0.016419187 -0.001283843
1.078191691
-0.003252409 0.077472292 -0.600680993 0.55217943
0.86034335 -7.549941609 cg25412831 11 27742138 p
BDNF BDNF NA Body island Body - island
NA 0.078654986 0.075402577 -0.003252409
1.043133924
-0.003021093 0.083892233 -0.598749699 0.553451289
0.861343985 -7.551119954 cg01642653 11
27743476 p BDNF BDNF NA TSS1500 island
TSS1500 - island V$BRACH_01 0.084990812 0.081969719
-0.003021093 1.036856208
-0.004788721 0.120320245 -0.595237032 0.555768408
0.863967252 -7.553253631 cg21346639 18 3447463
p DLGAP1 TGIF1 NA 5'UTR shore 5'UTR -
shore NA 0.122061598 0.117272877 -0.004788721
1.040834003
0.001641486 0.043398673 0.591983493 0.557919005
0.866325992 -7.555218946 cg15202447 6
29720635 p MOG IFITM4P 2051 IGR island
IGR - island NA 0.042801768 0.044443255
0.001641486 0.963065554
0.003493753 0.907598811 0.58674075 0.561393359
0.868815425 -7.558363663 cg13615337 6
29427915 p GABBR1 OR2H1 NA 5'UTR open
sea 5'UTR - open sea NA 0.906328355 0.909822108
0.003493753 0.99615996
0.00094682 0.023954798 0.58600284 0.561883248 0.868815425
-7.558804079 cg16723445 18 3451456 p DLGAP1
TGIF1 NA 5'UTR island 5'UTR - island NA
0.023610499 0.024557319 0.00094682 0.961444488
0.005051707 0.249740002 0.584727196 0.562730641
0.868815425 -7.559564158 cg03363743 17
28562474 q SLC6A4 SLC6A4 NA 5'UTR island
5'UTR - island NA 0.247903018 0.252954725
0.005051707 0.980029205
0.001898782 0.067176916 0.584087031 0.563156138
0.868815425 -7.559944983 cg01963885 22
20004367 q COMT ARVCF NA TSS200 island
TSS200 - island NA 0.06648645 0.068385232
0.001898782 0.972234034
-0.001703283 0.059855303 -0.583465794 0.563569208
0.868815425 -7.560314156 cg00063945 18 3450342
p DLGAP1 TGIF1 NA 5'UTR island 5'UTR -
island NA 0.060474679 0.058771396 -0.001703283
1.028981496
-0.003083449 0.057682536 -0.583081862 0.563824567
0.868815425 -7.560542117 cg21756465 2
172543834 q SLC25A12 DYNC1I2 NA TSS200 shore
TSS200 - shore NA 0.058803791 0.055720341
-0.003083449 1.05533796

0.002554438 0.105904916 0.582106505 0.564473554
0.868815425 -7.561120579 cg05818894 11
27740078 p BDNF BDNF NA Body shore
Body - shore V$SP1_Q6 0.10497603 0.107530468
0.002554438 0.976244519
0.002244855 0.06046915 0.581483648 0.564888189
0.868815425 -7.561489485 cg03732762 21
34443010 q OLIG2 OLIG1 NA 1stExon island
1stExon - island NA 0.059652839 0.061897694
0.002244855 0.963732817
0.013564041 0.658894935 0.580505761 0.565539477
0.868815425 -7.562067888 cg10433043 6
152432725 q ESR1 SYNE1 -10094 IGR open
sea IGR - open sea NA 0.653962556 0.667526597
0.013564041 0.979680149
0.001436698 0.044355214 0.579104718 0.566473252
0.868815425 -7.562894918 cg23952754 17
28431834 q SLC6A4 EFCAB5 NA Body open
sea Body - open sea NA 0.043832779 0.045269476
0.001436698 0.96826345
-0.004077491 0.117096197 -0.579052148 0.566508303
0.868815425 -7.562925911 cg02115911 21
34395548 q OLIG2 OLIG2 -2668 IGR island
IGR - island NA 0.118578921 0.114501431
-0.004077491 1.035610821
0.005469707 0.736728628 0.577059248 0.567837914
0.869045464 -7.564098833 cg01393604 6
29695590 p GABBR1/MOG LOC285830 NA Body shelf
Body - shelf NA 0.734739644 0.74020935
0.005469707 0.992610596
0.003366938 0.899271227 0.576923058 0.567928834
0.869045464 -7.564178844 cg06538238 6
29588575 p GABBR1/MOG GABBR1 NA Body open
sea Body - open sea NA 0.898046886 0.901413824
0.003366938 0.996264825
-0.001560948 0.068909891 -0.572641097 0.570791149
0.87227491 -7.566685008 cg20927575 6 29716340 p
MOG LOC285830 NA Body shore Body - shore
NA 0.069477508 0.06791656 -0.001560948
1.02298332
0.004071037 0.865881842 0.570669012 0.572111821
0.87227491 -7.567833082 cg13730341 6 29589573 p
GABBR1/MOG GABBR1 NA Body open sea Body - open sea
V$HSF2_01 0.864401465 0.868472502 0.004071037
0.995312417
-0.000903816 0.020636167 -0.569917076 0.572615779
0.87227491 -7.568269809 cg14555167 22 19842472 q
COMT GNB1L NA TSS200 island TSS200 - island
NA 0.020964828 0.020061011 -0.000903816
1.045053412
0.003452938 0.078578781 0.56960476 0.572825163
0.87227491 -7.568451038 cg27316393 6 152128675 q
ESR1 ESR1 NA 5'UTR shore 5'UTR - shore
NA 0.077323167 0.080776105 0.003452938
0.957252977
0.005512192 0.882075655 0.569004877 0.573227444
0.87227491 -7.56879886 cg10122187 22 19938620 q

COMT	COMT	NA	5'UTR	open sea	5'UTR - open sea
NA	0.880071222	0.885583413	0.005512192
0.993775639
0.002789237	0.914423173	0.566410275	0.574968996
0.872350266	-7.570299115	cg15209921 6
29430506	p	GABBR1	OR2H1	NA	3'UTR	open
sea	3'UTR - open sea	NA	0.913408905	0.916198141
0.002789237	0.996955641
0.003874201	0.828467921	0.565494128	0.575584558
0.872350266	-7.570827245	cg13802605 9	4495359
p	SLC1A1	SLC1A1	NA	Body	shelf	Body -
shelf	NA	0.827059121	0.830933322	0.003874201
0.995337531
0.002749402	0.072922862	0.56544151 0.575619922
0.872350266	-7.570857552	cg23164938 6
152128366 q	ESR1	ESR1	NA	TSS1500	shore
TSS1500 - shore	NA	0.07192308 0.074672481
0.002749402	0.963180532
0.007648527	0.546901165	0.565045511	0.575886104
0.872350266	-7.571085553	cg15331332 6
29692111	p	GABBR1/MOG HLA-F	NA	Body	shore
Body - shore	NA	0.544119883	0.55176841
0.007648527	0.986138157
0.004212859	0.862091905	0.563184356	0.577137946
0.872350266	-7.572155035	cg02928278 6
29574918	p	GABBR1/MOG GABBR1	NA	Body	open
sea	Body - open sea	NA	0.860559956	0.864772815
0.004212859	0.995128363
0.002064335	0.057448613	0.562516687	0.577587357
0.872350266	-7.572537856	cg05219421 6
29691003	p	GABBR1/MOG HLA-F	NA	TSS200	shore
TSS200 - shore	NA	0.056697945	0.058762281
0.002064335	0.96486971
-0.001471264	0.093033079	-0.562288863	0.577740746
0.872350266	-7.572668381	cg18293833 6
29600462	p	GABBR1/MOG GABBR1	NA	5'UTR	island
5'UTR - island	NA	0.093568084	0.09209682 -
0.001471264	1.015975188
0.002138095	0.921804475	0.560505861	0.578941895
0.872431333	-7.573688112	cg13654445 9
87636383	q	NTRK2	NTRK2	NA	3'UTR	open
sea	3'UTR - open sea	NA	0.921026986	0.923165081
0.002138095	0.997683952
0.001722897	0.030492977	0.560315926	0.57906992
0.872431333	-7.573796552	cg27034819 15
88799526	q	NTRK3	NTRK3	NA	5'UTR	island
5'UTR - island	V$CDPCR1_01	0.029866469
0.031589366	0.001722897	0.945459589
0.001637753	0.035430264	0.557185615	0.581181897
0.874155147	-7.575578553	cg27306787 22
19879176	q	COMT	TXNRD2	NA	Body	open
sea	Body - open sea	NA	0.034834717	0.03647247
0.001637753	0.955096186
-0.002957652	0.069403993	-0.556255684	0.581810034
0.874155147	-7.57610605	cg23165623 6
152128411 q	ESR1	ESR1	NA	TSS1500	shore

TSS1500 - shore NA 0.070479503 0.067521851
-0.002957652 1.043802887
0.001471495 0.028599734 0.555780555 0.582131095
0.874155147 -7.57637523 cg06046431 11
27744490 p BDNF BDNF NA TSS1500 island
TSS1500 - island V$P53_01 0.028064645 0.02953614
0.001471495 0.950179847
0.001568433 0.036622565 0.551751421 0.584857201
0.877285802 -7.578648824 cg13709765 18 3451484
p DLGAP1 TGIF1 NA 5'UTR island 5'UTR -
island NA 0.036052226 0.037620659 0.001568433
0.958309263
0.001956265 0.042729457 0.549919337 0.586098838
0.878185334 -7.579677279 cg18344922 6
29595661 p GABBR1/MOG GABBR1 NA Body island
Body - island NA 0.042018087 0.043974353
0.001956265 0.955513479
0.001236812 0.041126171 0.543899837 0.590187341
0.882809163 -7.583032723 cg14420670 6
29617961 p GABBR1/MOG MOG -6797 IGR island
IGR - island NA 0.040676421 0.041913233
0.001236812 0.970491133
-0.002350614 0.041886523 -0.543179341 0.590677629
0.882809163 -7.583431919 cg06613392 6
29521595 p GABBR1 UBD -1794 IGR island
IGR - island NA 0.042741291 0.040390678
-0.002350614 1.058196919
0.001505286 0.042837052 0.542528397 0.591120756
0.882809163 -7.583792131 cg01075763 18 3449181
p DLGAP1 TGIF1 NA 5'UTR island 5'UTR -
island V$FOXJ2_01;V$HFH3_01 0.042289675 0.043794961
0.001505286 0.965628785
-0.002925185 0.891671064 -0.540568509 0.592455904
0.882978621 -7.584874112 cg05437995 6
29571875 p GABBR1/MOG GABBR1 NA Body open
sea Body - open sea NA 0.892734768 0.889809583
-0.002925185 1.003287428
-0.001739453 0.036597851 -0.540466954 0.592525127
0.882978621 -7.584930072 cg14128584 6
29600583 p GABBR1/MOG GABBR1 NA 5'UTR island
5'UTR - island NA 0.037230379 0.035490926
-0.001739453 1.049011204
0.003419046 0.095651366 0.536344743 0.595338201
0.885194507 -7.587192816 cg18183163 2
171574141 q GAD1 SP5 NA 3'UTR shore
3'UTR - shore NA 0.094408076 0.097827122
0.003419046 0.965050122
-0.018570394 0.52931083 -0.535996069 0.595576436
0.885194507 -7.587383428 cg08022281 6
29648345 p GABBR1/MOG ZFP57 8176 IGR open
sea IGR - open sea NA 0.5360637 0.517493306 -
0.018570394 1.035885284
-0.002044633 0.139530634 -0.535041222 0.596229074
0.885194507 -7.587904796 cg25962210 11
27721222 p BDNF BDNF NA Body shore
Body - shore NA 0.140274137 0.138229504
-0.002044633 1.014791582

-0.0042786 0.684588009 -0.534498186 0.596600391
0.885194507 -7.588200899 cg16994534 6
29620138 p GABBR1/MOG MOG -4620 IGR shelf
IGR - shelf NA 0.686143863 0.681865263
-0.0042786 1.006274847
-0.001143946 0.024107748 -0.532617053 0.597887525
0.886143157 -7.589224344 cg26151531 22
19842652 q COMT GNB1L NA TSS200 island
TSS200 - island NA 0.024523728 0.023379782
-0.001143946 1.048928857
0.001801987 0.084258186 0.530493334 0.599342228
0.886651451 -7.590375509 cg27079446 18 3449844
p DLGAP1 TGIF1 NA 5'UTR island 5'UTR -
island NA 0.083602918 0.085404905 0.001801987
0.978900662
0.00171758 0.069565024 0.530224127 0.599526749
0.886651451 -7.59052111 cg05603292 6
29691631 p GABBR1/MOG HLA-F NA Body island
Body - island NA 0.068940449 0.070658029
0.00171758 0.975691651
-0.001735932 0.050463067 -0.525884166 0.602505168
0.889866617 -7.592858358 cg23918507 18 3771380
p DLGAP1 DLGAP1 NA Body island Body -
island NA 0.051094316 0.049358383 -0.001735932
1.035169973
-0.001341123 0.042301492 -0.524310362 0.603586955
0.889866617 -7.59370125 cg26057780 11
27721277 p BDNF BDNF NA Body shore
Body - shore NA 0.042789173 0.04144805 -
0.001341123 1.032356721
0.001600346 0.079326348 0.524215474 0.603652208
0.889866617 -7.593751991 cg16728223 6
29691603 p GABBR1/MOG HLA-F NA Body island
Body - island NA 0.078744404 0.08034475
0.001600346 0.980081511
0.003302312 0.056515964 0.519993387 0.606558998
0.889926007 -7.596000566 cg22305167 6
29521420 p GABBR1 UBD -1969 IGR island
IGR - island NA 0.055315124 0.058617436
0.003302312 0.943663315
-0.006917308 0.314387225 -0.519626141 0.606812146
0.889926007 -7.596195306 cg13819127 6
29717368 p MOG LOC285830 NA TSS1500 shore
TSS1500 - shore NA 0.31690261 0.309985302 -
0.006917308 1.022314955
-0.009407542 0.63736959 -0.519477651 0.606914517
0.889926007 -7.596274008 cg11182965 22
19864308 q COMT TXNRD2 NA 3'UTR open
sea 3'UTR - open sea NA 0.640790514 0.631382972
-0.009407542 1.014899898
0.001939918 0.071980433 0.518593454 0.607524257
0.889926007 -7.596742186 cg06378770 6
29521714 p GABBR1 UBD -1675 IGR island
IGR - island NA 0.071275009 0.073214926
0.001939918 0.973503804
0.004277549 0.206492804 0.517356202 0.608377943
0.889926007 -7.597395988 cg21829923 2

171573891 q GAD1 SP5 NA Body island
Body - island V$PAX4_01 0.204937331 0.20921488
0.004277549 0.979554279
0.002403532 0.894411161 0.51634419 0.609076632
0.889926007 -7.597929624 cg23742601 6
29589895 p GABBR1/MOG GABBR1 NA Body open
sea Body - open sea NA 0.89353715 0.895940682
0.002403532 0.997317309
0.00258973 0.057159483 0.514352582 0.61045272 0.889926007
-7.5989768 cg25774457 6 29600114 p GABBR1/MOG GABBR1
NA Body shore Body - shore NA
0.056217763 0.058807493 0.00258973 0.955962585
-0.014714625 0.656295202 -0.513705895 0.610899855
0.889926007 -7.599315968 cg12644888 6
29648360 p GABBR1/MOG ZFP57 8191 IGR open
sea IGR - open sea NA 0.661645975 0.64693135 -
0.014714625 1.022745265
-0.005572513 0.200286195 -0.51344551 0.611079934
0.889926007 -7.599452414 cg02723395 2
171678751 q GAD1 GAD1 NA Body island
Body - island NA 0.202312564 0.19674005 -
0.005572513 1.028324248
0.001296845 0.043231206 0.513168414 0.611271597
0.889926007 -7.599597541 cg20340655 11
27723075 p BDNF BDNF NA TSS1500 shore
TSS1500 - shore NA 0.042759626 0.044056471
0.001296845 0.970564029
-0.003159656 0.080299313 -0.512321872 0.61185731
0.889926007 -7.600040436 cg06515159 21
34400659 q OLIG2 OLIG2 NA 3'UTR shore
3'UTR - shore NA 0.081448279 0.078288623
-0.003159656 1.04035907
0.001350954 0.025393192 0.511293013 0.612569516
0.889926007 -7.600577746 cg23467008 6
152128537 q ESR1 ESR1 NA TSS1500 shore
TSS1500 - shore NA 0.024901936 0.02625289
0.001350954 0.948540751
0.011570686 0.412254027 0.509540993 0.613783196
0.889926007 -7.601490274 cg04836472 21
34405997 q OLIG2 OLIG2 7781 IGR island
IGR - island NA 0.408046505 0.419617191
0.011570686 0.972425615
-0.00247323 0.099452744 -0.509066955 0.614111768
0.889926007 -7.601736643 cg14026788 12
72233266 q TPH2 TBC1D15 NA TSS1500 shore
TSS1500 - shore NA 0.1003521 0.09787887 -0.00247323
1.025268273
-0.002339434 0.047652611 -0.508042945 0.614821821
0.889926007 -7.602268076 cg23912231 6
29691408 p GABBR1/MOG HLA-F NA Body island
Body - island NA 0.048503314 0.04616388 -
0.002339434 1.05067672
0.001836918 0.911521202 0.507978625 0.614866434
0.889926007 -7.602301421 cg02945359 6
29694676 p GABBR1/MOG LOC285830 NA Body shelf
Body - shelf NA 0.910853232 0.91269015
0.001836918 0.997987359

-0.001346209 0.035654852 -0.507434364 0.615243992
0.889926007 -7.602583414 cg21614759 6
152128426 q ESR1 ESR1 NA TSS1500 shore
TSS1500 - shore NA 0.036144382 0.034798173
-0.001346209 1.038686198
-0.001385158 0.05289136 -0.507206568 0.615402048
0.889926007 -7.602701351 cg01453816 6
29600108 p GABBR1/MOG GABBR1 NA Body shore
Body - shore V$TAXCREB_02 0.053395054
0.052009896 -0.001385158 1.026632585
0.001881047 0.051880276 0.506053953 0.616202076
0.890141964 -7.603297298 cg06362065 18 3594098
p DLGAP1 DLGAP1 NA Body open sea Body -
open sea NA 0.051196259 0.053077306 0.001881047
0.96456024
-0.001853522 0.910695635 -0.504345974 0.617388457
0.890645373 -7.604177936 cg07412232 6
29495773 p GABBR1 LINC01015 -1410 IGR open
sea IGR - open sea NA 0.911369643 0.90951612 -
0.001853522 1.002037922
-0.003024967 0.878479222 -0.503244372 0.618154196
0.890645373 -7.604744369 cg25693099 18 3879303
p DLGAP1 DLGAP1 NA 1stExon island 1stExon
- island NA 0.87957921 0.876554243 -0.003024967
1.003450975
0.003378027 0.878702377 0.502741734 0.618503731
0.890645373 -7.605002415 cg04480313 6
29455322 p GABBR1 MAS1L NA 1stExon open
sea 1stExon - open sea NA 0.877474003 0.88085203
0.003378027 0.996165046
0.001174713 0.026863646 0.500586029 0.620003836
0.891866717 -7.606106241 cg19339932 22
20004356 q COMT ARVCF NA TSS200 island
TSS200 - island NA 0.026436477 0.02761119
0.001174713 0.957455184
0.001060271 0.0289155 0.499221544 0.620954206
0.892295539 -7.606802509 cg13924755 6
29691755 p GABBR1/MOG HLA-F NA Body island
Body - island NA 0.028529947 0.029590218
0.001060271 0.964168192
0.003598563 0.080731555 0.49653868 0.622824762
0.893356183 -7.60816606 cg21164232 18 3452359
p DLGAP1 TGIF1 NA 5'UTR island 5'UTR -
island NA 0.079422987 0.08302155 0.003598563
0.956655073
-0.002001534 0.928978542 -0.495541927 0.623520371
0.893356183 -7.60867081 cg22098375 6
29590966 p GABBR1/MOG GABBR1 NA Body open
sea Body - open sea NA 0.929706373 0.927704838
-0.002001534 1.002157513
0.004101872 0.908221849 0.494773195 0.624057091
0.893356183 -7.609059409 cg04369302 6
29581439 p GABBR1/MOG GABBR1 NA Body open
sea Body - open sea NA 0.90673026 0.910832132
0.004101872 0.995496566
-0.002604277 0.057133115 -0.494070844 0.624547647
0.893356183 -7.609413933 cg12448003 11

27742365 p BDNF BDNF NA Body shore
Body - shore NA 0.058080125 0.055475848
-0.002604277 1.046944339
0.00332189 0.083290325 0.493484223 0.624957505
0.893356183 -7.609709659 cg25166896 22
20009063 q COMT C22orf25 NA 5'UTR island
5'UTR - island NA 0.082082365 0.085404254
0.00332189 0.96110394
-0.00276798 0.879938534 -0.490123583 0.627307839
0.895779879 -7.611397148 cg19931596 2
172645256 q SLC25A12 SLC25A12 NA Body open
sea Body - open sea NA 0.880945072 0.878177092
-0.00276798 1.003151961
-0.008616404 0.484260828 -0.488980335 0.628108298
0.895987645 -7.611968621 cg11834473 6
29591753 p GABBR1/MOG GABBR1 NA Body shelf
Body - shelf NA 0.487394066 0.478777661
-0.008616404 1.017996673
-0.002815259 0.925066452 -0.48663077 0.629754812
0.897400607 -7.613138963 cg18773129 22
19938916 q COMT COMT NA 5'UTR open
sea 5'UTR - open sea NA 0.926090183 0.923274924
-0.002815259 1.00304921
0.005374068 0.704808292 0.4853278 0.63066873 0.897767766
-7.613785591 cg17930583 6 29591082 p
GABBR1/MOG GABBR1 NA Body open sea Body - open sea
NA 0.702854085 0.708228153 0.005374068
0.992411954
0.002544667 0.076820657 0.478810326 0.635249018
0.903347875 -7.616994374 cg13612689 6
152128634 q ESR1 ESR1 NA 5'UTR shore
5'UTR - shore NA 0.075895324 0.078439991
0.002544667 0.967559061
0.002590329 0.718508447 0.47701831 0.636510967
0.903967014 -7.617869149 cg05114858 6
29526343 p GABBR1/MOG UBD NA Body open
sea Body - open sea NA 0.717566509 0.720156838
0.002590329 0.996403104
-0.004021861 0.88680504 -0.47631578 0.637005995
0.903967014 -7.618211207 cg15628633 6
29706377 p MOG LOC285830 NA Body open
sea Body - open sea NA 0.888267535 0.884245673
-0.004021861 1.004548354
-0.002519838 0.058543489 -0.474374657 0.638374661
0.904970504 -7.619153745 cg05522774 21
34443443 q OLIG2 OLIG1 NA 1stExon island
1stExon - island NA 0.059459794 0.056939955
-0.002519838 1.04425432
0.002624471 0.909404349 0.472258624 0.639868124
0.905393914 -7.620176888 cg19700470 6
29589973 p GABBR1/MOG GABBR1 NA Body open
sea Body - open sea NA 0.908449996 0.911074466
0.002624471 0.997119368
-0.00229693 0.044422731 -0.472076107 0.639997014
0.905393914 -7.620264927 cg21010859 11
27740161 p BDNF BDNF NA Body shore

Body - shore NA 0.045257978 0.042961048
-0.00229693 1.053465409
-0.006792634 0.840895844 -0.468657814 0.64241303
0.907301362 -7.621907576 cg07989678 2
172543677 q SLC25A12 DYNC1I2 NA TSS1500 shore
TSS1500 - shore NA 0.843365892 0.836573258
-0.006792634 1.008119593
0.006113359 0.391697638 0.468292031 0.642671798
0.907301362 -7.622082654 cg07420274 2
171676306 q GAD1 GAD1 NA Body shore
Body - shore NA 0.389474598 0.395587957
0.006113359 0.984546145
-0.000816418 0.031526538 -0.464506685 0.645352338
0.907671622 -7.623886549 cg16548348 22
19842481 q COMT GNB1L NA TSS200 island
TSS200 - island NA 0.031823417 0.031006999
-0.000816418 1.02633012
-0.001150763 0.031257123 -0.463898343 0.645783579
0.907671622 -7.624175105 cg04661128 9 4661938
p SLC1A1 PPAPDC2 NA TSS1500 shore TSS1500
- shore NA 0.031675582 0.030524819 -0.001150763
1.037699257
-0.002421538 0.893506594 -0.463709302 0.645917611
0.907671622 -7.624264697 cg15983385 6
29589056 p GABBR1/MOG GABBR1 NA Body open
sea Body - open sea NA 0.894387153 0.891965615
-0.002421538 1.002714833
0.004077779 0.208992054 0.463639622 0.645967018
0.907671622 -7.624297711 cg27293992 2
171671648 q GAD1 GAD1 -1552 IGR island
IGR - island NA 0.207509226 0.211587005
0.004077779 0.980727649
0.001142842 0.048180629 0.46138582 0.64756597 0.907671622
-7.62536292 cg12766058 6 29720894 p MOG
IFITM4P 2310 IGR island IGR - island NA
0.04776505 0.048907892 0.001142842 0.976632769
0.002788651 0.895856528 0.460514289 0.648184731
0.907671622 -7.625773455 cg21864378 6
29719180 p MOG IFITM4P NA TSS1500 shore
TSS1500 - shore NA 0.894842473 0.897631124
0.002788651 0.996893322
-0.02642229 0.560986585 -0.459172524 0.649137841
0.907671622 -7.626403997 cg13835168 6
29648756 p GABBR1/MOG ZFP57 8587 IGR open
sea IGR - open sea NA 0.57059469 0.544172401 -
0.02642229 1.048554996
0.003158821 0.728484082 0.45869498 0.649477206
0.907671622 -7.626627974 cg17728820 6
29709366 p MOG LOC285830 NA Body open
sea Body - open sea NA 0.72733542 0.730494241
0.003158821 0.995675776
0.004320828 0.289444254 0.458085535 0.649910416
0.907671622 -7.62691348 cg13666340 9 4666266
p SLC1A1 C9orf68 NA 5'UTR shelf 5'UTR -
shelf NA 0.287873044 0.292193872 0.004320828
0.985212462

0.001959035	0.911926323	0.456220763	0.651236714
0.907671622	-7.627784739	cg05157371 6
29588894	p	GABBR1/MOG GABBR1	NA	Body	open
sea	Body - open sea	NA	0.911213946	0.913172981
0.001959035	0.997854695
-0.008814678	0.739757215	-0.454829833	0.65222675
0.907671622	-7.628432325	cg04063345 6
152130058	q	ESR1	ESR1	NA	Body	shore
Body - shore	NA	0.742962552	0.734147874
-0.008814678	1.012006679
0.002955151	0.892639589	0.454646038	0.65235762
0.907671622	-7.62851775	cg01078434 6
29455532	p	GABBR1	MAS1L	NA	1stExon	open
sea	1stExon - open sea	NA	0.891564988	0.894520139
0.002955151	0.996696384
0.001516673	0.936664415	0.453594838	0.653106333
0.907671622	-7.629005675	cg05309760 6
152501942	q	ESR1	SYNE1	NA	Body	open
sea	Body - open sea	NA	0.936112898	0.937629571
0.001516673	0.998382439
-0.002589371	0.10959495 -0.452359329	0.653986786
0.907671622	-7.629577723	cg21950534 6
152128483	q	ESR1	ESR1	NA	TSS1500	shore
TSS1500 - shore	NA	0.110536539	0.107947168
-0.002589371	1.023987392
0.005042391	0.681017436	0.452269479	0.654050835
0.907671622	-7.629619264	cg10601943 6
29692824	p	GABBR1/MOG HLA-F	NA	Body	shore
Body - shore	NA	0.679183839	0.68422623
0.005042391	0.992630521
-0.002705189	0.853903357	-0.451943532	0.654283207
0.907671622	-7.629769893	cg13537510 6
29623721	p	GABBR1/MOG MOG	NA	TSS1500	open
sea	TSS1500 - open sea	NA	0.854887062	0.852181873
-0.002705189	1.003174427
0.001321385	0.038218942	0.450916758	0.655015435
0.907671622	-7.630243692	cg18699287 6
29721001	p	MOG	IFITM4P	2417	IGR	island
IGR - island	NA	0.037738439	0.039059824
0.001321385	0.966170226
-0.009187607	0.385521983	-0.450342856	0.655424856
0.907671622	-7.630508052	cg04522432 6
29618347	p	GABBR1/MOG MOG	-6411	IGR	shore
IGR - shore	NA	0.38886293 0.379675324	-
0.009187607	1.024198586
-0.002508839	0.870413756	-0.448537199	0.656713716
0.907671622	-7.631337634	cg25000210 6
29455302	p	GABBR1	MAS1L	NA	1stExon	open
sea	1stExon - open sea	NA	0.871326061	0.868817222
-0.002508839	1.002887649
0.002090534	0.919110476	0.447102251	0.65773873
0.907671622	-7.631994551	cg01373089 6
29589631	p	GABBR1/MOG GABBR1	NA	Body	open
sea	Body - open sea	NA	0.918350282	0.920440816
0.002090534	0.997728769
-0.001709325	0.919001354	-0.446604895	0.658094159
0.907671622	-7.632221755	cg12111808 6

29588168 p GABBR1/MOG GABBR1 NA Body open
sea Body - open sea NA 0.919622927 0.917913602
-0.001709325 1.001862185
0.001071816 0.050160451 0.44578545 0.658679942
0.907671622 -7.632595552 cg17290446 12
72233346 q TPH2 TBC1D15 NA TSS200 island
TSS200 - island NA 0.0497707 0.050842516
0.001071816 0.978918903
0.013522575 0.582306706 0.444985896 0.659251717
0.907671622 -7.632959621 cg05813221 6
29629988 p GABBR1/MOG MOG NA Body open
sea Body - open sea NA 0.577389406 0.590911981
0.013522575 0.977115754
0.002086142 0.912290621 0.444341071 0.659712994
0.907671622 -7.633252766 cg14656245 6
29627290 p GABBR1/MOG MOG NA Body open
sea Body - open sea NA 0.911532024 0.913618166
0.002086142 0.997716615
0.002854062 0.204451443 0.444245432 0.659781422
0.907671622 -7.633296209 cg05012697 15
88798331 q NTRK3 NTRK3 NA Body shore
Body - shore NA 0.203413602 0.206267664
0.002854062 0.986163309
0.002000285 0.911189614 0.441854212 0.661493243
0.907671622 -7.634379386 cg16128363 18 3880558
p DLGAP1 DLGAP1 NA TSS1500 shore TSS1500
- shore NA 0.910462237 0.912462523 0.002000285
0.997807816
0.001287232 0.033971504 0.441104833 0.662030088
0.907671622 -7.634717651 cg08541345 6
29716319 p MOG LOC285830 NA Body shore
Body - shore NA 0.033503419 0.034790651
0.001287232 0.963000635
0.001626881 0.101342077 0.438882373 0.663623299
0.907671622 -7.635717521 cg27230724 2
172751074 q SLC25A12 SLC25A12 NA TSS1500 shore
TSS1500 - shore NA 0.100750484 0.102377365
0.001626881 0.984108978
0.002076871 0.933405173 0.438440831 0.663940016
0.907671622 -7.635915574 cg18679753 6
29443446 p GABBR1 MAS1L -11097 IGR open
sea IGR - open sea NA 0.932649948 0.934726819
0.002076871 0.997778098
-0.00133461 0.059826436 -0.438396823 0.663971586
0.907671622 -7.635935302 cg24772753 2
171573419 q GAD1 SP5 NA Body island
Body - island NA 0.060311748 0.058977138
-0.00133461 1.022629277
0.001374253 0.040450359 0.437510981 0.664607201
0.907671622 -7.636332011 ch.15.1497565F 15
88581122 q NTRK3 NTRK3 NA Body open
sea Body - open sea NA 0.039950631 0.041324884
0.001374253 0.966745146
0.001470523 0.082182622 0.436061898 0.6656475
0.907671622 -7.636979247 cg03789152 12
72233372 q TPH2 TBC1D15 NA TSS200 island

TSS200 - island NA 0.081647886 0.083118409
0.001470523 0.982308095
0.00230389 0.875347996 0.436023265 0.665675243
0.907671622 -7.636996473 cg00224929 2
171705463 q GAD1 GAD1 NA Body open
sea Body - open sea NA 0.874510218 0.876814108
0.00230389 0.997372431
-0.001800363 0.044902627 -0.436014085 0.665681836
0.907671622 -7.637000566 cg24065044 11
27723409 p BDNF BDNF NA TSS1500 shore
TSS1500 - shore NA 0.045557305 0.043756942
-0.001800363 1.041144626
-0.004596821 0.347025447 -0.435353008 0.666156658
0.907671622 -7.637295101 cg14486905 2
171677602 q GAD1 GAD1 NA Body shore
Body - shore NA 0.348697018 0.344100197
-0.004596821 1.013358961
0.001387458 0.057212129 0.427057053 0.672127121
0.914895424 -7.640953693 cg05737526 18 3454175
p DLGAP1 TGIF1 NA 5'UTR shore 5'UTR -
shore NA 0.056707599 0.058095057 0.001387458
0.976117452
0.002294465 0.887269341 0.424949706 0.673647217
0.916053074 -7.641871966 cg24640415 6
29574953 p GABBR1/MOG GABBR1 NA Body open
sea Body - open sea V$ZID_01 0.88643499 0.888729455
0.002294465 0.997418264
-0.004388144 0.570915289 -0.422914748 0.67511642
0.916951352 -7.642754432 cg05812266 6
29595194 p GABBR1/MOG GABBR1 NA Body shore
Body - shore NA 0.572510977 0.568122833
-0.004388144 1.007723935
0.003514472 0.830863587 0.422178407 0.675648365
0.916951352 -7.643072716 cg00521620 6
29434416 p GABBR1 OR2H1 8186 IGR open
sea IGR - open sea NA 0.829585597 0.833100069
0.003514472 0.995781453
0.002212975 0.101849072 0.416805313 0.679535093
0.917911659 -7.645378631 ch.2.3495108F 2
172804051 q SLC25A12 HAT1 NA Body open
sea Body - open sea NA 0.101044354 0.103257329
0.002212975 0.978568349
0.002913713 0.843498592 0.415648241 0.680373255
0.917911659 -7.645871376 cg26371172 6
29454888 p GABBR1 MAS1L NA 1stExon open
sea 1stExon - open sea NA 0.84243906 0.845352772
0.002913713 0.996553259
0.000782017 0.046663129 0.415450907 0.680516241
0.917911659 -7.645955276 cg08967211 6
29596174 p GABBR1/MOG GABBR1 NA Body shore
Body - shore NA 0.046378759 0.047160777
0.000782017 0.983418042
0.001505587 0.943443932 0.415046178 0.680809541
0.917911659 -7.646127231 cg25197880 6
29695413 p GABBR1/MOG LOC285830 NA Body shelf
Body - shelf NA 0.942896445 0.944402033
0.001505587 0.998405776

-0.001004781 0.048534753 -0.41434873 0.681315089
0.917911659 -7.646423162 cg23727007 6
29716796 p MOG LOC285830 NA Body island
Body - island NA 0.048900128 0.047895347
-0.001004781 1.020978677
-0.002491039 0.902016745 -0.414304015 0.681347506
0.917911659 -7.646442118 cg11358777 6
29638498 p GABBR1/MOG MOG NA 3'UTR open
sea 3'UTR - open sea NA 0.902922577 0.900431538
-0.002491039 1.002766495
0.001666447 0.073151941 0.413752133 0.681747654
0.917911659 -7.646675911 cg19411146 6
152128471 q ESR1 ESR1 NA TSS1500 shore
TSS1500 - shore NA 0.07254596 0.074212408
0.001666447 0.977544887
-0.004269127 0.111903372 -0.411905159 0.683087501
0.917911659 -7.647456099 cg13974632 11
27740813 p BDNF BDNF NA Body island
Body - island NA 0.113455782 0.109186654
-0.004269127 1.039099357
-0.002337576 0.258218473 -0.411608097 0.683303096
0.917911659 -7.64758126 cg17053201 6
29593246 p GABBR1/MOG GABBR1 NA Body shelf
Body - shelf NA 0.259068501 0.256730925
-0.002337576 1.009105159
-0.002549244 0.694397499 -0.410903697 0.683814427
0.917911659 -7.647877687 cg15641340 6
29429909 p GABBR1 OR2H1 NA Body open
sea Body - open sea V$PPARG_01;V$PPARA_01 0.695324497
0.692775252 -0.002549244 1.003679758
-0.005937074 0.357967338 -0.410325592 0.684234193
0.917911659 -7.64812059 cg27204993 9 4662937
p SLC1A1 C9orf68 NA Body island Body -
island NA 0.360126274 0.354189201 -0.005937074
1.016762434
0.001681932 0.920451606 0.408858303 0.68530006
0.917911659 -7.648735584 cg12322235 6
29549724 p GABBR1/MOG SNORD32B NA TSS1500 open
sea TSS1500 - open sea NA 0.919839994 0.921521926
0.001681932 0.998174832
-0.003179494 0.844197733 -0.408326814 0.685686306
0.917911659 -7.648957812 cg23174322 6
29627215 p GABBR1/MOG MOG NA Body open
sea Body - open sea NA 0.845353913 0.842174419
-0.003179494 1.003775339
-0.000826367 0.044858905 -0.407703666 0.686139271
0.917911659 -7.649218001 cg23105568 6
29617607 p GABBR1/MOG MOG -7151 IGR shore
IGR - shore NA 0.045159402 0.044333036
-0.000826367 1.01863996
-0.001809704 0.929507225 -0.407316474 0.68642078
0.917911659 -7.64937947 cg05421799 2
172778343 q SLC25A12 HAT1 NA TSS1500 shore
TSS1500 - shore NA 0.9301653 0.928355595 -
0.001809704 1.001949366
0.000776439 0.054017472 0.406157257 0.687263864
0.918141568 -7.649861987 cg09492354 11

27720709 p BDNF BDNF NA Body shore
Body - shore NA 0.053735131 0.05451157
0.000776439 0.985756437
0.000823561 0.033529174 0.40251499 0.689915483
0.920784762 -7.6513692 cg11582100 2 171673207 q
GAD1 GAD1 NA 5'UTR island 5'UTR - island
NA 0.033229697 0.034053259 0.000823561
0.975815472
-0.002356131 0.872572469 -0.400253971 0.691563544
0.922084726 -7.652298073 cg15154411 6
29576987 p GABBR1/MOG GABBR1 NA Body open
sea Body - open sea NA 0.873429243 0.871073113
-0.002356131 1.002704859
0.00489253 0.257215028 0.39821727 0.69304941 0.923166107 -
7.653130355 cg04188862 15 88801474 q NTRK3 NTRK3-
AS1 5513 IGR shore IGR - shore NA
0.255435926 0.260328457 0.00489253 0.981206315
0.000633564 0.013194902 0.396759287 0.694113834
0.923684557 -7.653723567 cg04852989 22
19974685 q COMT ARVCF NA Body island
Body - island NA 0.012964515 0.013598079
0.000633564 0.953407831
0.000571999 0.017071513 0.395626208 0.694941493
0.923887232 -7.654183095 cg16956031 18 3451545
p DLGAP1 TGIF1 NA 5'UTR island 5'UTR -
island NA 0.016863513 0.017435512 0.000571999
0.96719345
-0.005267325 0.612689459 -0.393222258 0.69669872
0.924676275 -7.655153725 cg06346307 22
19949965 q COMT COMT NA 5'UTR open
sea 5'UTR - open sea NA 0.61460485 0.609337525 -
0.005267325 1.008644347
0.00869065 0.645262257 0.392964999 0.696886871
0.924676275 -7.65525725 cg08739755 2
172815385 q SLC25A12 HAT1 NA Body open
sea Body - open sea NA 0.642102021 0.650792671
0.00869065 0.986646054
-0.002595795 0.7792066 -0.389104717 0.699712495
0.927525865 -7.656802626 cg13927803 6
29524042 p GABBR1 UBD NA Body shelf
Body - shelf NA 0.780150525 0.77755473 -
0.002595795 1.003338408
0.002832047 0.809274706 0.387238156 0.701080333
0.928197727 -7.65754444 cg27241909 6
29714043 p MOG LOC285830 NA Body shelf
Body - shelf NA 0.808244871 0.811076917
0.002832047 0.996508289
-0.002847974 0.891092422 -0.386561627 0.701576352
0.928197727 -7.657812436 cg18078658 12
72374129 q TPH2 TPH2 NA Body open
sea Body - open sea NA 0.892128049 0.889280075
-0.002847974 1.003202561
-0.001093369 0.027444647 -0.382414442 0.704619896
0.930580243 -7.659445123 cg14514600 9 4666477
p SLC1A1 C9orf68 NA 1stExon shelf 1stExon
- shelf V$RFX1_02 0.027842236 0.026748866 -0.001093369
1.040875378

0.001157804 0.057233242 0.382176619 0.704794581
0.930580243 -7.659538221 cg01583365 2
172778734 q SLC25A12 HAT1 NA TSS200 island
TSS200 - island NA 0.056812222 0.057970026
0.001157804 0.98002754
0.001227242 0.070854116 0.381328163 0.705417918
0.930580243 -7.659869891 cg14276488 9 4491114
p SLC1A1 SLC1A1 NA Body shore Body -
shore NA 0.070407846 0.071635088 0.001227242
0.982868144
0.002123354 0.07198004 0.380357497 0.706131292
0.9306239 -7.660248437 cg26158180 15 88798666 q
NTRK3 NTRK3 NA Body shore Body - shore
NA 0.071207911 0.073331265 0.002123354
0.971044356
0.002651078 0.899844392 0.378340079 0.707614824
0.931220176 -7.661032143 cg06266853 6
29589707 p GABBR1/MOG GABBR1 NA Body open
sea Body - open sea NA 0.898880363 0.901531441
0.002651078 0.997059362
0.002205266 0.882956539 0.376692805 0.708827028
0.931220176 -7.661668995 cg21645752 6
29598695 p GABBR1/MOG GABBR1 NA Body shore
Body - shore NA 0.882154625 0.88435989
0.002205266 0.997506372
-0.005506414 0.524224114 -0.375911267 0.709402421
0.931220176 -7.661970182 cg02824029 6
29570008 p GABBR1/MOG GABBR1 NA 3'UTR open
sea 3'UTR - open sea V$TCF11MAFG_01;V$BACH1_01;V$NFE2_01;V$AP1_01
0.526226446 0.520720032 -0.005506414
1.010574615
0.004849476 0.664768601 0.375289093 0.709860608
0.931220176 -7.66220951 cg09083279 6
29454873 p GABBR1 MAS1L NA 1stExon open
sea 1stExon - open sea NA 0.663005155 0.667854631
0.004849476 0.992738725
-0.002323972 0.902466934 -0.375117068 0.709987312
0.931220176 -7.662275613 cg06787004 22
19938981 q COMT COMT NA 5'UTR open
sea 5'UTR - open sea NA 0.903312015 0.900988043
-0.002323972 1.002579359
-0.001066057 0.057788202 -0.372366951 0.712014028
0.932782799 -7.663328295 cg04627496 15
88799973 q NTRK3 NTRK3 NA TSS1500 island
TSS1500 - island NA 0.05817586 0.057109802 -
0.001066057 1.018666813
0.008512121 0.684302733 0.370995948 0.713025193
0.932782799 -7.663850215 cg13763482 9 4663107
p SLC1A1 C9orf68 NA Body shore Body -
shore NA 0.681207417 0.689719538 0.008512121
0.987658576
0.001378934 0.083490189 0.369983085 0.713772557
0.932782799 -7.66423457 cg21133153 6
29521488 p GABBR1 UBD -1901 IGR island
IGR - island NA 0.082988758 0.084367692
0.001378934 0.983655663

0.001224681 0.060773782 0.368851019 0.714608217
0.932782799 -7.664662927 cg16535180 22
20008367 q COMT C22orf25 NA TSS1500 island
TSS1500 - island NA 0.060328444 0.061553125
0.001224681 0.980103675
-0.002098572 0.895323331 -0.365899742 0.716788451
0.932782799 -7.665773525 cg18126978 6
29588988 p GABBR1/MOG GABBR1 NA Body open
sea Body - open sea NA 0.896086448 0.893987876
-0.002098572 1.002347428
-0.001918456 0.109655798 -0.365755535 0.716895044
0.932782799 -7.665827564 cg03793625 2
171669862 q GAD1 GAD1 -3338 IGR shore
IGR - shore NA 0.110353418 0.108434962
-0.001918456 1.017692227
0.004920274 0.396211429 0.363695041 0.718418735
0.932782799 -7.666597403 cg16289618 6
29705939 p MOG LOC285830 NA Body open
sea Body - open sea NA 0.394422238 0.399342513
0.004920274 0.98767906
0.001122737 0.083329922 0.363641675 0.718458214
0.932782799 -7.666617284 cg03770593 2
172750719 q SLC25A12 SLC25A12 NA 1stExon island
1stExon - island V$ER_Q6 0.082921654 0.084044391
0.001122737 0.986641143
0.001580083 0.928224238 0.363260232 0.718740417
0.932782799 -7.666759304 cg13427473 15
88406836 q NTRK3 NTRK3-AS1 -13152 IGR open
sea IGR - open sea NA 0.927649662 0.929229745
0.001580083 0.998299578
0.00080635 0.029393012 0.362085399 0.719609846
0.932782799 -7.667195793 cg03471611 17
28431762 q SLC6A4 EFCAB5 NA Body open
sea Body - open sea NA 0.029099793 0.029906143
0.00080635 0.973037312
0.002643283 0.872506568 0.36176209 0.719849177
0.932782799 -7.667315667 cg11806762 11
27732958 p BDNF BDNF NA Body open
sea Body - open sea NA 0.871545374 0.874188657
0.002643283 0.996976301
-0.001828345 0.894331165 -0.361456583 0.720075355
0.932782799 -7.667428842 cg10110652 6
29589927 p GABBR1/MOG GABBR1 NA Body open
sea Body - open sea NA 0.894996017 0.893167672
-0.001828345 1.002047034
-0.003686178 0.729116103 -0.360573642 0.720729174
0.932782799 -7.667755396 cg26646411 22
19841374 q COMT GNB1L NA 5'UTR shore
5'UTR - shore NA 0.730456531 0.726770353
-0.003686178 1.005071998
-0.002204723 0.104051911 -0.360503154 0.720781379
0.932782799 -7.667781432 cg27546977 6
29521152 p GABBR1 UBD -2237 IGR island
IGR - island NA 0.104853629 0.102648905
-0.002204723 1.0214783
0.006497551 0.352316487 0.358990867 0.721901755
0.932782799 -7.668338799 cg12749246 6

29521013	p	GABBR1	UBD	-2376	IGR	shore
IGR - shore	NA	0.349953742	0.356451292
0.006497551	0.981771563
0.006671552	0.61082341	0.358738962	0.722088438
0.932782799	-7.668431415	cg14713146	2
171782647	q	GAD1	GORASP2	-2301	IGR	shelf
IGR - shelf	NA	0.608397391	0.615068943
0.006671552	0.989153164
-0.003353345	0.597573312	-0.357775474	0.722802629
0.932824525	-7.668785058	cg11588423	6
29578191	p	GABBR1/MOG	GABBR1	NA	Body	open
sea	Body - open sea	NA	0.59879271	0.595439366	-
0.003353345	1.005631714
-0.001878569	0.16934782	-0.356310176	0.723889273
0.933346395	-7.669321079	cg07196069	9	4491175
p	SLC1A1	SLC1A1	NA	Body	island	Body -
island	NA	0.170030936	0.168152367	-0.001878569
1.011171826
0.0010164	0.934720792	0.355052249	0.724822599
0.933669789	-7.669779501	cg15370577	6
29580340	p	GABBR1/MOG	GABBR1	NA	Body	open
sea	Body - open sea	NA	0.934351192	0.935367592
0.0010164	0.998913368
0.000665057	0.01917712	0.34969167	0.728804691	0.936648677
-7.671714996	cg05101432	6	29720737	p	MOG
IFITM4P	2153	IGR	island	IGR - island	NA
0.018935281	0.019600338	0.000665057
0.966069106
0.002492692	0.91057341	0.349439972	0.728991853
0.936648677	-7.671805156	cg06643882	18	3773774
p	DLGAP1	DLGAP1	NA	Body	shelf	Body -
shelf	NA	0.909666976	0.912159668	0.002492692
0.997267264
-0.002544665	0.854807174	-0.34865693	0.729574231
0.936648677	-7.672085236	cg00237606	6
29455256	p	GABBR1	MAS1L	NA	1stExon	open
sea	1stExon - open sea	NA	0.855732507	0.853187842
-0.002544665	1.002982538
0.001047537	0.026683868	0.34704682	0.730772243
0.936648677	-7.672659182	cg04661674	6
29596874	p	GABBR1/MOG	GABBR1	NA	TSS1500	shore
TSS1500 - shore	V$BRACH_01	0.026302946	0.027350482
0.001047537	0.961699542
0.002966419	0.571689985	0.346306411	0.73132338
0.936648677	-7.672922225	cg26129669	6
29593913	p	GABBR1/MOG	GABBR1	NA	Body	shore
Body - shore	NA	0.570611287	0.573577706
0.002966419	0.994828218
0.001641011	0.817466161	0.345948461	0.731589878
0.936648677	-7.673049194	cg17546147	6
29710109	p	MOG	LOC285830	NA	Body	open
sea	Body - open sea	NA	0.81686943	0.818510441
0.001641011	0.997995125
-0.001832214	0.182436059	-0.345494442	0.73192795
0.936648677	-7.673210051	cg24249411	11
27744759	p	BDNF	BDNF	NA	TSS1500	shore

TSS1500 - shore NA 0.183102319 0.181270105
-0.001832214 1.010107646
-0.002318312 0.706624662 -0.342441214 0.734202858
0.938681784 -7.674286352 cg17987649 6
29526534 p GABBR1/MOG UBD NA Body open
sea Body - open sea NA 0.707467685 0.705149373
-0.002318312 1.003287689
0.000851327 0.025159864 0.340661665 0.735529898
0.939445235 -7.67490929 cg09742688 2
171672899 q GAD1 GAD1 NA TSS1500 island
TSS1500 - island NA 0.024850291 0.025701617
0.000851327 0.966876559
-0.002089623 0.884149486 -0.339579221 0.736337499
0.939445235 -7.675286626 cg04957663 6
29587487 p GABBR1/MOG GABBR1 NA Body open
sea Body - open sea NA 0.884909349 0.882819726
-0.002089623 1.002366987
0.002823992 0.071998089 0.338878868 0.736860188
0.939445235 -7.675530132 cg15899474 2
172779515 q SLC25A12 HAT1 NA Body shore
Body - shore NA 0.070971183 0.073795175
0.002823992 0.961732024
0.00323155 0.093014793 0.336930005 0.738315338
0.939536733 -7.676205105 cg16164802 6
29691808 p GABBR1/MOG HLA-F NA Body island
Body - island NA 0.091839684 0.095071234
0.00323155 0.966009172
0.002917818 0.902047477 0.335182415 0.73962104
0.939536733 -7.676807081 cg25329933 6
29636669 p GABBR1/MOG MOG NA 3'UTR open
sea 3'UTR - open sea NA 0.900986452 0.90390427
0.002917818 0.996771983
0.001345797 0.943476092 0.334053949 0.740464584
0.939536733 -7.677194142 cg26598649 18 3880086
p DLGAP1 DLGAP1 NA 1stExon island 1stExon
- island NA 0.942986712 0.944332509 0.001345797
0.99857487
-0.001776744 0.772747326 -0.333026934 0.741232576
0.939536733 -7.677545277 cg22319611 22
19840851 q COMT GNB1L NA 5'UTR shore
5'UTR - shore NA 0.773393415 0.77161667 -
0.001776744 1.002302627
0.001087538 0.923318619 0.332649815 0.74151465
0.939536733 -7.677673945 cg24628013 6
29580367 p GABBR1/MOG GABBR1 NA Body open
sea Body - open sea NA 0.922923151 0.924010689
0.001087538 0.998823024
0.001247349 0.048364956 0.332369337 0.741724462
0.939536733 -7.677769546 cg08870743 21
34398199 q OLIG2 OLIG2 NA TSS200 island
TSS200 - island NA 0.047911375 0.049158724
0.001247349 0.974626091
-0.001955506 0.877571243 -0.330737453 0.742945599
0.939536733 -7.678324185 cg08730070 22
19938378 q COMT COMT NA 5'UTR open
sea 5'UTR - open sea NA 0.878282336 0.87632683 -
0.001955506 1.00223148

-0.000608526 0.030152888 -0.330306178 0.743268434
0.939536733 -7.678470312 cg04207032 22
20004746 q COMT ARVCF NA TSS1500 shore
TSS1500 - shore NA 0.03037417 0.029765644 -
0.000608526 1.020443905
-0.004855114 0.722721113 -0.329840806 0.743616846
0.939536733 -7.678627779 cg22679406 6
29599331 p GABBR1/MOG GABBR1 NA Body shore
Body - shore NA 0.72448661 0.719631495 -
0.004855114 1.006746668
0.001342005 0.106464629 0.329596313 0.743799914
0.939536733 -7.67871042 cg12899157 18 3452302
p DLGAP1 TGIF1 NA 5'UTR island 5'UTR -
island NA 0.105976627 0.107318632 0.001342005
0.987495135
0.003285571 0.350456313 0.323970898 0.748016191
0.943990913 -7.680595039 cg13524919 21
34396506 q OLIG2 OLIG2 -1710 IGR island
IGR - island NA 0.34926156 0.35254713 0.003285571
0.99068048
-0.002936547 0.15003723 -0.322347452 0.749234447
0.944656887 -7.68113293 cg10364942 21
34401201 q OLIG2 OLIG2 NA 3'UTR shore
3'UTR - shore NA 0.151105065 0.148168518
-0.002936547 1.019818967
0.000876709 0.053229666 0.319549597 0.75133553
0.946433706 -7.682053633 cg18877200 6
29720927 p MOG IFITM4P 2343 IGR island
IGR - island NA 0.052910862 0.053787572
0.000876709 0.98370051
-0.000590252 0.016883128 -0.317691395 0.752732036
0.946867336 -7.682660712 cg24899205 22
19929184 q COMT TXNRD2 NA Body island
Body - island NA 0.017097765 0.016507513
-0.000590252 1.035756567
-0.002387373 0.890509138 -0.316086305 0.753939003
0.946867336 -7.683182267 cg27318000 18 3845667
p DLGAP1 DLGAP1 NA TSS1500 open sea TSS1500
- open sea NA 0.891377274 0.888989901 -0.002387373
1.002685489
-0.00189759 0.103334152 -0.315651032 0.75426642
0.946867336 -7.683323251 cg01306985 6
29720340 p MOG IFITM4P NA TSS1500 shore
TSS1500 - shore NA 0.104024184 0.102126595
-0.00189759 1.018580753
0.002013537 0.87644386 0.315409147 0.754448389
0.946867336 -7.683401513 cg05358170 6
29692399 p GABBR1/MOG HLA-F NA Body shore
Body - shore NA 0.875711665 0.877725201
0.002013537 0.997705961
0.00115017 0.066054755 0.314287845 0.755292124
0.947057401 -7.683763535 cg26852170 6
29720448 p MOG IFITM4P 1864 IGR island
IGR - island NA 0.065636512 0.066786682
0.00115017 0.982778453
-0.005251358 0.366952105 -0.313134364 0.75616039
0.947276224 -7.684134608 cg09641376 6

29520965 p GABBR1 UBD -2424 IGR shore
IGR - shore NA 0.36886169 0.363610332 -
0.005251358 1.014442268
0.003137198 0.777509813 0.311046561 0.757732774
0.947276224 -7.684802803 cg01176329 6
29592381 p GABBR1/MOG GABBR1 NA Body shelf
Body - shelf NA 0.776369014 0.779506212
0.003137198 0.995975403
0.001411298 0.930951004 0.309317016 0.759036138
0.947276224 -7.685352973 cg01418645 11
27679469 p BDNF BDNF NA Body open
sea Body - open sea NA 0.930437805 0.931849103
0.001411298 0.998485487
-0.001333784 0.16814164 -0.30823149 0.759854544
0.947276224 -7.685696722 cg18264657 18 4455862
p DLGAP1 DLGAP1-AS5 191260 IGR shore IGR -
shore NA 0.168626653 0.167292868 -0.001333784
1.007972755
0.001533768 0.087999823 0.307827288 0.760159354
0.947276224 -7.685824412 cg03565659 12
72332624 q TPH2 TPH2 NA TSS200 open
sea TSS200 - open sea NA 0.087442089 0.088975857
0.001533768 0.982761976
0.002656622 0.847089581 0.307770739 0.760202001
0.947276224 -7.685842263 cg02139827 21
34404457 q OLIG2 OLIG2 6241 IGR shore
IGR - shore NA 0.846123536 0.848780158
0.002656622 0.996870071
0.004843044 0.280349359 0.306741908 0.760978036
0.947276224 -7.686166466 cg17741339 6
152085619 q ESR1 ESR1 NA 5'UTR open
sea 5'UTR - open sea NA 0.278588253 0.283431296
0.004843044 0.982912815
0.000934607 0.07922742 0.306401904 0.761234552
0.947276224 -7.68627337 cg21434114 18 3450282
p DLGAP1 TGIF1 NA 5'UTR island 5'UTR -
island NA 0.078887563 0.079822169 0.000934607
0.988291398
0.00272187 0.763657451 0.305731657 0.7617403 0.947276224
-7.686483764 cg07821417 22 19972146 q COMT
ARVCF NA Body shelf Body - shelf NA
0.76266768 0.765389551 0.00272187 0.99644381
0.003545467 0.375571573 0.304220905 0.76288066
0.947276224 -7.686956317 cg04106006 11
27742454 p BDNF BDNF NA Body shore
Body - shore NA 0.374282312 0.377827779
0.003545467 0.990616182
-0.003359405 0.141876842 -0.303555687 0.763382956
0.947276224 -7.687163655 cg17413943 11
27739827 p BDNF BDNF NA Body shore
Body - shore NA 0.143098444 0.139739039
-0.003359405 1.024040562
0.000673771 0.038989741 0.303035151 0.763776078
0.947276224 -7.687325582 cg03434929 6
29617848 p GABBR1/MOG MOG -6910 IGR island
IGR - island NA 0.038744733 0.039418504
0.000673771 0.982907241

0.003197951 0.328825507 0.300336834 0.765814936
0.948745179 -7.688160542 cg18704527 22
19962042 q COMT ARVCF NA Body shore
Body - shore NA 0.327662616 0.330860567
0.003197951 0.990334445
0.001560383 0.09228693 0.299632344 0.766347531
0.948745179 -7.688377315 cg00465975 22
19929557 q COMT TXNRD2 NA TSS200 shore
TSS200 - shore NA 0.091719518 0.093279901
0.001560383 0.983272034
-0.001025114 0.046643888 -0.297176375 0.768205148
0.949163319 -7.689129062 cg04648747 12
72233970 q TPH2 TBC1D15 NA Body shore
Body - shore NA 0.047016657 0.045991543
-0.001025114 1.022289185
0.000836836 0.046585046 0.296985773 0.768349371
0.949163319 -7.689187146 cg00476814 22
19974536 q COMT ARVCF NA Body island
Body - island NA 0.046280742 0.047117578
0.000836836 0.98223941
-0.009839482 0.3490033 -0.296434198 0.768766782
0.949163319 -7.689355024 cg25439807 18 3771151
p DLGAP1 DLGAP1 NA Body shore Body -
shore NA 0.352581293 0.342741811 -0.009839482
1.028708146
0.001510757 0.069218536 0.293444584 0.771030421
0.949947872 -7.690259544 cg01777019 6
152128805 q ESR1 ESR1 NA 5'UTR shore
5'UTR - shore NA 0.06866917 0.070179927
0.001510757 0.97847309
-0.001261557 0.065678953 -0.292391003 0.771828646
0.949947872 -7.690576137 cg02285263 6
152129749 q ESR1 ESR1 NA Body island
Body - island NA 0.066137701 0.064876144
-0.001261557 1.019445622
-0.002283953 0.138508991 -0.291745961 0.772317474
0.949947872 -7.690769407 cg00729049 2
171679402 q GAD1 GAD1 NA Body island
Body - island NA 0.13933952 0.137055566 -
0.002283953 1.016664438
0.000618984 0.021354944 0.289547225 0.773984438
0.949947872 -7.691425012 cg14520770 22
19842507 q COMT GNB1L NA TSS200 island
TSS200 - island NA 0.021129859 0.021748842
0.000618984 0.971539496
0.000609751 0.027234727 0.288922638 0.774458167
0.949947872 -7.691610348 cg18094551 22
20004364 q COMT ARVCF NA TSS200 island
TSS200 - island NA 0.027013 0.027622751
0.000609751 0.977925769
0.00170306 0.859043822 0.288847777 0.774514951
0.949947872 -7.691632534 cg09603409 6
29587016 p GABBR1/MOG GABBR1 NA Body open
sea Body - open sea NA 0.858424528 0.860127587
0.00170306 0.998019993
-0.001304584 0.049892231 -0.287865011 0.77526054
0.949947872 -7.691923272 cg06581978 21

34408891 q OLIG2 OLIG2 10675 IGR shelf
IGR - shelf NA 0.050366626 0.049062042
-0.001304584 1.026590495
0.001918215 0.861805465 0.286668667 0.776168455
0.949947872 -7.692275863 cg02274336 6
29427807 p GABBR1 OR2H1 NA 5'UTR open
sea 5'UTR - open sea NA 0.861107932 0.863026147
0.001918215 0.997777338
0.000830106 0.139640341 0.285697432 0.776905769
0.949947872 -7.692561035 cg18095109 6
29720427 p MOG IFITM4P 1843 IGR island
IGR - island NA 0.139338484 0.14016859
0.000830106 0.994077803
-0.002784662 0.334453124 -0.283701791 0.77842142
0.949947872 -7.693143968 cg24351901 6
29692092 p GABBR1/MOG HLA-F NA Body island
Body - island NA 0.335465729 0.332681066
-0.002784662 1.008370368
-0.014683726 0.56593291 -0.283108734 0.778872006
0.949947872 -7.693316418 cg03198009 6
29648604 p GABBR1/MOG ZFP57 8435 IGR open
sea IGR - open sea NA 0.571272447 0.556588721
-0.014683726 1.026381645
-0.003837953 0.81776238 -0.282480897 0.779349102
0.949947872 -7.69349859 cg01066157 6
152451023 q ESR1 SYNE1 NA Body open
sea Body - open sea NA 0.819157999 0.815320046
-0.003837953 1.004707296
-0.002350445 0.0709719 -0.282296419 0.779489303
0.949947872 -7.693552041 cg01296653 9 4678949
p SLC1A1 CDC37L1 NA TSS1500 shore TSS1500
- shore NA 0.071826607 0.069476162 -0.002350445
1.033830956
0.002161515 0.787879369 0.282063726 0.779666159
0.949947872 -7.693619413 cg25191596 6
29455126 p GABBR1 MAS1L NA 1stExon open
sea 1stExon - open sea NA 0.787093363 0.789254879
0.002161515 0.997261321
0.002463035 0.847977306 0.281863539 0.779818319
0.949947872 -7.693677329 cg09902251 15
88590236 q NTRK3 NTRK3 NA Body open
sea Body - open sea NA 0.847081657 0.849544692
0.002463035 0.997100759
0.002001077 0.820318412 0.28024472 0.781049085
0.950600666 -7.694144165 cg04765420 6
29698866 p MOG LOC285830 NA Body open
sea Body - open sea NA 0.819590748 0.821591824
0.002001077 0.997564392
-0.000845174 0.15942056 -0.278476893 0.782393796
0.951219551 -7.694650916 cg21836827 6
29720549 p MOG IFITM4P 1965 IGR island
IGR - island NA 0.159727896 0.158882722
-0.000845174 1.005319483
0.001998599 0.074518574 0.276795995 0.783673014
0.951219551 -7.695129788 cg05054006 17
28443555 q SLC6A4 CCDC55 NA TSS1500 shore

TSS1500 - shore NA 0.07379181 0.075790409
0.001998599 0.973629922
0.003262185 0.823612935 0.276366107 0.784000272
0.951219551 -7.695251796 cg08451469 6
29711588 p MOG LOC285830 NA Body open
sea Body - open sea NA 0.822426686 0.825688871
0.003262185 0.996049135
0.000674992 0.032675359 0.275921301 0.784338928
0.951219551 -7.695377839 cg17625506 6
29691726 p GABBR1/MOG HLA-F NA Body island
Body - island NA 0.032429907 0.033104899
0.000674992 0.97961051
0.000792359 0.046429008 0.271999782 0.787326444
0.953996967 -7.696480319 cg01763173 2
171674437 q GAD1 GAD1 NA 5'UTR island
5'UTR - island NA 0.046140877 0.046933236
0.000792359 0.983117316
-0.001965477 0.364177152 -0.26987675 0.788945197
0.954537768 -7.697070625 cg19227924 17
28565709 q SLC6A4 BLMH -9504 IGR shelf
IGR - shelf NA 0.364891871 0.362926394
-0.001965477 1.005415635
-0.002335797 0.250984559 -0.26883749 0.789737951
0.954537768 -7.697357911 cg13620631 9
87489528 q NTRK2 NTRK2 NA 3'UTR open
sea 3'UTR - open sea NA 0.25183394 0.249498143 -
0.002335797 1.009361982
-0.002020738 0.851947023 -0.267495835 0.790761712
0.954537768 -7.697727157 cg03564415 6
29638575 p GABBR1/MOG MOG NA 3'UTR open
sea 3'UTR - open sea NA 0.852681836 0.850661098
-0.002020738 1.002375491
-0.002616868 0.753943001 -0.266305058 0.79167066
0.954537768 -7.698053338 cg18772882 15
88616079 q NTRK3 NTRK3 NA Body open
sea Body - open sea NA 0.754894589 0.752277721
-0.002616868 1.003478593
0.001870775 0.157168537 0.266146103 0.791792017
0.954537768 -7.69809677 cg15385139 6
29595506 p GABBR1/MOG GABBR1 NA Body island
Body - island NA 0.156488256 0.158359031
0.001870775 0.988186496
-0.001879096 0.108947939 -0.265519904 0.792270149
0.954537768 -7.698267617 cg01863613 17
28443583 q SLC6A4 CCDC55 NA TSS1500 shore
TSS1500 - shore V$MEF2_01 0.109631246 0.10775215 -
0.001879096 1.017439058
0.000470455 0.039220005 0.264938879 0.792713862
0.954537768 -7.698425781 cg22860601 2
171627721 q GAD1 GAD1 -45479 IGR island
IGR - island NA 0.03904893 0.039519385
0.000470455 0.988095589
-0.001587942 0.889455648 -0.264099685 0.793354856
0.954537768 -7.698653614 cg18701449 12
72335228 q TPH2 TPH2 NA Body open
sea Body - open sea NA 0.890033082 0.88844514 -
0.001587942 1.001787327

-0.001060155 0.910891161 -0.262284446 0.794741872
0.954733043 -7.69914397 cg05157433 6
29527885 p GABBR1/MOG UBD NA TSS200 open
sea TSS200 - open sea NA 0.911276672 0.910216517
-0.001060155 1.001164728
-0.000617004 0.01643463 -0.262060592 0.794912965
0.954733043 -7.699204207 cg20891813 6
29691735 p GABBR1/MOG HLA-F NA Body island
Body - island NA 0.016658995 0.016041992
-0.000617004 1.038461745
-0.003741735 0.112551674 -0.260005902 0.796483858
0.95559479 -7.699754713 cg14193550 6 29621467 p
GABBR1/MOG MOG -3291 IGR shelf IGR - shelf
NA 0.113912305 0.11017057 -0.003741735
1.033963108
-0.001266245 0.905901913 -0.259295061 0.797027526
0.95559479 -7.699944162 cg27012424 18 3773224 p
DLGAP1 DLGAP1 NA Body shore Body - shore
NA 0.906362366 0.905096121 -0.001266245
1.001399017
0.001886733 0.806708863 0.257023469 0.798765582
0.956361152 -7.700546108 cg08829877 22
19960832 q COMT ARVCF NA Body island
Body - island NA 0.806022779 0.807909511
0.001886733 0.997664674
-0.000675147 0.085565736 -0.255622082 0.799838342
0.956361152 -7.700914829 cg23666278 2
171785822 q GAD1 GORASP2 NA 5'UTR island
5'UTR - island NA 0.085811244 0.085136097
-0.000675147 1.007930208
-0.001863114 0.095578038 -0.252333899 0.802356992
0.956361152 -7.701772104 cg06085713 18 3453726
p DLGAP1 TGIF1 NA 5'UTR shore 5'UTR -
shore NA 0.096255534 0.09439242 -0.001863114
1.019737962
-0.00060057 0.04150196 -0.252032107 0.802588263
0.956361152 -7.701850231 cg10253022 22
19929467 q COMT TXNRD2 NA TSS200 island
TSS200 - island NA 0.041720349 0.041119779
-0.00060057 1.01460538
-0.002121146 0.428172297 -0.250583224 0.803698834
0.956361152 -7.702224019 cg08862148 6
29595315 p GABBR1/MOG GABBR1 NA Body island
Body - island NA 0.428943623 0.426822476
-0.002121146 1.004969623
0.001338967 0.867036487 0.249565149 0.804479439
0.956361152 -7.702485382 cg21491555 22
19967786 q COMT ARVCF NA Body island
Body - island NA 0.86654959 0.867888557
0.001338967 0.998457213
0.000631488 0.041573727 0.249512042 0.804520165
0.956361152 -7.702498986 cg16203801 6
29716637 p MOG LOC285830 NA Body island
Body - island NA 0.041344095 0.041975583
0.000631488 0.984955825
0.002867159 0.20950456 0.249493438 0.804534431
0.956361152 -7.702503751 cg10234998 6

29601491 p GABBR1/MOG GABBR1 NA TSS1500 shore
TSS1500 - shore NA 0.208461957 0.211329116
0.002867159 0.986432731
-0.005232175 0.701123747 -0.24900827 0.80490651
0.956361152 -7.702627896 cg15626350 6
152130207 q ESR1 ESR1 NA Body shore
Body - shore NA 0.703026356 0.697794181
-0.005232175 1.007498164
0.00048768 0.013386794 0.248790138 0.805073813
0.956361152 -7.702683633 cg10687604 6
29720687 p MOG IFITM4P 2103 IGR island
IGR - island NA 0.013209455 0.013697136
0.00048768 0.964395404
0.001083296 0.081254022 0.248421253 0.80535676
0.956361152 -7.70277778 cg05634149 21
34395317 q OLIG2 OLIG2 -2899 IGR island
IGR - island V$ARP1_01 0.080860097 0.081943392
0.001083296 0.986779959
-0.002739716 0.149241033 -0.245631379 0.807497551
0.957037623 -7.703485306 cg25661973 21
34400733 q OLIG2 OLIG2 NA 3'UTR shore
3'UTR - shore NA 0.150237294 0.147497577
-0.002739716 1.018574658
-0.000888066 0.089540163 -0.245326753 0.807731395
0.957037623 -7.703562078 cg21569006 21
34443672 q OLIG2 OLIG1 NA 1stExon island
1stExon - island NA 0.089863096 0.08897503 -
0.000888066 1.00998107
-0.00165528 0.115027955 -0.244944068 0.808025186
0.957037623 -7.703658389 cg09205920 6
29521506 p GABBR1 UBD -1883 IGR island
IGR - island NA 0.115629876 0.113974595
-0.00165528 1.014523245
-0.001769845 0.909602877 -0.241929847 0.810340222
0.958387041 -7.704411743 cg26569800 6
29578313 p GABBR1/MOG GABBR1 NA Body open
sea Body - open sea NA 0.910246457 0.908476611
-0.001769845 1.001948147
0.000528427 0.032481736 0.240416301 0.81150334
0.958387041 -7.704786523 cg21241839 6
29716536 p MOG LOC285830 NA Body island
Body - island NA 0.032289581 0.032818008
0.000528427 0.983898261
0.001470928 0.886598344 0.240119712 0.811731312
0.958387041 -7.704859689 cg07436579 6
29581117 p GABBR1/MOG GABBR1 NA Body open
sea Body - open sea V$PAX5_01 0.886063461 0.887534389
0.001470928 0.998342681
-0.00165208 0.096282787 -0.239167662 0.812463212
0.958387041 -7.705093943 cg26840770 11
27723290 p BDNF BDNF NA TSS1500 shore
TSS1500 - shore V$STAT5A_02;V$BRN2_01 0.096883544
0.095231464 -0.00165208 1.017348048
0.00214073 0.822298553 0.238697117 0.812825013
0.958387041 -7.70520938 cg21552290 6
29578423 p GABBR1/MOG GABBR1 NA Body open

sea	Body - open sea	NA	0.821520105	0.823660835
	0.00214073 0.997400957
0.001424781	0.097045601	0.236922732	0.814189707
	0.958387041	-7.705642642	cg06350404 18	3452443
	p	DLGAP1	TGIF1	NA	5'UTR	shore	5'UTR -
shore	NA	0.096527499	0.09795228 0.001424781
	0.985454336
0.002989419	0.523721157	0.236757343	0.81431694
	0.958387041	-7.705682862	cg00504285 22
	20019887	q	COMT	C22orf25	NA	5'UTR	open
sea	5'UTR - open sea	NA	0.522634095	0.525623515
	0.002989419	0.994312621
0.000321551	0.02266421 0.234610474	0.81596897 0.958387041
	-7.706202406	cg07038887 17	28618554	q	SLC6A4
	BLMH	NA	Body	island	Body - island	NA
	0.022547282	0.022868834	0.000321551
	0.985939292
0.002529184	0.50353576 0.234151758	0.816322067
	0.958387041	-7.706312804	cg27322282 18	3411906
	p	DLGAP1	TGIF1	NA	TSS200	open sea	TSS200
- open sea NA	0.502616056	0.505145241	0.002529184
	0.994993153
0.002875197	0.304418238	0.233088899	0.817140352
	0.958387041	-7.706567772	cg25583651 6
	29570040	p	GABBR1/MOG GABBR1	NA	3'UTR	open
sea	3'UTR - open sea	NA	0.303372712	0.306247908
	0.002875197	0.990611541
-0.002608103	0.134537404	-0.232846629	0.817326903
	0.958387041	-7.706625728	cg08755130 6
	29705878	p	MOG	LOC285830 NA	Body	open
sea	Body - open sea	NA	0.135485805	0.132877703
	-0.002608103	1.019627838
-0.001581046	0.904741413	-0.231620705	0.818271044
	0.958387041	-7.706918074	cg05115679 6
	29593490	p	GABBR1/MOG GABBR1	NA	Body	shore
	Body - shore	NA	0.905316339	0.903735293
	-0.001581046	1.001749457
0.00368714 0.496887684	0.231619494	0.818271976
	0.958387041	-7.706918362	cg00390484 22
	20019695	q	COMT	C22orf25	NA	5'UTR	open
sea	5'UTR - open sea	NA	0.495546906	0.499234047
	0.00368714 0.992614404
-0.000862088	0.044014375	-0.230440321	0.819180373
	0.958619652	-7.707198106	cg11209538 6
	29600112	p	GABBR1/MOG GABBR1	NA	Body	shore
	Body - shore	NA	0.044327861	0.043465773
	-0.000862088	1.019833721
-0.00112781	0.921979988	-0.228536599	0.820647474
	0.958619652	-7.707646737	cg02589899 6
	29627167	p	GABBR1/MOG MOG	NA	Body	open
sea	Body - open sea	V$TAXCREB_02	0.922390101
	0.92126229 -0.00112781	1.001224202
0.002768524	0.550024466	0.228014897	0.821049638
	0.958619652	-7.707769034	cg14352032 17
	28564834	q	SLC6A4	BLMH	-10379	IGR	shore
	IGR - shore	NA	0.54901773 0.551786254
	0.002768524	0.994982615

-0.001133706 0.900894753 -0.227133668 0.821729061
0.958619652 -7.707974978 cg26920808 12
72237548 q TPH2 TBC1D15 NA Body shelf
Body - shelf NA 0.90130701 0.900173303 -
0.001133706 1.001259432
0.000795347 0.050028721 0.226815623 0.821974306
0.958619652 -7.70804911 cg06078334 6
29595653 p GABBR1/MOG GABBR1 NA Body island
Body - island NA 0.049739504 0.050534851
0.000795347 0.984261416
0.000369348 0.052082595 0.225388775 0.823074778
0.95884611 -7.708380415 cg17851017 6 29600994 p
GABBR1/MOG GABBR1 NA TSS200 shore TSS200 - shore
NA 0.051948287 0.052317635 0.000369348
0.992940277
-0.001584997 0.883627828 -0.224746438 0.823570307
0.95884611 -7.708528881 cg01024792 6 29524067 p
GABBR1 UBD NA Body shelf Body - shelf
NA 0.88420419 0.882619193 -0.001584997
1.001795788
-0.000791314 0.066384439 -0.223122306 0.824823565
0.95940152 -7.708902389 cg14519950 9 4662766 p
SLC1A1 C9orf68 NA Body island Body - island
NA 0.06667219 0.065880876 -0.000791314
1.012011285
-0.00183554 0.813034038 -0.221792328 0.825850187
0.95940152 -7.709206238 cg27368718 18 3883519 p
DLGAP1 DLGAP1-AS3 5339 IGR shelf IGR - shelf
NA 0.813701507 0.811865967 -0.00183554
1.00226089
0.00118687 0.09504026 0.221402305 0.826151309 0.95940152 -
7.709295 cg12296326 6 29717114 p MOG LOC285830 NA
TSS1500 island TSS1500 - island NA 0.094608671
0.095795541 0.00118687 0.987610384
-0.000526196 0.050273281 -0.217052537 0.829511399
0.962031764 -7.71027437 cg17911882 9 4679326
p SLC1A1 CDC37L1 NA TSS1500 island TSS1500
- island NA 0.050464625 0.049938429 -0.000526196
1.010536895
-0.001341005 0.913097071 -0.216649734 0.82982272
0.962031764 -7.710364083 cg15180617 6
29705436 p MOG LOC285830 NA Body open
sea Body - open sea NA 0.913584709 0.912243704
-0.001341005 1.001470007
-0.000916957 0.066701999 -0.215550016 0.830672822
0.962103536 -7.710608167 cg25328597 11
27722638 p BDNF BDNF NA TSS200 island
TSS200 - island NA 0.067035438 0.066118481
-0.000916957 1.013868392
-0.00338552 0.436110487 -0.213920859 0.83193257
0.962103536 -7.710967485 cg06591579 6
29461269 p GABBR1 MAS1L 6726 IGR open
sea IGR - open sea NA 0.437341585 0.433956065
-0.00338552 1.007801527
0.001855358 0.181309673 0.213840777 0.831994505
0.962103536 -7.710985077 cg06212263 21
34392851 q OLIG2 OLIG2 -5365 IGR shore

IGR - shore NA 0.180634997 0.182490355
0.001855358 0.989833117
-0.002233761 0.238543828 -0.210464421 0.834606758
0.9631545 -7.711720814 cg13696752 9 4662858 p
SLC1A1 C9orf68 NA Body island Body - island
V$CP2_01;V$E2F_03 0.239356105 0.237122343 -
0.002233761 1.009420293
0.002572826 0.783099278 0.207220546 0.837118312
0.9631545 -7.712416684 cg05309280 2 171785085 q
GAD1 GORASP2 NA TSS1500 shore TSS1500 - shore
NA 0.782163705 0.78473653 0.002572826
0.996721416
-0.001072274 0.142823996 -0.20647048 0.837699294
0.9631545 -7.712576052 cg08470639 9 87285186 q
NTRK2 NTRK2 NA 5'UTR island 5'UTR - island
NA 0.143213913 0.14214164 -0.001072274
1.007543694
0.000291408 0.011848119 0.206357015 0.83778719
0.9631545 -7.71260011 cg00716604 12 72233552 q
TPH2 TBC1D15 NA 1stExon island 1stExon - island
NA 0.011742153 0.01203356 0.000291408
0.975783808
-0.000650517 0.065097898 -0.205607207 0.838368079
0.9631545 -7.71275876 cg26391350 2 171673572 q
GAD1 GAD1 NA 5'UTR island 5'UTR - island
NA 0.06533445 0.064683933 -0.000650517
1.010056856
-0.000471619 0.040107411 -0.205116352 0.838748404
0.9631545 -7.712862307 cg00060933 6 29617602 p
GABBR1/MOG MOG -7156 IGR shore IGR - shore
NA 0.040278909 0.03980729 -0.000471619
1.011847554
0.000483916 0.041651892 0.202007085 0.841158437
0.9631545 -7.713512478 cg10969051 6 29617771 p
GABBR1/MOG MOG -6987 IGR island IGR - island
NA 0.041475923 0.041959839 0.000483916
0.988467163
0.002111316 0.196279024 0.199844267 0.842835786
0.9631545 -7.713958897 cg08729810 21 34396944 q
OLIG2 OLIG2 NA TSS1500 island TSS1500 - island
NA 0.195511273 0.197622589 0.002111316
0.989316424
-0.003173602 0.571110274 -0.198641817 0.843768656
0.9631545 -7.714205017 cg03570920 12 72332964 q
TPH2 TPH2 NA Body open sea Body - open sea
NA 0.572264311 0.569090709 -0.003173602
1.005576619
-0.000648377 0.926636925 -0.196071901 0.845763184
0.9631545 -7.714726065 cg04066686 6 29580347 p
GABBR1/MOG GABBR1 NA Body open sea Body - open sea
NA 0.926872698 0.926224321 -0.000648377
1.000700022
0.001057529 0.055883593 0.194817117 0.846737405
0.9631545 -7.714978012 cg00782607 2 171672156 q
GAD1 GAD1 NA TSS1500 shore TSS1500 - shore
NA 0.055499037 0.056556566 0.001057529
0.981301393

0.004410401 0.793173 0.193312835 0.847905663
0.9631545 -7.715277928 cg02155405 2 172776401 q
SLC25A12 HAT1 -2534 IGR shelf IGR - shelf
NA 0.791569217 0.795979619 0.004410401
0.994459152
0.000595656 0.069899596 0.193090164 0.848078623
0.9631545 -7.715322126 cg14075496 9 4679516 p
SLC1A1 CDC37L1 NA TSS200 island TSS200 - island
NA 0.069682993 0.07027865 0.000595656
0.991524353
0.000511674 0.058786322 0.192940524 0.848194861
0.9631545 -7.715351799 cg06971248 17 28618449 q
SLC6A4 BLMH NA Body island Body - island
NA 0.058600259 0.059111933 0.000511674
0.991343981
0.000999451 0.08464886 0.192813165 0.848293794
0.9631545 -7.715377036 cg20723129 6 29521501 p
GABBR1 UBD -1888 IGR island IGR - island
NA 0.084285424 0.085284874 0.000999451
0.98828104
0.000822354 0.924377158 0.190439599 0.850138048
0.9631545 -7.715844333 cg13811469 22 20020657 q
COMT C22orf25 NA 5'UTR open sea 5'UTR - open sea
NA 0.924078121 0.924900474 0.000822354
0.999110874
0.001464368 0.639372926 0.19033917 0.8502161 0.9631545 -
7.715863978 cg11629449 6 29556084 p GABBR1/MOG OR2H2
NA 1stExon open sea 1stExon - open sea V$PPARG_01
0.638840428 0.640304796 0.001464368
0.997713014
-0.00076638 0.038835987 -0.19022479 0.850304996
0.9631545 -7.715886339 cg07855056 17 28443892 q
SLC6A4 MIR423 NA TSS1500 island TSS1500 - island
NA 0.039114671 0.038348291 -0.00076638
1.019984724
-0.002304397 0.814836286 -0.187513863 0.852412516
0.9631545 -7.716412391 cg04507121 2 172778341 q
SLC25A12 HAT1 NA TSS1500 shore TSS1500 - shore
NA 0.815674248 0.813369851 -0.002304397
1.002833148
-0.000436567 0.018866651 -0.187069952 0.852757725
0.9631545 -7.716497814 cg15490715 6 29521568 p
GABBR1 UBD -1821 IGR island IGR - island
NA 0.019025403 0.018588835 -0.000436567
1.023485495
0.000803444 0.078506778 0.18555852 0.853933317
0.9631545 -7.716787147 cg24703717 6 29691168 p
GABBR1/MOG HLA-F NA 1stExon island 1stExon - island
NA 0.078214617 0.079018061 0.000803444
0.989832147
-0.002424367 0.532419916 -0.184960089 0.854398871
0.9631545 -7.716901057 cg23601416 22 19950040 q
COMT COMT NA 5'UTR open sea 5'UTR - open sea
NA 0.533301504 0.530877137 -0.002424367
1.00456672
-0.001420689 0.809425613 -0.183630514 0.855433413
0.9631545 -7.717152824 cg12790373 6 29635347 p

GABBR1/MOG MOG NA 3'UTR open sea 3'UTR - open sea
NA 0.809942227 0.808521538 -0.001420689
1.001757144
0.000542495 0.08374569 0.183065457 0.855873163
0.9631545 -7.717259274 cg21619773 6 29720720 p
MOG IFITM4P 2136 IGR island IGR - island
NA 0.083548419 0.084090914 0.000542495
0.993548708
-0.002177489 0.568019624 -0.182646102 0.856199552
0.9631545 -7.717338064 cg27642588 6 29709602 p
MOG LOC285830 NA Body open sea Body - open sea
NA 0.568811439 0.566633949 -0.002177489
1.003842851
0.001207692 0.147078713 0.182459561 0.856344747
0.9631545 -7.717373054 cg07476327 2 171730024 q
GAD1 GORASP2 -54924 IGR open sea IGR - open sea
NA 0.146639552 0.147847244 0.001207692
0.991831488
-0.00084497 0.923365243 -0.182333343 0.856442993
0.9631545 -7.717396709 cg08907436 6 152125965 q
ESR1 ESR1 NA 5'UTR shelf 5'UTR - shelf
NA 0.923672505 0.922827535 -0.00084497
1.000915632
-0.000447422 0.036434633 -0.181937419 0.856751187
0.9631545 -7.717470804 cg18117895 11 27722066 p
BDNF BDNF NA Body island Body - island
NA 0.036597332 0.03614991 -0.000447422
1.01237685
0.000831335 0.939376188 0.181911534 0.856771337
0.9631545 -7.717475642 cg01321962 6 152126441 q
ESR1 ESR1 NA 5'UTR shelf 5'UTR - shelf
NA 0.939073885 0.93990522 0.000831335
0.999115512
0.00132697 0.733741961 0.181470619 0.857114581
0.9631545 -7.717557956 cg04335343 6 29597113 p
GABBR1/MOG GABBR1 NA TSS1500 shore TSS1500 - shore
NA 0.733259426 0.734586396 0.00132697
0.998193582
0.000710532 0.937562238 0.18136736 0.85719497 0.9631545 -
7.717577205 cg14914809 2 171705073 q GAD1 GAD1
NA Body open sea Body - open sea NA
0.937303863 0.938014395 0.000710532
0.999242515
0.000997359 0.900473633 0.181216282 0.85731259
0.9631545 -7.717605348 cg18950940 6 29580896 p
GABBR1/MOG GABBR1 NA Body open sea Body - open sea
NA 0.900110957 0.901108316 0.000997359
0.998893186
0.003536352 0.610887185 0.180708924 0.857707614
0.9631545 -7.717699687 cg20709110 22 19946873 q
COMT COMT NA 5'UTR open sea 5'UTR - open sea
NA 0.609601239 0.613137591 0.003536352
0.994232368
0.000825688 0.89903045 0.180673946 0.857734849
0.9631545 -7.717706181 cg20592995 17 28524160 q
SLC6A4 SLC6A4 NA 3'UTR open sea 3'UTR - open sea

NA 0.8987302 0.899555888 0.000825688
0.999082116
0.001251734 0.919102288 0.180266875 0.858051817
0.9631545 -7.717781667 cg10503635 6 29634213 p
GABBR1/MOG MOG NA 3'UTR open sea 3'UTR - open sea
NA 0.918647112 0.919898847 0.001251734
0.998639269
-0.000948498 0.913733672 -0.179950972 0.858297815
0.9631545 -7.71784013 cg17706972 6 152126337 q
ESR1 ESR1 NA 5'UTR shelf 5'UTR - shelf
NA 0.914078581 0.913130083 -0.000948498
1.001038733
0.001368793 0.701311929 0.179025032 0.859018938
0.9631545 -7.718010901 cg12021641 6 29555593 p
GABBR1/MOG OR2H2 NA TSS200 open sea TSS200 - open sea
NA 0.700814186 0.702182979 0.001368793
0.998050661
-0.001507962 0.734089174 -0.177583127 0.860142139
0.9631545 -7.718275081 cg13040666 6 29693534 p
GABBR1/MOG HLA-F NA Body shore Body - shore
NA 0.734637523 0.733129561 -0.001507962
1.002056883
0.00162409 0.297444858 0.176716817 0.860817112
0.9631545 -7.718432777 cg09144707 2 171678251 q
GAD1 GAD1 NA Body shore Body - shore
NA 0.29685428 0.29847837 0.00162409 0.994558768
0.00036563 0.016424803 0.175674365 0.861629463
0.9631545 -7.718621516 cg09545452 2 172779111 q
SLC25A12 HAT1 NA Body island Body - island
NA 0.016291846 0.016657476 0.00036563
0.978050096
0.001494368 0.872836966 0.16973762 0.866258686
0.9631545 -7.719675134 cg18389339 18 3730593 p
DLGAP1 DLGAP1 NA Body shore Body - shore
NA 0.87229356 0.873787928 0.001494368
0.998289782
0.000456622 0.033806553 0.169324364 0.866581107
0.9631545 -7.71974713 cg19868007 6 29691890 p
GABBR1/MOG HLA-F NA Body island Body - island
NA 0.033640509 0.034097131 0.000456622
0.986608199
-0.000551205 0.087998107 -0.169223889 0.8666595
0.9631545 -7.719764608 cg24675879 17 28444056 q
SLC6A4 MIR423 NA TSS200 shore TSS200 - shore
NA 0.088198545 0.087647341 -0.000551205
1.006288884
0.000322728 0.033162876 0.168820587 0.866974183
0.9631545 -7.71983466 cg00740645 6 29617868 p
GABBR1/MOG MOG -6890 IGR island IGR - island
NA 0.033045521 0.033368249 0.000322728
0.990328291
-0.001000126 0.826261407 -0.168055591 0.867571142
0.9631545 -7.719967077 cg08415141 6 29598310 p
GABBR1/MOG GABBR1 NA Body shore Body - shore
NA 0.826625089 0.825624963 -0.001000126
1.001211356

-0.001429369 0.204230421 -0.167430943 0.868058641
0.9631545 -7.720074756 cg19601636 22 20001066 q
COMT ARVCF NA 5'UTR shelf 5'UTR - shelf
NA 0.204750192 0.203320823 -0.001429369
1.007030116
-0.000724485 0.099149991 -0.167093019 0.868322391
0.9631545 -7.720132842 cg11950383 21 34400072 q
OLIG2 OLIG2 NA Body island Body - island
NA 0.09941344 0.098688955 -0.000724485
1.007341095
0.001689336 0.863873614 0.166135073 0.869070153
0.9631545 -7.720296866 cg21147063 21 34354927 q
OLIG2 OLIG2 -43289 IGR shelf IGR - shelf
NA 0.86325931 0.864948646 0.001689336
0.998046894
-0.00079148 0.110220011 -0.165788185 0.86934096
0.9631545 -7.720356029 cg13723118 9 87284722 q
NTRK2 NTRK2 NA 5'UTR island 5'UTR - island
NA 0.110507822 0.109716342 -0.00079148
1.007213875
0.000993791 0.094478985 0.165649061 0.869449576
0.9631545 -7.720379723 cg06816235 11 27742219 p
BDNF BDNF NA Body island Body - island
NA 0.094117606 0.095111398 0.000993791
0.989551284
0.000315343 0.044743243 0.164971913 0.869978268
0.9631545 -7.720494762 cg03670115 2 172544069 q
SLC25A12 DYNC1I2 NA 5'UTR island 5'UTR - island
NA 0.044628572 0.044943916 0.000315343
0.992983611
0.000417689 0.07843117 0.164669301 0.870214556
0.9631545 -7.72054602 cg20104535 2 172750996 q
SLC25A12 SLC25A12 NA TSS200 island TSS200 - island
NA 0.078279283 0.078696972 0.000417689
0.994692439
-0.001018642 0.754725269 -0.164503663 0.870343896
0.9631545 -7.720574037 cg09265315 6 29572317 p
GABBR1/MOG GABBR1 NA Body open sea Body - open sea
NA 0.755095685 0.754077042 -0.001018642
1.001350847
0.001801241 0.432557542 0.163762607 0.8709226
0.9631545 -7.720699037 cg07059469 6 152421432 q
ESR1 ESR1 NA 3'UTR open sea 3'UTR - open sea
NA 0.431902545 0.433703786 0.001801241
0.99584684
0.000589305 0.05030549 0.162694572 0.871156777
0.963203756 -7.720878202 cg14278853 6
29521756 p GABBR1 UBD -1633 IGR island
IGR - island NA 0.050091197 0.050680502
0.000589305 0.988372155
0.000756536 0.059096203 0.161902729 0.872375332
0.963203756 -7.721010279 cg15673034 18 3499093
p DLGAP1 DLGAP1 NA 3'UTR island 3'UTR -
island NA 0.058821098 0.059577635 0.000756536
0.987301661
-0.002459117 0.173346377 -0.157434297 0.87586741
0.965993174 -7.721743541 cg06297863 6

29626990 p GABBR1/MOG MOG NA Body open
sea Body - open sea NA 0.174240602 0.171781485
-0.002459117 1.014315379
0.000902788 0.850680173 0.156863102 0.876313983
0.965993174 -7.721835796 cg13873869 6
29638112 p GABBR1/MOG MOG NA 3'UTR open
sea 3'UTR - open sea NA 0.850351886 0.851254674
0.000902788 0.998939462
0.000600117 0.065792583 0.152735375 0.879542345
0.968771278 -7.722492523 cg24671939 18 3593798
p DLGAP1 FLJ35776 NA TSS1500 open sea TSS1500
- open sea NA 0.065574358 0.066174475 0.000600117
0.990931292
0.000230051 0.015316028 0.150749032 0.881096646
0.969428137 -7.72280232 cg18587988 22
20008614 q COMT C22orf25 NA TSS200 island
TSS200 - island NA 0.015232373 0.015462425
0.000230051 0.985121868
0.001877795 0.495535008 0.148272024 0.883035561
0.969428137 -7.72318297 cg20139800 6
29599178 p GABBR1/MOG GABBR1 NA Body shore
Body - shore NA 0.494852173 0.496729968
0.001877795 0.996219687
-0.000759332 0.064566858 -0.147719099 0.883468471
0.969428137 -7.72326708 cg24710480 6
29717136 p MOG LOC285830 NA TSS1500 island
TSS1500 - island NA 0.064842978 0.064083646
-0.000759332 1.011849076
-0.000836734 0.080631759 -0.14729205 0.883802853
0.969428137 -7.723331827 cg21433429 18 4455713
p DLGAP1 DLGAP1-AS5 191111 IGR shore IGR -
shore NA 0.080936026 0.080099292 -0.000836734
1.01044621
-0.001242163 0.881040768 -0.146811066 0.884179492
0.969428137 -7.723404527 cg09517033 6
29461584 p GABBR1 MAS1L 7041 IGR open
sea IGR - open sea NA 0.881492463 0.8802503 -
0.001242163 1.001411147
0.000676108 0.931211627 0.14621546 0.884645925
0.969428137 -7.723494224 cg04419754 22
20044371 q COMT C22orf25 NA Body open
sea Body - open sea NA 0.930965769 0.931641877
0.000676108 0.999274283
-0.000665623 0.058972045 -0.143833801 0.886511472
0.969428137 -7.723849254 cg03152033 18 4453856
p DLGAP1 DLGAP1-AS5 189254 IGR shore IGR -
shore NA 0.059214089 0.058548466 -0.000665623
1.011368752
0.000750269 0.831767541 0.143032852 0.887139002
0.969428137 -7.723967342 cg00162046 22
20006956 q COMT TANGO2 -1675 IGR shore
IGR - shore NA 0.831494716 0.832244985
0.000750269 0.9990985
0.001035985 0.848846864 0.14248443 0.887568724
0.969428137 -7.724047818 cg14447193 9
87433864 q NTRK2 NTRK2 NA Body open

sea Body - open sea NA 0.848470142 0.849506128
0.001035985 0.998780484
0.001192572 0.096281274 0.142361127 0.887665344
0.969428137 -7.72406587 cg24531536 6
29520698 p GABBR1 UBD -2691 IGR shore
IGR - shore NA 0.095847612 0.097040184
0.001192572 0.987710534
0.000422939 0.060807538 0.141476842 0.88835832
0.969428137 -7.724194869 cg04991728 6
29720954 p MOG IFITM4P 2370 IGR island
IGR - island NA 0.060653743 0.061076681
0.000422939 0.993075295
0.001779303 0.60126131 0.141114295 0.888642459
0.969428137 -7.724247526 cg00504902 6
29692183 p GABBR1/MOG HLA-F NA Body shore
Body - shore NA 0.60061429 0.602393594
0.001779303 0.997046277
0.000984182 0.121926854 0.137985111 0.891095507
0.969464908 -7.7246964 cg11251858 6 152129036 q
ESR1 ESR1 NA 5'UTR island 5'UTR - island
NA 0.121568969 0.122553152 0.000984182
0.991969338
0.00063416 0.918220776 0.137746835 0.891282344
0.969464908 -7.724730168 cg25414639 15
88360194 q NTRK3 NTRK3-AS1 -59794 IGR open
sea IGR - open sea NA 0.917990173 0.918624332
0.00063416 0.999309664
-0.000271764 0.02582374 -0.137524975 0.891456313
0.969464908 -7.724761557 cg10201663 6
29720685 p MOG IFITM4P 2101 IGR island
IGR - island NA 0.025922563 0.025650799
-0.000271764 1.010594758
0.001687149 0.125507875 0.137149146 0.891751028
0.969464908 -7.724814615 cg20801464 18 3454953
p DLGAP1 TGIF1 NA 5'UTR shelf 5'UTR -
shelf NA 0.124894366 0.126581515 0.001687149
0.986671443
0.001245607 0.481485297 0.136551738 0.892219532
0.969464908 -7.724898656 cg11712482 22
19928667 q COMT COMT NA TSS1500 shore
TSS1500 - shore NA 0.481032348 0.482277956
0.001245607 0.99741724
0.001054057 0.164908363 0.132912777 0.895074149
0.97179479 -7.725402657 cg06874218 18 3447454 p
DLGAP1 TGIF1 NA 5'UTR shore 5'UTR - shore
NA 0.16452507 0.165579126 0.001054057
0.993634125
-0.000877925 0.862038635 -0.131153314 0.89645489
0.972522037 -7.725641468 cg08884395 6
152127887 q ESR1 ESR1 NA TSS1500 shore
TSS1500 - shore NA 0.86235788 0.861479955 -
0.000877925 1.001019089
0.000612629 0.11338613 0.130218759 0.897188416
0.972546556 -7.725767022 cg02219071 6
29596540 p GABBR1/MOG GABBR1 NA TSS1500 shore
TSS1500 - shore NA 0.113163356 0.113775984
0.000612629 0.994615489

-0.000372076	0.952643361	-0.126742169	0.899917972	0.974500005	-7.726226214	cg16448399	18	3880076	p	DLGAP1	DLGAP1	NA	1stExon	island	1stExon - island	NA	0.952778662	0.952406586	-0.000372076	1.000390669
-0.000570947	0.078561007	-0.126109018	0.900415209	0.974500005	-7.726308505	cg01636003	11	27723385	p	BDNF	BDNF	NA	TSS1500	shore	TSS1500 - shore	NA	0.078768624	0.078197677	-0.000570947	1.007301329
-0.004283918	0.699519108	-0.123227392	0.90267877	0.975561203	-7.726677831	cg00495303	18	3771110	p	DLGAP1	DLGAP1	NA	Body	shore	Body - shore	NA	0.701076896	0.696792978	-0.004283918	1.00614805
0.001354412	0.375453355	0.122950898	0.902896004	0.975561203	-7.72671282	cg02014853	6	29595335	p	GABBR1/MOG	GABBR1	NA	Body	island	Body - island	NA	0.374960842	0.376315254	0.001354412	0.996400858
0.000231905	0.019743935	0.120490682	0.904829263	0.975561203	-7.727020688	cg07252731	15	88799999	q	NTRK3	NTRK3	NA	TSS1500	island	TSS1500 - island	NA	0.019659606	0.01989151	0.000231905	0.988341559
-0.000392664	0.049214829	-0.120386714	0.904910976	0.975561203	-7.727033561	cg21108220	6	29716658	p	MOG	LOC285830	NA	Body	island	Body - island	NA	0.049357616	0.048964952	-0.000392664	1.008019287
0.000590404	0.893286887	0.120322582	0.904961379	0.975561203	-7.727041496	cg12728623	22	19938992	q	COMT	COMT	NA	5'UTR	open sea	5'UTR - open sea	NA	0.893072195	0.893662599	0.000590404	0.999339344
0.000806889	0.706673019	0.117445014	0.907223393	0.976987785	-7.727393205	cg16733589	6	29628224	p	GABBR1/MOG	MOG	NA	Body	open sea	Body - open sea	NA	0.706379605	0.707186494	0.000806889	0.998859015
-0.001806412	0.237324234	-0.11682222	0.907713066	0.976987785	-7.727468206	cg04831505	12	72233240	q	TPH2	TBC1D15	NA	TSS1500	shore	TSS1500 - shore	NA	0.237981111	0.236174699	-0.001806412	1.007648626
0.000243381	0.013251114	0.113642227	0.910213912	0.978895065	-7.727844951	cg14481263	22	20008608	q	COMT	C22orf25	NA	TSS200	island	TSS200 - island	NA	0.013162612	0.013405993	0.000243381	0.981845358
0.001164879	0.241273509	0.112749383	0.910916241	0.978895065	-7.727948861	cg02965237	21	34393668	q	OLIG2	OLIG2	-4548	IGR	shore	IGR - shore	NA	0.240849917	0.242014796	0.001164879	0.995186745
0.00079942	0.863077376	0.111349447	0.912017605	0.979309328	-7.728110139	cg09721630	6													

29523835 p GABBR1 UBD NA Body shelf
Body - shelf NA 0.862786677 0.863586097
0.00079942 0.999074302
0.00174287 0.569773549 0.109317059 0.913616849
0.979409402 -7.728340694 cg22546130 22
19950026 q COMT COMT NA 5'UTR open
sea 5'UTR - open sea NA 0.569139778 0.570882648
0.00174287 0.996947061
-0.000858001 0.850983517 -0.108203519 0.914493226
0.979409402 -7.728465214 cg12344104 6
29693309 p GABBR1/MOG HLA-F NA Body shore
Body - shore NA 0.851295518 0.850437517
-0.000858001 1.001008894
0.000402568 0.052452894 0.107975949 0.914672342
0.979409402 -7.728490506 cg06879567 18 3594243
p DLGAP1 DLGAP1 NA Body open sea Body -
open sea V$HTF_01;V$RFX1_01 0.052306506 0.052709074
0.000402568 0.992362454
-0.000220757 0.041368344 -0.10759197 0.914974573
0.979409402 -7.728533059 cg06025631 11
27722549 p BDNF BDNF NA Body island
Body - island NA 0.041448619 0.041227862
-0.000220757 1.005354559
0.000422336 0.926840123 0.106172282 0.916092128
0.979838961 -7.728689075 cg24312412 17
28512027 q SLC6A4 CCDC55 NA Body open
sea Body - open sea NA 0.926686546 0.927108882
0.000422336 0.999544459
0.001036018 0.858228672 0.104302035 0.917564619
0.980647186 -7.728891444 cg24938286 6
29631295 p GABBR1/MOG MOG NA Body open
sea Body - open sea NA 0.857851939 0.858887957
0.001036018 0.998793768
0.001473961 0.483613018 0.100667564 0.920426969
0.982288848 -7.729274431 cg15094605 6
29429346 p GABBR1 OR2H1 NA 5'UTR open
sea 5'UTR - open sea NA 0.483077032 0.484550993
0.001473961 0.996958089
-0.001092161 0.438005058 -0.100510384 0.920550782
0.982288848 -7.729290688 cg12257233 6
29597083 p GABBR1/MOG GABBR1 NA TSS1500 shore
TSS1500 - shore NA 0.438402208 0.437310047
-0.001092161 1.002497452
-0.000950185 0.898756837 -0.09946287 0.921375974
0.982288848 -7.729398382 cg12055610 6
29585658 p GABBR1/MOG GABBR1 NA Body open
sea Body - open sea NA 0.899102359 0.898152173
-0.000950185 1.001057934
0.000256293 0.052711978 0.098705233 0.921972866
0.982288848 -7.729475571 cg15914769 11
27722774 p BDNF BDNF NA TSS200 shore
TSS200 - shore NA 0.05261878 0.052875074
0.000256293 0.995152839
-0.000540781 0.792692388 -0.097137592 0.923208054
0.982301879 -7.729633412 cg21297992 22
19961060 q COMT ARVCF NA Body island

Body - island NA 0.792889035 0.792348255
-0.000540781 1.000682503
-0.000557021 0.095816741 -0.096867088 0.923421211
0.982301879 -7.729660393 cg10829693 21
34396221 q OLIG2 OLIG2 -1995 IGR island
IGR - island NA 0.096019294 0.095462273
-0.000557021 1.005834986
-0.00028382 0.048579041 -0.09551776 0.924484567
0.982668911 -7.729793855 cg27569822 17
28563119 q SLC6A4 SLC6A4 NA TSS200 island
TSS200 - island NA 0.048682248 0.048398428
-0.00028382 1.00586424
0.000587181 0.884453457 0.092626815 0.926763289
0.983711368 -7.7300735 cg03898786 6 29586020 p
GABBR1/MOG GABBR1 NA Body open sea Body - open sea
NA 0.884239936 0.884827117 0.000587181
0.999336389
-0.000291425 0.053596151 -0.092328359 0.926998577
0.983711368 -7.730101881 cg02365078 12
72234032 q TPH2 TBC1D15 NA Body shore
Body - shore NA 0.053702123 0.053410699
-0.000291425 1.005456285
0.001211933 0.48740437 0.090506953 0.928434624
0.983711368 -7.730273099 cg10635145 11
27742435 p BDNF BDNF NA Body shore
Body - shore NA 0.486963667 0.4881756
0.001211933 0.997517424
-0.000837244 0.857703478 -0.0898109 0.928983475
0.983711368 -7.730337629 cg18217459 6
152429948 q ESR1 SYNE1 -12871 IGR open
sea IGR - open sea NA 0.858007931 0.857170687
-0.000837244 1.000976753
-0.000465264 0.066131409 -0.089205946 0.929460522
0.983711368 -7.73039331 cg10022526 11
27744557 p BDNF BDNF NA TSS1500 island
TSS1500 - island NA 0.066300595 0.065835332
-0.000465264 1.007067072
-0.001372736 0.732184748 -0.088700756 0.92985892
0.983711368 -7.73043952 cg17806418 6
29599319 p GABBR1/MOG GABBR1 NA Body shore
Body - shore NA 0.732683924 0.731311189
-0.001372736 1.001877087
-0.000178754 0.03355066 -0.087159221 0.9310747 0.983711368
-7.730578903 cg12912949 22 20004611 q COMT
ARVCF NA TSS1500 island TSS1500 - island NA
0.033615662 0.033436908 -0.000178754
1.005346009
-0.001012636 0.199432347 -0.086977542 0.931217998
0.983711368 -7.730595169 cg21670199 15
88801401 q NTRK3 NTRK3-AS1 5440 IGR shore
IGR - shore V$AHRARNT_02 0.199800578
0.198787942 -0.001012636 1.005094051
-0.001476956 0.301495142 -0.085222234 0.932602604
0.98441386 -7.73075058 cg18342026 6 29521046 p
GABBR1 UBD -2343 IGR shore IGR - shore
NA 0.302032217 0.300555261 -0.001476956
1.004914091

-0.000429965 0.11050816 -0.081141055 0.9358227 0.986155925
-7.731099675 cg00107488 22 19930437 q COMT
COMT NA 5'UTR shore 5'UTR - shore NA
0.110664511 0.110234546 -0.000429965
1.003900456
0.000187209 0.048191551 0.080954099 0.935970237
0.986155925 -7.731115257 cg25122820 6
29716643 p MOG LOC285830 NA Body island
Body - island NA 0.048123475 0.048310684
0.000187209 0.996124894
-0.000441353 0.875140547 -0.080389757 0.936415604
0.986155925 -7.731162073 cg15313740 2
171625337 q GAD1 GAD1 -47863 IGR shore
IGR - shore NA 0.875301039 0.874859686
-0.000441353 1.000504484
0.000213109 0.051185531 0.07928239 0.937289575
0.98631703 -7.731252987 cg13773705 15 88799820 q
NTRK3 NTRK3 NA TSS200 island TSS200 - island
NA 0.051108037 0.051321146 0.000213109
0.99584754
-0.000192869 0.064920365 -0.076759571 0.939280962
0.987652848 -7.7314554 cg17302062 18 3451564 p
DLGAP1 TGIF1 NA 5'UTR island 5'UTR - island
V$P53_01 0.0649905 0.064797631 -0.000192869
1.002976482
0.001469415 0.712881565 0.075715855 0.940104936
0.987760025 -7.731537228 cg26038589 17
28444874 q SLC6A4 CCDC55 NA Body shore
Body - shore NA 0.712347232 0.713816648
0.001469415 0.997941466
0.000203721 0.076503745 0.072199665 0.942881322
0.989916845 -7.731804655 cg03538731 18 3451548
p DLGAP1 TGIF1 NA 5'UTR island 5'UTR -
island NA 0.076429665 0.076633386 0.000203721
0.997341616
0.000179223 0.042924099 0.069996187 0.944621562
0.990983357 -7.731965765 cg21472700 2
172543946 q SLC25A12 DYNC1I2 NA TSS200 island
TSS200 - island NA 0.042858927 0.04303815
0.000179223 0.995835718
0.00019669 0.097228771 0.068200706 0.946039784
0.991412599 -7.732093352 cg09606766 11
27722971 p BDNF BDNF NA TSS1500 shore
TSS1500 - shore NA 0.097157247 0.097353937
0.00019669 0.99797964
0.000233468 0.115456046 0.066439944 0.947430755
0.991412599 -7.732215254 cg19070841 6
29691643 p GABBR1/MOG HLA-F NA Body island
Body - island NA 0.115371149 0.115604617
0.000233468 0.997980461
-0.000868803 0.602677482 -0.066359153 0.947494583
0.991412599 -7.732220771 cg03296810 15
88826454 q NTRK3 NTRK3-AS1 30493 IGR open
sea IGR - open sea NA 0.602993411 0.602124607
-0.000868803 1.001442897
0.00060305 0.674535521 0.065808545 0.94792959 0.991412599
-7.732258191 cg16343924 6 29430158 p GABBR1

OR2H1 NA Body open sea Body - open sea NA
0.67431623 0.67491928 0.00060305 0.999106486
-0.00037515 0.162922983 -0.063739923 0.949564049
0.991461499 -7.732395991 cg00860808 2
171670500 q GAD1 GAD1 -2700 IGR island
IGR - island NA 0.163059401 0.162684251
-0.00037515 1.002306001
-0.000410494 0.722422776 -0.063330702 0.949887409
0.991461499 -7.73242273 cg22113926 17
28566331 q SLC6A4 BLMH -8882 IGR shelf
IGR - shelf NA 0.722572047 0.722161552
-0.000410494 1.000568425
-0.000269892 0.032848305 -0.0626965 0.950388562
0.991461499 -7.732463829 cg15980539 6
152128865 q ESR1 ESR1 NA 5'UTR island
5'UTR - island NA 0.032946448 0.032676556
-0.000269892 1.0082595
-0.000673318 0.688243704 -0.061335556 0.951464065
0.991461499 -7.732550628 cg19786983 15
88336651 q NTRK3 NTRK3-AS1 -83337 IGR open
sea IGR - open sea NA 0.688488547 0.687815229
-0.000673318 1.000978923
0.000417087 0.871896834 0.060852849 0.951845552
0.991461499 -7.732580957 cg20041152 15
88795689 q NTRK3 NTRK3 NA Body shelf
Body - shelf NA 0.871745166 0.872162253
0.000417087 0.999521778
0.000294055 0.147306121 0.060246386 0.952324861
0.991461499 -7.732618722 cg21091227 18 4454304
p DLGAP1 DLGAP1-AS5 189702 IGR island IGR -
island NA 0.147199192 0.147493246 0.000294055
0.998006322
-0.0006406 0.600930122 -0.057575069 0.954436309
0.992904084 -7.732780566 cg03037684 6
152421333 q ESR1 ESR1 NA 3'UTR open
sea 3'UTR - open sea NA 0.601163067 0.600522467
-0.0006406 1.001066738
-0.000555145 0.660540731 -0.055097531 0.95639489
0.993548476 -7.732924112 cg22906524 22
19960525 q COMT ARVCF NA Body island
Body - island V$MYCMAX_03;V$MYCMAX_02 0.660742602
0.660187456 -0.000555145 1.000840891
-0.000763518 0.715896782 -0.054077658 0.957201217
0.993548476 -7.732981368 cg05951817 17
28562142 q SLC6A4 SLC6A4 NA 5'UTR shore
5'UTR - shore NA 0.716174425 0.715410906
-0.000763518 1.001067245
-0.000574941 0.541058296 -0.054035474 0.95723457
0.993548476 -7.732983713 cg25385819 18 3445098
p DLGAP1 TGIF1 NA 5'UTR shelf 5'UTR -
shelf NA 0.541267365 0.540692424 -0.000574941
1.001063342
0.000351086 0.149969133 0.051072017 0.959577784
0.994631613 -7.733143886 cg03402459 6
29521407 p GABBR1 UBD -1982 IGR island
IGR - island NA 0.149841465 0.150192551
0.000351086 0.997662427

-0.000460309 0.301417814 -0.05087667 0.959732259
0.994631613 -7.733154127 cg14067066 6
29717475 p MOG LOC285830 NA TSS1500 shore
TSS1500 - shore NA 0.301585199 0.30112489 -
0.000460309 1.001528632
-0.000482199 0.222302962 -0.0474418 0.962448704
0.995582993 -7.733327789 cg01089249 2
171676553 q GAD1 GAD1 NA Body island
Body - island NA 0.222478307 0.221996108
-0.000482199 1.002172106
-0.00038991 0.168816388 -0.047417825 0.962467666
0.995582993 -7.733328959 cg20196537 2
171574548 q GAD1 SP5 2691 IGR shore
IGR - shore NA 0.168958174 0.168568264
-0.00038991 1.002313069
-0.000433171 0.120196076 -0.046955218 0.962833552
0.995582993 -7.733351409 cg02226939 17
28619481 q SLC6A4 BLMH NA TSS1500 shore
TSS1500 - shore NA 0.120353593 0.119920422
-0.000433171 1.003612154
0.000890733 0.297374903 0.045290021 0.964150659
0.995873783 -7.7334304 cg13638427 6 29617320 p
GABBR1/MOG MOG -7438 IGR shore IGR - shore
NA 0.297051 0.297941733 0.000890733
0.997010379
-0.000183118 0.063380672 -0.043535614 0.965538439
0.995873783 -7.733510537 cg13504245 9
87282610 q NTRK2 NTRK2 NA TSS1500 shore
TSS1500 - shore NA 0.063447261 0.063264143
-0.000183118 1.002894499
0.000128648 0.091608605 0.042280034 0.966531702
0.995873783 -7.733565946 cg00415702 6
29720841 p MOG IFITM4P 2257 IGR island
IGR - island NA 0.091561824 0.091690472
0.000128648 0.998596932
-0.000223801 0.097539606 -0.041175735 0.967405334
0.995873783 -7.733613339 cg03167496 11
27743619 p BDNF BDNF NA TSS200 island
TSS200 - island NA 0.097620988 0.097397187
-0.000223801 1.002297818
0.000206754 0.068107452 0.040188611 0.968186301
0.995873783 -7.733654641 cg14121971 6
29621375 p GABBR1/MOG MOG -3383 IGR shelf
IGR - shelf NA 0.068032269 0.068239023
0.000206754 0.99697015
0.000157505 0.036199181 0.040183687 0.968190197
0.995873783 -7.733654845 cg16044251 6
29521695 p GABBR1 UBD -1694 IGR island
IGR - island NA 0.036141906 0.036299412
0.000157505 0.995660921
0.000276794 0.818877567 0.039979296 0.968351906
0.995873783 -7.733663271 cg00721193 22
19966373 q COMT ARVCF NA Body shore
Body - shore NA 0.818776915 0.819053709
0.000276794 0.999662056
-0.000388652 0.146646545 -0.039237761 0.968938601
0.995873783 -7.733693479 cg16257091 11

27743580 p BDNF BDNF NA TSS1500 island
TSS1500 - island NA 0.146787873 0.146399221
-0.000388652 1.002654741
-0.000249209 0.066365574 -0.037790947 0.970083355
0.996301824 -7.73375079 ch.6.2949012F 6
152044517 q ESR1 ESR1 NA 5'UTR open
sea 5'UTR - open sea NA 0.066456195 0.066206986
-0.000249209 1.003764089
0.000212412 0.80728441 0.035282539 0.97206822 0.997574154
-7.733845051 cg06477632 6 29526338 p
GABBR1/MOG UBD NA Body open sea Body - open sea
NA 0.807207169 0.807419581 0.000212412
0.999736925
0.000240874 0.877938837 0.034382258 0.972780644
0.997574154 -7.733877304 cg23426002 11
27679729 p BDNF BDNF NA Body open
sea Body - open sea NA 0.877851246 0.87809212
0.000240874 0.999725685
0.000223369 0.100652658 0.031358954 0.975173251
0.998649044 -7.733979515 cg14692377 17
28562685 q SLC6A4 SLC6A4 NA 1stExon island
1stExon - island NA 0.100571433 0.100794801
0.000223369 0.997783933
-0.000119179 0.93486366 -0.03039831 0.975933544
0.998649044 -7.734010024 cg25756033 17
28512754 q SLC6A4 CCDC55 NA 3'UTR open
sea 3'UTR - open sea NA 0.934906997 0.934787819
-0.000119179 1.000127492
0.000162258 0.920576897 0.029321392 0.976785888
0.998649044 -7.734043097 cg17375177 6
29627845 p GABBR1/MOG MOG NA Body open
sea Body - open sea NA 0.920517894 0.920680152
0.000162258 0.999823763
-0.000142135 0.902755093 -0.028543111 0.977401888
0.998649044 -7.734066257 cg25736198 12
72345225 q TPH2 TPH2 NA Body open
sea Body - open sea NA 0.902806779 0.902664644
-0.000142135 1.000157462
-0.000207141 0.84393524 -0.028345761 0.97755809 0.998649044
-7.734072031 cg13175282 22 19938541 q COMT
COMT NA 5'UTR open sea 5'UTR - open sea NA
0.844010564 0.843803423 -0.000207141
1.000245485
0.000105694 0.114155522 0.027467928 0.978252905
0.998649044 -7.734097227 cg02858594 21
34400211 q OLIG2 OLIG2 NA 3'UTR island
3'UTR - island NA 0.114117088 0.114222781
0.000105694 0.999074677
0.000277909 0.766780541 0.026601303 0.978938866
0.998649044 -7.734121324 cg23708209 15
88843899 q NTRK3 NTRK3-AS1 47938 IGR open
sea IGR - open sea NA 0.766679483 0.766957392
0.000277909 0.999637647
0.000101276 0.089131261 0.024583621 0.980535987
0.999532958 -7.734174434 cg09444802 6
29691312 p GABBR1/MOG HLA-F NA Body island

Body - island NA 0.089094433 0.089195709
0.000101276 0.998864564
6.20E-05 0.064493357 0.021413991 0.983045108
0.999663219 -7.734249411 cg10672884 2
171785479 q GAD1 GORASP2 NA TSS1500 island
TSS1500 - island NA 0.064470802 0.064532828
6.20E-05 0.999038846
-4.36E-05 0.022848125 -0.021325145 0.983115442
0.999663219 -7.734251364 cg11576659 6
29691135 p GABBR1/MOG HLA-F NA 1stExon island
1stExon - island NA 0.022863987 0.022820366
-4.36E-05 1.001911494
7.33E-05 0.901512026 0.016581602 0.98687082 0.999663219
-7.734343836 cg21919834 22 19948833 q COMT
COMT NA 5'UTR open sea 5'UTR - open sea NA
0.901485365 0.901558684 7.33E-05 0.999918675
-3.48E-05 0.038248105 -0.01508981 0.988051912
0.999663219 -7.734368133 cg18518183 2
172544446 q SLC25A12 DYNC1I2 NA 5'UTR island
5'UTR - island NA 0.038260744 0.038225987
-3.48E-05 1.000909251
3.41E-05 0.044750009 0.014766776 0.988307671
0.999663219 -7.734373093 cg04000205 18 3450626
p DLGAP1 TGIF1 NA 5'UTR island 5'UTR -
island NA 0.044737619 0.044771693 3.41E-05
0.999238939
-6.57E-05 0.920663242 -0.014258047 0.988710453
0.999663219 -7.734380687 cg26744084 6
29581182 p GABBR1/MOG GABBR1 NA Body open
sea Body - open sea NA 0.920687145 0.920621412
-6.57E-05 1.000071401
4.44E-05 0.048906348 0.01365653 0.989186705 0.999663219
-7.734389322 cg13355041 18 3593715 p DLGAP1
FLJ35776 NA TSS1500 open sea TSS1500 - open sea
V$P53_01 0.0488902 0.048934607 4.44E-05 0.999092524
-7.58E-05 0.062788771 -0.01303096 0.989682004
0.999663219 -7.734397908 cg19549714 18 3447713
p DLGAP1 TGIF1 NA 5'UTR shore 5'UTR -
shore NA 0.062816319 0.062740562 -7.58E-05
1.001207464
-0.000224943 0.125936774 -0.012526713 0.990081248
0.999663219 -7.734404535 cg18374181 21
34401798 q OLIG2 OLIG2 3582 IGR shore
IGR - shore NA 0.126018571 0.125793629
-0.000224943 1.001788183
-0.000167061 0.385359925 -0.011611312 0.990806035
0.999663219 -7.734415898 cg25730428 6
29454755 p GABBR1 MAS1L NA 1stExon open
sea 1stExon - open sea NA 0.385420675 0.385253613
-0.000167061 1.000433642
9.15E-05 0.474331084 0.00985243 0.992198686 0.999663219
-7.734435313 cg09577455 6 29593382 p
GABBR1/MOG GABBR1 NA Body shore Body - shore
NA 0.474297819 0.474389298 9.15E-05
0.999807165
-3.96E-05 0.860736401 -0.008139706 0.993554814
0.999663219 -7.734451161 cg14841796 6

29598285 p GABBR1/MOG GABBR1 NA Body shore
Body - shore NA 0.860750794 0.860711212
-3.96E-05 1.000045988
-2.88E-05 0.084838973 -0.007982221 0.993679512
0.999663219 -7.734452467 cg17301635 18 3451475
p DLGAP1 TGIF1 NA 5'UTR island 5'UTR -
island NA 0.084849463 0.084820614 -2.88E-05
1.000340118
9.90E-05 0.771977942 0.00612451 0.995150464 0.999663219
-7.734465944 cg00601836 6 152130332 q ESR1
ESR1 NA Body shore Body - shore NA
0.771941958 0.772040914 9.90E-05 0.999871825
-5.41E-05 0.281456333 -0.005222689 0.995864541
0.999663219 -7.734471206 cg21185936 6
29716247 p MOG LOC285830 NA Body shore
Body - shore NA 0.281476007 0.281421904
-5.41E-05 1.000192249
1.20E-05 0.074297321 0.004984168 0.996053406
0.999663219 -7.734472458 cg10446968 2
172750661 q SLC25A12 SLC25A12 NA Body shore
Body - shore NA 0.074292968 0.074304938
1.20E-05 0.999838907
-4.60E-05 0.219367767 -0.00435021 0.996555387
0.999663219 -7.734475502 cg26142965 6
29521803 p GABBR1 UBD -1586 IGR island
IGR - island NA 0.219384487 0.219338506
-4.60E-05 1.000209635
2.62E-05 0.870940188 0.003854218 0.996948125
0.999663219 -7.734477594 cg18371410 6
29699406 p MOG LOC285830 NA Body open
sea Body - open sea NA 0.870930663 0.870956857
2.62E-05 0.999969925
-1.85E-05 0.058301362 -0.003826051 0.996970428
0.999663219 -7.734477706 cg08362738 11
27722636 p BDNF BDNF NA TSS200 island
TSS200 - island NA 0.0583081 0.05828957 -1.85E-05
1.000317896
5.75E-05 0.410519915 0.003262003 0.997417055
0.999663219 -7.734479762 cg06343355 6
29521023 p GABBR1 UBD -2366 IGR shore
IGR - shore NA 0.410499004 0.410556509
5.75E-05 0.999859934
-6.70E-06 0.074935479 -0.00236876 0.998124348
0.999663219 -7.734482348 cg11594927 6
29720600 p MOG IFITM4P 2016 IGR island
IGR - island NA 0.074937916 0.074931214
-6.70E-06 1.000089442
5.76E-06 0.074096242 0.00144777 0.998853614 0.999663219
-7.734484156 cg11092416 6 29617549 p
GABBR1/MOG MOG -7209 IGR shore IGR - shore
NA 0.074094149 0.074099906 5.76E-06
0.999922308
1.03E-05 0.162603614 0.001281736 0.998985084
0.999663219 -7.734484389 cg21348357 2
171574592 q GAD1 SP5 2735 IGR shore
IGR - shore NA 0.162599886 0.162610138
1.03E-05 0.999936954

-4.02E-06 0.044412538 -0.001162612 0.99907941 0.999663219
-7.734484539 cg06991510 11 27723237 p BDNF
BDNF NA TSS1500 shore TSS1500 - shore NA
0.044413999 0.044409982 -4.02E-06 1.000090453
-4.66E-06 0.071971547 -0.001138505 0.999098499
0.999663219 -7.734484567 cg17521020 6
29617613 p GABBR1/MOG MOG -7145 IGR shore
IGR - shore NA 0.071973241 0.071968583
-4.66E-06 1.000064723
5.78E-06 0.615265118 0.00042532 0.999663219 0.999663219
-7.734485141 cg04856117 22 19949901 q COMT
COMT NA 5'UTR open sea 5'UTR - open sea NA
0.615263018 0.615268795 5.78E-06 0.999990611
