# Supplementary material for: DNA Methylation at the Neonatal State and at the Time of Diagnosis: Preliminary Support for an Association with the Estrogen Receptor 1, Gamma-Aminobutyric Acid B Receptor 1, and Myelin Oligodendrocyte Glycoprotein in Female Adolescent Patients with OCD
Source: Front Psychiatry. 2016 Mar 18;7:35. doi: 10.3389/fpsyt.2016.00035 (PMC4796012; doi:10.3389/fpsyt.2016.00035)
Supplement: Supplementary file 3 [file table_3.PDF]

logFC	AveExpr	t	P.Value	adj.P.Val	B	probeID	CHR	MAPINFO	arm	OCD_GENE(+/-100.000bp)	gene	distancetoGene	feature	cgi	feat.cgi	conserved_tfbs	Case_OLD_AVG	Ctrl_OLD_AVG	deltaBeta	Fold_change(Case/Ctrl)
-0.043923047	0.338441927	-4.160695459	0.000225986	0.176177735	-0.666833991	cg10234998	6	29601491	p	GABBR1/MOG	GABBR1	NA	TSS1500	shore	TSS1500 - shore	NA	0.353540474	0.309617428	-0.043923047	1.141862318
-0.047135875	0.418263738	-4.114323622	0.00025757	0.176177735	-0.794961818	cg17099072	6	29601489	p	GABBR1/MOG	GABBR1	NA	TSS1500	shore	TSS1500 - shore	NA	0.434466695	0.38733082	-0.047135875	1.121694099
-0.007765897	0.053215381	-3.606636995	0.001053024	0.480179119	-2.165158991	cg03851143	6	29691250	p	GABBR1/MOG	HLA-F	NA	1stExon	island	1stExon - island	NA	0.055884908	0.048119012	-0.007765897	1.161389349
0.030815407	0.854418515	3.353133865	0.002083064	0.59404672	-2.821716225	cg25077271	6	29614368	p	GABBR1/MOG	MOG	-10390	IGR	shelf	IGR - shelf	NA	0.843825719	0.874641126	0.030815407	0.964767942
0.009836474	0.07249404	3.297604966	0.002413266	0.59404672	-2.962568291	cg10446968	2	172750661	q	SLC25A12	SLC25A12	NA	Body	shore	Body - shore	NA	0.069112752	0.078949226	0.009836474	0.875407594
0.017687616	0.108716326	3.171995544	0.003355073	0.59404672	-3.276870796	cg12760563	18	3594396	p	DLGAP1	DLGAP1	NA	Body	open sea	Body - open sea	NA	0.102636208	0.120323824	0.017687616	0.852999885
-0.057039598	0.565715919	-3.152861059	0.003526269	0.59404672	-3.324202507	cg21100518	6	29595002	p	GABBR1/MOG	GABBR1	NA	Body	shore	Body - shore	NA	0.58532328	0.528283682	-0.057039598	1.107971531
-0.025741617	0.727468243	-3.023444932	0.004921973	0.59404672	-3.64032045	cg16343924	6	29430158	p	GABBR1	OR2H1	NA	Body	open sea	Body - open sea	NA	0.736316924	0.710575307	-0.025741617	1.036226445
0.094358728	0.424881857	3.008638347	0.005111549	0.59404672	-3.676027948	cg21114334	6	29720137	p	MOG	IFITM4P	NA	TSS1500	shore	TSS1500 - shore	NA	0.392446044	0.486804772	0.094358728	0.806167208
-0.036705111	0.879393679	-2.968666629	0.005658429	0.59404672	-3.771935426	cg08289189	22	19937277	q	COMT	COMT	NA	5'UTR	open sea	5'UTR - open sea	NA	0.892011061	0.85530595	-0.036705111	1.042914598
-0.04755445	0.636406231	-2.889069904	0.006916071	0.59404672	-3.960743016	cg06519422	6	29599226	p											

GABBR1/MOG GABBR1 NA Body shore Body - shore
NA 0.652753073 0.605198623 -0.04755445
1.0785766
0.142434732 0.556527023 2.878516937 0.007101332
0.59404672 -3.985552517 cg18505691 6 29723320 p
MOG IFITM4P 4736 IGR shelf IGR - shelf
NA 0.507565084 0.649999816 0.142434732
0.780869581
-0.010639296 0.916791609 -2.825616596 0.008102413
0.59404672 -4.109112438 cg24437600 12 72361228 q
TPH2 TPH2 NA Body open sea Body - open sea
NA 0.920448868 0.909809571 -0.010639296
1.011693982
0.018915042 0.279496491 2.811629499 0.008388425
0.59404672 -4.141554469 cg10262357 6 29691832 p
GABBR1/MOG HLA-F NA Body island Body - island
NA 0.272994445 0.291909487 0.018915042
0.935202373
0.005629528 0.044714596 2.804846729 0.008530501
0.59404672 -4.157251856 cg13770529 17 28443770 q
SLC6A4 CCDC55 NA TSS200 island TSS200 - island
NA 0.042779446 0.048408974 0.005629528
0.883709
-0.015711839 0.848362399 -2.802613319 0.008577775
0.59404672 -4.162415657 cg14841796 6 29598285 p
GABBR1/MOG GABBR1 NA Body shore Body - shore
NA 0.853763344 0.838051504 -0.015711839
1.01874806
-0.016539506 0.147123013 -2.799767691 0.008638363
0.59404672 -4.168991363 cg15385139 6 29595506 p
GABBR1/MOG GABBR1 NA Body island Body - island
NA 0.152808468 0.136268962 -0.016539506
1.121373978
-0.037992707 0.846902079 -2.771703845 0.009257715
0.59404672 -4.233625099 cg23627083 2 171787727 q
GAD1 GORASP2 NA Body shore Body - shore
NA 0.859962072 0.821969364 -0.037992707
1.046221562
0.049539697 0.569797124 2.753024333 0.009692711
0.59404672 -4.276426033 cg06301399 6 29634495 p
GABBR1/MOG MOG NA 3'UTR open sea 3'UTR - open sea
NA 0.552767854 0.60230755 0.049539697
0.917750166
-0.016359186 0.83728901 -2.720122284 0.010505636
0.59404672 -4.351382457 cg02681389 6 29570781 p
GABBR1/MOG GABBR1 NA 3'UTR open sea 3'UTR - open sea
NA 0.842912481 0.826553294 -0.016359186
1.019792053
-0.022434963 0.63776068 -2.711112153 0.010739091
0.59404672 -4.371811706 cg21879791 6 29594830 p
GABBR1/MOG GABBR1 NA Body shore Body - shore
NA 0.645472699 0.623037736 -0.022434963
1.036008995
0.00965739 0.083969608 2.707920132 0.01082295 0.59404672 -
4.379039063 cg04672351 11 27722889 p BDNF BDNF
NA TSS1500 shore TSS1500 - shore NA
0.08064988 0.09030727 0.00965739 0.893060769

0.011753017 0.237504012 2.69278272 0.011228994
0.59404672 -4.41324071 cg19456996 6 29600642 p
GABBR1/MOG GABBR1 NA 5'UTR island 5'UTR - island
NA 0.233463913 0.24521693 0.011753017
0.95207094
-0.014441032 0.873866693 -2.689121148 0.011329318
0.59404672 -4.42149568 cg16324072 22 19978018 q
COMT ARVCF NA Body shelf Body - shelf
NA 0.878830798 0.864389766 -0.014441032
1.016706621
0.005066688 0.025427029 2.683240764 0.011492181
0.59404672 -4.434738181 cg21053831 18 4455512 p
DLGAP1 DLGAP1-AS5 190910 IGR island IGR - island
NA 0.023685355 0.028752042 0.005066688
0.823779925
0.014502551 0.098871649 2.667523853 0.01193822
0.59404672 -4.470042692 ch.2.3495108F 2
172804051 q SLC25A12 HAT1 NA Body open
sea Body - open sea NA 0.093886397 0.108388948
0.014502551 0.866198987
0.141719253 0.580650521 2.658856172 0.012191031
0.59404672 -4.489456594 cg24900542 6 29723315 p
MOG IFITM4P 4731 IGR shelf IGR - shelf
NA 0.531934528 0.673653781 0.141719253
0.789625981
0.005291429 0.030323343 2.637680871 0.012829695
0.59404672 -4.536716128 cg22869726 21 34398265 q
OLIG2 OLIG2 NA 1stExon island 1stExon - island
NA 0.028504414 0.033795843 0.005291429
0.84342959
-0.019405998 0.909818817 -2.634349532 0.01293295
0.59404672 -4.544129139 cg20884110 6 152464870 q
ESR1 SYNE1 NA Body open sea Body - open sea
NA 0.916489629 0.897083631 -0.019405998
1.021632318
0.124959512 0.541079186 2.629473341 0.013085476
0.59404672 -4.554968996 cg11201654 6 29690766 p
GABBR1/MOG HLA-F NA TSS1500 shore TSS1500 - shore
NA 0.498124353 0.623083865 0.124959512
0.799449931
-0.014301801 0.909555205 -2.614092849 0.013577561
0.59404672 -4.589075608 cg14809932 6 29525723 p
GABBR1/MOG UBD NA Body shelf Body - shelf
NA 0.914471449 0.900169648 -0.014301801
1.015887895
-0.007074453 0.087964669 -2.580309044 0.014719125
0.59404672 -4.663537644 cg01779447 6 29720315 p
MOG IFITM4P NA TSS1500 shore TSS1500 - shore
NA 0.090396512 0.083322058 -0.007074453
1.084904936
-0.008399968 0.06701469 -2.562642684 0.015350766
0.59404672 -4.702224302 cg17587327 2 171670878 q
GAD1 GAD1 -2322 IGR shore IGR - shore
NA 0.069902179 0.061502211 -0.008399968
1.136579935
-0.039777354 0.474617198 -2.543448349 0.016065302
0.59404672 -4.744059159 cg25642476 6 29595011 p

GABBR1/MOG GABBR1 NA Body shore Body - shore
NA 0.488290664 0.44851331 -0.039777354
1.088687121
0.009561671 0.063165833 2.53682437 0.016318914
0.59404672 -4.758448249 cg13612689 6 152128634 q
ESR1 ESR1 NA 5'UTR shore 5'UTR - shore
NA 0.059879008 0.069440679 0.009561671
0.862304471
0.005496153 0.022670634 2.509714905 0.01739571
0.59404672 -4.817077803 cg23248007 6 29716601 p
MOG LOC285830 NA Body island Body - island
NA 0.020781332 0.026277485 0.005496153
0.790841742
-0.012277118 0.188240894 -2.506133445 0.017542749
0.59404672 -4.82479203 cg00594408 6 29595349 p
GABBR1/MOG GABBR1 NA Body island Body - island
NA 0.192461153 0.180184035 -0.012277118
1.068136547
0.058905638 0.499859487 2.503636742 0.017645926
0.59404672 -4.830165407 cg03147503 6 29633969 p
GABBR1/MOG MOG NA 3'UTR open sea 3'UTR - open sea
NA 0.479610674 0.538516312 0.058905638
0.890614942
0.005336747 0.050571579 2.486068816 0.018387828
0.59404672 -4.867873372 cg25777153 22 20008297 q
COMT C22orf25 NA TSS1500 shore TSS1500 - shore
NA 0.048737072 0.054073819 0.005336747
0.901306268
-0.01551653 0.896600743 -2.472774146 0.018968162
0.59404672 -4.896290383 cg07238832 11 27681475 p
BDNF BDNF NA Body open sea Body - open sea
NA 0.90193455 0.88641802 -0.01551653 1.017504755
0.026701377 0.160575109 2.465590547 0.019288674
0.59404672 -4.911602299 cg08863440 2 171680337 q
GAD1 GAD1 NA Body island Body - island
NA 0.151396511 0.178097888 0.026701377
0.850074713
-0.012185342 0.920849642 -2.450183726 0.019992865
0.59404672 -4.944340188 cg15054873 6 152464791 q
ESR1 SYNE1 NA Body open sea Body - open sea
NA 0.925038353 0.912853011 -0.012185342
1.013348635
0.009696513 0.06220482 2.447842082 0.020101928
0.59404672 -4.949303722 cg07375883 18 3448693 p
DLGAP1 TGIF1 NA 5'UTR island 5'UTR - island
NA 0.058871643 0.068568156 0.009696513
0.85858577
-0.020603533 0.90648948 -2.444818673 0.020243549
0.59404672 -4.955707598 cg14787188 6 29636011 p
GABBR1/MOG MOG NA 3'UTR open sea 3'UTR - open sea
NA 0.913571945 0.892968412 -0.020603533
1.023073082
0.02135406 0.802675311 2.429921544 0.020954758
0.59404672 -4.987182216 cg00140112 18 3879595 p
DLGAP1 DLGAP1 NA 1stExon island 1stExon - island
NA 0.795334853 0.816688912 0.02135406
0.973852885

0.003689821 0.013738527 2.426409566 0.021125714
0.59404672 -4.994583132 cg24738387 2 171627280 q
GAD1 GAD1 -45920 IGR island IGR - island
NA 0.012470151 0.016159972 0.003689821
0.771669097
0.007435532 0.040076178 2.426097426 0.02114097
0.59404672 -4.995240561 cg06106763 21 34444104 q
OLIG2 OLIG1 NA 1stExon island 1stExon - island
NA 0.037520214 0.044955746 0.007435532
0.834603301
-0.0198829 0.310035625 -2.420843426 0.021399271
0.59404672 -5.006297813 cg19227924 17 28565709 q
SLC6A4 BLMH -9504 IGR shelf IGR - shelf
NA 0.316870372 0.296987472 -0.0198829
1.066948615
-0.012835497 0.077643746 -2.418414975 0.021519631
0.59404672 -5.011403019 cg27646908 12 72332608 q
TPH2 TPH2 NA TSS200 open sea TSS200 - open sea
NA 0.082055948 0.069220451 -0.012835497
1.18542926
0.00533524 0.015371028 2.407717566 0.022057202
0.59404672 -5.033849632 cg20891813 6 29691735 p
GABBR1/MOG HLA-F NA Body island Body - island
NA 0.013537039 0.01887228 0.00533524 0.717297486
0.04846766 0.557347887 2.405964054 0.022146479
0.59404672 -5.03752252 cg22298860 6 29690822 p
GABBR1/MOG HLA-F NA TSS1500 shore TSS1500 - shore
NA 0.540687129 0.589154789 0.04846766
0.917733572
0.01168551 0.117085069 2.384208097 0.023281846
0.607034703 -5.082938233 cg20627916 6
152128328 q ESR1 ESR1 NA TSS1500 shore
TSS1500 - shore V$OCT1_02 0.113068175 0.124753685
0.01168551 0.906331344
-0.023114078 0.891714072 -2.379801569 0.023518157
0.607034703 -5.092102013 cg18354203 11
27696004 p BDNF BDNF NA Body open
sea Body - open sea NA 0.899659536 0.876545458
-0.023114078 1.026369514
-0.014544204 0.816931353 -2.360048716 0.024604472
0.615201041 -5.133034549 cg09083279 6
29454873 p GABBR1 MAS1L NA 1stExon open
sea 1stExon - open sea NA 0.821930924 0.807386719
-0.014544204 1.018013926
0.015496447 0.066887289 2.353644671 0.024966322
0.615201041 -5.146253983 cg14121971 6
29621375 p GABBR1/MOG MOG -3383 IGR shelf
IGR - shelf NA 0.061560385 0.077056833
0.015496447 0.798895862
0.022647967 0.84158053 2.345361849 0.025441486
0.615201041 -5.163314247 cg08698936 6
29689809 p GABBR1/MOG HLA-F NA TSS1500 shore
TSS1500 - shore NA 0.833795291 0.856443258
0.022647967 0.973555788
0.006744002 0.054271351 2.336343571 0.02596815
0.615201041 -5.181841205 cg03732762 21
34443010 q OLIG2 OLIG1 NA 1stExon island

1stExon - island NA 0.0519531 0.058697102
0.006744002 0.885105026
-0.015057377 0.598716967 -2.334397537 0.026083085
0.615201041 -5.185832495 cg11588423 6
29578191 p GABBR1/MOG GABBR1 NA Body open
sea Body - open sea NA 0.60389294 0.588835563 -
0.015057377 1.025571446
0.016217957 0.83377068 2.323366543 0.026743337
0.6200828 -5.208412488 cg23330212 11 27672697 p
BDNF BDNFOS NA Body open sea Body - open sea
NA 0.828195757 0.844413714 0.016217957
0.980793826
0.070989109 0.394582363 2.295499288 0.028479158
0.632759818 -5.265117232 cg14172108 21
34405553 q OLIG2 OLIG2 7337 IGR shore
IGR - shore NA 0.370179857 0.441168966
0.070989109 0.839088616
0.010840565 0.04229361 2.275224502 0.029805265
0.632759818 -5.306065427 cg24377657 11
27723245 p BDNF BDNF NA TSS1500 shore
TSS1500 - shore NA 0.038567166 0.049407731
0.010840565 0.780589702
-0.016228309 0.38502513 -2.272464465 0.029990028
0.632759818 -5.311619608 cg08862148 6
29595315 p GABBR1/MOG GABBR1 NA Body island
Body - island NA 0.390603611 0.374375302
-0.016228309 1.043347702
-0.018116724 0.894230074 -2.26940981 0.030195716
0.632759818 -5.317761012 cg08190562 6
29528774 p GABBR1/MOG UBD NA TSS1500 open
sea TSS1500 - open sea NA 0.900457698 0.882340974
-0.018116724 1.020532566
0.046784986 0.595000521 2.266170431 0.030415229
0.632759818 -5.324267311 cg06486622 6
29718119 p MOG LOC285830 NA TSS1500 shore
TSS1500 - shore NA 0.578918182 0.625703169
0.046784986 0.925228144
0.012103736 0.907237637 2.258748764 0.030923573
0.632759818 -5.339148479 cg27297993 6
29581228 p GABBR1/MOG GABBR1 NA Body open
sea Body - open sea V$PAX4_04 0.903076978 0.915180714
0.012103736 0.986774485
0.013145185 0.067210538 2.257805181 0.030988748
0.632759818 -5.341037932 cg27316393 6
152128675 q ESR1 ESR1 NA 5'UTR shore
5'UTR - shore NA 0.06269188 0.075837066
0.013145185 0.82666542
-0.012412841 0.080418544 -2.257780863 0.03099043
0.632759818 -5.341086619 cg25729445 6
29595347 p GABBR1/MOG GABBR1 NA Body island
Body - island NA 0.084685458 0.072272617
-0.012412841 1.171750263
0.012901689 0.766424654 2.244339325 0.031932409
0.635221272 -5.367940039 cg13927803 6
29524042 p GABBR1 UBD NA Body shelf
Body - shelf NA 0.761989698 0.774891387
0.012901689 0.983350326

0.017259076	0.875682579	2.232035145	0.032817141
0.635221272	-5.392419275	cg16971273 6
29634336 p	GABBR1/MOG MOG	NA	3'UTR	open
sea	3'UTR - open sea	NA	0.869749771	0.887008848
0.017259076	0.980542385
0.005321474	0.058394184	2.21958563 0.03373464 0.635221272
-5.417087927	cg09547815 6	29691943	p
GABBR1/MOG HLA-F	NA	Body	island	Body - island
NA	0.056564927	0.061886401	0.005321474
0.914012224
0.013318362	0.101757095	2.219582892	0.033734844
0.635221272	-5.417093342	cg02892153 18	3593461
p	DLGAP1	FLJ35776	NA	TSS1500	open sea	TSS1500
- open sea NA	0.097178908	0.11049727 0.013318362
0.879468859
0.036789993	0.696917519	2.21246932 0.034269356
0.635221272	-5.431143603	cg02475474 6
29635158	p	GABBR1/MOG MOG	NA	3'UTR	open
sea	3'UTR - open sea	NA	0.684270959	0.721060951
0.036789993	0.948977972
0.02370552 0.705814333	2.209306237	0.034509455
0.635221272	-5.437380519	cg21224669 6
29639803	p	GABBR1/MOG MOG	NA	3'UTR	open
sea	3'UTR - open sea	NA	0.69766556 0.721371081
0.02370552 0.967138243
0.028805525	0.602861241	2.204017256	0.034914289
0.635221272	-5.44779464	cg00390484 22
20019695	q	COMT	C22orf25	NA	5'UTR	open
sea	5'UTR - open sea	NA	0.592959342	0.621764866
0.028805525	0.953671355
-0.020040519	0.454427073	-2.199877255	0.035234136
0.635221272	-5.455933619	cg08093277 6
29595299	p	GABBR1/MOG GABBR1	NA	Body	island
Body - island	NA	0.461316001	0.441275482
-0.020040519	1.045414984
0.014085893	0.079438299	2.193715859	0.035715001
0.635221272	-5.468025711	cg03747251 11
27722722	p	BDNF	BDNF	NA	TSS200	shore
TSS200 - shore	NA	0.074596273	0.088682166
0.014085893	0.84116431
0.009363328	0.846734945	2.187454232	0.036209689
0.635221272	-5.480288944	cg04960880 6
29577006	p	GABBR1/MOG GABBR1	NA	Body	open
sea	Body - open sea	NA	0.843516301	0.852879629
0.009363328	0.989021513
-0.00886807	0.917693437	-2.175011759	0.037210875
0.635221272	-5.504580443	cg22098375 6
29590966	p	GABBR1/MOG GABBR1	NA	Body	open
sea	Body - open sea	NA	0.920741836	0.911873766
-0.00886807	1.009725107
-0.010026143	0.926645677	-2.172171265	0.037442869
0.635221272	-5.510111594	cg15543523 6
152127812 q	ESR1	ESR1	NA	TSS1500	shore
TSS1500 - shore	NA	0.930092164	0.920066021
-0.010026143	1.0108972
0.009292375	0.928412619	2.170646727	0.037567915
0.635221272	-5.513078043	cg18116160 6

29575145 p GABBR1/MOG GABBR1 NA Body open
sea Body - open sea NA 0.925218364 0.93451074
0.009292375 0.990056427
0.008553587 0.064127061 2.170112939 0.037611786
0.635221272 -5.514116325 cg03837627 17
28443756 q SLC6A4 CCDC55 NA TSS200 island
TSS200 - island NA 0.061186765 0.069740352
0.008553587 0.877350963
0.007899083 0.060821825 2.148432542 0.039432661
0.637203176 -5.556127008 cg10523903 18 4455646
p DLGAP1 DLGAP1-AS5 191044 IGR island IGR -
island V$PAX5_01;V$AHRARNT_01 0.058106516 0.066005599
0.007899083 0.880327076
0.024577991 0.72176183 2.148405508 0.039434979
0.637203176 -5.556179197 cg18056738 6
29715160 p MOG LOC285830 NA Body shore
Body - shore NA 0.713313146 0.737891137
0.024577991 0.966691576
-0.01277853 0.912667366 -2.147574213 0.039506337
0.637203176 -5.557783757 cg13499155 12
72376553 q TPH2 TPH2 NA Body open
sea Body - open sea NA 0.917059985 0.904281456
-0.01277853 1.014131141
0.007016111 0.060504111 2.146574498 0.039592303
0.637203176 -5.559712788 cg11854392 22
19879320 q COMT TXNRD2 NA Body open
sea Body - open sea NA 0.058092323 0.065108434
0.007016111 0.892239598
0.007925423 0.103171813 2.138790159 0.040267391
0.63918179 -5.574710371 cg25346576 17 28443852 q
SLC6A4 CCDC55 NA 5'UTR island 5'UTR - island
NA 0.100447449 0.108372872 0.007925423
0.92686894
0.093059919 0.576942156 2.100186228 0.043768575
0.63918179 -5.648481904 cg18280909 6 29723301 p
MOG IFITM4P 4717 IGR shelf IGR - shelf
NA 0.544952809 0.638012728 0.093059919
0.854140968
-0.016085559 0.872625728 -2.09798489 0.043976111
0.63918179 -5.652658128 cg23306453 6 29599012 p
GABBR1/MOG GABBR1 NA Body shore Body - shore
NA 0.878155139 0.86206958 -0.016085559
1.018659235
-0.007854134 0.064842006 -2.095521746 0.044209362
0.63918179 -5.657327112 cg06515159 21 34400659 q
OLIG2 OLIG2 NA 3'UTR shore 3'UTR - shore
NA 0.067541864 0.059687731 -0.007854134
1.131587059
-0.004561783 0.044498109 -2.093239888 0.04442642
0.63918179 -5.661648759 cg12766058 6 29720894 p
MOG IFITM4P 2310 IGR island IGR - island
NA 0.046066222 0.041504439 -0.004561783
1.109910725
0.010613059 0.050249411 2.092311633 0.044514989
0.63918179 -5.663405778 cg08362738 11 27722636 p
BDNF BDNF NA TSS200 island TSS200 - island

NA 0.046601173 0.057214231 0.010613059
0.814503178
-0.013609605 0.851785847 -2.091950347 0.044549502
0.63918179 -5.664089467 cg14291693 11 27683959 p
BDNF BDNF NA Body open sea Body - open sea
NA 0.856464149 0.842854544 -0.013609605
1.016147039
0.006361909 0.04920294 2.085654131 0.045154792
0.63918179 -5.675989925 cg17290446 12 72233346 q
TPH2 TBC1D15 NA TSS200 island TSS200 - island
NA 0.047016034 0.053377943 0.006361909
0.880813897
-0.006833104 0.071907279 -2.082990724 0.045413018
0.63918179 -5.681015841 cg03565659 12 72332624 q
TPH2 TPH2 NA TSS200 open sea TSS200 - open sea
NA 0.074256159 0.067423055 -0.006833104
1.101346698
-0.022861196 0.784576538 -2.072142305 0.046478328
0.63918179 -5.701436686 cg27491398 6 29708311 p
MOG LOC285830 NA Body open sea Body - open sea
NA 0.792435074 0.769573878 -0.022861196
1.029706305
0.036402639 0.39439786 2.068711511 0.046819788
0.63918179 -5.707877865 cg20108357 11 27718978 p
BDNF BDNF NA Body shelf Body - shelf
NA 0.381884452 0.418287092 0.036402639
0.912972117
-0.018487432 0.758169195 -2.062354782 0.047458308
0.63918179 -5.719790881 cg01176329 6 29592381 p
GABBR1/MOG GABBR1 NA Body shelf Body - shelf
NA 0.764524249 0.746036817 -0.018487432
1.024780857
-0.019610792 0.226624428 -2.061374999 0.047557404
0.63918179 -5.721624585 cg04018625 2 171608293 q
GAD1 SP5 36436 IGR open sea IGR - open sea
NA 0.233365638 0.213754846 -0.019610792
1.091744315
-0.015526932 0.886365042 -2.058384036 0.047861042
0.63918179 -5.727218184 cg05358170 6 29692399 p
GABBR1/MOG HLA-F NA Body shore Body - shore
NA 0.891702425 0.876175493 -0.015526932
1.017721258
0.008255773 0.889718674 2.057696243 0.047931106
0.63918179 -5.728503596 cg14080521 11 27761859 p
BDNF BDNF-AS 85417 IGR open sea IGR - open sea
NA 0.886880752 0.895136525 0.008255773
0.99077708
0.008008966 0.05086662 2.056129992 0.048090995
0.63918179 -5.731429525 cg15673034 18 3499093 p
DLGAP1 DLGAP1 NA 3'UTR island 3'UTR - island
NA 0.048113538 0.056122504 0.008008966
0.857294928
0.007226885 0.087719286 2.050914037 0.04862685
0.63918179 -5.741161221 cg18867480 11 27744816 p
BDNF BDNF NA TSS1500 shore TSS1500 - shore
NA 0.085235045 0.092461929 0.007226885
0.921839355

-0.0176204 0.86141061 -2.049072847 0.048817253 0.63918179 -
5.744591917 cg10665848 6 29631447 p GABBR1/MOG MOG
NA Body open sea Body - open sea NA
0.867467622 0.849847223 -0.0176204 1.020733608
0.004154881 0.0463472 2.032522082 0.050558458
0.63918179 -5.775324921 cg17111795 17 28618907 q
SLC6A4 BLMH NA 1stExon island 1stExon - island
NA 0.04491896 0.049073841 0.004154881
0.915334098
-0.016884755 0.864388823 -2.028038001 0.051039491
0.63918179 -5.783618412 cg03739409 12 72335078 q
TPH2 TPH2 NA Body open sea Body - open sea
NA 0.870192958 0.853308203 -0.016884755
1.019787405
0.016257134 0.837848625 2.026264306 0.05123087
0.63918179 -5.786895044 cg17606558 6 29456563 p
GABBR1 MAS1L NA TSS1500 open sea TSS1500 - open
sea NA 0.832260235 0.848517369 0.016257134
0.980840541
0.010556736 0.824882537 2.018620646 0.05206283
0.63918179 -5.800990261 cg18055585 15 88668892 q
NTRK3 NTRK3 NA Body open sea Body - open sea
NA 0.821253659 0.831810395 0.010556736
0.987308723
-0.010856094 0.888322527 -2.016232169 0.052325216
0.63918179 -5.805386282 cg10110652 6 29589927 p
GABBR1/MOG GABBR1 NA Body open sea Body - open sea
NA 0.892054309 0.881198215 -0.010856094
1.012319696
0.006318801 0.066181012 2.013823632 0.052590978
0.63918179 -5.809815156 cg00298481 11 27722063 p
BDNF BDNF NA Body island Body - island
NA 0.064008924 0.070327725 0.006318801
0.910152063
0.011293257 0.08347692 2.00444557 0.053637063 0.63918179 -
5.827020752 cg26840770 11 27723290 p BDNF BDNF
NA TSS1500 shore TSS1500 - shore
V$STAT5A_02;V$BRN2_01 0.079594863 0.09088812
0.011293257 0.87574551
0.028678805 0.444754986 2.000767929 0.054052232
0.63918179 -5.833751016 cg20720918 2 171785124 q
GAD1 GORASP2 NA TSS1500 shore TSS1500 - shore
NA 0.434896647 0.463575452 0.028678805
0.938135626
0.005317925 0.139381204 1.999307122 0.05421792
0.63918179 -5.836421706 cg18095109 6 29720427 p
MOG IFITM4P 1843 IGR island IGR - island
NA 0.137553167 0.142871092 0.005317925
0.962778159
0.010516276 0.859328134 1.996080904 0.05458542
0.63918179 -5.842314611 cg02274336 6 29427807 p
GABBR1 OR2H1 NA 5'UTR open sea 5'UTR - open sea
NA 0.855713164 0.86622944 0.010516276
0.987859711
-0.011612476 0.81230401 -1.994990372 0.054710134
0.63918179 -5.844304872 cg21797131 22 19970601 q
COMT ARVCF NA Body shelf Body - shelf

NA 0.816295799 0.804683322 -0.011612476
1.014431114
0.010529677 0.041311583 1.992489469 0.05499708
0.63918179 -5.848865922 cg02324737 2 172543905 q
SLC25A12 DYNC1I2 NA TSS200 island TSS200 - island
NA 0.037692006 0.048221683 0.010529677
0.781640201
0.025112042 0.843437024 1.992284252 0.055020685
0.63918179 -5.849239991 cg03150111 6 29718049 p
MOG LOC285830 NA TSS1500 shore TSS1500 - shore
NA 0.834804759 0.859916801 0.025112042
0.970797126
0.01371052 0.068511811 1.9922241 0.055027605 0.63918179 -
5.849349631 cg08755130 6 29705878 p MOG
LOC285830 NA Body open sea Body - open sea NA
0.06379882 0.07750934 0.01371052 0.823111382
0.005288739 0.088658292 1.991299268 0.055134102
0.63918179 -5.851035005 cg24675879 17 28444056 q
SLC6A4 MIR423 NA TSS200 shore TSS200 - shore
NA 0.086840288 0.092129028 0.005288739
0.942594206
-0.033350827 0.079259149 -1.983109329 0.056085073
0.639455074 -5.865933427 cg01426208 6
29720641 p MOG IFITM4P 2057 IGR island
IGR - island NA 0.090723496 0.057372668
-0.033350827 1.581301675
-0.0126253 0.089294016 -1.97972719 0.056481944
0.639455074 -5.872071972 cg24710480 6
29717136 p MOG LOC285830 NA TSS1500 island
TSS1500 - island NA 0.093633963 0.081008664
-0.0126253 1.155851219
-0.022258661 0.812361083 -1.97906454 0.056559988
0.639455074 -5.873273719 cg17264941 6
29698405 p MOG LOC285830 NA Body open
sea Body - open sea NA 0.820012498 0.797753837
-0.022258661 1.027901666
-0.018866272 0.349052285 -1.968683551 0.057794944
0.648061338 -5.892059131 cg07601542 21
34396986 q OLIG2 OLIG2 NA TSS1500 island
TSS1500 - island NA 0.355537566 0.336671294
-0.018866272 1.056037661
-0.002566149 0.017029332 -1.963713915 0.058394415
0.64823099 -5.901024848 cg16956031 18 3451545 p
DLGAP1 TGIF1 NA 5'UTR island 5'UTR - island
NA 0.017911446 0.015345297 -0.002566149
1.167227066
0.028358886 0.264512364 1.958621978 0.059014244
0.64823099 -5.910192812 cg07704699 11 27742832 p
BDNF BDNF NA Body shore Body - shore
NA 0.254763997 0.283122883 0.028358886
0.899835415
0.006007717 0.043889786 1.955142733 0.059441047
0.64823099 -5.916446418 cg04648747 12 72233970 q
TPH2 TBC1D15 NA Body shore Body - shore
NA 0.041824633 0.04783235 0.006007717
0.874400547

-0.014281082 0.860067757 -1.952997943 0.059705486
0.64823099 -5.920297126 cg01465527 6 29548535 p
GABBR1/MOG SNORD32B NA TSS1500 open sea TSS1500 - open
sea NA 0.864976879 0.850695797 -0.014281082
1.016787531
-0.013773076 0.860198202 -1.941272017 0.061169362
0.653757441 -5.941290898 cg17532626 6
29589036 p GABBR1/MOG GABBR1 NA Body open
sea Body - open sea NA 0.864932697 0.851159621
-0.013773076 1.016181543
0.00701622 0.061911992 1.940157687 0.061310084
0.653757441 -5.94328079 cg24438313 6
29717010 p MOG LOC285830 NA TSS200 island
TSS200 - island NA 0.059500167 0.066516387
0.00701622 0.894518925
-0.042442783 0.588279492 -1.931533153 0.062408748
0.653757441 -5.958651433 cg17806418 6
29599319 p GABBR1/MOG GABBR1 NA Body shore
Body - shore NA 0.602869199 0.560426416
-0.042442783 1.075733016
0.00584391 0.063867046 1.927623103 0.062912438
0.653757441 -5.96560213 cg00033220 18 3451606
p DLGAP1 TGIF1 NA 5'UTR island 5'UTR -
island NA 0.061858202 0.067702112 0.00584391
0.913682013
-0.02060793 0.906481446 -1.921043043 0.063768027
0.653757441 -5.977274049 cg03063857 6
29585617 p GABBR1/MOG GABBR1 NA Body open
sea Body - open sea NA 0.913565422 0.892957492
-0.02060793 1.023078288
0.007310531 0.054338666 1.918505366 0.064100675
0.653757441 -5.98176703 cg26813908 17
28443598 q SLC6A4 CCDC55 NA TSS1500 shore
TSS1500 - shore NA 0.051825671 0.059136202
0.007310531 0.876378077
-0.006647886 0.074180584 -1.917996009 0.064167624
0.653757441 -5.982668284 cg24544803 6
29596840 p GABBR1/MOG GABBR1 NA TSS1500 shore
TSS1500 - shore NA 0.076465795 0.069817909
-0.006647886 1.095217489
0.06803161 0.381149712 1.914623887 0.064612377
0.653757441 -5.988630127 cg04186657 6
29690893 p GABBR1/MOG HLA-F NA TSS1500 shore
TSS1500 - shore NA 0.357763846 0.425795456
0.06803161 0.840224669
-0.037949027 0.715170851 -1.912883356 0.064842979
0.653757441 -5.991704099 cg26038589 17
28444874 q SLC6A4 CCDC55 NA Body shore
Body - shore NA 0.728215829 0.690266802
-0.037949027 1.054977332
-0.016997435 0.903598184 -1.909790284 0.065254534
0.653757441 -5.997161341 cg27318000 18 3845667
p DLGAP1 DLGAP1 NA TSS1500 open sea TSS1500
- open sea NA 0.909441052 0.892443617 -0.016997435
1.019045948
0.00375195 0.044991261 1.904191439 0.066005242
0.653757441 -6.007021808 cg08967211 6

29596174 p GABBR1/MOG GABBR1 NA Body shore
Body - shore NA 0.043701528 0.047453478
0.00375195 0.920934141
-0.018474033 0.856838182 -1.901662249 0.0663468
0.653757441 -6.011468581 cg01715172 6
152128024 q ESR1 ESR1 NA TSS1500 shore
TSS1500 - shore NA 0.86318863 0.844714597 -
0.018474033 1.021870148
-0.003445616 0.042215406 -1.900257503 0.066537164
0.653757441 -6.013936348 cg10491628 6
29521220 p GABBR1 UBD -2169 IGR island
IGR - island NA 0.043399837 0.039954221
-0.003445616 1.086239098
0.011809835 0.11663936 1.890533539 0.067867873
0.653757441 -6.030979002 cg11423684 6
29691993 p GABBR1/MOG HLA-F NA Body island
Body - island NA 0.112579729 0.124389565
0.011809835 0.905057663
-0.01635575 0.871772869 -1.889492939 0.068011627
0.653757441 -6.03279868 cg03534481 21
34439545 q OLIG2 OLIG1 -2905 IGR shelf
IGR - shelf NA 0.877395158 0.861039408
-0.01635575 1.018995356
0.017085104 0.17086724 1.884143196 0.068754816
0.653757441 -6.042141063 cg00089464 6
29717223 p MOG LOC285830 NA TSS1500 shore
TSS1500 - shore NA 0.164994236 0.18207934
0.017085104 0.906166707
0.034962182 0.66849001 1.884056531 0.068766913
0.653757441 -6.042292234 cg01644592 6
29633971 p GABBR1/MOG MOG NA 3'UTR open
sea 3'UTR - open sea NA 0.65647176 0.691433942
0.034962182 0.949435253
0.031029511 0.734024522 1.88370089 0.068816573
0.653757441 -6.042912526 cg13813710 6
29726626 p MOG IFITM4P 8042 IGR open
sea IGR - open sea NA 0.723358128 0.754387639
0.031029511 0.958867949
-0.014551663 0.902971614 -1.87587667 0.069916952
0.659068456 -6.056535501 cg12055610 6
29585658 p GABBR1/MOG GABBR1 NA Body open
sea Body - open sea NA 0.907973748 0.893422085
-0.014551663 1.016287557
0.00486158 0.093169605 1.871531134 0.070534608
0.659068456 -6.064082054 cg25456960 6
29717019 p MOG LOC285830 NA TSS200 island
TSS200 - island NA 0.091498437 0.096360017
0.00486158 0.949547747
-0.017866166 0.335625913 -1.866969952 0.071187954
0.659068456 -6.07198803 cg02014853 6
29595335 p GABBR1/MOG GABBR1 NA Body island
Body - island NA 0.341767408 0.323901242
-0.017866166 1.055159301
0.012159091 0.194790895 1.863344445 0.071710974
0.659068456 -6.078261157 cg21535772 2
171679906 q GAD1 GAD1 NA Body island

Body - island NA 0.190611207 0.202770298
0.012159091 0.940035148
0.02959383 0.214949902 1.860707498 0.072093453
0.659068456 -6.082817659 cg11497864 6
29717269 p MOG LOC285830 NA TSS1500 shore
TSS1500 - shore NA 0.204777023 0.234370853
0.02959383 0.873730758
-0.02783338 0.462152972 -1.859519913 0.072266278
0.659068456 -6.08486805 cg12991385 6
29599259 p GABBR1/MOG GABBR1 NA Body shore
Body - shore NA 0.471720697 0.443887316
-0.02783338 1.062703709
0.043674015 0.392677735 1.851949992 0.073376277
0.664759914 -6.097912965 cg05279622 6
29629758 p GABBR1/MOG MOG NA Body open
sea Body - open sea NA 0.377664792 0.421338807
0.043674015 0.896344665
-0.014861721 0.154855483 -1.844497219 0.07448334
0.669638313 -6.110714184 cg16713743 21
34397135 q OLIG2 OLIG2 NA TSS1500 island
TSS1500 - island NA 0.1599642 0.145102479 -
0.014861721 1.10242224
0.01131184 0.0921832 1.839593615 0.075219512 0.669638313
-6.119114151 cg06350404 18 3452443 p DLGAP1
TGIF1 NA 5'UTR shore 5'UTR - shore NA
0.088294755 0.099606594 0.01131184 0.886434838
-0.014073866 0.105561652 -1.838508486 0.07538326
0.669638313 -6.120970563 cg06684850 11
27742369 p BDNF BDNF NA Body shore
Body - shore NA 0.110399544 0.096325678
-0.014073866 1.146107106
-0.006198989 0.0537446 -1.830589846 0.07658745 0.669989693
-6.134490788 cg01453816 6 29600108 p
GABBR1/MOG GABBR1 NA Body shore Body - shore
V$TAXCREB_02 0.055875502 0.049676513 -
0.006198989 1.12478712
0.025558021 0.649378832 1.828013766 0.076982721
0.669989693 -6.138879001 cg09313122 11
27827916 p BDNF BDNF-AS 151474 IGR open
sea IGR - open sea NA 0.640593263 0.666151283
0.025558021 0.96163331
0.010404746 0.909445033 1.826079922 0.077280592
0.669989693 -6.142169915 cg19700470 6
29589973 p GABBR1/MOG GABBR1 NA Body open
sea Body - open sea NA 0.905868402 0.916273148
0.010404746 0.988644493
0.009002531 0.921527114 1.825423979 0.077381851
0.669989693 -6.143285524 cg21569150 6
29580360 p GABBR1/MOG GABBR1 NA Body open
sea Body - open sea NA 0.918432494 0.927435025
0.009002531 0.990293087
0.012867036 0.794172982 1.812603316 0.079383836
0.67440944 -6.165025332 cg04765420 6 29698866 p
MOG LOC285830 NA Body open sea Body - open sea
NA 0.789749938 0.802616974 0.012867036
0.983968647

-0.026563529 0.533008088 -1.811569946 0.079547105
0.67440944 -6.16677219 cg01765653 6 29599160 p
GABBR1/MOG GABBR1 NA Body shore Body - shore
NA 0.542139301 0.515575772 -0.026563529
1.051522066
-0.025648645 0.22721588 -1.810139887 0.079773521
0.67440944 -6.169188295 cg24531536 6 29520698 p
GABBR1 UBD -2691 IGR shore IGR - shore
NA 0.236032601 0.210383956 -0.025648645
1.121913503
0.012571296 0.2416059 1.809567644 0.079864276
0.67440944 -6.170154675 cg08805037 22 20009275 q
COMT C22orf25 NA 5'UTR shore 5'UTR - shore
NA 0.237284517 0.249855813 0.012571296
0.949685797
0.018406564 0.75655152 1.801582785 0.081139832
0.678239783 -6.18361324 cg12546695 6
29425610 p GABBR1 OR2H1 NA TSS1500 open
sea TSS1500 - open sea NA 0.750224264 0.768630828
0.018406564 0.97605279
-0.026960059 0.84904526 -1.790608451 0.082921185
0.678239783 -6.202031517 cg13504410 2
172546870 q SLC25A12 DYNC1I2 NA Body shelf
Body - shelf NA 0.858312781 0.831352722
-0.026960059 1.032429146
0.007700336 0.055199624 1.788293675 0.083301131
0.678239783 -6.205904696 cg21756465 2
172543834 q SLC25A12 DYNC1I2 NA TSS200 shore
TSS200 - shore NA 0.052552634 0.06025297
0.007700336 0.872199893
-0.012115541 0.846135146 -1.785192434 0.083812481
0.678239783 -6.211087412 cg24984698 17
28548496 q SLC6A4 SLC6A4 NA Body open
sea Body - open sea NA 0.850299864 0.838184322
-0.012115541 1.014454508
-0.0162905 0.913274719 -1.784918942 0.083857703
0.678239783 -6.211544112 cg04369302 6
29581439 p GABBR1/MOG GABBR1 NA Body open
sea Body - open sea NA 0.918874579 0.902584079
-0.0162905 1.018048734
0.016973279 0.34929036 1.782923835 0.084188225
0.678239783 -6.214873983 cg08805586 6
29689688 p GABBR1/MOG HLA-F NA TSS1500 shore
TSS1500 - shore NA 0.343455796 0.360429074
0.016973279 0.952908133
0.005283749 0.056209842 1.781908126 0.084356917
0.678239783 -6.216568051 cg06971248 17
28618449 q SLC6A4 BLMH NA Body island
Body - island NA 0.054393553 0.059677302
0.005283749 0.911461329
0.044619459 0.608278532 1.780200644 0.084641148
0.678239783 -6.219414128 cg21013431 18 3495888
p DLGAP1 DLGAP1-AS1 -142 IGR shelf IGR -
shelf NA 0.592940592 0.637560052 0.044619459
0.930015283
-0.007063096 0.936521467 -1.779368432 0.084779973
0.678239783 -6.220800472 cg01321962 6

152126441 q ESR1 ESR1 NA 5'UTR shelf
5'UTR - shelf NA 0.938949407 0.931886311
-0.007063096 1.007579354
0.004071232 0.028622343 1.773061642 0.085838324
0.678976296 -6.231289423 cg00414306 2
172779015 q SLC25A12 HAT1 NA 1stExon island
1stExon - island NA 0.027222857 0.031294089
0.004071232 0.869904122
0.006104507 0.090119549 1.772905353 0.085864692
0.678976296 -6.231548962 cg13933279 15
88418502 q NTRK3 NTRK3-AS1 -1486 IGR open
sea IGR - open sea NA 0.088021124 0.094125631
0.006104507 0.935145115
-0.006865311 0.930902437 -1.769449167 0.086449562
0.679672418 -6.237283663 cg25756033 17
28512754 q SLC6A4 CCDC55 NA 3'UTR open
sea 3'UTR - open sea NA 0.933262388 0.926397077
-0.006865311 1.007410765
0.007315388 0.122567183 1.765379116 0.087142631
0.681206393 -6.244025157 cg11718030 11
27744363 p BDNF BDNF NA TSS1500 island
TSS1500 - island NA 0.120052518 0.127367906
0.007315388 0.942564903
0.005665348 0.069496688 1.752714586 0.089329332
0.694332534 -6.264920685 cg01642653 11
27743476 p BDNF BDNF NA TSS1500 island
TSS1500 - island V$BRACH_01 0.067549225 0.073214573
0.005665348 0.922619941
0.013098844 0.121810838 1.748007968 0.090153712
0.694987334 -6.272654712 cg19846314 2
171680113 q GAD1 GAD1 NA Body island
Body - island NA 0.11730811 0.130406954
0.013098844 0.899554099
-0.016938174 0.878529934 -1.746335728 0.09044815
0.694987334 -6.275398453 cg23266797 6
29636003 p GABBR1/MOG MOG NA 3'UTR open
sea 3'UTR - open sea NA 0.884352432 0.867414257
-0.016938174 1.019527204
0.02074311 0.603061156 1.739034386 0.091743242
0.694987334 -6.287352818 cg03296810 15
88826454 q NTRK3 NTRK3-AS1 30493 IGR open
sea IGR - open sea NA 0.595930712 0.616673822
0.02074311 0.966362915
-0.0097543 0.123218481 -1.736699426 0.092160692
0.694987334 -6.291167088 cg09926027 9
87285693 q NTRK2 NTRK2 NA Body island
Body - island NA 0.126571521 0.116817221
-0.0097543 1.083500531
-0.021973307 0.804333548 -1.735085348 0.092450195
0.694987334 -6.293801288 cg25633045 17
28458728 q SLC6A4 CCDC55 NA Body open
sea Body - open sea NA 0.811886873 0.789913566
-0.021973307 1.027817356
0.007923684 0.922083243 1.735020927 0.092461765
0.694987334 -6.293906382 cg16128363 18 3880558
p DLGAP1 DLGAP1 NA TSS1500 shore TSS1500

- shore NA 0.919359477 0.927283161 0.007923684
0.991454947
0.006097418 0.125557293 1.722574433 0.09472021
0.707934992 -6.314150587 cg19070841 6
29691643 p GABBR1/MOG HLA-F NA Body island
Body - island NA 0.123461305 0.129558723
0.006097418 0.952937032
0.007505587 0.067264287 1.714941368 0.096128027
0.707934992 -6.32650603 cg10131972 22
19879787 q COMT TXNRD2 NA Body open
sea Body - open sea NA 0.064684242 0.072189828
0.007505587 0.896029867
-0.015287185 0.488899642 -1.708113027 0.097402251
0.707934992 -6.337520293 cg05812266 6
29595194 p GABBR1/MOG GABBR1 NA Body shore
Body - shore NA 0.494154612 0.478867426
-0.015287185 1.031923629
-0.008063641 0.920283431 -1.707422077 0.097531973
0.707934992 -6.338632778 cg13254553 6
29457282 p GABBR1 MAS1L 2739 IGR open
sea IGR - open sea NA 0.923055308 0.914991667
-0.008063641 1.008812803
-0.00949866 0.88155828 -1.707085265 0.09759526 0.707934992
-6.339174936 cg00806253 6 29571432 p
GABBR1/MOG GABBR1 NA Body open sea Body - open sea
V$TAXCREB_01 0.884823444 0.875324785 -
0.00949866 1.010851582
-0.0192051 0.836162506 -1.701121125 0.098721625
0.707934992 -6.348760535 cg18004110 6
29589729 p GABBR1/MOG GABBR1 NA Body open
sea Body - open sea NA 0.842764259 0.823559159
-0.0192051 1.023319636
-0.00998028 0.921951968 -1.700738508 0.098794255
0.707934992 -6.349374528 cg14847514 12
72340376 q TPH2 TPH2 NA Body open
sea Body - open sea NA 0.925382689 0.91540241 -
0.00998028 1.010902614
0.040274381 0.500166031 1.697180649 0.099471759
0.707934992 -6.355078373 cg20704602 6
29635371 p GABBR1/MOG MOG NA 3'UTR open
sea 3'UTR - open sea NA 0.486321713 0.526596094
0.040274381 0.923519408
0.019194356 0.600960838 1.69468236 0.099949813
0.707934992 -6.359077604 cg00504285 22
20019887 q COMT C22orf25 NA 5'UTR open
sea 5'UTR - open sea NA 0.594362778 0.613557134
0.019194356 0.96871627
0.006415479 0.136638078 1.694332895 0.100016836
0.707934992 -6.359636632 cg03402459 6
29521407 p GABBR1 UBD -1982 IGR island
IGR - island NA 0.134432757 0.140848236
0.006415479 0.954451123
0.006962997 0.902919628 1.691316848 0.100596845
0.707934992 -6.364457311 cg20592995 17
28524160 q SLC6A4 SLC6A4 NA 3'UTR open
sea 3'UTR - open sea NA 0.900526098 0.907489095
0.006962997 0.992327184

0.008490761 0.875156442 1.68721303 0.101390548
0.707934992 -6.371005115 cg04480313 6
29455322 p GABBR1 MAS1L NA 1stExon open
sea 1stExon - open sea NA 0.872237743 0.880728504
0.008490761 0.990359389
-0.017283732 0.911648296 -1.686433815 0.101541842
0.707934992 -6.372246884 cg25329933 6
29636669 p GABBR1/MOG MOG NA 3'UTR open
sea 3'UTR - open sea NA 0.917589579 0.900305847
-0.017283732 1.019197623
-0.013818086 0.517260554 -1.684015395 0.102012606
0.707934992 -6.376097863 cg04517749 6
29570507 p GABBR1/MOG GABBR1 NA 3'UTR open
sea 3'UTR - open sea V$OCT_C 0.522010521 0.508192434
-0.013818086 1.027190659
0.014401163 0.090958319 1.682961753 0.102218274
0.707934992 -6.37777419 cg15893431 21
34442183 q OLIG2 OLIG1 NA TSS1500 shore
TSS1500 - shore NA 0.086007919 0.100409082
0.014401163 0.856575095
-0.010224927 0.82621709 -1.680599145 0.102680706
0.707934992 -6.381529874 cg21552290 6
29578423 p GABBR1/MOG GABBR1 NA Body open
sea Body - open sea NA 0.829731909 0.819506981
-0.010224927 1.012476926
0.003152826 0.057466879 1.677543669 0.103281337
0.707934992 -6.386380423 cg15306595 2
171673272 q GAD1 GAD1 NA 5'UTR island
5'UTR - island NA 0.056383095 0.059535921
0.003152826 0.947043298
-0.01112893 0.88493469 -1.675467465 0.103691135
0.707934992 -6.38967217 cg19548470 18 3880510
p DLGAP1 DLGAP1 NA TSS1500 shore TSS1500
- shore NA 0.888760259 0.877631329 -0.01112893
1.012680643
0.005737821 0.028115092 1.673822559 0.104016764
0.707934992 -6.392277689 cg11032634 22
19929254 q COMT TXNRD2 NA 1stExon island
1stExon - island NA 0.026142716 0.031880537
0.005737821 0.820021193
0.005576715 0.029006934 1.670906633 0.1045961
0.708353783 -6.396891225 cg06570025 21
34444245 q OLIG2 OLIG1 NA 1stExon island
1stExon - island NA 0.027089938 0.032666653
0.005576715 0.829284163
0.013071081 0.720584484 1.662462806 0.106288876
0.715539321 -6.410212895 cg09545764 6
29715162 p MOG LOC285830 NA Body shore
Body - shore NA 0.7160913 0.729162381
0.013071081 0.982073841
-0.009170238 0.092914811 -1.659131412 0.106962969
0.715539321 -6.415453194 cg13013381 6
29600103 p GABBR1/MOG GABBR1 NA Body shore
Body - shore V$TAXCREB_02 0.09606708 0.086896842
-0.009170238 1.105530164
0.030800414 0.341484083 1.657834817 0.107226287
0.715539321 -6.417490359 cg23098068 2

172650722 q SLC25A12 SLC25A12 NA Body open
sea Body - open sea NA 0.330896441 0.361696854
0.030800414 0.914844675
0.00710695 0.926088835 1.653573695 0.108095447
0.717837723 -6.424175859 cg24628013 6
29580367 p GABBR1/MOG GABBR1 NA Body open
sea Body - open sea NA 0.923645821 0.930752771
0.00710695 0.992364299
-0.0337085 0.376926424 -1.645987144 0.109657367
0.724692167 -6.436042975 cg21644740 6
29599248 p GABBR1/MOG GABBR1 NA Body shore
Body - shore NA 0.388513721 0.354805221
-0.0337085 1.095005648
-0.026938583 0.313693447 -1.63756023 0.111414166
0.732762398 -6.449170719 cg04831505 12
72233240 q TPH2 TBC1D15 NA TSS1500 shore
TSS1500 - shore NA 0.322953585 0.296015002
-0.026938583 1.091004114
0.014348237 0.882220961 1.634451416 0.112068124
0.733536812 -6.453999388 cg05292788 6
29586060 p GABBR1/MOG GABBR1 NA Body open
sea Body - open sea NA 0.877288755 0.891636992
0.014348237 0.983907984
-0.043974504 0.363865998 -1.624398878 0.11420448
0.740699343 -6.469560123 cg06512249 6
29599390 p GABBR1/MOG GABBR1 NA Body shore
Body - shore NA 0.378982234 0.33500773 -
0.043974504 1.131264147
-0.013046378 0.860436338 -1.624208347 0.114245293
0.740699343 -6.469854269 cg19930203 22
19956281 q COMT COMT NA 3'UTR open
sea 3'UTR - open sea NA 0.86492103 0.851874652 -
0.013046378 1.015314903
0.046796542 0.417512256 1.619361315 0.115287628
0.743931488 -6.47732743 cg11768167 6
29690889 p GABBR1/MOG HLA-F NA TSS1500 shore
TSS1500 - shore NA 0.401425944 0.448222486
0.046796542 0.895595283
-0.005515703 0.044279822 -1.612104606 0.116862791
0.7458429 -6.488480465 cg05733135 11 27740876 p
BDNF BDNF NA Body island Body - island
NA 0.046175845 0.040660141 -0.005515703
1.135653834
0.078323572 0.249875448 1.610175294 0.11728454
0.7458429 -6.491438531 cg00274965 21 34405681 q
OLIG2 OLIG2 7465 IGR island IGR - island
NA 0.22295172 0.301275292 0.078323572
0.740026567
-0.003830281 0.048310834 -1.609388623 0.117456866
0.7458429 -6.49264381 cg23165623 6 152128411 q
ESR1 ESR1 NA TSS1500 shore TSS1500 - shore
NA 0.049627493 0.045797212 -0.003830281
1.083635681
0.00416655 0.02857652 1.60746259 0.117879657 0.7458429 -6.495592628
cg15568960 15 88790380 q NTRK3 NTRK3 NA
Body open sea Body - open sea NA 0.027144269
0.031310819 0.00416655 0.866929383

-0.008731475		0.928210137		-1.60550854		0.118309875
	0.7458429	-6.498581275		cg18773129	22		19938916	q
	COMT	COMT	NA	5'UTR	open sea	5'UTR - open sea
	NA	0.931211582		0.922480107		-0.008731475
	1.009465218
0.004399693		0.042371348		1.595479442		0.120538304
	0.746973014		-6.513871791		cg23952754	17
	28431834	q		SLC6A4	EFCAB5	NA		Body		open
sea		Body - open sea		NA		0.040858953		0.045258647
	0.004399693		0.90278777
-0.104272716		0.731492188		-1.593802225		0.120914315
	0.746973014		-6.516420947		cg11383134	6
	29648590	p		GABBR1/MOG	ZFP57	8421		IGR		open
sea		IGR - open sea		NA		0.767335934		0.663063218
	-0.104272716		1.157259087
-0.011819299		0.107660371		-1.589762296		0.121823965
	0.746973014		-6.522551752		cg01306985	6
	29720340	p		MOG		IFITM4P	NA		TSS1500	shore
	TSS1500 - shore		NA		0.111723255		0.099903956
	-0.011819299		1.118306616
0.009515749		0.055331156		1.589160008		0.121960058
	0.746973014		-6.52346462		cg01558660	9		4679986
	p		SLC1A1	CDC37L1	NA		Body		island	Body -
island	NA		0.052060117		0.061575866		0.009515749
	0.84546301
-0.010802677		0.916612721		-1.587243947		0.122393839
	0.746973014		-6.526366773		cg11441173	6
	29707887	p		MOG		LOC285830	NA		Body		open
sea		Body - open sea		NA		0.920326141		0.909523464
	-0.010802677		1.011877293
0.004851704		0.042301616		1.586873069		0.122477949
	0.746973014		-6.526928175		cg25457956	11
	27743664	p		BDNF		BDNF		NA		TSS200	island
	TSS200 - island		NA		0.040633842		0.045485547
	0.004851704		0.893335239
-0.015631858		0.548428911		-1.585442871		0.12280274
	0.746973014		-6.529092042		cg21481950	6
	29601498	p		GABBR1/MOG	GABBR1	NA		TSS1500	shore
	TSS1500 - shore		NA		0.553802362		0.538170504
	-0.015631858		1.029046293
-0.011457662		0.786560901		-1.582960702		0.123368102
	0.746973014		-6.532843571		cg09963892	15
	88496177	q		NTRK3		NTRK3		NA		Body		open
sea		Body - open sea		NA		0.790499472		0.77904181	-
0.011457662		1.014707378
-0.003992882		0.049476288		-1.582565705		0.123458267
	0.746973014		-6.533440105		cg18877200	6
	29720927	p		MOG		IFITM4P	2343		IGR		island
	IGR - island		NA		0.050848841		0.046855959
	-0.003992882		1.085216098
0.009025248		0.098468934		1.578684493		0.124347084
	0.746973014		-6.539294835		cg09205920	6
	29521506	p		GABBR1	UBD		-1883		IGR		island
	IGR - island		NA		0.095366505		0.104391753
	0.009025248		0.913544435
-0.027642747		0.834900185		-1.577685211		0.12457677
	0.746973014		-6.540800245		cg27390206	17

28585657 q SLC6A4 BLMH NA Body open
sea Body - open sea NA 0.84440238 0.816759632 -
0.027642747 1.03384441
-0.007330102 0.908930776 -1.574648221 0.125276952
0.746973014 -6.545370447 cg02945359 6
29694676 p GABBR1/MOG LOC285830 NA Body shelf
Body - shelf NA 0.911450499 0.904120397
-0.007330102 1.00810744
0.006232197 0.115420172 1.572921999 0.125676366
0.746973014 -6.547964792 cg03984780 11
27722617 p BDNF BDNF NA TSS200 island
TSS200 - island NA 0.113277854 0.119510051
0.006232197 0.94785211
-0.005053324 0.943791431 -1.567524005 0.126932067
0.746973014 -6.556061751 cg02117021 6
29425960 p GABBR1 OR2H1 NA TSS1500 open
sea TSS1500 - open sea NA 0.945528511 0.940475187
-0.005053324 1.00537316
-0.017463729 0.558722509 -1.565831916 0.127327788
0.746973014 -6.558594973 cg27642588 6
29709602 p MOG LOC285830 NA Body open
sea Body - open sea NA 0.564725666 0.547261937
-0.017463729 1.031911097
-0.010645819 0.769403546 -1.564356797 0.127673586
0.746973014 -6.560801458 cg21468949 6
29430334 p GABBR1 OR2H1 NA Body open
sea Body - open sea NA 0.773063046 0.762417227
-0.010645819 1.013963246
-0.013476615 0.777223316 -1.563938852 0.1277717
0.746973014 -6.561426297 cg12423733 6
29454623 p GABBR1 MAS1L NA 1stExon open
sea 1stExon - open sea NA 0.781855902 0.768379287
-0.013476615 1.017539014
0.010347154 0.110217347 1.55951393 0.128814238
0.749863307 -6.568032899 cg10810078 2
172543808 q SLC25A12 DYNC1I2 NA TSS200 shore
TSS200 - shore NA 0.106660513 0.117007668
0.010347154 0.911568573
0.004367275 0.047168273 1.548630109 0.131408012
0.757349717 -6.584214603 cg26232187 15
88799300 q NTRK3 NTRK3 NA Body shore
Body - shore NA 0.045667022 0.050034297
0.004367275 0.912714373
-0.003665847 0.031103302 -1.544479344 0.132408309
0.757349717 -6.590360185 cg22617773 6
29521751 p GABBR1 UBD -1638 IGR island
IGR - island NA 0.032363437 0.02869759 -
0.003665847 1.127740587
0.008870909 0.141286166 1.54351926 0.132640559
0.757349717 -6.591779658 cg14293300 21
34399361 q OLIG2 OLIG2 NA Body island
Body - island NA 0.138236791 0.1471077
0.008870909 0.939697861
0.00754963 0.039685507 1.543343995 0.132682992
0.757349717 -6.592038703 cg07159484 11
27722523 p BDNF BDNF NA Body island

Body - island NA 0.037090322 0.044639952
0.00754963 0.830877282
-0.024053916 0.869175397 -1.542578843 0.132868371
0.757349717 -6.593169316 cg27602828 18 3845348
p DLGAP1 DLGAP1 NA TSS200 open sea TSS200
- open sea NA 0.877443931 0.853390015 -0.024053916
1.028186311
0.005105015 0.07896628 1.535934637 0.134486961
0.757443372 -6.602966733 cg00465250 2
171627741 q GAD1 GAD1 -45459 IGR island
IGR - island NA 0.077211431 0.082316445
0.005105015 0.93798306
0.012276403 0.693158636 1.534250562 0.134899745
0.757443372 -6.605444253 cg03085859 6
29457094 p GABBR1 MAS1L NA TSS1500 open
sea TSS1500 - open sea NA 0.688938622 0.701215025
0.012276403 0.98249267
0.006633623 0.915812145 1.530643894 0.135787227
0.757443372 -6.61074231 cg09519060 6
29572346 p GABBR1/MOG GABBR1 NA Body open
sea Body - open sea NA 0.913531838 0.92016546
0.006633623 0.992790838
-0.003974723 0.042244782 -1.527316094 0.136610274
0.757443372 -6.615621187 cg05522774 21
34443443 q OLIG2 OLIG1 NA 1stExon island
1stExon - island NA 0.043611093 0.03963637 -
0.003974723 1.100279693
0.003853991 0.029142289 1.526803311 0.136737456
0.757443372 -6.616372161 cg09859456 12
72234313 q TPH2 TBC1D15 NA Body shore
Body - shore NA 0.027817479 0.03167147
0.003853991 0.878313479
-0.013404208 0.683165554 -1.526772333 0.136745142
0.757443372 -6.616417522 cg26129669 6
29593913 p GABBR1/MOG GABBR1 NA Body shore
Body - shore NA 0.687773251 0.674369043
-0.013404208 1.019876666
-0.039884284 0.333326559 -1.522897992 0.137709207
0.757443372 -6.622084395 cg25439807 18 3771151
p DLGAP1 DLGAP1 NA Body shore Body -
shore NA 0.347036781 0.307152497 -0.039884284
1.129851733
0.007485282 0.844967931 1.516723254 0.139257027
0.757443372 -6.631090283 cg10833114 12
72246108 q TPH2 MRS2P2 NA TSS1500 open
sea TSS1500 - open sea NA 0.842394865 0.849880147
0.007485282 0.991192544
-0.007957144 0.108275489 -1.51588557 0.139468086
0.757443372 -6.632309614 cg00287322 2
171670379 q GAD1 GAD1 -2821 IGR island
IGR - island NA 0.111010757 0.103053613
-0.007957144 1.077213634
0.0093143 0.858447745 1.514163631 0.139902749
0.757443372 -6.634814236 cg03049917 15
88752391 q NTRK3 NTRK3 NA Body open
sea Body - open sea NA 0.855245954 0.864560254
0.0093143 0.989226546

-0.01577234 0.841004441 -1.51274135 0.140262593
0.757443372 -6.636881137 cg13150094 6
29714824 p MOG LOC285830 NA Body shore
Body - shore NA 0.846426183 0.830653842
-0.01577234 1.018987863
-0.006991591 0.135262783 -1.510402606 0.140855928
0.757443372 -6.640276219 cg18676033 6
29691815 p GABBR1/MOG HLA-F NA Body island
Body - island NA 0.137666143 0.130674552
-0.006991591 1.053503845
-0.005579845 0.089676151 -1.50952523 0.141079037
0.757443372 -6.641548705 cg22839866 6
152128584 q ESR1 ESR1 NA TSS1500 shore
TSS1500 - shore NA 0.091594223 0.086014378
-0.005579845 1.064871073
0.003772439 0.97226683 1.507411907 0.141617607
0.757443372 -6.644611097 cg25663764 22
19965534 q COMT ARVCF NA Body shore
Body - shore NA 0.970970054 0.974742493
0.003772439 0.99612981
-0.015284001 0.553753333 -1.507224573 0.141665428
0.757443372 -6.644882382 cg07059469 6
152421432 q ESR1 ESR1 NA 3'UTR open
sea 3'UTR - open sea NA 0.559007209 0.543723207
-0.015284001 1.028109895
-0.01292153 0.858338682 -1.506917709 0.141743789
0.757443372 -6.645326698 cg04321753 6
29586923 p GABBR1/MOG GABBR1 NA Body open
sea Body - open sea NA 0.862780458 0.849858928
-0.01292153 1.015204323
0.014963549 0.153933457 1.503221124 0.1426905
0.758356547 -6.650672923 cg03861097 21
34393589 q OLIG2 OLIG2 -4627 IGR shore
IGR - shore NA 0.148789737 0.163753286
0.014963549 0.908621382
-0.013895667 0.29825267 -1.500581407 0.143369651
0.758356547 -6.654483677 cg14067066 6
29717475 p MOG LOC285830 NA TSS1500 shore
TSS1500 - shore NA 0.303029306 0.289133638
-0.013895667 1.048059673
-0.014490708 0.197563062 -1.498796482 0.14383035
0.758356547 -6.657057144 cg17014345 6
29716186 p MOG LOC285830 NA Body shore
Body - shore NA 0.202544243 0.188053535
-0.014490708 1.077056291
0.009058052 0.860231863 1.484327257 0.147608971
0.758356547 -6.677820393 cg21265702 6
152201605 q ESR1 ESR1 NA Body open
sea Body - open sea NA 0.857118157 0.866176209
0.009058052 0.989542483
0.010023438 0.173473803 1.480522189 0.148615755
0.758356547 -6.683251563 cg03363743 17
28562474 q SLC6A4 SLC6A4 NA 5'UTR island
5'UTR - island NA 0.170028246 0.180051684
0.010023438 0.944330218
0.015762224 0.386476791 1.48040908 0.148645766
0.758356547 -6.683412823 cg10216820 12

72332539 q TPH2 TPH2 NA TSS200 open
sea TSS200 - open sea NA 0.381058526 0.39682075
0.015762224 0.96027873
0.003836166 0.049968374 1.477684396 0.149370177
0.758356547 -6.687294193 cg05434863 6
29600206 p GABBR1/MOG GABBR1 NA 5'UTR island
5'UTR - island NA 0.048649692 0.052485858
0.003836166 0.926910483
-0.015107955 0.799807559 -1.472396535 0.150784113
0.758356547 -6.694809088 cg27521571 22
19938424 q COMT COMT NA 5'UTR open
sea 5'UTR - open sea NA 0.805000918 0.789892963
-0.015107955 1.019126585
0.018798046 0.766440021 1.470035122 0.151418982
0.758356547 -6.698157449 cg16403860 21
34394412 q OLIG2 OLIG2 -3804 IGR shore
IGR - shore NA 0.759978193 0.778776239
0.018798046 0.97586207
0.00977396 0.867945948 1.465208313 0.152723311
0.758356547 -6.70498703 cg08884395 6
152127887 q ESR1 ESR1 NA TSS1500 shore
TSS1500 - shore NA 0.864586149 0.874360109
0.00977396 0.988821585
0.0044456 0.063028404 1.464429991 0.15293447 0.758356547
-6.706086463 cg26852170 6 29720448 p MOG
IFITM4P 1864 IGR island IGR - island NA
0.061500229 0.065945828 0.0044456 0.932587108
-0.015587195 0.70207601 -1.462615487 0.153427649
0.758356547 -6.708647595 cg04335343 6
29597113 p GABBR1/MOG GABBR1 NA TSS1500 shore
TSS1500 - shore NA 0.707434108 0.691846913
-0.015587195 1.022529832
0.003404501 0.072567165 1.460545859 0.153991717
0.758356547 -6.711565442 cg11594927 6
29720600 p MOG IFITM4P 2016 IGR island
IGR - island NA 0.071396868 0.074801369
0.003404501 0.954486114
0.008506371 0.875132879 1.458398011 0.154578848
0.758356547 -6.71458975 cg01558785 6
29638162 p GABBR1/MOG MOG NA 3'UTR open
sea 3'UTR - open sea NA 0.872208814 0.880715184
0.008506371 0.99034152
-0.077760102 0.557890008 -1.455121433 0.155477958
0.758356547 -6.71919589 cg03198009 6
29648604 p GABBR1/MOG ZFP57 8435 IGR open
sea IGR - open sea NA 0.584620043 0.506859941
-0.077760102 1.153415363
0.006646376 0.127850558 1.454417543 0.15567165
0.758356547 -6.720184221 cg17781710 2
171671480 q GAD1 GAD1 -1720 IGR shore
IGR - shore NA 0.125565866 0.132212242
0.006646376 0.949729496
0.00498913 0.043832387 1.453989778 0.155789454
0.758356547 -6.720784641 cg06362065 18 3594098
p DLGAP1 DLGAP1 NA Body open sea Body -
open sea NA 0.042117374 0.047106503 0.00498913
0.894088317

0.017212945	0.789354824	1.452660517	0.156155977
0.758356547	-6.722649432	cg00433866	6
29623646	p	GABBR1/MOG	MOG	NA	TSS1500	open
sea	TSS1500 - open sea	NA	0.783437874	0.800650819
0.017212945	0.978501308
0.023130236	0.329945562	1.450575921	0.156732151
0.758356547	-6.725570863	cg09567915	6
29717260	p	MOG	LOC285830	NA	TSS1500	shore
TSS1500 - shore	NA	0.321994543	0.345124779
0.023130236	0.932980077
-0.030183702	0.602007601	-1.449122784	0.157134791
0.758356547	-6.727605172	cg22679406	6
29599331	p	GABBR1/MOG	GABBR1	NA	Body	shore
Body - shore	NA	0.612383248	0.582199546
-0.030183702	1.051844255
-0.007454076	0.900405179	-1.448617469	0.157274997
0.758356547	-6.728312165	cg25396488	6
29641118	p	GABBR1/MOG	ZFP57	NA	Body	open
sea	Body - open sea	NA	0.902967517	0.895513442
-0.007454076	1.0083238
0.039060427	0.408361974	1.447798092	0.157502556
0.758356547	-6.72945811	cg04836472	21
34405997	q	OLIG2	OLIG2	7781	IGR	island
IGR - island	NA	0.394934952	0.43399538
0.039060427	0.909998056
0.013873067	0.502571499	1.445450544	0.158155967
0.758356547	-6.732738141	cg05489989	6
29456162	p	GABBR1	MAS1L	NA	TSS1500	open
sea	TSS1500 - open sea	NA	0.497802632	0.511675699
0.013873067	0.972886993
-0.003163849	0.042700715	-1.439914799	0.159705292
0.758356547	-6.740454304	cg22305167	6
29521420	p	GABBR1	UBD	-1969	IGR	island
IGR - island	NA	0.043788288	0.04062444	-
0.003163849	1.077880409
-0.015600261	0.302176223	-1.438000362	0.160243888
0.758356547	-6.743116757	cg21185936	6
29716247	p	MOG	LOC285830	NA	Body	shore
Body - shore	NA	0.307538812	0.291938552
-0.015600261	1.053436793
-0.009715573	0.913956105	-1.43620115	0.160751378
0.758356547	-6.745616131	cg24962941	6
29577916	p	GABBR1/MOG	GABBR1	NA	Body	open
sea	Body - open sea	NA	0.917295833	0.907580261
-0.009715573	1.010704918
0.004245646	0.937819981	1.435852041	0.160849996
0.758356547	-6.746100778	cg05309760	6
152501942	q	ESR1	SYNE1	NA	Body	open
sea	Body - open sea	NA	0.93636054	0.940606186
0.004245646	0.995486266
-0.076930356	0.783985868	-1.435079011	0.161068536
0.758356547	-6.747173561	cg16885113	6
29648507	p	GABBR1/MOG	ZFP57	8338	IGR	open
sea	IGR - open sea	NA	0.810430677	0.733500322
-0.076930356	1.104881147
-0.009872691	0.906539787	-1.428635647	0.162899259
0.758356547	-6.756095684	cg04892672	6

29695164 p GABBR1/MOG LOC285830 NA Body shelf
Body - shelf NA 0.909933525 0.900060833
-0.009872691 1.010968916
-0.054521887 0.778329945 -1.425087684 0.163914325
0.758356547 -6.760993486 cg11747594 6
29648225 p GABBR1/MOG ZFP57 8056 IGR open
sea IGR - open sea NA 0.797071844 0.742549957
-0.054521887 1.073425211
0.004622148 0.060094946 1.424839193 0.163985604
0.758356547 -6.761336115 cg16535180 22
20008367 q COMT C22orf25 NA TSS1500 island
TSS1500 - island NA 0.058506083 0.063128231
0.004622148 0.926781601
-0.07903168 0.565492019 -1.423316637 0.164422883
0.758356547 -6.763434329 cg13835168 6
29648756 p GABBR1/MOG ZFP57 8587 IGR open
sea IGR - open sea NA 0.592659159 0.513627479
-0.07903168 1.153869649
0.00352579 0.035515561 1.422302113 0.164714765
0.758356547 -6.764831335 ch.15.1497565F 15
88581122 q NTRK3 NTRK3 NA Body open
sea Body - open sea NA 0.03430357 0.03782936 0.00352579
0.906797524
-0.024055005 0.190653263 -1.422079931 0.164778742
0.758356547 -6.765137165 cg17825311 6
29717188 p MOG LOC285830 NA TSS1500 shore
TSS1500 - shore NA 0.198922171 0.174867167
-0.024055005 1.137561581
-0.009322173 0.902538794 -1.421216771 0.165027474
0.758356547 -6.766324889 cg17242574 6
29625991 p GABBR1/MOG MOG NA Body open
sea Body - open sea NA 0.905743291 0.896421118
-0.009322173 1.010399323
-0.011915131 0.916386451 -1.420187849 0.16532436
0.758356547 -6.767739875 cg23148731 6
29697938 p MOG LOC285830 NA Body open
sea Body - open sea NA 0.920482277 0.908567146
-0.011915131 1.0131142
0.009250139 0.676991576 1.419011231 0.165664378
0.758356547 -6.769356869 cg07269000 6
29526234 p GABBR1/MOG UBD NA Body open
sea Body - open sea NA 0.673811841 0.68306198
0.009250139 0.986457834
0.00493465 0.092957087 1.417600372 0.166072814
0.758356547 -6.771294219 cg02736969 21
34442674 q OLIG2 OLIG1 NA 1stExon island
1stExon - island NA 0.091260801 0.096195451
0.00493465 0.948701836
0.011021283 0.87886136 1.416107252 0.166505928
0.758356547 -6.77334268 cg09298147 6
29550134 p GABBR1/MOG SNORD32B 105 IGR open
sea IGR - open sea NA 0.875072794 0.886094077
0.011021283 0.987561949
-0.004426848 0.063790783 -1.41344441 0.167280554
0.758356547 -6.776991202 cg14519950 9 4662766
p SLC1A1 C9orf68 NA Body island Body -

island	NA	0.065312511	0.060885664	-0.004426848
1.072707542
0.007023384	0.116790749	1.412197835	0.167644159
0.758356547	-6.77869713	cg18513624	22
19974307	q	COMT	ARVCF	NA	Body	island
Body - island	NA	0.114376461	0.121399845
0.007023384	0.942146681
-0.010224296	0.884868896	-1.411604049	0.167817575
0.758356547	-6.779509256	cg07463294	12
72243682	q	TPH2	TBC1D15	NA	Body	open
sea	Body - open sea	NA	0.888383498	0.878159202
-0.010224296	1.011642873
0.006223969	0.077333419	1.409406054	0.16846073
0.758356547	-6.782512849	cg04352676	6
29596516	p	GABBR1/MOG	GABBR1	NA	TSS1500	shore
TSS1500 - shore	NA	0.07519393	0.081417899
0.006223969	0.923555274
0.004051261	0.024898723	1.407466127	0.169029981
0.758356547	-6.78516036	cg14402472	6
29691165	p	GABBR1/MOG	HLA-F	NA	1stExon	island
1stExon - island	NA	0.023506102	0.027557363
0.004051261	0.852988074
0.00258982	0.029397286	1.40512005	0.169720432	0.758356547
-6.788357866	cg11582100	2	171673207	q	GAD1
GAD1	NA	5'UTR	island	5'UTR - island	NA
0.028507035	0.031096856	0.00258982	0.916717594
0.004372615	0.071016129	1.404298885	0.169962624
0.758356547	-6.789475932	cg27278787	18	3499813
p	DLGAP1	DLGAP1	NA	Body	shore	Body -
shore	NA	0.069513043	0.073885658	0.004372615
0.940819164
0.007722346	0.097947738	1.404238577	0.169980422
0.758356547	-6.789558024	cg01382110	18	4454189
p	DLGAP1	DLGAP1-AS5	189587	IGR	island	IGR -
island	NA	0.095293182	0.103015528	0.007722346
0.925037068
0.005620049	0.903971919	1.403850828	0.170094887
0.758356547	-6.790085747	cg25490334	6
152387590	q	ESR1	ESR1	NA	Body	open
sea	Body - open sea	NA	0.902040027	0.907660076
0.005620049	0.993808201
0.021822363	0.597209648	1.401917645	0.170666474
0.758356547	-6.792714876	cg05442477	21
34444584	q	OLIG2	OLIG1	NA	1stExon	shore
1stExon - shore	NA	0.589708211	0.611530574
0.021822363	0.964315173
-0.008124309	0.917020977	-1.395207893	0.17266206
0.758356547	-6.801815308	cg17706972	6
152126337	q	ESR1	ESR1	NA	5'UTR	shelf
5'UTR - shelf	NA	0.919813708	0.911689399
-0.008124309	1.008911268
-0.010158409	0.880612208	-1.394298332	0.172933979
0.758356547	-6.803045975	cg15803671	18	3457981
p	DLGAP1	TGIF1	NA	3'UTR	open sea	3'UTR -
open sea	NA	0.884104161	0.873945752	-0.010158409
1.011623615

0.008065616 0.84206227 1.39311452 0.17328839 0.758356547 -
6.804646651 cg26074662 22 19969472 q COMT ARVCF
NA Body shore Body - shore NA
0.839289715 0.84735533 0.008065616 0.990481425
-0.00691482 0.060017037 -1.393039149 0.173310974
0.758356547 -6.804748522 cg11116776 6
29691329 p GABBR1/MOG HLA-F NA Body island
Body - island NA 0.062394006 0.055479186
-0.00691482 1.124638094
-0.012704651 0.820583256 -1.392411965 0.173498991
0.758356547 -6.805596033 cg26718763 6
29573014 p GABBR1/MOG GABBR1 NA Body open
sea Body - open sea NA 0.82495048 0.812245829 -
0.012704651 1.015641387
-0.00357083 0.048191733 -1.391386738 0.173806677
0.758356547 -6.80698069 cg13773705 15
88799820 q NTRK3 NTRK3 NA TSS200 island
TSS200 - island NA 0.049419206 0.045848376
-0.00357083 1.077883457
0.012481876 0.727558384 1.390930134 0.173943848
0.758356547 -6.807597083 cg22906524 22
19960525 q COMT ARVCF NA Body island
Body - island V$MYCMAX_03;V$MYCMAX_02 0.723267739
0.735749616 0.012481876 0.983035157
-0.005731687 0.088175477 -1.390370384 0.174112121
0.758356547 -6.808352473 cg05661333 6
29600200 p GABBR1/MOG GABBR1 NA 5'UTR island
5'UTR - island NA 0.090145745 0.084414058
-0.005731687 1.067899674
-0.008143215 0.926776953 -1.39033077 0.174124034
0.758356547 -6.808405922 cg23908638 6
29579475 p GABBR1/MOG GABBR1 NA Body open
sea Body - open sea NA 0.929576183 0.921432968
-0.008143215 1.008837556
-0.019630605 0.429331617 -1.388326487 0.174727643
0.758356547 -6.811108455 cg22584138 17
28562220 q SLC6A4 SLC6A4 NA 5'UTR shore
5'UTR - shore NA 0.436079638 0.416449033
-0.019630605 1.047138073
-0.002606863 0.031617625 -1.386841461 0.175175928
0.758356547 -6.813108607 cg19783435 22
19974682 q COMT ARVCF NA Body island
Body - island NA 0.032513735 0.029906871
-0.002606863 1.087166056
0.005600622 0.901808306 1.383119209 0.176303514
0.760697588 -6.818113705 cg12083232 6
29571438 p GABBR1/MOG GABBR1 NA Body open
sea Body - open sea NA 0.899883092 0.905483714
0.005600622 0.993814773
0.00702861 0.900048355 1.378654138 0.177663595
0.760697588 -6.824101907 cg12728623 22
19938992 q COMT COMT NA 5'UTR open
sea 5'UTR - open sea NA 0.897632271 0.90466088
0.00702861 0.99223067
-0.011259965 0.831312742 -1.377780924 0.177930534
0.760697588 -6.825270984 cg22561592 15
88320395 q NTRK3 NTRK3-AS1 -99593 IGR open

sea IGR - open sea NA 0.835183355 0.82392339 -
0.011259965 1.013666277
0.012061865 0.451340695 1.377642722 0.177972811
0.760697588 -6.825455953 cg09577455 6
29593382 p GABBR1/MOG GABBR1 NA Body shore
Body - shore NA 0.447194429 0.459256294
0.012061865 0.973736092
-0.078976801 0.711764659 -1.375931205 0.178497022
0.760697588 -6.827745262 cg20228636 6
29648525 p GABBR1/MOG ZFP57 8356 IGR open
sea IGR - open sea NA 0.738912934 0.659936133
-0.078976801 1.1196734
0.005310579 0.937833577 1.363571317 0.182318464
0.774570369 -6.844202724 cg04419754 22
20044371 q COMT C22orf25 NA Body open
sea Body - open sea NA 0.936008065 0.941318644
0.005310579 0.994358362
0.003429453 0.041844168 1.359213634 0.183680836
0.775812289 -6.849973607 cg11308211 2
172750783 q SLC25A12 SLC25A12 NA 5'UTR island
5'UTR - island NA 0.040665293 0.044094746
0.003429453 0.92222536
-0.008472496 0.859388669 -1.359008967 0.183745016
0.775812289 -6.850244243 cg01066157 6
152451023 q ESR1 SYNE1 NA Body open
sea Body - open sea NA 0.86230109 0.853828594 -
0.008472496 1.009922947
0.043413526 0.619964088 1.355437655 0.184867723
0.778150909 -6.854960848 cg15841167 6
29633622 p GABBR1/MOG MOG NA 3'UTR open
sea 3'UTR - open sea NA 0.605040689 0.648454215
0.043413526 0.933050746
0.004953899 0.063269125 1.349974514 0.186595443
0.783014008 -6.862154597 cg01777019 6
152128805 q ESR1 ESR1 NA 5'UTR shore
5'UTR - shore NA 0.061566222 0.066520121
0.004953899 0.925527811
-0.010394302 0.680247859 -1.342846976 0.188868289
0.790127889 -6.871501056 cg21734356 18 3498854
p DLGAP1 DLGAP1 NA 3'UTR shore 3'UTR -
shore NA 0.683820901 0.673426598 -0.010394302
1.015434946
0.004504046 0.055043402 1.33818663 0.190365919
0.793965173 -6.87758838 cg24809935 6
29720530 p MOG IFITM4P 1946 IGR island
IGR - island NA 0.053495136 0.057999183
0.004504046 0.922342923
0.016446415 0.167087277 1.33392848 0.191742296
0.797274958 -6.883133836 cg18204321 18 3453798
p DLGAP1 TGIF1 NA 5'UTR shore 5'UTR -
shore NA 0.161433822 0.177880238 0.016446415
0.907542197
-0.034384909 0.495195863 -1.33169977 0.192465742
0.797857984 -6.886030026 cg15626350 6
152130207 q ESR1 ESR1 NA Body shore
Body - shore NA 0.507015676 0.472630767
-0.034384909 1.07275216

-0.005729343 0.082436643 -1.325398337 0.19452258
0.800497332 -6.894195255 cg10829693 21
34396221 q OLIG2 OLIG2 -1995 IGR island
IGR - island NA 0.084406104 0.078676762
-0.005729343 1.072821273
-0.00486447 0.924269129 -1.321196244 0.195903542
0.800497332 -6.899620957 cg12322235 6
29549724 p GABBR1/MOG SNORD32B NA TSS1500 open
sea TSS1500 - open sea NA 0.92594129 0.92107682 -0.00486447
1.005281286
0.003254407 0.025017516 1.321137116 0.195923027
0.800497332 -6.899697193 cg15818307 6
29521430 p GABBR1 UBD -1959 IGR island
IGR - island NA 0.023898814 0.027153221
0.003254407 0.880146558
0.005441585 0.950935121 1.319109749 0.196592032
0.800497332 -6.902309288 cg14053318 22
19967559 q COMT ARVCF NA Body island
Body - island V$NRSF_01 0.949064576 0.954506161
0.005441585 0.994299057
0.019169296 0.637150933 1.316764465 0.197368131
0.800497332 -6.90532651 cg17264271 6
152126938 q ESR1 ESR1 NA 5'UTR shore
5'UTR - shore NA 0.630561487 0.649730784
0.019169296 0.970496554
-0.003212748 0.030068604 -1.315840711 0.197674463
0.800497332 -6.906513604 cg05237001 6
29691245 p GABBR1/MOG HLA-F NA 1stExon island
1stExon - island NA 0.031172986 0.027960237
-0.003212748 1.114904212
0.040548856 0.288900458 1.314610453 0.198083002
0.800497332 -6.908093421 cg10915739 21
34405733 q OLIG2 OLIG2 7517 IGR island
IGR - island NA 0.274961788 0.315510645
0.040548856 0.87148181
-0.012907955 0.200499246 -1.314564807 0.198098173
0.800497332 -6.908152012 ch.6.2958553R 6
152519966 q ESR1 SYNE1 NA Body open
sea Body - open sea NA 0.204936355 0.1920284 -
0.012907955 1.067218989
-0.007761294 0.911498915 -1.313750801 0.198368856
0.800497332 -6.909196543 cg04324598 6
29602034 p GABBR1/MOG GABBR1 NA TSS1500 shore
TSS1500 - shore NA 0.91416686 0.906405566 -
0.007761294 1.008562717
-0.041441787 0.652893085 -1.306137978 0.200914103
0.808383803 -6.918937225 cg00495303 18 3771110
p DLGAP1 DLGAP1 NA Body shore Body -
shore NA 0.6671387 0.625696913 -0.041441787
1.066233005
0.029860478 0.467810011 1.304363319 0.201511011
0.808407808 -6.921200617 cg16118803 6
29629716 p GABBR1/MOG MOG NA Body open
sea Body - open sea NA 0.457545472 0.48740595
0.029860478 0.938735918
-0.010558178 0.093999323 -1.300343928 0.202867944
0.808937592 -6.92631671 cg26131286 6

29521783 p GABBR1 UBD -1606 IGR island
IGR - island NA 0.097628697 0.087070519
-0.010558178 1.121260079
0.019189743 0.14991892 1.298963292 0.203335648
0.808937592 -6.928070784 cg14730102 21
34401634 q OLIG2 OLIG2 3418 IGR shore
IGR - shore NA 0.143322446 0.162512189
0.019189743 0.881918131
-0.012722155 0.893216814 -1.295944236 0.204361249
0.808937592 -6.931900605 cg18389339 18 3730593
p DLGAP1 DLGAP1 NA Body shore Body -
shore NA 0.897590055 0.8848679 -0.012722155
1.014377462
-0.010567125 0.766735375 -1.293107851 0.205328383
0.808937592 -6.9354914 cg22319611 22 19840851 q
COMT GNB1L NA 5'UTR shore 5'UTR - shore
NA 0.770367824 0.759800699 -0.010567125
1.013907759
0.005370749 0.123444661 1.290661594 0.206165289
0.808937592 -6.938582617 cg00860808 2
171670500 q GAD1 GAD1 -2700 IGR island
IGR - island NA 0.121598466 0.126969214
0.005370749 0.957700392
-0.01294327 0.739111765 -1.286727082 0.207516796
0.808937592 -6.943543421 cg08415141 6
29598310 p GABBR1/MOG GABBR1 NA Body shore
Body - shore NA 0.743561014 0.730617745
-0.01294327 1.017715514
-0.07154322 0.694635953 -1.286057893 0.207747331
0.808937592 -6.944385806 cg24100841 6
29649024 p GABBR1/MOG ZFP57 8855 IGR open
sea IGR - open sea NA 0.719228935 0.647685715
-0.07154322 1.110459778
-0.007830221 0.351553909 -1.284325586 0.208345014
0.808937592 -6.946564628 cg06014588 15
88801339 q NTRK3 NTRK3-AS1 5378 IGR shore
IGR - shore NA 0.354245547 0.346415326
-0.007830221 1.022603564
-0.007747027 0.876603518 -1.282514592 0.208971241
0.808937592 -6.94883959 cg02547724 6
29455365 p GABBR1 MAS1L NA 1stExon open
sea 1stExon - open sea NA 0.879266559 0.871519532
-0.007747027 1.008889103
0.012163088 0.770886238 1.28242335 0.20900283 0.808937592
-6.948954131 cg10601943 6 29692824 p
GABBR1/MOG HLA-F NA Body shore Body - shore
NA 0.766705176 0.778868264 0.012163088
0.984383639
-0.008268721 0.889582702 -1.282091969 0.209117586
0.808937592 -6.94937007 cg08921491 15
88577641 q NTRK3 NTRK3 NA Body open
sea Body - open sea V$POU6F1_01 0.892425075
0.884156354 -0.008268721 1.009352103
0.016296229 0.250845722 1.278936947 0.210212561
0.808937592 -6.953325307 cg17741339 6
152085619 q ESR1 ESR1 NA 5'UTR open

sea	5'UTR - open sea	NA	0.245243894	0.261540123
	0.016296229	0.937691285
0.005876387	0.925931969	1.278791008	0.210263315
	0.808937592	-6.953508049	cg20408707	6
	29431410	p	GABBR1	OR2H1	NA	3'UTR	open
sea	3'UTR - open sea	NA	0.923911961	0.929788348
	0.005876387	0.993679866
0.004471772	0.111701713	1.278161295	0.210482421
	0.808937592	-6.954296344	cg14629287	6
	29716767	p	MOG	LOC285830	NA	Body	island
	Body - island	NA	0.110164541	0.114636313
	0.004471772	0.960991662
-0.0145875	0.781250196	-1.275847613	0.211288946
	0.808937592	-6.957189681	cg07529654	18	3447016
	p	DLGAP1	TGIF1	NA	5'UTR	shore	5'UTR -
shore	NA	0.786264649	0.771677149	-0.0145875
	1.018903631
0.018290217	0.250984808	1.275752308	0.211322218
	0.808937592	-6.957308762	cg07664000	17
	28659293	q	SLC6A4	TMIGD1	NA	5'UTR	open
sea	5'UTR - open sea	NA	0.244697546	0.262987762
	0.018290217	0.930452216
-0.005485516	0.062890883	-1.274683415	0.211695656
	0.808937592	-6.958643761	cg21108220	6
	29716658	p	MOG	LOC285830	NA	Body	island
	Body - island	NA	0.064776529	0.059291013
	-0.005485516	1.092518507
-0.010299457	0.897025843	-1.271158536	0.212930684
	0.809791372	-6.963039021	cg16650906	6
	29697700	p	MOG	LOC285830	NA	Body	open
sea	Body - open sea	NA	0.900566282	0.890266824
	-0.010299457	1.011568956
0.005890503	0.925716077	1.269116324	0.213648712
	0.809791372	-6.965580481	cg07584093	6
	152126180	q	ESR1	ESR1	NA	5'UTR	shelf
	5'UTR - shelf	NA	0.923691217	0.92958172
	0.005890503	0.993663276
0.005628273	0.047040211	1.268201044	0.213971112
	0.809791372	-6.966718316	cg26015683	6
	29720519	p	MOG	IFITM4P	1935	IGR	island
	IGR - island	NA	0.045105492	0.050733765
	0.005628273	0.88906258
0.003178303	0.0428638	1.266329287	0.214631568
	0.809791372	-6.969042892	cg04481212	11
	27740495	p	BDNF	BDNF	NA	Body	shore
	Body - shore	NA	0.041771258	0.044949561
	0.003178303	0.92929179
-0.008188893	0.885454511	-1.265629599	0.214878851
	0.809791372	-6.969911054	cg22115805	15
	88804912	q	NTRK3	NTRK3-AS1	8951	IGR	shelf
	IGR - shelf	NA	0.888269443	0.880080549
	-0.008188893	1.00930471
0.005102647	0.923403968	1.262895176	0.215847309
	0.809979574	-6.973299723	cg13654445	9
	87636383	q	NTRK2	NTRK2	NA	3'UTR	open
sea	3'UTR - open sea	NA	0.921649933	0.926752579
	0.005102647	0.994494058

-0.012499905	0.836467329	-1.262146703	0.216112971
0.809979574	-6.974226123	cg09842161 6
29598321	p	GABBR1/MOG GABBR1	NA	Body	shore
Body - shore	V$BRN2_01	0.840764171	0.828264266
-0.012499905	1.015091687
0.005688052	0.085016631	1.260409575	0.216730496
0.810074641	-6.976374288	cg05603292 6
29691631	p	GABBR1/MOG HLA-F	NA	Body	island
Body - island	NA	0.083061363	0.088749415
0.005688052	0.935908851
-0.011868748	0.804275512	-1.254234262	0.218936506
0.81535564 -6.983989123	cg17723549 17	28443042	q
SLC6A4	CCDC55	NA	TSS1500	shore	TSS1500 - shore
NA	0.808355394	0.796486647	-0.011868748
1.014901376
-0.002921581	0.062641398	-1.253122542	0.219335435
0.81535564 -6.985356401	cg00920970 6	152129388	q
ESR1	ESR1	NA	Body	island	Body - island
V$AP2_Q6;V$HEN1_02;V$E47_01	0.063645692	0.060724111
-0.002921581	1.048112372
0.008041943	0.112949365	1.247506626	0.221359009
0.815776484	-6.992246492	cg11251858 6
152129036 q	ESR1	ESR1	NA	5'UTR	island
5'UTR - island	NA	0.110184947	0.11822689
0.008041943	0.931978732
0.012386426	0.730907722	1.243950328	0.222647675
0.815776484	-6.996595164	cg08983330 6
29718869	p	MOG	IFITM4P	NA	Body	shore
Body - shore	NA	0.726649888	0.739036313
0.012386426	0.983239761
0.008657653	0.070518279	1.243642638	0.222759434
0.815776484	-6.996970882	cg05219421 6
29691003	p	GABBR1/MOG HLA-F	NA	TSS200	shore
TSS200 - shore	NA	0.06754221 0.076199863
0.008657653	0.886382302
0.010525532	0.095469422	1.242878843	0.223037041
0.815776484	-6.997903178	cg08179037 6
29705815	p	MOG	LOC285830 NA	Body	open
sea	Body - open sea	NA	0.09185127 0.102376802
0.010525532	0.89718831
-0.036906282	0.751447315	-1.240526153	0.223893774
0.815776484	-7.000771638	cg15708526 6
29648271	p	GABBR1/MOG ZFP57	8102	IGR	open
sea	IGR - open sea	NA	0.76413385 0.727227568	-
0.036906282	1.050749289
-0.007638562	0.881899239	-1.237886343	0.224857995
0.815776484	-7.00398429	cg05189570 11
27680480	p	BDNF	BDNF	NA	Body	open
sea	Body - open sea	NA	0.884524995	0.876886433
-0.007638562	1.008711005
-0.013282782	0.710080724	-1.23523963	0.225827855
0.815776484	-7.007199106	cg26272069 6
29591706	p	GABBR1/MOG GABBR1	NA	Body	shelf
Body - shelf	NA	0.71464668 0.701363898	-
0.013282782	1.018938503
-0.010592841	0.813059254	-1.232613961	0.226793092
0.815776484	-7.01038219	cg27474149 6

29572333 p GABBR1/MOG GABBR1 NA Body open
sea Body - open sea NA 0.816700543 0.806107702
-0.010592841 1.013140727
0.006799207 0.17147347 1.23182879 0.227082331 0.815776484
-7.011332853 cg27657867 11 27818018 p BDNF
BDNF-AS 141576 IGR open sea IGR - open sea NA
0.169136243 0.17593545 0.006799207 0.961353968
0.01266844 0.862465086 1.23155851 0.22718196 0.815776484 -
7.011659973 cg24058145 6 29523592 p GABBR1 UBD
NA 3'UTR shore 3'UTR - shore NA
0.85811031 0.87077875 0.01266844 0.985451597
-0.04976356 0.601857982 -1.228829049 0.228189906
0.815776484 -7.014959788 cg12463578 6
29644756 p GABBR1/MOG ZFP57 NA 1stExon open
sea 1stExon - open sea NA 0.618964205 0.569200646
-0.04976356 1.087427095
-0.003770665 0.082674637 -1.227686676 0.228612755
0.815776484 -7.016338899 cg22593533 21
34397654 q OLIG2 OLIG2 NA TSS1500 island
TSS1500 - island NA 0.083970803 0.080200139
-0.003770665 1.047015679
0.007560072 0.908374229 1.226711402 0.228974215
0.815776484 -7.017515359 cg02405128 6
29432580 p GABBR1 OR2H1 6350 IGR open
sea IGR - open sea NA 0.905775454 0.913335526
0.007560072 0.991722569
-0.005858301 0.919351887 -1.226385788 0.22909499
0.815776484 -7.017907954 cg01373089 6
29589631 p GABBR1/MOG GABBR1 NA Body open
sea Body - open sea NA 0.921365679 0.915507377
-0.005858301 1.006398968
-0.050941586 0.648296363 -1.224812215 0.229679323
0.815776484 -7.019803884 cg10648573 6
29648348 p GABBR1/MOG ZFP57 8179 IGR open
sea IGR - open sea NA 0.665807533 0.614865947
-0.050941586 1.082849906
-0.011413728 0.294204832 -1.223686009 0.230098211
0.815776484 -7.021159443 cg13524919 21
34396506 q OLIG2 OLIG2 -1710 IGR island
IGR - island NA 0.298128301 0.286714573
-0.011413728 1.039808678
0.006198184 0.919254149 1.221344784 0.230970842
0.815776484 -7.023973829 cg04847841 21
34351148 q OLIG2 OLIG2 -47068 IGR shore
IGR - shore NA 0.917123523 0.923321708
0.006198184 0.99328708
-0.076910532 0.717391526 -1.221154986 0.231041692
0.815776484 -7.024201771 cg03449857 6
29648623 p GABBR1/MOG ZFP57 8454 IGR open
sea IGR - open sea NA 0.743829522 0.666918989
-0.076910532 1.115322152
-0.078568634 0.712843608 -1.220612629 0.231244239
0.815776484 -7.024852947 cg15570656 6
29648628 p GABBR1/MOG ZFP57 8459 IGR open
sea IGR - open sea NA 0.739851576 0.661282941
-0.078568634 1.118812433

0.002497756 0.012654601 1.216144686 0.232917854
0.815776484 -7.030207319 cg14481263 22
20008608 q COMT C22orf25 NA TSS200 island
TSS200 - island NA 0.011795997 0.014293753
0.002497756 0.825255411
0.008077661 0.859875604 1.21113265 0.234805973
0.815776484 -7.036192448 cg18656132 6
29571363 p GABBR1/MOG GABBR1 NA Body open
sea Body - open sea NA 0.857098908 0.865176569
0.008077661 0.990663569
0.002776811 0.04050452 1.210630644 0.234995711
0.815776484 -7.036790678 cg10969051 6
29617771 p GABBR1/MOG MOG -6987 IGR island
IGR - island NA 0.039549991 0.042326802
0.002776811 0.934395918
-0.003665247 0.043864062 -1.204058249 0.237490314
0.815776484 -7.044602006 cg18344922 6
29595661 p GABBR1/MOG GABBR1 NA Body island
Body - island NA 0.04512399 0.041458743 -
0.003665247 1.088407094
0.008282476 0.872940339 1.201496891 0.238467787
0.815776484 -7.04763569 cg01976913 6
29430900 p GABBR1 OR2H1 NA 3'UTR open
sea 3'UTR - open sea NA 0.870093238 0.878375714
0.008282476 0.990570691
-0.012702012 0.827846017 -1.198425466 0.239643834
0.815776484 -7.051265724 cg24727561 6
29524112 p GABBR1 UBD NA Body shelf
Body - shelf NA 0.832212334 0.819510322
-0.012702012 1.015499514
0.005445479 0.927180204 1.196441959 0.240405591
0.815776484 -7.05360547 cg08907436 6
152125965 q ESR1 ESR1 NA 5'UTR shelf
5'UTR - shelf NA 0.92530832 0.9307538 0.005445479
0.994149387
-0.014604307 0.849034216 -1.196039633 0.240560321
0.815776484 -7.054079621 cg18977283 6
29579492 p GABBR1/MOG GABBR1 NA Body open
sea Body - open sea NA 0.854054447 0.83945014 -
0.014604307 1.017397468
-0.034480277 0.544623687 -1.19424155 0.241252739
0.815776484 -7.056196929 cg04063345 6
152130058 q ESR1 ESR1 NA Body shore
Body - shore NA 0.556476282 0.521996005
-0.034480277 1.066054676
0.025453051 0.738151413 1.191487819 0.242316013
0.815776484 -7.059433906 cg13763482 9 4663107
p SLC1A1 C9orf68 NA Body shore Body -
shore NA 0.729401927 0.754854978 0.025453051
0.966280873
-0.005215857 0.936129652 -1.189568929 0.243058977
0.815776484 -7.061685499 cg21372810 2
172542111 q SLC25A12 DYNC1I2 -1871 IGR shore
IGR - shore NA 0.937922603 0.932706745
-0.005215857 1.005592174
0.003431007 0.07884957 1.189258916 0.243179166
0.815776484 -7.062048951 cg09749751 6

29600125 p GABBR1/MOG GABBR1 NA Body shore
Body - shore NA 0.077670161 0.081101168
0.003431007 0.957694728
0.007627397 0.880768472 1.189086704 0.24324595
0.815776484 -7.062250812 cg08730070 22
19938378 q COMT COMT NA 5'UTR open
sea 5'UTR - open sea NA 0.878146554 0.885773951
0.007627397 0.991389003
-0.009038228 0.101963487 -1.18832767 0.243540466
0.815776484 -7.063140204 cg14531834 9
87655988 q NTRK2 NTRK2 371362 IGR open
sea IGR - open sea NA 0.105070378 0.09603215 -
0.009038228 1.094116689
-0.022300689 0.600476962 -1.186997957 0.244057045
0.815776484 -7.064697032 cg00504902 6
29692183 p GABBR1/MOG HLA-F NA Body shore
Body - shore NA 0.608142824 0.585842135
-0.022300689 1.038066038
-0.009605935 0.867822992 -1.18569533 0.244563883
0.815776484 -7.066220603 cg21645752 6
29598695 p GABBR1/MOG GABBR1 NA Body shore
Body - shore NA 0.871125032 0.861519097
-0.009605935 1.011149997
-0.007685426 0.900081276 -1.183435355 0.24544505
0.815776484 -7.068860269 cg18950940 6
29580896 p GABBR1/MOG GABBR1 NA Body open
sea Body - open sea NA 0.902723141 0.895037714
-0.007685426 1.008586707
0.004246851 0.069460081 1.182959898 0.245630728
0.815776484 -7.06941502 cg14075496 9 4679516
p SLC1A1 CDC37L1 NA TSS200 island TSS200
- island NA 0.068000226 0.072247077 0.004246851
0.941217677
0.002734222 0.036290337 1.182787571 0.245698052
0.815776484 -7.069616035 cg14337085 12
72233324 q TPH2 TBC1D15 NA TSS200 island
TSS200 - island NA 0.035350448 0.03808467
0.002734222 0.928206756
-0.011226097 0.88685176 -1.180606919 0.246551146
0.815776484 -7.072157408 cg24034959 6
29524905 p GABBR1/MOG UBD NA Body shelf
Body - shelf NA 0.89071073 0.879484634 -
0.011226097 1.012764403
-0.004765342 0.917272903 -1.180561188 0.24656906
0.815776484 -7.072210658 cg07511633 6
29455331 p GABBR1 MAS1L NA 1stExon open
sea 1stExon - open sea NA 0.91891099 0.914145647 -
0.004765342 1.005212893
0.015747779 0.751041071 1.179913189 0.246822996
0.815776484 -7.072964993 cg21157690 6
152126895 q ESR1 ESR1 NA 5'UTR shore
5'UTR - shore NA 0.745627772 0.76137555
0.015747779 0.979316675
-0.009043625 0.870701626 -1.178720087 0.247291049
0.815776484 -7.074352892 cg13730341 6
29589573 p GABBR1/MOG GABBR1 NA Body open

sea Body - open sea V$HSF2_01 0.873810372 0.864766747
-0.009043625 1.010457878
-0.003171756 0.060135369 -1.177200485 0.247888129
0.815776484 -7.076118736 cg03760316 18 3594197
p DLGAP1 DLGAP1 NA Body open sea Body -
open sea NA 0.06122566 0.058053904 -0.003171756
1.054634672
-0.005434469 0.885477949 -1.175162704 0.24869047
0.815776484 -7.07848345 cg17085250 6
29591016 p GABBR1/MOG GABBR1 NA Body open
sea Body - open sea NA 0.887346047 0.881911578
-0.005434469 1.006162147
-0.021525268 0.351113694 -1.173870529 0.249200227
0.815776484 -7.079980992 cg07420274 2
171676306 q GAD1 GAD1 NA Body shore
Body - shore NA 0.358513005 0.336987737
-0.021525268 1.063875523
0.013563574 0.7844138 1.173363785 0.249400343
0.815776484 -7.080567861 cg27241909 6
29714043 p MOG LOC285830 NA Body shelf
Body - shelf NA 0.779751321 0.793314896
0.013563574 0.982902659
-0.004609825 0.929670828 -1.17143807 0.250161895
0.815776484 -7.082795946 cg04718263 6
29554942 p GABBR1/MOG OR2H2 NA TSS1500 open
sea TSS1500 - open sea NA 0.931255455 0.92664563 -
0.004609825 1.004974744
0.015582074 0.877989912 1.170937541 0.250360115
0.815776484 -7.083374518 cg10600786 6
29719569 p MOG IFITM4P NA TSS1500 shore
TSS1500 - shore NA 0.872633574 0.888215648
0.015582074 0.98245688
0.020842893 0.197771097 1.170867206 0.250387978
0.815776484 -7.083455801 cg02613510 11
27723789 p BDNF BDNF NA Body shore
Body - shore NA 0.190606353 0.211449246
0.020842893 0.901428388
-0.008503261 0.054659477 -1.168229172 0.251434678
0.815776484 -7.086501242 cg07016276 6
29692009 p GABBR1/MOG HLA-F NA Body island
Body - island NA 0.057582473 0.049079212
-0.008503261 1.173255858
0.016586991 0.830982707 1.166738074 0.252027719
0.815776484 -7.088219832 cg06877423 6
152200760 q ESR1 ESR1 NA Body open
sea Body - open sea NA 0.825280929 0.841867919
0.016586991 0.980297396
-0.085871562 0.709598195 -1.164924068 0.252750564
0.815776484 -7.090307881 cg07134666 6
29648400 p GABBR1/MOG ZFP57 8231 IGR open
sea IGR - open sea NA 0.739116544 0.653244982
-0.085871562 1.131453841
0.005094415 0.113117724 1.163872283 0.253170371
0.815776484 -7.091517193 cg27217194 6
29600139 p GABBR1/MOG GABBR1 NA Body shore
Body - shore NA 0.111366519 0.116460934
0.005094415 0.956256447

0.025863161 0.709693773 1.161378417 0.254167802
0.815776484 -7.094380562 cg15628633 6
29706377 p MOG LOC285830 NA Body open
sea Body - open sea NA 0.700803311 0.726666472
0.025863161 0.964408485
-0.007533188 0.487088541 -1.159749744 0.254820741
0.815776484 -7.096247503 cg07839627 6
29599836 p GABBR1/MOG GABBR1 NA Body shore
Body - shore NA 0.489678074 0.482144886
-0.007533188 1.015624324
-0.002413605 0.016970317 -1.156678315 0.256055408
0.815776484 -7.09976172 cg00845219 22
19842449 q COMT GNB1L NA 1stExon island
1stExon - island NA 0.017799994 0.015386389
-0.002413605 1.156866241
0.004920258 0.055712629 1.153901113 0.257175544
0.815776484 -7.102931922 cg19549714 18 3447713
p DLGAP1 TGIF1 NA 5'UTR shore 5'UTR -
shore NA 0.05402129 0.058941549 0.004920258
0.91652308
-0.004256843 0.936568237 -1.15280105 0.25762022
0.815776484 -7.104185717 cg25758242 6
29427011 p GABBR1 OR2H1 NA 5'UTR open
sea 5'UTR - open sea NA 0.938031527 0.933774684
-0.004256843 1.004558747
-0.003263356 0.057257253 -1.151990009 0.257948422
0.815776484 -7.105109396 cg05737526 18 3454175
p DLGAP1 TGIF1 NA 5'UTR shore 5'UTR -
shore NA 0.058379031 0.055115675 -0.003263356
1.059209218
0.003232649 0.041094916 1.151884449 0.257991162
0.815776484 -7.105229573 cg11241206 11
27723128 p BDNF BDNF NA TSS1500 shore
TSS1500 - shore NA 0.039983693 0.043216342
0.003232649 0.925198458
0.004900165 0.105723844 1.150807739 0.258427394
0.815776484 -7.10645479 cg22377998 22
20004881 q COMT ARVCF NA TSS1500 shore
TSS1500 - shore NA 0.104039412 0.108939577
0.004900165 0.955019423
0.004070518 0.056572269 1.149040287 0.259144643
0.815776484 -7.108463737 cg13504245 9
87282610 q NTRK2 NTRK2 NA TSS1500 shore
TSS1500 - shore NA 0.055173029 0.059243546
0.004070518 0.931291807
-0.015306346 0.811972093 -1.146835305 0.260041471
0.815776484 -7.110966013 cg05866854 2
171784945 q GAD1 GORASP2 NA TSS1500 shore
TSS1500 - shore NA 0.81723365 0.801927304 -
0.015306346 1.01908695
0.010319814 0.724992537 1.144511315 0.260989137
0.815776484 -7.113598555 cg23270523 18 4450489
p DLGAP1 DLGAP1-AS5 185887 IGR shelf IGR -
shelf NA 0.721445101 0.731764916 0.010319814
0.985897363
0.010466584 0.65069853 1.143969096 0.261210601
0.815776484 -7.114212057 cg09072576 22

19838287	q	COMT	GNB1L	NA	5'UTR	shelf
5'UTR - shelf	NA	0.647100641	0.657567226
0.010466584	0.984082867
-0.036673253	0.812773489	-1.143635622	0.261346872
0.815776484	-7.114589238	cg25699073 6
29648381	p	GABBR1/MOG ZFP57	8212	IGR	open
sea	IGR - open sea	NA	0.82537992 0.788706667	-
0.036673253	1.046497962
-0.006032681	0.100861866	-1.142534418	0.261797235
0.815776484	-7.115834053	cg10217445 21
34397784	q	OLIG2	OLIG2	NA	TSS1500	island
TSS1500 - island	NA	0.1029356	0.096902919	-
0.006032681	1.062254895
0.007720444	0.184212375	1.140420082	0.262663519
0.815776484	-7.118221031	cg18584905 17
28563300	q	SLC6A4	SLC6A4	NA	TSS1500	shore
TSS1500 - shore	NA	0.181558473	0.189278917
0.007720444	0.959211284
-0.003973342	0.945029666	-1.138945016	0.263269107
0.815776484	-7.119883893	cg03467156 6
29425910	p	GABBR1	OR2H1	NA	TSS1500	open
sea	TSS1500 - open sea	NA	0.946395503	0.942422161
-0.003973342	1.004216096
0.002259253	0.029837283	1.138251805	0.263554054
0.815776484	-7.120664675	cg16639998 2
171626995 q	GAD1	GAD1	-46205	IGR	shore
IGR - shore	NA	0.029060665	0.031319918
0.002259253	0.927865296
0.010263634	0.761309816	1.135248874	0.264790993
0.815776484	-7.124041896	cg12833048 6
29639582	p	GABBR1/MOG MOG	NA	3'UTR	open
sea	3'UTR - open sea	NA	0.757781692	0.768045326
0.010263634	0.986636682
-0.004708974	0.95221133 -1.133795044	0.265391345
0.815776484	-7.12567398	cg16448399 18	3880076
p	DLGAP1	DLGAP1	NA	1stExon	island	1stExon
- island	NA	0.953830039	0.949121066	-0.004708974
1.004961404
0.007222977	0.821681454	1.132159974	0.266067713
0.815776484	-7.127507222	cg13537510 6
29623721	p	GABBR1/MOG MOG	NA	TSS1500	open
sea	TSS1500 - open sea	NA	0.819198556	0.826421533
0.007222977	0.991259936
0.004198493	0.077043625	1.132060219	0.266109018
0.815776484	-7.127618988	cg12635048 6
29720957	p	MOG	IFITM4P	2373	IGR	island
IGR - island	NA	0.075600393	0.079798886
0.004198493	0.947386571
-0.004595914	0.926544847	-1.128891521	0.267423474
0.815776484	-7.131164502	cg04066686 6
29580347	p	GABBR1/MOG GABBR1	NA	Body	open
sea	Body - open sea	NA	0.928124693	0.923528778
-0.004595914	1.004976472
-0.011637853	0.861612793	-1.127475223	0.268012501
0.815776484	-7.132746258	cg07379508 2
171630114 q	GAD1	GAD1	-43086	IGR	shelf

IGR - shelf NA 0.865613305 0.853975452
-0.011637853 1.013627854
0.007652417 0.851585743 1.125332186 0.268905547
0.815776484 -7.135136171 cg22965752 6
29425885 p GABBR1 OR2H1 NA TSS1500 open
sea TSS1500 - open sea NA 0.848955225 0.856607641
0.007652417 0.991066603
0.004940948 0.111652902 1.1250347 0.269029685
0.815776484 -7.135467596 cg27193031 11
27721088 p BDNF BDNF NA Body shore
Body - shore NA 0.109954451 0.114895399
0.004940948 0.95699612
0.01321196 0.142085942 1.124520719 0.26924426 0.815776484
-7.136040023 cg17369088 17 28444068 q SLC6A4
MIR423 NA TSS200 shore TSS200 - shore NA
0.137544331 0.150756291 0.01321196 0.912362132
-0.011879745 0.889178288 -1.124372109 0.269306325
0.815776484 -7.136205487 cg25252977 6
29696650 p MOG LOC285830 NA Body open
sea Body - open sea NA 0.89326195 0.881382206 -
0.011879745 1.013478539
-0.005868116 0.84803005 -1.123647638 0.269609034
0.815776484 -7.137011832 cg14377523 6
29572373 p GABBR1/MOG GABBR1 NA Body open
sea Body - open sea NA 0.850047215 0.844179099
-0.005868116 1.006951269
0.026389336 0.409848397 1.122000587 0.27029814
0.815776484 -7.138843233 cg11935738 6
29520752 p GABBR1 UBD -2637 IGR shore
IGR - shore NA 0.400777063 0.427166399
0.026389336 0.938222351
0.013224307 0.642093727 1.118292055 0.271854373
0.815776484 -7.142957768 cg25385819 18 3445098
p DLGAP1 TGIF1 NA 5'UTR shelf 5'UTR -
shelf NA 0.637547872 0.650772179 0.013224307
0.979679053
0.024551625 0.522670647 1.115898858 0.272862052
0.815776484 -7.145606287 cg05813221 6
29629988 p GABBR1/MOG MOG NA Body open
sea Body - open sea NA 0.514231026 0.538782651
0.024551625 0.9544313
0.002260297 0.024542508 1.115349453 0.273093761
0.815776484 -7.146213565 cg18094551 22
20004364 q COMT ARVCF NA TSS200 island
TSS200 - island NA 0.023765531 0.026025827
0.002260297 0.913151809
0.002361118 0.059865377 1.114811679 0.273320702
0.815776484 -7.14680772 cg21930443 17
28443747 q SLC6A4 CCDC55 NA TSS200 island
TSS200 - island NA 0.059053743 0.061414861
0.002361118 0.961554615
-0.055021783 0.64147296 -1.113763086 0.273763597
0.815776484 -7.147965488 cg00588198 6
29648452 p GABBR1/MOG ZFP57 8283 IGR open
sea IGR - open sea NA 0.660386697 0.605364915
-0.055021783 1.090890272

-0.007857685 0.084688776 -1.113047605 0.274066091
0.815776484 -7.148754883 cg19539318 2
172544773 q SLC25A12 DYNC1I2 NA 5'UTR shore
5'UTR - shore NA 0.087389855 0.07953217 -
0.007857685 1.098798826
0.003480528 0.062446522 1.112142188 0.274449229
0.815776484 -7.149753164 cg21834061 6
29521162 p GABBR1 UBD -2227 IGR island
IGR - island NA 0.061250091 0.064730618
0.003480528 0.946230592
0.002251386 0.052659796 1.109477472 0.275579059
0.815776484 -7.152686826 cg09492354 11
27720709 p BDNF BDNF NA Body shore
Body - shore NA 0.051885882 0.054137267
0.002251386 0.958413398
0.003883546 0.065690916 1.108292053 0.27608274
0.815776484 -7.153989796 cg06860277 22
19930072 q COMT COMT NA 5'UTR shore
5'UTR - shore NA 0.064355947 0.068239493
0.003883546 0.943089466
0.007187646 0.766547401 1.108095692 0.276166236
0.815776484 -7.154205504 cg13040666 6
29693534 p GABBR1/MOG HLA-F NA Body shore
Body - shore NA 0.764076647 0.771264293
0.007187646 0.990680697
0.005752428 0.913890652 1.107937768 0.276233401
0.815776484 -7.154378962 cg00886856 22
19960296 q COMT ARVCF NA Body shore
Body - shore NA 0.911913255 0.917665683
0.005752428 0.993731456
0.006595003 0.06129208 1.106389186 0.276892637
0.815776484 -7.156078663 ch.6.2949012F 6
152044517 q ESR1 ESR1 NA 5'UTR open
sea 5'UTR - open sea NA 0.059025047 0.06562005
0.006595003 0.899497135
-0.005692772 0.892073113 -1.105512098 0.277266513
0.815776484 -7.157040365 cg25693099 18 3879303
p DLGAP1 DLGAP1 NA 1stExon island 1stExon
- island NA 0.894030004 0.888337232 -0.005692772
1.006408346
-0.054253916 0.810333513 -1.105016287 0.277478022
0.815776484 -7.157583696 cg22494932 6
29648379 p GABBR1/MOG ZFP57 8210 IGR open
sea IGR - open sea NA 0.828983296 0.774729381
-0.054253916 1.070029505
-0.0043154 0.04955826 -1.103409352 0.278164318 0.815776484
-7.159343093 cg10022526 11 27744557 p BDNF
BDNF NA TSS1500 island TSS1500 - island NA
0.051041679 0.046726279 -0.0043154 1.092354882
-0.046671215 0.477382235 -1.103064136 0.278311912
0.815776484 -7.159720753 cg08041448 6
29648901 p GABBR1/MOG ZFP57 8732 IGR open
sea IGR - open sea NA 0.493425465 0.44675425 -
0.046671215 1.104467311
-0.004531701 0.939002025 -1.102438363 0.278579599
0.815776484 -7.160405058 cg04852097 6
29431228 p GABBR1 OR2H1 NA 3'UTR open

sea 3'UTR - open sea NA 0.940559797 0.936028096
-0.004531701 1.004841416
0.004709887 0.066711795 1.100420653 0.279443962
0.815776484 -7.162609047 cg14692377 17
28562685 q SLC6A4 SLC6A4 NA 1stExon island
1stExon - island NA 0.065092772 0.069802659
0.004709887 0.932525679
0.002140079 0.025457759 1.099875664 0.279677757
0.815776484 -7.16320371 cg16044251 6
29521695 p GABBR1 UBD -1694 IGR island
IGR - island NA 0.024722106 0.026862186
0.002140079 0.92033113
0.017361084 0.464795515 1.0941911 0.28212468 0.819476405
-7.16939012 cg23606396 6 29717917 p MOG
LOC285830 NA TSS1500 shore TSS1500 - shore NA
0.458827642 0.476188726 0.017361084
0.96354159
0.005043904 0.066275201 1.09409769 0.282165015
0.819476405 -7.169491528 cg15753746 2
171679591 q GAD1 GAD1 NA Body island
Body - island NA 0.06454136 0.069585263
0.005043904 0.927514781
-0.004585887 0.929626677 -1.09275946 0.282743321
0.819476405 -7.170943464 cg18761756 18 3732002
p DLGAP1 DLGAP1 NA Body shelf Body -
shelf NA 0.931203075 0.926617188 -0.004585887
1.004949063
0.004964862 0.079285262 1.089727217 0.284056797
0.81980328 -7.174227253 cg00655307 6 152128743 q
ESR1 ESR1 NA 5'UTR shore 5'UTR - shore
NA 0.07757859 0.082543452 0.004964862
0.939851534
0.005554161 0.091594588 1.086304046 0.285544804
0.81980328 -7.177924228 cg12296326 6 29717114 p
MOG LOC285830 NA TSS1500 island TSS1500 - island
NA 0.089685346 0.095239506 0.005554161
0.941682184
0.00459634 0.881873609 1.083805707 0.286634279
0.81980328 -7.180615582 cg20041152 15 88795689 q
NTRK3 NTRK3 NA Body shelf Body - shelf
NA 0.880293617 0.884889957 0.00459634
0.99480575
-0.005580776 0.1291958 -1.083740265 0.286662857
0.81980328 -7.180686003 cg20997792 6 29595491 p
GABBR1/MOG GABBR1 NA Body island Body - island
NA 0.131114191 0.125533415 -0.005580776
1.044456498
0.002906354 0.048824074 1.083686146 0.286686491
0.81980328 -7.180744237 cg23918507 18 3771380 p
DLGAP1 DLGAP1 NA Body island Body - island
NA 0.047825014 0.050731369 0.002906354
0.94271089
-0.001501564 0.014402283 -1.082176756 0.287346214
0.81980328 -7.182367284 cg18587988 22 20008614 q
COMT C22orf25 NA TSS200 island TSS200 - island
NA 0.014918445 0.013416881 -0.001501564
1.111916026

0.007156987 0.889588192 1.081788154 0.287516238
0.81980328 -7.182784809 cg18281744 6 29455512 p
GABBR1 MAS1L NA 1stExon open sea 1stExon - open
sea NA 0.887127977 0.894284964 0.007156987
0.991996973
0.005590872 0.906397788 1.08148192 0.287650274
0.81980328 -7.183113736 cg05972518 6 29577348 p
GABBR1/MOG GABBR1 NA Body open sea Body - open sea
NA 0.904475926 0.910066798 0.005590872
0.993856636
-0.031581263 0.659227742 -1.078603707 0.288912197
0.821687912 -7.186201013 cg12644888 6
29648360 p GABBR1/MOG ZFP57 8191 IGR open
sea IGR - open sea NA 0.670083802 0.638502538
-0.031581263 1.049461454
0.00245912 0.022302668 1.076231005 0.28995542 0.822944013
-7.188740312 cg09742688 2 171672899 q GAD1
GAD1 NA TSS1500 island TSS1500 - island NA
0.021457346 0.023916466 0.00245912 0.897178789
0.010472803 0.095295318 1.072597709 0.291558042
0.824650801 -7.192618645 cg24703717 6
29691168 p GABBR1/MOG HLA-F NA 1stExon island
1stExon - island NA 0.091695292 0.102168095
0.010472803 0.897494389
0.005170953 0.084531665 1.071415779 0.292080726
0.824650801 -7.193877658 cg24611631 9 4490288
p SLC1A1 SLC1A1 NA TSS200 island TSS200
- island NA 0.08275415 0.087925103 0.005170953
0.941189116
0.001942493 0.036995512 1.070773058 0.292365233
0.824650801 -7.194561754 cg18518183 2
172544446 q SLC25A12 DYNC1I2 NA 5'UTR island
5'UTR - island NA 0.03632778 0.038270273
0.001942493 0.949242771
0.014999279 0.345139293 1.068374111 0.293428872
0.825947937 -7.197111761 cg18704527 22
19962042 q COMT ARVCF NA Body shore
Body - shore NA 0.33998329 0.354982569
0.014999279 0.957746435
-0.017720239 0.793514648 -1.065755488 0.294593014
0.827522059 -7.199889203 cg11587584 6
29692372 p GABBR1/MOG HLA-F NA Body shore
Body - shore NA 0.79960598 0.781885741 -
0.017720239 1.022663464
-0.018896175 0.245356844 -1.063104122 0.295775012
0.827523764 -7.202694909 cg06297863 6
29626990 p GABBR1/MOG MOG NA Body open
sea Body - open sea NA 0.251852404 0.232956229
-0.018896175 1.081114702
0.00670263 0.849556061 1.063040423 0.295803451
0.827523764 -7.202762236 cg06760467 6
29496514 p GABBR1 LINC01015 -669 IGR open
sea IGR - open sea NA 0.847252032 0.853954662
0.00670263 0.99215107
-0.013474389 0.843933015 -1.061634761 0.296431496
0.827588338 -7.204247003 cg25804443 18 3875823
p DLGAP1 DLGAP1 NA Body shelf Body -

shelf	NA	0.848564836	0.835090447	-0.013474389
1.016135245
-0.009604439	0.201106038	-1.054743262	0.299524116
0.832971856	-7.211499845	cg26142965 6
29521803	p	GABBR1	UBD	-1586	IGR	island
IGR - island	NA	0.204407564	0.194803124
-0.009604439	1.049303316
-0.00857242	0.875314126	-1.05067128	0.301362019
0.832971856	-7.215764641	cg13680362 6
29426320	p	GABBR1	OR2H1	NA	1stExon	open
sea	1stExon - open sea	NA	0.878260895	0.869688475
-0.00857242	1.009856886
-0.001702214	0.015209363	-1.050095334	0.301622608
0.832971856	-7.216366618	cg24710870 6
29720670	p	MOG	IFITM4P	2086	IGR	island
IGR - island	NA	0.015794499	0.014092284
-0.001702214	1.120790569
0.005183423	0.069200095	1.045414897	0.30374612
0.832971856	-7.221247173	cg13663738 21
34392756	q	OLIG2	OLIG2	-5460	IGR	shore
IGR - shore	NA	0.067418293	0.072601716
0.005183423	0.928604677
-0.011494932	0.859958461	-1.043947256	0.304414127
0.832971856	-7.222773375	cg18371410 6
29699406	p	MOG	LOC285830 NA	Body	open
sea	Body - open sea	NA	0.863909844	0.852414912
-0.011494932	1.013485137
0.009506995	0.728880885	1.042809974	0.304932471
0.832971856	-7.22395466	cg17728820 6
29709366	p	MOG	LOC285830 NA	Body	open
sea	Body - open sea	NA	0.725612855	0.73511985
0.009506995	0.987067422
0.002210582	0.044319889	1.04218816 0.305216137
0.832971856	-7.224600024	cg09805507 6
29691426	p	GABBR1/MOG HLA-F	NA	Body	island
Body - island	NA	0.043560001	0.045770583
0.002210582	0.951702997
-0.007883324	0.872249359	-1.040281815	0.306086939
0.832971856	-7.22657633	cg16150863 6
29456577	p	GABBR1	MAS1L	NA	TSS1500	open
sea	TSS1500 - open sea	NA	0.874959251	0.867075928
-0.007883324	1.009091849
0.012996149	0.832985709	1.040078749	0.306179799
0.832971856	-7.226786649	cg10647703 6
29708958	p	MOG	LOC285830 NA	Body	open
sea	Body - open sea	NA	0.828518283	0.841514432
0.012996149	0.984556238
0.010023588	0.83416866 1.038113033	0.307079717
0.832971856	-7.228820597	cg19022254 6
29601705	p	GABBR1/MOG GABBR1	NA	TSS1500	shore
TSS1500 - shore	NA	0.830723052	0.84074664
0.010023588	0.988077754
0.005655981	0.89586781 1.038051734	0.307107809
0.832971856	-7.228883965	cg20496034 6
29574810	p	GABBR1/MOG GABBR1	NA	Body	open
sea	Body - open sea	NA	0.893923567	0.899579548
0.005655981	0.993712639

-0.006274492 0.914360214 -1.037733519 0.307253672
0.832971856 -7.229212869 cg13615337 6
29427915 p GABBR1 OR2H1 NA 5'UTR open
sea 5'UTR - open sea NA 0.91651707 0.910242578 -
0.006274492 1.006893209
0.017746136 0.662114774 1.034932427 0.308539702
0.832971856 -7.232103988 cg20893956 6
152126736 q ESR1 ESR1 NA 5'UTR shelf
5'UTR - shelf NA 0.65601454 0.673760676
0.017746136 0.973661069
0.008817761 0.683987581 1.034491244 0.308742597
0.832971856 -7.232558685 cg23134100 6
29701494 p MOG LOC285830 NA Body open
sea Body - open sea NA 0.680956476 0.689774237
0.008817761 0.987216454
0.008251172 0.854424721 1.033884372 0.30902184
0.832971856 -7.23318385 cg00114160 6
29430096 p GABBR1 OR2H1 NA Body open
sea Body - open sea NA 0.85158838 0.859839552
0.008251172 0.990403824
0.004663732 0.057431637 1.031567992 0.310089298
0.832971856 -7.235566902 cg17810098 22
19929066 q COMT TXNRD2 NA Body shore
Body - shore NA 0.055828479 0.060492211
0.004663732 0.922903595
-0.006798859 0.917174792 -1.031423028 0.310156186
0.832971856 -7.235715872 cg02837432 12
72232889 q TPH2 TBC1D15 NA TSS1500 shore
TSS1500 - shore NA 0.9195119 0.912713041 -
0.006798859 1.007449065
-0.00225425 0.038077218 -1.029242732 0.311163412
0.832971856 -7.237954063 cg03202557 6
29617599 p GABBR1/MOG MOG -7159 IGR shore
IGR - shore NA 0.038852117 0.036597867
-0.00225425 1.06159512
-0.013117852 0.174165316 -1.029232646 0.311168076
0.832971856 -7.237964406 cg11092416 6
29617549 p GABBR1/MOG MOG -7209 IGR shore
IGR - shore NA 0.178674578 0.165556726
-0.013117852 1.079234787
0.003642541 0.095010832 1.028141497 0.311673004
0.832971856 -7.239082856 cg06841846 17
28564094 q SLC6A4 SLC6A4 NA TSS1500 shore
TSS1500 - shore NA 0.093758709 0.09740125
0.003642541 0.962602728
-0.007363167 0.908411306 -1.025310115 0.312985863
0.832971856 -7.241979904 cg03881768 6
29582192 p GABBR1/MOG GABBR1 NA Body open
sea Body - open sea NA 0.910942395 0.903579228
-0.007363167 1.008148889
-0.004797655 0.907147473 -1.024658185 0.31328869
0.832971856 -7.242645895 cg03741824 21
34350407 q OLIG2 OLIG2 -47809 IGR shore
IGR - shore NA 0.908796667 0.903999012
-0.004797655 1.005307146
-0.012257389 0.629292654 -1.023516389 0.313819551
0.832971856 -7.243811363 cg06346307 22

19949965 q COMT COMT NA 5'UTR open
sea 5'UTR - open sea NA 0.633506131 0.621248742
-0.012257389 1.019730244
-0.006557823 0.880330455 -1.023094315 0.314015945
0.832971856 -7.24424188 cg21652192 6
29571483 p GABBR1/MOG GABBR1 NA Body open
sea Body - open sea NA 0.882584706 0.876026883
-0.006557823 1.00748587
0.001628207 0.018517086 1.022743265 0.314179355
0.832971856 -7.244599824 cg07252731 15
88799999 q NTRK3 NTRK3 NA TSS1500 island
TSS1500 - island NA 0.01795739 0.019585597
0.001628207 0.916867124
0.014907081 0.206514844 1.022717957 0.314191139
0.832971856 -7.244625625 cg18132851 6
152085641 q ESR1 ESR1 NA 5'UTR open
sea 5'UTR - open sea NA 0.201390535 0.216297616
0.014907081 0.931080697
-0.005146556 0.880192468 -1.020190165 0.315369563
0.83425901 -7.247199613 cg19530293 12 72426134 q
TPH2 TPH2 NA 3'UTR open sea 3'UTR - open sea
NA 0.881961597 0.876815041 -0.005146556
1.005869603
-0.01318991 0.407272395 -1.01865998 0.316084393
0.83425901 -7.248754865 cg20139800 6 29599178 p
GABBR1/MOG GABBR1 NA Body shore Body - shore
NA 0.411806427 0.398616517 -0.01318991
1.033089221
0.002543803 0.031204663 1.017758251 0.316506159
0.83425901 -7.249670342 cg17748329 6 29717058 p
MOG LOC285830 NA TSS1500 island TSS1500 - island
NA 0.030330231 0.032874034 0.002543803
0.922619688
-0.003955666 0.067536196 -1.016050658 0.317305913
0.834758633 -7.251401888 cg11813455 6
152128515 q ESR1 ESR1 NA TSS1500 shore
TSS1500 - shore NA 0.068895956 0.064940291
-0.003955666 1.060912339
0.002989921 0.072771941 1.013619973 0.318446721
0.836151851 -7.253861972 cg22215258 2
172544429 q SLC25A12 DYNC1I2 NA 5'UTR island
5'UTR - island NA 0.071744155 0.074734077
0.002989921 0.959992521
0.010902448 0.795254552 1.011604802 0.319394645
0.837034242 -7.255897329 cg06122864 6
29629187 p GABBR1/MOG MOG NA Body open
sea Body - open sea NA 0.791506835 0.802409283
0.010902448 0.986412859
0.008394736 0.81231448 1.00482885 0.322596191 0.843599992
-7.262713304 cg25836061 22 19939028 q COMT
COMT NA 5'UTR open sea 5'UTR - open sea NA
0.809428789 0.817823525 0.008394736
0.989735272
0.00306135 0.045305948 1.003696527 0.32313333 0.843599992
-7.263848125 cg22762215 6 29521272 p GABBR1
UBD -2117 IGR island IGR - island NA
0.044253609 0.047314959 0.00306135 0.935298475

-0.008621672 0.919395324 -1.002297882 0.323797648
0.843724156 -7.265248199 cg07189962 6
152126092 q ESR1 ESR1 NA 5'UTR shelf
5'UTR - shelf NA 0.922359023 0.913737352
-0.008621672 1.009435612
0.004010182 0.049134746 0.997849722 0.325916594
0.846880536 -7.26968872 cg24772753 2
171573419 q GAD1 SP5 NA Body island
Body - island NA 0.047756246 0.051766427
0.004010182 0.92253317
0.001880727 0.017643108 0.997157677 0.326247107
0.846880536 -7.27037791 cg13550731 2
172543902 q SLC25A12 DYNC1I2 NA TSS200 island
TSS200 - island NA 0.016996608 0.018877335
0.001880727 0.900371159
0.005150762 0.932340272 0.989910694 0.329721896
0.84962562 -7.277568014 cg25504668 6 29572379 p
GABBR1/MOG GABBR1 NA Body open sea Body - open sea
NA 0.930569697 0.935720459 0.005150762
0.994495405
0.00500792 0.928092317 0.98939253 0.329971305 0.84962562 -
7.278080223 cg17071948 6 29590143 p GABBR1/MOG GABBR1
NA Body open sea Body - open sea NA
0.926370844 0.931378764 0.00500792 0.994623111
0.004562232 0.08984373 0.989055676 0.330133511
0.84962562 -7.27841307 cg13227691 12 72233472 q
TPH2 TBC1D15 NA TSS200 island TSS200 - island
NA 0.088275463 0.092837694 0.004562232
0.950857989
-0.015250082 0.87281313 -0.988323345 0.330486341
0.84962562 -7.279136324 cg16073467 6 29586772 p
GABBR1/MOG GABBR1 NA Body open sea Body - open sea
NA 0.878055345 0.862805263 -0.015250082
1.017674999
-0.002559098 0.030159058 -0.984176038 0.332489295
0.84962562 -7.283222721 cg08541345 6 29716319 p
MOG LOC285830 NA Body shore Body - shore
NA 0.031038748 0.02847965 -0.002559098
1.089857073
-0.052202853 0.767263351 -0.982782949 0.333163931
0.84962562 -7.284591723 cg19636627 6 29649084 p
GABBR1/MOG ZFP57 8915 IGR open sea IGR - open sea
NA 0.785208082 0.733005228 -0.052202853
1.071217574
-0.001680491 0.042845983 -0.982520173 0.33329129
0.84962562 -7.284849752 cg26196213 6 29617956 p
GABBR1/MOG MOG -6802 IGR island IGR - island
NA 0.043423652 0.041743161 -0.001680491
1.040257876
0.011936359 0.822802686 0.979424644 0.334794073
0.84962562 -7.287884464 cg25189241 2 171785027 q
GAD1 GORASP2 NA TSS1500 shore TSS1500 - shore
NA 0.818699563 0.830635922 0.011936359
0.985629855
0.00713984 0.711500686 0.97879145 0.335102031 0.84962562 -
7.288504108 cg17930583 6 29591082 p GABBR1/MOG GABBR1

NA Body open sea Body - open sea NA
0.709046366 0.716186206 0.00713984 0.990030749
-0.008966656 0.892397939 -0.976513357 0.33621158
0.84962562 -7.290730329 cg15014679 11 27695210 p
BDNF BDNF NA Body open sea Body - open sea
NA 0.895480227 0.886513571 -0.008966656
1.010114516
0.004807806 0.915472847 0.975092348 0.336904939
0.84962562 -7.29211651 cg14656245 6 29627290 p
GABBR1/MOG MOG NA Body open sea Body - open sea
NA 0.913820164 0.91862797 0.004807806
0.994766319
0.002668825 0.023937479 0.974244313 0.337319182
0.84962562 -7.292942853 cg26375461 6 29716541 p
MOG LOC285830 NA Body island Body - island
NA 0.02302007 0.025688895 0.002668825
0.896109778
0.007837327 0.790615559 0.973383948 0.337739799
0.84962562 -7.293780521 cg23708209 15 88843899 q
NTRK3 NTRK3-AS1 47938 IGR open sea IGR - open sea
NA 0.787921477 0.795758804 0.007837327
0.990151128
0.003970506 0.059442507 0.972002792 0.338415759
0.84962562 -7.295123781 cg25774457 6 29600114 p
GABBR1/MOG GABBR1 NA Body shore Body - shore
NA 0.058077645 0.062048152 0.003970506
0.936009263
0.008672959 0.442351642 0.971682704 0.338572545
0.84962562 -7.29543483 cg12257233 6 29597083 p
GABBR1/MOG GABBR1 NA TSS1500 shore TSS1500 - shore
NA 0.439370313 0.448043271 0.008672959
0.980642588
0.002334683 0.02469505 0.971184767 0.338816543
0.84962562 -7.295918514 cg12941622 2 172778750 q
SLC25A12 HAT1 NA TSS200 island TSS200 - island
NA 0.023892502 0.026227186 0.002334683
0.910982291
-0.004699716 0.921479686 -0.968437862 0.340164698
0.84962562 -7.298582593 cg02450267 6 29627395 p
GABBR1/MOG MOG NA Body open sea Body - open sea
NA 0.923095214 0.918395497 -0.004699716
1.005117313
0.004418531 0.897531695 0.968078556 0.340341308
0.84962562 -7.29893054 cg01057705 22 19892666 q
COMT TXNRD2 NA Body shore Body - shore
NA 0.896012824 0.900431356 0.004418531
0.995092872
-0.004231873 0.075422874 -0.967426722 0.340661862
0.84962562 -7.299561455 cg23268677 22 19929097 q
COMT TXNRD2 NA Body island Body - island
NA 0.076877581 0.072645708 -0.004231873
1.058253586
0.00610792 0.833730347 0.966249378 0.34124136 0.84962562 -7.3007
cg13802605 9 4495359 p SLC1A1 SLC1A1 NA
Body shelf Body - shelf NA 0.83163075
0.83773867 0.00610792 0.992709039

-0.00419964 0.09043225 -0.965895208 0.341415815
0.84962562 -7.301042242 cg05265512 18 3447583 p
DLGAP1 TGIF1 NA 5'UTR shore 5'UTR - shore
NA 0.091875876 0.087676236 -0.00419964
1.04789941
-0.012285784 0.308732956 -0.965701965 0.341511027
0.84962562 -7.301228928 cg09641376 6 29520965 p
GABBR1 UBD -2424 IGR shore IGR - shore
NA 0.312956194 0.30067041 -0.012285784
1.040861301
0.008803152 0.807453854 0.965543234 0.341589248
0.84962562 -7.301382247 cg19382714 6 29624846 p
GABBR1/MOG MOG NA 1stExon open sea 1stExon - open
sea NA 0.80442777 0.813230922 0.008803152
0.989175089
0.004424607 0.908118098 0.960323679 0.344168074
0.853732456 -7.306410571 cg06787004 22
19938981 q COMT COMT NA 5'UTR open
sea 5'UTR - open sea NA 0.90659714 0.911021747
0.004424607 0.995143248
-0.005256588 0.926700989 -0.954964486 0.346829407
0.853732456 -7.311546663 cg01418645 11
27679469 p BDNF BDNF NA Body open
sea Body - open sea NA 0.928507941 0.923251353
-0.005256588 1.005693561
0.005713428 0.842197693 0.954365944 0.347127489
0.853732456 -7.312118603 cg08292919 6
29565696 p GABBR1/MOG GABBR1 -4309 IGR open
sea IGR - open sea NA 0.840233702 0.845947131
0.005713428 0.993246116
0.003347076 0.077755265 0.952126626 0.348244211
0.853732456 -7.314255396 cg27079446 18 3449844
p DLGAP1 TGIF1 NA 5'UTR island 5'UTR -
island NA 0.076604707 0.079951784 0.003347076
0.958136306
-0.003413875 0.078449553 -0.951294732 0.348659677
0.853732456 -7.315047996 cg04711050 9 4490757
p SLC1A1 SLC1A1 NA 1stExon island 1stExon
- island NA 0.079623072 0.076209197 -0.003413875
1.044796102
-0.002559665 0.088162082 -0.943882257 0.352376184
0.853732456 -7.322081439 cg23666278 2
171785822 q GAD1 GORASP2 NA 5'UTR island
5'UTR - island NA 0.089041967 0.086482302
-0.002559665 1.029597559
0.002340207 0.968004831 0.942634961 0.353004134
0.853732456 -7.323259846 cg15136600 6
29571468 p GABBR1/MOG GABBR1 NA Body open
sea Body - open sea NA 0.967200385 0.969540592
0.002340207 0.997586272
-0.005532626 0.8594572 -0.942076233 0.353285665
0.853732456 -7.323787236 cg06045576 22
19948957 q COMT COMT NA 5'UTR open
sea 5'UTR - open sea NA 0.861359041 0.855826415
-0.005532626 1.006464659
-0.006516777 0.817136271 -0.939832275 0.354417848
0.853732456 -7.32590236 cg03639249 6

29574225 p GABBR1/MOG GABBR1 NA Body open
sea Body - open sea NA 0.819376413 0.812859636
-0.006516777 1.0080171
0.012264785 0.722004991 0.939284468 0.354694606
0.853732456 -7.326417991 cg12790373 6
29635347 p GABBR1/MOG MOG NA 3'UTR open
sea 3'UTR - open sea NA 0.717788971 0.730053756
0.012264785 0.983200162
-0.002111837 0.045508835 -0.938511788 0.355085216
0.853732456 -7.327144804 cg13477819 6
29617973 p GABBR1/MOG MOG -6785 IGR island
IGR - island NA 0.046234779 0.044122942
-0.002111837 1.047862561
-0.009000263 0.628365835 -0.938434972 0.355124065
0.853732456 -7.327217029 cg11629449 6
29556084 p GABBR1/MOG OR2H2 NA 1stExon open
sea 1stExon - open sea V$PPARG_01 0.631459675 0.622459412
-0.009000263 1.014459197
0.007845083 0.842818755 0.937025161 0.355837545
0.853732456 -7.328541591 cg07326586 6
29528119 p GABBR1/MOG UBD NA TSS1500 open
sea TSS1500 - open sea NA 0.840122007 0.84796709
0.007845083 0.990748364
-0.012120534 0.299069397 -0.936389936 0.356159331
0.853732456 -7.329137789 cg02333875 6
29618205 p GABBR1/MOG MOG -6553 IGR shore
IGR - shore NA 0.30323583 0.291115296 -
0.012120534 1.041634824
-0.003070534 0.060249396 -0.935117341 0.356804568
0.853732456 -7.330331048 cg06358612 17
28619293 q SLC6A4 BLMH NA TSS1500 shore
TSS1500 - shore NA 0.061304892 0.058234358
-0.003070534 1.052727189
-0.003551098 0.913062867 -0.934180027 0.357280302
0.853732456 -7.331208947 cg01311802 6
29430435 p GABBR1 OR2H1 NA Body open
sea Body - open sea NA 0.914283556 0.910732458
-0.003551098 1.003899167
-0.004624746 0.919809774 -0.933828199 0.35745898
0.853732456 -7.331538258 cg25414639 15
88360194 q NTRK3 NTRK3-AS1 -59794 IGR open
sea IGR - open sea NA 0.921399531 0.916774785
-0.004624746 1.005044582
-0.016486181 0.315212927 -0.933023354 0.357867948
0.853732456 -7.33229115 cg20215212 17
28618041 q SLC6A4 BLMH NA Body shore
Body - shore NA 0.320880052 0.304393871
-0.016486181 1.054160686
0.002716761 0.073040683 0.932948427 0.357906037
0.853732456 -7.33236121 cg22043168 11
27741077 p BDNF BDNF NA Body island
Body - island NA 0.072106796 0.074823557
0.002716761 0.963691101
0.019934669 0.60171588 0.932099499 0.35833777 0.853732456
-7.333154615 cg20198768 6 29635579 p
GABBR1/MOG MOG NA 3'UTR open sea 3'UTR - open sea

NA 0.594863338 0.614798007 0.019934669
0.967575254
0.003679572 0.097574393 0.931584126 0.358600036
0.853732456 -7.333635947 cg10580691 18 3451079
p DLGAP1 TGIF1 NA 5'UTR island 5'UTR -
island NA 0.09630954 0.099989112 0.003679572
0.963200273
0.00284378 0.034817251 0.931164057 0.358813897
0.853732456 -7.334028083 cg21632158 6
29521356 p GABBR1 UBD -2033 IGR island
IGR - island NA 0.033839702 0.036683482
0.00284378 0.922477915
0.003312128 0.057470378 0.929972053 0.359421215
0.853732456 -7.335139912 cg21164232 18 3452359
p DLGAP1 TGIF1 NA 5'UTR island 5'UTR -
island NA 0.056331834 0.059643962 0.003312128
0.944468344
-0.006277336 0.914502777 -0.92311736 0.362926763
0.853732456 -7.341507379 cg13228862 18 3729405
p DLGAP1 DLGAP1 NA Body island Body -
island NA 0.916660611 0.910383275 -0.006277336
1.006895267
-0.003803161 0.143295556 -0.922514051 0.363236371
0.853732456 -7.342065669 cg01292475 9
87284571 q NTRK2 NTRK2 NA TSS200 island
TSS200 - island NA 0.144602893 0.140799732
-0.003803161 1.027011138
0.013783901 0.229444267 0.921662807 0.363673509
0.853732456 -7.342852804 cg05009707 6
29555799 p GABBR1/MOG OR2H2 NA 1stExon open
sea 1stExon - open sea NA 0.224706052 0.238489952
0.013783901 0.942203435
0.004157126 0.078360132 0.920091867 0.364481137
0.853732456 -7.344303625 cg13015908 21
34481860 q OLIG2 OLIG1 39410 IGR open
sea IGR - open sea NA 0.076931119 0.081088246
0.004157126 0.948733297
-0.003020367 0.953811348 -0.91857352 0.365262842
0.853732456 -7.345703645 cg20067272 6
29571693 p GABBR1/MOG GABBR1 NA Body open
sea Body - open sea NA 0.954849599 0.951829232
-0.003020367 1.003173224
0.002447732 0.05659984 0.9182389 0.365435265 0.853732456
-7.346011892 cg14857596 12 72233493 q TPH2
TBC1D15 NA 1stExon island 1stExon - island NA
0.055758432 0.058206164 0.002447732
0.957947203
-0.011860733 0.806484105 -0.916592027 0.366284642
0.853732456 -7.347527421 cg21026120 6
29581121 p GABBR1/MOG GABBR1 NA Body open
sea Body - open sea V$PAX5_01 0.810561232 0.798700499
-0.011860733 1.014850038
0.004171713 0.066572749 0.914654909 0.367285363
0.853732456 -7.349306743 cg21608605 6
152128258 q ESR1 ESR1 NA TSS1500 shore
TSS1500 - shore NA 0.065138723 0.069310436
0.004171713 0.939811185

0.003010481 0.05609956 0.914298311 0.367469777
0.853732456 -7.349633903 cg23164938 6
152128366 q ESR1 ESR1 NA TSS1500 shore
TSS1500 - shore NA 0.055064707 0.058075188
0.003010481 0.948162355
-0.015379353 0.564026747 -0.911591665 0.368871485
0.853732456 -7.35211317 cg03570920 12
72332964 q TPH2 TPH2 NA Body open
sea Body - open sea NA 0.569313399 0.553934046
-0.015379353 1.02776387
0.006960102 0.112021214 0.91146725 0.368936 0.853732456
-7.352226965 cg04105250 2 171679114 q GAD1
GAD1 NA Body island Body - island NA
0.109628679 0.116588781 0.006960102
0.940302129
-0.008093659 0.658204014 -0.908516001 0.370468522
0.853732456 -7.354921988 cg03037684 6
152421333 q ESR1 ESR1 NA 3'UTR open
sea 3'UTR - open sea NA 0.66098621 0.65289255 -0.008093659
1.012396619
0.006019817 0.898716405 0.908362955 0.370548109
0.853732456 -7.355061521 cg20704972 6
29574700 p GABBR1/MOG GABBR1 NA Body open
sea Body - open sea NA 0.896647093 0.90266691
0.006019817 0.993331076
-0.01566049 0.380381108 -0.906461291 0.371537933
0.853732456 -7.356793411 cg04522432 6
29618347 p GABBR1/MOG MOG -6411 IGR shore
IGR - shore NA 0.385764402 0.370103912
-0.01566049 1.042313765
0.004519665 0.898686204 0.906298906 0.371622535
0.853732456 -7.356941139 cg09149541 22
19898335 q COMT TXNRD2 NA Body shelf
Body - shelf NA 0.897132569 0.901652233
0.004519665 0.994987353
-0.00677205 0.695710515 -0.905070635 0.372262864
0.853732456 -7.358057733 cg00028318 6
29570739 p GABBR1/MOG GABBR1 NA 3'UTR open
sea 3'UTR - open sea NA 0.698038407 0.691266358
-0.00677205 1.009796584
0.004282928 0.916974957 0.904505158 0.372557901
0.853732456 -7.358571312 cg05598246 6
29624414 p GABBR1/MOG MOG NA TSS1500 open
sea TSS1500 - open sea NA 0.9155027 0.919785628
0.004282928 0.995343558
0.007892774 0.719338954 0.904354321 0.372636626
0.853732456 -7.358708254 cg26701815 18 3446566
p DLGAP1 TGIF1 NA 5'UTR shore 5'UTR -
shore NA 0.716625813 0.724518586 0.007892774
0.989106183
0.002346144 0.107482528 0.903643978 0.373007514
0.853732456 -7.35935287 cg19766164 6
29716939 p MOG LOC285830 NA TSS200 island
TSS200 - island NA 0.106676041 0.109022184
0.002346144 0.978480132
-0.002909815 0.042674177 -0.9025575 0.373575255
0.853732456 -7.360337887 cg10253022 22

19929467 q COMT TXNRD2 NA TSS200 island
TSS200 - island NA 0.043674426 0.040764611
-0.002909815 1.071380909
0.006144672 0.857550304 0.901207902 0.374281269
0.853732456 -7.361559885 cg22310628 6
29692995 p GABBR1/MOG HLA-F NA Body shore
Body - shore NA 0.855438073 0.861582745
0.006144672 0.992868158
-0.004036894 0.921766357 -0.900138992 0.37484106
0.853732456 -7.3625265 cg12111808 6 29588168 p
GABBR1/MOG GABBR1 NA Body open sea Body - open sea
NA 0.92315404 0.919117146 -0.004036894
1.004392143
-0.001888174 0.022271865 -0.899862696 0.374985846
0.853732456 -7.362776178 cg19655006 2
172750994 q SLC25A12 SLC25A12 NA TSS200 island
TSS200 - island NA 0.022920925 0.021032751
-0.001888174 1.08977304
0.003402792 0.182251834 0.898673037 0.375609667
0.853732456 -7.363850392 cg26741280 17
28563089 q SLC6A4 SLC6A4 NA TSS200 island
TSS200 - island NA 0.181082124 0.184484916
0.003402792 0.981555175
0.001493638 0.039384313 0.898666211 0.375613248
0.853732456 -7.363856551 cg03867475 21
34444382 q OLIG2 OLIG1 NA 1stExon island
1stExon - island NA 0.038870875 0.040364513
0.001493638 0.962996259
0.002679894 0.070518159 0.895895373 0.377068803
0.853732456 -7.366353251 cg15961225 2
171626884 q GAD1 GAD1 -46316 IGR shore
IGR - shore NA 0.069596945 0.072276839
0.002679894 0.962921815
-0.004326584 0.917069522 -0.895472931 0.377291037
0.853732456 -7.366733255 cg02589899 6
29627167 p GABBR1/MOG MOG NA Body open
sea Body - open sea V$TAXCREB_02 0.918556786
0.914230202 -0.004326584 1.004732489
-0.005357727 0.894742332 -0.895395817 0.377331614
0.853732456 -7.366802604 cg18701449 12
72335228 q TPH2 TPH2 NA Body open
sea Body - open sea NA 0.896584051 0.891226323
-0.005357727 1.006011636
0.003529852 0.119007133 0.894280809 0.377918632
0.853732456 -7.367804697 cg06260077 11
27721350 p BDNF BDNF NA Body shore
Body - shore NA 0.117793746 0.121323599
0.003529852 0.970905471
-0.007361449 0.880064574 -0.893937318 0.378099589
0.853732456 -7.368113164 cg23426002 11
27679729 p BDNF BDNF NA Body open
sea Body - open sea NA 0.882595072 0.875233623
-0.007361449 1.008410839
0.001531635 0.025350074 0.893845883 0.378147768
0.853732456 -7.368195257 cg24847163 6
29600980 p GABBR1/MOG GABBR1 NA TSS200 shore

TSS200 - shore	NA	0.024823574	0.026355209
0.001531635	0.941884923
-0.011302371	0.494288065	-0.893568386	0.37829401
0.853732456	-7.368444352	cg18014983 6
29618382	p	GABBR1/MOG MOG	-6376	IGR	shore
IGR - shore	NA	0.498173255	0.486870885
-0.011302371	1.023214307
0.008270578	0.805152222	0.892589263	0.378810306
0.853732456	-7.369322676	cg15198068 22
19843949	q	COMT	GNB1L	NA	TSS1500	shore
TSS1500 - shore	NA	0.80230921 0.810579788
0.008270578	0.989796713
-0.00223741	0.037785057	-0.892584964	0.378812574
0.853732456	-7.369326531	cg27066254 17
28443640	q	SLC6A4	CCDC55	NA	TSS200	shore
TSS200 - shore	NA	0.038554167	0.036316757
-0.00223741	1.061608199
-0.013643303	0.880723438	-0.884909243	0.382875761
0.860636913	-7.376180283	cg02979010 12
72319077	q	TPH2	TBC1D15	NA	3'UTR	open
sea	3'UTR - open sea	NA	0.885413323	0.87177002 -
0.013643303	1.015650117
0.003594649	0.106519355	0.883925278	0.383398645
0.860636913	-7.377054805	cg03696345 21
34398114	q	OLIG2	OLIG2	NA	TSS200	island
TSS200 - island	NA	0.105283695	0.108878344
0.003594649	0.96698472
0.002812859	0.089800944	0.883239136	0.383763536
0.860636913	-7.377664083	cg04057037 6
29600203	p	GABBR1/MOG GABBR1	NA	5'UTR	island
5'UTR - island	NA	0.088834024	0.091646882
0.002812859	0.969307652
0.003724721	0.095655872	0.880435897	0.385256613
0.86257127 -7.38014861	cg27230724 2	172751074 q
SLC25A12	SLC25A12	NA	TSS1500	shore	TSS1500 - shore
NA	0.094375499	0.09810022 0.003724721
0.962031471
-0.005877407	0.85191978 -0.877545845	0.386799818
0.864611358	-7.382702208	cg13175282 22
19938541	q	COMT	COMT	NA	5'UTR	open
sea	5'UTR - open sea	NA	0.853940139	0.848062731
-0.005877407	1.006930393
0.003738689	0.930233724	0.874770039	0.388285735
0.866516942	-7.38514733	cg21627017 6
29719792	p	MOG	IFITM4P	NA	TSS1500	shore
TSS1500 - shore	NA	0.928948549	0.932687239
0.003738689	0.995991486
0.005002833	0.092635565	0.869736027	0.390989774
0.871130311	-7.389562805	cg02115911 21
34395548	q	OLIG2	OLIG2	-2668	IGR	island
IGR - island	NA	0.090915841	0.095918674
0.005002833	0.947842972
-0.004150073	0.873463895	-0.862100258	0.395114171
0.877511213	-7.396213973	cg16912910 6
29497165	p	GABBR1	LINC01015 -18	IGR	open
sea	IGR - open sea	NA	0.874890482	0.870740409
-0.004150073	1.004766143

0.002890399 0.930743975 0.860871593 0.395780389
0.877511213 -7.39727898 cg17675298 22
19961051 q COMT ARVCF NA Body island
Body - island NA 0.9297504 0.932640799
0.002890399 0.996900844
0.002767524 0.059725263 0.858416234 0.397113888
0.877511213 -7.399402946 cg20253551 6
152129400 q ESR1 ESR1 NA Body island
Body - island V$HEN1_02;V$E47_01 0.058773927
0.061541451 0.002767524 0.95502992
0.002119474 0.027538624 0.858350981 0.397149365
0.877511213 -7.399459313 cg14888846 17
28443962 q SLC6A4 MIR423 NA TSS200 island
TSS200 - island NA 0.026810055 0.028929529
0.002119474 0.926736657
-0.003671722 0.038545081 -0.856825644 0.397979246
0.877511213 -7.400775767 cg20034792 6
29521781 p GABBR1 UBD -1608 IGR island
IGR - island NA 0.039807236 0.036135514
-0.003671722 1.10160979
0.002572538 0.06048141 0.855707464 0.398588303
0.877511213 -7.401739402 cg26391350 2
171673572 q GAD1 GAD1 NA 5'UTR island
5'UTR - island NA 0.0595971 0.062169638
0.002572538 0.958620669
0.002776648 0.064004074 0.854644199 0.399167993
0.877511213 -7.402654597 cg14276488 9 4491114
p SLC1A1 SLC1A1 NA Body shore Body -
shore NA 0.063049601 0.065826249 0.002776648
0.957818529
0.030080607 0.428207513 0.852656762 0.400252966
0.877511213 -7.40436235 cg08065408 6
29520774 p GABBR1 UBD -2615 IGR shore
IGR - shore NA 0.417867304 0.447947912
0.030080607 0.932847978
-0.004375405 0.072661689 -0.851549515 0.400858234
0.877511213 -7.405312132 cg26158180 15
88798666 q NTRK3 NTRK3 NA Body shore
Body - shore NA 0.074165734 0.06979033 -
0.004375405 1.062693557
0.008459181 0.670658376 0.851057579 0.401127332
0.877511213 -7.40573373 cg16994534 6
29620138 p GABBR1/MOG MOG -4620 IGR shelf
IGR - shelf NA 0.667750533 0.676209714
0.008459181 0.987490299
0.002609421 0.052296877 0.848320902 0.402626418
0.877511213 -7.408074862 cg24102938 17
28444044 q SLC6A4 MIR423 NA TSS200 shore
TSS200 - shore NA 0.051399889 0.054009309
0.002609421 0.951685736
-0.004386338 0.077831316 -0.848225301 0.40267885
0.877511213 -7.408156515 cg10002133 15
88798448 q NTRK3 NTRK3 NA Body shore
Body - shore NA 0.07933912 0.074952781 -
0.004386338 1.058521364
0.010274212 0.695447951 0.848185816 0.402700506
0.877511213 -7.408190236 cg06061002 6

29638918 p GABBR1/MOG MOG NA 3'UTR open
sea 3'UTR - open sea NA 0.69191619 0.702190402
0.010274212 0.985368339
-0.005414089 0.776969518 -0.84794228 0.402834095
0.877511213 -7.408398193 cg20642417 6
29577082 p GABBR1/MOG GABBR1 NA Body open
sea Body - open sea NA 0.778830611 0.773416522
-0.005414089 1.007000224
0.003734501 0.077972619 0.844797939 0.404561387
0.879072503 -7.411078027 cg02348151 21
34442257 q OLIG2 OLIG1 NA TSS200 island
TSS200 - island NA 0.076688884 0.080423385
0.003734501 0.953564489
0.009371984 0.791236329 0.844034916 0.40498124
0.879072503 -7.411726895 cg12646029 6
29427451 p GABBR1 OR2H1 NA 5'UTR open
sea 5'UTR - open sea NA 0.788014709 0.797386693
0.009371984 0.988246626
-0.00205462 0.053857675 -0.842250649 0.405964097
0.879072503 -7.413242033 cg11851910 21
34482086 q OLIG2 OLIG1 39636 IGR open
sea IGR - open sea NA 0.05456395 0.05250933 -0.00205462
1.039128665
0.003837363 0.887271675 0.84087896 0.4067207 0.879072503
-7.414404739 cg24430207 6 29589714 p
GABBR1/MOG GABBR1 NA Body open sea Body - open sea
NA 0.885952582 0.889789945 0.003837363
0.995687338
0.003388812 0.127392435 0.8384497 0.408062807
0.879072503 -7.416459444 cg00118317 21
34392029 q OLIG2 OLIG2 -6187 IGR shore
IGR - shore NA 0.126227531 0.129616343
0.003388812 0.973855056
-0.006614859 0.902006432 -0.838351788 0.408116959
0.879072503 -7.416542141 cg20168024 6
29456611 p GABBR1 MAS1L NA TSS1500 open
sea TSS1500 - open sea NA 0.90428029 0.897665431 -
0.006614859 1.007368958
0.001835067 0.041726043 0.838107628 0.408252016
0.879072503 -7.416748318 cg03670115 2
172544069 q SLC25A12 DYNC1I2 NA 5'UTR island
5'UTR - island NA 0.041095239 0.042930306
0.001835067 0.957254742
-0.011647066 0.808301904 -0.837047235 0.408838892
0.879072503 -7.417643082 cg25437807 6
29589545 p GABBR1/MOG GABBR1 NA Body open
sea Body - open sea NA 0.812305583 0.800658517
-0.011647066 1.014546858
0.003305376 0.070623228 0.835748374 0.409558467
0.879072503 -7.41873759 cg20723129 6
29521501 p GABBR1 UBD -1888 IGR island
IGR - island NA 0.069487005 0.072792381
0.003305376 0.954591731
0.003309733 0.051824474 0.834993926 0.409976796
0.879072503 -7.419372593 cg21032292 21
34395093 q OLIG2 OLIG2 -3123 IGR shore

IGR - shore NA 0.050686753 0.053996486
0.003309733 0.938704659
-0.002276834 0.034240068 -0.830846941 0.412280982
0.881337828 -7.422853219 cg06613392 6
29521595 p GABBR1 UBD -1794 IGR island
IGR - island NA 0.03502273 0.032745896 -
0.002276834 1.069530362
0.012305964 0.515607739 0.830773626 0.412321791
0.881337828 -7.422914605 cg02754084 12
72338080 q TPH2 TPH2 NA Body open
sea Body - open sea NA 0.511377564 0.523683528
0.012305964 0.976501144
0.004359883 0.901553598 0.825111121 0.415481202
0.886705592 -7.427640033 cg15983385 6
29589056 p GABBR1/MOG GABBR1 NA Body open
sea Body - open sea NA 0.900054888 0.904414771
0.004359883 0.995179332
-0.00776819 0.83778032 -0.821085808 0.417736226
0.890129528 -7.430980381 cg13801656 9 4685060
p SLC1A1 CDC37L1 NA Body open sea Body -
open sea NA 0.840450635 0.832682445 -0.00776819
1.009329115
-0.009977787 0.690477676 -0.814078747 0.421679639
0.896876046 -7.436757738 cg07579946 22
19949893 q COMT COMT NA 5'UTR open
sea 5'UTR - open sea NA 0.69390754 0.683929753 -
0.009977787 1.014588906
-0.002438011 0.062030202 -0.813133095 0.422213577
0.896876046 -7.437533796 cg01455471 6
29600468 p GABBR1/MOG GABBR1 NA 5'UTR island
5'UTR - island NA 0.062868269 0.060430258
-0.002438011 1.04034421
0.008415121 0.840763636 0.810604763 0.423643173
0.898517614 -7.439604449 cg25017994 6
29627296 p GABBR1/MOG MOG NA Body open
sea Body - open sea NA 0.837870938 0.84628606
0.008415121 0.99005641
0.008490153 0.79119745 0.808026508 0.425104049
0.90022034 -7.441709617 cg18484299 2 171787393 q
GAD1 GORASP2 NA Body shore Body - shore
NA 0.78827896 0.796769113 0.008490153
0.989344274
-0.003318607 0.048424356 -0.806112156 0.426190741
0.900587143 -7.44326854 cg15710245 11
27722620 p BDNF BDNF NA TSS200 island
TSS200 - island NA 0.049565128 0.04624652 -
0.003318607 1.071759086
-0.007589255 0.914320982 -0.804526291 0.42709225
0.900587143 -7.444557276 cg00521863 2
171783942 q GAD1 GORASP2 -1006 IGR shore
IGR - shore NA 0.916929788 0.909340534
-0.007589255 1.008345888
0.002907242 0.082563063 0.804245073 0.427252234
0.900587143 -7.44478555 cg17301635 18 3451475
p DLGAP1 TGIF1 NA 5'UTR island 5'UTR -
island NA 0.081563699 0.08447094 0.002907242
0.965582945

0.005824142 0.111331321 0.799205854 0.430125229
0.901855878 -7.448863074 cg00674706 6
29521145 p GABBR1 UBD -2244 IGR island
IGR - island NA 0.109329272 0.115153414
0.005824142 0.949422759
-0.003504347 0.906228769 -0.79857396 0.430486317
0.901855878 -7.449372639 cg12760319 2
172541622 q SLC25A12 DYNC1I2 -2360 IGR shelf
IGR - shelf NA 0.907433388 0.903929041
-0.003504347 1.003876794
0.008243481 0.736406081 0.792519297 0.433955511
0.901855878 -7.454235546 cg24444631 6
29636366 p GABBR1/MOG MOG NA 3'UTR open
sea 3'UTR - open sea NA 0.733572384 0.741815865
0.008243481 0.98888743
0.003299251 0.928122923 0.792505241 0.433963584
0.901855878 -7.454246794 cg07854670 2
171667934 q GAD1 GAD1 -5266 IGR shelf
IGR - shelf NA 0.926988805 0.930288056
0.003299251 0.996453517
0.001794658 0.041648033 0.791071862 0.434787362
0.901855878 -7.455392811 cg21472700 2
172543946 q SLC25A12 DYNC1I2 NA TSS200 island
TSS200 - island NA 0.041031119 0.042825777
0.001794658 0.958093977
0.008495168 0.857975945 0.790558529 0.43508261
0.901855878 -7.455802747 cg11358777 6
29638498 p GABBR1/MOG MOG NA 3'UTR open
sea 3'UTR - open sea NA 0.855055731 0.863550899
0.008495168 0.990162516
0.001357961 0.036068414 0.790471119 0.435132896
0.901855878 -7.455872525 cg20340655 11
27723075 p BDNF BDNF NA TSS1500 shore
TSS1500 - shore NA 0.035601615 0.036959576
0.001357961 0.963258209
-0.004431664 0.921836153 -0.790057376 0.435370969
0.901855878 -7.456202709 cg09595044 6
29426132 p GABBR1 OR2H1 NA TSS200 open
sea TSS200 - open sea NA 0.923359537 0.918927873
-0.004431664 1.004822646
0.002963639 0.073316563 0.789777504 0.435532055
0.901855878 -7.456425965 cg10558494 11
27721280 p BDNF BDNF NA Body shore
Body - shore NA 0.072297812 0.075261451
0.002963639 0.960622085
-0.027344006 0.521950345 -0.789625239 0.43561971
0.901855878 -7.456547395 cg08022281 6
29648345 p GABBR1/MOG ZFP57 8176 IGR open
sea IGR - open sea NA 0.531349848 0.504005841
-0.027344006 1.054253353
0.002546157 0.041506258 0.7881643 0.436461271
0.901855878 -7.457711343 cg05016953 17
28562813 q SLC6A4 SLC6A4 NA 1stExon island
1stExon - island NA 0.040631016 0.043177173
0.002546157 0.941030021
0.002057537 0.042271481 0.786895905 0.437192715
0.901855878 -7.458720209 cg16489427 6

29721015 p MOG IFITM4P 2431 IGR island
IGR - island NA 0.041564202 0.04362174
0.002057537 0.95283228
0.001581744 0.026218586 0.78582503 0.437810831
0.901855878 -7.459570753 cg27034819 15
88799526 q NTRK3 NTRK3 NA 5'UTR island
5'UTR - island V$CDPCR1_01 0.025674861
0.027256605 0.001581744 0.941968415
0.002406992 0.064071735 0.785276166 0.438127843
0.901855878 -7.460006257 cg15313332 11
27721270 p BDNF BDNF NA Body shore
Body - shore NA 0.063244331 0.065651323
0.002406992 0.963336733
-0.004114953 0.915091118 -0.78427864 0.438704346
0.901855878 -7.46079701 cg25743221 6
152501416 q ESR1 SYNE1 NA Body open
sea Body - open sea NA 0.916505633 0.91239068 -
0.004114953 1.004510078
-0.004731257 0.882196732 -0.783681461 0.439049694
0.901855878 -7.461269939 cg01024792 6
29524067 p GABBR1 UBD NA Body shelf
Body - shelf NA 0.883823101 0.879091844
-0.004731257 1.005381983
-0.007428169 0.503097706 -0.781595801 0.440257112
0.901855878 -7.462918935 cg14352032 17
28564834 q SLC6A4 BLMH -10379 IGR shore
IGR - shore NA 0.505651139 0.498222971
-0.007428169 1.014909325
-0.002482505 0.060673644 -0.780287228 0.441015681
0.901855878 -7.463951381 cg13641185 6
29521143 p GABBR1 UBD -2246 IGR island
IGR - island NA 0.061527005 0.0590445 -
0.002482505 1.042044644
0.003033956 0.058758989 0.780015341 0.44117339
0.901855878 -7.464165687 cg24025650 6
29720582 p MOG IFITM4P 1998 IGR island
IGR - island NA 0.057716067 0.060750023
0.003033956 0.950058356
0.001569854 0.043649646 0.778730214 0.441919291
0.901855878 -7.465177674 cg09523380 6
29720748 p MOG IFITM4P 2164 IGR island
IGR - island NA 0.043110009 0.044679863
0.001569854 0.964864395
0.00646435 0.567368373 0.776734501 0.443079122
0.901855878 -7.466746037 cg27322282 18 3411906
p DLGAP1 TGIF1 NA TSS200 open sea TSS200
- open sea NA 0.565146253 0.571610603 0.00646435
0.98869099
0.015812042 0.118135503 0.776520096 0.443203835
0.901855878 -7.4669143 cg18374181 21 34401798 q
OLIG2 OLIG2 3582 IGR shore IGR - shore
NA 0.112700114 0.128512156 0.015812042
0.876960729
-0.005531429 0.717797207 -0.775244415 0.44394629
0.901855878 -7.467914518 cg17987649 6
29526534 p GABBR1/MOG UBD NA Body open

sea	Body - open sea	NA	0.719698636	0.714167207
-0.005531429	1.007745286
-0.003637354	0.04223579 -0.774913059	0.444139264
0.901855878	-7.468174065	cg23912231 6
29691408	p	GABBR1/MOG HLA-F	NA	Body	island
Body - island	NA	0.043486131	0.039848777
-0.003637354	1.091278937
0.002804927	0.072745419	0.774382079	0.444448598
0.901855878	-7.46858975	cg05645755 18	4454548
p	DLGAP1	DLGAP1-AS5 189946	IGR	island	IGR -
island	NA	0.071781225	0.074586153	0.002804927
0.962393449
0.003827456	0.918248158	0.772520282	0.445534245
0.901855878	-7.470045118	cg05183668 21
34350584	q	OLIG2	OLIG2	-47632	IGR	shore
IGR - shore	NA	0.91693247 0.920759927
0.003827456	0.995843154
0.002222097	0.08767867 0.771057402	0.446388387
0.901855878	-7.471186286	cg14446129 2
172778865 q	SLC25A12	HAT1	NA	TSS200	island
TSS200 - island	V$ELK1_01 0.086914824	0.089136921
0.002222097	0.97507097
0.003633835	0.852836244	0.767374932	0.448542815
0.901855878	-7.474049695	cg20486877 6
29594481	p	GABBR1/MOG GABBR1	NA	Body	shore
Body - shore	NA	0.851587114	0.855220949
0.003633835	0.995750999
-0.001332016	0.020986382	-0.766694144	0.448941787
0.901855878	-7.474577615	cg27220153 6
29720768	p	MOG	IFITM4P	2184	IGR	island
IGR - island	NA	0.021444263	0.020112247
-0.001332016	1.066229099
-0.001566201	0.019663196	-0.765606898	0.449579398
0.901855878	-7.475419788	cg27237300 21
34442292	q	OLIG2	OLIG1	NA	TSS200	island
TSS200 - island	NA	0.020201578	0.018635376
-0.001566201	1.084044561
-0.020179541	0.740772079	-0.764379153	0.450300051
0.901855878	-7.476369406	cg11617938 6
29692281	p	GABBR1/MOG HLA-F	NA	Body	shore
Body - shore	NA	0.747708796	0.727529255
-0.020179541	1.027737085
-0.001824338	0.03686289 -0.76418591	0.450413542
0.901855878	-7.476518739	cg18488157 6
29521598	p	GABBR1	UBD	-1791	IGR	island
IGR - island	NA	0.037490006	0.035665668
-0.001824338	1.051151096
0.007336273	0.143641476	0.763460053	0.450839986
0.901855878	-7.477079337	cg20196537 2
171574548 q	GAD1	SP5	2691	IGR	shore
IGR - shore	NA	0.141119632	0.148455905
0.007336273	0.950582814
0.004808832	0.886898392	0.760001109	0.452875419
0.901855878	-7.479743711	cg16924391 6
29719411	p	MOG	IFITM4P	NA	TSS1500	shore
TSS1500 - shore	NA	0.885245356	0.890054188
0.004808832	0.994597147

0.002493248 0.096815091 0.758553952 0.453728617
0.901855878 -7.480854974 cg09606766 11
27722971 p BDNF BDNF NA TSS1500 shore
TSS1500 - shore NA 0.095958037 0.098451285
0.002493248 0.974675313
0.001955537 0.06004835 0.758496206 0.453762682
0.901855878 -7.480899275 cg01583365 2
172778734 q SLC25A12 HAT1 NA TSS200 island
TSS200 - island NA 0.059376134 0.061331672
0.001955537 0.968115365
0.003158577 0.926102734 0.758060773 0.454019597
0.901855878 -7.481233217 cg12457376 22
19928061 q COMT COMT NA TSS1500 shore
TSS1500 - shore NA 0.925016973 0.92817555
0.003158577 0.996597005
0.001193611 0.021540682 0.757999894 0.454055524
0.901855878 -7.481279891 cg05101432 6
29720737 p MOG IFITM4P 2153 IGR island
IGR - island NA 0.021130379 0.02232399
0.001193611 0.946532363
-0.007853717 0.451855794 -0.757360942 0.454432692
0.901855878 -7.481769544 cg13468667 18 3411996
p DLGAP1 TGIF1 NA TSS200 open sea TSS200
- open sea NA 0.454555509 0.446701792 -0.007853717
1.017581566
-0.009203743 0.311795074 -0.756103768 0.455175332
0.901855878 -7.482731799 cg09144707 2
171678251 q GAD1 GAD1 NA Body shore
Body - shore NA 0.31495886 0.305755117 -
0.009203743 1.030101681
-0.001800799 0.077239378 -0.754940171 0.455863333
0.901855878 -7.483621056 cg03409187 6
29521624 p GABBR1 UBD -1765 IGR island
IGR - island NA 0.077858403 0.076057604
-0.001800799 1.023676778
-0.00291906 0.082580412 -0.754464731 0.456144623
0.901855878 -7.483984022 cg01636003 11
27723385 p BDNF BDNF NA TSS1500 shore
TSS1500 - shore NA 0.083583839 0.080664779
-0.00291906 1.036187541
0.004099977 0.911159523 0.754025369 0.456404658
0.901855878 -7.484319248 cg03200120 6
29641443 p GABBR1/MOG ZFP57 NA Body open
sea Body - open sea NA 0.909750156 0.913850133
0.004099977 0.995513513
0.001709832 0.028347655 0.753254339 0.456861202
0.901855878 -7.484907078 cg03471611 17
28431762 q SLC6A4 EFCAB5 NA Body open
sea Body - open sea NA 0.027759901 0.029469732
0.001709832 0.941980097
-0.004662248 0.086218693 -0.751846478 0.45769552
0.902200967 -7.485978926 cg00465975 22
19929557 q COMT TXNRD2 NA TSS200 shore
TSS200 - shore NA 0.087821341 0.083159093
-0.004662248 1.0560642
-0.003790782 0.061337202 -0.746486921 0.460879858
0.905672294 -7.490041618 cg14843922 21

34398849 q OLIG2 OLIG2 NA 5'UTR island
5'UTR - island NA 0.062640283 0.058849501
-0.003790782 1.064414854
0.001484981 0.014255238 0.744812658 0.461877262
0.905672294 -7.491305004 cg17298239 18 3499253
p DLGAP1 DLGAP1 NA Body island Body -
island NA 0.013744775 0.015229757 0.001484981
0.902494702
-0.004839442 0.88843989 -0.743193942 0.462842775
0.905672294 -7.49252387 cg13460297 2
172581104 q SLC25A12 DYNC1I2 NA Body open
sea Body - open sea NA 0.890103448 0.885264007
-0.004839442 1.005466664
-0.012945789 0.691592876 -0.74232779 0.463359891
0.905672294 -7.493175017 cg08725892 6
29629986 p GABBR1/MOG MOG NA Body open
sea Body - open sea NA 0.696042991 0.683097202
-0.012945789 1.018951606
0.001384901 0.037927456 0.741676486 0.463748959
0.905672294 -7.493664164 cg22860601 2
171627721 q GAD1 GAD1 -45479 IGR island
IGR - island NA 0.037451396 0.038836297
0.001384901 0.964340035
0.00547558 0.781936486 0.741228127 0.464016906
0.905672294 -7.494000653 cg23270757 6
29427699 p GABBR1 OR2H1 NA 5'UTR open
sea 5'UTR - open sea NA 0.780054255 0.785529835
0.00547558 0.993029444
-0.003243666 0.90448716 -0.740706076 0.464329006
0.905672294 -7.4943922 cg16922688 6 29588173 p
GABBR1/MOG GABBR1 NA Body open sea Body - open sea
NA 0.90560217 0.902358504 -0.003243666
1.003594653
-0.010518679 0.844206589 -0.737947362 0.465980289
0.905672294 -7.496456855 cg07989678 2
172543677 q SLC25A12 DYNC1I2 NA TSS1500 shore
TSS1500 - shore NA 0.847822385 0.837303706
-0.010518679 1.012562561
-0.001711395 0.038198818 -0.737031248 0.466529402
0.905672294 -7.497140839 cg21229268 21
34442350 q OLIG2 OLIG1 NA TSS200 island
TSS200 - island NA 0.03878711 0.037075715 -
0.001711395 1.04615946
-0.007000171 0.860945198 -0.736644563 0.466761291
0.905672294 -7.497429298 cg09926649 22
19938096 q COMT COMT NA 5'UTR open
sea 5'UTR - open sea NA 0.863351507 0.856351336
-0.007000171 1.008174415
0.003628362 0.933644292 0.736167023 0.467047758
0.905672294 -7.49778533 cg26598649 18 3880086
p DLGAP1 DLGAP1 NA 1stExon island 1stExon
- island NA 0.932397042 0.936025405 0.003628362
0.996123649
0.002815881 0.057164436 0.735205523 0.467624853
0.905672294 -7.498501505 cg25627226 18 4455337
p DLGAP1 DLGAP1-AS5 190735 IGR island IGR -

island NA 0.056196477 0.059012358 0.002815881
0.952283198
-0.002546772 0.047421395 -0.734430267 0.468090464
0.905672294 -7.499078296 cg00346247 6
29716851 p MOG LOC285830 NA TSS200 island
TSS200 - island NA 0.048296848 0.045750076
-0.002546772 1.055667055
0.0031225 0.901127106 0.733139963 0.468866005
0.905672294 -7.500036981 cg10939667 6
152201611 q ESR1 ESR1 NA Body open
sea Body - open sea NA 0.900053746 0.903176246
0.0031225 0.996542757
0.003815652 0.88142404 0.731623221 0.469778599
0.905672294 -7.501161823 cg08196667 6
29549180 p GABBR1/MOG SNORD32B NA TSS1500 open
sea TSS1500 - open sea NA 0.880112409 0.883928061
0.003815652 0.9956833
-0.001415655 0.039804835 -0.72999731 0.47075802
0.905672294 -7.502365125 cg19348622 6
29691760 p GABBR1/MOG HLA-F NA Body island
Body - island NA 0.040291467 0.038875812
-0.001415655 1.036414802
0.003151526 0.939748064 0.728873848 0.471435463
0.905672294 -7.503195064 cg25197880 6
29695413 p GABBR1/MOG LOC285830 NA Body shelf
Body - shelf NA 0.938664727 0.941816253
0.003151526 0.996653778
-0.018936708 0.754930004 -0.728056696 0.471928557
0.905672294 -7.503797944 cg04071440 6
29648275 p GABBR1/MOG ZFP57 8106 IGR open
sea IGR - open sea NA 0.761439498 0.74250279 -
0.018936708 1.025503888
-0.004131454 0.143098495 -0.726176226 0.473064418
0.905672294 -7.505182839 cg21829923 2
171573891 q GAD1 SP5 NA Body island
Body - island V$PAX4_01 0.144518682 0.140387228
-0.004131454 1.029428988
-0.00581516 0.714759184 -0.725581485 0.473423988
0.905672294 -7.505620122 cg12021641 6
29555593 p GABBR1/MOG OR2H2 NA TSS200 open
sea TSS200 - open sea NA 0.716758145 0.710942985
-0.00581516 1.008179503
0.004057688 0.177092014 0.723688205 0.474569679
0.905672294 -7.507009848 cg00619335 2
171670134 q GAD1 GAD1 -3066 IGR island
IGR - island V$CMYB_01 0.175697184 0.179754872
0.004057688 0.977426548
-0.004217975 0.893251779 -0.723596324 0.47462532
0.905672294 -7.507077201 cg01078434 6
29455532 p GABBR1 MAS1L NA 1stExon open
sea 1stExon - open sea NA 0.894701707 0.890483733
-0.004217975 1.004736722
-0.007559813 0.335643626 -0.723500248 0.474683505
0.905672294 -7.507147622 cg24351901 6
29692092 p GABBR1/MOG HLA-F NA Body island
Body - island NA 0.338242312 0.330682499
-0.007559813 1.022861243

0.003936278 0.912547641 0.720671181 0.476398681
0.907188737 -7.509217176 cg25736198 12
72345225 q TPH2 TPH2 NA Body open
sea Body - open sea NA 0.911194545 0.915130823
0.003936278 0.995698672
-0.007687292 0.807520907 -0.717386838 0.478394332
0.907188737 -7.511609937 cg01405582 6
29692365 p GABBR1/MOG HLA-F NA Body shore
Body - shore NA 0.810163413 0.802476121
-0.007687292 1.009579465
-0.006685463 0.115031399 -0.716614279 0.478864453
0.907188737 -7.512171236 cg02226939 17
28619481 q SLC6A4 BLMH NA TSS1500 shore
TSS1500 - shore NA 0.117329527 0.110644064
-0.006685463 1.060423151
-0.005193623 0.855638135 -0.714879749 0.479920921
0.907188737 -7.51342932 cg13698224 9
87309394 q NTRK2 NTRK2 NA Body open
sea Body - open sea NA 0.857423444 0.85222982 -
0.005193623 1.006094159
0.010553091 0.633387613 0.714026553 0.480441073
0.907188737 -7.514047073 cg05255330 18 3498963
p DLGAP1 DLGAP1 NA 3'UTR island 3'UTR -
island NA 0.629759987 0.640313079 0.010553091
0.983518856
-0.015431169 0.749452967 -0.713547967 0.480732985
0.907188737 -7.514393279 cg15584790 6
29692475 p GABBR1/MOG HLA-F NA Body shore
Body - shore NA 0.754757431 0.739326263
-0.015431169 1.020871933
0.001730647 0.061780011 0.712741304 0.481225235
0.907188737 -7.514976305 cg23947039 11
27722037 p BDNF BDNF NA Body island
Body - island NA 0.061185102 0.062915749
0.001730647 0.972492627
0.001550581 0.029418706 0.712271767 0.481511894
0.907188737 -7.515315376 cg14545305 2
171670978 q GAD1 GAD1 -2222 IGR shore
IGR - shore NA 0.028885693 0.030436274
0.001550581 0.949054835
-0.001292137 0.022432485 -0.711420975 0.482031561
0.907188737 -7.515929212 cg14555167 22
19842472 q COMT GNB1L NA TSS200 island
TSS200 - island NA 0.022876657 0.021584519
-0.001292137 1.059864109
-0.003363696 0.092934164 -0.711292921 0.482109804
0.907188737 -7.51602154 cg25804470 6
29600193 p GABBR1/MOG GABBR1 NA 5'UTR island
5'UTR - island NA 0.094090435 0.090726739
-0.003363696 1.037075024
0.002324017 0.081720556 0.708293825 0.483944379
0.908853584 -7.518179309 cg25725890 17
28563054 q SLC6A4 SLC6A4 NA TSS200 island
TSS200 - island NA 0.080921675 0.083245692
0.002324017 0.972082435
0.007933504 0.849383517 0.7076752 0.484323291
0.908853584 -7.518623295 cg22179059 6

29714945 p MOG LOC285830 NA Body shore
Body - shore NA 0.846656375 0.854589879
0.007933504 0.990716595
-0.004391572 0.744543871 -0.704791719 0.486091665
0.909734714 -7.520687802 cg06087028 6
29526389 p GABBR1/MOG UBD NA Body open
sea Body - open sea NA 0.746053474 0.741661902
-0.004391572 1.005921259
0.001536162 0.027714386 0.704711361 0.486140999
0.909734714 -7.52074522 cg00370229 6
29521602 p GABBR1 UBD -1787 IGR island
IGR - island NA 0.02718633 0.028722492
0.001536162 0.946517106
0.001342637 0.015223509 0.703658125 0.486787873
0.909734714 -7.521497192 cg26770917 21
34444339 q OLIG2 OLIG1 NA 1stExon island
1stExon - island V$CART1_01 0.014761977 0.016104614
0.001342637 0.91663029
0.005836836 0.133852072 0.699262026 0.489493116
0.912398273 -7.524624079 cg10364942 21
34401201 q OLIG2 OLIG2 NA 3'UTR shore
3'UTR - shore NA 0.13184566 0.137682496
0.005836836 0.95760655
0.009316609 0.366992179 0.698089105 0.490216331
0.912398273 -7.525455152 cg27204993 9 4662937
p SLC1A1 C9orf68 NA Body island Body -
island NA 0.363789595 0.373106204 0.009316609
0.975029606
0.00140013 0.030477418 0.693960025 0.49276708 0.912398273
-7.528370043 cg15299832 21 34398131 q OLIG2
OLIG2 NA TSS200 island TSS200 - island NA
0.029996124 0.031396253 0.00140013 0.955404583
-0.001849767 0.042714323 -0.69187828 0.494055905
0.912398273 -7.529833275 cg10699871 22
19879696 q COMT TXNRD2 NA Body open
sea Body - open sea NA 0.04335018 0.041500413 -
0.001849767 1.044572255
0.001840404 0.063219697 0.691704597 0.494163519
0.912398273 -7.529955162 cg24759658 12
72233341 q TPH2 TBC1D15 NA TSS200 island
TSS200 - island NA 0.062587058 0.064427463
0.001840404 0.971434464
0.003520616 0.943303733 0.690111221 0.495151387
0.912398273 -7.531071972 cg20806676 6
29579306 p GABBR1/MOG GABBR1 NA Body open
sea Body - open sea NA 0.942093521 0.945614137
0.003520616 0.9962769
-0.003265455 0.898183552 -0.687241901 0.496933104
0.912398273 -7.533076797 cg01095157 2
171784674 q GAD1 GORASP2 NA TSS1500 shore
TSS1500 - shore NA 0.899306052 0.896040597
-0.003265455 1.003644316
-0.012545577 0.537719387 -0.686880054 0.497158048
0.912398273 -7.533329047 cg11182965 22
19864308 q COMT TXNRD2 NA 3'UTR open
sea 3'UTR - open sea NA 0.542031929 0.529486352
-0.012545577 1.023693863

0.001554334 0.029930092 0.686499249 0.49739484
0.912398273 -7.533594375 cg13293756 2
172778961 q SLC25A12 HAT1 NA 1stExon island
1stExon - island NA 0.029395789 0.030950124
0.001554334 0.949779361
0.003704751 0.069627521 0.684454477 0.498667391
0.912398273 -7.535016637 cg01176826 6
29720527 p MOG IFITM4P 1943 IGR island
IGR - island NA 0.068354012 0.072058764
0.003704751 0.948587073
-0.002919619 0.128155055 -0.683684202 0.499147236
0.912398273 -7.535551342 cg23009221 6
152128588 q ESR1 ESR1 NA TSS1500 shore
TSS1500 - shore NA 0.129158674 0.126239056
-0.002919619 1.023127692
0.010449221 0.68430224 0.682798303 0.499699428
0.912398273 -7.536165587 cg12017635 12
72343656 q TPH2 TPH2 NA Body open
sea Body - open sea NA 0.68071032 0.691159541
0.010449221 0.984881608
0.00144221 0.048618168 0.681865855 0.500281001
0.912398273 -7.536811271 cg17016394 21
34442360 q OLIG2 OLIG1 NA TSS200 island
TSS200 - island NA 0.048122408 0.049564618
0.00144221 0.970902429
-0.00270579 0.920775626 -0.681693714 0.500388407
0.912398273 -7.536930378 cg15320474 6
29528005 p GABBR1/MOG UBD NA TSS1500 open
sea TSS1500 - open sea NA 0.921705742 0.918999951
-0.00270579 1.002944278
-0.009644151 0.515928058 -0.681656718 0.500411493
0.912398273 -7.536955973 cg23601416 22
19950040 q COMT COMT NA 5'UTR open
sea 5'UTR - open sea NA 0.519243234 0.509599084
-0.009644151 1.018924975
0.001744633 0.022322397 0.680379231 0.501208995
0.912398273 -7.537838929 cg06046431 11
27744490 p BDNF BDNF NA TSS1500 island
TSS1500 - island V$P53_01 0.021722679 0.023467312
0.001744633 0.925656888
0.00263477 0.094103796 0.678673224 0.50227511 0.912398273
-7.539015556 cg21950534 6 152128483 q ESR1
ESR1 NA TSS1500 shore TSS1500 - shore NA
0.093198094 0.095832864 0.00263477 0.972506613
0.003254597 0.094548627 0.677293893 0.503137998
0.912398273 -7.539964778 cg12899157 18 3452302
p DLGAP1 TGIF1 NA 5'UTR island 5'UTR -
island NA 0.093429859 0.096684456 0.003254597
0.96633795
0.00352121 0.045564191 0.67727263 0.503151306 0.912398273
-7.539979397 cg00782607 2 171672156 q GAD1
GAD1 NA TSS1500 shore TSS1500 - shore NA
0.044353775 0.047874985 0.00352121 0.926449899
-0.001388173 0.049762346 -0.676063229 0.503908574
0.912398273 -7.540810108 cg11539173 22
19843009 q COMT GNB1L NA TSS1500 island

TSS1500 - island NA 0.050239531 0.048851358
-0.001388173 1.028416262
0.007298087 0.44210548 0.674559088 0.504851273
0.912398273 -7.541841257 cg24547396 22
19928740 q COMT COMT NA TSS1500 shore
TSS1500 - shore NA 0.439596762 0.44689485
0.007298087 0.98366934
-0.007473889 0.210572262 -0.674300045 0.505013722
0.912398273 -7.542018616 cg02723395 2
171678751 q GAD1 GAD1 NA Body island
Body - island NA 0.213141411 0.205667523
-0.007473889 1.036339661
0.003189448 0.923759697 0.674011072 0.505194976
0.912398273 -7.54221639 cg05087623 6
29527870 p GABBR1/MOG UBD NA TSS200 open
sea TSS200 - open sea NA 0.922663324 0.925852772
0.003189448 0.996555124
-0.004510532 0.845766498 -0.67347701 0.505530052
0.912398273 -7.542581686 cg00721193 22
19966373 q COMT ARVCF NA Body shore
Body - shore NA 0.847316993 0.842806462
-0.004510532 1.005351799
-0.009063952 0.802636866 -0.672749042 0.505986983
0.912398273 -7.543079158 cg08451469 6
29711588 p MOG LOC285830 NA Body open
sea Body - open sea NA 0.805752599 0.796688647
-0.009063952 1.011377032
0.002753527 0.046867941 0.671771622 0.506600849
0.912398273 -7.543746278 cg21956337 15
88799707 q NTRK3 NTRK3 NA TSS200 island
TSS200 - island NA 0.045921416 0.048674943
0.002753527 0.943430298
-0.00624326 0.878701669 -0.670676041 0.507289413
0.912398273 -7.544492925 cg18217459 6
152429948 q ESR1 SYNE1 -12871 IGR open
sea IGR - open sea NA 0.88084779 0.87460453 -0.00624326
1.007138381
-0.004846651 0.12531471 -0.669488981 0.508036052
0.912398273 -7.54530058 cg25166896 22
20009063 q COMT C22orf25 NA 5'UTR island
5'UTR - island NA 0.126980747 0.122134095
-0.004846651 1.039683039
-0.001598384 0.037164507 -0.669426477 0.508075382
0.912398273 -7.545343067 cg05200610 12
72233572 q TPH2 TBC1D15 NA 1stExon island
1stExon - island V$AP2_Q6;V$FAC1_01 0.037713951
0.036115567 -0.001598384 1.04425748
-0.005660227 0.798883213 -0.667095944 0.509543063
0.912398273 -7.54692453 cg06477632 6
29526338 p GABBR1/MOG UBD NA Body open
sea Body - open sea NA 0.800828916 0.795168689
-0.005660227 1.007118272
0.005649982 0.869571151 0.666590924 0.509861412
0.912398273 -7.547266522 cg16834011 22
19931790 q COMT COMT NA 5'UTR shelf
5'UTR - shelf NA 0.867628969 0.873278952
0.005649982 0.993530151

-0.003527429 0.10240968 -0.666571284 0.509873794
0.912398273 -7.547279816 cg05171584 6
152128535 q ESR1 ESR1 NA TSS1500 shore
TSS1500 - shore NA 0.103622234 0.100094805
-0.003527429 1.03524088
0.002291009 0.045633191 0.665952203 0.510264198
0.912398273 -7.547698693 cg06879567 18 3594243
p DLGAP1 DLGAP1 NA Body open sea Body -
open sea V$HTF_01;V$RFX1_01 0.044845656 0.047136665
0.002291009 0.951396455
-0.002019433 0.062855355 -0.664663204 0.511077588
0.912398273 -7.548569628 cg01963885 22
20004367 q COMT ARVCF NA TSS200 island
TSS200 - island NA 0.063549535 0.061530102
-0.002019433 1.032820245
-0.005248348 0.156304229 -0.663904574 0.511556634
0.912398273 -7.549081443 cg27293992 2
171671648 q GAD1 GAD1 -1552 IGR island
IGR - island NA 0.158108349 0.152860001
-0.005248348 1.034334345
-0.003882808 0.890051235 -0.66055623 0.513673917
0.912448438 -7.551333635 cg21905167 22
19949585 q COMT COMT NA 5'UTR open
sea 5'UTR - open sea NA 0.89138595 0.887503142 -
0.003882808 1.004374979
0.005234011 0.157756675 0.660486356 0.513718152
0.912448438 -7.551380516 cg21670199 15
88801401 q NTRK3 NTRK3-AS1 5440 IGR shore
IGR - shore V$AHRARNT_02 0.155957483
0.161191494 0.005234011 0.967529236
0.00418962 0.882989589 0.659974902 0.514041999
0.912448438 -7.551723524 cg02373484 6
29631227 p GABBR1/MOG MOG NA Body open
sea Body - open sea NA 0.881549407 0.885739027
0.00418962 0.995269916
-0.00360348 0.796502996 -0.659178727 0.51454635
0.912448438 -7.552256967 cg13224710 18 3411743
p DLGAP1 TGIF1 NA TSS1500 open sea TSS1500
- open sea NA 0.797741692 0.794138212 -0.00360348
1.004537598
-0.00360879 0.9202827 -0.658589573 0.514919733
0.912448438 -7.5526513 cg07169712 6 29571419 p
GABBR1/MOG GABBR1 NA Body open sea Body - open sea
V$RREB1_01;V$TAXCREB_01 0.921523221 0.917914432
-0.00360879 1.003931509
0.00276305 0.08600405 0.653382811 0.51822598 0.914065584 -
7.556121385 cg03167496 11 27743619 p BDNF BDNF
NA TSS200 island TSS200 - island NA
0.085054251 0.087817302 0.00276305 0.968536371
0.001330831 0.048101974 0.653152933 0.518372215
0.914065584 -7.556273971 cg12312205 18 3594173
p DLGAP1 DLGAP1 NA Body open sea Body -
open sea NA 0.047644501 0.048975332 0.001330831
0.972826504
0.002294169 0.058098262 0.653039269 0.51844453
0.914065584 -7.556349398 cg25328597 11
27722638 p BDNF BDNF NA TSS200 island

TSS200 - island NA 0.057309641 0.05960381
0.002294169 0.961509692
0.002713478 0.105918879 0.652944168 0.518505039
0.914065584 -7.556412497 cg02858594 21
34400211 q OLIG2 OLIG2 NA 3'UTR island
3'UTR - island NA 0.104986121 0.107699599
0.002713478 0.974805124
0.001582889 0.046173017 0.649350319 0.520794459
0.915984677 -7.558790446 cg23105568 6
29617607 p GABBR1/MOG MOG -7151 IGR shore
IGR - shore NA 0.045628899 0.047211788
0.001582889 0.966472589
0.001277833 0.033800729 0.649133421 0.520932806
0.915984677 -7.558933552 cg21241839 6
29716536 p MOG LOC285830 NA Body island
Body - island NA 0.033361473 0.034639306
0.001277833 0.963110318
-0.001787876 0.072978115 -0.646416901 0.522667197
0.916081074 -7.560721934 cg20104535 2
172750996 q SLC25A12 SLC25A12 NA TSS200 island
TSS200 - island NA 0.073592698 0.071804822
-0.001787876 1.024899108
-0.001364162 0.037556232 -0.645421495 0.523303502
0.916081074 -7.561375416 cg02954262 18 3451750
p DLGAP1 TGIF1 NA 5'UTR island 5'UTR -
island NA 0.038025163 0.036661001 -0.001364162
1.037210168
-0.004991965 0.79923571 -0.642557549 0.525136576
0.916081074 -7.56325012 cg21297992 22
19961060 q COMT ARVCF NA Body island
Body - island NA 0.800951698 0.795959733
-0.004991965 1.00627163
0.001653656 0.035459325 0.641722212 0.525671883
0.916081074 -7.563795391 cg26057780 11
27721277 p BDNF BDNF NA Body shore
Body - shore NA 0.034890881 0.036544537
0.001653656 0.95474957
0.001145832 0.033447553 0.641227397 0.525989112
0.916081074 -7.564118059 cg13344806 18 3450921
p DLGAP1 TGIF1 NA 5'UTR island 5'UTR -
island NA 0.033053673 0.034199505 0.001145832
0.966495655
-0.005950174 0.785508777 -0.64090278 0.526197282
0.916081074 -7.56432961 cg13210820 6
29632605 p GABBR1/MOG MOG NA 3'UTR open
sea 3'UTR - open sea NA 0.787554149 0.781603975
-0.005950174 1.007612773
-0.001906545 0.025982667 -0.640461107 0.526480588
0.916081074 -7.564617277 cg25381667 11
27743651 p BDNF BDNF NA TSS200 island
TSS200 - island NA 0.026638042 0.024731497
-0.001906545 1.077089753
-0.001953604 0.897300236 -0.639559014 0.527059478
0.916081074 -7.56520422 cg13908968 9
87188847 q NTRK2 NTRK2 -94619 IGR open
sea IGR - open sea NA 0.897971788 0.896018184
-0.001953604 1.002180317

-0.001848264 0.05480628 -0.638763106 0.527570511
0.916081074 -7.565721406 cg13974632 11
27740813 p BDNF BDNF NA Body island
Body - island NA 0.055441621 0.053593357
-0.001848264 1.034486811
-0.003403858 0.910042453 -0.638073694 0.528013378
0.916081074 -7.566168882 cg03724721 22
19939061 q COMT COMT NA 5'UTR open
sea 5'UTR - open sea NA 0.91121253 0.907808671 -
0.003403858 1.003749533
0.00375837 0.897442716 0.637373529 0.528463357
0.916081074 -7.566622856 cg24937995 21
34448038 q OLIG2 OLIG1 5588 IGR shelf
IGR - shelf NA 0.896150776 0.899909146
0.00375837 0.995823612
0.003183344 0.875221684 0.636502505 0.529023427
0.916081074 -7.567186934 cg12867320 6
29574715 p GABBR1/MOG GABBR1 NA Body open
sea Body - open sea V$TAL1ALPHAE47_01 0.87412741 0.877310754
0.003183344 0.996371475
0.002695918 0.050760632 0.634519608 0.530299615
0.916854547 -7.56846826 cg03152033 18 4453856
p DLGAP1 DLGAP1-AS5 189254 IGR shore IGR -
shore NA 0.04983391 0.052529828 0.002695918
0.948678339
0.001946305 0.044499357 0.632742164 0.531444965
0.916854547 -7.569613512 cg13355041 18 3593715
p DLGAP1 FLJ35776 NA TSS1500 open sea TSS1500
- open sea V$P53_01 0.043830314 0.045776619 0.001946305
0.957482552
-0.00524499 0.854066278 -0.632686674 0.531480742
0.916854547 -7.569649215 cg14447193 9
87433864 q NTRK2 NTRK2 NA Body open
sea Body - open sea NA 0.855869243 0.850624253
-0.00524499 1.006166048
0.001920926 0.053259469 0.630014081 0.53320544
0.917517401 -7.571365191 cg02285263 6
152129749 q ESR1 ESR1 NA Body island
Body - island NA 0.052599151 0.054520077
0.001920926 0.964766631
-0.010491115 0.509015299 -0.629277637 0.533681208
0.917517401 -7.571836789 cg03127104 6
29599250 p GABBR1/MOG GABBR1 NA Body shore
Body - shore NA 0.51262162 0.502130505 -
0.010491115 1.020893204
-0.009497163 0.548909196 -0.625851152 0.535897789
0.917517401 -7.574023945 cg22546130 22
19950026 q COMT COMT NA 5'UTR open
sea 5'UTR - open sea NA 0.552173845 0.542676683
-0.009497163 1.01750059
-0.005114737 0.699722845 -0.62555348 0.536090581
0.917517401 -7.574213401 cg22113926 17
28566331 q SLC6A4 BLMH -8882 IGR shelf
IGR - shelf NA 0.701481036 0.696366299
-0.005114737 1.007344895
-0.007244105 0.606112248 -0.624765348 0.536601204
0.917517401 -7.574714592 cg27639046 2

171608303 q GAD1 SP5 36446 IGR open
sea IGR - open sea NA 0.608602409 0.601358304
-0.007244105 1.012046238
0.002892565 0.889748154 0.62396277 0.53712145 0.917517401
-7.575224336 cg00786657 15 88515810 q NTRK3
NTRK3 NA Body open sea Body - open sea NA
0.888753835 0.8916464 0.002892565 0.996755928
0.005102784 0.593146531 0.623773414 0.537244232
0.917517401 -7.575344509 cg06542928 2
171608308 q GAD1 SP5 36451 IGR open
sea IGR - open sea NA 0.591392449 0.596495233
0.005102784 0.99144539
0.008481231 0.792004829 0.623262127 0.537575836
0.917517401 -7.575668815 cg12931591 18 3411821
p DLGAP1 TGIF1 NA TSS1500 open sea TSS1500
- open sea NA 0.789089406 0.797570637 0.008481231
0.989366169
0.006082644 0.831301692 0.620231418 0.539543664
0.917517401 -7.577585845 cg26310969 17
28662096 q SLC6A4 TMIGD1 NA TSS1500 open
sea TSS1500 - open sea NA 0.829210784 0.835293427
0.006082644 0.992717957
0.007351799 0.447672339 0.619967262 0.539715359
0.917517401 -7.5777525 cg16716320 15 88406509 q
NTRK3 NTRK3-AS1 -13479 IGR open sea IGR - open sea
NA 0.445145158 0.452496957 0.007351799
0.983752821
0.004692587 0.834605013 0.618705194 0.540536065
0.917517401 -7.578547782 cg11219691 22
19967280 q COMT ARVCF NA Body island
Body - island NA 0.832991936 0.837684523
0.004692587 0.994398145
0.003766447 0.887010882 0.617204383 0.541512874
0.917517401 -7.579491447 cg18595174 11
27701991 p BDNF BDNF NA Body open
sea Body - open sea NA 0.885716166 0.889482613
0.003766447 0.995765575
0.002446015 0.082103929 0.615452745 0.5426541
0.917517401 -7.580589994 cg21434114 18 3450282
p DLGAP1 TGIF1 NA 5'UTR island 5'UTR -
island NA 0.081263111 0.083709126 0.002446015
0.97077959
0.002886551 0.894196531 0.614738815 0.5431196
0.917517401 -7.581036864 cg14592798 9
87257941 q NTRK2 NTRK2 -25525 IGR open
sea IGR - open sea NA 0.893204279 0.89609083
0.002886551 0.996778729
0.001628799 0.075635841 0.613764185 0.543755419
0.917517401 -7.581646097 cg21133153 6
29521488 p GABBR1 UBD -1901 IGR island
IGR - island NA 0.075075941 0.07670474
0.001628799 0.978765341
-0.004191086 0.879415217 -0.61310068 0.544188491
0.917517401 -7.582060307 cg11147094 15
88420438 q NTRK3 NTRK3 NA Body open
sea Body - open sea NA 0.880855902 0.876664816
-0.004191086 1.004780717

0.002678733 0.065339394 0.612666212 0.544472168
0.917517401 -7.582331299 cg20294320 6
29617586 p GABBR1/MOG MOG -7172 IGR shore
IGR - shore NA 0.064418579 0.067097312
0.002678733 0.960076895
0.008977287 0.870953199 0.609200355 0.546737876
0.917517401 -7.584486344 cg05913325 6
29639793 p GABBR1/MOG MOG NA 3'UTR open
sea 3'UTR - open sea NA 0.867867256 0.876844543
0.008977287 0.989761826
0.003429219 0.093705362 0.608337889 0.547302448
0.917517401 -7.585020764 cg05542661 6
29717068 p MOG LOC285830 NA TSS1500 island
TSS1500 - island NA 0.092526568 0.095955787
0.003429219 0.96426251
-0.001043817 0.019084234 -0.607147354 0.548082273
0.917517401 -7.585757254 cg14583606 9 4490315
p SLC1A1 SLC1A1 NA TSS200 island TSS200
- island NA 0.019443046 0.018399229 -0.001043817
1.056731562
0.001863174 0.063301106 0.606894773 0.548247792
0.917517401 -7.585913324 cg10672884 2
171785479 q GAD1 GORASP2 NA TSS1500 island
TSS1500 - island NA 0.06266064 0.064523814
0.001863174 0.971124243
0.001499552 0.043208063 0.60476149 0.549646791
0.917517401 -7.587228956 cg10190161 2
172778811 q SLC25A12 HAT1 NA TSS200 island
TSS200 - island V$MIF1_01;V$RFX1_01 0.042692592
0.044192144 0.001499552 0.966067453
-0.003970674 0.71520466 -0.604188718 0.550022726
0.917517401 -7.587581424 cg15641340 6
29429909 p GABBR1 OR2H1 NA Body open
sea Body - open sea V$PPARG_01;V$PPARA_01 0.71656958
0.712598906 -0.003970674 1.005572102
0.000951949 0.026963678 0.602091339 0.551400461
0.917517401 -7.588869304 cg04920689 15
88800510 q NTRK3 NTRK3 NA TSS1500 island
TSS1500 - island NA 0.026636445 0.027588394
0.000951949 0.965494584
-0.004097584 0.091958811 -0.599242023 0.553274973
0.917517401 -7.590611894 cg09444802 6
29691312 p GABBR1/MOG HLA-F NA Body island
Body - island NA 0.093367356 0.089269772
-0.004097584 1.045901137
0.005008259 0.822458799 0.598893823 0.553504272
0.917517401 -7.590824292 cg02965092 12
72347052 q TPH2 TPH2 NA Body open
sea Body - open sea NA 0.82073721 0.825745468
0.005008259 0.993934865
-0.001447237 0.048229387 -0.596475059 0.555098436
0.917517401 -7.592296385 cg17221604 6
29720989 p MOG IFITM4P 2405 IGR island
IGR - island NA 0.048726875 0.047279638
-0.001447237 1.030610154
0.004820576 0.842593119 0.595672287 0.555628048
0.917517401 -7.592783675 cg04385220 6

29726705	p	MOG	IFITM4P	8121	IGR	open
sea	IGR - open sea	NA	0.840936046		0.845756622
0.004820576		0.99430028
-0.000981113		0.033456153		-0.593414129		0.557119205
0.917517401		-7.594150958		cg16548348	22
19842481	q	COMT	GNB1L	NA	TSS200	island
TSS200 - island	NA	0.033793411		0.032812298
-0.000981113		1.029900771
0.003456007		0.233956791		0.59332109	0.557180686
0.917517401		-7.594207182		cg02035039	6
29691888	p	GABBR1/MOG	HLA-F	NA	Body	island
Body - island	NA	0.232768788		0.236224795
0.003456007		0.985369838
-0.005641973		0.777652072		-0.591934507		0.558097369
0.917517401		-7.595044093		cg07821417	22
19972146	q	COMT	ARVCF	NA	Body	shelf
Body - shelf	NA	0.779591501		0.773949528
-0.005641973		1.007289846
-0.004528079		0.804546135		-0.591139327		0.558623416
0.917517401		-7.595523183		cg13369999	6
29711465	p	MOG	LOC285830	NA	Body	open
sea	Body - open sea	NA	0.806102662		0.801574583
-0.004528079		1.00564898
0.003122571		0.061178916		0.590654514		0.558944265
0.917517401		-7.595814969		cg06412358	21
34392373	q	OLIG2	OLIG2	-5843	IGR	island
IGR - island	NA	0.060105532		0.063228103
0.003122571		0.950614191
-0.002893212		0.922024036		-0.589035184		0.560016617
0.917517401		-7.596787871		cg15209921	6
29430506	p	GABBR1	OR2H1	NA	3'UTR	open
sea	3'UTR - open sea	NA	0.923018578		0.920125366
-0.002893212		1.003144367
-0.004838526		0.709665929		-0.588464696		0.560394654
0.917517401		-7.597130001		cg13311832	6
29549147	p	GABBR1/MOG	SNORD32B	NA	TSS1500	open
sea	TSS1500 - open sea	NA	0.711329173		0.706490647
-0.004838526		1.006848677
0.003665002		0.877743137		0.588164357		0.560593727
0.917517401		-7.597309988		cg25654517	6
29627131	p	GABBR1/MOG	MOG	NA	Body	open
sea	Body - open sea	NA	0.876483293		0.880148295
0.003665002		0.995835927
-0.005006191		0.880890046		-0.587846509		0.560804445
0.917517401		-7.59750037		cg04615964	6
29591153	p	GABBR1/MOG	GABBR1	NA	Body	open
sea	Body - open sea	NA	0.882610924		0.877604733
-0.005006191		1.00570438
0.005500155		0.068877377		0.587778288		0.560849677
0.917517401		-7.59754122		cg11257193	17
28444188	q	SLC6A4	MIR423	NA	Body	shore
Body - shore	NA	0.066986699		0.072486854
0.005500155		0.924122035
-0.003571268		0.867391217		-0.587536704		0.56100987
0.917517401		-7.597685838		cg26126367	17
28559497	q	SLC6A4	SLC6A4	NA	5'UTR	shelf

5'UTR - shelf NA 0.86861884 0.865047572 -
0.003571268 1.004128406
0.001049201 0.027252794 0.586886771 0.561440949
0.917517401 -7.598074616 cg25990314 22
19841927 q COMT GNB1L NA 5'UTR island
5'UTR - island NA 0.026892132 0.027941333
0.001049201 0.962449859
0.004608943 0.806217287 0.586407184 0.561759151
0.917517401 -7.598361226 cg16902425 6
29526459 p GABBR1/MOG UBD NA Body open
sea Body - open sea NA 0.804632962 0.809241906
0.004608943 0.994304615
-0.009778052 0.784692765 -0.58565362 0.562259319
0.917517401 -7.598811107 cg19409546 12
72477363 q TPH2 TPH2 144737 IGR open
sea IGR - open sea NA 0.788053971 0.778275919
-0.009778052 1.012563734
0.006865637 0.872273591 0.584788216 0.562833998
0.917517401 -7.599327058 cg10122187 22
19938620 q COMT COMT NA 5'UTR open
sea 5'UTR - open sea NA 0.869913528 0.876779165
0.006865637 0.99216948
-0.000939771 0.022579843 -0.584735425 0.562869064
0.917517401 -7.599358508 cg26151531 22
19842652 q COMT GNB1L NA TSS200 island
TSS200 - island NA 0.022902889 0.021963118
-0.000939771 1.042788597
0.003602242 0.894062438 0.584540999 0.562998219
0.917517401 -7.599474311 cg14273545 9
87449508 q NTRK2 NTRK2 NA Body open
sea Body - open sea NA 0.892824167 0.896426409
0.003602242 0.995981553
0.003818375 0.87521744 0.584321099 0.563144314
0.917517401 -7.599605242 cg09043524 22
19891284 q COMT TXNRD2 NA Body shelf
Body - shelf NA 0.873904873 0.877723248
0.003818375 0.995649682
-0.005958515 0.886722656 -0.583954555 0.563387878
0.917517401 -7.599823378 cg09517033 6
29461584 p GABBR1 MAS1L 7041 IGR open
sea IGR - open sea NA 0.888770895 0.88281238 -
0.005958515 1.006749469
0.006261107 0.84216363 0.581621911 0.564939136
0.918949747 -7.601208435 cg00521620 6
29434416 p GABBR1 OR2H1 8186 IGR open
sea IGR - open sea NA 0.840011375 0.846272482
0.006261107 0.992601547
-0.003214147 0.898503016 -0.58036987 0.565772657
0.919212583 -7.601949624 cg23742601 6
29589895 p GABBR1/MOG GABBR1 NA Body open
sea Body - open sea NA 0.899607879 0.896393732
-0.003214147 1.003585642
0.001349944 0.04911828 0.577858337 0.567446523
0.920838485 -7.603431696 cg04378940 6
29617766 p GABBR1/MOG MOG -6992 IGR island
IGR - island NA 0.048654237 0.050004181
0.001349944 0.973003377

0.00400549 0.90123145 0.576106716 0.568615398 0.92164202 -
7.604461615 cg18078658 12 72374129 q TPH2 TPH2
NA Body open sea Body - open sea NA
0.899854562 0.903860052 0.00400549 0.995568462
0.009689561 0.357769475 0.570072641 0.57265121
0.925717771 -7.607986087 cg09740560 6
29602390 p GABBR1/MOG GABBR1 NA TSS1500 shore
TSS1500 - shore NA 0.354438689 0.364128249
0.009689561 0.973389705
0.004006947 0.8434358 0.569942825 0.572738192
0.925717771 -7.608061513 cg25699759 15
88576311 q NTRK3 NTRK3 NA Body open
sea Body - open sea NA 0.842058412 0.846065358
0.004006947 0.995264023
-0.007217573 0.296163896 -0.569313361 0.573160053
0.925717771 -7.608427004 cg13819127 6
29717368 p MOG LOC285830 NA TSS1500 shore
TSS1500 - shore NA 0.298644937 0.291427364
-0.007217573 1.024766284
-0.001772724 0.082234806 -0.567066193 0.57466734
0.92705769 -7.609728569 cg11950383 21 34400072 q
OLIG2 OLIG2 NA Body island Body - island
NA 0.08284418 0.081071456 -0.001772724
1.021866192
0.002328629 0.938657133 0.565463037 0.575743857
0.927700349 -7.610654039 cg14914809 2
171705073 q GAD1 GAD1 NA Body open
sea Body - open sea NA 0.937856667 0.940185296
0.002328629 0.997523223
0.010995768 0.456764016 0.561363403 0.578501284
0.92872505 -7.613008993 cg15094605 6 29429346 p
GABBR1 OR2H1 NA 5'UTR open sea 5'UTR - open sea
NA 0.452984221 0.463979988 0.010995768
0.976301204
-0.002599951 0.068893555 -0.56135176 0.578509124
0.92872505 -7.613015657 cg20927575 6 29716340 p
MOG LOC285830 NA Body shore Body - shore
NA 0.069787288 0.067187337 -0.002599951
1.038697039
-0.006350716 0.907276101 -0.560579903 0.579029008
0.92872505 -7.613457148 cg25978138 6 29648161 p
GABBR1/MOG ZFP57 7992 IGR open sea IGR - open sea
NA 0.909459159 0.903108443 -0.006350716
1.007032064
0.002155672 0.0948546 0.560481401 0.579095371
0.92872505 -7.613513446 cg23221504 2 171673110 q
GAD1 GAD1 NA TSS200 island TSS200 - island
NA 0.094113588 0.09626926 0.002155672
0.977607889
0.004692844 0.834786456 0.559354531 0.579854829
0.928854105 -7.614156818 cg23174322 6
29627215 p GABBR1/MOG MOG NA Body open
sea Body - open sea NA 0.833173291 0.837866135
0.004692844 0.994399053
0.001650197 0.92754841 0.557023752 0.581427218
0.930060522 -7.615483518 cg07119172 17
28512018 q SLC6A4 CCDC55 NA Body open

sea Body - open sea NA 0.926981155 0.928631352
0.001650197 0.998222979
0.007413893 0.72847522 0.554600275 0.583064355
0.930060522 -7.616857222 cg18458352 6
29711249 p MOG LOC285830 NA Body open
sea Body - open sea NA 0.725926695 0.733340587
0.007413893 0.989890247
-0.007501132 0.196732063 -0.553606113 0.583736595
0.930060522 -7.617419045 cg12772565 6
29618315 p GABBR1/MOG MOG -6443 IGR shore
IGR - shore NA 0.199310577 0.191809444
-0.007501132 1.039107214
-0.003983147 0.737290199 -0.553528624 0.583789007
0.930060522 -7.617462794 cg00758854 6
29576422 p GABBR1/MOG GABBR1 NA Body open
sea Body - open sea NA 0.738659406 0.734676259
-0.003983147 1.005421636
-0.032175304 0.700314552 -0.553205928 0.584007301
0.930060522 -7.61764492 cg02157626 6
29648736 p GABBR1/MOG ZFP57 8567 IGR open
sea IGR - open sea NA 0.711374813 0.679199508
-0.032175304 1.047372392
0.003671284 0.838117752 0.550427448 0.585888502
0.930421896 -7.619208746 cg13570637 22
20052305 q COMT C22orf25 NA 3'UTR open
sea 3'UTR - open sea NA 0.836855748 0.840527032
0.003671284 0.995632164
0.001584051 0.083802145 0.550190945 0.586048766
0.930421896 -7.619341502 cg16728223 6
29691603 p GABBR1/MOG HLA-F NA Body island
Body - island NA 0.083257627 0.084841678
0.001584051 0.98132933
-0.001254441 0.038642456 -0.549268893 0.586673785
0.930421896 -7.61985854 cg10694442 22
20004377 q COMT ARVCF NA TSS200 island
TSS200 - island V$SP1_01 0.03907367 0.037819229 -
0.001254441 1.033169396
-0.002029611 0.922062717 -0.547435106 0.587917792
0.930421896 -7.620884302 cg11912202 21
34350875 q OLIG2 OLIG2 -47341 IGR island
IGR - island NA 0.922760395 0.920730785
-0.002029611 1.002204347
-0.001891881 0.062528639 -0.547314598 0.587999587
0.930421896 -7.620951592 cg02100602 21
34396665 q OLIG2 OLIG2 -1551 IGR island
IGR - island NA 0.063178973 0.061287092
-0.001891881 1.030869159
0.007445126 0.278888992 0.544466092 0.589934621
0.930421896 -7.622537937 cg12749246 6
29521013 p GABBR1 UBD -2376 IGR shore
IGR - shore NA 0.276329729 0.283774856
0.007445126 0.973763965
0.003551842 0.848399092 0.544412951 0.589970749
0.930421896 -7.622567454 cg15400220 6
29712541 p MOG LOC285830 NA Body shelf
Body - shelf NA 0.847178146 0.850729988
0.003551842 0.995824948

0.003463737		0.873209972		0.544188823		0.590123138
	0.930421896		-7.622691915		cg10477592	21
	34406589	q		OLIG2	OLIG2	8373	IGR	shore
	IGR - shore		NA		0.872019312		0.875483049
	0.003463737		0.996043628
0.003365622		0.9013508	0.539874	0.593060558		0.930421896
	-7.625078185		cg13690679	17	28622405	q		SLC6A4
	TMIGD1	-20961	IGR	shelf	IGR - shelf		NA
	0.900193867		0.903559489		0.003365622
	0.996275152
-0.004172954		0.882394044		-0.53974379		0.593149311
	0.930421896		-7.625149906		cg19931596	2
	172645256	q		SLC25A12	SLC25A12	NA	Body	open
sea	Body - open sea		NA	0.883828497		0.879655543
	-0.004172954		1.00474385
-0.003315489		0.885406716		-0.537245785		0.594853213
	0.930421896		-7.626522553		cg03215160	6
	29424926	p		GABBR1	OR2H1	NA	TSS1500	open
sea	TSS1500 - open sea	NA	0.886546415		0.883230927
	-0.003315489		1.003753818
0.001737701		0.052571691		0.536325637		0.59548144
	0.930421896		-7.627026596		cg07813142	2
	171573223	q		GAD1	SP5	NA	Body	island
	Body - island		NA	0.051974357		0.053712057
	0.001737701		0.96764786
0.003136235		0.159594878		0.534558342		0.596688944
	0.930421896		-7.627992315		cg18264657	18	4455862
	p	DLGAP1	DLGAP1-AS5	191260	IGR	shore	IGR -
shore	NA	0.158516797		0.161653032		0.003136235
	0.980598972
-0.006014219		0.109436968		-0.534373814		0.59681509
	0.930421896		-7.628092967		cg20927242	6
	29692011	p		GABBR1/MOG	HLA-F	NA	Body	island
	Body - island		NA	0.111504356		0.105490136
	-0.006014219		1.057012155
-0.002765468		0.169002894		-0.533995196		0.597073958
	0.930421896		-7.628299382		cg26949694	11
	27742060	p		BDNF	BDNF	NA	Body	island
	Body - island		NA	0.169953523		0.167188055
	-0.002765468		1.016541062
0.00311543	0.849959316		0.533842472		0.597178393
	0.930421896		-7.628382602		cg00542638	22
	20007500	q		COMT	C22orf25	NA	TSS1500	shore
	TSS1500 - shore		NA	0.848888387		0.852003816
	0.00311543	0.99634341
-0.001143922		0.019331999		-0.532970453		0.597774863
	0.930421896		-7.628857328		cg09029526	6
	29720653	p		MOG	IFITM4P	2069	IGR	island
	IGR - island		NA	0.019725222		0.018581301
	-0.001143922		1.06156302
-0.003416415		0.893296309		-0.531907395		0.598502388
	0.930421896		-7.629435024		cg04957663	6
	29587487	p		GABBR1/MOG	GABBR1	NA	Body	open
sea	Body - open sea		NA	0.894470702		0.891054287
	-0.003416415		1.003834127
0.004059955		0.298453797		0.531228324		0.598967345
	0.930421896		-7.629803457		cg03443455	2

171671795 q GAD1 GAD1 NA TSS1500 island
TSS1500 - island NA 0.297058188 0.301118143
0.004059955 0.986517069
0.002061716 0.066184461 0.531114647 0.599045196
0.930421896 -7.629865088 cg03448612 2
171672694 q GAD1 GAD1 NA TSS1500 island
TSS1500 - island NA 0.065475746 0.067537461
0.002061716 0.969473016
0.00498878 0.874021331 0.53101833 0.599111161 0.930421896
-7.629917297 cg14317321 6 29700690 p MOG
LOC285830 NA Body open sea Body - open sea NA
0.872306438 0.877295218 0.00498878 0.994313454
0.004899081 0.168121589 0.530279256 0.599617454
0.930421896 -7.630317604 cg20386586 18 3449692
p DLGAP1 TGIF1 NA 5'UTR island 5'UTR -
island NA 0.16643753 0.171336611 0.004899081
0.971406689
-0.001152378 0.063728119 -0.527293437 0.601664913
0.930421896 -7.631929247 cg17302062 18 3451564
p DLGAP1 TGIF1 NA 5'UTR island 5'UTR -
island V$P53_01 0.064124249 0.062971871 -0.001152378
1.018299885
-0.001812389 0.023037736 -0.526349939 0.602312583
0.930421896 -7.632436657 cg15126544 2
171678954 q GAD1 GAD1 NA Body island
Body - island NA 0.023660745 0.021848356
-0.001812389 1.082953106
0.001061964 0.033505049 0.523056948 0.604575649
0.930421896 -7.634200614 cg23399426 2
171627790 q GAD1 GAD1 -45410 IGR island
IGR - island NA 0.033139999 0.034201963
0.001061964 0.968950203
0.002092546 0.094528667 0.522865367 0.604707434
0.930421896 -7.634302904 cg14384532 15
88800624 q NTRK3 NTRK3 NA TSS1500 island
TSS1500 - island NA 0.093809354 0.0959019
0.002092546 0.978180349
-0.001881888 0.061373221 -0.522041602 0.605274239
0.930421896 -7.634742311 cg04630810 9 4662735
p SLC1A1 C9orf68 NA Body island Body -
island NA 0.06202012 0.060138231 -0.001881888
1.031292723
-0.003442377 0.181426903 -0.519274488 0.60718002
0.930421896 -7.636213339 cg05012697 15
88798331 q NTRK3 NTRK3 NA Body shore
Body - shore NA 0.18261022 0.179167843 -
0.003442377 1.019213141
-0.002392282 0.905869008 -0.518234639 0.607896916
0.930421896 -7.636764148 cg26920808 12
72237548 q TPH2 TBC1D15 NA Body shelf
Body - shelf NA 0.906691355 0.904299073
-0.002392282 1.002645454
-0.002372082 0.902173392 -0.518084963 0.608000138
0.930421896 -7.636843341 cg03175417 22
20050298 q COMT C22orf25 NA Body open
sea Body - open sea NA 0.902988795 0.900616713
-0.002372082 1.002633842

-0.004443498 0.137086013 -0.516152596 0.609333511
0.930421896 -7.637863744 cg06085713 18 3453726
p DLGAP1 TGIF1 NA 5'UTR shore 5'UTR -
shore NA 0.138613465 0.134169967 -0.004443498
1.033118425
-0.001108635 0.023798006 -0.516144748 0.609338929
0.930421896 -7.63786788 cg02149189 6
29521138 p GABBR1 UBD -2251 IGR island
IGR - island NA 0.0241791 0.023070465 -
0.001108635 1.048054298
0.009464673 0.522151394 0.515616276 0.609703825
0.930421896 -7.638146287 cg18731680 22
19953712 q COMT COMT NA Body open
sea Body - open sea NA 0.518897912 0.528362585
0.009464673 0.982086784
-0.008036546 0.441649794 -0.515371509 0.609872864
0.930421896 -7.638275139 cg20265360 2
172756307 q SLC25A12 HAT1 -22628 IGR open
sea IGR - open sea NA 0.444412356 0.43637581 -
0.008036546 1.018416571
-0.002409635 0.041480457 -0.511640926 0.612451948
0.930421896 -7.64023157 cg24065044 11
27723409 p BDNF BDNF NA TSS1500 shore
TSS1500 - shore NA 0.042308769 0.039899133
-0.002409635 1.060393192
0.001151429 0.052888218 0.511058506 0.612855051
0.930421896 -7.640535747 cg12217400 6
29521604 p GABBR1 UBD -1785 IGR island
IGR - island NA 0.052492415 0.053643844
0.001151429 0.978535673
0.001665541 0.065234604 0.510940953 0.612936427
0.930421896 -7.6405971 cg17514757 6 29720651 p
MOG IFITM4P 2067 IGR island IGR - island
NA 0.064662074 0.066327615 0.001665541
0.974889177
0.001143118 0.042495749 0.510368997 0.613332432
0.930421896 -7.640895413 cg16017358 2
172779282 q SLC25A12 HAT1 NA Body island
Body - island NA 0.042102802 0.043245921
0.001143118 0.97356701
0.002970454 0.068029407 0.51005301 0.613551262
0.930421896 -7.641060079 cg25412831 11
27742138 p BDNF BDNF NA Body island
Body - island NA 0.067008313 0.069978767
0.002970454 0.957552067
-0.013070016 0.531833777 -0.509737328 0.613769918
0.930421896 -7.641224488 cg15331332 6
29692111 p GABBR1/MOG HLA-F NA Body shore
Body - shore NA 0.536326595 0.52325658 -
0.013070016 1.024978214
0.007592462 0.148739447 0.509401085 0.614002854
0.930421896 -7.641399494 cg01089319 2
171676809 q GAD1 GAD1 NA Body island
Body - island NA 0.146129538 0.153722001
0.007592462 0.950609132
0.001993022 0.076083523 0.509116286 0.614200184
0.930421896 -7.641547636 ch.2.171328329F 2

171620083 q GAD1 SP5 48226 IGR open
sea IGR - open sea NA 0.075398422 0.077391444
0.001993022 0.974247515
0.00107968 0.040419028 0.508205026 0.614831769
0.930421896 -7.642021093 cg14420670 6
29617961 p GABBR1/MOG MOG -6797 IGR island
IGR - island NA 0.040047888 0.041127568
0.00107968 0.973748022
0.002524023 0.080268676 0.50807932 0.614918918
0.930421896 -7.642086339 cg25980242 18 4454472
p DLGAP1 DLGAP1-AS5 189870 IGR island IGR -
island NA 0.079401043 0.081925066 0.002524023
0.969191078
0.009010971 0.457744869 0.506781037 0.61581932
0.930421896 -7.642759273 cg06591579 6
29461269 p GABBR1 MAS1L 6726 IGR open
sea IGR - open sea NA 0.454647348 0.463658319
0.009010971 0.980565493
0.005679324 0.540138782 0.505932025 0.616408466
0.930421896 -7.643198422 cg11834473 6
29591753 p GABBR1/MOG GABBR1 NA Body shelf
Body - shelf NA 0.538186514 0.543865838
0.005679324 0.989557491
0.001682018 0.122280825 0.502234398 0.618977338
0.930421896 -7.645102559 cg14372466 18 3448468
p DLGAP1 TGIF1 NA 5'UTR island 5'UTR -
island NA 0.121702631 0.123384649 0.001682018
0.986367688
0.002063142 0.917154756 0.500648751 0.620080442
0.930421896 -7.645914895 cg05157433 6
29527885 p GABBR1/MOG UBD NA TSS200 open
sea TSS200 - open sea NA 0.916445551 0.918508693
0.002063142 0.997753813
-0.001401914 0.039320262 -0.500428424 0.62023379
0.930421896 -7.64602757 cg18699287 6
29721001 p MOG IFITM4P 2417 IGR island
IGR - island NA 0.03980217 0.038400256 -
0.001401914 1.036507934
0.001110149 0.051155707 0.499797273 0.620673169
0.930421896 -7.646350069 cg19862860 22
20008420 q COMT C22orf25 NA TSS1500 island
TSS1500 - island NA 0.050774093 0.051884242
0.001110149 0.978603349
-0.003011842 0.904491202 -0.499743716 0.620710459
0.930421896 -7.646377416 cg02404255 6
152419175 q ESR1 ESR1 NA Body open
sea Body - open sea NA 0.905526523 0.902514681
-0.003011842 1.003337167
-0.001269149 0.025937209 -0.499354216 0.62098169
0.930421896 -7.646576218 cg15395148 6
29720485 p MOG IFITM4P 1901 IGR island
IGR - island NA 0.026373479 0.02510433 -
0.001269149 1.050554984
-0.003470006 0.824114235 -0.498520617 0.621562352
0.930421896 -7.647001177 cg03160788 6
29572219 p GABBR1/MOG GABBR1 NA Body open

sea	Body - open sea	NA	0.825307049	0.821837043
	-0.003470006	1.004222256
0.002377419	0.911296011	0.496697203	0.622833354
	0.930421896	-7.647928294	cg00145961 15
	88576296	q	NTRK3	NTRK3	NA	Body	open
sea	Body - open sea	NA	0.910478774	0.912856193
	0.002377419	0.997395626
0.00106216 0.040897683	0.494506023	0.624362264
	0.930421896	-7.649037978	cg17597787 2
	171785529 q	GAD1	GORASP2	NA	TSS200	island
	TSS200 - island	NA	0.040532566	0.041594726
	0.00106216 0.97446407
0.003168123	0.2115333 0.494098054	0.624647115
	0.930421896	-7.649244053	cg25565730 6
	152085565 q	ESR1	ESR1	NA	5'UTR	open
sea	5'UTR - open sea	NA	0.210444258	0.213612381
	0.003168123	0.985168823
-0.003421243	0.865800778	-0.492247004	0.625940286
	0.930421896	-7.650176959	cg09721630 6
	29523835	p	GABBR1	UBD	NA	Body	shelf
	Body - shelf	NA	0.86697683 0.863555587	-
0.003421243	1.00396181
-0.002313249	0.081672066	-0.49092833	0.626862268
	0.930421896	-7.650839452	cg09539438 9
	87283789	q	NTRK2	NTRK2	NA	TSS1500	island
	TSS1500 - island	V$MEIS1AHOXA9_01	0.082467246
	0.080153996	-0.002313249	1.028860071
0.001079656	0.024283198	0.488200799	0.628771223
	0.930421896	-7.652204189	cg04661674 6
	29596874	p	GABBR1/MOG GABBR1	NA	TSS1500	shore
	TSS1500 - shore	V$BRACH_01 0.023912067	0.024991722
	0.001079656	0.956799495
0.001315079	0.036082547	0.487605134	0.629188466
	0.930421896	-7.652501238	cg02720618 6
	152129791 q	ESR1	ESR1	NA	Body	shore
	Body - shore	NA	0.035630489	0.036945568
	0.001315079	0.964404959
0.002933289	0.099137007	0.486776886	0.629768833
	0.930421896	-7.652913678	cg07671949 6
	152128338 q	ESR1	ESR1	NA	TSS1500	shore
	TSS1500 - shore	V$OCT1_02	0.098128689	0.101061978
	0.002933289	0.970975345
0.001762295	0.078046519	0.486057158	0.630273352
	0.930421896	-7.653271518	cg06816235 11
	27742219	p	BDNF	BDNF	NA	Body	island
	Body - island	NA	0.07744073 0.079203025
	0.001762295	0.97774965
-0.001997677	0.062360275	-0.485216791	0.630862665
	0.930421896	-7.653688677	cg21433429 18	4455713
	p	DLGAP1	DLGAP1-AS5 191111	IGR	shore	IGR -
shore	NA	0.063046976	0.0610493	-0.001997677
	1.032722341
0.003141375	0.893880221	0.484758597	0.631184081
	0.930421896	-7.653915826	cg26371172 6
	29454888	p	GABBR1	MAS1L	NA	1stExon	open
sea	1stExon - open sea	NA	0.892800374	0.895941749
	0.003141375	0.996493773

0.006562935 0.500492457 0.484748293 0.631191311
0.930421896 -7.653920932 cg12349676 21
34350934 q OLIG2 OLIG2 -47282 IGR island
IGR - island NA 0.498236448 0.504799383
0.006562935 0.986998924
0.001437967 0.047133062 0.483873427 0.631805221
0.930421896 -7.654354049 cg17911882 9 4679326
p SLC1A1 CDC37L1 NA TSS1500 island TSS1500
- island NA 0.04663876 0.048076728 0.001437967
0.970090144
0.005854063 0.843282 0.483534712 0.632042976
0.930421896 -7.654521528 cg24938286 6
29631295 p GABBR1/MOG MOG NA Body open
sea Body - open sea NA 0.841269666 0.847123728
0.005854063 0.993089484
-0.004859062 0.875273312 -0.483477487 0.632083148
0.930421896 -7.654549812 cg04337734 2
172734703 q SLC25A12 SLC25A12 NA Body open
sea Body - open sea NA 0.876943615 0.872084553
-0.004859062 1.005571779
-0.00281156 0.873380181 -0.483236902 0.632252051
0.930421896 -7.654668686 cg00224929 2
171705463 q GAD1 GAD1 NA Body open
sea Body - open sea NA 0.874346655 0.871535095
-0.00281156 1.003225986
-0.003443646 0.912541192 -0.48307357 0.63236673
0.930421896 -7.654749357 cg27012424 18 3773224
p DLGAP1 DLGAP1 NA Body shore Body -
shore NA 0.913724946 0.9102813 -0.003443646
1.003783057
0.001962413 0.92988233 0.481185014 0.633693398
0.930421896 -7.655680168 cg13427473 15
88406836 q NTRK3 NTRK3-AS1 -13152 IGR open
sea IGR - open sea NA 0.929207751 0.931170164
0.001962413 0.99789253
-0.003299056 0.278137401 -0.477921374 0.635988942
0.930421896 -7.657280244 cg18397357 17
28565258 q SLC6A4 BLMH -9955 IGR shelf
IGR - shelf NA 0.279271451 0.275972395
-0.003299056 1.011954297
-0.002819088 0.892676117 -0.477410996 0.636348259
0.930421896 -7.657529499 cg21919834 22
19948833 q COMT COMT NA 5'UTR open
sea 5'UTR - open sea NA 0.893645178 0.890826091
-0.002819088 1.003164576
0.001062344 0.029703772 0.476542915 0.636959612
0.930421896 -7.657952842 cg15555970 18 3452317
p DLGAP1 TGIF1 NA 5'UTR island 5'UTR -
island NA 0.029338591 0.030400935 0.001062344
0.965055548
0.005760323 0.580514457 0.475461344 0.637721678
0.930421896 -7.658479238 cg00094412 6
29592854 p GABBR1/MOG GABBR1 NA Body shelf
Body - shelf NA 0.578534346 0.584294669
0.005760323 0.990141408
-0.001872325 0.095455974 -0.475384985 0.637775496
0.930421896 -7.658516357 cg04408897 6

29596901 p GABBR1/MOG GABBR1 NA TSS1500 shore
TSS1500 - shore NA 0.096099585 0.09422726 -
0.001872325 1.019870311
0.003150997 0.165490961 0.475278227 0.637850741
0.930421896 -7.658568243 cg15462887 11
27744049 p BDNF BDNF NA TSS1500 island
TSS1500 - island NA 0.164407806 0.167558802
0.003150997 0.981194685
0.002050324 0.923905134 0.474583832 0.638340262
0.930421896 -7.658905452 cg00041368 18 3879131
p DLGAP1 DLGAP1 NA 1stExon shore 1stExon
- shore NA 0.923200335 0.925250659 0.002050324
0.997784034
0.001394574 0.052157506 0.473144883 0.63935519
0.930421896 -7.659602681 cg27280904 21
34443601 q OLIG2 OLIG1 NA 1stExon island
1stExon - island NA 0.051678121 0.053072695
0.001394574 0.973723324
0.001656961 0.932086664 0.472943682 0.639497158
0.930421896 -7.659700004 cg24952408 6
29696264 p MOG LOC285830 NA Body open
sea Body - open sea NA 0.931517083 0.933174044
0.001656961 0.998224382
-0.002662118 0.91514862 -0.472674372 0.639687207
0.930421896 -7.659830209 cg10591943 17
28447544 q SLC6A4 CCDC55 NA Body shelf
Body - shelf NA 0.916063723 0.913401605
-0.002662118 1.00291451
0.001329996 0.074822086 0.472223949 0.64000512
0.930421896 -7.660047815 cg03538731 18 3451548
p DLGAP1 TGIF1 NA 5'UTR island 5'UTR -
island NA 0.0743649 0.075694895 0.001329996
0.982429528
-0.004425197 0.922974185 -0.469720148 0.641773589
0.93200241 -7.661253706 cg25927551 6 29707293 p
MOG LOC285830 NA Body open sea Body - open sea
NA 0.924495346 0.920070149 -0.004425197
1.00480963
0.002093162 0.907001377 0.468269087 0.64279947
0.932502306 -7.661949678 cg05157371 6
29588894 p GABBR1/MOG GABBR1 NA Body open
sea Body - open sea NA 0.906281852 0.908375014
0.002093162 0.997695707
0.003394277 0.06526723 0.466741599 0.643880155
0.933080563 -7.662680013 cg15899474 2
172779515 q SLC25A12 HAT1 NA Body shore
Body - shore NA 0.064100448 0.067494724
0.003394277 0.949710499
0.001090126 0.045840607 0.464765779 0.645279202
0.934118464 -7.663621216 cg03539474 2
172751088 q SLC25A12 SLC25A12 NA TSS1500 shore
TSS1500 - shore NA 0.045465876 0.046556002
0.001090126 0.97658463
0.003040745 0.865669661 0.458681845 0.649595393
0.939050026 -7.666494625 cg15154411 6
29576987 p GABBR1/MOG GABBR1 NA Body open

sea Body - open sea NA 0.864624405 0.86766515
0.003040745 0.996495486
0.002109408 0.077737802 0.458029747 0.650058754
0.939050026 -7.66680039 cg22124493 6
29717032 p MOG LOC285830 NA TSS1500 island
TSS1500 - island NA 0.077012694 0.079122101
0.002109408 0.973339851
0.001028542 0.015775968 0.453398137 0.653353905
0.939824325 -7.668959776 cg16525287 6
29720702 p MOG IFITM4P 2118 IGR island
IGR - island NA 0.015422407 0.016450949
0.001028542 0.937478257
-0.003757468 0.788986217 -0.453120165 0.653551894
0.939824325 -7.669088686 cg10781870 17
28648320 q SLC6A4 TMIGD1 NA Body open
sea Body - open sea NA 0.790277846 0.786520378
-0.003757468 1.004777331
-0.004125074 0.167622617 -0.452617418 0.653910047
0.939824325 -7.669321636 cg21446343 22
19974866 q COMT ARVCF NA Body shore
Body - shore NA 0.169040611 0.164915537
-0.004125074 1.025013253
0.002735335 0.094709357 0.452560437 0.653950645
0.939824325 -7.669348022 cg16164802 6
29691808 p GABBR1/MOG HLA-F NA Body island
Body - island NA 0.093769086 0.096504421
0.002735335 0.971655858
-0.004003608 0.179447461 -0.451859629 0.654450048
0.939824325 -7.669672279 cg08729810 21
34396944 q OLIG2 OLIG2 NA TSS1500 island
TSS1500 - island NA 0.180823701 0.176820093
-0.004003608 1.022642268
-0.003463727 0.854995362 -0.449726616 0.65597105
0.939824325 -7.670656149 cg02139827 21
34404457 q OLIG2 OLIG2 6241 IGR shore
IGR - shore NA 0.856186019 0.852722291
-0.003463727 1.004061965
0.006731032 0.377831162 0.449651784 0.656024438
0.939824325 -7.670690582 cg16289618 6
29705939 p MOG LOC285830 NA Body open
sea Body - open sea NA 0.375517369 0.382248401
0.006731032 0.982390948
-0.001012113 0.039590108 -0.44924824 0.656312374
0.939824325 -7.670876173 cg01075763 18 3449181
p DLGAP1 TGIF1 NA 5'UTR island 5'UTR -
island V$FOXJ2_01;V$HFH3_01 0.039938022 0.038925909 -
0.001012113 1.026001011
-0.001199369 0.049603275 -0.446942801 0.657958374
0.939824325 -7.671933293 cg21709140 2
171785487 q GAD1 GORASP2 NA TSS1500 island
TSS1500 - island NA 0.050015558 0.04881619 -
0.001199369 1.024569062
-0.001863351 0.089510722 -0.446719276 0.658118055
0.939824325 -7.672035501 cg05929831 6
29720460 p MOG IFITM4P 1876 IGR island
IGR - island NA 0.090151249 0.088287898
-0.001863351 1.021105395

-0.002286465 0.904912501 -0.446672 0.65815183 0.939824325
-7.672057111 cg13599596 9 4541807 p SLC1A1
SLC1A1 NA Body open sea Body - open sea NA
0.905698473 0.903412008 -0.002286465
1.002530922
-0.002536988 0.91002364 -0.441715254 0.661697086
0.94333036 -7.674310398 cg26569800 6 29578313 p
GABBR1/MOG GABBR1 NA Body open sea Body - open sea
NA 0.910895729 0.908358741 -0.002536988
1.002792936
0.004857416 0.084334168 0.438614889 0.66391864
0.94333036 -7.675707167 cg05054006 17 28443555 q
SLC6A4 CCDC55 NA TSS1500 shore TSS1500 - shore
NA 0.082664432 0.087521848 0.004857416
0.944500532
0.001018439 0.029127991 0.438203987 0.664213304
0.94333036 -7.675891556 cg04207032 22 20004746 q
COMT ARVCF NA TSS1500 shore TSS1500 - shore
NA 0.028777903 0.029796342 0.001018439
0.965819999
-0.004386062 0.177718821 -0.436943358 0.665117657
0.94333036 -7.676456189 cg01089249 2 171676553 q
GAD1 GAD1 NA Body island Body - island
NA 0.179226529 0.174840468 -0.004386062
1.025086074
0.004122436 0.844659481 0.436610946 0.665356209
0.94333036 -7.676604807 cg17745803 6 29631321 p
GABBR1/MOG MOG NA Body open sea Body - open sea
NA 0.843242393 0.847364829 0.004122436
0.995134993
0.005819121 0.104046356 0.435615638 0.666070693
0.94333036 -7.677049133 cg14193550 6 29621467 p
GABBR1/MOG MOG -3291 IGR shelf IGR - shelf
NA 0.102046033 0.107865155 0.005819121
0.946051883
0.002947663 0.921790325 0.435195032 0.666372721
0.94333036 -7.677236599 cg00040027 2 172747482 q
SLC25A12 SLC25A12 NA Body shelf Body - shelf
NA 0.920777066 0.923724729 0.002947663
0.996808938
0.005518906 0.565672175 0.435154772 0.666401634
0.94333036 -7.677254534 cg11712482 22 19928667 q
COMT COMT NA TSS1500 shore TSS1500 - shore
NA 0.563775051 0.569293957 0.005518906
0.990305701
-0.006608925 0.61866891 -0.434238414 0.667059859
0.94333036 -7.677662301 cg04856117 22 19949901 q
COMT COMT NA 5'UTR open sea 5'UTR - open sea
NA 0.620940727 0.614331803 -0.006608925
1.010757906
-0.006723098 0.511463857 -0.433208954 0.667799647
0.94333036 -7.678119385 cg23732781 6 29595016 p
GABBR1/MOG GABBR1 NA Body shore Body - shore
NA 0.513774921 0.507051824 -0.006723098
1.013259191
-0.002962296 0.881809065 -0.431409885 0.669093302
0.94333036 -7.678915604 cg06979684 11 27677125 p

BDNF BDNF NA 3'UTR open sea 3'UTR - open sea
NA 0.882827354 0.879865057 -0.002962296
1.003366763
-0.001128437 0.031182483 -0.430352241 0.669854302
0.94333036 -7.67938216 cg19868007 6 29691890 p
GABBR1/MOG HLA-F NA Body island Body - island
NA 0.031570383 0.030441946 -0.001128437
1.037068491
-0.002238128 0.930091523 -0.429429009 0.670518879
0.94333036 -7.679788497 cg22659852 6 152464820 q
ESR1 SYNE1 NA Body open sea Body - open sea
NA 0.930860879 0.928622752 -0.002238128
1.002410157
0.002722671 0.928405747 0.428912335 0.670890919
0.94333036 -7.680015522 cg18860567 6 29578925 p
GABBR1/MOG GABBR1 NA Body open sea Body - open sea
NA 0.927469829 0.9301925 0.002722671
0.997073003
-0.000958768 0.043306012 -0.428400993 0.671259202
0.94333036 -7.680239938 cg13604811 6 29720802 p
MOG IFITM4P 2218 IGR island IGR - island
NA 0.043635589 0.04267682 -0.000958768
1.022465802
-0.003008091 0.209296201 -0.427872142 0.671640183
0.94333036 -7.68047176 cg19601636 22 20001066 q
COMT ARVCF NA 5'UTR shelf 5'UTR - shelf
NA 0.210330233 0.207322142 -0.003008091
1.014509261
-0.001018615 0.046434553 -0.426565814 0.672581633
0.943683769 -7.681043176 cg05128992 18 3450160
p DLGAP1 TGIF1 NA 5'UTR island 5'UTR -
island NA 0.046784702 0.045766086 -0.001018615
1.022257005
0.004064655 0.11375067 0.424007674 0.674426801
0.944243163 -7.682157158 cg25661973 21
34400733 q OLIG2 OLIG2 NA 3'UTR shore
3'UTR - shore NA 0.112353445 0.1164181
0.004064655 0.965085713
0.002777451 0.891197815 0.42392756 0.67448462 0.944243163
-7.682191938 cg18971999 6 29712014 p MOG
LOC285830 NA Body open sea Body - open sea NA
0.890243066 0.893020517 0.002777451
0.996889824
-0.001973689 0.018313998 -0.423142884 0.675051033
0.944243163 -7.682532246 cg03205258 22
19929274 q COMT TXNRD2 NA 1stExon island
1stExon - island V$ELK1_02 0.018992454 0.017018765
-0.001973689 1.115971341
-0.001733435 0.933588926 -0.419637344 0.677583836
0.945132935 -7.684044958 cg18679753 6
29443446 p GABBR1 MAS1L -11097 IGR open
sea IGR - open sea NA 0.934184794 0.932451359
-0.001733435 1.001859008
0.001043741 0.050685463 0.419440183 0.677726402
0.945132935 -7.684129668 cg20973396 6
29691899 p GABBR1/MOG HLA-F NA Body island

Body - island	NA	0.050326678	0.051370418
0.001043741	0.979682081
0.004424136	0.550604292	0.419393995	0.677759802
0.945132935	-7.684149507	cg12061917 6
29601556	p	GABBR1/MOG GABBR1	NA	TSS1500	shore
TSS1500 - shore	NA	0.549083495	0.553507631
0.004424136	0.992007091
0.002652414	0.090713973	0.41467937 0.681172568
0.947038495	-7.686163177	cg01225698 11
27742355	p	BDNF	BDNF	NA	Body	shore
Body - shore	NA	0.089802206	0.09245462
0.002652414	0.971311179
0.004156377	0.756021321	0.41460941 0.681223262
0.947038495	-7.686192888	cg00359010 6
29635692	p	GABBR1/MOG MOG	NA	3'UTR	open
sea	3'UTR - open sea	NA	0.754592567	0.758748944
0.004156377	0.994522066
0.00313555 0.279789041	0.412706998	0.682602342
0.947038495	-7.686998919	cg17053201 6
29593246	p	GABBR1/MOG GABBR1	NA	Body	shelf
Body - shelf	NA	0.278711195	0.281846745
0.00313555 0.988874982
0.00210255 0.094304127	0.412526728	0.68273308 0.947038495
-7.687075107	cg13555101 9	4490751	p	SLC1A1
SLC1A1	NA	1stExon	island	1stExon - island	NA
0.093581375	0.095683925	0.00210255 0.97802609
-0.001816537	0.091276214	-0.411833677	0.683235796
0.947038495	-7.687367707	cg13612847 2
171672205 q	GAD1	GAD1	NA	TSS1500	shore
TSS1500 - shore	NA	0.091900648	0.090084111
-0.001816537	1.020164899
0.000789495	0.039388498	0.411772791	0.683279967
0.947038495	-7.687393389	cg06025631 11
27722549	p	BDNF	BDNF	NA	Body	island
Body - island	NA	0.039117109	0.039906604
0.000789495	0.980216432
0.004374747	0.71373633 0.407718444	0.68622387 0.950156128
-7.689095095	cg19786983 15	88336651	q	NTRK3
NTRK3-AS1 -83337	IGR	open sea	IGR - open sea	NA
0.712232511	0.716607258	0.004374747
0.993895196
-0.000579903	0.013799139	-0.405988339	0.687481641
0.950404229	-7.68981619	cg18943949 22
19842501	q	COMT	GNB1L	NA	TSS200	island
TSS200 - island	NA	0.013998481	0.013418578
-0.000579903	1.043216427
0.002558748	0.828760214	0.403479383	0.68930724
0.950404229	-7.690856512	cg08829877 22
19960832	q	COMT	ARVCF	NA	Body	island
Body - island	NA	0.827880644	0.830439392
0.002558748	0.996918802
0.001166353	0.061558319	0.402361503	0.690121258
0.950404229	-7.691317977	cg05901579 15
88800567	q	NTRK3	NTRK3	NA	TSS1500	island
TSS1500 - island	NA	0.061157385	0.062323738
0.001166353	0.981285574

0.00580156 0.829358337 0.402001484 0.690383497
0.950404229 -7.691466325 cg24764793 6
152126745 q ESR1 ESR1 NA 5'UTR shelf
5'UTR - shelf NA 0.827364051 0.833165611
0.00580156 0.993036727
0.002096854 0.164988192 0.401211366 0.690959158
0.950404229 -7.691791437 cg07196069 9 4491175
p SLC1A1 SLC1A1 NA Body island Body -
island NA 0.164267398 0.166364252 0.002096854
0.987396006
-0.001447694 0.045967092 -0.399863488 0.69194162
0.950404229 -7.692344589 cg09793121 21
34398263 q OLIG2 OLIG2 NA 1stExon island
1stExon - island NA 0.046464737 0.045017043
-0.001447694 1.032158798
0.004117743 0.679764372 0.397530669 0.693643287
0.950404229 -7.693297595 cg14528040 6
29628293 p GABBR1/MOG MOG NA Body open
sea Body - open sea NA 0.678348897 0.68246664
0.004117743 0.993966382
-0.002781298 0.71112222 -0.397430547 0.693716357
0.950404229 -7.693338374 cg16733589 6
29628224 p GABBR1/MOG MOG NA Body open
sea Body - open sea NA 0.712078291 0.709296993
-0.002781298 1.003921204
0.001098583 0.02695173 0.395014364 0.695480613
0.950404229 -7.69431937 cg11576659 6
29691135 p GABBR1/MOG HLA-F NA 1stExon island
1stExon - island NA 0.026574092 0.027672675
0.001098583 0.960300802
-0.003302137 0.08856022 -0.394798664 0.695638197
0.950404229 -7.694406658 cg24217789 6
29717384 p MOG LOC285830 NA TSS1500 shore
TSS1500 - shore NA 0.08969533 0.086393192 -
0.003302137 1.038222202
-0.003741452 0.879401094 -0.394661706 0.695738262
0.950404229 -7.694462057 cg03898786 6
29586020 p GABBR1/MOG GABBR1 NA Body open
sea Body - open sea NA 0.880687218 0.876945767
-0.003741452 1.004266457
0.001534413 0.069658908 0.394468256 0.695879611
0.950404229 -7.694540275 cg06212631 2
171785547 q GAD1 GORASP2 NA TSS200 island
TSS200 - island NA 0.069131453 0.070665866
0.001534413 0.978286362
0.000594717 0.022602666 0.394383693 0.695941403
0.950404229 -7.694574454 cg07038887 17
28618554 q SLC6A4 BLMH NA Body island
Body - island NA 0.022398232 0.022992948
0.000594717 0.974134852
-0.00213614 0.13367479 -0.394034276 0.69619675 0.950404229
-7.694715607 cg18861140 18 3449789 p DLGAP1
TGIF1 NA 5'UTR island 5'UTR - island NA
0.134409088 0.132272948 -0.00213614
1.016149485
-0.001655187 0.100557962 -0.393175947 0.696824153
0.950404229 -7.695061818 cg05818894 11

27740078 p BDNF BDNF NA Body shore
Body - shore V$SP1_Q6 0.101126933 0.099471746
-0.001655187 1.01663977
-0.001586971 0.06416972 -0.391208941 0.698262771
0.9514178 -7.6958524 cg25669230 12 72332708 q TPH2
TPH2 NA 1stExon open sea 1stExon - open sea NA
0.064715241 0.06312827 -0.001586971 1.025138832
-0.007301884 0.565205624 -0.388217023 0.700453164
0.953452664 -7.697047386 cg05951817 17
28562142 q SLC6A4 SLC6A4 NA 5'UTR shore
5'UTR - shore NA 0.567715646 0.560413762
-0.007301884 1.013029452
-0.004188012 0.809320767 -0.38577589 0.702242261
0.954937787 -7.698015654 cg12904135 2
171669275 q GAD1 GAD1 -3925 IGR shore
IGR - shore NA 0.810760397 0.806572384
-0.004188012 1.005192359
-0.002570164 0.867958477 -0.384050035 0.703508179
0.955709224 -7.698696558 cg03906120 6
29583573 p GABBR1/MOG GABBR1 NA Body open
sea Body - open sea NA 0.868841971 0.866271806
-0.002570164 1.002966927
0.004746021 0.555990857 0.382734991 0.704473345
0.956070969 -7.699213354 cg11046380 2
172543743 q SLC25A12 DYNC1I2 NA TSS1500 shore
TSS1500 - shore NA 0.554359412 0.559105433
0.004746021 0.991511402
0.006646066 0.503268953 0.380775466 0.705912448
0.957074558 -7.699980165 cg13638427 6
29617320 p GABBR1/MOG MOG -7438 IGR shore
IGR - shore NA 0.500984368 0.507630434
0.006646066 0.986907668
-0.002963773 0.323059976 -0.379482506 0.706862621
0.957413926 -7.700483996 cg13666340 9 4666266
p SLC1A1 C9orf68 NA 5'UTR shelf 5'UTR -
shelf NA 0.324078773 0.321114999 -0.002963773
1.009229634
-0.001999219 0.884094794 -0.376741661 0.708878399
0.958314024 -7.701546413 cg25000210 6
29455302 p GABBR1 MAS1L NA 1stExon open
sea 1stExon - open sea NA 0.884782025 0.882782806
-0.001999219 1.002264678
-0.002154391 0.111515702 -0.376673969 0.708928211
0.958314024 -7.701572556 cg24567537 6
29520527 p GABBR1 UBD -2862 IGR shore
IGR - shore NA 0.112256274 0.110101883
-0.002154391 1.019567249
0.001601147 0.096164199 0.375436912 0.70983874
0.958597628 -7.702049484 cg18293833 6
29600462 p GABBR1/MOG GABBR1 NA 5'UTR island
5'UTR - island NA 0.095613804 0.097214951
0.001601147 0.983529828
0.002545493 0.850694929 0.374092721 0.710828618
0.958987721 -7.702565953 cg02403412 22
19894638 q COMT TXNRD2 NA Body island
Body - island NA 0.849819916 0.852365408
0.002545493 0.997013614

-0.002034856	0.879386623	-0.372130528	0.712274516
0.959991663	-7.703316576	cg12166917 6
29571429 p	GABBR1/MOG GABBR1	NA	Body	open
sea	Body - open sea	V$TAXCREB_01	0.880086104
0.878051249	-0.002034856	1.002317467
-0.006253416	0.800537921	-0.369572569	0.71416105
0.961082712	-7.70428923	cg03536022 6
29706879 p	MOG	LOC285830 NA	Body	open
sea	Body - open sea	NA	0.802687532	0.796434117
-0.006253416	1.007851767
-0.00298982	0.866629411	-0.367248688	0.715876537
0.961082712	-7.70516711	cg18356448 18	3881547
p	DLGAP1	DLGAP1	NA	TSS1500	shore	TSS1500
- shore	NA	0.867657162	0.864667342	-0.00298982
1.003457769
-0.001968815	0.104874492	-0.366856227	0.7161664
0.961082712	-7.705314826	cg21091227 18	4454304
p	DLGAP1	DLGAP1-AS5 189702	IGR	island	IGR -
island	NA	0.105551273	0.103582457	-0.001968815
1.019007234
-0.000639777	0.016973806	-0.366320757	0.716561955
0.961082712	-7.705516115	cg26332258 6
29720425 p	MOG	IFITM4P	1841	IGR	island
IGR - island	NA	0.017193729	0.016553952
-0.000639777	1.038647992
0.002830512	0.751493322	0.364470095	0.717929666
0.961082712	-7.706209557	cg17514431 6
29717656 p	MOG	LOC285830 NA	TSS1500	shore
TSS1500 - shore	NA	0.750520334	0.753350846
0.002830512	0.996242771
0.000990451	0.04663594 0.36429328 0.718060389	0.961082712
-7.706275627	cg06078334 6	29595653 p
GABBR1/MOG GABBR1	NA	Body	island	Body - island
NA	0.046295472	0.047285923	0.000990451
0.979053999
-0.002724784	0.676652075	-0.363830181	0.718402807
0.961082712	-7.706448521	cg15437231 12
72259306 q	TPH2	TBC1D15	NA	Body	open
sea	Body - open sea	NA	0.677588719	0.674863935
-0.002724784	1.004037531
0.0038855 0.846319397	0.363422369	0.718704397
0.961082712	-7.706600595	cg27368718 18	3883519
p	DLGAP1	DLGAP1-AS3 5339	IGR	shelf	IGR -
shelf	NA	0.844983756	0.848869256	0.0038855
0.995422734
-0.002150163	0.912478116	-0.359335588	0.721729216
0.963724013	-7.708115214	cg26744084 6
29581182 p	GABBR1/MOG GABBR1	NA	Body	open
sea	Body - open sea	NA	0.913217235	0.911067072
-0.002150163	1.002360049
0.003350132	0.224554707	0.358744339	0.722167206
0.963724013	-7.708332933	cg02965237 21
34393668 q	OLIG2	OLIG2	-4548	IGR	shore
IGR - shore	NA	0.223403099	0.226753232
0.003350132	0.985225644
0.000858788	0.037977927	0.357174973	0.723330237
0.963724013	-7.708909104	cg01763173 2

171674437 q GAD1 GAD1 NA 5'UTR island
5'UTR - island NA 0.037682718 0.038541506
0.000858788 0.97771784
0.004312666 0.7856323 0.356152543 0.724088303
0.963724013 -7.709283127 cg11361387 22
19949873 q COMT COMT NA 5'UTR open
sea 5'UTR - open sea NA 0.784149821 0.788462487
0.004312666 0.994530284
0.001768847 0.223523386 0.353259973 0.726234492
0.963724013 -7.710335519 cg13965062 9
87284706 q NTRK2 NTRK2 NA 5'UTR island
5'UTR - island NA 0.222915345 0.224684192
0.001768847 0.992127408
-0.001752971 0.904136513 -0.351901895 0.72724292
0.963724013 -7.710826685 cg06266853 6
29589707 p GABBR1/MOG GABBR1 NA Body open
sea Body - open sea NA 0.904739096 0.902986125
-0.001752971 1.001941304
0.004465124 0.733922346 0.351686404 0.727402975
0.963724013 -7.710904448 cg11234013 11
27646797 p BDNF BDNFOS NA Body open
sea Body - open sea NA 0.732387459 0.736852584
0.004465124 0.993940274
-0.001242365 0.053824729 -0.351484349 0.727553064
0.963724013 -7.710977319 cg27351358 11
27743258 p BDNF BDNF NA TSS1500 shore
TSS1500 - shore NA 0.054251792 0.053009427
-0.001242365 1.02343668
0.000710042 0.038941826 0.351407573 0.727610096
0.963724013 -7.711004997 cg06087185 6
29521499 p GABBR1 UBD -1890 IGR island
IGR - island NA 0.038697749 0.039407791
0.000710042 0.981982192
0.003412476 0.83464206 0.350477893 0.72830083 0.963724013
-7.711339678 cg13682912 6 29627142 p
GABBR1/MOG MOG NA Body open sea Body - open sea
NA 0.833469021 0.836881497 0.003412476
0.99592239
0.001117135 0.951292251 0.349469255 0.729050488
0.963724013 -7.711701789 cg15156844 18 3880020
p DLGAP1 DLGAP1 NA 1stExon island 1stExon
- island NA 0.950908236 0.952025371 0.001117135
0.99882657
0.001024074 0.045170335 0.349357859 0.729133299
0.963724013 -7.711741717 cg00476814 22
19974536 q COMT ARVCF NA Body island
Body - island NA 0.04481831 0.045842384
0.001024074 0.977660979
-0.002715138 0.86845739 -0.348353816 0.729879843
0.96377956 -7.712101036 cg16891968 6 29698284 p
MOG LOC285830 NA Body open sea Body - open sea
NA 0.869390718 0.866675581 -0.002715138
1.003132818
-0.000945682 0.030476739 -0.345982888 0.73164378
0.965177136 -7.71294545 cg11108676 15
88801004 q NTRK3 NTRK3 NA TSS1500 island

TSS1500 - island NA 0.030801817 0.029856135
-0.000945682 1.031674629
0.001428152 0.790069432 0.344229163 0.732949484
0.965968106 -7.713566363 cg12074883 6
29591155 p GABBR1/MOG GABBR1 NA Body open
sea Body - open sea NA 0.789578504 0.791006657
0.001428152 0.998194512
-0.005295995 0.34615909 -0.342448978 0.734275718
0.966784583 -7.714193441 cg14486905 2
171677602 q GAD1 GAD1 NA Body shore
Body - shore NA 0.347979588 0.342683593
-0.005295995 1.015454475
-0.001315376 0.074556922 -0.339392661 0.736554603
0.967424032 -7.715262516 cg17521020 6
29617613 p GABBR1/MOG MOG -7145 IGR shore
IGR - shore NA 0.075009083 0.073693707
-0.001315376 1.017849231
0.001914346 0.914320556 0.338547599 0.737185138
0.967424032 -7.715556432 cg02039276 6
29719081 p MOG IFITM4P NA TSS200 shore
TSS200 - shore NA 0.9136625 0.915576846
0.001914346 0.997909137
0.006885667 0.209213767 0.338387317 0.737304752
0.967424032 -7.715612097 cg14005211 2
171676925 q GAD1 GAD1 NA Body island
Body - island NA 0.206846819 0.213732486
0.006885667 0.967783714
-0.001018504 0.07898115 -0.336903937 0.73841207 0.967424032
-7.716126022 cg09124190 18 3448419 p DLGAP1
TGIF1 NA 5'UTR island 5'UTR - island
V$HEN1_02 0.079331261 0.078312757 -0.001018504
1.013005595
0.000884099 0.036626491 0.336152337 0.738973345
0.967424032 -7.716385563 cg08319422 6
29617817 p GABBR1/MOG MOG -6941 IGR island
IGR - island NA 0.036322582 0.03720668
0.000884099 0.976238192
0.000831969 0.043575633 0.336110664 0.739004469
0.967424032 -7.716399936 cg10901968 17
28563108 q SLC6A4 SLC6A4 NA TSS200 island
TSS200 - island NA 0.043289644 0.044121613
0.000831969 0.981143731
0.000550334 0.019994331 0.3325067 0.74169787 0.968684166
-7.717636297 cg14520770 22 19842507 q COMT
GNB1L NA TSS200 island TSS200 - island NA
0.019805154 0.020355488 0.000550334
0.972963851
0.000746942 0.043365388 0.331668383 0.742324858
0.968684166 -7.717921989 cg22841338 6
29720580 p MOG IFITM4P 1996 IGR island
IGR - island NA 0.043108627 0.043855569
0.000746942 0.982968138
-0.002418876 0.862006446 -0.331283449 0.742612815
0.968684166 -7.718052931 cg09603409 6
29587016 p GABBR1/MOG GABBR1 NA Body open
sea Body - open sea NA 0.862837934 0.860419058
-0.002418876 1.002811277

-0.003596013 0.72739946 -0.331033946 0.742799481
0.968684166 -7.718137724 cg18772882 15
88616079 q NTRK3 NTRK3 NA Body open
sea Body - open sea NA 0.728635589 0.725039577
-0.003596013 1.004959746
-0.005180567 0.747604972 -0.327537477 0.745417027
0.969764567 -7.71931931 cg08739755 2
172815385 q SLC25A12 HAT1 NA Body open
sea Body - open sea NA 0.749385792 0.744205225
-0.005180567 1.006961208
0.00130511 0.08273358 0.327419019 0.745505763 0.969764567
-7.719359123 cg23619332 11 27722060 p BDNF
BDNF NA Body island Body - island NA
0.082284949 0.083590059 0.00130511 0.98438678
-0.002093475 0.910335299 -0.325815774 0.746707073
0.969764567 -7.719896558 cg22166516 2
172641053 q SLC25A12 SLC25A12 NA 3'UTR open
sea 3'UTR - open sea NA 0.91105493 0.908961456 -
0.002093475 1.002303149
0.001064579 0.933129248 0.323150703 0.748705436
0.969764567 -7.720784137 cg08388004 11
27679632 p BDNF BDNF NA Body open
sea Body - open sea V$PAX5_02;V$P53_02 0.932763299
0.933827877 0.001064579 0.998859985
-0.00429594 0.342354178 -0.322485696 0.749204359
0.969764567 -7.721004483 cg06343355 6
29521023 p GABBR1 UBD -2366 IGR shore
IGR - shore NA 0.343830907 0.339534967
-0.00429594 1.012652423
0.00097508 0.928041473 0.321990121 0.749576236
0.969764567 -7.721168395 cg24312412 17
28512027 q SLC6A4 CCDC55 NA Body open
sea Body - open sea NA 0.92770629 0.928681369
0.00097508 0.998950039
0.00412498 0.691742457 0.321297936 0.75009575 0.969764567
-7.721396917 cg23325570 6 29711975 p MOG
LOC285830 NA Body open sea Body - open sea NA
0.690324495 0.694449476 0.00412498 0.99406007
0.004188979 0.382123067 0.319829332 0.751198393
0.969764567 -7.721880153 cg20322862 18 3412088
p DLGAP1 TGIF1 NA 5'UTR open sea 5'UTR -
open sea NA 0.380683105 0.384872084 0.004188979
0.989115919
-0.001336915 0.082052503 -0.319373936 0.751540418
0.969764567 -7.722029552 cg26069562 6
29691981 p GABBR1/MOG HLA-F NA Body island
Body - island NA 0.082512068 0.081175153
-0.001336915 1.01646951
-0.001880431 0.188044332 -0.319160634 0.751700635
0.969764567 -7.722099456 cg09990446 6
29716108 p MOG LOC285830 NA Body shore
Body - shore NA 0.18869073 0.1868103 -0.001880431
1.010065987
-0.002668475 0.125623568 -0.319004852 0.751817655
0.969764567 -7.72215048 cg26751972 6
29692000 p GABBR1/MOG HLA-F NA Body island

Body - island NA 0.126540857 0.123872381
-0.002668475 1.021542139
-0.000535265 0.016223992 -0.318129277 0.752475477
0.969764567 -7.7224368 cg09545452 2 172779111 q
SLC25A12 HAT1 NA Body island Body - island
NA 0.016407989 0.015872724 -0.000535265
1.033722315
0.005179907 0.728981019 0.31763948 0.752843545
0.969764567 -7.722596627 cg14713146 2
171782647 q GAD1 GORASP2 -2301 IGR shelf
IGR - shelf NA 0.727200426 0.732380334
0.005179907 0.992927298
-0.00176211 0.910385047 -0.314889897 0.754910868
0.971512763 -7.723489306 cg26089753 6
152127821 q ESR1 ESR1 NA TSS1500 shore
TSS1500 - shore NA 0.910990773 0.909228662
-0.00176211 1.001938028
0.004039421 0.811197291 0.312106444 0.757005532
0.972386719 -7.724385125 cg04507121 2
172778341 q SLC25A12 HAT1 NA TSS1500 shore
TSS1500 - shore NA 0.80980874 0.813848161
0.004039421 0.995036641
-0.000655906 0.029775573 -0.311172567 0.757708733
0.972386719 -7.724683911 cg19128271 22
19842843 q COMT GNB1L NA TSS1500 island
TSS1500 - island NA 0.03000104 0.029345134 -
0.000655906 1.02235144
-0.002186795 0.90189823 -0.309858435 0.75869862 0.972386719
-7.725102849 cg09414638 6 152239860 q ESR1
ESR1 NA Body open sea Body - open sea NA
0.902649941 0.900463145 -0.002186795
1.002428524
-0.007761759 0.64734852 -0.308974446 0.759364729
0.972386719 -7.725383668 cg20140394 18 4456154
p DLGAP1 DLGAP1-AS5 191552 IGR shore IGR -
shore NA 0.650016624 0.642254865 -0.007761759
1.01208517
-0.00214305 0.133969986 -0.308962248 0.759373921
0.972386719 -7.725387537 cg16257091 11
27743580 p BDNF BDNF NA TSS1500 island
TSS1500 - island NA 0.134706659 0.13256361 -
0.00214305 1.016166194
-0.000724224 0.075297901 -0.308324199 0.759854826
0.972386719 -7.725589725 cg19690984 6
29521631 p GABBR1 UBD -1758 IGR island
IGR - island NA 0.075546853 0.074822629
-0.000724224 1.009679211
-0.001599117 0.896793111 -0.304823519 0.762495056
0.972678014 -7.72669164 cg21167956 9 4603153
p SLC1A1 C9orf68 NA 3'UTR open sea 3'UTR -
open sea NA 0.897342808 0.895743691 -0.001599117
1.001785239
0.00108821 0.929983696 0.304577887 0.762680422
0.972678014 -7.726768488 cg12209876 6
152381560 q ESR1 ESR1 NA Body open
sea Body - open sea NA 0.929609624 0.930697834
0.00108821 0.998830759

-0.002391154 0.741464178 -0.30311819 0.763782275
0.972678014 -7.727223898 cg09265315 6
29572317 p GABBR1/MOG GABBR1 NA Body open
sea Body - open sea NA 0.742286137 0.739894983
-0.002391154 1.003231748
0.001996192 0.887596716 0.302474236 0.764268525
0.972678014 -7.727424113 cg10625096 6
29518072 p GABBR1 UBD -5317 IGR shelf
IGR - shelf NA 0.886910525 0.888906718
0.001996192 0.997754328
0.001710093 0.885442679 0.300618069 0.765670658
0.972678014 -7.727998856 cg23105820 6
29624956 p GABBR1/MOG MOG NA 1stExon open
sea 1stExon - open sea NA 0.884854834 0.886564927
0.001710093 0.998071102
0.001212344 0.895490083 0.299172261 0.766763368
0.972678014 -7.728444098 cg21864713 6
29555314 p GABBR1/MOG OR2H2 NA TSS1500 open
sea TSS1500 - open sea NA 0.89507334 0.896285684
0.001212344 0.998647369
-0.001234666 0.942221091 -0.297092731 0.768335883
0.972678014 -7.729080754 cg06872047 6
29585579 p GABBR1/MOG GABBR1 NA Body open
sea Body - open sea NA 0.942645508 0.941410841
-0.001234666 1.001311507
0.004164082 0.303813458 0.296807447 0.768551689
0.972678014 -7.72916775 cg20208009 22
19974048 q COMT ARVCF NA Body shore
Body - shore NA 0.302382055 0.306546136
0.004164082 0.986416136
-0.002933984 0.75227389 -0.2949587 0.76995065 0.972678014 -
7.729729504 cg00667298 6 29576329 p GABBR1/MOG GABBR1
NA Body open sea Body - open sea NA
0.753282447 0.750348463 -0.002933984
1.003910162
-0.000640248 0.053077487 -0.292156792 0.77207237
0.972678014 -7.730574228 cg17851017 6
29600994 p GABBR1/MOG GABBR1 NA TSS200 shore
TSS200 - shore NA 0.053297572 0.052657324
-0.000640248 1.012158764
-0.000540016 0.032594671 -0.291952842 0.772226879
0.972678014 -7.730635403 cg25638186 6
29720443 p MOG IFITM4P 1859 IGR island
IGR - island NA 0.032780302 0.032240285
-0.000540016 1.016749759
-0.001633323 0.803256033 -0.290187093 0.773564978
0.972678014 -7.731163257 cg17546147 6
29710109 p MOG LOC285830 NA Body open
sea Body - open sea NA 0.803817488 0.802184165
-0.001633323 1.002036095
0.001903227 0.913569035 0.28998985 0.773714494
0.972678014 -7.731222023 cg05115679 6
29593490 p GABBR1/MOG GABBR1 NA Body shore
Body - shore NA 0.912914801 0.914818028
0.001903227 0.997919557
0.002125445 0.844701319 0.289195959 0.774316377
0.972678014 -7.731458151 cg13873869 6

29638112 p GABBR1/MOG MOG NA 3'UTR open
sea 3'UTR - open sea NA 0.843970697 0.846096142
0.002125445 0.997487939
0.001614737 0.904422235 0.288601457 0.774767187
0.972678014 -7.731634553 cg03366312 21
34388564 q OLIG2 OLIG2 -9652 IGR shelf
IGR - shelf NA 0.903867169 0.905481906
0.001614737 0.99821671
-0.000948117 0.036062215 -0.28472701 0.77770712
0.972678014 -7.732775343 cg05238769 21
34399260 q OLIG2 OLIG2 NA Body island
Body - island NA 0.03638813 0.035440013 -
0.000948117 1.026752727
-0.002324627 0.815498577 -0.284095365 0.77818673
0.972678014 -7.73295987 cg03564415 6
29638575 p GABBR1/MOG MOG NA 3'UTR open
sea 3'UTR - open sea NA 0.816297668 0.813973041
-0.002324627 1.002855902
-0.001910597 0.172110731 -0.282664218 0.779273733
0.972678014 -7.733376452 cg04188862 15
88801474 q NTRK3 NTRK3-AS1 5513 IGR shore
IGR - shore NA 0.172767499 0.170856902
-0.001910597 1.01118244
-0.001999883 0.424523769 -0.281247417 0.780350284
0.972678014 -7.733786797 cg03316098 6
29601398 p GABBR1/MOG GABBR1 NA TSS1500 shore
TSS1500 - shore NA 0.425211229 0.423211346
-0.001999883 1.004725495
0.000516901 0.023242514 0.280611 0.780834007
0.972678014 -7.733970453 cg14480858 9 4666499
p SLC1A1 C9orf68 NA 1stExon shelf 1stExon
- shelf NA 0.02306483 0.02358173 0.000516901 0.978080489
0.002935136 0.825080364 0.278949691 0.782097142
0.972678014 -7.73444792 cg08027810 6
29593479 p GABBR1/MOG GABBR1 NA Body shore
Body - shore NA 0.824071411 0.827006547
0.002935136 0.996450891
-0.00044957 0.021369257 -0.278123476 0.782725558
0.972678014 -7.734684327 cg16723445 18 3451456
p DLGAP1 TGIF1 NA 5'UTR island 5'UTR -
island NA 0.021523797 0.021074227 -0.00044957
1.021332692
-0.00145461 0.901868687 -0.278105915 0.782738917
0.972678014 -7.734689345 cg18126978 6
29588988 p GABBR1/MOG GABBR1 NA Body open
sea Body - open sea NA 0.902368709 0.900914099
-0.00145461 1.001614593
-0.001277966 0.163463588 -0.277432266 0.783251405
0.972678014 -7.734881569 cg21836827 6
29720549 p MOG IFITM4P 1965 IGR island
IGR - island NA 0.163902888 0.162624922
-0.001277966 1.007858365
0.000814286 0.041636087 0.276782563 0.783745768
0.972678014 -7.735066521 cg16203801 6
29716637 p MOG LOC285830 NA Body island
Body - island NA 0.041356176 0.042170462
0.000814286 0.980690608

0.001173697 0.928685606 0.276160905 0.784218878
0.972678014 -7.735243086 cg05421799 2
172778343 q SLC25A12 HAT1 NA TSS1500 shore
TSS1500 - shore NA 0.928282147 0.929455844
0.001173697 0.998737221
0.004271428 0.327533829 0.275805901 0.78448909
0.972678014 -7.735343737 cg01138652 22
19973978 q COMT ARVCF NA Body shore
Body - shore NA 0.326065526 0.330336954
0.004271428 0.987069482
0.0034141 0.579991847 0.275458847 0.784753276
0.972678014 -7.73544201 cg07746998 6
152126785 q ESR1 ESR1 NA 5'UTR shelf
5'UTR - shelf NA 0.57881825 0.58223235 0.0034141
0.99413619
0.001270234 0.934393357 0.274686171 0.785341551
0.972678014 -7.735660362 cg05844420 2
171621820 q GAD1 SP5 49963 IGR open
sea IGR - open sea NA 0.933956714 0.935226948
0.001270234 0.998641791
-0.002205216 0.130164449 -0.274510132 0.785475596
0.972678014 -7.735710024 cg16850687 18 3594398
p DLGAP1 DLGAP1 NA Body open sea Body -
open sea NA 0.130922492 0.128717277 -0.002205216
1.017132238
-0.001109047 0.914612948 -0.2742488 0.785674599
0.972678014 -7.735783689 cg24936467 6
29598417 p GABBR1/MOG GABBR1 NA Body shore
Body - shore NA 0.914994183 0.913885136
-0.001109047 1.001213552
0.000563226 0.037284633 0.273938018 0.785911278
0.972678014 -7.735871202 cg18117895 11
27722066 p BDNF BDNF NA Body island
Body - island NA 0.037091024 0.03765425
0.000563226 0.985042167
-0.003063128 0.744324293 -0.273289717 0.786405064
0.972678014 -7.736053439 cg04351905 21
34441330 q OLIG2 OLIG1 NA TSS1500 shore
TSS1500 - shore NA 0.745377243 0.742314115
-0.003063128 1.004126458
-0.000636615 0.051771321 -0.273091433 0.786556107
0.972678014 -7.736109091 cg00267325 6
29691936 p GABBR1/MOG HLA-F NA Body island
Body - island NA 0.051990157 0.051353542
-0.000636615 1.012396711
0.001349331 0.887684402 0.272533895 0.786980858
0.972678014 -7.736265358 cg20795635 6
29578496 p GABBR1/MOG GABBR1 NA Body open
sea Body - open sea NA 0.88722057 0.8885699 0.001349331
0.998481459
-0.000648816 0.037014578 -0.271460461 0.787798822
0.972678014 -7.736565326 cg08870743 21
34398199 q OLIG2 OLIG2 NA TSS200 island
TSS200 - island NA 0.037237608 0.036588793
-0.000648816 1.017732616
0.000530853 0.021882871 0.271002501 0.788147865
0.972678014 -7.736692943 cg15980539 6

152128865 q ESR1 ESR1 NA 5'UTR island
5'UTR - island NA 0.02170039 0.022231243
0.000530853 0.976121308
0.001154783 0.067779823 0.270124709 0.788817016
0.972678014 -7.736936952 cg24900983 6
152128528 q ESR1 ESR1 NA TSS1500 shore
TSS1500 - shore NA 0.067382866 0.06853765
0.001154783 0.9831511
-0.004003823 0.826294654 -0.270073457 0.788856091
0.972678014 -7.736951174 cg02155405 2
172776401 q SLC25A12 HAT1 -2534 IGR shelf
IGR - shelf NA 0.827670968 0.823667145
-0.004003823 1.004860972
-0.000924194 0.068035661 -0.269578013 0.789233852
0.972678014 -7.737088525 cg20954537 11
27721668 p BDNF BDNF NA Body island
Body - island NA 0.068353352 0.067429158
-0.000924194 1.013706148
-0.002195792 0.109110085 -0.26921273 0.789512402
0.972678014 -7.73718963 cg17413943 11
27739827 p BDNF BDNF NA Body shore
Body - shore NA 0.109864888 0.107669096
-0.002195792 1.020393893
-0.000477774 0.030147432 -0.267882181 0.790527263
0.972678014 -7.737556753 cg26491697 18 3451447
p DLGAP1 TGIF1 NA 5'UTR island 5'UTR -
island NA 0.030311667 0.029833893 -0.000477774
1.016014471
0.001111746 0.909347885 0.267009794 0.791192867
0.972678014 -7.737796478 cg07412232 6
29495773 p GABBR1 LINC01015 -1410 IGR open
sea IGR - open sea NA 0.908965723 0.910077469
0.001111746 0.998778405
-0.002923164 0.453638348 -0.266781049 0.79136742
0.972678014 -7.737859207 cg11450715 2
171627404 q GAD1 GAD1 -45796 IGR island
IGR - island NA 0.454643186 0.451720022
-0.002923164 1.006471185
-0.002449721 0.756262496 -0.264748537 0.792918881
0.973395462 -7.73841423 cg26646411 22
19841374 q COMT GNB1L NA 5'UTR shore
5'UTR - shore NA 0.757104588 0.754654866
-0.002449721 1.003246149
-0.002631365 0.861526621 -0.264072844 0.793434844
0.973395462 -7.738597807 cg26987604 6
29696647 p MOG LOC285830 NA Body open
sea Body - open sea NA 0.862431153 0.859799788
-0.002631365 1.003060439
-0.00061291 0.041663173 -0.262722328 0.794466387
0.973395462 -7.738963326 cg15202447 6
29720635 p MOG IFITM4P 2051 IGR island
IGR - island NA 0.041873861 0.041260951
-0.00061291 1.014854481
-0.001649966 0.839306956 -0.262289171 0.794797318
0.973395462 -7.739080165 cg00162046 22
20006956 q COMT TANGO2 -1675 IGR shore

IGR - shore NA 0.839874132 0.838224166
-0.001649966 1.001968407
0.002891365 0.619688335 0.260462359 0.796193426
0.973755139 -7.739570814 cg15922678 6
29694484 p GABBR1/MOG LOC285830 NA Body shelf
Body - shelf NA 0.618694429 0.621585794
0.002891365 0.995348406
0.000442674 0.02724983 0.259859769 0.796654095
0.973755139 -7.739731911 cg21614759 6
152128426 q ESR1 ESR1 NA TSS1500 shore
TSS1500 - shore NA 0.02709766 0.027540335
0.000442674 0.983926303
-0.002549606 0.833393203 -0.257076618 0.798782722
0.973755139 -7.740471136 cg14140375 6
29692582 p GABBR1/MOG HLA-F NA Body shore
Body - shore NA 0.83426963 0.831720024 -
0.002549606 1.003065462
-0.00109254 0.930631253 -0.256385172 0.799311801
0.973755139 -7.74065356 cg15370577 6
29580340 p GABBR1/MOG GABBR1 NA Body open
sea Body - open sea NA 0.931006813 0.929914273
-0.00109254 1.001174882
0.000779976 0.062852806 0.256152045 0.799490207
0.973755139 -7.740714956 cg08856772 2
172751016 q SLC25A12 SLC25A12 NA TSS1500 island
TSS1500 - island NA 0.062584689 0.063364665
0.000779976 0.987690679
-0.001192106 0.116004713 -0.255567386 0.799937678
0.973755139 -7.740868684 cg01583131 11
27744675 p BDNF BDNF NA TSS1500 shore
TSS1500 - shore NA 0.1164145 0.115222394 -
0.001192106 1.010346131
0.000766005 0.062208182 0.253514629 0.80150931
0.973755139 -7.741405662 cg05634149 21
34395317 q OLIG2 OLIG2 -2899 IGR island
IGR - island V$ARP1_01 0.061944868 0.062710873
0.000766005 0.987785133
0.001915947 0.882193627 0.25281621 0.802044226
0.973755139 -7.741587377 cg21288364 6
29524117 p GABBR1 UBD NA Body shelf
Body - shelf NA 0.88153502 0.883450967
0.001915947 0.997831292
0.001748484 0.931283517 0.252179955 0.802531615
0.973755139 -7.741752483 cg06643882 18 3773774
p DLGAP1 DLGAP1 NA Body shelf Body -
shelf NA 0.930682475 0.93243096 0.001748484
0.99812481
0.001100495 0.059921738 0.251454672 0.803087301
0.973755139 -7.741940187 cg11286436 15
88798582 q NTRK3 NTRK3 NA Body shore
Body - shore NA 0.059543443 0.060643938
0.001100495 0.981853174
-0.000681502 0.051455764 -0.251216374 0.803269899
0.973755139 -7.742001741 cg19538089 2
171673547 q GAD1 GAD1 NA 5'UTR island
5'UTR - island NA 0.05169003 0.051008529 -
0.000681502 1.01336053

0.000744402 0.059276235 0.25074293 0.803632714
0.973755139 -7.742123863 cg02527472 11
27743348 p BDNF BDNF NA TSS1500 shore
TSS1500 - shore NA 0.059020347 0.059764749
0.000744402 0.987544464
0.000468914 0.022650753 0.24856874 0.805299428
0.974911166 -7.742681733 cg19760323 6
29716554 p MOG LOC285830 NA Body island
Body - island NA 0.022489563 0.022958478
0.000468914 0.979575519
0.001888963 0.880795598 0.245255203 0.807841338
0.975260079 -7.743522639 cg00539542 6
29644544 p GABBR1/MOG ZFP57 NA Body open
sea Body - open sea NA 0.880146267 0.88203523
0.001888963 0.997858404
-0.001637753 0.883314075 -0.243899138 0.80888223
0.975260079 -7.743863537 cg24782003 18 4459167
p DLGAP1 DLGAP1-AS5 194565 IGR shelf IGR -
shelf NA 0.883877052 0.882239299 -0.001637753
1.001856359
-0.001465557 0.896995152 -0.243791695 0.808964717
0.975260079 -7.743890467 cg05863862 6
29649807 p GABBR1/MOG ZFP57 9638 IGR open
sea IGR - open sea NA 0.897498938 0.89603338 -
0.001465557 1.001635606
-0.005196785 0.781103614 -0.242691737 0.809809308
0.975260079 -7.744165479 cg10433043 6
152432725 q ESR1 SYNE1 -10094 IGR open
sea IGR - open sea NA 0.782890009 0.777693224
-0.005196785 1.006682307
0.00065183 0.024896297 0.242319107 0.810095481
0.975260079 -7.744258363 cg01543173 21
34442534 q OLIG2 OLIG1 NA 1stExon island
1stExon - island NA 0.024672231 0.025324061
0.00065183 0.974260447
0.00184252 0.832963529 0.24157357 0.81066812 0.975260079 -
7.744443774 cg06308109 6 29548691 p GABBR1/MOG
SNORD32B NA TSS1500 open sea TSS1500 - open sea NA
0.832330163 0.834172682 0.00184252 0.997791202
-0.000608375 0.040495151 -0.235383044 0.815427065
0.975260079 -7.745961333 cg17869426 21
34442160 q OLIG2 OLIG1 NA TSS1500 shore
TSS1500 - shore NA 0.04070428 0.040095905 -
0.000608375 1.015172996
0.001345236 0.921886555 0.23368625 0.816732725
0.975260079 -7.746370434 cg03732055 6
152201038 q ESR1 ESR1 NA Body open
sea Body - open sea NA 0.92142413 0.922769366
0.001345236 0.998542175
0.00042446 0.018071505 0.232962511 0.817289795
0.975260079 -7.746544032 cg08729407 17
28431896 q SLC6A4 EFCAB5 NA Body open
sea Body - open sea NA 0.017925597 0.018350057
0.00042446 0.976868737
-0.000351364 0.013562227 -0.232770338 0.817437729
0.975260079 -7.746590037 cg10687604 6
29720687 p MOG IFITM4P 2103 IGR island

IGR - island NA 0.013683008 0.013331644
-0.000351364 1.026355639
-0.001218711 0.074769035 -0.232430349 0.817699467
0.975260079 -7.746671335 cg04049102 6
29716651 p MOG LOC285830 NA Body island
Body - island NA 0.075187967 0.073969256
-0.001218711 1.016475913
0.000506872 0.049836223 0.231369723 0.818516121
0.975260079 -7.746924192 cg19622911 18 3771570
p DLGAP1 DLGAP1 NA Body island Body -
island NA 0.049661986 0.050168858 0.000506872
0.989896681
-0.001301621 0.89749484 -0.230350592 0.819301019
0.975260079 -7.747166071 cg26219152 6
152443475 q ESR1 SYNE1 NA 3'UTR open
sea 3'UTR - open sea NA 0.897942273 0.896640651
-0.001301621 1.001451665
0.005223457 0.532485629 0.229605682 0.819874842
0.975260079 -7.747342193 cg20709110 22
19946873 q COMT COMT NA 5'UTR open
sea 5'UTR - open sea NA 0.530690065 0.535913522
0.005223457 0.990253172
0.000690066 0.030588309 0.227368795 0.821598579
0.975260079 -7.747867652 cg18388802 22
20004382 q COMT ARVCF NA TSS200 island
TSS200 - island V$SP1_01 0.030351099 0.031041165
0.000690066 0.977769327
-0.001100804 0.922046905 -0.22690714 0.821954442
0.975260079 -7.74797546 cg03810301 6
29590017 p GABBR1/MOG GABBR1 NA Body open
sea Body - open sea NA 0.922425307 0.921324503
-0.001100804 1.001194806
0.001607371 0.086390472 0.2242045 0.824038515
0.975260079 -7.748602209 cg21153898 6
29521788 p GABBR1 UBD -1601 IGR island
IGR - island NA 0.085837938 0.087445309
0.001607371 0.981618557
0.000822538 0.059578452 0.224132168 0.82409431
0.975260079 -7.74861888 cg24671939 18 3593798
p DLGAP1 FLJ35776 NA TSS1500 open sea TSS1500
- open sea NA 0.059295704 0.060118242 0.000822538
0.986317996
0.001076441 0.924167002 0.223781964 0.824364461
0.975260079 -7.74869952 cg13811469 22
20020657 q COMT C22orf25 NA 5'UTR open
sea 5'UTR - open sea NA 0.923796976 0.924873416
0.001076441 0.998836122
-0.001944851 0.887899361 -0.223440721 0.82462772
0.975260079 -7.748777975 cg21147063 21
34354927 q OLIG2 OLIG2 -43289 IGR shelf
IGR - shelf NA 0.888567903 0.886623053
-0.001944851 1.002193548
0.000894315 0.080135411 0.222093465 0.825667292
0.975260079 -7.749086556 cg27546977 6
29521152 p GABBR1 UBD -2237 IGR island
IGR - island NA 0.07982799 0.080722306
0.000894315 0.98892108

0.000507704	0.051458503	0.221982509	0.825752922
0.975260079	-7.749111887	cg09697651 6
29716568 p	MOG	LOC285830 NA	Body	island
Body - island	NA	0.051283979	0.051791684
0.000507704	0.990197171
0.002261417	0.557138399	0.221593771	0.826052948
0.975260079	-7.749200535	cg12315353 6
29573089 p	GABBR1/MOG GABBR1	NA	Body	open
sea	Body - open sea	NA	0.556361037	0.558622454
0.002261417	0.995951797
0.000880202	0.914061409	0.221468464	0.826149665
0.975260079	-7.749229077	cg14111380 6
29576818 p	GABBR1/MOG GABBR1	NA	Body	open
sea	Body - open sea	NA	0.913758839	0.914639041
0.000880202	0.999037651
-0.001270778	0.137339598	-0.220833735	0.826639617
0.975260079	-7.749373407	cg03628748 9
87285133 q	NTRK2	NTRK2	NA	5'UTR	island
5'UTR - island	NA	0.137776428	0.13650565 -
0.001270778	1.009309344
-0.00218469	0.788843628	-0.220602556	0.826818083
0.975260079	-7.749425872	cg01335087 22
19950166 q	COMT	COMT	NA	Body	open
sea	Body - open sea	NA	0.789594615	0.787409925
-0.00218469	1.002774527
-0.00389658	0.588940875	-0.220221804	0.827112037
0.975260079	-7.749512162	cg23892836 6
29692085 p	GABBR1/MOG HLA-F	NA	Body	island
Body - island	NA	0.590280325	0.586383744
-0.00389658	1.006645104
0.000470045	0.025155343	0.219825497	0.827418027
0.975260079	-7.749601819	cg20340508 21
34442377 q	OLIG2	OLIG1	NA	TSS200	island
TSS200 - island	NA	0.024993765	0.02546381
0.000470045	0.981540665
-0.000575896	0.028661137	-0.219134947	0.82795127
0.975260079	-7.749757659	cg14514600 9	4666477
p	SLC1A1	C9orf68	NA	1stExon	shelf	1stExon
- shelf	V$RFX1_02 0.028859102	0.028283206	-0.000575896
1.020361765
0.001428333	0.921421883	0.218756357	0.828243652
0.975260079	-7.74984289	cg17036562 12
72335305 q	TPH2	TPH2	NA	Body	open
sea	Body - open sea	NA	0.920930894	0.922359226
0.001428333	0.998451436
0.000958272	0.935710477	0.217602019	0.829135293
0.975260079	-7.750101856	cg16527407 6
29582672 p	GABBR1/MOG GABBR1	NA	Body	open
sea	Body - open sea	NA	0.935381071	0.936339343
0.000958272	0.998976576
0.00121435 0.901573949	0.217592484	0.829142659
0.975260079	-7.75010399	cg23991188 6
29607758 p	GABBR1/MOG MOG	-17000	IGR	open
sea	IGR - open sea	NA	0.901156516	0.902370867
0.00121435 0.998654266
0.001122315	0.913605872	0.217425585	0.829271596
0.975260079	-7.750141317	cg15242223 6

29573377 p GABBR1/MOG GABBR1 NA Body open
sea Body - open sea NA 0.913220076 0.914342391
0.001122315 0.998772544
-0.001307783 0.915546015 -0.216707297 0.829826558
0.975260079 -7.75030164 cg20838323 6
29549759 p GABBR1/MOG SNORD32B NA TSS1500 open
sea TSS1500 - open sea NA 0.915995566 0.914687782
-0.001307783 1.00142976
-0.004162233 0.417063421 -0.21489064 0.831230538
0.975269177 -7.750704758 cg12588917 6
29692082 p GABBR1/MOG HLA-F NA Body island
Body - island NA 0.418494188 0.414331955
-0.004162233 1.010045648
0.001046174 0.772483545 0.213608497 0.832221766
0.975269177 -7.750987231 cg02408532 6
29556369 p GABBR1/MOG OR2H2 NA 1stExon open
sea 1stExon - open sea NA 0.772123923 0.773170097
0.001046174 0.998646903
-0.001390969 0.062045516 -0.213298298 0.832461623
0.975269177 -7.751055318 cg20830965 17
28619340 q SLC6A4 BLMH NA TSS1500 shore
TSS1500 - shore NA 0.062523661 0.061132692
-0.001390969 1.022753276
-0.000689442 0.037550309 -0.213008185 0.832685964
0.975269177 -7.751118908 cg14128584 6
29600583 p GABBR1/MOG GABBR1 NA 5'UTR island
5'UTR - island NA 0.037787304 0.037097862
-0.000689442 1.018584413
0.001409739 0.422668057 0.2107418 0.834439025
0.976466654 -7.751612703 cg11742207 15
88784902 q NTRK3 NTRK3 NA Body open
sea Body - open sea NA 0.422183459 0.423593198
0.001409739 0.996671951
0.000836022 0.091977412 0.209841109 0.835135954
0.976466654 -7.751807481 cg00528793 22
19842837 q COMT GNB1L NA TSS1500 island
TSS1500 - island NA 0.091690029 0.092526051
0.000836022 0.990964469
-0.00115297 0.11172876 -0.207002039 0.837333631
0.978171013 -7.752415998 cg00107488 22
19930437 q COMT COMT NA 5'UTR shore
5'UTR - shore NA 0.112125093 0.110972123
-0.00115297 1.010389726
0.000305606 0.015900488 0.206110923 0.838023704
0.978171013 -7.752605292 cg13433942 9 4679784
p SLC1A1 CDC37L1 NA 1stExon island 1stExon
- island V$PAX5_01 0.015795436 0.016101042 0.000305606
0.981019489
-0.000906237 0.892982871 -0.204282356 0.839440141
0.97879015 -7.752991172 cg06132455 22 19839565 q
COMT C22orf29 NA Body shelf Body - shelf
NA 0.893294389 0.892388153 -0.000906237
1.001015518
0.000414049 0.030660187 0.203579005 0.839985114
0.97879015 -7.753138686 cg13924755 6 29691755 p
GABBR1/MOG HLA-F NA Body island Body - island

NA 0.030517858 0.030931907 0.000414049
0.986614178
-0.002129959 0.875769789 -0.201540771 0.841564836
0.979087999 -7.7535633 cg16438069 9 4488463 p
SLC1A1 SLC1A1 -1964 IGR shore IGR - shore
NA 0.876501963 0.874372004 -0.002129959
1.002435987
0.001387886 0.153036189 0.201402354 0.84167214
0.979087999 -7.753591981 cg06874218 18 3447454
p DLGAP1 TGIF1 NA 5'UTR shore 5'UTR -
shore NA 0.152559104 0.15394699 0.001387886
0.99098465
0.00057119 0.034576935 0.200258745 0.842558807
0.979286701 -7.753828194 cg27306787 22
19879176 q COMT TXNRD2 NA Body open
sea Body - open sea NA 0.034380589 0.034951778
0.00057119 0.983657798
0.000785613 0.043370712 0.196749694 0.845280758
0.980687216 -7.754544615 cg14278853 6
29521756 p GABBR1 UBD -1633 IGR island
IGR - island NA 0.043100658 0.04388627
0.000785613 0.982098912
-0.001360369 0.911149511 -0.1956813 0.846109891
0.980687216 -7.754760233 cg00376979 21
34350727 q OLIG2 OLIG2 -47489 IGR island
IGR - island NA 0.911617138 0.910256769
-0.001360369 1.001494489
-0.001564724 0.78907532 -0.195186904 0.84649363 0.980687216
-7.754859614 cg20757073 6 29598333 p
GABBR1/MOG GABBR1 NA Body shore Body - shore
NA 0.789613194 0.788048469 -0.001564724
1.001985569
0.000776435 0.058578292 0.195009563 0.846631288
0.980687216 -7.754895201 cg18183163 2
171574141 q GAD1 SP5 NA 3'UTR shore
3'UTR - shore NA 0.058311393 0.059087827
0.000776435 0.986859662
0.000540689 0.036417226 0.193403037 0.847878545
0.981301057 -7.75521611 cg09575114 9 4666694
p SLC1A1 C9orf68 NA TSS200 shelf TSS200
- shelf NA 0.036231364 0.036772053 0.000540689
0.985296198
0.000424907 0.05435747 0.191494636 0.849360688
0.981313285 -7.75559388 cg15914769 11
27722774 p BDNF BDNF NA TSS200 shore
TSS200 - shore NA 0.054211408 0.054636315
0.000424907 0.992222993
-0.000897974 0.923328867 -0.191461432 0.84938648
0.981313285 -7.755600419 cg02342892 6
29431208 p GABBR1 OR2H1 NA 3'UTR open
sea 3'UTR - open sea NA 0.923637545 0.922739571
-0.000897974 1.000973161
-0.000488452 0.056402294 -0.190618764 0.850041113
0.981313285 -7.755766007 cg04627496 15
88799973 q NTRK3 NTRK3 NA TSS1500 island
TSS1500 - island NA 0.0565702 0.056081747 -
0.000488452 1.008709661

0.001702142 0.885989473 0.188695575 0.851535562
0.982209653 -7.756141194 cg11806762 11
27732958 p BDNF BDNF NA Body open
sea Body - open sea NA 0.885404362 0.887106504
0.001702142 0.998081243
0.000543266 0.045597364 0.187079243 0.852791994
0.982830201 -7.756453581 cg27569822 17
28563119 q SLC6A4 SLC6A4 NA TSS200 island
TSS200 - island NA 0.045410617 0.045953882
0.000543266 0.988178039
-0.000736931 0.921042235 -0.182213226 0.856576874
0.986361248 -7.757377845 cg02887726 6
29641082 p GABBR1/MOG ZFP57 NA Body open
sea Body - open sea NA 0.921295555 0.920558624
-0.000736931 1.000800526
0.000652917 0.051070076 0.180842109 0.857643983
0.986759435 -7.757633889 cg06380702 6
29720825 p MOG IFITM4P 2241 IGR island
IGR - island NA 0.050845636 0.051498552
0.000652917 0.987321663
0.000253639 0.010733923 0.179717136 0.858519727
0.986936964 -7.757842526 cg00716604 12
72233552 q TPH2 TBC1D15 NA 1stExon island
1stExon - island NA 0.010646735 0.010900373
0.000253639 0.976731255
-0.001328618 0.118700651 -0.177668859 0.860114693
0.98757632 -7.758219063 cg14026788 12 72233266 q
TPH2 TBC1D15 NA TSS1500 shore TSS1500 - shore
NA 0.119157363 0.117828745 -0.001328618
1.011275839
-0.000920178 0.870797561 -0.176501536 0.86102394
0.98757632 -7.758431726 cg21491555 22 19967786 q
COMT ARVCF NA Body island Body - island
NA 0.871113872 0.870193694 -0.000920178
1.00105744
-0.000867857 0.076043504 -0.176063966 0.86136482
0.98757632 -7.758511083 cg23497217 11 27723214 p
BDNF BDNF NA TSS1500 shore TSS1500 - shore
NA 0.07634183 0.075473974 -0.000867857
1.011498745
0.001429981 0.854583272 0.171869933 0.864633463
0.98757632 -7.759261725 cg09902251 15 88590236 q
NTRK3 NTRK3 NA Body open sea Body - open sea
NA 0.854091716 0.855521697 0.001429981
0.998328527
-0.000437949 0.047538097 -0.170952617 0.865348703
0.98757632 -7.759423497 cg23727007 6 29716796 p
MOG LOC285830 NA Body island Body - island
NA 0.047688642 0.047250693 -0.000437949
1.009268626
-0.001215191 0.887487744 -0.170106683 0.866008389
0.98757632 -7.759571915 cg25798600 6 29454954 p
GABBR1 MAS1L NA 1stExon open sea 1stExon - open
sea NA 0.887905466 0.886690275 -0.001215191
1.00137048
0.000331586 0.02249499 0.169570579 0.866426511
0.98757632 -7.759665594 cg05141870 17 28444127 q

SLC6A4 MIR423 NA Body shore Body - shore
NA 0.022381007 0.022712593 0.000331586
0.985400786
0.001858719 0.227523604 0.168666232 0.867131924
0.98757632 -7.759822949 cg22402007 9 87282823 q
NTRK2 NTRK2 NA TSS1500 shore TSS1500 - shore
NA 0.22688467 0.228743388 0.001858719
0.991874222
0.001476495 0.667699935 0.166787536 0.868597706
0.98757632 -7.760147157 cg09781307 11 27648324 p
BDNF BDNFOS NA Body open sea Body - open sea
V$GRE_C 0.66719239 0.668668885 0.001476495
0.997791889
-0.000490003 0.042844873 -0.165972844 0.869233486
0.98757632 -7.760286622 cg20664238 15 88798877 q
NTRK3 NTRK3 NA Body shore Body - shore
NA 0.043013312 0.042523309 -0.000490003
1.011523163
-0.000375585 0.0411501 -0.165394591 0.869684805
0.98757632 -7.760385197 cg01009697 9 87283470 q
NTRK2 NTRK2 NA TSS1500 island TSS1500 - island
NA 0.041279208 0.040903623 -0.000375585
1.009182194
-0.000387203 0.095861815 -0.165261076 0.869789018
0.98757632 -7.760407909 cg07306299 6 29617902 p
GABBR1/MOG MOG -6856 IGR island IGR - island
NA 0.095994916 0.095607712 -0.000387203
1.004049924
-0.000956895 0.901636625 -0.164738719 0.870196758
0.98757632 -7.760496589 cg19535685 22 19845442 q
COMT GNB1L 11781 IGR shelf IGR - shelf
NA 0.901965557 0.901008662 -0.000956895
1.001062026
-0.000702179 0.930420505 -0.163723465 0.870989346
0.98757632 -7.760668147 cg13497069 6 29426119 p
GABBR1 OR2H1 NA TSS200 open sea TSS200 - open sea
NA 0.930661879 0.9299597 -0.000702179
1.000755064
-0.000297329 0.013824775 -0.163661754 0.871037527
0.98757632 -7.760678541 cg04852989 22 19974685 q
COMT ARVCF NA Body island Body - island
NA 0.013926982 0.013629652 -0.000297329
1.021814937
-0.000518838 0.064431366 -0.163514137 0.871152781
0.98757632 -7.760703387 cg01388957 9 4679439 p
SLC1A1 CDC37L1 NA TSS200 island TSS200 - island
NA 0.064609717 0.064090879 -0.000518838
1.008095348
-0.000751351 0.06396289 -0.162853163 0.871668881
0.98757632 -7.760814368 cg00858840 2 171573839 q
GAD1 SP5 NA Body island Body - island
NA 0.064221167 0.063469816 -0.000751351
1.011837926
-0.0004743 0.043520452 -0.161155436 0.872994757
0.98757632 -7.761097366 cg02996397 6 29720477 p
MOG IFITM4P 1893 IGR island IGR - island

NA 0.043683493 0.043209193 -0.0004743
1.010976831
-0.001347373 0.874944111 -0.160669649 0.873374212
0.98757632 -7.761177798 cg12344104 6 29693309 p
GABBR1/MOG HLA-F NA Body shore Body - shore
NA 0.875407271 0.874059897 -0.001347373
1.001541512
0.000344398 0.039483702 0.160413526 0.873574286
0.98757632 -7.761220107 cg00060933 6 29617602 p
GABBR1/MOG MOG -7156 IGR shore IGR - shore
NA 0.039365315 0.039709713 0.000344398
0.991327109
0.000966232 0.81478549 0.1579733 0.87548092 0.98757632 -7.761619828
cg25191596 6 29455126 p GABBR1 MAS1L NA
1stExon open sea 1stExon - open sea NA 0.814453348
0.81541958 0.000966232 0.998815049
-0.00053504 0.042937919 -0.156791385 0.876404665
0.98757632 -7.761811232 cg11209538 6 29600112 p
GABBR1/MOG GABBR1 NA Body shore Body - shore
NA 0.043121839 0.042586799 -0.00053504
1.012563518
0.000376545 0.057696382 0.155545871 0.877378307
0.98757632 -7.762011383 cg00063945 18 3450342 p
DLGAP1 TGIF1 NA 5'UTR island 5'UTR - island
NA 0.057566944 0.05794349 0.000376545
0.993501496
-0.000633122 0.117451856 -0.155286013 0.877581467
0.98757632 -7.762052941 cg25962210 11 27721222 p
BDNF BDNF NA Body shore Body - shore
NA 0.117669492 0.117036369 -0.000633122
1.005409626
-0.000552754 0.083514933 -0.154403899 0.878271179
0.98757632 -7.762193495 cg21619773 6 29720720 p
MOG IFITM4P 2136 IGR island IGR - island
NA 0.083704942 0.083152188 -0.000552754
1.006647498
-0.000248384 0.025770844 -0.153423173 0.879038109
0.98757632 -7.762348822 cg19339932 22 20004356 q
COMT ARVCF NA TSS200 island TSS200 - island
NA 0.025856226 0.025607841 -0.000248384
1.009699568
-0.000381295 0.035353148 -0.152782419 0.879539243
0.98757632 -7.762449771 cg24000444 18 3771452 p
DLGAP1 DLGAP1 NA Body island Body - island
NA 0.035484218 0.035102923 -0.000381295
1.010862201
0.000433351 0.084310798 0.152222368 0.879977301
0.98757632 -7.76253766 cg03789152 12 72233372 q
TPH2 TBC1D15 NA TSS200 island TSS200 - island
NA 0.084161833 0.084595184 0.000433351
0.994877356
-0.003527183 0.58521067 -0.152178832 0.880011355
0.98757632 -7.762544479 cg14791008 12 72332386 q
TPH2 TPH2 NA TSS1500 open sea TSS1500 - open
sea NA 0.58642314 0.582895956 -0.003527183
1.006051138

0.001123424 0.865587992 0.150510361 0.881316629
0.987960568 -7.762804328 cg16472542 22
20020575 q COMT C22orf25 NA 5'UTR open
sea 5'UTR - open sea NA 0.865201815 0.866325239
0.001123424 0.998703231
-0.000219727 0.032562667 -0.14989498 0.881798138
0.987960568 -7.762899446 cg00740645 6
29617868 p GABBR1/MOG MOG -6890 IGR island
IGR - island NA 0.032638198 0.032418471
-0.000219727 1.006777834
0.000630897 0.049408932 0.148129913 0.883179479
0.988698468 -7.763170109 cg02365078 12
72234032 q TPH2 TBC1D15 NA Body shore
Body - shore NA 0.049192061 0.049822958
0.000630897 0.987337223
-0.00084325 0.883884711 -0.142571725 0.887531728
0.992758302 -7.764001509 cg15313740 2
171625337 q GAD1 GAD1 -47863 IGR shore
IGR - shore NA 0.884174578 0.883331328
-0.00084325 1.000954625
-0.001995063 0.747550184 -0.137986153 0.891125069
0.994891145 -7.764663521 cg23717106 18 3768748
p DLGAP1 DLGAP1 NA Body shelf Body -
shelf NA 0.748235987 0.746240924 -0.001995063
1.002673484
-0.000328429 0.035167347 -0.135675381 0.892936727
0.994891145 -7.764988933 cg17625506 6
29691726 p GABBR1/MOG HLA-F NA Body island
Body - island NA 0.035280245 0.034951816
-0.000328429 1.009396622
-0.000853163 0.902967533 -0.135451148 0.893112558
0.994891145 -7.765020218 cg23600322 18 3457201
p DLGAP1 TGIF1 NA Body open sea Body -
open sea NA 0.903260808 0.902407645 -0.000853163
1.00094543
0.001774431 0.325214249 0.134960819 0.893497066
0.994891145 -7.765088449 cg11565911 12
72233249 q TPH2 TBC1D15 NA TSS1500 shore
TSS1500 - shore NA 0.324604289 0.32637872
0.001774431 0.994563276
0.000635696 0.088394722 0.133593456 0.89456947
0.994891145 -7.765277417 cg12801329 2
171670795 q GAD1 GAD1 -2405 IGR shore
IGR - shore NA 0.088176202 0.088811898
0.000635696 0.99284222
-0.000532006 0.034768988 -0.133008538 0.895028274
0.994891145 -7.765357665 cg07855056 17
28443892 q SLC6A4 MIR423 NA TSS1500 island
TSS1500 - island NA 0.034951866 0.034419859
-0.000532006 1.015456397
0.000292884 0.037670521 0.132859454 0.89514522
0.994891145 -7.765378062 cg03434929 6
29617848 p GABBR1/MOG MOG -6910 IGR island
IGR - island NA 0.037569842 0.037862726
0.000292884 0.992264582
0.000565167 0.088058161 0.130977253 0.896621881
0.994891145 -7.765633616 cg11215918 21

34395699 q OLIG2 OLIG2 -2517 IGR island
IGR - island NA 0.087863885 0.088429052
0.000565167 0.993608809
0.000428003 0.056792011 0.130731884 0.89681441
0.994891145 -7.765666662 cg19411146 6
152128471 q ESR1 ESR1 NA TSS1500 shore
TSS1500 - shore NA 0.056644885 0.057072888
0.000428003 0.992500765
-0.00073244 0.104570559 -0.130456504 0.897030495
0.994891145 -7.765703677 cg05119316 6
29716135 p MOG LOC285830 NA Body shore
Body - shore NA 0.104822335 0.104089895
-0.00073244 1.00703661
0.00068879 0.083324441 0.12818246 0.898815197 0.994891145
-7.766006356 cg24650785 11 27741916 p BDNF
BDNF NA Body shore Body - shore
V$CEBP_C;V$ZIC2_01 0.08308767 0.08377646 0.00068879 0.991778239
-0.000601361 0.907558261 -0.128054454 0.898915674
0.994891145 -7.766023236 cg12248981 6
29570707 p GABBR1/MOG GABBR1 NA 3'UTR open
sea 3'UTR - open sea NA 0.907764979 0.907163618
-0.000601361 1.000662902
0.0009744 0.862658027 0.127301435 0.899506782
0.994891145 -7.766122193 cg19372359 6
29575128 p GABBR1/MOG GABBR1 NA Body open
sea Body - open sea NA 0.862323077 0.863297477
0.0009744 0.998871304
-0.001663868 0.695559684 -0.127157041 0.899620136
0.994891145 -7.766141102 cg12508693 11
27657625 p BDNF BDNFOS NA Body open
sea Body - open sea NA 0.696131638 0.69446777 -
0.001663868 1.002395889
0.000496104 0.879541122 0.121673713 0.903926285
0.996469278 -7.766843293 cg25635805 6
29581054 p GABBR1/MOG GABBR1 NA Body open
sea Body - open sea NA 0.879370586 0.87986669
0.000496104 0.99943616
0.000792562 0.934963223 0.12129611 0.904222934
0.996469278 -7.766890511 cg12550837 6
29697949 p MOG LOC285830 NA Body open
sea Body - open sea NA 0.93469078 0.935483342
0.000792562 0.999152778
0.000691386 0.080068821 0.120916339 0.9045213
0.996469278 -7.766937852 cg12074493 17
28564117 q SLC6A4 SLC6A4 NA TSS1500 shore
TSS1500 - shore NA 0.079831157 0.080522543
0.000691386 0.991413759
0.001651806 0.297721993 0.12071254 0.904681419
0.996469278 -7.766963196 cg19352507 6
29717262 p MOG LOC285830 NA TSS1500 shore
TSS1500 - shore NA 0.297154185 0.298805991
0.001651806 0.994471978
0.000835926 0.844151031 0.120702621 0.904689213
0.996469278 -7.766964428 cg25562031 6
29624808 p GABBR1/MOG MOG NA 1stExon open
sea 1stExon - open sea V$HTF_01;V$CREB_02;V$XBP1_01;V$ATF_01;V$ATF6_01

0.843863681 0.844699607 0.000835926
0.999010387
-0.001742097 0.880582267 -0.118546523 0.906383456
0.996784057 -7.767229914 cg20704819 6
29585653 p GABBR1/MOG GABBR1 NA Body open
sea Body - open sea NA 0.881181113 0.879439016
-0.001742097 1.001980918
0.000677015 0.876273322 0.118484389 0.906432286
0.996784057 -7.767237494 cg02928278 6
29574918 p GABBR1/MOG GABBR1 NA Body open
sea Body - open sea NA 0.876040598 0.876717613
0.000677015 0.999227784
0.000823047 0.112980118 0.116027727 0.908363268
0.99759926 -7.767534006 cg01863613 17 28443583 q
SLC6A4 CCDC55 NA TSS1500 shore TSS1500 - shore
V$MEF2_01 0.112697195 0.113520243 0.000823047
0.992749769
0.000230726 0.017058997 0.115443153 0.908822838
0.99759926 -7.767603648 cg24899205 22 19929184 q
COMT TXNRD2 NA Body island Body - island
NA 0.016979685 0.01721041 0.000230726
0.98659387
-0.002084441 0.325716715 -0.114758261 0.909361314
0.99759926 -7.767684794 cg21156276 9 4491917 p
SLC1A1 SLC1A1 NA Body shore Body - shore
NA 0.326433242 0.324348801 -0.002084441
1.006426541
-0.000406316 0.088583269 -0.11296191 0.910773851
0.998327435 -7.767895334 cg00415702 6
29720841 p MOG IFITM4P 2257 IGR island
IGR - island NA 0.08872294 0.088316624 -
0.000406316 1.004600674
-0.000565176 0.909436904 -0.112058147 0.911484625
0.998327435 -7.768000004 cg06564500 22
19892663 q COMT TXNRD2 NA Body shore
Body - shore NA 0.909631183 0.909066007
-0.000565176 1.000621711
-0.000819494 0.072161967 -0.108620059 0.914189219
0.998903248 -7.768390511 cg01296653 9 4678949
p SLC1A1 CDC37L1 NA TSS1500 shore TSS1500
- shore NA 0.072443668 0.071624175 -0.000819494
1.01144157
-0.000682397 0.152795632 -0.108167406 0.914545379
0.998903248 -7.768441019 cg07476327 2
171730024 q GAD1 GORASP2 -54924 IGR open
sea IGR - open sea NA 0.153030206 0.152347809
-0.000682397 1.004479205
0.001049536 0.227444599 0.10526094 0.916832698
0.998903248 -7.768760306 cg13696752 9 4662858
p SLC1A1 C9orf68 NA Body island Body -
island V$CP2_01;V$E2F_03 0.227083821 0.228133357
0.001049536 0.995399463
-0.000421839 0.047267211 -0.105074113 0.916979751
0.998903248 -7.768780532 cg21034903 2
172544336 q SLC25A12 DYNC1I2 NA 5'UTR island
5'UTR - island V$STAT3_01;V$STAT1_01 0.047412218
0.046990379 -0.000421839 1.008977136

0.000165716 0.025954814 0.103076116 0.918552578
0.998903248 -7.768994596 cg10201663 6
29720685 p MOG IFITM4P 2101 IGR island
IGR - island NA 0.025897849 0.026063565
0.000165716 0.993641852
-0.000578176 0.911959427 -0.101263864 0.919979474
0.998903248 -7.769185207 cg15479387 6
29712425 p MOG LOC285830 NA Body open
sea Body - open sea NA 0.912158174 0.911579999
-0.000578176 1.000634256
-0.00083297 0.413925753 -0.101067331 0.920134233
0.998903248 -7.769205675 cg24302412 6
29595196 p GABBR1/MOG GABBR1 NA Body shore
Body - shore NA 0.414212086 0.413379116
-0.00083297 1.002015027
0.000187103 0.018085284 0.099854169 0.9210896
0.998903248 -7.769331141 cg05720454 21
34442511 q OLIG2 OLIG1 NA 1stExon island
1stExon - island NA 0.018020967 0.01820807
0.000187103 0.989724172
-0.000897544 0.906286319 -0.099827329 0.921110738
0.998903248 -7.7693339 cg10819807 6 29461549 p
GABBR1 MAS1L 7006 IGR open sea IGR - open sea
NA 0.906594849 0.905697305 -0.000897544
1.000990998
-0.000242543 0.040923441 -0.099359968 0.921478819
0.998903248 -7.769381818 cg04000205 18 3450626
p DLGAP1 TGIF1 NA 5'UTR island 5'UTR -
island NA 0.041006816 0.040764273 -0.000242543
1.005949891
-0.000811087 0.882637736 -0.098928775 0.92181843
0.998903248 -7.769425828 cg27527874 6
29571074 p GABBR1/MOG GABBR1 NA 3'UTR open
sea 3'UTR - open sea NA 0.882916547 0.88210546 -
0.000811087 1.00091949
0.000372327 0.077130309 0.098928488 0.921818656
0.998903248 -7.769425857 cg03770593 2
172750719 q SLC25A12 SLC25A12 NA 1stExon island
1stExon - island V$ER_Q6 0.077002322 0.077374649
0.000372327 0.995187998
-0.000154559 0.030943228 -0.098531324 0.922131479
0.998903248 -7.769466225 cg12912949 22
20004611 q COMT ARVCF NA TSS1500 island
TSS1500 - island NA 0.030996358 0.030841799
-0.000154559 1.005011348
-0.001107132 0.87466915 -0.093813638 0.925848278
0.998903248 -7.769933317 cg21022792 6
29589798 p GABBR1/MOG GABBR1 NA Body open
sea Body - open sea NA 0.875049727 0.873942595
-0.001107132 1.001266825
-0.000308057 0.037984142 -0.093738094 0.925907808
0.998903248 -7.76994061 cg06991510 11
27723237 p BDNF BDNF NA TSS1500 shore
TSS1500 - shore NA 0.038090036 0.03778198 -
0.000308057 1.008153517
0.001444524 0.680511525 0.093253765 0.926289485
0.998903248 -7.769987229 cg02824029 6

29570008 p GABBR1/MOG GABBR1 NA 3'UTR open
sea 3'UTR - open sea V$TCF11MAFG_01;V$BACH1_01;V$NFE2_01;V$AP1_01
0.68001497 0.681459493 0.001444524 0.997880251
0.000259081 0.041289365 0.092820485 0.926630946
0.998903248 -7.77002873 cg15688670 11
27723190 p BDNF BDNF NA TSS1500 shore
TSS1500 - shore NA 0.041200306 0.041459386
0.000259081 0.993750993
0.000310339 0.066643998 0.092457551 0.92691698
0.998903248 -7.770063344 cg08447405 17
28619272 q SLC6A4 BLMH NA TSS200 island
TSS200 - island NA 0.066537319 0.066847658
0.000310339 0.995357519
0.002252944 0.431587357 0.090955015 0.928101257
0.998903248 -7.770205204 cg17977304 6
29623992 p GABBR1/MOG MOG NA TSS1500 open
sea TSS1500 - open sea NA 0.430812908 0.433065852
0.002252944 0.994797687
-0.001130633 0.802393464 -0.090063546 0.928803978
0.998903248 -7.770288272 cg05309280 2
171785085 q GAD1 GORASP2 NA TSS1500 shore
TSS1500 - shore NA 0.802782119 0.801651486
-0.001130633 1.00141038
0.000910521 0.766165464 0.089304026 0.929402734
0.998903248 -7.7703584 cg14298020 6 29712462 p
MOG LOC285830 NA Body open sea Body - open sea
NA 0.765852472 0.766762993 0.000910521
0.998812513
0.002012846 0.302409291 0.089010988 0.929633757
0.998903248 -7.770385299 cg23019585 6
29635110 p GABBR1/MOG MOG NA 3'UTR open
sea 3'UTR - open sea NA 0.301717375 0.303730221
0.002012846 0.993372915
-0.000186769 0.047173172 -0.085567106 0.93234928
0.998903248 -7.770694793 cg24279419 6
29617791 p GABBR1/MOG MOG -6967 IGR island
IGR - island NA 0.047237373 0.047050604
-0.000186769 1.003969535
-0.000479065 0.888648401 -0.085076834 0.932735931
0.998903248 -7.770737861 cg18423469 18 3726858
p DLGAP1 DLGAP1 NA Body shelf Body -
shelf NA 0.88881308 0.888334015 -0.000479065
1.000539285
-0.000423713 0.116243667 -0.083522609 0.933961771
0.998903248 -7.770872754 cg02219071 6
29596540 p GABBR1/MOG GABBR1 NA TSS1500 shore
TSS1500 - shore NA 0.116389319 0.115965606
-0.000423713 1.003653782
0.001063597 0.231208524 0.082268166 0.934951289
0.998903248 -7.770979817 cg18342026 6
29521046 p GABBR1 UBD -2343 IGR shore
IGR - shore NA 0.230842913 0.23190651
0.001063597 0.995413682
-0.000263451 0.051682149 -0.081709306 0.935392158
0.998903248 -7.771026992 cg25122820 6
29716643 p MOG LOC285830 NA Body island

Body - island NA 0.051772711 0.05150926 -
0.000263451 1.005114634
-0.000275915 0.066758549 -0.081573447 0.935499336
0.998903248 -7.771038412 cg14665414 6
29596574 p GABBR1/MOG GABBR1 NA TSS1500 shore
TSS1500 - shore NA 0.066853395 0.06657748 -
0.000275915 1.004144269
0.00042702 0.922606855 0.08138926 0.935644643 0.998903248
-7.771053864 cg09646983 6 152125861 q ESR1
ESR1 NA 5'UTR shelf 5'UTR - shelf NA
0.922460067 0.922887087 0.00042702 0.9995373
0.001348561 0.480025747 0.081317137 0.935701542
0.998903248 -7.771059905 cg25583651 6
29570040 p GABBR1/MOG GABBR1 NA 3'UTR open
sea 3'UTR - open sea NA 0.479562179 0.48091074
0.001348561 0.997195819
0.000380581 0.919983184 0.081294031 0.93571977
0.998903248 -7.771061839 cg00923678 6
29581105 p GABBR1/MOG GABBR1 NA Body open
sea Body - open sea V$PAX5_01 0.919852359 0.92023294
0.000380581 0.99958643
-0.000438152 0.918142848 -0.08105885 0.93590531
0.998903248 -7.771081495 cg01350824 6
29550425 p GABBR1/MOG SNORD32B 396 IGR open
sea IGR - open sea NA 0.918293463 0.917855311
-0.000438152 1.000477365
-0.001405327 0.82802203 -0.080293418 0.936509204
0.998903248 -7.771145073 cg19103838 6
29562688 p GABBR1/MOG OR2H2 7005 IGR open
sea IGR - open sea NA 0.828505112 0.827099785
-0.001405327 1.001699102
-0.001032627 0.468349539 -0.079878098 0.936836891
0.998903248 -7.771179319 cg10635145 11
27742435 p BDNF BDNF NA Body shore
Body - shore NA 0.468704505 0.467671878
-0.001032627 1.002208016
-0.000636478 0.170770973 -0.078565499 0.937872601
0.998987273 -7.771286382 cg15617548 2
171785815 q GAD1 GORASP2 NA 5'UTR island
5'UTR - island NA 0.170989763 0.170353285
-0.000636478 1.003736224
-0.00047029 0.901969615 -0.077362824 0.938821672
0.998987273 -7.771382922 cg24444839 21
34350730 q OLIG2 OLIG2 -47486 IGR island
IGR - island NA 0.902131277 0.901660987
-0.00047029 1.000521582
0.000560748 0.130446776 0.07550011 0.940291782
0.998987273 -7.771529507 cg05218375 11
27723218 p BDNF BDNF NA TSS1500 shore
TSS1500 - shore NA 0.130254019 0.130814768
0.000560748 0.995713412
-0.001034979 0.362300805 -0.072078505 0.942992766
0.998987273 -7.771789462 cg23122901 22
19880135 q COMT TXNRD2 NA Body open
sea Body - open sea NA 0.362656579 0.3616216 -
0.001034979 1.00286205

0.000566533 0.89286491 0.071825024 0.943192889
0.998987273 -7.771808241 cg16580937 21
34347126 q OLIG2 OLIG2 -51090 IGR shelf
IGR - shelf NA 0.892670164 0.893236697
0.000566533 0.999365753
-0.000385146 0.884847022 -0.071211478 0.943677299
0.998987273 -7.77185342 cg07436579 6
29581117 p GABBR1/MOG GABBR1 NA Body open
sea Body - open sea V$PAX5_01 0.884979416 0.88459427 -
0.000385146 1.000435393
-0.000616106 0.88868534 -0.070630318 0.944136161
0.998987273 -7.771895858 cg05437995 6
29571875 p GABBR1/MOG GABBR1 NA Body open
sea Body - open sea NA 0.888897126 0.88828102 -
0.000616106 1.000693594
0.000119252 0.017520236 0.070091091 0.94456193
0.998987273 -7.771934923 cg15490715 6
29521568 p GABBR1 UBD -1821 IGR island
IGR - island NA 0.017479244 0.017598495
0.000119252 0.993223796
-0.000752836 0.752848782 -0.070089606 0.944563103
0.998987273 -7.77193503 cg25730428 6
29454755 p GABBR1 MAS1L NA 1stExon open
sea 1stExon - open sea NA 0.753107569 0.752354733
-0.000752836 1.00100064
-0.000692699 0.813813205 -0.06964935 0.944910738
0.998987273 -7.771966702 cg21632181 6
29700035 p MOG LOC285830 NA Body open
sea Body - open sea NA 0.81405132 0.813358621 -
0.000692699 1.000851653
0.000525942 0.840653005 0.069126736 0.94532342
0.998987273 -7.77200404 cg03258475 6
29577223 p GABBR1/MOG GABBR1 NA Body open
sea Body - open sea NA 0.840472212 0.840998155
0.000525942 0.999374621
-0.000182063 0.02744555 -0.066805577 0.947156502
0.998987273 -7.772166476 cg14589148 11
27743648 p BDNF BDNF NA TSS200 island
TSS200 - island NA 0.027508134 0.027326071
-0.000182063 1.006662612
0.000559713 0.264524983 0.065732776 0.948003822
0.998987273 -7.772239678 cg13620631 9
87489528 q NTRK2 NTRK2 NA 3'UTR open
sea 3'UTR - open sea NA 0.264332581 0.264892294
0.000559713 0.997887017
-0.000315477 0.88086643 -0.065068857 0.948528229
0.998987273 -7.772284386 cg06309882 6
29443400 p GABBR1 MAS1L -11143 IGR open
sea IGR - open sea NA 0.880974876 0.880659398
-0.000315477 1.000358229
0.000188584 0.035248363 0.064298979 0.94913636
0.998987273 -7.772335662 cg13709765 18 3451484
p DLGAP1 TGIF1 NA 5'UTR island 5'UTR -
island NA 0.035183538 0.035372122 0.000188584
0.99466857
-0.000135001 0.03818863 -0.06366644 0.949636029
0.998987273 -7.772377334 cg09503780 2

171786316 q GAD1 GORASP2 NA Body island
Body - island NA 0.038235037 0.038100035
-0.000135001 1.003543356
0.000591158 0.72264623 0.063522327 0.949749873
0.998987273 -7.77238677 cg09755181 6
29712115 p MOG LOC285830 NA Body open
sea Body - open sea NA 0.72244302 0.723034177
0.000591158 0.999182394
0.000212414 0.048795148 0.061509252 0.951340236
0.998987273 -7.772516353 cg05724110 21
34398532 q OLIG2 OLIG2 NA 5'UTR island
5'UTR - island NA 0.048722131 0.048934545
0.000212414 0.995659222
0.000362598 0.226693083 0.060109461 0.952446215
0.998987273 -7.772603999 cg08816824 6
29716244 p MOG LOC285830 NA Body shore
Body - shore NA 0.22656844 0.226931038
0.000362598 0.998402167
-0.000361825 0.893177502 -0.059831492 0.952665851
0.998987273 -7.772621163 cg21661983 12
72353490 q TPH2 TPH2 NA Body open
sea Body - open sea NA 0.893301879 0.892940054
-0.000361825 1.000405206
0.00048559 0.856087073 0.058447836 0.953759194
0.998987273 -7.772705421 cg13821571 21
34406537 q OLIG2 OLIG2 8321 IGR island
IGR - island NA 0.855920152 0.856405741
0.00048559 0.999432992
0.000339761 0.922084851 0.057741306 0.954317518
0.998987273 -7.772747685 cg17375177 6
29627845 p GABBR1/MOG MOG NA Body open
sea Body - open sea NA 0.921968058 0.92230782
0.000339761 0.999631618
0.00030763 0.154816721 0.057410448 0.954578982
0.998987273 -7.7727673 cg14738290 6 29690998 p
GABBR1/MOG HLA-F NA TSS200 shore TSS200 - shore
NA 0.154710973 0.155018603 0.00030763
0.998015528
-0.000773151 0.109093676 -0.0568267 0.955040307
0.998987273 -7.772801632 cg20801464 18 3454953
p DLGAP1 TGIF1 NA 5'UTR shelf 5'UTR -
shelf NA 0.109359447 0.108586296 -0.000773151
1.007120153
0.000104833 0.01876058 0.054920168 0.956547111
0.998987273 -7.77291132 cg12173216 6
29720762 p MOG IFITM4P 2178 IGR island
IGR - island NA 0.018724544 0.018829377
0.000104833 0.994432476
0.00026124 0.894860495 0.053511213 0.957660765
0.998987273 -7.772989976 cg24640415 6
29574953 p GABBR1/MOG GABBR1 NA Body open
sea Body - open sea V$ZID_01 0.894770693 0.895031934
0.00026124 0.999708121
0.00029228 0.839987084 0.051263801 0.959437325
0.998987273 -7.773111209 cg02039214 15
88521695 q NTRK3 NTRK3 NA Body open

sea Body - open sea NA 0.839886613 0.840178893
0.00029228 0.999652122
-0.000217692 0.121845027 -0.050497017 0.96004351
0.998987273 -7.773151383 cg08470639 9
87285186 q NTRK2 NTRK2 NA 5'UTR island
5'UTR - island NA 0.121919859 0.121702167
-0.000217692 1.001788727
-0.000183977 0.05392959 -0.050484216 0.96005363 0.998987273
-7.773152048 cg04991728 6 29720954 p MOG
IFITM4P 2370 IGR island IGR - island NA
0.053992832 0.053808855 -0.000183977
1.003419084
-0.000130041 0.084162779 -0.050470009 0.960064862
0.998987273 -7.773152786 cg13723118 9
87284722 q NTRK2 NTRK2 NA 5'UTR island
5'UTR - island NA 0.084207481 0.08407744 -
0.000130041 1.001546681
0.000128503 0.054960777 0.049566474 0.960779188
0.998987273 -7.773199323 cg03541635 22
20004063 q COMT ARVCF NA 5'UTR island
5'UTR - island NA 0.054916604 0.055045107
0.000128503 0.997665496
-0.000162781 0.062987136 -0.046208291 0.96343442
0.998987273 -7.773364916 cg06378770 6
29521714 p GABBR1 UBD -1675 IGR island
IGR - island NA 0.063043092 0.062880311
-0.000162781 1.002588744
0.000319783 0.723958991 0.044629235 0.964683089
0.998987273 -7.773438767 cg05114858 6
29526343 p GABBR1/MOG UBD NA Body open
sea Body - open sea NA 0.723849065 0.724168849
0.000319783 0.999558412
-0.000109397 0.023793861 -0.044481163 0.964800185
0.998987273 -7.77344556 cg11281641 2
171674855 q GAD1 GAD1 NA 5'UTR island
5'UTR - island NA 0.023831466 0.023722069
-0.000109397 1.004611613
-7.10E-05 0.010795183 -0.042408412 0.9664394 0.998987273
-7.773538288 cg11525479 15 88799523 q NTRK3
NTRK3 NA 5'UTR island 5'UTR - island NA
0.010819589 0.01074859 -7.10E-05 1.006605425
0.000250406 0.133797063 0.041555194 0.967114204
0.998987273 -7.773575173 cg21348357 2
171574592 q GAD1 SP5 2735 IGR shore
IGR - shore NA 0.133710986 0.133961392
0.000250406 0.99813076
-0.000222105 0.898916348 -0.040429673 0.968004407
0.998987273 -7.773622683 cg00237606 6
29455256 p GABBR1 MAS1L NA 1stExon open
sea 1stExon - open sea NA 0.898992697 0.898770591
-0.000222105 1.000247122
-0.000255983 0.155864232 -0.038629754 0.969428095
0.998987273 -7.773695949 cg24249411 11
27744759 p BDNF BDNF NA TSS1500 shore
TSS1500 - shore NA 0.155952226 0.155696244
-0.000255983 1.001644112

-0.000374806 0.885351971 -0.038517639 0.969516779
0.998987273 -7.773700402 cg13209878 6
29586025 p GABBR1/MOG GABBR1 NA Body open
sea Body - open sea NA 0.88548081 0.885106004 -
0.000374806 1.000423459
0.000281381 0.894144975 0.03820744 0.969762149
0.998987273 -7.773712656 cg21864378 6
29719180 p MOG IFITM4P NA TSS1500 shore
TSS1500 - shore NA 0.89404825 0.894329631
0.000281381 0.999685372
0.000178255 0.090820272 0.035724856 0.971726005
0.998987273 -7.773807158 cg03793625 2
171669862 q GAD1 GAD1 -3338 IGR shore
IGR - shore NA 0.090758996 0.090937252
0.000178255 0.998039791
-7.19E-05 0.017256204 -0.034152443 0.972969962
0.998987273 -7.77386373 cg23253569 21
34398222 q OLIG2 OLIG2 NA TSS200 island
TSS200 - island NA 0.017280909 0.017209039
-7.19E-05 1.004176294
-0.000452106 0.734023427 -0.034150193 0.972971742
0.998987273 -7.77386381 cg16518990 12
72244947 q TPH2 TBC1D15 NA Body open
sea Body - open sea NA 0.734178839 0.733726733
-0.000452106 1.000616178
0.000463891 0.501025451 0.032727207 0.974097544
0.998987273 -7.773912808 cg12292060 6
29597991 p GABBR1/MOG GABBR1 NA Body shelf
Body - shelf NA 0.500865989 0.50132988
0.000463891 0.999074679
-0.000114366 0.083430746 -0.031935979 0.974723551
0.998987273 -7.773939151 cg07063032 18 3450262
p DLGAP1 TGIF1 NA 5'UTR island 5'UTR -
island NA 0.083470059 0.083355693 -0.000114366
1.001372024
0.000338737 0.350438983 0.029922346 0.976316779
0.998987273 -7.774003285 cg04106006 11
27742454 p BDNF BDNF NA Body shore
Body - shore NA 0.350322542 0.350661279
0.000338737 0.999034005
0.000365231 0.583656103 0.029522876 0.97663286
0.998987273 -7.774015511 cg15999796 6
29623821 p GABBR1/MOG MOG NA TSS1500 open
sea TSS1500 - open sea NA 0.583530555 0.583895786
0.000365231 0.999374493
0.000195768 0.868503913 0.028575348 0.977382608
0.998987273 -7.774043856 cg20720118 6
29523976 p GABBR1 UBD NA Body shelf
Body - shelf NA 0.868436618 0.868632385
0.000195768 0.999774626
-8.15E-05 0.038783201 -0.027908148 0.977910554
0.998987273 -7.774063259 cg27254482 21
34398085 q OLIG2 OLIG2 NA TSS200 island
TSS200 - island NA 0.038811224 0.038729702
-8.15E-05 1.002104896
-0.000143858 0.890877863 -0.027825374 0.977976052
0.998987273 -7.774065635 cg24251942 15

88576320 q NTRK3 NTRK3 NA Body open
sea Body - open sea NA 0.890927314 0.890783456
-0.000143858 1.000161496
-0.000138102 0.893340694 -0.027427581 0.978290825
0.998987273 -7.774076951 cg10441070 6
152126250 q ESR1 ESR1 NA 5'UTR shelf
5'UTR - shelf NA 0.893388167 0.893250065
-0.000138102 1.000154606
5.83E-05 0.028563191 0.027184135 0.978483466
0.998987273 -7.774083797 cg27357571 21
34398226 q OLIG2 OLIG2 NA TSS200 island
TSS200 - island NA 0.028543138 0.028601475
5.83E-05 0.99796035
0.000273711 0.88425942 0.02619711 0.979264519 0.998987273
-7.774110925 cg06898502 18 3410587 p DLGAP1
TGIF1 NA TSS1500 open sea TSS1500 - open sea NA
0.884165332 0.884439043 0.000273711
0.999690526
-0.000207325 0.131607616 -0.026119652 0.979325815
0.998987273 -7.774113012 cg00729049 2
171679402 q GAD1 GAD1 NA Body island
Body - island NA 0.131678884 0.131471559
-0.000207325 1.001576957
0.000192662 0.870526264 0.025788323 0.979588007
0.998987273 -7.774121867 cg16101636 6
29711438 p MOG LOC285830 NA Body open
sea Body - open sea NA 0.870460036 0.870652699
0.000192662 0.999778714
0.000208088 0.778495315 0.025096675 0.98013534
0.998987273 -7.774139988 cg01393604 6
29695590 p GABBR1/MOG LOC285830 NA Body shelf
Body - shelf NA 0.778423785 0.778631872
0.000208088 0.999732753
0.000137903 0.904985571 0.024282934 0.980779305
0.998987273 -7.774160678 cg11348701 2
171704223 q GAD1 GAD1 NA Body open
sea Body - open sea V$FREAC2_01 0.904938167
0.90507607 0.000137903 0.999847634
-5.40E-05 0.029489507 -0.024248905 0.980806234
0.998987273 -7.774161528 cg20563534 22
20004369 q COMT ARVCF NA TSS200 island
TSS200 - island NA 0.029508068 0.029454071
-5.40E-05 1.001833261
-7.55E-05 0.050372099 -0.023163441 0.98166525 0.998987273
-7.774188026 cg06581978 21 34408891 q OLIG2
OLIG2 10675 IGR shelf IGR - shelf NA
0.05039806 0.050322537 -7.55E-05 1.001500779
-0.000141979 0.859347923 -0.022885494 0.981885216
0.998987273 -7.774194616 cg21080452 6
29589960 p GABBR1/MOG GABBR1 NA Body open
sea Body - open sea NA 0.859396728 0.85925475 -
0.000141979 1.000165234
-0.000376285 0.778557235 -0.021388737 0.983069767
0.998987273 -7.774228737 cg15018934 6
29689744 p GABBR1/MOG HLA-F NA TSS1500 shore
TSS1500 - shore NA 0.778686582 0.778310298
-0.000376285 1.000483463

9.93E-05 0.91964656 0.019031311 0.984935538 0.998987273
-7.774277799 cg12710376 15 88495888 q NTRK3
NTRK3 NA Body open sea Body - open sea NA
0.919612427 0.919711723 9.93E-05 0.999892036
-2.85E-05 0.014734085 -0.016992168 0.986549476
0.998987273 -7.774315622 cg17137980 22
19842443 q COMT GNB1L NA 1stExon island
1stExon - island NA 0.014743886 0.014715375
-2.85E-05 1.001937497
-0.00027983 0.821786409 -0.016903672 0.98661952
0.998987273 -7.774317166 cg15180617 6
29705436 p MOG LOC285830 NA Body open
sea Body - open sea NA 0.821882601 0.821602771
-0.00027983 1.00034059
-0.000171556 0.775777579 -0.016263269 0.987126399
0.998987273 -7.774328103 cg20103692 6
29454672 p GABBR1 MAS1L NA 1stExon open
sea 1stExon - open sea NA 0.775836551 0.775664995
-0.000171556 1.000221173
-8.75E-05 0.091618632 -0.016220741 0.98716006 0.998987273
-7.774328814 ch.2.3493243F 2 172737063 q
SLC25A12 SLC25A12 NA Body open sea Body - open sea
NA 0.091648696 0.091561239 -8.75E-05
1.000955175
-0.00012762 0.184921035 -0.016166149 0.987203269
0.998987273 -7.774329724 cg06212263 21
34392851 q OLIG2 OLIG2 -5365 IGR shore
IGR - shore NA 0.184964905 0.184837284
-0.00012762 1.000690451
0.000156365 0.125312542 0.014992525 0.988132207
0.998987273 -7.774348553 cg21346639 18 3447463
p DLGAP1 TGIF1 NA 5'UTR shore 5'UTR -
shore NA 0.125258792 0.125415156 0.000156365
0.998753229
2.46E-05 0.022007839 0.013509155 0.989306336
0.998987273 -7.774370322 cg24399395 21
34392203 q OLIG2 OLIG2 -6013 IGR island
IGR - island NA 0.021999366 0.022024014
2.46E-05 0.998880858
-6.82E-05 0.797411279 -0.012039258 0.990469825
0.998987273 -7.774389658 cg13473117 22
19867665 q COMT TXNRD2 NA Body open
sea Body - open sea NA 0.797434736 0.797366497
-6.82E-05 1.00008558
9.10E-05 0.855385649 0.011008107 0.99128604 0.998987273
-7.774401895 cg24177217 6 29702053 p MOG
LOC285830 NA Body open sea Body - open sea NA
0.855354357 0.855445387 9.10E-05 0.999893588
-0.000265605 0.665302082 -0.010871518 0.991394159
0.998987273 -7.774403434 cg00601836 6
152130332 q ESR1 ESR1 NA Body shore
Body - shore NA 0.665393384 0.665127779
-0.000265605 1.000399329
-2.86E-05 0.036571113 -0.010426095 0.99174674 0.998987273
-7.774408318 cg21010859 11 27740161 p BDNF
BDNF NA Body shore Body - shore NA
0.036580942 0.03655235 -2.86E-05 1.000782221

-8.18E-05 0.8350989 -0.010167398 0.991951516 0.998987273
-7.774411061 cg101391516 29549352 p
GABBR1/MOG SNORD32B NA TSS1500 open sea TSS1500 - open
sea NA 0.835127008 0.835045241 -8.18E-05
1.000097919
4.19E-05 0.88280104 0.007913847 0.993735377 0.998987273
-7.774432042 cg13206902 6 29523786 p GABBR1
UBD NA Body shore Body - shore NA
0.882786637 0.882828535 4.19E-05 0.999952541
4.10E-05 0.904258979 0.007718494 0.993890016
0.998987273 -7.774433614 cg06538238 6
29588575 p GABBR1/MOG GABBR1 NA Body open
sea Body - open sea NA 0.904244888 0.90428588 4.10E-
05 0.999954669
-4.45E-05 0.884380171 -0.006929155 0.994514848
0.998987273 -7.774439567 cg14093720 18 3712400
p DLGAP1 DLGAP1 NA Body open sea Body -
open sea NA 0.884395482 0.88435094 -4.45E-05 1.000050367
-5.61E-05 0.917988377 -0.005782122 0.995422833
0.998987273 -7.774447075 cg10503635 6
29634213 p GABBR1/MOG MOG NA 3'UTR open
sea 3'UTR - open sea NA 0.918007678 0.91795153 -5.61E-
05 1.000061167
3.48E-05 0.900547859 0.005376602 0.995743842
0.998987273 -7.774449405 cg18868933 6
29708164 p MOG LOC285830 NA Body open
sea Body - open sea NA 0.900535882 0.900570724
3.48E-05 0.999961311
-2.27E-05 0.049658214 -0.0049968 0.996044494 0.998987273
-7.774451433 cg12448003 11 27742365 p BDNF
BDNF NA Body shore Body - shore NA
0.049666017 0.049643316 -2.27E-05 1.000457282
6.87E-06 0.016545704 0.004465437 0.996465122
0.998987273 -7.774454022 cg23467008 6
152128537 q ESR1 ESR1 NA TSS1500 shore
TSS1500 - shore NA 0.016543343 0.016550212
6.87E-06 0.99958496
9.21E-06 0.042413323 0.003105541 0.997541623
0.998987273 -7.774459323 cg15814717 6
29521228 p GABBR1 UBD -2161 IGR island
IGR - island NA 0.042410157 0.042419368
9.21E-06 0.999782859
-8.46E-06 0.045209408 -0.002000952 0.998416025
0.998987273 -7.774462228 cg04661128 9 4661938
p SLC1A1 PPAPDC2 NA TSS1500 shore TSS1500
- shore NA 0.045212316 0.045203857 -8.46E-06
1.00018713
-5.68E-06 0.056996963 -0.001777557 0.998592866
0.998987273 -7.774462662 cg18588589 6
29521705 p GABBR1 UBD -1684 IGR island
IGR - island NA 0.056998916 0.056993236
-5.68E-06 1.000099661
-4.41E-06 0.077736957 -0.001279324 0.998987273
0.998987273 -7.774463446 cg21569006 21
34443672 q OLIG2 OLIG1 NA 1stExon island
1stExon - island NA 0.077738474 0.077734062
-4.41E-06 1.000056758
